# Construction and validation of a novel gene signature for predicting the prognosis of osteosarcoma

**DOI:** 10.1038/s41598-022-05341-5

**Published:** 2022-01-24

**Authors:** Jinpo Yang, Anran Zhang, Huan Luo, Chao Ma

**Affiliations:** 1 Department of Medical Oncology, The Affiliated Cancer Hospital of Zhengzhou University, Henan Cancer Hospital, Zhengzhou, China; 2 Department of Oncology, Henan Provincial People’s Hospital, Zhengzhou University People’s Hospital, Henan University People’s Hospital, Zhengzhou, China; 3 Charité – Universitätsmedizin Berlin, corporate member of Freie Universität Berlin, Humboldt-Universität zu Berlin, and the Berlin Institute of Health, Berlin, Germany

**Keywords:** Bone cancer, Cancer microenvironment, Cancer models, Cancer prevention, Cancer therapy, Tumour biomarkers, Tumour immunology, Oncology, Cancer, Bone cancer, Cancer metabolism, Cancer microenvironment, Cancer models, Cancer therapy, Tumour biomarkers, Tumour heterogeneity, Tumour immunology

## Abstract

Osteosarcoma (OS) is the most common type of primary malignant bone tumor. The high-throughput sequencing technology has shown potential abilities to illuminate the pathogenic genes in OS. This study was designed to find a powerful gene signature that can predict clinical outcomes. We selected OS cases with gene expression and survival data in the TARGET-OS dataset and GSE21257 datasets as training cohort and validation cohort, respectively. The univariate Cox regression and Kaplan–Meier analysis were conducted to determine potential prognostic genes from the training cohort. These potential prognostic genes underwent a LASSO regression, which then generated a gene signature. The harvested signature’s predictive ability was further examined by the Kaplan–Meier analysis, Cox analysis, and receiver operating characteristic (ROC curve). More importantly, we listed similar studies in the most recent year and compared theirs with ours. Finally, we performed functional annotation, immune relevant signature correlation identification, and immune infiltrating analysis to better study he functional mechanism of the signature and the immune cells’ roles in the gene signature’s prognosis ability. A seventeen-gene signature (*UBE2L3, PLD3, SLC45A4, CLTC, CTNNBIP1, FBXL5, MKL2, SELPLG, C3orf14, WDR53, ZFP90, UHRF2, ARX, CORT, DDX26B, MYC, and SLC16A3*) was generated from the LASSO regression. The signature was then confirmed having strong and stable prognostic capacity in all studied cohorts by several statistical methods. We revealed the superiority of our signature after comparing it to our predecessors, and the GO and KEGG annotations uncovered the specifically mechanism of action related to the gene signature. Six immune signatures, including *PRF1, CD8A, HAVCR2, LAG3, CD274,* and *GZMA* were identified associating with our signature. The immune-infiltrating analysis recognized the vital roles of T cells CD8 and Mast cells activated, which potentially support the seventeen-gene signature’s prognosis ability. We identified a robust seventeen-gene signature that can accurately predict OS prognosis. We identified potential immunotherapy targets to the gene signature. The T cells CD8 and Mast cells activated were identified linked with the seventeen-gene signature predictive power.

## Introduction

Osteosarcoma (OS) is a bone tumor that occurs predominantly in adolescents and young adults^1–3^. In the 0–24 age group, the incidence of osteosarcoma among men, women and children is 4.4 per million persons per year^1–3^. The latest advances in molecular genetics of osteosarcoma have changed our views on the cause of the disease and the continued treatment of patients^1–3^. Surgical removal of clinically visible tumors and systemic chemotherapy are currently popular disease management strategies^1–3^. Although the cure rate for patients with local disease is close to 70%, the 5-year overall survival rate for patients with metastatic disease is less than 25%, and most patients die from lung metastases^4^. Unfortunately, the treatment paradigm for OS has remained unchanged for approximately 30 years^1,4^. Therefore, continued efforts are urgently needed for a steady prognostic model for OS patients.

The rise of throughput sequencing technology helps clarify disease-causing genes, explore disease pathogenesis, develop biomarkers, and profoundly change our understanding of biology and human diversity^5^. Researchers have developed many statistical models that use genomic data to accurately predict whether the prognostic risk of cancer patients is high or low^6–10^. Many researchers have screened multiple biomarkers related to OS by mining gene expression data^5^. Gene signature can contain more than one single gene with a unique characteristic pattern of gene expression resulting from an altered or unaltered biological process or pathogenic medical condition^11^. Gene signature has a more stable ability and higher fault tolerance for prognostic prediction in cancer studies^7–12^.

Finding multiple molecules from the OS gene profile to construct a gene signature can better predict outcome potentially. To fill in the void and find a promising gene signature that targets OS outcomes, this work tried to identify a prognostic gene signature from the TARGET database. More importantly, the signature we found was further tested in an independent dataset for its prognostic ability and was compared to the models built in the most recent year for its superiority. In the end, the functional annotation, immune relevant signature correlation analysis, and 22 tumor-infiltrating immune cells (TICs) analysis were conducted for the full understanding of the gene signature we discovered.

## Materials and methods

### Database selection

The Therapeutically Applicable Research to Generate Effective Treatments (TARGET) is a dynamically updated database of the National Cancer Institute (NCI) Office of Cancer Genomics (OCG). Its mission is to advance the molecular understanding of cancer to improve patient prognosis^13^. The TARGET Osteosarcoma (TARGET-OS) project has elucidated a comprehensive molecular profile to identify the genetic changes that drive the occurrence and development of high-risk or difficult-to-treat childhood cancers. OS datasets are available without restrictions on their use in publications or presentations and can be obtained from the official web patrol (https://ocg.cancer.gov/programs/target/projects/osteosarcoma) or GDC Xena Hub (https://gdc.xenahubs.net). TARGET-OS was set as training cohort. Eighty-eight OS cases were included, and their gene expression profile, survival time, survival status, and clinical characteristics were obtained. Gene Expression Omnibus (GEO) is an internationally recognized and widely researched public repository for archiving and free distribution of microarray^14^. We searched the GEO using the keyword “Osteosarcoma” and set the filters as follows: (1) organisms: homo sapiens; (2) entry type: series; (3) study type: expression profiling by array; (4) the number of samples with expression data is greater than 50; (5) the number of samples with survival data is greater than 50. One dataset named GSE21257 (n = 53) was obtained from GEO (https://www.ncbi.nlm.nih.gov/geo/query/acc.cgi?acc=GSE21257) and treated as validation cohorts to exam the gene signature we constructed. We strictly obeyed the guidelines of the two databases. This study was approved by the Institutional Review Board of Henan Cancer Hospital, which waived the requirement for informed consent due to the use of data obtained from the public databases. All methods were performed in accordance with relevant guidelines and regulations.

### Identification of the potential prognostic genes

We implemented a univariate Cox proportional hazard model and Kaplan–Meier estimator to identify genes with potential prognostic ability. In our research, gene expression, survival status, and survival time were input into the R language. With the help of the “survival” and “survminer” R packages, Kaplan–Meier estimator could sort out genes with the ability to distinguish patients' outcomes. The Kaplan–Meier significance threshold was set to p < 0.05. Similarly, each gene’s univariate Cox model was built using the gene expression data, survival status, survival time, and the adoption of the “survival” R package. The univariate Cox model significance threshold was set to p < 0.05. The gene in both tests having a p value < 0.05 was considered the potential prognostic gene.

### Gene signature construction and validation

Subsequently, we put the potential prognostic genes identified in the previous step into the LASSO model to detect the best lambda^15–18^. Specifically, we utilized the expression data of the potential prognostic genes, patients’ survival data, and the "glmnet" R package to perform the LASSO Cox regression with tenfold cross-validation. Then the R program outputted a list of prognostic genes with coefficients based on the best lambda value selected. According to the instructions and characteristics of the "glmnet" R software package, the selected genes with coefficients would be out putted. The calculation method of the risk score level of each OS is using the following formula:

$$Risk score={\sum }_{i}^{n}Expi*\beta i$$

In the above formula, n represents each hub gene in the gene signature; Expi represents the expression level of each gene; βi represents the coefficient of each gene.

To test our signature's ability in all studied cohorts, the Kaplan–Meier analysis was used to determine the outcome differences between high- and low-risk patients, of which the OS were classified according to the median risk score. In addition, univariate and multivariable Cox analyses further examined the predictive potential of the gene signature. The area under the curve (AUC) is a measure of the classifier's ability to distinguish classes and is used as a summary of the ROC curve^19^. The higher the AUC, the better the model's performance in determining between positive and negative classes.

### Comparison of gene signature with previously published models

We searched PubMed (https://pubmed.ncbi.nlm.nih.gov/) using the keyword "gene signature prognosis osteosarcoma" and made the selection based on criteria we set as follows: (1) the impact factor > 4 (Journal Citation Reports Year 2020, Clarivate, https://jcr.clarivate.com/jcr/home); (2) the online publication date of the article is the most recent year (i.e. May 18, 2020, to May 18, 2021); (3) the candidate study contains specifically findings of the signature’s composition and coefficients. We extracted the gene signatures and the coefficients from the studies and applied them to the studied cohorts to calculate the risk score of each case. The most important thing was that we used the risk scores to build Kaplan–Meier analysis and Cox model to strictly assess the prognostic ability of our predecessors and ours, thus for horizontal comparison.

### Function analysis of the gene signature in OS

Gene ontology (GO), including Biological Process (BP), Cellular Components (CC), and Molecular Functions (MF), and Kyoto Encyclopedia of Genes and Genomes (KEGG) were conducted to find the potential function of genes between high- and low-risk groups^20–22^. Enrich items with p value < 0.05 were considered significant.

### Correlations between gene signature and immune relevant signatures

We analyzed the immune activity and tolerance of low- and high-risk groups in the training cohort. Firstly, we picked *CD274, CTLA4, HAVCR2, IDO1, LAG3*, and *PDCD1* as immune-checkpoint-relevant signatures, and *CD8A, CXCL10, CXCL9, GZMA, GZMB, IFNG, PRF1, TBX2,* and *TNF* as immune-activity-relevant signatures. We adopted an integrated analysis including the Pearson correlation coefficient and Wilcoxon rank-sum to determine the interaction between gene signature and immune relevant signatures.

### Determine the relationships between our signature and 22 TICs

We applied a comprehensive analysis based on the Pearson coefficient and Wilcoxon's rank-sum test to evaluate the relationship between 22 TICs and the signatures of this study. In the following analysis, in order to determine the prognostic ability of the 22 TICs, we combined two kinds of statistical approaches, including univariate Cox models and Kaplan–Meier analysis. Together with the evidence found in the first half of this section, we could infer the potential TICs that play crucial roles in the signature’s prognosis ability.

### Statistical analysis

We adopt the "CIBERSORT" R package to estimate the abundance of 22 TICs using the gene expression data of the cohorts. LASSO regression was carried out by the "glmnet" R package. Kaplan–Meier plots were constructed by the integration of the "survival" and "survminer" R packages. Cox models, including univariate and multivariable were built via the "survival" R package. The ROC curves was made possible with the help of the “pROC” R package. R software (version 4.0.4, Windows 64-bit) carried out all the processes in this study.

## Results

### Cohorts’ characteristics

**Figure 1:**
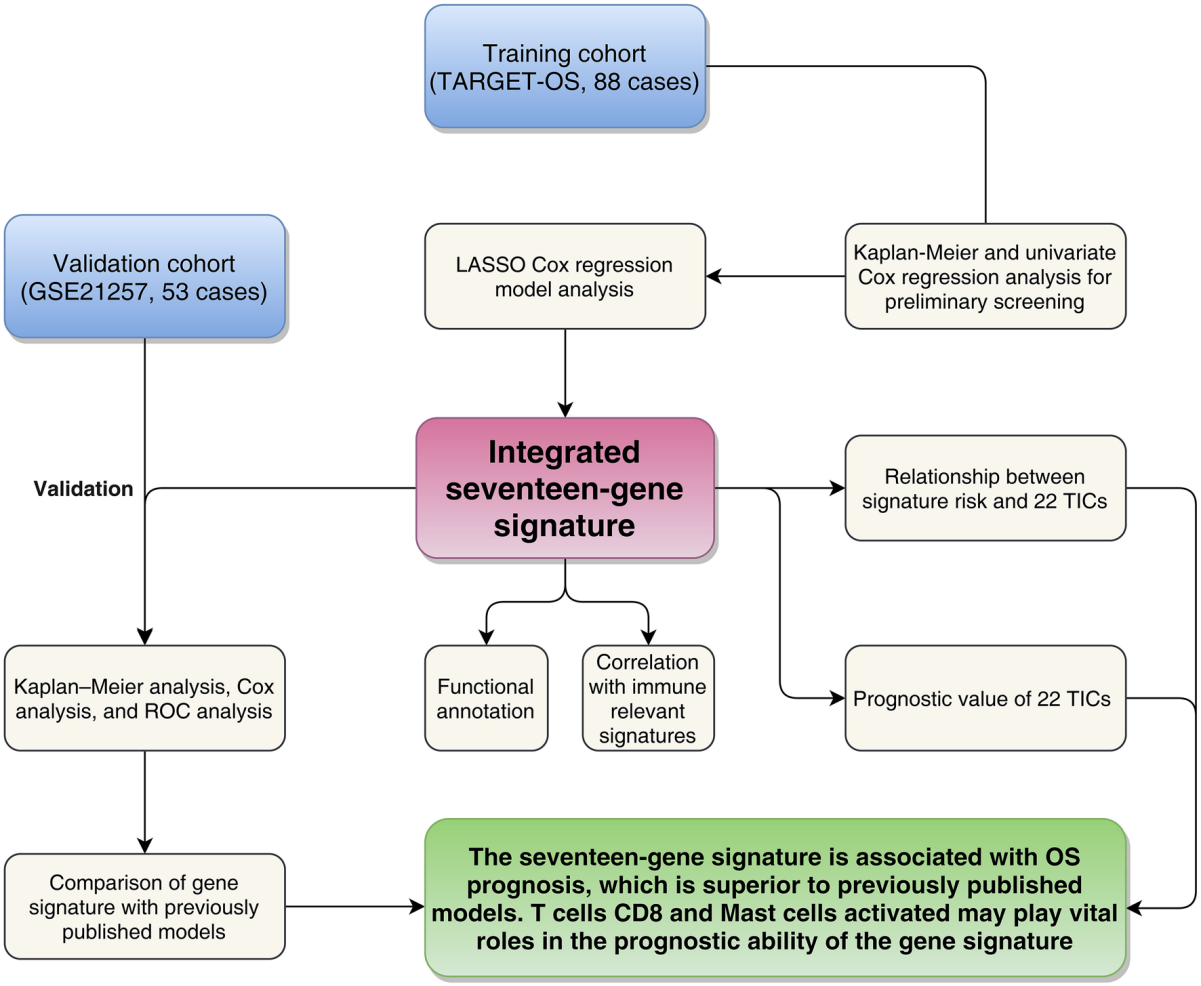
Flow chart of the study. *LASSO* least absolute shrinkage and selection operator Cox regression model, *ROC* receiver operating characteristic, *OS* osteosarcoma, *TICs* tumor-infiltrating immune cells.

As Fig. 1 demonstrates, 88 OS cases that came from the TARGET-OS cohort were taken for model training. The dataset GSE21257, contained 53 OS cases, were selected for model validating. For patients included in the study, we have collected their clinical characteristics and shown them in Table 1 in detail.

**Table 1:** Clinical characteristics of patients involved in the study.

Characteristics	Training cohort (TARGET-OS, n = 88)	Validation cohort (GSE21257, n = 53)
**Age**		
< 14	39 (44.32%)	15 (28.3%)
≥ 14	45 (51.14%)	38 (71.7%)
Unknown	4 (4.55%)	0
**Gender**		
Female	37 (42.05%)	19 (35.85%)
Male	47 (53.41%)	34 (64.15%)
Unknown	4 (4.55%)	0
**Race**		
Non-White	13 (14.77%)	NA
White	51 (57.95%)	NA
Unknown	24 (27.27%)	NA
**Ethnicity**		
Not Hispanic or Latino	52 (59.09%)	NA
Hispanic or Latino	11 (12.5%)	NA
Unknown	25 (28.41%)	NA
**Tumor location**		
Femur	NA	27 (50.94%)
Fibula	NA	2 (3.77%)
Humerus	NA	8 (15.09%)
Tibia	NA	15 (28.3%)
Unknown	NA	1 (1.89%)
**Histological subtype**		
Chondroblastic	NA	6 (11.32%)
Fibroblastic	NA	5 (9.43%)
Osteoblastic	NA	32 (60.38%)
Others	NA	10 (18.87%)
**Metastatic status**		
Non-metastatic	63 (71.59%)	39 (73.58%)
Metastatic	21 (23.86%)	14 (26.42%)
Unknown	4 (4.55%)	0
**Survival status**		
Alive	58 (65.91%)	30 (56.6%)
Dead	27 (30.68%)	23 (43.4%)
Unknown	3 (3.41%)	0

### Prognostic gene signature identification

**Figure 2:**
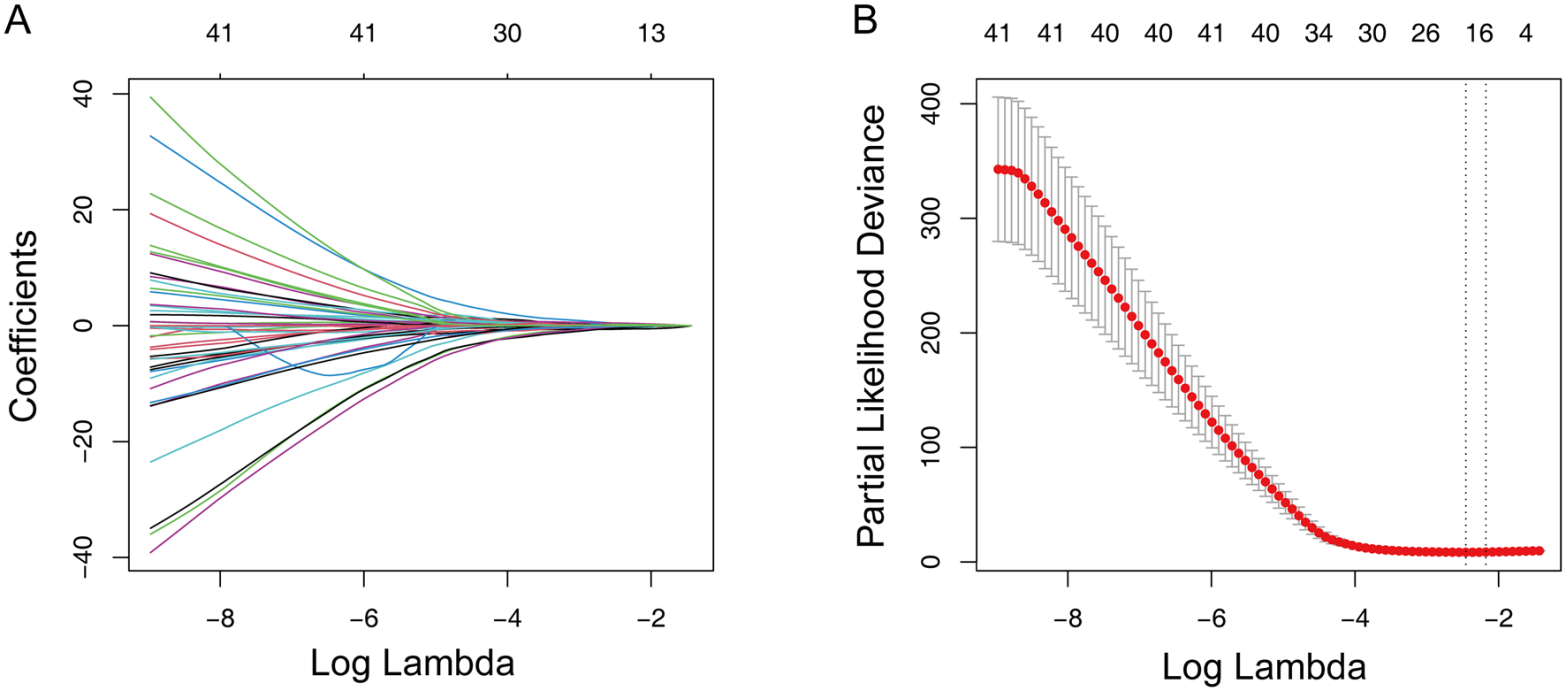
LASSO regression analysis for the construction of prognostic gene signature. (**A**) Cross-validation for tuning parameter screening upon LASSO regression analysis. (**B**) Screening of optimal parameter (lambda) at which the vertical lines were drawn. *LASSO* the least absolute shrinkage and selection operator Cox regression model.

The univariate Cox regression and Kaplan–Meier analysis were conducted to test each gene’s prognostic ability. As shown in Table S1, 70 genes were identified by the Kaplan–Meier estimator, while, 80 genes were determined from the univariate Cox regression model, which has the predictive ability. We intersected them, found 57 genes suitable for our study, and included them in our next analyses (Table S2). The LASSO algorithm displayed when 17 genes existed, the model could achieve the optimized ability (Fig. 2A,B). Table 2 shows the coefficients of the 17 genes.

**Table 2:** Prognostic genes obtained from LASSO Cox regression model.

Gene symbol	Description	Risk coefficient
*C3orf14*	Chromosome 3 Open Reading Frame 14	− 0.106872216
*UHRF2*	Ubiquitin Like With PHD And Ring Finger Domains 2	0.321564173
*DDX26B*	Integrator Complex Subunit 6 Like	0.334217953
*ZFP90*	ZFP90 Zinc Finger Protein	− 0.505473926
*FBXL5*	F-Box And Leucine Rich Repeat Protein 5	− 0.24934174
*UBE2L3*	Ubiquitin Conjugating Enzyme E2 L3	− 0.308369838
*MYC*	MYC Proto-Oncogene, BHLH Transcription Factor	0.250664103
*CLTC*	Clathrin Heavy Chain	− 0.356348745
*ARX*	Aristaless Related Homeobox	0.444234486
*CTNNBIP1*	Catenin Beta Interacting Protein 1	− 0.601488248
*CORT*	Cortistatin	0.220902785
*SELPLG*	Selectin P Ligand	− 0.070201073
*WDR53*	WD Repeat Domain 53	− 0.059234551
*SLC16A3*	Solute Carrier Family 16 Member 3	0.002731127
*MKL2*	Myocardin Related Transcription Factor B	− 0.028375916
*SLC45A4*	Solute Carrier Family 45 Member 4	− 0.156290246
*PLD3*	Phospholipase D Family Member 3	− 0.128837662

### Confirmation of the prognostic capacity of the seventeen-gene signature

**Figure 3:**
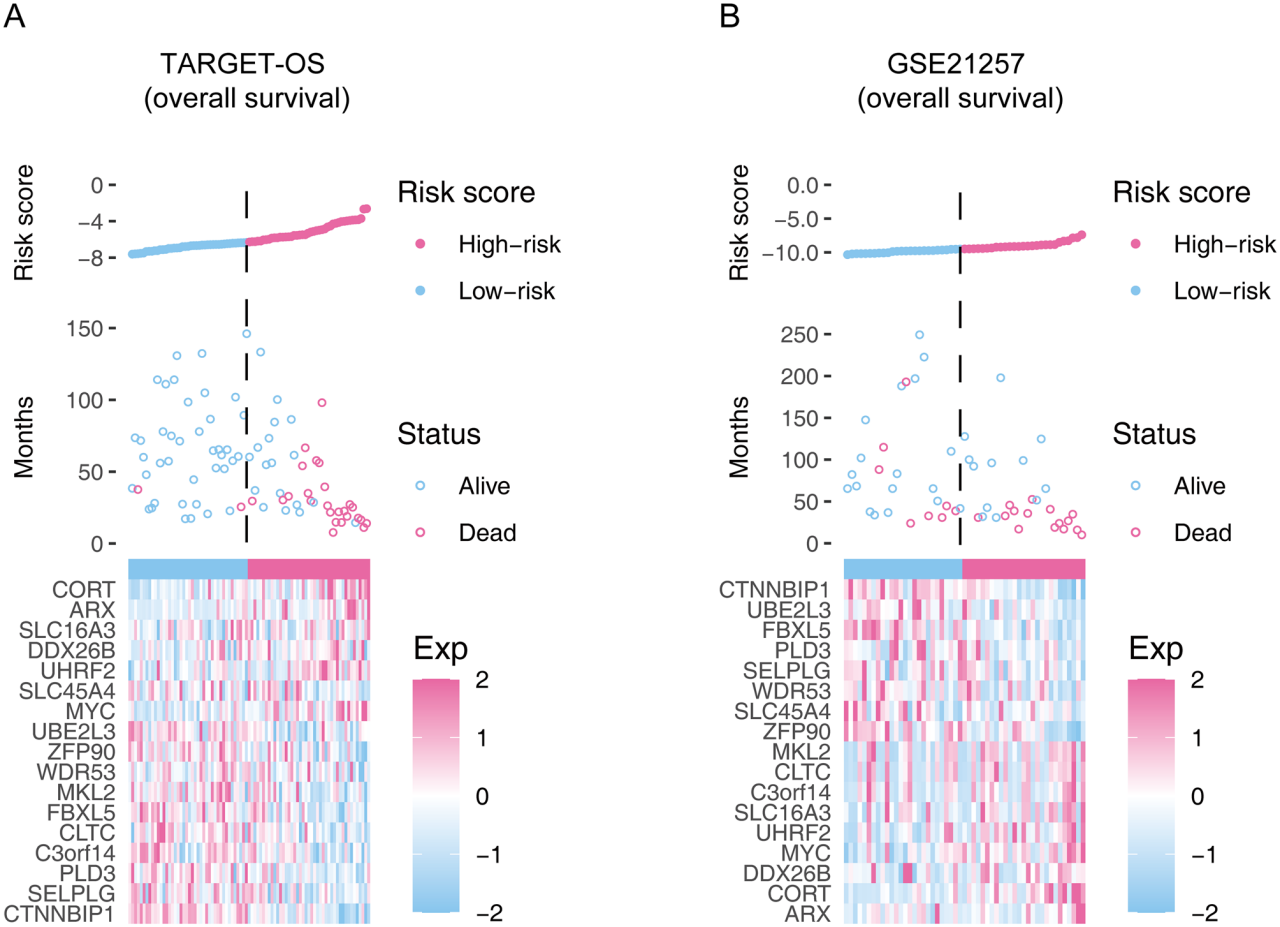
The overall distributions of the risk score (upper), survival status (middle), and gene expression profiles (bottom) of the seventeen-gene signature in the training (**A**) and validation (**B**) cohorts.

In the risk plot in Fig. 3, we displayed the survival time, survival status, and relative expression of the hub genes for each sample, so as to show the distinguishing ability of the signature in a macroscopic view. In the training cohort, *UBE2L3, PLD3, SLC45A4, CLTC, CTNNBIP1, FBXL5, MKL2, SELPLG, C3orf14, WDR53,* and *ZFP90* have protective abilities for OS patients, while *UHRF2, ARX, CORT, DDX26B, MYC,* and *SLC16A3* display unfavored for the OS prognosis (Figure S1A).

**Figure 4:**
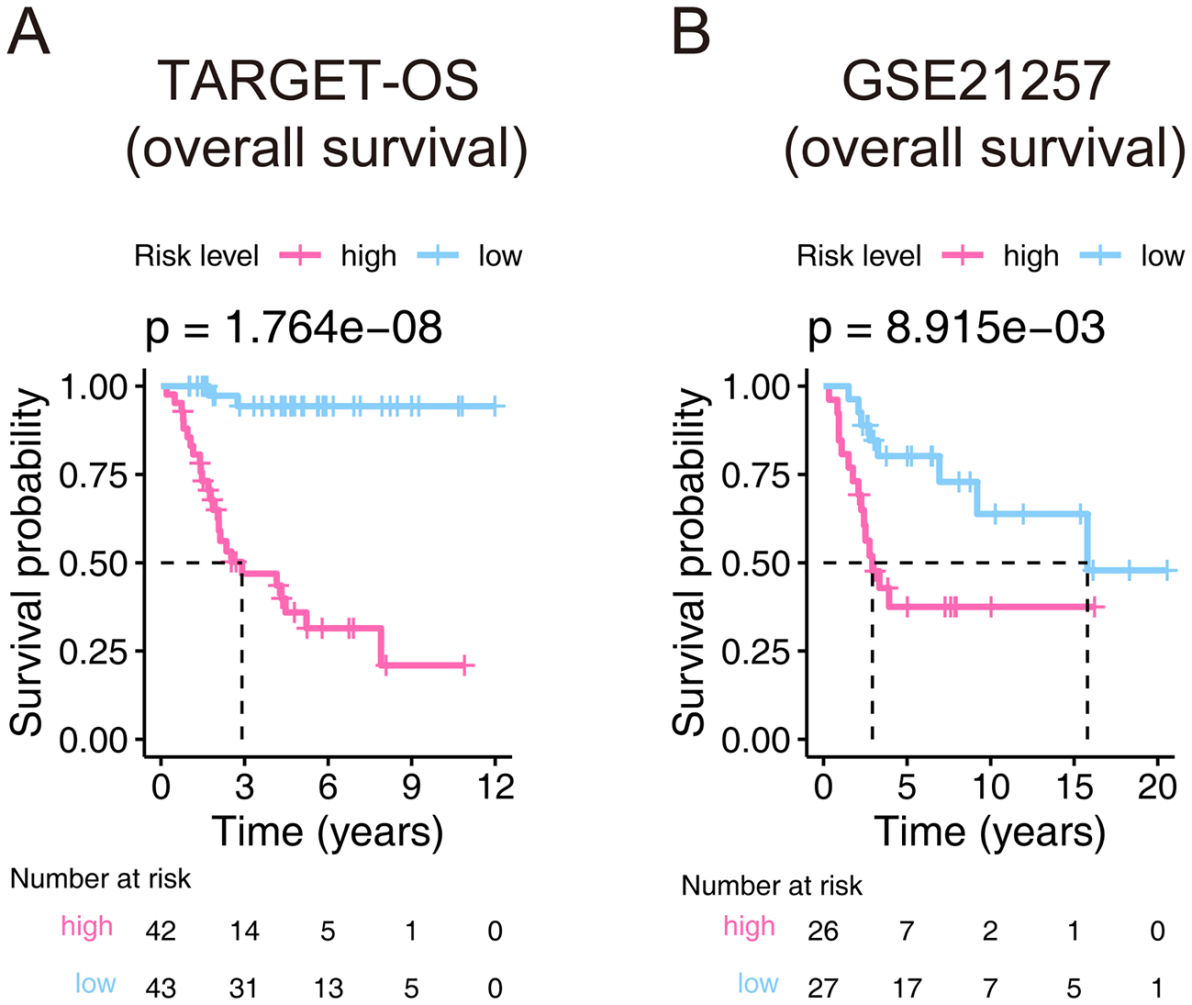
Kaplan–Meier estimator that evaluating the prognosis capacity of the seventeen-gene signature in the training (**A**) and validation (**B**) cohorts. The bottom part indicates the number of patients at risk. The two-sided log-rank test measured the differences between the high- and low-risk groups with a p value < 0.05.

After drawing the risk plot, we first chose the Kaplan–Meier estimator to estimate the ability of the model we built. As shown in Fig. 4, the survival probability of the high-risk group in the training cohort is lower than that of the low-risk group (p value = 1.764E−08), the same is happening in the validation cohort (p value = 8.915E−03), which demonstrated significant survival differences occurred in the signature distinguished patients.

**Figure 5:**
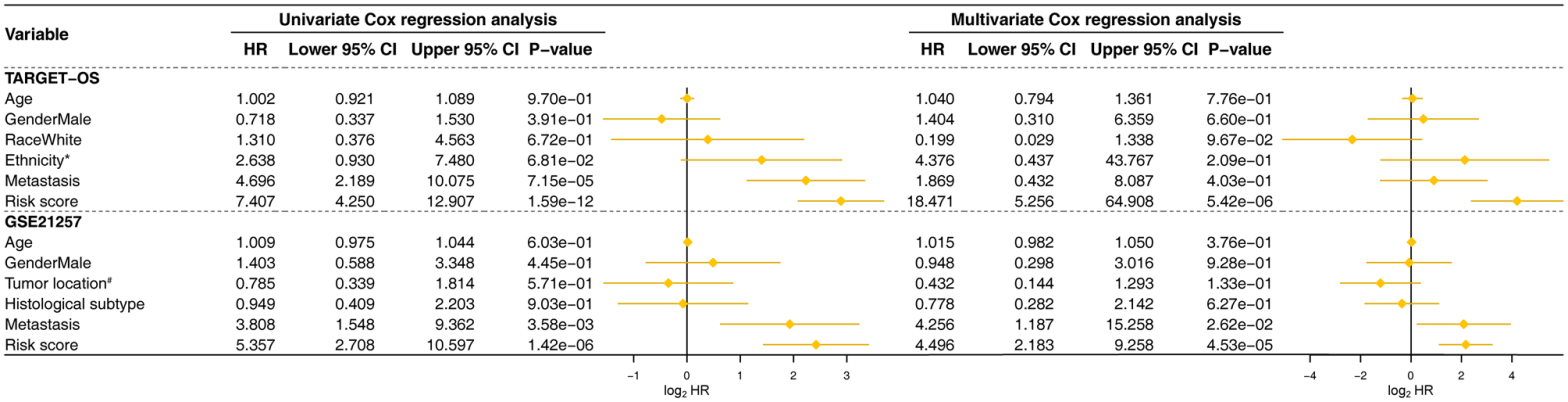
Univariate and multivariate Cox proportional-hazards models that built for testing the predicting ability of the seventeen-gene signature in two cohorts. *HR* hazard ratio, *CI* confidence interval.

The univariate and multivariable Cox regression were established to exam the signature’s prognostic capacity (Fig. 5). Analysis in the training cohort showed that the risk score affected the OS patients’ outcomes (p value ≤ 5.42E−06). Consistently, the results in the validation cohort proved that risk score was the best one affecting prognosis in either univariate or multivariable examination, furtherly confirmed the powerful predictive capacity of the gene signature (p value ≤ 4.53E−05).

**Figure 6:**
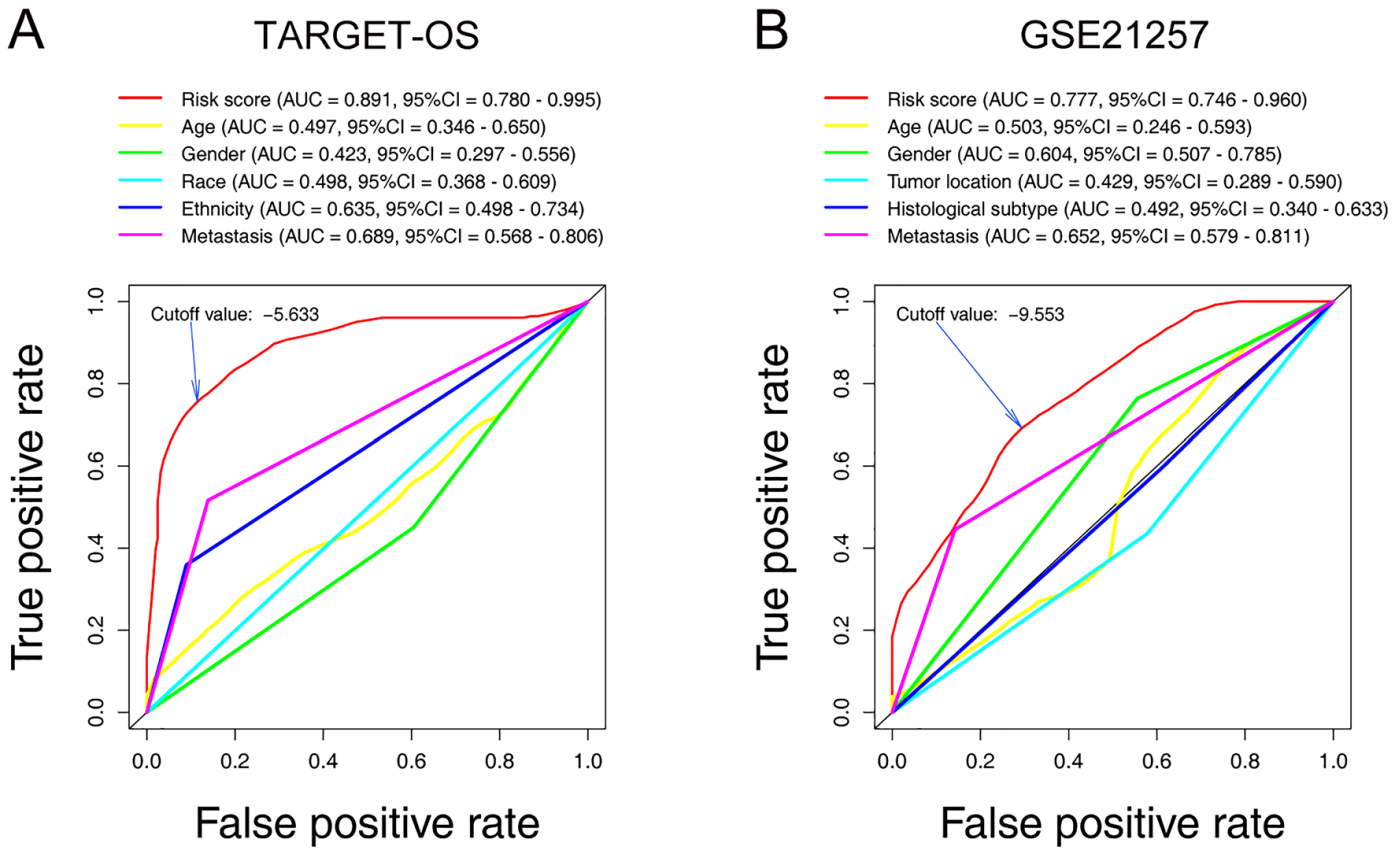
ROC curves that constructed for examining the predictive ability of the seventeen-gene signature in the training (**A**) and validation (**B**) cohorts. *ROC* receiver operating characteristic, *AUC* area under the ROC curve, *95% CI* 95% confidence interval.

As shown in Fig. 6A, ROC analysis indicated that the area under the curve (AUC) for our seventeen-gene signature risk score reached 0.891 (95% CI 0.780–0.995, best cutoff = − 5.633), which was the best among other clinical factors. In the GSE21257 cohort, the AUC as well arrived at 0.777 (95% CI 0.780–0.995, best cutoff = − 9.553), topping other characteristics (Fig. 6B).

### Our gene signature is superior to previous ones

**Figure 7:**
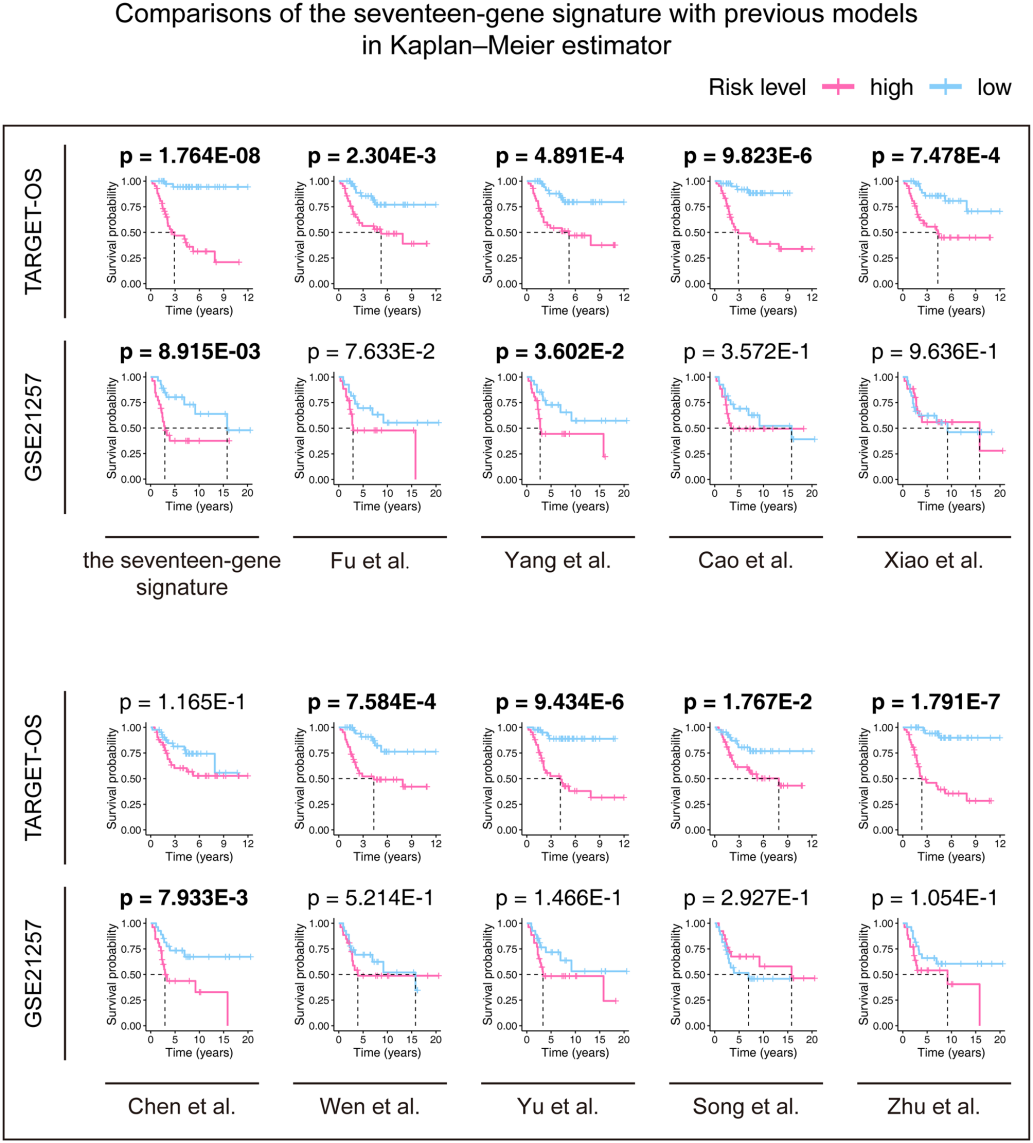
Comparisons between the seventeen-gene signature and previous studies conducted in the training and validation cohorts using Kaplan–Meier estimator. The two-sided log-rank test measured the differences between the high- and low-risk groups. The bold p value indicates that < 0.05, which considers significantly.

Based on the screening criteria set, we found nine studies that suit for our comparison (Table 3). We applied these discovered signatures and their risk score equations to our training and validation cohorts to calculate the risk score of each OS patient. Then, the Kaplan–Meier estimators were built against our signature and previous models (Fig. 7), demonstrating Yang et al.'s and ours have the ability predicting the outcomes of OS. However, our gene signature (p value ≤ 8.915E−03) seemed to be stronger than Yang et al.'s (p value ≤ 3.602E−02) in terms of their p values.

**Table 3:** Candidate research for comparison to our signature.

Authors	Published online date	PMID	Gene signature composition
Fu et al	2021 Mar 18	33816483	*DCN, P4HA1*
Yang et al	2021 May 5	33952718	*P4HA1, ABCB6, STC2*
Cao et al	2020 Dec 23	33425993	*GJA5, APBB1IP, NPC2, FKBP11*
Xiao et al	2020 Dec 15	33384961	*IFITM3, VAMP8, ACTA2, GZMA, CDCA7, EVI2B, SLC7A7*
Chen et al	2020 Dec 14	33381518	*MSR1, TLR7*
Wen et al	2020 Dec 3	33281116	*COCH, MYOM2, PDE1B*
Yu et al	2020 Aug 21	32820615	*CXCR3, SSTR3, SAA1, CCL4, PYY, CCR9, CXCL9, CXCL11, C3, CXCL2, S1PR4, CXCL10, CXCR6*
Song et al	2020 Jul 24	32850346	*CD4, CD68, CSF1R*
Zhu et al	2020 Jun 22	32581649	*SLC18B1, RBMXL1, DOK3, HS3ST2, ATP6V0D1, CCAR1, C1QTNF1*

*PMID* PubMed ID.

**Figure 8:**
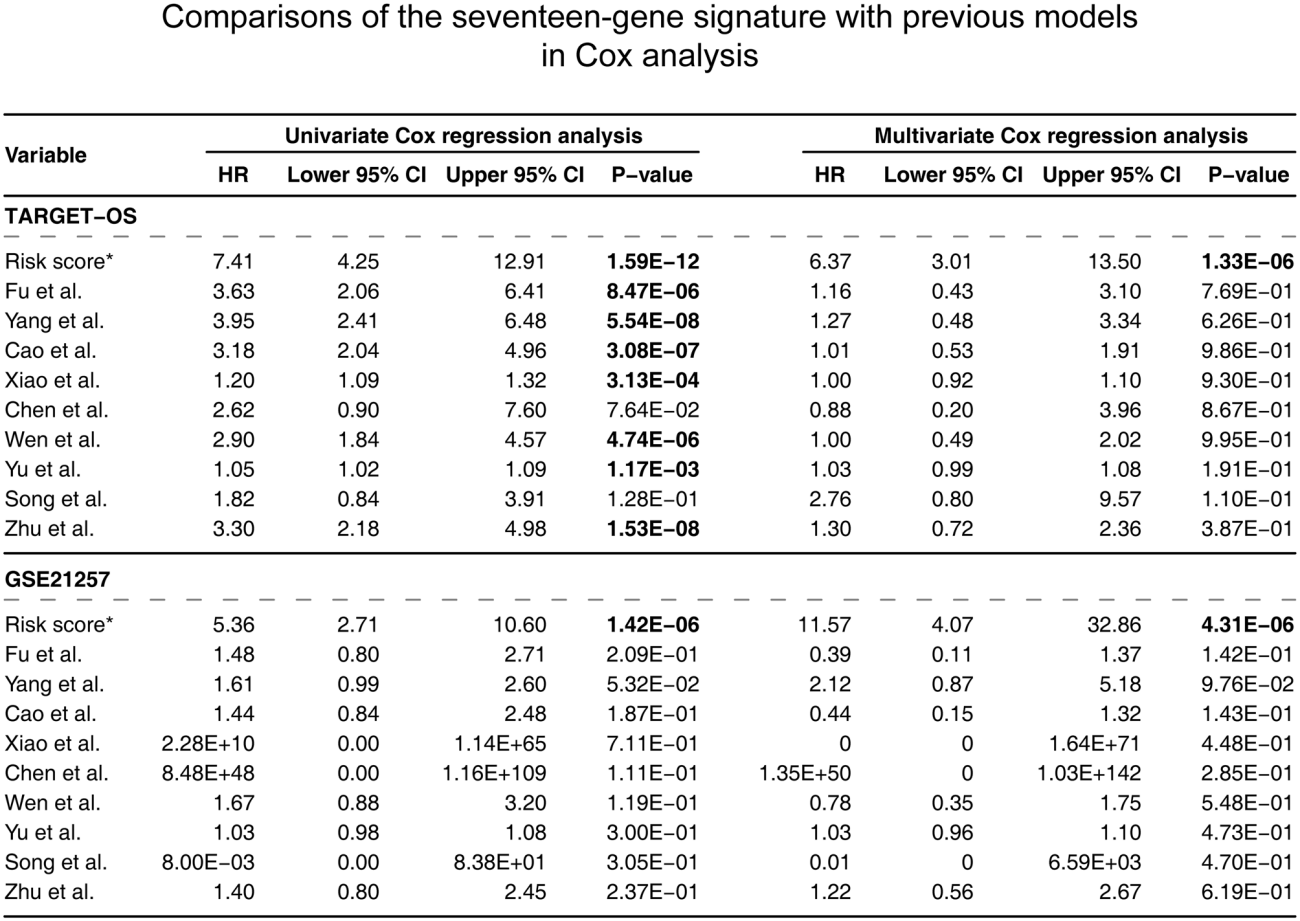
Comparisons between the seventeen-gene signature and previous studies conducted in the training and validation cohorts using Cox models. *HR* hazard ratio, *CI* confidence interval. *The seventeen-gene signature that identified in this study; the bold p value indicates that < 0.05, which considers significantly.

Additionally, Cox univariate and multivariable regression were constructed using these selected prognosis models (Fig. 8). The results in the training cohort demonstrated that only our gene signature (p value ≤ 1.33E−06) having the prognosis capabilities in both the univariate and multivariable analyses. The Cox analysis of the validation cohort determined that only our gene signature passed all the univariate and multivariable tests (p value ≤ 4.31E−06).

### GO and KEGG enrichment analysis with the seventeen-gene signature

According to the risk score for each case in the TARGET-OS cohort, we conducted GO and KEGG enrichment analysis between high-risk and low-risk groups. The GO enrichment result showed the differences between the two groups mainly focus on extracellular matrix organization, extracellular structure organization, collagen—containing extracellular matrix, endoplasmic reticulum lumen, and extracellular matrix structural constituent (Figure S2A). KEGG analysis was showed that the enriched items were mainly related to protein digestion and absorption, complement and coagulation cascades, and Wnt signaling pathway (Figure S2B).

### Relationships between the seventeen-gene signature and immune relevant signatures

**Figure 9:**
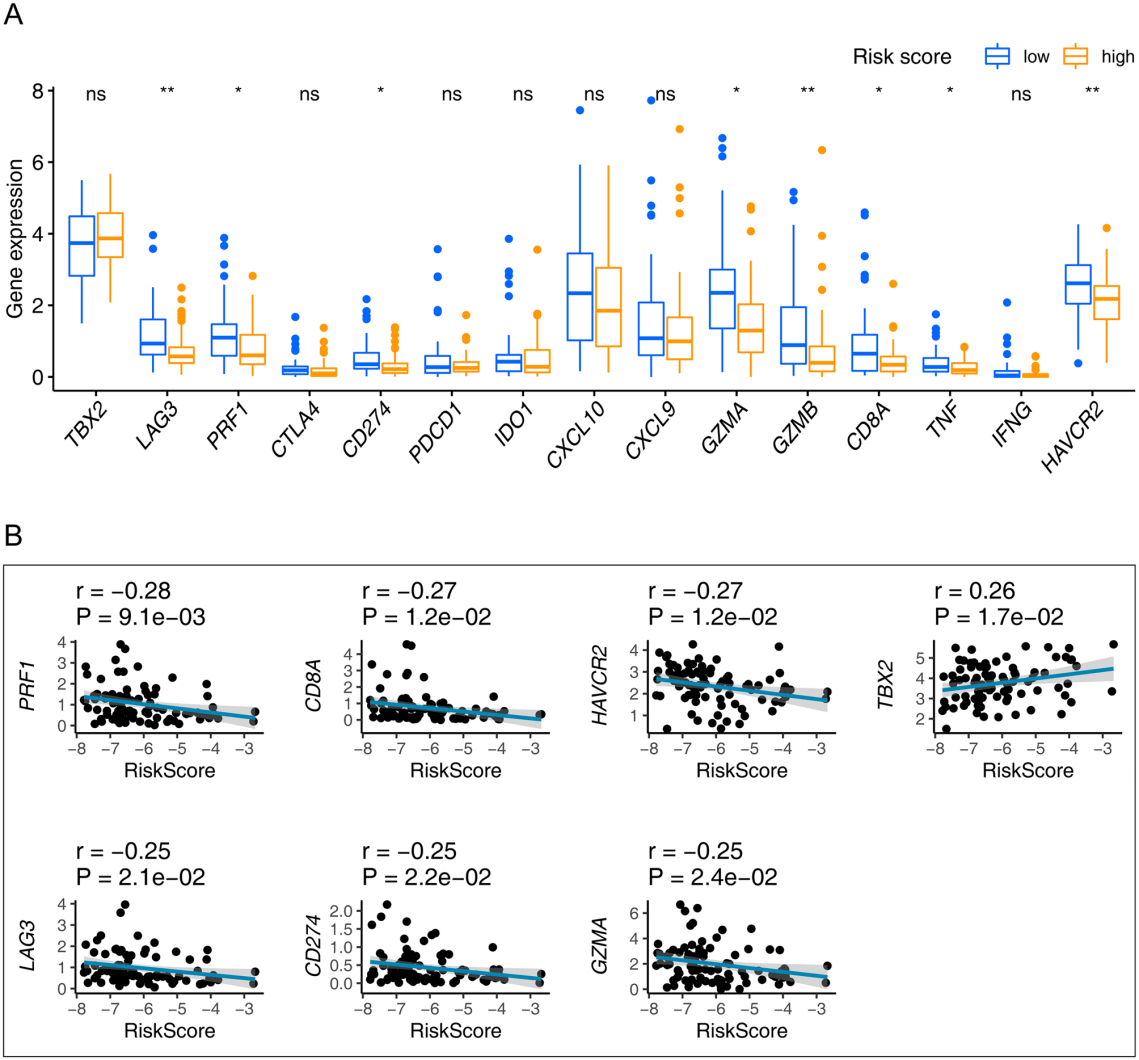
Identification of the relationships between the seventeen-gene signature and immune relevant signatures. (**A**) Wilcoxon rank-sum was adopted to differentiate immune relevant signatures between the high- and low-risk groups. (**B**) The Pearson coefficient was applied for the correlation test between the immune relevant signatures and seventeen-gene signature. Only correlations with p value < 0.05 were plotted. ns: p value > 0.05; *p value < 0.05; **p value < 0.01; ***p value < 0.001; p value < 0.05 was considered statistically significant.

We observed that 8/15 of the immune relevant signatures in the high-risk group were significantly under expressed, as demonstrated by the Wilcoxon test (Fig. 9A). The Pearson coefficient test discovered 7/15 of the immune relevant signatures correlated with the seventeen-gene signature (Fig. 9B, Table S3). Incorporating the above findings, six genes, including *PRF1, CD8A, HAVCR2, LAG3, CD274,* and *GZMA* were identified associating with the seventeen-gene signature.

### The seventeen-gene signature and 22 TICs

The GO and KEGG analysis suggested that the difference between the two groups was related to the immune response, so we further conducted 22 TICs analysis to better study how the seventeen-gene signature interact with the immune microenvironment. CIBERSORT algorithm was used to determine the proportion of the tumor-infiltrating immune subpopulations. We visual outputted the 22 TICs distribution and inner correlation in Figure S3.

**Figure 10:**
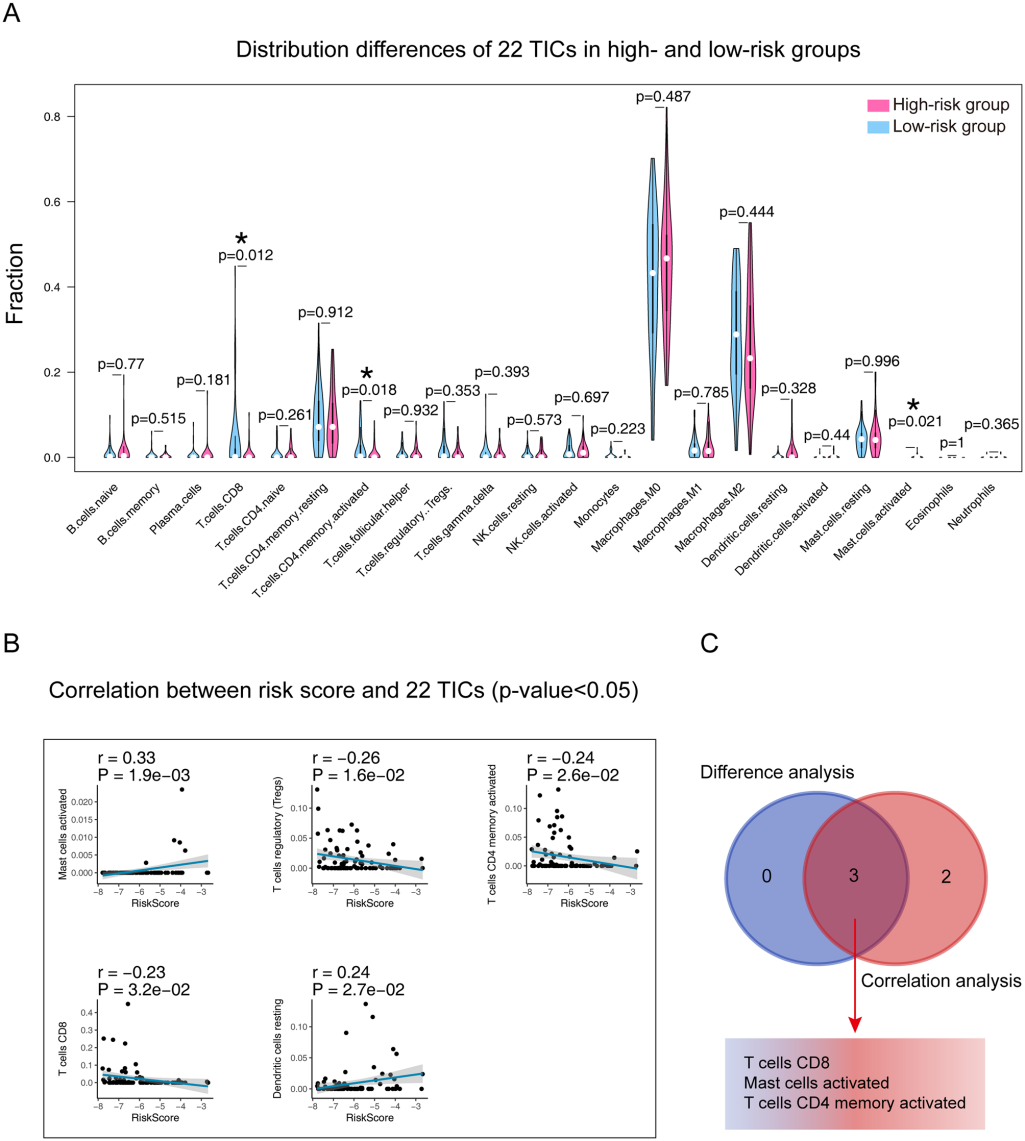
Integrating analysis for the relationship between TICs and the seventeen-gene signature. (**A**) Wilcoxon rank-sum was adopted to differentiate each of 22 TICs between the high- and low-risk groups. (**B**) The Pearson coefficient was applied for the correlation test between the TICs and the seventeen-gene signature. Only correlations with p value < 0.05 were plotted. (**C**) The Venn diagram that integrating the results from (**A**) and (**B**). TIC: tumor-infiltrating immune cell; *p value < 0.05; p value < 0.05 was considered statistically significant.

Combining the findings from difference analysis (Fig. 10A) and correlation analysis (Fig. 10B, Table S4), three TICs (Fig. 10C), including T cells CD8, Mast cells activated, and T cells CD4 memory activated were identified associating with the seventeen-gene signature. Among them, Mast cells activated were found positively correlated with the gene signature, while the others negatively.

**Figure 11:**
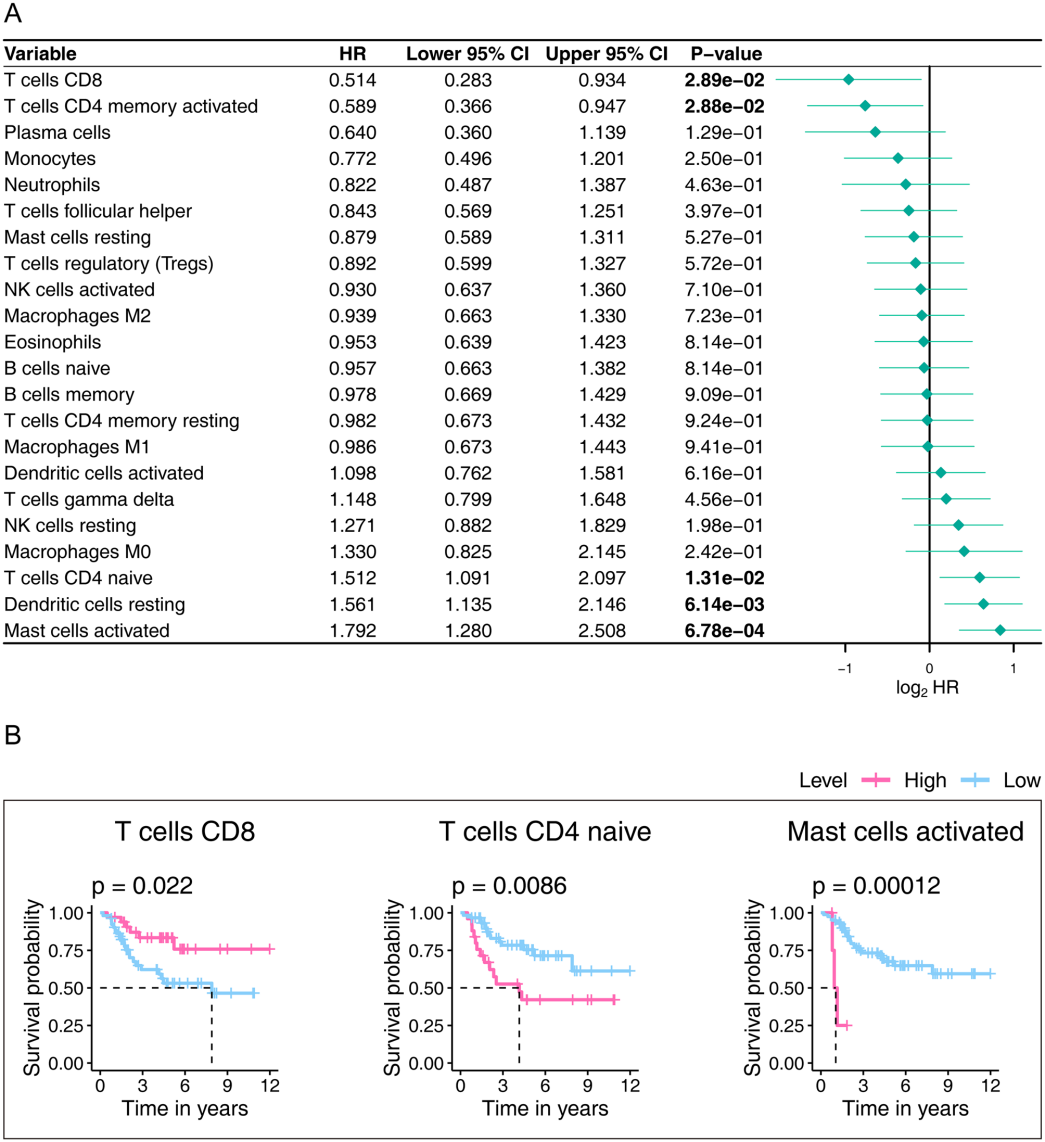
Univariate Cox proportional-hazards model (**A**) and Kaplan–Meier estimator (**B**) that built for evaluating the prognostic ability of 22 TICs. Only graphs with a p value < 0.05 in the log-rank test were plotted in (**B**). The bold p value indicates that < 0.05, which considers significant. *TIC* tumor-infiltrating immune cell.

We further tested the 22 TICs prognostic abilities by consulting the Kaplan–Meier estimator and univariate Cox proportional-hazard model. As displayed in Fig. 11, the univariate Cox proportional-hazard model (Fig. 11A) indicated that T cells CD8, T cells CD4 memory activated, T cells CD4 naïve, Dendritic cells resting, and Mast cells activated impacted prognosis. Additionally, Kaplan–Meier estimator (Fig. 11B; Table S5) highlighted that T cells CD8, T cells CD4 naïve, and Mast cells activated can predict the survival rate of OS. From the above survival analysis, it can be determined that T cells CD8, T cells CD4 naïve, and Mast cells activated have potential prognostic ability in OS.

The results of this part found that T cells CD8 and Mast cells activated were significantly related to our signature and closely related to the OS prognosis, potentially implying that T cells CD8 and Mast cells activated infiltrations play vital roles in the discovered signature in OS patients.

## Discussion

In this study, we innovatively discovered a robust seventeen-gene prognostic signature for the OS prognosis by mining TARGET and GEO databases. Specifically, our novelty lay in using univariate Cox model, Kaplan–Meier estimator, and LASSO regression in the model training phase. The adoption of an independent cohort, Kaplan–Meier analysis, Cox regression, ROC curve in the validation process, moreover, highlighted our innovativeness. Most importantly, we compared our signature with published research to prove ours' superiority. At the end of the study, we discovered important mechanisms related to gene signature through function annotations, immune gene correlation analysis, and immune infiltration analysis and speculated that the T cells CD8 and Mast cells activated might potentially help the predictive ability of the signature. This study we worked on designed to shed light on the development of future OS research.

Our signature consists of seventeen genes (Table 2), which were *UBE2L3, PLD3, SLC45A4, CLTC, CTNNBIP1, FBXL5, MKL2, SELPLG, C3orf14, WDR53, ZFP90, UHRF2, ARX, CORT, DDX26B, MYC,* and *SLC16A3*, respectively. After tested in the two cohorts (Figure S1), *UBE2L3, PLD3, SLC45A4, CTNNBIP1, FBXL5, SELPLG, WDR53,* and *ZFP90*, showed solid protective impacts on OS, while *UHRF2, ARX, CORT, DDX26B, MYC,* and *SLC16A3* witnessed effects on OS prognosis unfavorably. Our findings suggest that *CTNNBIP1* is a suppressor of cancer migration, thus making it a potential prognostic predictor for OS. Rothzerg et al. also demonstrated that high expression of CTNNB1 was associated with a good OS prognosis, which is consistent with our findings^23^. In the *UHRF* family, *UHRF1* and UHRF2 have a multidomain architecture and have similarities in sequence and domain organization^24^. *UHRF1* is a well-known epigenetic regulator. Significant *UHRF1* overexpression has been shown in many kinds of tumors^25^. Liu et al. reported that *UHRF1* promotes the proliferation of human OS cells and increases the invasiveness of human OS cells by down-regulating the E-cadherin expression and increasing EMT in an Rb1-dependent manner^25^. *CORT* is an endogenous cyclic neuropeptide, which can regulate the growth and metastasis of lung cancer and thyroid cancer, and regulate inflammation by inhibiting immune infiltration^26^. Wu et al. found that the expression of *CORT* was higher in high-risk OS populations, confirmed that the high expression of *CORT* was related to the poor prognosis of OS^27^. According to previous studies, *MYC* is widely involved in many cancers, and its expression is estimated to be elevated or dysregulated in up to 70% of human cancers^28^. *MYC* mediated transcriptional amplification through super enhancers is an important hallmark of cancer^29^. The dysregulated expression of the oncogene *MYC* is usually associated with the oncogenesis and progression of OS^30^. *MYC* proto-oncogene boosts the oncogenic transcription amplification process in cancer and is a crucial target for cancer therapy^30^. It is reported that the *MYC* gene is amplified in OS, and its expression is often up regulated in patients with OS^30^. *MYC* overexpression, coupled with the loss of Ink4a/Arf, can further the transformation of bone marrow stromal cells into OS^30^. Above all, high *MYC* levels are related to low apoptosis and poor outcomes in patients with OS^30^. Chen's team recently demonstrated that *MYC*-driven super-enhancer signaling is essential for OS tumorigenesis, and the *MYC*/super-enhancer axis targeting therapeutic strategy to be a promising perspective for OS patients^30^. The genes *ARX*^31,32^, *DDX26B*^33,34^, and *SLC16A3*^35,36^ have been reported to be involved in the occurrence and development of certain cancers, but whether they play an important role in OS has not yet been revealed, implied more efforts are needed.

Freshly, with the widespread application of bioinformatics, potential gene signatures associated with OS prognosis were generated from the publicly databases, which witnessed by more and more involved research. To judge the pros or cons of our signature, we found nine studies published in the most recent past year and compared them horizontally^37–45^. The comparison results once again confirmed our discovery is superior in predicting the prognosis of OS.

KEGG analysis was showed that the enriched items were mainly related to protein digestion and absorption, complement and coagulation cascades, and Wnt signaling pathway (Figure S3B). Wnt signaling is one of the key cascades regulating development and stemness, and it is also closely related to cancer^46^. The role of Wnt signaling in carcinogenesis has been most prominently described in colorectal cancer, but abnormal Wnt signaling has been observed in more cancer entities^46^. Constitutive Wnt signal activation is common in human OS, while gene mutations that activate components of the Wnt pathway are rare in OS^1^. Wnt signaling may play a key role in OS proliferation, metastasis and OS cancer stem cell maintenance^1^.

Immunotherapy is a type of therapy that helps the individual's immune system eliminate or control cancer^47^. Recently, immunotherapy has begun to show good prospects in various adult cancers, but whether this method is effective in OS is still rarely reported. Several biological characteristics of OS suggest that the regulation of the immune response may bring benefits, and the various immune approaches available now make immunotherapy potential for OS^48^. One of the main challenges of immunotherapy is identifying biomarkers that predict response so that treatments can be tailored to maximize benefits^48^. In the present study, six genes, including *PRF1, CD8A, HAVCR2, LAG3, CD274* (*PD-L1*), and *GZMA*, were identified as closely related to our seventeen-gene signature and might guide future OS immunotherapy.

Combining the findings of immune infiltration analysis and the 22 TICs survival analysis, we speculated that the extensive infiltration of T cells CD8 and Mast cells activated in tumors may help our signature to achieve stable predictive ability. There is evidence that *PD-1* is involved in the progression of OS disease, and the percentage of *PD-1* in peripheral blood CD4 + and CD8 + T lymphocytes in OS patients is significantly up-regulated^49^. More importantly, in vivo and in vitro experiments conducted by researchers have confirmed that *PD-L1* in OS is significantly expressed^49^. Therefore, inhibition of *PD-1/PD-L1* is an interesting therapeutic target that can restore the function of the immune system to OS cells^49^. Mast cells are immune cells that accumulate in tumors and their microenvironment during disease progression^50^. They play a multi-faceted role in the tumor microenvironment by regulating various events in tumor biology, such as angiogenesis, cell proliferation, and survival^50^. Invasion and transfer. Mast cells are recruited in the early stages of tumor development and play a key role in angiogenesis and tissue remodeling and promote tumor occurrence and growth^50^. As tumor growth progresses, mast cells recruit immune cells or suppress anti-tumor responses^50^. We know from previous studies that mast cells affect the homeostasis of OS and affect tumor progression, but we have not yet understood its underlying mechanism^50–52^. Interestingly, our research showed that T cells CD8 and Mast cells activated can potentially target the gene signature in OS treatment. Thus, further research should consider closely to the roles that the T cells CD8 and Mast cells activated play in the remodeling of the tumor microenvironment.

In the end, we must clarify the limitations of this research. The seventeen-gene signature we derived was from retrospective data. We believe that more prospective data can make our results more effective and rigorous. In addition, although it has absolute superiority compared with previous studies, its proof results are derived from the analysis results of three public databases. There is still no wet laboratory data to explain and support the prognostic ability of these 17 genes and their role in immune infiltration. Therefore, ongoing research is needed to reveal more evidence to for the seventeen -gene signature’s promising future.

## Conclusion

The present work identified a novel and robust seventeen-gene signature for the OS prognosis by mining TARGET and GEO databases. In addition, we determined the reliability and applicability of the signature by applying it to an independent cohort. Through comparison, we confirm that our signature is superior to previous research. We identified our signature’s potential immunotherapy targets and the important role of T cells CD8 and Mast cells activated in the seventeen-gene signature prognostic capacity. The real-world influence of the seventeen-gene signature and the underlying mechanisms between it and tumor immunity in OS remained a lack of research and warranted further investigation.

## Supplementary Information

Supplementary Information.

## Data Availability

Publicly available datasets were used in this study. Data from TARGET-OS (https://ocg.cancer.gov/programs/target/projects/osteosarcoma) and data from GSE21257 (https://www.ncbi.nlm.nih.gov/geo/query/acc.cgi?acc=GSE21257) were downloaded and analyzed in this work.
