# Supplementary material for: Construction and validation of a novel gene signature for predicting the prognosis of osteosarcoma
Source: Sci Rep. 2022 Jan 24;12:1279. doi: 10.1038/s41598-022-05341-5 (PMC8786962; doi:10.1038/s41598-022-05341-5)
Supplement: Supplementary file 1 — Supplementary Information. [file 41598_2022_5341_MOESM1_ESM.pdf]

Figure S1

A

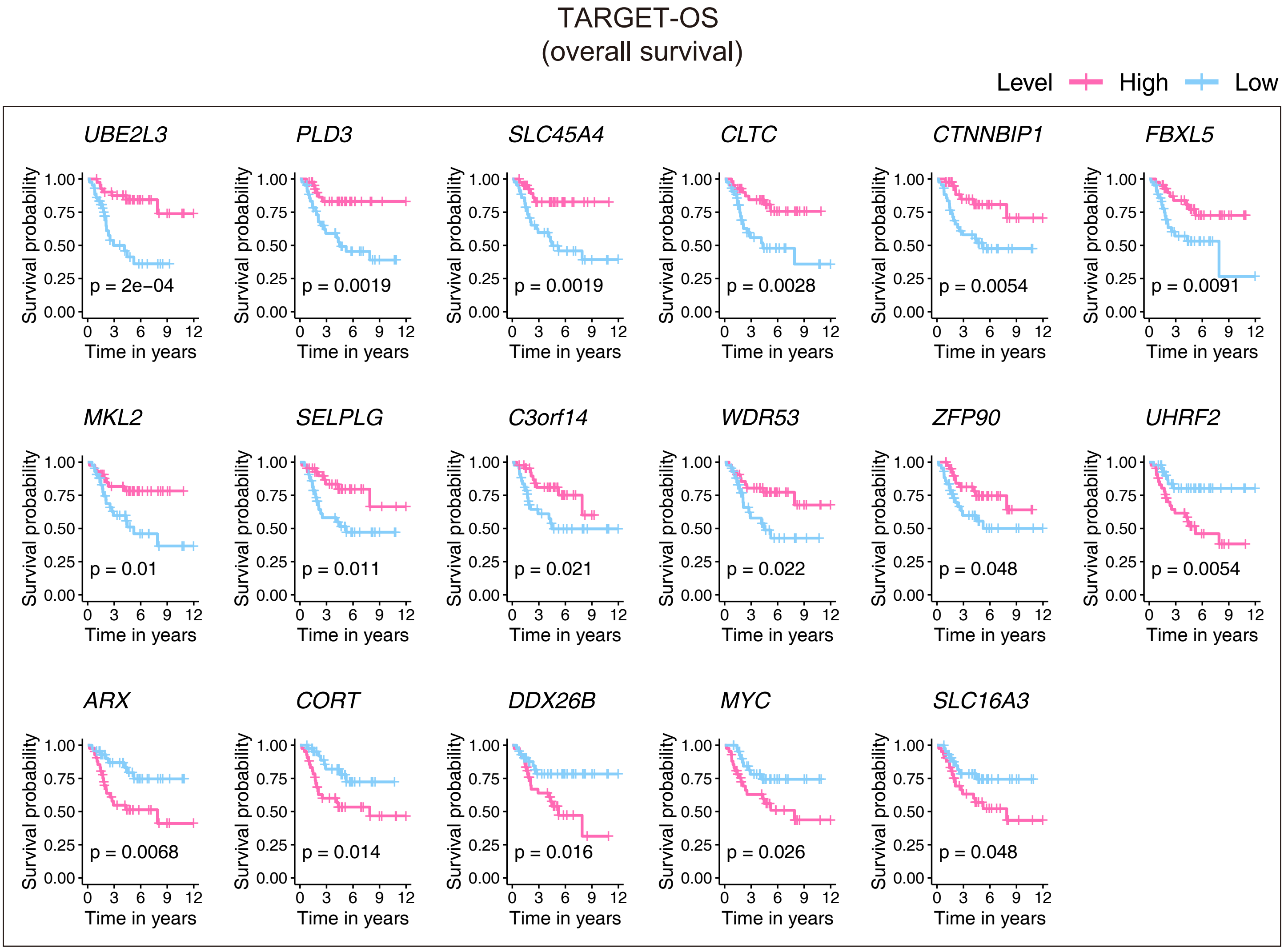

B

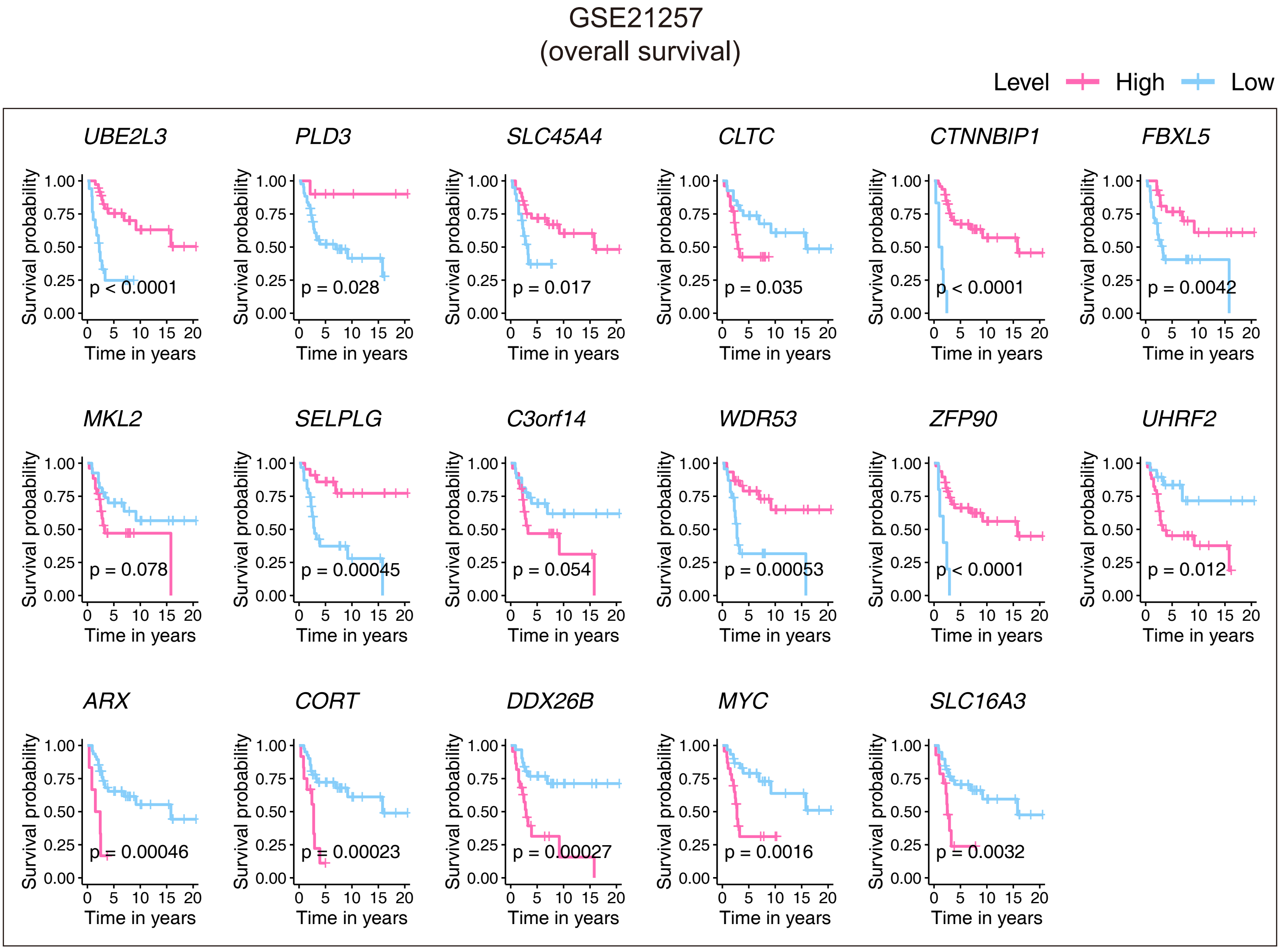

Figure S1. The prognostic prediction ability of each gene in the seventeen-gene signature tested using Kaplan–Meier estimator in the training (A) and validation (B) cohorts. The two-sided log-rank test measured the differences between the high and low expression level group. P-value < 0.05 was considered as significantly.

Figure S2

A

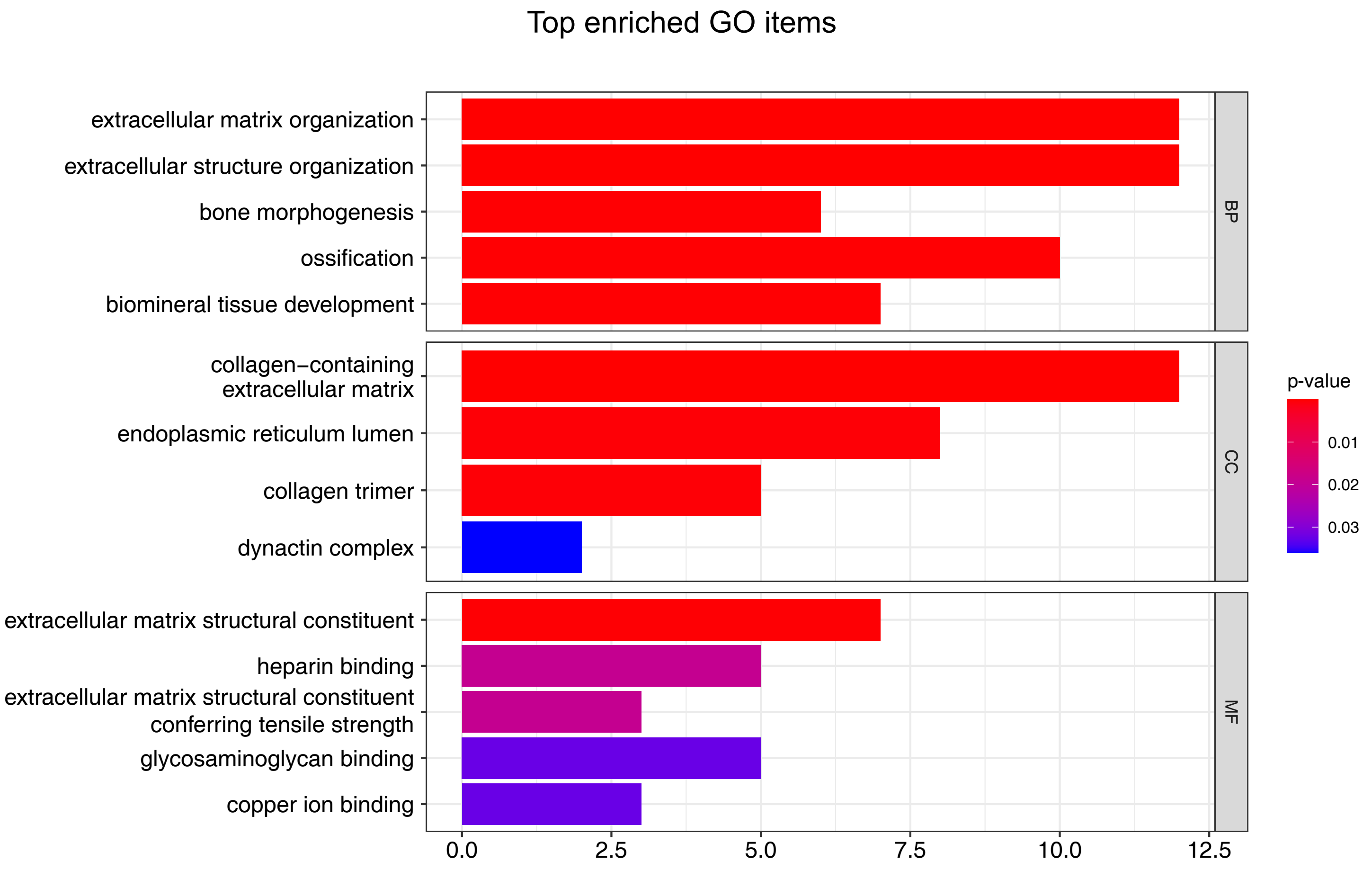

B

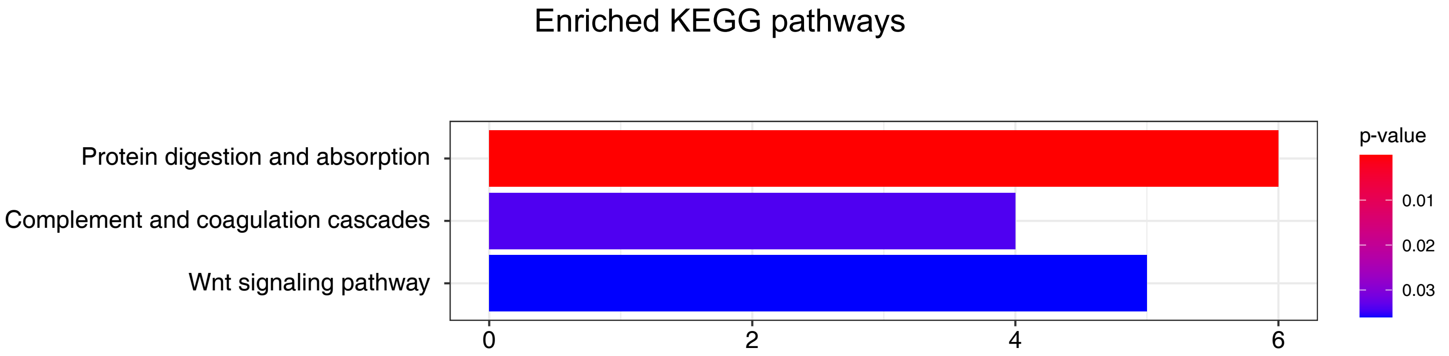

Figure S2. Functional annotation including GO (A) and KEGG (B) of the seventeen-gene signature in the training cohort. GO: Gene Ontology; BP: Biological Process; CC: Cellular Component; MF: Molecular Function; KEGG: Kyoto Encyclopedia of Genes and Genomes.

Figure S3

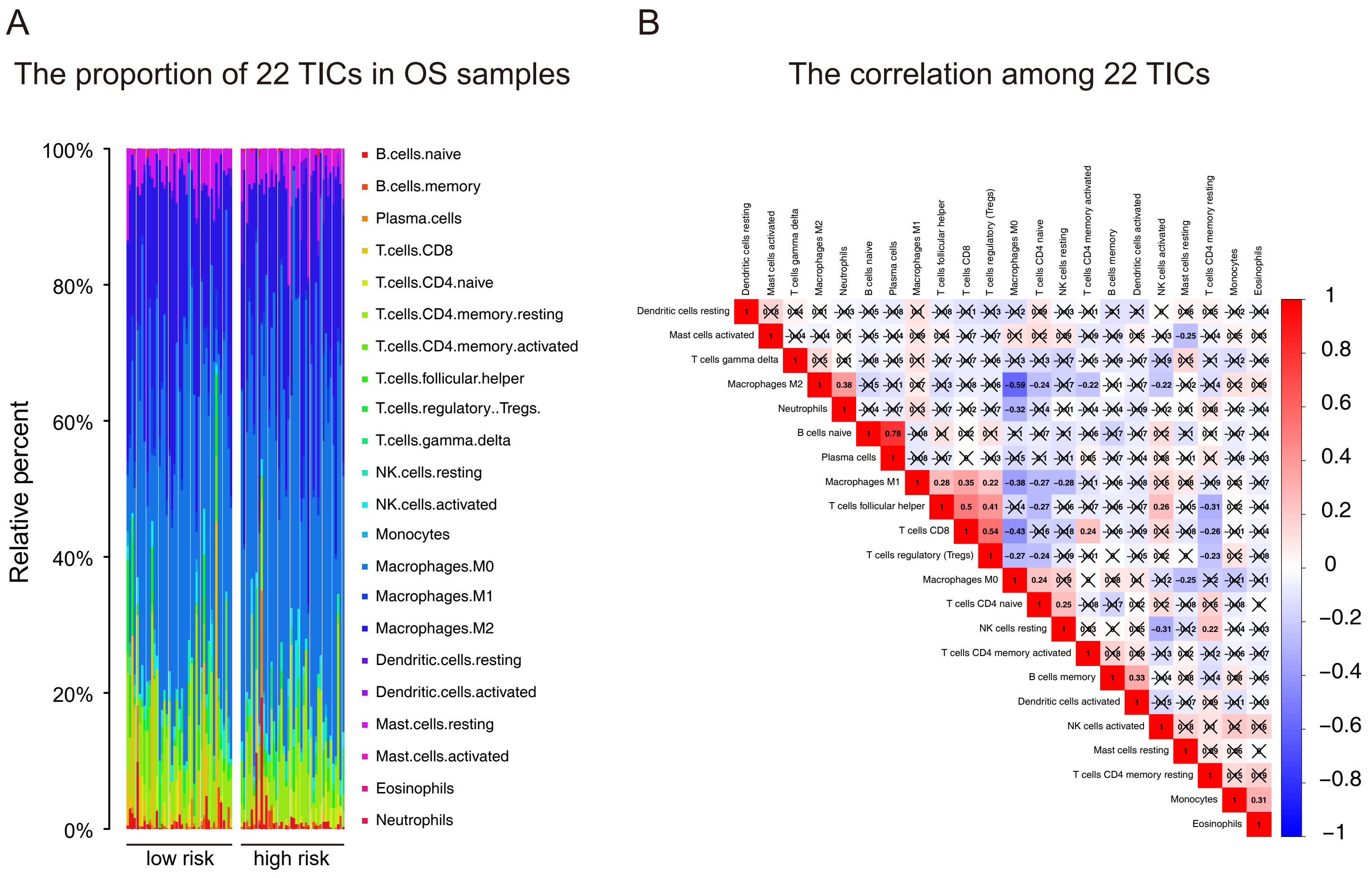

Figure S3. Profiles and correlation analysis of the 22 TICs in OS tumor samples in the training cohort. (**A**) The distribution of 22 TICs in high- and low-risk OSs in the training cohort, which displayed in the form of bar plots. (**B**) The inner correlations between 22 TICs, which shown in the form of heatmap. TIC: tumor-infiltrating immune cell; OS: osteosarcoma; P-value < 0.05 was considered statistically significant.

Table S1. Kaplan–Meier estimator and univariate Cox proportional-hazards model built for each gene in the training cohort.

ID	KM_pvalue	HR	HR_95L	HR_95H	Cox_pvalue
A1BG	0.728522123	0.237319819	0.037696015	1.494075591	0.125461987
A2M	0.91475861	2.224413481	0.575334385	8.600242682	0.246556984
A2ML1	0.539893443	0.808376575	5.37E-06	121614.7543	0.972100419
A3GALT2	0.055009185	2.83399614	0.620394661	12.94584661	0.178944706
A4GALT	0.665673105	0.989738163	0.027221907	35.98504782	0.995511088
A4GNT	0.136477673	0.625604792	0.288970532	1.354398849	0.233972365
AAAS	0.943523192	1.03225079	0.598896475	1.779175097	0.909019437
AACS	0.584305905	0.088971643	0.000641093	12.34759432	0.336399832
AADAC	0.927902603	14.5685822	0.825504344	257.1077775	0.067394144
AADACL2	0.264846106	36.45105159	0.715136359	1857.938203	0.07300397
AADAT	0.048440578	1.541487933	1.053239832	2.256072146	0.025954766
AAK1	0.59358941	0.635503544	0.319816958	1.262799689	0.19567754
AAMP	0.524190924	0.857748306	0.050130606	14.67630679	0.915655036
AANAT	0.215168496	0.467196568	0.1518764	1.437172808	0.184386436
AARS	0.925890791	1.256453097	0.688201398	2.293913364	0.457296186
AARSD1	0.284412732	1.126084991	0.512234156	2.475561988	0.767642524
AASDH	0.895622947	3.652446219	0.433936973	30.742629	0.233321988
AASDHPPT	0.761641006	0.178390604	0.009428737	3.375129473	0.250522188
AASS	0.756032738	2861.406224	0.364873917	22439657.07	0.08192886
AATF	0.730883092	0.204550719	0.008319321	5.029376483	0.33139693
AATK	0.181601748	2.002382666	0.239499167	16.7413373	0.521618777
ABAT	0.465386089	0.763051557	0.407788057	1.427819354	0.397599564
ABCA1	0.107843582	3.132083745	0.700563459	14.00294073	0.135119757
ABCA10	0.951534267	0.006131266	1.42E-07	264.0361122	0.349407408
ABCA12	0.321632144	0.692260926	0.277050383	1.729740217	0.431183558
ABCA13	0.525340999	1.096581827	0.848555218	1.417104837	0.480980446
ABCA2	0.673317518	5.987584581	0.282405673	126.9491817	0.250749818
ABCA3	0.226230025	1.925568777	0.591156599	6.272136892	0.276821045
ABCA4	0.804053175	0.3758823	0.020235392	6.982197571	0.511591815
ABCA5	0.219873275	1.376868982	0.415263739	4.565214867	0.601018175
ABCA6	0.979142635	0.915092348	0.359786658	2.327473764	0.852214074

ABCA7	0.41306816	1.126092835	0.830939069	1.526086713	0.443821806
ABCA8	0.863211552	0.921641472	0.455596884	1.864417937	0.820425166
ABCA9	0.210077293	0.773680124	0.545438041	1.097431586	0.150239531
ABCB1	0.378929326	0.768783262	0.355105065	1.664374186	0.504625481
ABCB10	0.157913385	1.28191958	0.667558275	2.461684426	0.455650287
ABCB11	0.91920299	1.646649903	0.835030244	3.247134968	0.149986363
ABCB4	0.124034967	0.226004536	0.043290142	1.179900265	0.07777077
ABCB5	0.620983124	0.825124141	0.321859148	2.115303706	0.689016362
ABCB6	0.477894418	0.636796986	0.180793276	2.242950681	0.482355057
ABCB7	0.153940809	0.902514825	0.045715436	17.8174612	0.946264194
ABCB8	0.243551058	0.980940052	0.102161944	9.418804618	0.986696118
ABCB9	0.485709012	1.236724936	0.892833431	1.713072689	0.201220599
ABCC1	0.862939102	5.787535354	0.000115363	290349.8122	0.750529472
ABCC10	0.480961618	162.8238149	0.003969194	6679340.411	0.347367386
ABCC11	0.059042292	1.307222296	0.642493775	2.65968356	0.459762095
ABCC12	0.327724211	0.35400809	0.001073701	116.7193578	0.725572145
ABCC13	0.41068005	0.402692546	0.013694216	11.84158942	0.598017892
ABCC2	0.803489336	0.409299494	0.001675478	99.98703167	0.750157045
ABCC3	0.532221932	0.769937069	0.23490077	2.523631957	0.665999295
ABCC4	0.882025663	0.883777867	0.002932709	266.3282465	0.966162848
ABCC5	0.984489883	2.083984689	0.512054214	8.481508534	0.305205511
ABCC6	0.29640846	2.598977957	0.020197371	334.433941	0.699943238
ABCC8	0.290617895	0.058152377	0.000269639	12.54157884	0.299482619
ABCC9	0.930338656	0.663342927	0.23353583	1.884181282	0.440932633
ABCD1	0.734323151	2.701754509	0.527770839	13.83077065	0.232905713
ABCD2	0.886722129	1.097529214	0.55004268	2.189958013	0.791757117
ABCD3	0.719757586	0.34977937	8.86E-05	1380.980355	0.80365223
ABCD4	0.16051052	293.6201524	0.106766849	807486.5452	0.159633826
ABCE1	0.957285759	0.153878834	4.24E-05	558.316457	0.654486982
ABCF1	0.397886124	0.966408189	0.5411935	1.725713238	0.908046114
ABCF2	0.863312417	0.994763183	0.49755128	1.988847844	0.988148657
ABCF3	0.543277201	1.138737924	0.444237537	2.918988049	0.786764557
ABCG1	0.055305982	1.418052545	0.478127807	4.205722799	0.528891308

ABCG2	0.612555482	0.992603695	0.559502187	1.760962008	0.979751281
ABCG4	0.468782327	2.163587607	0.243530983	19.22183074	0.488616512
ABCG5	0.255116944	238.0479736	0.114612737	494420.0713	0.160273719
ABCG8	0.274469717	0.746120034	0.364628559	1.526745757	0.422735224
ABHD1	0.34302944	0.772269484	0.332124616	1.795711989	0.548345703
ABHD10	0.335239172	25.28936957	0.012988492	49239.91222	0.403191351
ABHD11	0.472003896	0.725028603	0.269711658	1.94899427	0.523918904
ABHD14A	0.627331408	1.638394087	0.136444655	19.67343594	0.697042046
ABHD14B	0.164273255	0.011151303	4.80E-05	2.588888106	0.105723916
ABHD2	0.068866927	2.375534277	0.67085322	8.41191923	0.179867127
ABHD3	0.308238244	165.2102942	0.007684678	3551800.097	0.315653295
ABHD4	0.580858247	0.79102291	0.431254163	1.450924534	0.448800305
ABHD5	0.427247544	0.936801188	0.237858725	3.689570209	0.925630703
ABHD6	0.48035553	5.948447648	0.031222618	1133.281957	0.505588582
ABHD8	0.120548631	0.701907491	0.195705753	2.517422805	0.587009798
ABI1	0.896514743	0.229754926	0.002957242	17.85018813	0.50781267
ABI2	0.480751379	1.647921943	0.738599784	3.676749964	0.222481742
ABI3	0.966984781	1.347113862	0.009848657	184.2602329	0.905482868
ABI3BP	0.327175757	1.045755182	0.840816267	1.300645508	0.687675293
ABL1	0.17374033	0.890468708	0.317239122	2.499485294	0.825637322
ABL2	0.259370679	2.399443916	0.960471809	5.994273913	0.060981743
ABLIM1	0.572548472	1.130228338	0.485297569	2.632232629	0.776554554
ABLIM2	0.288778625	0.240493556	0.01544317	3.745160424	0.309002748
ABLIM3	0.340366305	6.209753417	0.280376809	137.5329065	0.247926579
ABO	0.573335603	29.47354478	0.004071042	213382.6684	0.455561254
ABR	0.146755433	0.747656345	0.386846166	1.44499302	0.387023708
ABRA	0.973424636	1.10291918	0.699044547	1.740133332	0.673719138
ABT1	0.60963915	0.64835081	0.229749704	1.829637933	0.412987933
ABTB1	0.622806482	0.343382468	5.63E-07	209490.4057	0.875033355
ABTB2	0.861168898	0.014770245	6.60E-06	33.06665837	0.284158505
ACAA1	0.101424974	0.786738638	0.398644265	1.55265669	0.489237297
ACAA2	0.836045921	0.069270773	0.000180575	26.57313811	0.379141466
ACACA	0.09864004	1.64374616	0.654412086	4.128746238	0.290231168

ACACB	0.865550983	1.194499454	0.514268174	2.774484242	0.679355968
ACAD10	0.368519735	0.672182284	0.35820184	1.26138108	0.216122732
ACAD11	0.903497343	0.711021745	0.239413245	2.111628879	0.539152282
ACAD8	0.274738481	0.391789565	0.042126012	3.643807111	0.410200294
ACAD9	0.687127806	1.374477995	0.51068336	3.699336823	0.52891697
ACADL	0.133225491	0.088615456	0.003898169	2.014458093	0.128374422
ACADM	0.761320364	3.339933487	0.089547432	124.5725925	0.513675409
ACADS	0.870796511	0.005666731	5.09E-07	63.03623312	0.276479599
ACADSB	0.296271581	1.64296557	0.708339187	3.810795612	0.247416591
ACADVL	0.820113	0.665683528	0.15672158	2.827527397	0.581323631
ACAT1	0.867964526	0.844489509	0.074129178	9.620537464	0.891690859
ACAT2	0.058587452	0.924038813	0.644970649	1.323855168	0.666723645
ACBD3	0.564416682	0.999067348	0.009287103	107.4754517	0.999688088
ACBD4	0.312103748	0.572970778	0.213771796	1.535728838	0.268239311
ACBD5	0.244978914	1.232263228	0.989825093	1.534081802	0.061696678
ACBD7	0.467934145	15.71791195	0.000421975	585467.3687	0.607964711
ACD	0.806426244	0.856847162	0.434087091	1.691335851	0.656107603
ACE	0.055875158	64.45837139	0.006395117	649695.9132	0.375741019
ACE2	0.59035525	0.939378473	0.246096357	3.585717101	0.92709169
ACHE	0.268760583	2.821748944	0.950627343	8.375802738	0.061657508
ACIN1	0.362766772	0.61015448	0.22295858	1.669765252	0.336131373
ACLY	0.076485101	1.79858879	0.72407456	4.467663707	0.206057963
ACMSD	0.405408712	1.353794526	0.487696726	3.757990405	0.560903723
ACN9	0.325225941	0.151384413	0.009416443	2.433746995	0.182761962
ACO1	0.442673465	0.189561671	0.000597293	60.1608145	0.571475798
ACO2	0.133958031	1.591546886	0.877842524	2.885507845	0.125822859
ACOT1	0.879108728	0.927218518	0.453114836	1.897386959	0.836134603
ACOT11	0.789730083	0.467875565	0.080186987	2.729963449	0.398664834
ACOT12	0.318411051	1.492570991	0.626542981	3.555650979	0.365836934
ACOT2	0.214517149	0.777422617	0.485801806	1.244099793	0.293943198
ACOT4	0.733078347	1.149111541	0.580887701	2.273171445	0.689654515
ACOT7	0.538373339	0.652183877	0.131239442	3.24097544	0.601313967
ACOT8	0.066609967	1.384458179	0.757710733	2.529625578	0.290153039

ACOT9	0.194519817	0.587655251	0.266055158	1.297996613	0.188555938
ACOX1	0.840962415	2.385826831	0.027211598	209.1817506	0.703233977
ACOX2	0.999595661	0.681088149	0.228989511	2.025774299	0.489825034
ACOX3	0.278949128	1.193512411	0.669380231	2.12804593	0.548809843
ACOXL	0.611443276	0.581277273	0.03640678	9.280778661	0.701119548
ACP1	0.387340753	2.366491433	0.000109596	51099.27043	0.865663467
ACP2	0.405068606	2.323338105	0.481411982	11.21264146	0.293858128
ACP5	0.633908749	3.497790841	0.941102399	13.0002227	0.061575738
ACP6	0.180387199	1.673284792	0.444801251	6.294681019	0.446338931
ACPP	0.050881093	0.403664396	0.117743876	1.383893164	0.148987707
ACPT	0.850069204	0.989135344	0.673137288	1.453475755	0.955636816
ACR	0.441999347	1.154958377	0.376908307	3.539133603	0.80092593
ACRBP	0.209872545	0.341761352	5.92E-06	19733.17066	0.847794317
ACRC	0.644337399	0.149938085	0.002161371	10.4014672	0.38034927
ACRV1	0.928723644	0.302828696	4.27E-06	21476.17142	0.833961233
ACSBG1	0.511272398	0.949131158	0.369760331	2.436307737	0.913561825
ACSBG2	0.773676063	10.92706487	0.004800841	24870.80032	0.544321989
ACSL1	0.221571013	0.767624833	0.316969872	1.859002813	0.557870252
ACSL3	0.766247814	2.675477191	0.018645547	383.9082001	0.697726977
ACSL4	0.492123443	1.531416715	0.663025576	3.537174491	0.318358872
ACSL5	0.299502372	0.789207729	0.306408999	2.032736773	0.623849282
ACSL6	0.604176216	1.035400443	0.814706151	1.315878218	0.77607674
ACSM1	0.662499307	0.915752293	0.513692265	1.632499298	0.765419355
ACSM3	0.512320277	0.446009762	0.145955432	1.362914049	0.156572406
ACSS1	0.727233566	1.047536098	0.291239869	3.76779416	0.943311401
ACSS2	0.815643506	0.631823508	0.060641392	6.582977932	0.700993254
ACTA2	0.798952899	1.669958677	1.107085485	2.519012325	0.014484622
ACTB	0.246206858	3.879817585	0.06945328	216.7353999	0.508904688
ACTG1	0.771332249	0.833476662	0.31716739	2.190273549	0.711751547
ACTG2	0.904245566	0.894842917	0.466412263	1.716815592	0.738218876
ACTL6A	0.786677785	1.338367987	0.719196947	2.490595761	0.35770062
ACTL6B	0.735878885	0.927927692	0.606034482	1.420793417	0.730743268
ACTL7A	0.882425757	0.482171868	0.000700411	331.933047	0.826808139

ACTL7B	0.54960297	1.08460853	0.836668191	1.406024126	0.539661496
ACTL8	0.588117665	0.973876116	0.281829896	3.36527353	0.966625111
ACTN1	0.406562749	1.555570318	0.0427128	56.65278401	0.809646515
ACTN2	0.702128827	1.80975173	0.213889998	15.31255014	0.5861426
ACTN3	0.898817465	1.165619773	0.864940738	1.570823752	0.314040148
ACTN4	0.650688554	0.34775864	0.000657216	184.0126023	0.741316524
ACTR10	0.370797012	1.826212181	0.430708159	7.743180298	0.413863544
ACTR1A	0.493730025	2.383561392	0.007340581	773.9666208	0.768462715
ACTR1B	0.304464953	0.707497088	0.439914596	1.137839331	0.153490739
ACTR2	0.153299728	23.12686393	0.944086514	566.5284135	0.054266054
ACTR3	0.950872733	1.46432065	0.04667699	45.93772983	0.828263948
ACTR3B	0.217104519	1.163934191	0.280279022	4.833550479	0.834467513
ACTR5	0.324140508	0.64653065	0.211197032	1.979203386	0.444855092
ACTR6	0.75271576	0.706481798	0.192744165	2.589528621	0.600083862
ACTR8	0.782524515	1.469846127	0.039733605	54.37330975	0.83439296
ACTRT1	0.751160779	1.531944346	0.618446214	3.794757612	0.356719363
ACVR1	0.595062999	1.189140375	0.915845013	1.543989225	0.19354134
ACVR1B	0.068462954	1.891942035	0.267720185	13.37009632	0.52276663
ACVR1C	0.8069834	15.62616197	0.32397948	753.6802576	0.164515861
ACVR2A	0.113260799	2.569930107	0.626214863	10.54676461	0.19011767
ACVR2B	0.936591019	76.07418285	0.158672238	36473.18123	0.168999081
ACVRL1	0.904708469	10.28935822	0.005174624	20459.62899	0.547468429
ACY1	0.831829993	1.552353067	0.509583625	4.728958949	0.439062052
ACY3	0.289327576	0.009513706	6.30E-06	14.35976128	0.212581967
ACYP1	0.969565506	1.948185014	0.00065026	5836.783406	0.870294639
ACYP2	0.409233559	1.49132625	0.006292881	353.4237962	0.886087063
ADA	0.154966852	1.037137582	0.765427603	1.405298631	0.81400496
ADAL	0.649897076	1.42099728	0.609379827	3.313587321	0.41601077
ADAM10	0.738163592	1.099950553	0.368018244	3.287584896	0.864589634
ADAM11	0.446664512	1.455033239	0.249099791	8.499090742	0.677065182
ADAM12	0.931616505	0.895399595	0.313805571	2.554895481	0.836376187
ADAM15	0.118287447	3.545258101	0.930989553	13.50053281	0.063575119
ADAM17	0.404219978	0.808654115	0.584980886	1.117851017	0.198585007

ADAM18	0.175883935	2.24545218	0.443439509	11.37033437	0.328375097
ADAM19	0.413041617	3.075506106	0.926300214	10.2113091	0.066516639
ADAM2	0.728523068	1.469636047	0.017083577	126.4272774	0.865482557
ADAM20	0.509630947	0.685620694	0.163201228	2.88034435	0.606285166
ADAM21	0.326527214	0.125447813	0.005522315	2.849738342	0.19265875
ADAM22	0.372886825	1.099723337	0.508474119	2.378471926	0.809149031
ADAM23	0.354086742	1.142926398	0.497159249	2.62748959	0.753110218
ADAM28	0.269624643	1.041378424	0.722876997	1.500212381	0.82767708
ADAM30	0.67363517	0.241365733	0.009001122	6.472239749	0.39695744
ADAM32	0.488749401	0.390214574	0.101748815	1.496503058	0.170014195
ADAM33	0.517933264	1.836607704	0.921804092	3.659267611	0.083905704
ADAM3A	0.278493587	0.969901516	0.57348913	1.640325683	0.909243794
ADAM5	0.255732236	0.409506713	0.124795172	1.343767907	0.140859082
ADAM6	0.499697569	0.727498845	0.371317378	1.425342849	0.353857177
ADAM7	0.627720513	1.210876643	0.374331422	3.916909345	0.749380436
ADAM8	0.937864143	0.507301161	0.007751133	33.20217627	0.750396429
ADAM9	0.464202356	0.858373269	0.676034636	1.089891892	0.210040395
ADAMDEC1	0.775513748	1.226247197	0.913346718	1.646343234	0.174801159
ADAMTS1	0.584337327	0.241884254	0.007089334	8.252960125	0.430658087
ADAMTS10	0.131723757	0.60432466	0.287472386	1.270411743	0.18398265
ADAMTS12	0.956255744	0.950299398	0.362590698	2.490601526	0.917407255
ADAMTS13	0.954252461	118.0814911	0.0463457	300852.9051	0.23311804
ADAMTS14	0.534708926	0.997852919	0.740789687	1.344120289	0.988716752
ADAMTS15	0.688017085	0.696707087	0.157789065	3.076263659	0.633402284
ADAMTS16	0.169188748	1.75046657	0.860971545	3.558925064	0.121987365
ADAMTS17	0.466598408	0.552228303	0.270153352	1.128825895	0.103572272
ADAMTS18	0.604273934	0.733423368	0.404197525	1.330809329	0.307795293
ADAMTS19	0.491103775	0.951583819	0.41004189	2.208339654	0.908018397
ADAMTS2	0.718014298	3.111234693	0.345879156	27.98602101	0.311200377
ADAMTS20	0.943121564	1.036522643	0.040789439	26.33964121	0.982661885
ADAMTS3	0.36023353	0.932659241	0.176909965	4.916926308	0.934492253
ADAMTS4	0.741918355	0.965357543	0.719199249	1.295767742	0.814400414
ADAMTS5	0.938285408	0.984258914	0.726787504	1.332942029	0.91832385

ADAMTS6	0.245474564	2.609278652	0.81040314	8.401170661	0.10792509
ADAMTS7	0.925054532	0.725195046	0.326415195	1.611162296	0.430163479
ADAMTS8	0.763221278	0.426304901	0.112811781	1.610965337	0.208762462
ADAMTS9	0.25726996	0.854499643	0.666588384	1.095383086	0.214622016
ADAMTSL1	0.761188015	0.095585623	0.00016744	54.5663691	0.468472554
ADAMTSL2	0.287869789	1.314319594	0.365898647	4.721077844	0.675265989
ADAMTSL3	0.251004552	2.099337104	0.924374977	4.767779727	0.076383921
ADAMTSL5	0.194665518	0.02969048	0.000123045	7.164243259	0.208944286
ADAR	0.929550529	1.988870784	0.318576524	12.41650497	0.461846225
ADARB1	0.48395938	1.148420828	0.030090668	43.82988171	0.940631881
ADARB2	0.849581246	1.078765869	0.341113333	3.411581101	0.897306308
ADAT1	0.980829873	0.933292474	0.683465075	1.274439432	0.664056173
ADCK1	0.228744122	4.15866296	0.02027511	852.9905708	0.599784062
ADCK2	0.064942586	0.158582787	0.018530734	1.357123788	0.092728245
ADCK4	0.609972848	1.090651504	0.43406452	2.740423712	0.853544956
ADCK5	0.35705457	1.89593267	0.077033265	46.66244774	0.69548624
ADCY1	0.854375443	2.209387686	0.53746727	9.082216194	0.271723589
ADCY2	0.594928742	0.868147244	0.559962816	1.345945866	0.527385756
ADCY3	0.511801592	0.965134875	0.720909571	1.292097323	0.811571196
ADCY4	0.832470356	1.716038094	0.01465421	200.9515849	0.824147172
ADCY5	0.16753943	0.617769385	0.150505634	2.535712476	0.503815169
ADCY6	0.276428272	0.383022435	0.030719187	4.775718345	0.456004901
ADCY7	0.947535049	1.520528295	0.34288863	6.742732461	0.58132314
ADCY8	0.705929429	1.126212539	0.768096655	1.651295673	0.542701874
ADCY9	0.117706951	1.351862754	0.916002835	1.995117082	0.128974339
ADCYAP1	0.892222476	0.979465916	0.038172526	25.1320409	0.990001163
ADCYAP1R1	0.629506091	0.956473174	0.296683858	3.083554796	0.940602523
ADD1	0.218024151	2.654663696	0.634055115	11.11455325	0.181439792
ADD2	0.835860885	2.283717969	0.001028132	5072.66396	0.833634635
ADD3	0.630779646	0.18973417	0.003817612	9.429732405	0.404264495
ADH1A	0.814790906	1.021150329	0.558365079	1.867502166	0.945822704
ADH1B	0.62886185	0.001434522	1.54E-08	133.8904519	0.262173642
ADH1C	0.845770287	1.06359244	0.006348287	178.1943478	0.981175507

ADH4	0.832705474	1.15699812	0.823015698	1.626511686	0.401389517
ADH5	0.793530992	26.96414289	0.836677942	868.9902835	0.062980742
ADH6	0.510124388	2.498308373	0.001219304	5118.94125	0.813936534
ADHFE1	0.426557929	0.077367068	0.002343627	2.554017111	0.151455698
ADI1	0.801962527	0.94682475	0.852117288	1.052058348	0.309544316
ADIPOQ	0.982303522	1.002829539	0.673983321	1.492124587	0.988880762
ADIPOR1	0.651456947	0.550316387	0.07979232	3.795454562	0.544382098
ADIPOR2	0.365276544	1.220088892	0.691502091	2.152729433	0.492309251
ADK	0.628418622	1.120198712	0.187100317	6.706803998	0.901069551
ADM	0.862438608	0.949248738	0.439380663	2.050780208	0.894570146
ADM2	0.402798544	347.3219817	0.002702796	44632502.44	0.329701282
ADNP	0.156564217	1.901985951	0.771634924	4.688163333	0.162491837
ADORA1	0.212985776	0.578023482	0.222777227	1.499754487	0.25982815
ADORA2A	0.267869201	1.089334317	0.940362239	1.261906535	0.254107996
ADORA2B	0.188135779	0.536399988	0.254832281	1.129075744	0.100948569
ADORA3	0.553711883	0.964243344	0.000516144	1801.368218	0.992440905
ADPGK	0.973995581	2.357889295	0.000716616	7758.189124	0.8355515
ADPRH	0.279249373	0.857257574	0.660079003	1.113337258	0.248129816
ADPRHL1	0.574921792	0.963262493	0.822029217	1.128761132	0.643583761
ADPRHL2	0.260181836	0.65979872	0.361232383	1.205136559	0.176093887
ADRA1A	0.391889433	1.562734558	0.980605108	2.490441136	0.060436868
ADRA1B	0.338326337	1.088604808	0.541305486	2.189263656	0.811757029
ADRA1D	0.544554302	2.23530924	0.029942048	166.875937	0.714703488
ADRA2A	0.254066704	2.051794399	0.255064058	16.5051097	0.499276613
ADRA2B	0.566997735	0.528620395	6.67E-05	4187.192196	0.889308744
ADRA2C	0.497319875	0.137061572	0.000838589	22.40177835	0.444705204
ADRB1	0.090360905	1.407553075	0.826569589	2.396901223	0.208150258
ADRB2	0.245986336	1.195006646	0.592052499	2.412017326	0.619066471
ADRB3	0.476455561	0.661449254	0.196755681	2.223646671	0.504046224
ADRBK1	0.725919705	2.227306088	0.732516374	6.772397973	0.158137247
ADRBK2	0.90521011	0.998872817	0.757115104	1.317827235	0.993635456
ADRM1	0.996219782	0.866862066	0.234818115	3.200135743	0.830229447
ADSL	0.446960053	16.85425333	0.333898434	850.7552788	0.158030172

ADSS	0.678079101	1.202367745	0.951211925	1.519838175	0.123178006
ADSSL1	0.258922039	2.051142392	0.424517576	9.910508663	0.37138835
AEBP1	0.87569401	0.75566001	0.296371174	1.926712518	0.557425317
AEBP2	0.497717932	1.499766505	0.332890171	6.756881899	0.597675125
AES	0.815519949	1.001735876	0.783598962	1.280597364	0.988956629
AFF1	0.218505338	0.846760528	0.589743892	1.215787737	0.367445928
AFF2	0.342764285	57.23468036	0.00494663	662230.3713	0.396543762
AFF3	0.234432953	5.625608463	0.151182808	209.3324702	0.349220478
AFF4	0.169182295	1.27823975	0.965232004	1.692750398	0.086707219
AFG3L2	0.100042844	1.212582746	0.86668691	1.696526047	0.260615162
AFM	0.072550587	2.518547523	0.978840632	6.480198528	0.055414631
AFMID	0.099385777	0.348666453	0.047847212	2.540760271	0.298445521
AFP	0.508059021	0.545694602	0.185607092	1.604370804	0.270980077
AGA	0.501431835	0.36533834	0.023469837	5.686963287	0.47218205
AGBL2	0.502297165	0.998736442	0.836839329	1.191954591	0.988820641
AGBL3	0.111263089	0.42246111	0.17402549	1.025558893	0.056885345
AGBL4	0.432328601	0.259876543	0.014534453	4.646602055	0.359723021
AGER	0.920159297	0.910116725	0.721057787	1.148746282	0.427924805
AGGF1	0.703062175	1.610672995	0.651875281	3.979699142	0.301698822
AGL	0.372640478	0.140731995	0.003045832	6.502491	0.316023668
AGMAT	0.468561108	1.368247612	0.770545399	2.429579789	0.284518625
AGPAT1	0.794834965	1.318661623	0.543750145	3.197918178	0.54053797
AGPAT2	0.117593153	1.076435879	0.935732385	1.23829657	0.302747333
AGPAT3	0.165198636	281.6183312	0.000624361	127024026.9	0.395801565
AGPAT4	0.708990736	0.714907036	0.31242993	1.635861424	0.426831129
AGPAT5	0.793581106	0.878008856	0.204499528	3.769688659	0.861080906
AGPAT6	0.77099176	1.016586259	0.794015511	1.301545886	0.896187049
AGPS	0.479099192	13.15342928	0.181028912	955.7186184	0.238652004
AGR2	0.645479729	0.100852718	4.32E-06	2354.549093	0.654851588
AGRN	0.778513765	0.124844086	0.001494275	10.4305063	0.356783767
AGRP	0.092698041	0.022276221	1.82E-05	27.26525933	0.294311935
AGT	0.138864796	2.152617472	0.519777499	8.914895306	0.290307418
AGTPBP1	0.499949735	1.001510596	0.741645703	1.352429428	0.992142007

AGTR1	0.067589011	0.650234224	0.195928425	2.157954095	0.48189687
AGTR2	0.937960313	0.982968426	0.690177518	1.399968706	0.924148318
AGTRAP	0.732373358	0.880328476	0.258998714	2.992208782	0.838207198
AGXT	0.996387785	0.838522244	0.189057264	3.719082456	0.816750612
AGXT2	0.09065186	0.467327304	0.1845225	1.183567366	0.108604081
AHCTF1	0.621292162	4.926650945	0.689312384	35.21174156	0.112019448
AHCY	0.267696441	0.439383453	0.18503905	1.043335548	0.06234721
AHCYL1	0.193108575	0.867514571	0.305578531	2.46280892	0.789497723
AHDC1	0.171256871	0.054581995	0.002083964	1.429580782	0.080905154
AHI1	0.781418523	0.884278213	0.517589776	1.510748459	0.652672225
AHNAK	0.70089948	1.030953969	0.803618682	1.322600023	0.810451484
AHR	0.30672962	0.768572307	1.38E-05	42692.80769	0.962336125
AHRR	0.843305398	0.903558424	0.275803431	2.960143835	0.866973658
AHSA1	0.635175511	0.745215713	0.000377766	1470.079779	0.939443678
AHSA2	0.460090219	0.421232033	0.119115751	1.489613459	0.17973346
AHSG	0.626006733	0.47622289	0.000575574	394.0211926	0.828652464
AICDA	0.100416368	0.146038719	0.008287868	2.573316389	0.188755603
AIF1	0.66556671	1.641329678	0.214355848	12.56771458	0.633298322
AIG1	0.935804066	1.051258205	0.04693141	23.54806312	0.974860813
AIM1	0.58251958	5055.308858	0.014566594	1754435304	0.190116371
AIM1L	0.524586217	1.084725022	0.930626989	1.264339404	0.298203899
AIM2	0.727804818	0.67736864	0.157231027	2.918178964	0.601144624
AIP	0.941636226	1.272659975	0.543670278	2.979128116	0.578471821
AIPL1	0.851065576	0.770709758	0.336638013	1.764487394	0.537715502
AIRE	0.339133076	5.71159309	0.198873136	164.0357079	0.309072935
AJAP1	0.714531346	17.27571238	0.000145037	2057753.798	0.632787309
AK1	0.803868123	3.05419807	0.946084473	9.859717733	0.061863425
AK2	0.776069878	0.75686542	0.465110034	1.231633853	0.262148812
AK3	0.629762455	0.13027154	0.001216715	13.94794677	0.392686701
AK5	0.52316666	0.357452028	0.091880682	1.390629133	0.137751982
AK7	0.278053507	0.534157803	0.180013437	1.585018118	0.258488926
AKAP1	0.21386481	0.321950547	0.007603638	13.63191686	0.553163783
AKAP10	0.258872119	0.140419655	0.018167037	1.085354725	0.059908825

AKAP11	0.970406693	0.980822089	0.754907781	1.274343694	0.884732662
AKAP12	0.895404344	1.135917523	0.700522937	1.841922014	0.605333085
AKAP13	0.278925201	0.749504023	0.262648733	2.138812071	0.589919226
AKAP14	0.601924341	1.664206257	0.007551778	366.7457643	0.853204323
AKAP2	0.498700138	5.223289299	0.000278363	98011.31592	0.741940216
AKAP3	0.723660341	0.899795944	0.403031773	2.008855864	0.796662644
AKAP4	0.390716292	0.579061924	0.144932493	2.31357858	0.439477974
AKAP5	0.199307758	3.692138312	0.075757522	179.9410145	0.510067075
AKAP6	0.94976284	1.140578746	0.733122763	1.774491178	0.559691785
AKAP7	0.454296846	1.245617888	0.404380837	3.836887856	0.701994095
AKAP8	0.939533308	0.706244016	0.195224373	2.554909531	0.596012366
AKAP8L	0.826164049	1.032548305	0.432828128	2.463231786	0.942439608
AKAP9	0.865476428	0.747887137	0.397324744	1.407753176	0.368013001
AKNA	0.139825871	0.522334233	0.166830494	1.635390774	0.264733427
AKR1A1	0.253310563	0.998503404	0.494192887	2.017449206	0.996669906
AKR1B1	0.721149597	0.979105891	0.489837211	1.95707538	0.95234932
AKR1B10	0.792521958	0.966272586	0.626458816	1.490413555	0.876687164
AKR1C1	0.192813861	0.260595641	0.036351217	1.868165451	0.180860741
AKR1C2	0.388684741	0.197231467	0.024956085	1.558748145	0.123775084
AKR1C3	0.86332014	1.151102066	0.641003919	2.067126155	0.637562995
AKR1C4	0.936249252	7.05133686	0.315765658	157.4628217	0.217747375
AKR1D1	0.178964293	1.215182156	0.783509423	1.884683999	0.384086732
AKR7A2	0.321859973	0.959578566	0.473808333	1.943382925	0.908764409
AKR7A3	0.125881814	0.564868319	4.74E-06	67359.23014	0.923702789
AKT1	0.799456506	0.660811529	0.2830536	1.542717977	0.338203495
AKT1S1	0.749125039	1.621584596	0.322676999	8.14912936	0.557312575
AKT2	0.710143158	1.124384224	0.431087292	2.932677224	0.810577577
AKT3	0.290182664	1.196549569	0.83076896	1.723380314	0.335061312
ALAD	0.751776315	23.48478056	0.000113765	4848008.395	0.613198122
ALAS1	0.535427833	1.393305255	0.345627426	5.616740427	0.64098927
ALAS2	0.307287622	1.015484704	0.441903257	2.333563214	0.971125392
ALB	0.722010732	0.802422685	0.379195329	1.698022406	0.564916029
ALCAM	0.253946378	0.066707437	4.84E-07	9192.972523	0.653847386

ALDH16A1	0.917297355	0.836441059	0.545735947	1.282000295	0.412361666
ALDH1A1	0.214784113	15.84806239	0.000786262	319436.7108	0.584794013
ALDH1A2	0.136382889	0.582572116	0.199040421	1.705132404	0.32410423
ALDH1A3	0.351845446	0.750969617	0.105277771	5.356832306	0.77511512
ALDH1B1	0.352679997	0.945212238	0.024918151	35.85443359	0.975768446
ALDH1L1	0.25176909	2.164756905	0.470800307	9.95363085	0.321110653
ALDH1L2	0.44904083	67.85093382	0.271948783	16928.73625	0.134244434
ALDH2	0.95686193	4.070264412	0.011571099	1431.761406	0.638887723
ALDH3A1	0.495587501	1.139259547	0.399367884	3.249916597	0.807405168
ALDH3A2	0.7448806	1.489979927	0.198707433	11.17240635	0.698066396
ALDH3B1	0.642804513	0.097069375	0.000361787	26.0442006	0.413671375
ALDH3B2	0.604092403	3.468506099	0.004262844	2822.185273	0.716048267
ALDH4A1	0.404614803	0.195045302	0.026340838	1.444246776	0.109573224
ALDH5A1	0.493823063	0.638198733	0.201728701	2.019036561	0.444705803
ALDH6A1	0.093815849	0.381631159	0.072934791	1.996884337	0.253918582
ALDH7A1	0.408084754	2.050418604	0.610020179	6.891930129	0.245691668
ALDH8A1	0.321191226	1.394112122	0.713108791	2.725458768	0.331343918
ALDH9A1	0.568608886	0.940006015	0.302970349	2.916494343	0.914711817
ALDOA	0.533877224	1.182615463	0.201538142	6.939526798	0.852615906
ALDOB	0.361043362	1.326401554	0.242919648	7.242481615	0.744314145
ALDOC	0.079274464	2.465940111	0.658719333	9.23133772	0.180203611
ALG1	0.752080744	0.933836715	0.797027075	1.094129721	0.397026872
ALG10	0.082983884	0.108661866	0.001064426	11.09274365	0.347005695
ALG12	0.765909185	1.143277112	0.668379526	1.955599331	0.624917346
ALG14	0.501092612	1.116486974	0.619244717	2.013005728	0.714077635
ALG2	0.518920147	0.003521337	8.08E-07	15.34680663	0.186424814
ALG3	0.739854006	0.975495583	0.386450201	2.462391348	0.958118224
ALG5	0.454769544	1.702761496	0.225255701	12.87157973	0.606046426
ALG6	0.888169334	0.17894808	0.012159054	2.633627182	0.209788257
ALG8	0.942403417	0.749105998	0.233753734	2.400645269	0.62685602
ALG9	0.128822281	0.475451695	0.141949848	1.592494238	0.228005399
ALK	0.579217651	0.664253781	0.327482895	1.347346969	0.256909295
ALKBH2	0.226738118	1.220550263	0.657168371	2.266912119	0.528081053

ALKBH3	0.443369205	1.100845237	0.956253109	1.267300701	0.181116569
ALKBH4	0.825821799	2.627425579	0.307606081	22.44222592	0.377399468
ALKBH5	0.981677736	0.615596606	0.049673324	7.629028086	0.705598836
ALKBH6	0.440785857	0.262074182	0.031200303	2.201352909	0.217475565
ALKBH7	0.263414599	1.118275391	0.865093048	1.44555531	0.393379195
ALKBH8	0.46002956	111.3086752	0.002648243	4678431.187	0.385647369
ALLC	0.142943095	0.816768585	0.430233991	1.550576978	0.5360189
ALMS1	0.897151677	1.203938641	0.485767195	2.983874304	0.688575186
ALOX12	0.952891414	1.995944666	0.517863522	7.692750962	0.31537587
ALOX12B	0.172857127	0.414706955	0.101016251	1.702516737	0.22189298
ALOX12P2	0.524539216	0.906965101	0.705025822	1.166745483	0.447320578
ALOX15	0.337453785	0.961241384	0.834341511	1.1074422	0.584228006
ALOX15B	0.337827859	0.000226407	4.28E-09	11.98422084	0.130425097
ALOX5	0.502504405	760.6089242	0.005934188	97490335.47	0.268917598
ALOX5AP	0.693557084	1.012816376	0.051818508	19.79595803	0.993300804
ALOXE3	0.610719909	0.190889322	7.23E-05	504.0829438	0.680362279
ALPI	0.256324643	0.606957108	0.113323056	3.250855951	0.559811148
ALPK3	0.929035632	0.784438205	0.162351885	3.790182657	0.76258247
ALPL	0.987331244	0.917387139	0.199151875	4.22591634	0.911901349
ALPP	0.316216215	1.561135094	3.29E-06	741729.8308	0.94675133
ALPPL2	0.146325569	1.091047736	0.839361057	1.418203943	0.514894672
ALS2	0.17323938	0.945437771	0.34603315	2.583141469	0.912878374
ALS2CL	0.428265304	0.735888218	0.154155323	3.512895032	0.700580742
ALS2CR11	0.878008688	1.066676992	0.363160287	3.133051293	0.906529521
ALS2CR12	0.339790706	1.064160768	0.279769801	4.047749741	0.927308476
ALX3	0.783746453	5.104361909	0.004377535	5951.868047	0.650943426
ALX4	0.284384894	1.95052943	0.748770381	5.081083809	0.171410867
AMACR	0.351731632	1.3920692	0.529280654	3.661302646	0.502574104
AMBN	0.459734525	1.039320834	0.860549204	1.255230719	0.688805167
AMBP	0.931256856	13.22281663	0.002972095	58828.16958	0.546900255
AMD1	0.467746014	2.880089051	0.284253941	29.18134718	0.370618048
AMDHD1	0.878939667	0.087318894	1.28E-05	597.5155199	0.588413346
AMDHD2	0.514152715	0.008271452	1.21E-05	5.665016532	0.150049792

AMELX	0.676048141	0.550912879	0.113283611	2.679160712	0.460050804
AMELY	0.567199767	0.93462931	0.80443001	1.085901739	0.377093019
AMFR	0.904543814	0.49666007	0.103795505	2.376511624	0.380920145
AMH	0.449175618	0.995310783	0.79470628	1.246553073	0.967352806
AMHR2	0.466959363	0.004638149	1.64E-06	13.11014139	0.185079171
AMICA1	0.309278132	1.054281131	0.56215936	1.977212836	0.86913772
AMIGO1	0.052051727	1.180170444	0.577188559	2.413080189	0.649864926
AMIGO2	0.961290137	1.078476449	0.283919786	4.096619926	0.911656765
AMIGO3	0.837459371	1.203918173	0.439028092	3.301426479	0.718421326
AMMECR1	0.230415873	1.099219629	0.801637397	1.507269741	0.556995186
AMN	0.716065778	0.321957749	0.01839107	5.636256749	0.437758694
AMOT	0.277184918	2.76450142	0.403871383	18.92302456	0.300143024
AMOTL1	0.271475705	0.27405254	0.047864247	1.569121002	0.145964095
AMOTL2	0.253624893	0.553349098	0.195741923	1.564280251	0.264380062
AMPD1	0.471912154	1.269349076	0.523651933	3.076942858	0.597537394
AMPD2	0.692365476	1.01616741	0.176334753	5.855885959	0.985680416
AMPD3	0.798764827	1.045076704	0.661060867	1.652170581	0.850343637
AMPH	0.443196699	1.214715796	0.462522481	3.19018972	0.69297083
AMT	0.138723811	0.46755335	0.034923503	6.259570665	0.565736274
AMY1A	0.33041578	5.176142624	0.899986364	29.76984267	0.065488704
AMY1B	0.143293622	0.791732342	0.35260765	1.777726893	0.571481904
AMY1C	0.936102688	1.080278309	0.738894374	1.579388432	0.690284938
AMY2A	0.569272647	0.639459173	0.307799276	1.328489265	0.230695566
AMY2B	0.249529602	0.565776063	0.149045936	2.147677172	0.402676806
AMZ1	0.224826216	52.19711472	0.144446426	18861.93284	0.188137747
AMZ2	0.700060332	0.735681072	0.36639427	1.477170044	0.388104392
ANAPC1	0.094130455	0.460492767	0.169030481	1.254528696	0.129391389
ANAPC10	0.514848574	0.965902374	0.519289635	1.796622411	0.91275446
ANAPC11	0.990126314	0.980675228	0.831995064	1.15592501	0.816054446
ANAPC13	0.878145592	0.945282899	0.13147941	6.796195357	0.955413815
ANAPC2	0.117725555	0.596341655	0.282487743	1.258898406	0.175093796
ANAPC4	0.117606321	2.362804935	0.581943876	9.593446031	0.229088631
ANAPC5	0.253772997	0.068341323	2.34E-05	199.2513449	0.509760569

ANAPC7	0.225655751	1.29057196	0.361273468	4.610291459	0.694558149
ANG	0.793549727	0.780100203	0.166957445	3.644978668	0.752223289
ANGEL1	0.92543491	2.432156153	0.588152133	10.05757391	0.219772806
ANGEL2	0.454496551	0.04876148	6.22E-05	38.22550956	0.37431691
ANGPT1	0.321415514	5.930107298	0.198004444	177.6029457	0.304763554
ANGPT2	0.834774301	0.022138096	1.05E-05	46.65662656	0.32914454
ANGPT4	0.424454013	2.980356503	0.914912041	9.708610759	0.069927776
ANGPTL1	0.147821703	1.383342258	0.912113216	2.098024422	0.126744654
ANGPTL2	0.419951126	0.677789456	0.231727358	1.982495945	0.477564898
ANGPTL3	0.395699467	1.312140465	0.365644263	4.708709462	0.676896036
ANGPTL4	0.718839151	1.104119402	0.332948546	3.661465612	0.871356695
ANGPTL5	0.912069529	0.0164209	8.01E-06	33.66906135	0.290904633
ANGPTL6	0.712467644	746.3719435	0.000889789	626070697.7	0.341820323
ANGPTL7	0.436702789	0.123480185	0.003285339	4.641030385	0.258297752
ANK1	0.882663551	4.902295949	0.012662177	1897.975835	0.601057312
ANK2	0.165301026	0.517112977	0.218879217	1.221704987	0.132721178
ANK3	0.405947836	1.936790782	0.037633526	99.6759784	0.742338659
ANKDD1A	0.825872665	1.210538579	0.465182616	3.150168554	0.695385715
ANKFN1	0.472129269	0.965789879	0.836112702	1.115579381	0.636085252
ANKFY1	0.97771141	0.148602928	1.24E-05	1774.966871	0.690614689
ANKH	0.699943074	1.057184563	0.904608368	1.235495094	0.484374183
ANKHD1	0.951245763	0.005794568	1.53E-07	219.777732	0.338310384
ANKK1	0.082412089	1.429418639	0.86610944	2.359098689	0.162222465
ANKMY1	0.251772346	0.42975973	0.046608404	3.962663598	0.456198363
ANKMY2	0.185927696	0.946165905	0.295575936	3.028764561	0.925729827
ANKRA2	0.474740894	1.117959413	0.963699236	1.296912152	0.141054082
ANKRD1	0.492217159	1.089279516	0.870815132	1.36255081	0.453987737
ANKRD10	0.085636436	1.654571109	0.575355702	4.75810972	0.350141998
ANKRD11	0.743495225	7.959352815	4.07E-05	1555762.541	0.73859748
ANKRD12	0.621041842	0.03606517	8.10E-06	160.5989244	0.438283264
ANKRD13B	0.062558782	1.453203529	0.990532843	2.131984326	0.055963229
ANKRD13C	0.248759992	0.610301894	0.145320184	2.563087884	0.500030645
ANKRD13D	0.555545493	0.687630194	0.000504267	937.6685621	0.918999856

ANKRD16	0.676365313	0.958575812	0.210581189	4.363483704	0.956368333
ANKRD17	0.110331881	1.314830427	0.979971627	1.764111333	0.067991839
ANKRD2	0.282578122	36.59226274	0.012440594	107631.0085	0.3770091
ANKRD20A2	0.620820336	0.919217431	0.762745475	1.107788527	0.376291495
ANKRD20A3	0.998826916	3.429929552	0.002049917	5738.973571	0.744832142
ANKRD22	0.647903677	1.673516316	4.16E-06	672679.5794	0.937660459
ANKRD23	0.769718109	0.89257818	0.61289953	1.299879943	0.55350851
ANKRD24	0.529263181	0.705867248	0.009817661	50.75023294	0.873125877
ANKRD26	0.330324913	0.624226807	0.363289851	1.072584621	0.087961043
ANKRD27	0.85379748	0.371838682	0.061479016	2.248962566	0.281322209
ANKRD28	0.450411223	0.948255479	0.278658539	3.226846942	0.932234549
ANKRD29	0.526283807	0.054234981	1.47E-05	199.8820806	0.486694626
ANKRD30A	0.409893387	1.181885819	0.539019518	2.591472189	0.676548653
ANKRD30B	0.115537663	0.715024216	0.427640365	1.195536417	0.200897862
ANKRD31	0.254555898	1.73725646	0.584921594	5.159768489	0.32002211
ANKRD32	0.285769068	0.939436815	0.864119829	1.021318456	0.14285745
ANKRD33	0.182556768	1.214632221	0.762269417	1.93544618	0.413363771
ANKRD35	0.862971096	1.278684913	0.381245326	4.288669258	0.690517692
ANKRD36	0.761897387	0.540595679	0.016059497	18.197562	0.731721174
ANKRD37	0.076224219	0.351285906	0.043126965	2.861360326	0.328282401
ANKRD39	0.874795382	1.536710478	0.783421501	3.014314885	0.211339507
ANKRD40	0.832981968	0.158863101	0.003807513	6.628338705	0.333835037
ANKRD42	0.331530152	0.694316105	0.179271346	2.68907924	0.597436042
ANKRD44	0.16701283	137.5316735	0.009657913	1958493.658	0.31294101
ANKRD45	0.858350978	1.282527075	0.559709714	2.938801412	0.556410106
ANKRD46	0.334425447	0.650781713	0.352631102	1.201019525	0.169420244
ANKRD49	0.749227023	543.407784	0.145818863	2025060.504	0.133340452
ANKRD50	0.670951793	0.890133994	0.696725532	1.13723194	0.351789769
ANKRD6	0.308314859	0.103482424	0.000116733	91.73606151	0.512445943
ANKRD7	0.578549495	0.723541964	0.012116299	43.20733469	0.876754068
ANKRD9	0.195160483	1.179620283	0.833377839	1.669715639	0.351428572
ANKS1A	0.174039516	1.10799056	0.93596132	1.311638693	0.233574377
ANKS1B	0.417854922	2.244276935	0.149125918	33.77534243	0.558978323

ANKS3	0.59904135	1.191936639	0.518827507	2.738314628	0.679068395
ANKS4B	0.09923217	0.116785355	0.005956374	2.289785578	0.157265943
ANKS6	0.443231011	0.102442922	8.24E-06	1273.47584	0.63573982
ANKZF1	0.307056973	0.959107945	0.673211487	1.366417638	0.817159748
ANLN	0.360981081	0.794891264	0.405760291	1.557205413	0.503452388
ANP32A	0.799793223	1.089353689	0.824199081	1.439811676	0.547584257
ANP32B	0.21292456	0.622598205	0.207713317	1.866170794	0.397529163
ANP32C	0.993047691	1.915470912	0.087772002	41.80181286	0.679455507
ANP32D	0.553390241	0.769729271	0.204992231	2.890271237	0.698237064
ANP32E	0.321434544	0.791056683	0.364280701	1.717825495	0.553570781
ANPEP	0.53574173	0.723565026	0.333475905	1.569967542	0.412961842
ANTXR1	0.158216275	9.74352259	0.022916036	4142.785988	0.460986589
ANTXR2	0.053992083	0.381560759	0.087862191	1.657010958	0.198465743
ANTXRL	0.16764676	0.422552294	0.170098024	1.049691449	0.063524098
ANXA1	0.859586752	1.985303788	0.084069617	46.88294406	0.67077017
ANXA10	0.611501128	20.24419698	0.072604554	5644.652956	0.295093067
ANXA11	0.569828709	1.018667645	0.013241996	78.36309413	0.993339994
ANXA13	0.665269458	1.886031268	1.02505987	3.470152379	0.041397517
ANXA2	0.725169337	155.6389935	0.085214146	284266.1406	0.187742337
ANXA2P1	0.610753754	0.962289764	0.770860071	1.201257691	0.734109881
ANXA2P3	0.902645843	0.813621552	0.394752503	1.676949544	0.576187247
ANXA3	0.890647469	0.947181871	0.402246995	2.230354753	0.901168176
ANXA4	0.994303151	2.828628657	0.331128098	24.16327735	0.342073165
ANXA5	0.03557234	0.420397926	0.196286843	0.900388498	0.025748239
ANXA6	0.259060933	0.487373107	0.22854044	1.039345796	0.062873102
ANXA7	0.081507509	0.112610695	0.002180378	5.816041206	0.277866628
ANXA8	0.873577215	0.654130393	0.120400929	3.553847745	0.623051855
ANXA9	0.893200773	0.602800885	0.112569897	3.227940305	0.554374176
AOAH	0.601349401	1.168205013	0.572747833	2.382729142	0.66901539
AOC2	0.338388499	0.634106603	0.191099413	2.104094296	0.456640698
AOC3	0.419693811	6.345905857	0.063723453	631.9576067	0.431199938
AOX1	0.576322617	0.956745988	0.726244967	1.260405135	0.753217597
AP1B1	0.576103416	1.04145555	0.214026087	5.06774515	0.959871311

AP1G1	0.139690844	1.262330419	0.584017914	2.728474672	0.553599808
AP1G2	0.949317267	1.312474667	0.098123058	17.55540224	0.837184801
AP1M1	0.412343199	0.861307496	0.341653082	2.171356391	0.751642449
AP1M2	0.693264106	0.928242811	0.644999458	1.3358689	0.688498779
AP1S1	0.958150595	1.086783603	0.493570582	2.39296798	0.836281412
AP1S2	0.892579932	0.869431795	0.03668818	20.60368344	0.930962656
AP1S3	0.690029338	0.075546235	0.000173059	32.97856889	0.404945857
AP2A1	0.646476848	0.842445091	0.000120045	5912.087001	0.969733245
AP2A2	0.955599944	0.557900718	0.116654607	2.668160473	0.464858706
AP2B1	0.771972674	0.883259386	0.296441252	2.631709107	0.823650901
AP2M1	0.730503542	0.22979676	0.000485064	108.8651323	0.63989473
AP2S1	0.390730912	0.574386149	0.265064822	1.244674589	0.159950725
AP3B1	0.378013102	0.118900245	0.001987985	7.111356818	0.307647486
AP3B2	0.978761318	1.024154985	0.938150741	1.118043601	0.593801615
AP3D1	0.321104217	0.797847639	0.537678172	1.18390682	0.262047848
AP3M1	0.645369075	1.000654259	0.054878444	18.24594287	0.999647706
AP3M2	0.70626957	1.008999007	0.343409016	2.964625126	0.987001842
AP3S1	0.556365537	19.71002062	0.026166879	14846.43686	0.377761162
AP3S2	0.141630679	0.151334903	0.007060463	3.243732535	0.227245599
AP4B1	0.627062649	0.102469207	0.000208259	50.41764421	0.471303365
AP4E1	0.957975061	0.824277863	0.396349013	1.714231585	0.604961255
AP4M1	0.425600297	0.689653953	0.384699262	1.23634907	0.212180389
AP4S1	0.992872334	1.162507505	0.283204211	4.771905392	0.834455279
APAF1	0.251735026	0.89262177	0.706743228	1.127387702	0.340339167
APBA1	0.880595812	1.63772715	0.677003497	3.961796698	0.273735636
APBA2	0.33798596	84.56984894	0.091571092	78103.89949	0.202749398
APBA3	0.935913239	0.14151317	4.95E-05	404.3605593	0.630087764
APBB1	0.935273558	0.968719251	0.865499699	1.084248773	0.580365409
APBB1IP	0.854879904	1.164497184	0.360548087	3.761089692	0.799042758
APBB2	0.262299131	16.6027602	0.012141266	22703.69793	0.445690635
APBB3	0.083257994	1.085950181	0.857102389	1.375900721	0.494672673
APC	0.52868381	1.141735334	0.326584976	3.991486651	0.835568286
APC2	0.097055863	32.05296624	0.381203907	2695.126224	0.12516507

APCDD1	0.138957059	0.638693604	0.291854911	1.397713403	0.261864097
APCS	0.237258448	0.698932572	0.333020012	1.466899054	0.343638079
APEH	0.944098719	0.894232364	0.513217971	1.558112862	0.693143349
APEX1	0.077704884	1.973811766	0.706178926	5.51692035	0.194770351
APEX2	0.077855892	1.522979452	0.842821102	2.75202698	0.163466438
APH1A	0.601875205	1.026737169	0.456709812	2.308225457	0.949098389
APH1B	0.391242994	1.021396118	0.482335454	2.16291384	0.955896829
API5	0.999876842	0.072818277	2.45E-05	216.425799	0.520824992
APIP	0.525084919	13.62946814	0.060950489	3047.759023	0.343951251
APITD1	0.331717025	1.397643024	0.761912712	2.563818653	0.27946485
APLN	0.777267572	0.412949195	0.135540415	1.258126867	0.119711677
APLP1	0.196694311	340.1803939	0.007289434	15875403.09	0.287889238
APLP2	0.264366579	3.850764716	0.004069739	3643.572638	0.699764823
APOA1	0.869141197	0.39619113	0.0046115	34.03825211	0.68365532
APOA1BP	0.088204931	1.749875214	0.643519501	4.758306873	0.272944823
APOA2	0.355363361	1.018273805	0.685039743	1.513607863	0.928652428
APOA4	0.811959814	1.161888096	0.324509468	4.16007569	0.817651054
APOA5	0.259357026	0.486057871	0.224956639	1.050212407	0.066457364
APOB	0.455941871	0.381662778	0.103952774	1.401275506	0.14662992
APOBEC1	0.169359778	0.611457126	0.171452683	2.180658886	0.448309135
APOBEC2	0.299971491	0.832343953	0.656263476	1.055668161	0.130218708
APOBEC3A	0.40302449	0.987268363	0.472016778	2.064966475	0.972850967
APOBEC3B	0.073090306	0.423209084	0.099173284	1.8059897	0.245433713
APOBEC3C	0.639160839	1.808353033	0.693739375	4.713788504	0.225541314
APOBEC3D	0.478630202	0.960138318	0.79769075	1.155667895	0.667101347
APOBEC3F	0.10083122	0.161049554	0.008569083	3.02680686	0.222458911
APOBEC3G	0.242976709	0.752390296	0.255129777	2.218836088	0.606135522
APOBEC4	0.925823426	1.030328329	0.465318074	2.281399592	0.941275252
APOC1	0.662784003	0.972110642	0.431535279	2.189853637	0.945575212
APOC2	0.669940177	54.02823248	4.38E-07	6657244373	0.674684892
APOC3	0.872023312	0.868866394	0.338003027	2.233497193	0.770434951
APOC4	0.077708515	1.502312857	0.967273064	2.333305874	0.070011147
APOD	0.576607064	0.765973302	0.330310971	1.776250721	0.5344346

APOE	0.448249511	0.973675666	0.001660597	570.9055591	0.993454914
APOF	0.314660311	0.880630004	0.526477328	1.473015385	0.628161895
APOH	0.407518092	1.129585794	0.099978009	12.76244727	0.921536918
APOL1	0.225478539	0.798524056	0.337332941	1.890241331	0.608825663
APOL2	0.846303386	1.00176968	0.144002674	6.968915645	0.998574504
APOL3	0.553685965	2.462326409	0.056416468	107.4695311	0.639988886
APOL4	0.248482787	2.87E-05	2.07E-11	39.62330116	0.147077692
APOL5	0.802431435	0.986095556	0.621398939	1.564831199	0.952609854
APOL6	0.430233605	1.124987241	0.578458714	2.187876612	0.728571181
APOLD1	0.921902502	0.261469836	1.60E-05	4266.343916	0.78635291
APOM	0.117833971	1.251815693	0.893764198	1.753306447	0.191354889
APP	0.689855934	1.119746599	0.761029322	1.647548142	0.56595729
APPBP2	0.70060795	7.945961497	0.120863371	522.3940342	0.331789237
APRT	0.984115519	0.749374049	0.387700353	1.448441976	0.390845332
APTX	0.671843374	1.321058627	0.225940207	7.724149301	0.75729969
AQP1	0.829701372	0.936405557	0.412400808	2.126221265	0.875211911
AQP10	0.675571785	2.018733739	0.125537324	32.46274322	0.620118978
AQP11	0.192591134	0.433450804	0.183069093	1.026276998	0.057304294
AQP12A	0.46669891	1.214672512	0.000321718	4586.097056	0.963088378
AQP2	0.225533219	0.474279994	0.124451867	1.807457921	0.274476463
AQP3	0.708478428	0.936191217	0.579434589	1.512602132	0.787650679
AQP4	0.142593863	2.070928718	0.527335108	8.132865973	0.296910507
AQP5	0.564844416	0.951370861	0.764994735	1.183153913	0.654063443
AQP6	0.830324779	1.091737322	0.982474273	1.213151747	0.102819585
AQP7	0.398549843	1.774372785	0.274662671	11.46278367	0.546885532
AQP7P1	0.77883336	0.382287445	0.000124697	1171.986018	0.814394538
AQP7P2	0.489446162	0.797869217	0.351379172	1.811704672	0.589417325
AQP8	0.449653704	1.029293596	0.850338924	1.245909455	0.767007797
AQP9	0.53485472	0.001569444	3.91E-09	630.1675247	0.326680963
AQR	0.411435615	0.366260071	0.067882144	1.976166808	0.242839287
AR	0.066609967	1.580087414	0.872207067	2.86248109	0.13130571
ARAF	0.314908742	0.739663787	0.361600536	1.513002506	0.408872038
ARC	0.536074936	0.845678494	0.390607496	1.83092266	0.670613921

ARCN1	0.562362569	0.818436	0.329724343	2.03150753	0.665780727
AREG	0.657218825	0.508425286	0.085595484	3.01997559	0.456802467
ARF1	0.538168334	7.667314506	0.267334135	219.9034988	0.23422489
ARF3	0.207570872	1.402758547	0.106987125	18.3922275	0.796595306
ARF4	0.765306718	1.036336264	0.545142489	1.970114005	0.913285441
ARF5	0.727558158	0.756613126	0.275486684	2.078007597	0.588465929
ARF6	0.071477942	0.874244777	0.612073234	1.248713206	0.459993247
ARFGAP1	0.302540493	1.094855619	0.829561214	1.444991407	0.522104481
ARFGAP3	0.474901289	1.006608113	0.376112077	2.69403711	0.989537716
ARFGEF1	0.853855472	0.915322118	0.625055619	1.340384047	0.649367653
ARFGEF2	0.123922265	1.396775396	0.519695089	3.754088785	0.507680734
ARFIP1	0.296779406	0.829536433	0.559649655	1.22957405	0.3519935
ARFIP2	0.231809323	1.141187871	0.82883119	1.571260557	0.418287098
ARFRP1	0.321603179	3.659428394	4.01E-05	334010.4424	0.823831696
ARG1	0.477159515	0.553538702	1.00E-05	30610.31722	0.915466523
ARG2	0.723941847	0.958858671	0.763568934	1.204095544	0.717682772
ARGFX	0.401772742	48.07019924	0.000302096	7649049.434	0.526266718
ARGFXP2	0.135191333	0.622124725	0.236659761	1.63542451	0.335821888
ARHGAP1	0.648866887	0.888675938	0.274515772	2.876865385	0.843894488
ARHGAP10	0.684804719	0.644040328	0.046279765	8.962619866	0.743276458
ARHGAP11A	0.418814561	0.661002266	0.200221114	2.182207397	0.496889737
ARHGAP12	0.213640234	1.562424885	0.998496217	2.444848041	0.050775018
ARHGAP15	0.050150129	1.355972309	0.81333207	2.260652161	0.242932549
ARHGAP17	0.305097811	0.890435155	0.520399885	1.523587509	0.671962055
ARHGAP18	0.810470904	1.234159395	0.304536276	5.001536861	0.768241064
ARHGAP19	0.204080191	0.564270017	0.261986319	1.215333125	0.143802113
ARHGAP20	0.983000711	1.088619184	0.001794197	660.5137838	0.979280999
ARHGAP21	0.796301526	1.108059708	0.561828226	2.185358906	0.767141664
ARHGAP22	0.657320151	26.94211632	0.028616713	25365.51381	0.345803045
ARHGAP23	0.225473436	0.487507408	0.191917562	1.238362294	0.130919212
ARHGAP24	0.055257411	1.425875841	0.148640591	13.67810705	0.758426602
ARHGAP25	0.276045338	1.509610837	0.50958732	4.472098869	0.457307509
ARHGAP26	0.519864657	1.400311131	0.468309398	4.187127724	0.546854507

ARHGAP27	0.546613211	2.174059653	0.172531338	27.39522815	0.548023143
ARHGAP28	0.318292915	2.420189782	0.646629011	9.058236608	0.189343481
ARHGAP29	0.494893309	1.167017712	0.464175998	2.934081789	0.742647498
ARHGAP30	0.510056786	0.934584687	0.425606284	2.052245396	0.866132431
ARHGAP4	0.459306715	1.606420698	0.210580133	12.25465775	0.647507457
ARHGAP5	0.170249803	0.604527515	0.012113842	30.16825874	0.800819486
ARHGAP6	0.692782108	18.97651857	0.10992255	3276.018041	0.262776043
ARHGAP8	0.675449523	0.914270948	0.788632206	1.059925477	0.234700476
ARHGAP9	0.487500312	0.571145092	0.155358508	2.099702943	0.399103445
ARHGDIA	0.946423392	1.243542218	0.314508062	4.916876341	0.755984885
ARHGDIB	0.298255671	1.001897667	0.325336512	3.085417406	0.997364131
ARHGDIG	0.277017133	1.370702212	0.980926053	1.915357991	0.064725371
ARHGEF1	0.956472934	10.41013216	0.000446063	242949.555	0.648004423
ARHGEF10	0.306296603	1.074167644	0.241032678	4.787052692	0.925238296
ARHGEF10L	0.631363632	1.461206235	0.127090131	16.80007443	0.760836028
ARHGEF11	0.185832921	0.35363115	0.109263188	1.144529939	0.08279577
ARHGEF12	0.551602361	0.948547973	0.329442539	2.731108316	0.922012616
ARHGEF15	0.815484636	0.810468634	0.271917048	2.415660993	0.706075331
ARHGEF16	0.133692356	0.801485728	0.589866504	1.089025006	0.157146091
ARHGEF17	0.238713112	0.663959565	0.407053697	1.083007736	0.100895118
ARHGEF18	0.972859583	0.719635648	0.290648848	1.781790875	0.476923728
ARHGEF19	0.130313272	0.638173669	0.136676748	2.979772633	0.567823109
ARHGEF2	0.17588185	0.580502563	0.284106414	1.186116217	0.135755495
ARHGEF3	0.857171646	1.14626981	0.365298458	3.596879338	0.815005973
ARHGEF4	0.771892037	0.343847661	0.000437388	270.3120542	0.75364721
ARHGEF5	0.43664103	1.004979206	0.890670922	1.133957762	0.93574273
ARHGEF6	0.312840975	0.268029877	0.048064645	1.494654022	0.133197
ARHGEF7	0.304298874	9.641142893	0.019915688	4667.256996	0.472510584
ARHGEF9	0.741478155	0.813375029	0.384722773	1.7196251	0.588667853
ARID1A	0.391211615	0.776513761	0.215002539	2.804495354	0.699457697
ARID1B	0.225472042	1.080278044	0.87738426	1.330090708	0.466906399
ARID2	0.139591492	0.003952751	1.07E-05	1.4635801	0.066692709
ARID3A	0.377501451	1.655294629	0.925617359	2.960186822	0.089255256

ARID3B	0.270851813	0.008231161	2.73E-05	2.483819881	0.099423602
ARID3C	0.086130648	0.088180467	3.31E-06	2349.040593	0.640448929
ARID4A	0.210662837	1.626760182	0.591605546	4.4731641	0.345756799
ARID4B	0.733822297	0.88187966	0.3026117	2.569998896	0.817831952
ARID5A	0.334474075	0.8940762	0.388134948	2.059521451	0.792560954
ARID5B	0.671976278	0.521057923	0.155462156	1.746414471	0.290779263
ARIH1	0.936903791	1.321121017	0.822389892	2.122303251	0.249547003
ARIH2	0.153987665	0.623240651	0.244379829	1.589447502	0.322242912
ARL1	0.231854637	1.012860952	0.881252843	1.164123686	0.857196847
ARL10	0.407945893	0.951096175	0.38061758	2.376621524	0.914546408
ARL11	0.580607886	5.328156556	0.231462654	122.6515457	0.295794217
ARL13A	0.367902725	0.747268569	0.27666233	2.018382166	0.565521333
ARL13B	0.259405468	0.377414465	5.45E-05	2611.243663	0.828992988
ARL14	0.348166748	0.846755521	0.000672053	1066.871846	0.963573669
ARL15	0.39560054	2.04612034	0.180737659	23.1640072	0.563091289
ARL16	0.606182445	0.393727659	0.081941522	1.891854898	0.244476308
ARL2	0.933284031	1.079162691	0.672045308	1.732907143	0.752550001
ARL2BP	0.37725499	0.000107623	1.75E-09	6.624979078	0.1043966
ARL3	0.430864308	0.024298262	0.000108509	5.441068738	0.178169755
ARL4C	0.102804072	1.190827969	0.016363979	86.65809779	0.936363458
ARL4D	0.225731524	2.118319286	0.073452633	61.09075231	0.661655205
ARL5B	0.464446904	1.120031611	0.624636268	2.008322081	0.703593017
ARL5C	0.561959738	0.475660223	0.041716658	5.423556396	0.549582302
ARL6	0.388577435	0.611092888	0.237488121	1.572434512	0.307096314
ARL6IP4	0.834064124	1.070954103	0.54867785	2.09037542	0.840782913
ARL6IP5	0.68549355	0.408299375	0.019297504	8.638856952	0.565128998
ARL6IP6	0.978500688	0.134599877	0.004068476	4.453050384	0.261293377
ARL8A	0.144386028	0.610742504	0.302555742	1.232851848	0.168864521
ARL8B	0.090195131	1.828779587	8.72E-05	38364.89122	0.905360435
ARL9	0.526688578	0.692901953	0.217897441	2.203390338	0.534239607
ARMC1	0.913025569	0.588905637	0.044284636	7.831380833	0.688379978
ARMC2	0.909739793	17.21869779	0.002856593	103789.2177	0.521619162
ARMC4	0.52106391	1.911481204	0.002771964	1318.112526	0.845957933

ARMC5	0.260068406	1.124209817	0.807411327	1.565308376	0.488141436
ARMC6	0.71235448	0.520908686	0.185169895	1.465388628	0.216510933
ARMC7	0.854116578	0.762641133	0.332352074	1.750016151	0.522558095
ARMC8	0.891349154	1.130414518	0.304333371	4.198806651	0.854722989
ARMC9	0.666984183	1.324376791	0.326301114	5.375323007	0.694271146
ARMCX1	0.065303621	2.527205077	0.43217014	14.77835906	0.30352101
ARMCX2	0.325534654	0.719544565	0.255724119	2.024620845	0.532909301
ARMCX3	0.710045795	1.025089333	0.435225957	2.414396762	0.954789501
ARMCX4	0.960399908	0.587554865	0.219765277	1.570861076	0.28920633
ARMCX5	0.156649335	2.046830428	0.503643826	8.318407937	0.316714132
ARMCX6	0.702880412	1.165432392	0.899661507	1.509715209	0.24634204
ARNT	0.072060201	0.929221218	0.68040261	1.269031099	0.644334451
ARNT2	0.606277855	0.875353192	0.221995072	3.451622609	0.849165764
ARNTL	0.53792359	0.593879789	0.314909972	1.119981059	0.107423082
ARNTL2	0.224415436	0.583641628	0.300419431	1.133873229	0.112022573
ARPC1A	0.332554665	2.906597831	0.411605065	20.52528426	0.284677495
ARPC2	0.233153744	2.903743781	0.896879975	9.401177618	0.075338414
ARPC3	0.245426037	1.099954658	0.714132215	1.694224437	0.665541727
ARPC4	0.557511712	1.117196911	0.240863464	5.181893991	0.88742427
ARPC5	0.485104895	5.268077695	0.057863625	479.621567	0.470345858
ARPC5L	0.96322586	1.001523279	0.814945732	1.230816776	0.98845414
ARR3	0.099586215	245825.5062	0.202479785	2.9845E+11	0.08247132
ARRB1	0.588696069	3.060437288	0.185643536	50.45301644	0.434048933
ARRB2	0.949829378	0.717740487	0.217086413	2.373024643	0.586732371
ARRDC1	0.580775066	1.014691403	0.350396363	2.938382791	0.9785522
ARRDC2	0.89697513	0.797729155	0.577653671	1.101649374	0.17001477
ARRDC3	0.76192835	0.780297599	0.247609249	2.458972539	0.671851437
ARRDC4	0.383735523	1.344843766	0.832124814	2.173477734	0.226412739
ARSA	0.945386724	0.78506776	0.277764774	2.218896869	0.648043644
ARSB	0.816852075	1.119717343	0.2894891	4.330964209	0.869858969
ARSD	0.066609967	1.911708798	0.838742758	4.357272231	0.123168887
ARSE	0.468350007	1.071601588	0.795648734	1.443262479	0.648957173
ARSF	0.816313326	1.74883723	0.138182291	22.13331133	0.666013248

ARSG	0.215287632	1.081934208	0.840863069	1.39211921	0.540335996
ARSH	0.448325647	0.70720366	0.407209353	1.228206113	0.218660471
ARSI	0.132130901	0.745772418	0.238353385	2.333411372	0.614242945
ARSJ	0.494785082	1.355083434	0.658345509	2.78919061	0.409369513
ARSK	0.635756014	0.783308526	0.188802063	3.249817491	0.736547969
ART1	0.210492914	5.205411732	0.025252999	1072.993805	0.543982294
ART3	0.128088315	1.750887376	0.480673205	6.377735582	0.395740872
ART4	0.609595557	0.01884316	1.85E-05	19.1735765	0.260991723
ART5	0.113632981	0.54340241	0.238177147	1.239775446	0.147267984
ARTN	0.27087194	0.94667444	0.297767367	3.009706891	0.926014624
ARV1	0.351348245	0.855334869	0.229409965	3.189040803	0.81597122
ARVCF	0.616832687	1.874384642	0.598250946	5.872648942	0.280915128
ARX	0.006810533	2.93007972	1.842841359	4.658766269	5.53E-06
AS3MT	0.766987814	0.127438857	0.000491955	33.01247228	0.467466716
ASAH1	0.269961867	0.913052074	0.470795166	1.770757539	0.78780714
ASAH2	0.732243007	12.13944263	0.000197482	746224.0708	0.65722063
ASB1	0.117169071	1.32898481	0.650228957	2.716274945	0.435502953
ASB10	0.38227021	0.06736725	0.003114094	1.457356946	0.085460127
ASB11	0.307956315	1.020757507	0.739592203	1.408811348	0.900542455
ASB12	0.364245167	0.824704173	0.084671973	8.032610457	0.868195309
ASB13	0.092823932	0.00117473	3.12E-08	44.23796092	0.209469621
ASB14	0.211320049	0.391618093	0.034825046	4.403862944	0.447689031
ASB15	0.850847464	1.34122913	0.064553704	27.86665168	0.849570406
ASB16	0.891585965	1.043933888	0.835292998	1.304689449	0.705468272
ASB17	0.436192089	0.634684713	0.31644472	1.272970158	0.200444975
ASB18	0.900809482	0.648732016	0.13950404	3.016781656	0.581054058
ASB2	0.215837491	1.096968982	0.528532143	2.276760199	0.803809968
ASB4	0.693194062	0.186108883	0.000878521	39.42593734	0.538347195
ASB5	0.469732105	0.484201121	0.02538603	9.235423177	0.629711434
ASB6	0.356818581	0.027048124	7.50E-05	9.749455975	0.229419728
ASB7	0.638514876	0.889856117	0.531637261	1.489443963	0.657021117
ASB8	0.425688576	357.90751	0.000769309	166510238.3	0.377165151
ASB9	0.103106369	0.026196218	6.08E-05	11.27781317	0.23919649

ASCC1	0.331089393	1.606309946	0.134103484	19.24060109	0.70833497
ASCC2	0.816977998	0.861609445	0.11859044	6.259955163	0.88296353
ASCC3	0.166458923	1.321302065	0.456503993	3.824367751	0.607374369
ASCL1	0.494879765	0.009117292	4.21E-06	19.75847477	0.230660823
ASCL2	0.071789417	1.122261204	0.856157573	1.471072908	0.403544178
ASCL3	0.198360707	0.08162438	0.00011376	58.56667275	0.455171721
ASCL4	0.994509111	0.485098087	0.110767829	2.124444944	0.337052499
ASF1A	0.343317813	1.654989555	0.506341717	5.409371444	0.404432591
ASF1B	0.876450671	1.440290854	0.690920446	3.00242634	0.330322581
ASGR1	0.177501795	0.361312229	0.01211101	10.77916088	0.556801269
ASGR2	0.533426563	0.849948333	0.394824144	1.829706161	0.677707232
ASH1L	0.560677587	0.654913839	0.17529234	2.446839011	0.529098091
ASH2L	0.141898539	0.001981315	2.03E-06	1.932059949	0.076325337
ASIP	0.493264173	1.195195842	0.495807562	2.881144237	0.691225044
ASL	0.487922968	0.74565459	0.365781541	1.520035061	0.419287382
ASMT	0.698633907	0.61118932	0.301076263	1.240723465	0.172916998
ASMTL	0.576269616	0.246225272	0.000850379	71.29396576	0.627955344
ASNA1	0.58173933	13.60536692	0.000336963	549336.3369	0.62951641
ASNS	0.269881978	0.724263938	0.278868471	1.881023878	0.507662356
ASNSD1	0.403121559	3.02959214	0.928511263	9.88510199	0.066204734
ASPA	0.741362079	0.912620966	0.478119327	1.741985694	0.781614901
ASPH	0.301789527	1.515776934	0.644390147	3.565510312	0.340573528
ASPHD1	0.830987566	111.1340012	0.001091492	11315485.13	0.423303044
ASPHD2	0.632100912	1.459541559	0.529181311	4.025579735	0.465096656
ASPM	0.505408665	48.62391048	0.00219898	1075173.096	0.44667044
ASPN	0.544738909	0.014537216	7.83E-05	2.698266226	0.112393218
ASPSCR1	0.029887161	0.449356235	0.201897281	1.000117609	0.050033685
ASRGL1	0.581263525	0.93279448	0.390724076	2.226905365	0.875483349
ASTE1	0.666903775	0.65022303	0.05892342	7.175245205	0.725316954
ASTL	0.751868103	1.263362129	0.461914965	3.455362978	0.648826923
ASTN2	0.299664545	0.005879247	5.98E-06	5.78284193	0.144057658
ASXL1	0.175875637	0.040985783	0.000555208	3.025593323	0.145522479
ASXL2	0.449178867	13.84597241	3.85E-05	4977376.303	0.687211081

ASZ1	0.128178343	0.690021625	0.433629941	1.098009611	0.117472748
ATAD1	0.947649114	0.542107151	0.146245723	2.00949578	0.359687397
ATAD2	0.434638295	0.001812794	2.77E-06	1.188347903	0.056415324
ATAD3A	0.324408722	59.86255367	0.063466548	56463.21481	0.241613435
ATAD3B	0.230369931	1.239545592	0.572228017	2.685071738	0.586084944
ATCAY	0.939368956	1.20543715	0.684113792	2.124030739	0.517979419
ATE1	0.325670914	1.8765944	0.568767489	6.191645284	0.301376355
ATF1	0.683829461	0.895975089	0.563896353	1.423615095	0.641973096
ATF2	0.254112153	0.72678618	0.000454924	1161.113555	0.932424398
ATF3	0.373294716	0.856557035	0.410863596	1.785726364	0.6795514
ATF4	0.04733989	2.388561762	1.172733539	4.864896501	0.01644083
ATF5	0.960423149	7.14421902	6.87E-05	742657.7012	0.738665657
ATF6	0.878250985	4.20776384	0.037352142	474.0096761	0.551082885
ATF7	0.831155771	0.031943097	6.55E-06	155.8396499	0.426744744
ATF7IP	0.12190865	1.984461757	0.732711345	5.374679255	0.177601747
ATF7IP2	0.354883679	1.083527614	0.743894725	1.578223439	0.675885347
ATG10	0.902397008	0.991650852	0.50634297	1.942105392	0.980495458
ATG12	0.752042696	0.543246247	0.232723174	1.268100983	0.158302944
ATG16L1	0.89039407	1.166388358	0.475288971	2.862388742	0.73685402
ATG16L2	0.636012155	0.716442703	0.202804402	2.530961562	0.604558583
ATG3	0.837162653	0.157068502	0.00046257	53.33354736	0.533575723
ATG4A	0.733223121	0.843892745	0.056041975	12.70752799	0.902370563
ATG4B	0.936338706	0.053566682	0.000434149	6.609227137	0.233534458
ATG4C	0.78537398	0.948182701	0.320804854	2.802483891	0.92333784
ATG4D	0.234505129	1.112462992	0.001343742	920.9905844	0.975198208
ATG5	0.134006358	0.000643973	1.67E-07	2.481496047	0.081120721
ATG7	0.064526687	6.64600721	0.011476575	3848.657819	0.55952611
ATG9A	0.264506841	0.34604745	0.067925129	1.762953417	0.201450406
ATG9B	0.083830396	8.439483313	0.439715197	161.9795702	0.157092166
ATHL1	0.300453392	2.69942922	0.051831635	140.5882344	0.622442484
ATIC	0.449281982	1.092997532	0.435964956	2.740228519	0.849602901
ATM	0.542346001	1.168783067	0.762794793	1.790853675	0.473783223
ATN1	0.058899409	0.448803634	0.158178889	1.273398131	0.132136462

ATOH1	0.200094627	0.51888607	0.16440746	1.637655339	0.263226307
ATOH7	0.581684963	0.009903896	2.53E-06	38.78331656	0.274250232
ATOH8	0.85473953	1.030283042	0.704245689	1.507262541	0.877855057
ATOX1	0.065230695	1.967056785	0.718485485	5.385373089	0.187979527
ATP10A	0.759703578	0.60534846	0.004923128	74.43373136	0.837998435
ATP10B	0.327227607	0.792856346	0.297133213	2.115620733	0.64298691
ATP10D	0.231849722	15.11756036	0.018392843	12425.51933	0.427722011
ATP11A	0.986799181	1.025563953	0.901463648	1.166748568	0.701282655
ATP11B	0.798555556	0.857783215	0.569500629	1.291995137	0.462910205
ATP11C	0.613827995	1.969001418	0.000299914	12926.92334	0.879912275
ATP12A	0.684112774	1.079115915	0.877391546	1.327219488	0.470822878
ATP13A1	0.532373385	1.264773232	0.510753166	3.131945986	0.611649359
ATP13A2	0.951924059	10.8408665	0.000293102	400967.2556	0.656967215
ATP13A3	0.680316116	1.038127765	0.845691343	1.274352948	0.720552929
ATP13A4	0.513186566	0.913324371	3.29E-05	25387.10938	0.986144778
ATP13A5	0.301579884	0.167667572	0.001230905	22.83881537	0.476324345
ATP1A1	0.543111697	0.833859396	0.312286049	2.22655317	0.716918205
ATP1A2	0.631675528	1.20936781	0.600950367	2.433762556	0.594195762
ATP1A3	0.605966441	0.878023583	0.50994409	1.51178419	0.638919608
ATP1A4	0.865744959	0.995595027	0.845963889	1.171692399	0.957629796
ATP1B1	0.722040022	1.272579964	0.003895091	415.7694323	0.934957551
ATP1B2	0.940362787	1.069164549	0.393110968	2.90786299	0.895769987
ATP1B3	0.515622141	1.696765908	0.485190334	5.933783803	0.407817374
ATP1B4	0.356727544	1.023621472	0.769850982	1.361043816	0.872399802
ATP2A1	0.751970241	0.076351497	4.23E-06	1379.764802	0.606998142
ATP2A2	0.065369076	1.391508115	0.8817711	2.19591551	0.155780471
ATP2A3	0.41751601	0.735402423	0.280910711	1.92522642	0.531369629
ATP2B1	0.446925592	2.265812479	0.599487415	8.56382647	0.2279318
ATP2B2	0.886973079	1.163668228	0.696017689	1.945530649	0.563239705
ATP2B3	0.159060617	0.814460961	0.401305071	1.652973523	0.569835426
ATP2B4	0.555142461	0.169788091	8.28E-06	3482.429396	0.72631036
ATP2C1	0.0849943	1.351768151	0.701633843	2.604317269	0.367651216
ATP4A	0.813547208	0.812446624	0.212139658	3.111485718	0.761762509

ATP4B	0.56192019	2.20971908	0.000271651	17974.77844	0.862972708
ATP5A1	0.507214179	0.115552982	0.001448331	9.219228766	0.334131853
ATP5B	0.669937022	6.064271272	0.237727672	154.695437	0.275426367
ATP5C1	0.82163241	6.541573656	2.51E-05	1702407.442	0.767828795
ATP5D	0.078328385	2.46583677	0.324501185	18.73753087	0.383069851
ATP5E	0.143669032	0.034473762	0.000608716	1.952373189	0.102025939
ATP5F1	0.658785561	0.763069568	7.15E-05	8149.024374	0.954437784
ATP5G1	0.454015311	1.046296107	0.120814584	9.061286383	0.967224995
ATP5G2	0.460592688	3.540764712	0.006892851	1818.843126	0.69135024
ATP5G3	0.288908514	0.015380009	8.07E-05	2.931350938	0.119120332
ATP5H	0.380779766	1.13815046	0.644174376	2.010925177	0.655889998
ATP5I	0.756146528	1.072205881	0.714072062	1.60995719	0.73675123
ATP5J	0.633729905	1.123401844	0.217737617	5.796112402	0.889455241
ATP5J2	0.291503972	1.155736665	0.887826689	1.504490973	0.282059515
ATP5L	0.181753004	0.023099284	4.37E-05	12.22174653	0.238948283
ATP5O	0.867569546	1.027691679	0.878726548	1.201909956	0.732443518
ATP5S	0.204606921	1.636270477	0.940436544	2.846955586	0.081397422
ATP6AP1	0.726982819	0.609470734	0.131708066	2.82028721	0.526415558
ATP6AP2	0.745065406	1.046145399	0.464784973	2.354680679	0.913214525
ATP6V0A1	0.677729436	0.420602339	0.006244985	28.32774136	0.68679661
ATP6V0A2	0.942796726	2.387388507	0.371569258	15.33933114	0.359216164
ATP6V0A4	0.37108534	0.230441151	0.021616696	2.45657907	0.224136854
ATP6V0B	0.458588713	0.000297872	4.57E-11	1941.23928	0.310489679
ATP6V0C	0.497512777	0.969915121	0.237141729	3.96697513	0.966096115
ATP6V0D1	0.194332867	144.4351832	0.02765064	754467.967	0.254914572
ATP6V0D2	0.982538016	1.073364236	0.470929391	2.446461837	0.866244005
ATP6V1A	0.937114483	0.83481405	0.267105592	2.609134809	0.756161226
ATP6V1B1	0.730764925	3.061810389	0.416233833	22.52263537	0.27173707
ATP6V1B2	0.067253442	0.010848227	1.01E-05	11.65250203	0.203946071
ATP6V1C1	0.743367533	1.113879203	0.20980467	5.913723852	0.899242723
ATP6V1C2	0.472646261	0.725799013	0.224022903	2.351474783	0.593104254
ATP6V1D	0.30404349	12.00312356	0.025043709	5752.940855	0.430027422
ATP6V1E1	0.049105093	0.573283685	0.338769103	0.970142144	0.038181513

ATP6V1E2	0.489409257	0.426508117	0.09477958	1.919286548	0.266825392
ATP6V1F	0.18268721	1.925137435	0.741426469	4.998680648	0.178488663
ATP6V1G1	0.72738892	1.637070254	0.006919309	387.3217679	0.859718357
ATP6V1G2	0.346336125	1.112910703	0.476966535	2.596765482	0.804547829
ATP6V1G3	0.083765105	1.311334144	0.890424426	1.931210764	0.169955333
ATP6V1H	0.832723249	0.606561655	0.202404388	1.817732537	0.371964664
ATP7A	0.132799464	5.13031902	0.091911254	286.3650768	0.425558373
ATP7B	0.436994244	1.597278947	0.628809364	4.057350577	0.324830199
ATP8A1	0.530304633	1.441022245	0.54209747	3.830575172	0.463900471
ATP8A2	0.13573542	81.14174484	0.057473927	114555.9936	0.234818493
ATP8B1	0.401319587	0.552495554	0.223762824	1.364173596	0.198248637
ATP8B2	0.312215866	0.651732556	0.386644802	1.098567269	0.108036603
ATP8B3	0.863662584	7.030407993	0.447075081	110.5555615	0.16534825
ATP8B4	0.48544061	1.04E-05	2.56E-11	4.260285996	0.081844846
ATP9A	0.82954823	1.235682579	0.262044522	5.82691607	0.789124797
ATP9B	0.572517062	1.045636891	0.791813443	1.380825896	0.7530944
ATPAF1	0.168314593	0.389024522	0.135237112	1.119072099	0.079897286
ATPAF2	0.618304104	0.767370258	0.394127713	1.494076904	0.436043908
ATPIF1	0.795003482	1.043835384	0.822895667	1.324095329	0.723669808
ATR	0.257887035	0.481147873	0.208836161	1.108540181	0.085799549
ATRN	0.749239089	2.766465031	0.090380552	84.67893328	0.559935534
ATRNL1	0.106476646	0.563718738	0.231168038	1.374665885	0.2075586
ATRX	0.831043489	1.170348617	0.866738849	1.580309785	0.304612172
ATXN1	0.314144052	1.698233142	0.858338766	3.359973845	0.128212878
ATXN10	0.781798178	0.251854437	0.000445128	142.499853	0.669819332
ATXN2	0.386003309	1.918986597	0.523528183	7.034023537	0.325372655
ATXN2L	0.067635317	0.107802187	0.00015125	76.83504333	0.506316087
ATXN3	0.155904225	171.1700911	0.20326469	144143.0876	0.134556517
ATXN3L	0.516106717	2.0987888	0.023266762	189.3221973	0.746886231
ATXN7	0.971711693	0.387110239	0.027949976	5.361519263	0.479119178
ATXN7L2	0.460709891	0.937376447	0.284121027	3.092606735	0.91543551
AUH	0.129822422	0.022433859	0.000303363	1.658995618	0.083735444
AUP1	0.400406337	0.005773065	1.74E-06	19.11424859	0.212586718

AURKAIP1	0.643057963	0.716262322	0.189684156	2.704662977	0.622536503
AURKB	0.268178335	0.008529178	3.73E-05	1.949601566	0.08560203
AURKC	0.131738337	0.183096887	0.022294682	1.503698071	0.11404656
AUTS2	0.995668045	0.881916901	0.524677523	1.482391348	0.635322468
AVEN	0.30764557	0.747048462	0.324935604	1.717513864	0.492351226
AVIL	0.331816166	1.302546993	0.150140956	11.30023887	0.810495242
AVP	0.275282405	0.561685164	0.001941599	162.4898589	0.841887915
AVPI1	0.706665452	0.77797194	0.004575746	132.2713898	0.923670631
AVPR1A	0.19463594	0.991212405	0.661479409	1.485310077	0.965882582
AVPR1B	0.875312675	1.259421806	0.470340927	3.372326743	0.646249547
AVPR2	0.650775712	0.240373191	7.63E-05	757.5716077	0.728709745
AXIN1	0.774583753	0.963620891	0.562067544	1.652052733	0.892823403
AXIN2	0.72651423	0.122147103	8.55E-05	174.4861158	0.570528899
AXL	0.658003272	0.551702901	0.080679064	3.772677491	0.544298403
AZGP1	0.727212952	0.933586493	0.719240075	1.211811981	0.605588358
AZI2	0.103812176	0.196870568	0.034843284	1.112352684	0.065848966
AZIN1	0.468906799	0.495601702	0.155044445	1.584197657	0.236419584
AZU1	0.073762504	0.382208164	0.104132028	1.402864071	0.147137857
B2M	0.519398415	1.157500822	0.725643074	1.846373517	0.539276105
B3GALNT2	0.749408996	0.885507473	0.323334956	2.425112007	0.813003536
B3GALT1	0.777488047	0.715566411	0.235698952	2.172412241	0.554734215
B3GALT2	0.925605782	0.865052989	0.500090423	1.496362736	0.604126929
B3GALT4	0.073542645	1.234724923	0.882397451	1.727731234	0.218671493
B3GALT5	0.357617185	1.218490926	0.760976285	1.951072808	0.410660162
B3GALT6	0.987028313	0.545172801	0.007579194	39.21437719	0.780945298
B3GAT1	0.795088495	0.914388035	0.412217992	2.028309039	0.82573169
B3GAT2	0.697518331	0.976618542	0.586263027	1.626887134	0.92759963
B3GAT3	0.588930109	0.871712109	0.251462566	3.021849386	0.828629315
B3GNT3	0.957101161	307.8576337	0.006126903	15468878.58	0.299535025
B3GNT4	0.197045277	0.036462163	5.50E-08	24169.76608	0.628243861
B3GNT5	0.722304412	0.095138667	3.43E-05	264.1534092	0.560905748
B3GNT6	0.782340804	1.110122206	0.386799764	3.186070491	0.846012567
B3GNT7	0.549431676	1.165164034	0.250632865	5.416716699	0.845412653

B3GNTL1	0.47525498	0.026026429	0.000100827	6.718214731	0.197849709
B4GALNT2	0.312356864	1.874326009	0.010475997	335.3473727	0.812350769
B4GALNT3	0.990898226	1.984926742	0.006182852	637.2356929	0.815903603
B4GALNT4	0.672566915	0.022938042	2.57E-06	204.5920538	0.415981487
B4GALT1	0.187867362	0.166007193	0.024976388	1.10337765	0.063145292
B4GALT2	0.478757721	0.886481739	0.276977082	2.837237897	0.839128148
B4GALT3	0.37571751	1.955776105	0.665750967	5.745481963	0.222459957
B4GALT4	0.502767711	0.009157678	1.28E-05	6.571879728	0.161874184
B4GALT5	0.25768454	0.878362685	0.358984929	2.149173805	0.7763405
B4GALT6	0.975624947	13.0244427	0.00062856	269880.692	0.612729315
B4GALT7	0.348582974	0.002759727	1.86E-07	40.93544549	0.229178961
BAALC	0.82056758	0.533595303	0.036402578	7.821532535	0.646588924
BAAT	0.221775807	0.179583173	0.002032229	15.86933493	0.452669237
BACE1	0.54195956	0.059619072	0.000388144	9.157502093	0.272295837
BACE2	0.500304375	0.747570581	0.418112912	1.336628834	0.32644948
BACH1	0.594102301	0.629217163	0.168787218	2.345641111	0.490155054
BACH2	0.190848955	46496.32398	0.02103965	1.02754E+11	0.14933011
BAD	0.076685045	0.205670362	0.017309046	2.443826089	0.21043915
BAG1	0.662616625	1.297076559	0.356630571	4.717508079	0.692956804
BAG2	0.52676833	1.032880696	0.736256695	1.449008939	0.85142119
BAG3	0.721984198	0.94655424	0.715389106	1.252416233	0.700622967
BAG4	0.184270278	0.745296302	0.402119177	1.381348143	0.350414122
BAG5	0.539456591	1.901620199	0.600284733	6.024073546	0.274626395
BAHD1	0.153954176	4.062624246	0.836703968	19.7261115	0.082066358
BAI1	0.992529692	1.072803842	0.440466607	2.612929256	0.877036267
BAI2	0.222897297	601.0198333	0.809461107	446253.4852	0.057790354
BAI3	0.205420568	0.764685022	0.364622706	1.603693826	0.477691914
BAIAP2	0.409230418	0.988137781	0.478638601	2.039986478	0.974260298
BAIAP2L1	0.393060425	0.648899763	0.227914299	1.847496644	0.417869078
BAIAP2L2	0.059060495	21.27197008	0.131197437	3448.975242	0.238938454
BAIAP3	0.085095203	1.648082244	0.812896016	3.341356127	0.165899594
BAK1	0.852528647	0.003862176	1.24E-07	120.637945	0.292661919
BAMBI	0.247930208	0.481119889	0.047062185	4.918521052	0.537325901

BANF1	0.467024363	1.200035926	0.369646995	3.895841831	0.761500163
BANK1	0.100019336	220.8965744	0.376934593	129452.954	0.096930892
BANP	0.058337242	5.363202125	0.000136488	210743.3134	0.755666832
BAP1	0.344892415	1.05710158	0.332981378	3.355934671	0.924937508
BARD1	0.966380136	0.895367478	0.377633263	2.122913947	0.801880112
BARHL1	0.367624342	1.341216301	0.634705212	2.834167945	0.441848688
BARHL2	0.616057962	0.833747503	0.381288419	1.823120932	0.648751194
BARX1	0.865326678	0.642977119	1.85E-05	22384.69913	0.934032891
BARX2	0.279923109	0.701695293	0.190597604	2.583328822	0.594218646
BASP1	0.486847418	0.671363141	0.118097573	3.816576888	0.653155101
BATF	0.392138982	0.392340457	0.028305548	5.438193106	0.485486592
BATF2	0.987501335	0.557943937	0.143275441	2.172748066	0.400223503
BAX	0.807517021	0.651344634	0.292906885	1.448411949	0.293071195
BAZ1A	0.083801022	0.627272418	0.198392136	1.983297793	0.427157271
BAZ1B	0.163502785	0.000401688	9.08E-08	1.776450599	0.067880326
BAZ2A	0.212749699	0.269300947	0.01044367	6.944206216	0.428816693
BAZ2B	0.505069611	1.303478258	0.842992853	2.015504121	0.233307556
BBC3	0.590589216	0.967375097	0.493547572	1.896098027	0.923042462
BBOX1	0.492456505	0.258042028	0.005765356	11.5492753	0.484888479
BBS1	0.086028053	0.007150162	3.85E-07	132.7405883	0.324531324
BBS2	0.776484853	0.739379081	0.309875888	1.764194785	0.496179825
BBS4	0.000421943	0.270682686	0.106099794	0.690567945	0.006242367
BBS5	0.219676351	3595.549504	0.782369195	16524137.61	0.057050768
BBS7	0.2761294	23.31100277	0.004823625	112654.4601	0.466899128
BBX	0.796901428	1.416822565	0.587274933	3.418137002	0.438099441
BCAM	0.798489258	2.858668374	0.397826585	20.54157559	0.296534053
BCAN	0.251032283	0.636018254	0.275693279	1.467279945	0.288685525
BCAP29	0.624091072	0.735196243	0.15919105	3.395376283	0.693539022
BCAP31	0.879558077	0.007095454	5.95E-06	8.462603178	0.170974854
BCAR1	0.985588736	0.499543322	0.149931866	1.664379551	0.258348674
BCAR3	0.169844531	47.33401694	0.007326872	305793.411	0.388855092
BCAS1	0.127542779	1.417264172	0.685698498	2.929330805	0.346502949
BCAS2	0.265505617	1.271113579	0.564057091	2.864479071	0.562797816

BCAS3	0.14071159	1.147952324	0.475954823	2.768738701	0.758714682
BCAS4	0.207815228	1.590433956	0.511533276	4.944898578	0.422711828
BCAT1	0.182091827	1.475992309	0.691050847	3.152522435	0.314638645
BCAT2	0.968406794	1.017800914	0.750407718	1.380474474	0.909661748
BCCIP	0.451621387	0.603017454	0.248199427	1.46507208	0.264094704
BCHE	0.506248863	1.096405106	0.831374638	1.445923535	0.514464581
BCKDHA	0.972877419	1.173794799	0.138360661	9.957991062	0.883219595
BCKDHB	0.394978508	1.48722575	0.459488692	4.813699379	0.507764018
BCKDK	0.595026236	2.307642462	8.59E-05	62023.79164	0.872330706
BCL10	0.904883037	1.016112615	0.264403365	3.904961064	0.981434386
BCL11A	0.54092601	24.41567286	0.003511398	169768.5798	0.479024276
BCL11B	0.22650469	213.5893395	0.172889926	263869.659	0.139737303
BCL2	0.533398931	0.80338107	0.463704288	1.391880904	0.434948402
BCL2A1	0.180425782	1.0394079	0.766047861	1.410314993	0.803944589
BCL2L1	0.589983683	0.426105685	0.079459577	2.285011598	0.319463965
BCL2L10	0.246327809	0.411492448	7.31E-05	2315.426958	0.840274773
BCL2L11	0.20791055	2.345232359	0.854564663	6.4361599	0.097956323
BCL2L12	0.080597147	0.156755281	0.000578182	42.49913903	0.516812397
BCL2L13	0.423015643	0.309981548	0.068640519	1.39988102	0.12784618
BCL2L14	0.386036386	0.742935527	0.542948585	1.01658465	0.063288365
BCL2L2	0.689466594	3.77677856	0.242236698	58.88478672	0.343007559
BCL3	0.595023792	1.088269134	0.463812714	2.553465378	0.845868356
BCL6	0.728110486	0.922535967	0.572136877	1.487533219	0.74081027
BCL6B	0.290604587	0.019838438	0.000293381	1.341474924	0.068253985
BCL7A	0.317281607	1.489365772	0.777348607	2.853559372	0.22984543
BCL7B	0.470746468	0.943168318	0.691212391	1.286965466	0.712141654
BCL7C	0.693171036	1.094677344	0.862891587	1.388724268	0.456162746
BCL9	0.589662189	1.213450612	0.370532983	3.973903687	0.749236398
BCL9L	0.415716547	1.289096929	0.676306392	2.45712729	0.440355346
BCLAF1	0.656788529	1195.627297	0.037596444	38022867.78	0.180339576
BCOR	0.664615231	0.995299269	0.353148807	2.805108263	0.992888745
BCORL1	0.139925724	1.254385898	0.955953417	1.645983951	0.102047918
BCR	0.422340578	1.017085896	0.557276827	1.856283394	0.955986231

BCS1L	0.992703318	3.45305746	0.038667715	308.3607579	0.588702854
BCYRN1	0.855317928	0.98199725	0.753208457	1.280281162	0.893214198
BDH2	0.545698602	7.267541805	0.018326971	2881.936285	0.515842487
BDKRB1	0.445227368	0.860182493	0.638267843	1.159253015	0.322520074
BDKRB2	0.430398194	0.44848448	0.125496945	1.602734863	0.217189758
BDNF	0.275016912	2.630647369	0.655888394	10.55104138	0.172307756
BDP1	0.419184499	53.27334992	0.273295581	10384.54337	0.139471068
BECN1	0.296763559	0.970147476	0.665867163	1.413474305	0.874589884
BET1	0.857685383	0.780033471	0.328570213	1.851817942	0.573333179
BET1L	0.509162834	2.29137991	0.931478177	5.636655824	0.071011148
BEX1	0.372525301	0.029622663	0.000445806	1.96834978	0.100243363
BFAR	0.202853897	0.253383783	0.019658909	3.265864919	0.292542898
BFSP1	0.362406645	1.381839538	0.74470921	2.564061893	0.305171298
BFSP2	0.384553437	0.064755883	6.77E-06	619.7032524	0.558375321
BGLAP	0.918909328	0.873640568	0.165300822	4.617326356	0.873647706
BGN	0.492874968	0.788609862	0.318030426	1.955490621	0.608266207
BHLHB9	0.85310307	2.962761983	0.96016869	9.142100406	0.058856792
BHMT	0.661282346	1.429489703	0.206938094	9.874647887	0.717077883
BHMT2	0.535832335	0.397709224	0.075928688	2.083173454	0.275129997
BICC1	0.101882644	1.270923734	0.789785843	2.045171045	0.323295776
BICD1	0.822714741	1.042257088	0.799598095	1.358557312	0.759548271
BICD2	0.0984261	1.233212646	0.968672134	1.569998121	0.088831475
BID	0.385283591	0.619007972	0.146111419	2.6224567	0.514961044
BIK	0.742682595	1.50198275	0.628572203	3.589010415	0.360048041
BIN1	0.739244143	1.065082147	0.734454183	1.544548328	0.739519769
BIN2	0.498685059	0.468061541	0.138367826	1.583327662	0.222115857
BIN3	0.989630695	1.469005656	0.667901425	3.230982204	0.338909065
BIRC2	0.612674804	4.646743222	0.099989068	215.9458335	0.432862189
BIRC3	0.334040451	2.742685105	0.858164215	8.765596907	0.088766352
BIRC5	0.437920209	1.114894204	0.624660843	1.989862337	0.712898788
BIRC6	0.468388621	6.646314303	0.005968928	7400.574413	0.596684151
BIRC7	0.669604294	1.189424732	0.904044894	1.56489042	0.215237833
BIRC8	0.066915201	2.153744251	0.402141639	11.53477742	0.370230649

BIVM	0.246022075	47.45057616	0.000632467	3559961.403	0.500378399
BLCAP	0.505326818	0.830192157	0.101375343	6.798684966	0.862293929
BLK	0.054600786	1.318369325	0.729118736	2.383833514	0.360406278
BLM	0.261938203	2.811001371	0.688765668	11.47230338	0.149768052
BLMH	0.338787696	0.72929276	0.407251366	1.305994218	0.28827239
BLNK	0.733732564	0.924156189	0.774298472	1.103017367	0.382243621
BLOC1S1	0.843055551	1.421777427	0.437071242	4.624992127	0.558730149
BLOC1S2	0.528576943	1.079768603	0.836322563	1.394079614	0.55602235
BLOC1S3	0.980015026	0.86371817	0.024179225	30.85330788	0.935994445
BLVRA	0.661730624	1.162841978	0.574195498	2.354949612	0.675189686
BLVRB	0.295951675	0.570632265	0.019387517	16.79540451	0.745097738
BLZF1	0.738908572	1.178979361	0.355093659	3.914438622	0.787993681
BMF	0.387242048	0.940763165	0.80696318	1.096748097	0.43531215
BMP1	0.538788096	0.557237547	0.164493045	1.887700988	0.347554084
BMP10	0.691624447	1.165413954	0.827207765	1.641896683	0.381422552
BMP15	0.069969662	2.671239725	1.282452313	5.563966471	0.008678579
BMP2	0.978118969	1.820153441	0.755950402	4.382507818	0.181579187
BMP2K	0.905839469	0.572829794	0.2378957	1.379318638	0.213980027
BMP2KL	0.159055688	0.180913441	0.026551854	1.232669956	0.080757595
BMP3	0.080371252	1.301439779	0.553254537	3.061421793	0.546055523
BMP4	0.53018319	0.354412972	0.040699556	3.086238906	0.347534064
BMP5	0.295765615	0.503135095	0.15873414	1.594773023	0.243207334
BMP6	0.202295733	6070.072135	0.025174357	1463623297	0.168306382
BMP7	0.459961559	0.311570642	0.046846655	2.072213372	0.227714344
BMP8A	0.271234866	1.952168327	0.280769525	13.57327215	0.498966556
BMP8B	0.791085197	0.440434862	0.046736669	4.150549735	0.473715857
BMPER	0.142640919	1.019311929	0.832461427	1.248102045	0.853120426
BMPR1A	0.778312973	1.831667071	0.585380251	5.731324647	0.298391541
BMPR1B	0.482588914	1.293171845	0.58933558	2.837591138	0.521383676
BMPR2	0.521616333	3.770586832	0.611188746	23.2617586	0.152823561
BMX	0.176776263	3.696539457	0.890731287	15.34065789	0.071765936
BNC1	0.391270043	0.719135279	0.384990897	1.343292931	0.301033328
BNC2	0.988838951	0.621349484	0.010921027	35.35154446	0.817477534

BNIP1	0.332928058	0.910504713	0.762757025	1.086871448	0.299346373
BNIP2	0.79690711	1.096893521	0.459894196	2.616200438	0.834816241
BNIP3	0.20732949	1.526385467	1.008985379	2.309104417	0.045250641
BNIP3L	0.760494083	1.123545836	0.32791303	3.849664786	0.85291786
BNIPL	0.391718161	3.992093026	0.960066009	16.59969895	0.056921366
BOC	0.079897532	0.000899668	2.81E-08	28.779732	0.185115331
BOK	0.229745628	3.722885322	0.004800235	2887.332555	0.698596927
BOLA1	0.407168174	3.248494283	0.798781117	13.21102225	0.099748251
BOLA3	0.206701916	2.38246678	0.033798927	167.9387039	0.689272304
BOLL	0.432597499	1.630964849	0.0475912	55.89365957	0.786180083
BOP1	0.59864624	0.475912817	0.118009937	1.919270651	0.296654203
BPESC1	0.681011168	1.061823891	0.812752693	1.387223917	0.660058428
BPGM	0.493671921	1.622639026	0.44697878	5.89056467	0.46182285
BPHL	0.69815627	0.839251844	0.174647552	4.032943196	0.826799148
BPI	0.826362645	1.000249997	0.324299647	3.085109915	0.999652944
BPNT1	0.437627633	0.902619267	0.00643782	126.5524034	0.967595959
BPY2C	0.555562202	1.043963102	0.79467924	1.371445111	0.757271484
BRAF	0.998211672	1.118174099	0.415706893	3.007680016	0.824896521
BRAP	0.267632382	535.7862471	0.001346286	213228762.7	0.339499643
BRCA1	0.560504868	0.974887695	0.524604863	1.811660707	0.935886096
BRCA2	0.251095575	1.148071596	0.633689232	2.079991773	0.648816937
BRD1	0.975931142	0.248644414	0.016604477	3.723335801	0.313500358
BRD2	0.880282119	0.070308743	1.57E-05	315.8231018	0.536103381
BRD3	0.168129296	1.303493493	0.746168651	2.277092833	0.351738013
BRD4	0.78679669	0.976080387	0.430529784	2.212931503	0.953771274
BRD7	0.175668023	0.230397752	0.000187257	283.4768336	0.685940542
BRD8	0.056674417	0.011502767	1.16E-05	11.35979234	0.204363573
BRD9	0.923037863	0.767222157	0.219930947	2.676430242	0.677660366
BRDT	0.418335333	1.403960808	0.558493347	3.529327535	0.470652131
BRE	0.650282432	122.4442628	0.003473539	4316231.41	0.368139621
BRF1	0.228050364	0.907124045	0.554355524	1.484379603	0.698060999
BRF2	0.945906484	1.623686015	0.31975295	8.244978735	0.558786658
BRI3	0.727569608	90.67895131	0.026791894	306908.9529	0.277027747

BRI3BP	0.800528919	0.013564474	9.48E-06	19.40916088	0.246058801
BRIP1	0.295413452	1.520219539	0.82616926	2.797329262	0.178230876
BRMS1	0.109803435	1.501286853	0.887028304	2.540913526	0.130165973
BRMS1L	0.077696561	0.891335161	0.720283304	1.10300817	0.289991952
BRPF1	0.573413828	1.211088707	0.908844068	1.613847641	0.191058811
BRPF3	0.662260248	1.520818677	0.251856011	9.183379974	0.647687207
BRS3	0.354407038	0.435475686	0.061055741	3.105999018	0.406916138
BRSK1	0.529391732	0.770962236	0.119955825	4.9550138	0.784069574
BRSK2	0.400177055	1.218805159	0.882067373	1.684095865	0.230390908
BRWD1	0.198817808	2.62931761	0.324286012	21.31856088	0.365285116
BRWD3	0.925412415	1.496084619	0.518215704	4.31918441	0.456434795
BSCL2	0.256737407	0.947839841	0.560770632	1.602081694	0.841450727
BSDC1	0.320725476	1.031000977	0.752162382	1.413209488	0.849496276
BSG	0.398879658	3.532582811	0.933586499	13.3668828	0.063062729
BSN	0.373128929	0.53865004	0.088016871	3.296457419	0.503252573
BSND	0.980766162	3.298301479	0.000217495	50018.64431	0.808026536
BSPRY	0.807842153	1.006384199	0.757470266	1.337094274	0.964985506
BST1	0.858705804	1.125641516	0.217317023	5.830508834	0.887842124
BST2	0.809221634	1.476871749	0.799995154	2.726454219	0.212555582
BTAF1	0.018128901	2.161298445	1.160348572	4.025696316	0.015157217
BTBD1	0.522105062	2.388243254	0.916227398	6.225207688	0.074916268
BTBD10	0.784741541	1.277837353	0.340029853	4.802132185	0.716633009
BTBD11	0.61031196	0.987828707	0.00051332	1900.970671	0.997467655
BTBD2	0.161271515	0.607590064	0.117725413	3.135819836	0.551810886
BTBD3	0.998875732	2.500739871	0.328390883	19.04346381	0.376208363
BTBD6	0.128911453	0.535180142	0.122131745	2.345154282	0.406941614
BTBD7	0.865084108	0.018610426	7.45E-05	4.647917345	0.157221436
BTBD8	0.508911097	0.417739331	0.096558128	1.807265243	0.242789187
BTBD9	0.77830802	0.759890966	0.285432029	2.023018517	0.582583151
BTC	0.805528734	0.081404672	0.001587779	4.173579671	0.211777672
BTD	0.772964365	0.990509114	0.3455631	2.839158186	0.985838971
BTF3	0.818103395	0.026194916	0.0001975	3.474289381	0.144138624
BTF3L4	0.576507781	0.844218528	0.455885656	1.56334141	0.590119065

BTG1	0.128046377	0.154745068	0.018134232	1.320487991	0.088041046
BTG2	0.30169062	0.900370535	0.031941772	25.37952786	0.95087653
BTG3	0.709953059	0.930992168	0.486757094	1.780654926	0.82889979
BTG4	0.987435557	0.806379812	0.181017366	3.592187965	0.777693151
BTK	0.125245516	0.009616374	8.25E-06	11.21412508	0.197377777
BTLA	0.125519277	1.193118891	0.780121656	1.824757303	0.415344102
BTN1A1	0.660032031	1.1335461	0.535952211	2.397465174	0.742920628
BTN2A1	0.573811683	1.432836598	0.602163063	3.409409915	0.416127417
BTN2A2	0.810200484	0.546155792	0.13313203	2.240528801	0.400998422
BTN3A1	0.886562478	0.345898027	0.043651106	2.740948753	0.314791043
BTN3A2	0.338304777	3.836560009	0.957587152	15.37112593	0.057595666
BTN3A3	0.553007288	0.487445815	0.005948087	39.94618851	0.749238744
BTNL2	0.3758762	0.57204162	0.19238226	1.700944853	0.315096114
BTNL3	0.377396494	2.238977795	0.883593353	5.673448709	0.089303654
BTNL8	0.769851987	0.025774881	1.10E-06	601.7572775	0.475923234
BTNL9	0.61382518	1.272283961	0.886154095	1.826664783	0.19189641
BTRC	0.600141561	0.732259812	0.411374332	1.3034465	0.289512368
BUB1	0.645646676	0.681306582	0.026177529	17.73195104	0.817489395
BUB1B	0.429697466	0.144057383	0.019660682	1.055534591	0.056549742
BUB3	0.606705412	0.818302808	0.492124443	1.360671056	0.439584788
BVES	0.111740645	1.335934398	0.844312603	2.113815084	0.216044857
BYSL	0.900648759	0.008116715	9.52E-08	691.9368818	0.405957693
BZRAP1	0.730375511	0.466277511	0.088374888	2.460141368	0.368591321
BZW1	0.120006192	2.604482234	0.767907341	8.83352372	0.124498968
BZW2	0.110263687	1.993583163	0.986456231	4.028940869	0.054608688
C10orf10	0.42501924	0.516117453	0.099235006	2.684307049	0.431735976
C10orf107	0.53328916	0.983604445	0.741981713	1.303910443	0.908493949
C10orf11	0.26551027	0.015457375	4.50E-05	5.309594778	0.161639373
C10orf111	0.612735573	1.077383423	0.944432811	1.229049887	0.267349768
C10orf113	0.774500645	1.931598359	0.086736436	43.01620392	0.67755273
C10orf12	0.407152291	0.236651793	0.010399529	5.385250632	0.3660316
C10orf120	0.461819286	0.162812274	3.91E-07	67861.68782	0.783373383
C10orf128	0.484501224	0.002888815	1.25E-07	66.73723676	0.2540631

C10orf32	0.853870091	0.358333471	0.047986018	2.675839399	0.317084421
C10orf35	0.207394486	0.508457874	0.143033912	1.807469333	0.29591643
C10orf53	0.335855911	44.46879843	0.003816434	518147.0741	0.426993673
C10orf55	0.846755687	0.830997866	0.584977819	1.180484851	0.301329026
C10orf62	0.086310651	0.244603729	0.012928409	4.627869133	0.347905938
C10orf67	0.85192241	1.040687918	0.810006472	1.337065047	0.755096463
C10orf71	0.148208099	0.24001133	0.016780888	3.432800357	0.293107405
C10orf76	0.829898079	1.266250853	0.372711496	4.301963429	0.705204776
C10orf82	0.640975534	0.606553073	0.17171284	2.142569137	0.437458086
C10orf88	0.126149033	1.427032429	0.434208951	4.68995756	0.558034161
C10orf90	0.703304914	39.19922305	0.002883691	532851.5299	0.449943727
C10orf91	0.067990066	1.16523076	0.982560903	1.381861134	0.07879167
C10orf95	0.811999857	5.456884277	0.013222183	2252.093053	0.580804034
C10orf99	0.7903584	2.680196261	0.14694945	48.88383052	0.505732508
C11orf1	0.927817228	0.37624562	0.000216071	655.1592018	0.797379892
C11orf16	0.902898753	2.01998113	0.800813749	5.095221917	0.136378601
C11orf24	0.813118214	0.920668185	0.62636278	1.353257144	0.674048181
C11orf30	0.300088498	0.000396741	1.00E-08	15.7402116	0.147120764
C11orf31	0.522719076	0.520254099	0.105225981	2.572219567	0.422932111
C11orf39	0.250573397	2.04067144	0.325999806	12.77405646	0.445933676
C11orf40	0.793599158	0.691954046	0.34991877	1.368318713	0.289811585
C11orf42	0.527983225	1.525825397	0.832804366	2.795546273	0.17139414
C11orf44	0.981045608	0.562824267	0.071317043	4.441731471	0.585525497
C11orf45	0.651425836	0.256239341	0.005533645	11.86534468	0.486522353
C11orf49	0.311416176	0.161617517	2.06E-05	1269.749886	0.690434763
C11orf52	0.183912332	0.098244142	0.007232359	1.33454533	0.081306305
C11orf53	0.31047073	0.715673086	0.327690816	1.563022037	0.401266074
C11orf54	0.722455268	0.045080503	4.30E-07	4725.139939	0.599249533
C12orf10	0.168233381	0.186406493	0.009158211	3.794122981	0.274556472
C12orf29	0.301914896	1.066506259	0.639402064	1.778905113	0.80516425
C12orf4	0.136354103	0.830282222	0.088442361	7.79455184	0.870691296
C12orf40	0.129907157	0.280569301	0.021455994	3.668864481	0.332571492
C12orf42	0.278182816	7.332407602	0.762998134	70.46439417	0.084407465

C12orf43	0.268605189	0.876049632	0.311563048	2.463266948	0.80190541
C12orf45	0.175407329	1.89070486	0.783707423	4.561351294	0.156320253
C12orf49	0.355776102	1.39973496	0.186789695	10.48911163	0.743477703
C12orf5	0.151372026	1.50213634	0.929987628	2.426283445	0.096260682
C12orf50	0.457178244	0.228792775	0.000182187	287.3208978	0.685381348
C12orf54	0.196159907	0.106175261	0.010858164	1.038222129	0.053889738
C12orf57	0.652452293	0.674459307	0.322095533	1.412299489	0.296273851
C12orf60	0.661074391	0.234041459	8.25E-06	6641.875057	0.781317504
C14orf1	0.350252462	1046.238531	0.023292608	46994097.03	0.203335043
C14orf105	0.284005333	1.299525303	0.475657638	3.550381363	0.609403248
C14orf119	0.079652465	2.352068997	0.866626784	6.383634423	0.093159147
C14orf132	0.539777223	3.751807002	0.06260036	224.8558272	0.526649309
C14orf142	0.422511503	0.036191819	9.78E-05	13.39231435	0.271331793
C14orf159	0.067208526	0.013221485	9.66E-06	18.0960404	0.240369104
C14orf166	0.49127014	1.103911793	0.838152151	1.453937982	0.481727915
C14orf169	0.868385169	2.095773619	0.719597545	6.103782722	0.174897749
C14orf2	0.921022945	3.423956643	1.55E-05	754503.244	0.844551265
C14orf28	0.350993609	0.68594136	0.190972971	2.463780861	0.563386333
C14orf37	0.105970847	2.600631383	0.777874999	8.694563522	0.120648881
C14orf39	0.832772725	1.575846671	0.127178901	19.52598038	0.723226971
C14orf79	0.772487829	0.124552015	0.000997725	15.54858424	0.397665528
C14orf80	0.635430818	0.585440956	0.201997189	1.696761798	0.324072698
C14orf93	0.999148879	0.276717391	0.020339236	3.764768449	0.3347365
C15orf26	0.130060652	0.668081122	0.410386748	1.087589664	0.104747639
C15orf27	0.635657233	1.156742	0.260822819	5.130118832	0.84805921
C15orf32	0.355997139	7.23137329	0.610526537	85.65190286	0.116714196
C15orf39	0.763862826	0.401536846	0.084818048	1.900914279	0.250045535
C15orf40	0.396535939	16.43946506	0.277637918	973.411749	0.178769929
C15orf41	0.165709998	1.347611306	0.857865689	2.116947039	0.195436658
C15orf43	0.223096077	0.008195097	3.93E-06	17.08218985	0.217908137
C15orf48	0.147885819	0.551006776	0.144989802	2.093998773	0.381591709
C16orf45	0.076537795	1.441834582	0.98983433	2.10023728	0.056556662
C16orf46	0.942884039	2.929271252	0.469294891	18.28409009	0.250029358

C16orf47	0.505635637	2.046593871	0.641129259	6.533076464	0.226532692
C16orf52	0.831837935	1.292900015	0.560969792	2.979822573	0.546508488
C16orf54	0.79823626	1.191215725	0.849445197	1.670496117	0.310493541
C16orf58	0.768023952	0.862304985	0.284203102	2.616332762	0.793625474
C17orf47	0.888565215	0.056506506	5.87E-07	5442.58581	0.623590793
C17orf49	0.157484867	2.573399064	0.83383784	7.942051114	0.100191557
C17orf50	0.839858519	771.4446676	0.014835231	40115781.04	0.230155744
C17orf51	0.20294654	0.000741308	4.73E-08	11.61538146	0.14364005
C17orf53	0.431025009	2.257121074	0.685763576	7.429084485	0.180455948
C17orf58	0.385357579	8.231877934	0.713125526	95.02368354	0.091207781
C17orf59	0.81845416	3.480718906	0.003924118	3087.420816	0.718746182
C17orf62	0.967982179	0.989707122	0.684077336	1.431885164	0.956214791
C17orf64	0.607728533	1.211870631	0.472926523	3.105409312	0.688965067
C17orf67	0.221549384	0.703419611	0.238542852	2.074256868	0.523725048
C17orf70	0.254216714	0.829394435	0.496531055	1.385402024	0.474850974
C17orf74	0.145511216	0.478272473	0.185335383	1.234219578	0.127285627
C17orf75	0.883716944	0.966628275	0.34954941	2.673070522	0.947854895
C17orf77	0.154290333	1.408392116	0.000445236	4455.092783	0.933628498
C17orf78	0.529513331	1.19221583	0.935923725	1.518690623	0.154529442
C17orf80	0.294517506	1.679660629	0.788603232	3.577540285	0.178843772
C18orf21	0.693095203	3.803675139	0.052525393	275.446671	0.540908031
C18orf25	0.833507221	1.846845245	0.455367207	7.490300802	0.390464707
C18orf54	0.582841081	1.113511539	0.659361566	1.880467427	0.687566336
C18orf8	0.090149751	0.715848001	0.311924852	1.642826332	0.430277629
C19orf12	0.426189547	0.033345783	3.88E-06	286.4872888	0.461836641
C19orf18	0.744810864	566.7881321	0.028236004	11377275.22	0.209747142
C19orf24	0.525894871	1.057782695	0.210361538	5.318958212	0.945650663
C19orf25	0.290238814	2.188198928	0.493427319	9.703991574	0.302800789
C19orf26	0.618947725	0.820114121	0.60956834	1.103382718	0.190176626
C19orf33	0.610327708	1.958238705	0.496447581	7.72427739	0.337145088
C19orf35	0.215565567	0.192663956	0.00015299	242.6256068	0.651151922
C1D	0.665650937	1.161325844	0.71657013	1.882129411	0.543779464
C1GALT1	0.421333471	1.369201796	9.61E-05	19511.2065	0.948658341

C1GALT1C1	0.867424549	1.181419541	0.473726223	2.946326517	0.720668308
C1orf100	0.489003296	0.528665537	0.158117313	1.767594231	0.300662996
C1orf101	0.511791967	0.485276712	2.50E-05	9425.059477	0.885880747
C1orf105	0.769651539	1.020284293	0.814236844	1.278473268	0.86149342
C1orf106	0.441690196	0.000596621	6.39E-09	55.72877862	0.203574036
C1orf109	0.105154381	1.672767424	0.527402899	5.305528016	0.382340423
C1orf110	0.307774227	1.624635212	0.895398631	2.947781559	0.110380319
C1orf111	0.145585653	0.031769204	7.40E-06	136.3813252	0.4189713
C1orf112	0.073723837	0.767522879	0.220730999	2.668820291	0.677320764
C1orf115	0.413023904	1.965197964	0.782229188	4.937175826	0.150603031
C1orf116	0.112558448	0.0002952	1.40E-10	622.3185512	0.273947934
C1orf122	0.894464008	0.949904081	0.790068696	1.142075072	0.584561373
C1orf123	0.473694046	0.973799479	0.755529522	1.255126899	0.837539659
C1orf127	0.145994871	0.568129827	0.110705406	2.91558933	0.498035177
C1orf131	0.924597207	2.967873171	0.013096516	672.5659857	0.694210655
C1orf132	0.364756901	0.75639418	0.306552902	1.866340694	0.544599236
C1orf137	0.818897098	2.113926636	0.509898698	8.763869842	0.30222755
C1orf140	0.303172624	3.132252858	0.294217453	33.34611145	0.344078435
C1orf141	0.892638055	1.220461153	0.272726809	5.461602513	0.794417137
C1orf146	0.160088098	1.616893369	0.781335305	3.345995184	0.195331045
C1orf147	0.569402535	0.776685083	0.266390571	2.264493508	0.643444996
C1orf158	0.534216374	2.001966445	0.296467587	13.5187448	0.476274428
C1orf159	0.626704102	17.60698606	0.01459869	21235.19017	0.428161665
C1orf162	0.540225265	0.371424769	0.052353651	2.635085705	0.321816404
C1orf167	0.732087808	1.040434865	0.614443611	1.761764123	0.88272882
C1orf168	0.894137538	1.315338559	0.422935347	4.090732873	0.635877107
C1orf174	0.678844464	0.22808748	0.013738967	3.786594689	0.302492357
C1orf177	0.542851047	3.404883626	0.510932997	22.69031863	0.205491971
C1orf185	0.967367315	0.924998685	0.809353469	1.057167973	0.252574326
C1orf186	0.522188486	1.502888825	0.633807636	3.563659841	0.355071929
C1orf189	0.138475119	0.400684952	0.059911671	2.679752162	0.345531078
C1orf198	0.475891689	0.936310404	0.216512887	4.049076185	0.929809575
C1orf21	0.380949529	0.510731909	0.012156766	21.45694691	0.724605788

C1orf27	0.791800647	1.095114352	0.69010091	1.737826202	0.699762395
C1orf35	0.175787764	0.903277616	0.568099749	1.436209844	0.667238254
C1orf43	0.794772619	1.567276349	0.585906961	4.192397964	0.370749981
C1orf50	0.752601837	1.244424983	0.457122524	3.387699043	0.668680209
C1orf52	0.590168769	0.899814018	0.438532161	1.846307609	0.773446365
C1orf53	0.130724554	26476.8071	0.036718417	19091817488	0.138925898
C1orf54	0.638495798	0.813766163	0.036736737	18.02597114	0.896263214
C1orf56	0.744115501	1.509182344	0.663053584	3.435063773	0.32670195
C1orf61	0.488788732	5.934448066	0.005638614	6245.803244	0.615981958
C1orf64	0.920767418	0.025944325	4.64E-05	14.49259236	0.25783368
C1orf74	0.820784082	1.723681087	0.334977644	8.869476939	0.514775328
C1orf86	0.210420879	1.075098577	0.839739284	1.376423577	0.565683298
C1orf87	0.384447206	0.613080688	0.028433632	13.21913181	0.754842247
C1orf94	0.906669799	0.566410253	0.007608431	42.1664587	0.796027427
C1orf95	0.666190771	1.318300224	0.255813997	6.793668446	0.741151626
C1QA	0.227424845	1.232029406	0.682430483	2.224250666	0.488759438
C1QB	0.184342056	0.80317151	0.094746335	6.808542813	0.840702877
C1QBP	0.928719339	0.704847324	0.275920443	1.800554339	0.464803522
C1QC	0.918736226	0.143104514	0.002063871	9.922567646	0.368694219
C1QL1	0.62709018	0.500223496	0.204979708	1.220723498	0.128058394
C1QL2	0.177624443	1.923572689	0.825852059	4.480381024	0.129410721
C1QL3	0.342247833	0.873780712	0.335225987	2.277546377	0.782520003
C1QL4	0.291953451	0.347109562	0.100725686	1.19617005	0.0936981
C1QTNF1	0.998812031	0.808278451	0.300212462	2.176172332	0.673599892
C1QTNF2	0.195134915	0.682182249	0.190023093	2.449031926	0.557555832
C1QTNF3	0.835574588	1.269521392	0.751588523	2.144370911	0.372256165
C1QTNF4	0.329854457	1.967581302	0.574029163	6.744215149	0.281560565
C1QTNF6	0.47486339	0.042664714	0.000109085	16.68684166	0.300312397
C1QTNF7	0.227045606	0.843824735	0.237570228	2.997177678	0.792870002
C1QTNF8	0.550461226	0.544339488	0.118613131	2.498083268	0.434030571
C1R	0.62633638	0.000962873	1.15E-07	8.045857058	0.1317037
C1RL	0.884823818	1.064132973	0.123111944	9.197962048	0.954953969
C1S	0.982797145	12.61496233	0.002852581	55787.11065	0.553947343

C2	0.771224078	0.250149881	0.013276209	4.713315523	0.354960201
C20orf141	0.26720663	0.020690516	5.77E-06	74.17364861	0.353046983
C20orf144	0.219757836	0.005516297	5.66E-06	5.375480531	0.13861358
C20orf173	0.369692329	0.881484749	0.665002809	1.168439217	0.380309929
C20orf194	0.931061904	1.117371979	0.292230577	4.272380236	0.871163329
C20orf195	0.956906617	1.623424048	0.352989997	7.466233209	0.533685281
C20orf24	0.789325722	6.283075839	0.036629153	1077.749259	0.483830276
C20orf27	0.833888897	1.214584538	0.645511799	2.285342579	0.546659822
C20orf62	0.290159636	0.304624451	0.06713368	1.382257851	0.123451637
C20orf85	0.916556509	1.349640247	0.699754895	2.603095468	0.370966776
C20orf96	0.842392433	1.41330349	0.275370918	7.253586428	0.678477569
C21orf2	0.532243463	1.55358277	0.718356907	3.359916777	0.262949034
C21orf33	0.663135866	0.439473707	0.103395696	1.867941762	0.265438172
C21orf58	0.941474042	0.692260112	0.259083623	1.849688747	0.463271987
C21orf59	0.259480938	1.094835278	0.931510885	1.28679579	0.271672184
C21orf62	0.417910458	0.776550496	0.498622406	1.209393453	0.263206297
C21orf91	0.525674831	1.219765276	0.438781384	3.390816888	0.703330796
C22orf15	0.47107243	0.952132725	0.236794839	3.828447988	0.944918882
C22orf23	0.355845632	0.855385733	0.690606081	1.05948206	0.152507621
C2orf15	0.262552439	1.52E-05	7.85E-12	29.51315504	0.133166499
C2orf16	0.479076867	1.021449963	0.862592241	1.209563427	0.805619632
C3	0.961798223	0.365100017	0.119900217	1.111741292	0.076143563
C3AR1	0.951859921	0.026979791	1.87E-07	3889.342385	0.551118163
C3orf14	0.020953361	0.277249513	0.131871185	0.582896803	0.000715463
C3orf17	0.75097094	1.189441625	0.548097876	2.581238574	0.660762953
C3orf18	0.833875703	1.579938221	0.435609095	5.730378021	0.486557092
C3orf20	0.132558281	0.71586285	0.463751522	1.105030594	0.131278861
C3orf22	0.231437655	1.393318752	0.552058602	3.516541788	0.482549013
C3orf30	0.054561818	0.011133567	0.000113447	1.092640147	0.054592843
C3orf33	0.935828408	0.966221412	0.871069001	1.071767928	0.515928264
C3orf35	0.055793796	2.954849825	0.863407834	10.11241403	0.084348379
C3orf36	0.266217449	0.900677124	0.74526891	1.088492049	0.27902715
C3orf38	0.681715287	0.836735195	0.166915423	4.194494273	0.828425654

C3orf52	0.652953917	1.046549595	0.423495662	2.586250942	0.921481102
C3orf56	0.352615083	0.102294591	5.37E-05	194.7810894	0.554039632
C3orf58	0.68337507	1.034150434	0.65034838	1.644452656	0.88716072
C3orf62	0.883509994	0.984819165	0.682171867	1.421736713	0.934920507
C4B	0.057901957	0.407242953	0.149402658	1.110066077	0.079110653
C4BPA	0.72354247	0.544823791	7.14E-05	4156.620685	0.894079677
C4BPB	0.446335519	0.900585031	0.259461014	3.125916236	0.869009195
C4orf17	0.642986821	1.019549295	0.268226715	3.875381181	0.977328698
C5	0.054272494	0.563843693	0.206356618	1.54063249	0.263892094
C5AR1	0.09401345	1.304588339	0.745598294	2.282664469	0.351597863
C5orf15	0.664702456	19.75961697	0.062600172	6237.082934	0.309536224
C6	0.80964485	2.68396197	0.489380896	14.71992863	0.255540462
C6orf1	0.170159874	0.542820433	0.235779648	1.249700836	0.150988907
C6orf10	0.820264218	1.198881658	0.985721489	1.458137258	0.069372855
C6orf106	0.642594091	0.168761495	0.001847853	15.41271978	0.439833958
C6orf118	0.179419303	0.028207828	7.36E-05	10.81675202	0.239787259
C6orf120	0.491031154	1.475942997	0.504703902	4.316209404	0.47705646
C6orf136	0.993006229	56.27061321	0.00648422	488321.1443	0.383737268
C6orf141	0.1466198	0.633371374	0.254887552	1.573867752	0.325417246
C6orf15	0.82035064	0.710722822	0.326512111	1.547038876	0.389538904
C6orf163	0.687660017	1.009601896	0.638389854	1.596666963	0.967405917
C6orf165	0.569438568	0.538741561	0.21108954	1.374973248	0.195717913
C6orf195	0.99727331	1.013693857	0.483479521	2.125374893	0.971277127
C6orf201	0.343807915	0.045456945	0.000131467	15.71748324	0.300041155
C6orf203	0.471856634	3.241308633	0.200496953	52.40020603	0.407547593
C6orf211	0.246179426	1.456417799	0.587805239	3.60859799	0.416698464
C6orf25	0.62054018	0.041189177	3.72E-05	45.63297859	0.372518818
C6orf47	0.4862734	0.348240281	0.003095681	39.17434872	0.661559866
C6orf48	0.979144863	1.04540326	0.48030658	2.275354997	0.910903369
C6orf52	0.857354987	1.422771146	0.529790088	3.820905263	0.484193248
C6orf57	0.436586464	1.53093581	0.202169643	11.59305831	0.680121264
C6orf62	0.894828356	0.698768433	0.225720245	2.163196851	0.534147247
C6orf89	0.69441448	0.439695378	1.13E-05	17178.17191	0.878937534

C7	0.425966809	1.457465577	0.050949062	41.69273819	0.825750777
C7orf25	0.3777315	1.278301705	0.84421104	1.935600427	0.246080339
C7orf26	0.986572051	1.008426842	0.481899497	2.110242286	0.982229677
C7orf31	0.050212266	0.119672782	0.006162239	2.324086055	0.160692887
C7orf33	0.455671555	0.712785491	0.237385885	2.14024164	0.546145869
C7orf34	0.334023991	0.824891591	0.279280464	2.436425828	0.727560604
C8A	0.253033984	0.120749044	0.003207416	4.5458183	0.253455846
C8B	0.350985272	1.746057518	0.665027804	4.58434495	0.257764413
C8G	0.090360905	1.395743458	0.974886891	1.998282898	0.068598648
C8orf17	0.416597054	1.089714364	0.770615676	1.540946325	0.626963728
C8orf22	0.865005329	1.307178429	0.542621814	3.148998809	0.550411043
C8orf31	0.553781052	0.60710314	0.164331391	2.242871677	0.4541665
C8orf33	0.169535143	1.377619375	0.572003721	3.31787202	0.475012076
C8orf34	0.529243736	0.438582187	0.113516537	1.694504956	0.232012555
C8orf37	0.953689429	0.915668291	0.662441926	1.265693469	0.593752294
C8orf4	0.992243444	1.060352287	0.55608962	2.021880883	0.858759197
C8orf44	0.957954041	0.990938656	0.811444884	1.210136929	0.928861419
C8orf46	0.961290432	0.562728822	0.090426518	3.501890094	0.537646353
C8orf48	0.792846621	11591.47058	0.240629612	558377619.7	0.088937362
C8orf49	0.274509746	4.824369428	0.019880175	1170.741228	0.574362452
C8orf58	0.477760247	0.553156595	0.165019859	1.854214519	0.337334026
C8orf59	0.904067803	1.06842978	0.906107952	1.259830236	0.431129585
C8orf76	0.721828194	1.178032118	0.538394689	2.577587965	0.68171469
C9	0.725274528	0.841994898	0.355677661	1.993252559	0.695682891
C9orf106	0.535369812	0.468084166	0.108453864	2.020239568	0.308945296
C9orf114	0.798654786	0.935573343	0.51431019	1.701886329	0.827313667
C9orf116	0.226491417	1.494002577	0.594134426	3.756799139	0.393487166
C9orf117	0.448947032	0.992989852	0.408573507	2.413345037	0.987612381
C9orf142	0.746980264	6.200924202	0.095215066	403.8379889	0.391810128
C9orf152	0.708929668	1.399073934	0.491521922	3.982340938	0.529220589
C9orf153	0.413464849	1.052924403	0.454102562	2.441408374	0.904334584
C9orf156	0.427201995	1.016185084	0.53358421	1.935274893	0.961039659
C9orf16	0.747492783	0.927572123	0.046318369	18.57556849	0.960785069

C9orf24	0.231020944	0.136453553	0.015218457	1.223486232	0.075119745
C9orf3	0.335606519	3.102866427	0.570644204	16.87177403	0.189981108
C9orf40	0.907044011	1.53453918	0.433880637	5.427323316	0.506416626
C9orf41	0.266043822	1.414323537	0.832614057	2.402446907	0.199727546
C9orf43	0.58139791	0.029520483	4.63E-06	188.0653117	0.430572946
C9orf47	0.173000611	1.691004002	0.781962177	3.656819495	0.181890854
C9orf50	0.204922281	3.085753319	0.000777677	12243.99129	0.789829847
C9orf57	0.406600174	6.309637058	0.001595662	24949.85204	0.662905302
C9orf62	0.077649674	24.98055977	0.140027364	4456.474417	0.223720981
C9orf64	0.624438141	0.689030489	0.084926618	5.59027342	0.727305731
C9orf66	0.055624473	79532.87706	0.120611293	52445159939	0.098827595
C9orf72	0.746127916	0.913141906	0.688676712	1.210768603	0.52787176
C9orf78	0.279769677	1.042546886	0.624603911	1.740149222	0.873347839
C9orf84	0.816997655	1.117518916	0.738224487	1.691692093	0.599417372
C9orf85	0.392590753	0.542860512	0.170906874	1.724316457	0.30019906
C9orf89	0.566981252	0.995024407	0.217836285	4.545035141	0.994864908
C9orf9	0.509066701	1.474066013	0.840717809	2.584542145	0.175616538
C9orf91	0.843750537	1.123105233	0.554664965	2.274103187	0.747045299
CA1	0.622187076	2.542888945	0.018984757	340.603994	0.708768789
CA10	0.436868632	0.37331726	0.063694477	2.188035499	0.274786482
CA11	0.162632092	0.875426798	0.271168052	2.826188681	0.823926667
CA12	0.999296811	0.571157217	0.12998623	2.5096548	0.458323691
CA13	0.364901611	0.847317099	0.186687516	3.845711166	0.830019873
CA14	0.585396042	0.532544856	0.020814881	13.62506127	0.703260821
CA2	0.809002889	0.926777625	0.760156704	1.129920662	0.452048966
CA3	0.546847649	1.146459447	0.715727261	1.836410788	0.56963089
CA4	0.904684282	0.622825051	0.214499163	1.808450156	0.383973637
CA5A	0.561144953	0.93030038	0.731295341	1.183460018	0.556317003
CA5B	0.181534104	235.72698	0.20760711	267655.6166	0.128019539
CA6	0.303392929	1.059576287	0.90678644	1.238110605	0.466381396
CA7	0.126991556	2.116240268	0.769080751	5.823150383	0.146624012
CA8	0.551080009	0.005076171	1.10E-06	23.34725622	0.219521674
CA9	0.400346691	0.058650351	0.000505816	6.800622217	0.242207689

CAB39	0.882045755	0.927103443	0.695325987	1.23614076	0.60608556
CAB39L	0.152630481	0.285256222	0.06512164	1.24952492	0.096035437
CABIN1	0.303213036	1.147357033	0.844555754	1.558722625	0.379245178
CABLES1	0.341432766	2.905430512	0.001513908	5575.985064	0.782141683
CABLES2	0.837674423	0.159235798	0.000231249	109.6481631	0.581572044
CABP1	0.216858982	0.112391923	0.003721313	3.394485475	0.208725214
CABP2	0.248959428	0.925078469	0.708716233	1.2074934	0.566708302
CABP4	0.075497399	1.511890824	0.805978624	2.836072568	0.197776593
CABP5	0.346781922	1.66843769	0.676217615	4.116551043	0.266613066
CABP7	0.350083343	0.87233314	0.534998308	1.422369186	0.5840037
CABYR	0.736475762	1.446335375	0.004537773	460.9939875	0.900146227
CACHD1	0.206622349	0.479875371	0.183459417	1.255211508	0.134488732
CACNA1A	0.726451164	0.737534679	0.316317464	1.719656564	0.480908661
CACNA1B	0.202731251	0.305353135	0.079404905	1.174241524	0.084305484
CACNA1C	0.822213222	0.1144862	0.012542305	1.045030443	0.054741156
CACNA1D	0.304314071	10.62977896	0.355530706	317.8127763	0.172745116
CACNA1E	0.660435	1.108884887	0.458741516	2.680432557	0.81847165
CACNA1F	0.153100358	3920.598976	0.019951166	770435974.5	0.183353975
CACNA1G	0.974722405	1.176847796	0.794751843	1.742645515	0.416211735
CACNA1H	0.077708515	1.460652218	0.88569082	2.408859674	0.137705085
CACNA1I	0.608944781	1.030719118	0.46922918	2.264100244	0.939928527
CACNA1S	0.599936382	1.0569774	0.042420753	26.33619503	0.973055687
CACNA2D1	0.752900003	0.99975193	0.798648449	1.251494224	0.99827244
CACNA2D2	0.973281536	1.016076068	0.01614286	63.95462618	0.993979086
CACNA2D3	0.597441869	0.664036105	0.038070853	11.58219245	0.778952238
CACNA2D4	0.232971074	0.660264577	0.21617136	2.016683944	0.466203941
CACNB1	0.574416108	1.053187639	0.442169899	2.508547521	0.906836452
CACNB2	0.413379066	1.03136052	0.858255289	1.239380097	0.7418538
CACNB3	0.126249544	0.593011734	0.324494237	1.083726234	0.089395531
CACNB4	0.47046032	0.853847221	0.407891644	1.787374384	0.67507404
CACNG1	0.301052169	1.113094956	0.663707772	1.866755875	0.684637869
CACNG2	0.92425033	0.755375366	0.302245856	1.887840419	0.54831357
CACNG3	0.325081557	0.756288245	0.326426603	1.752222106	0.514662062

CACNG4	0.848589006	0.180473363	1.72E-06	18942.52413	0.771617204
CACNG5	0.855241131	0.726560075	0.276367092	1.910102751	0.517166782
CACNG6	0.051201425	0.011357587	9.20E-05	1.40263292	0.068413758
CACNG7	0.637784926	1.190920472	0.53254096	2.663253493	0.670466858
CACNG8	0.061204742	0.006174174	1.48E-05	2.581098518	0.098525144
CACYBP	0.497637816	1.047311372	0.793265989	1.382715415	0.744338413
CAD	0.599983237	0.844611237	0.403023783	1.77003981	0.654611909
CADPS	0.518529962	0.621246185	0.253820396	1.520550859	0.297254927
CADPS2	0.688827929	1.460418686	0.721517564	2.956023312	0.292477125
CAGE1	0.088180497	1.096128963	0.864445044	1.389907562	0.448687795
CALB1	0.712297703	1.691328359	0.686824769	4.164951157	0.253071564
CALB2	0.681570997	0.971383346	0.310751727	3.036461338	0.96017872
CALCA	0.71215013	0.762421121	0.464639379	1.251047572	0.283033264
CALCB	0.868480549	1.118595212	0.895361647	1.397485867	0.3237464
CALCOCO1	0.446585625	1.700325177	0.546738651	5.287911698	0.359163257
CALCOCO2	0.545843671	0.021684714	5.16E-05	9.111920877	0.213850596
CALCR	0.454596941	1.979529248	0.839530071	4.667535062	0.118689706
CALCRL	0.319572726	0.666477925	0.256854046	1.729358875	0.404261005
CALD1	0.870756949	1.083441605	0.459535137	2.554419927	0.85468751
CALM1	0.235471827	1.395217663	0.60599265	3.212303529	0.433772527
CALM2	0.333813989	6837.477199	0.464528838	100641963.8	0.071329287
CALM3	0.124363304	1.832516912	0.016091413	208.6900773	0.802041692
CALML3	0.910890506	0.103428543	0.003555277	3.008897659	0.187041101
CALML4	0.24808457	0.10754012	0.00050425	22.93480493	0.415069013
CALML5	0.353426411	0.782698377	0.213064719	2.875261329	0.712080909
CALML6	0.172798379	1.819550884	0.953014003	3.473994516	0.069660456
CALN1	0.8920213	0.716058853	0.379116688	1.352460331	0.303291917
CALR	0.243636503	0.755958173	0.001766752	323.459563	0.927888281
CALR3	0.251768624	0.241669389	0.012176219	4.796570647	0.351576162
CALU	0.796079141	0.618228284	0.200229563	1.908840059	0.403134438
CAMK1	0.173051499	0.299184382	0.000246732	362.7875678	0.739069598
CAMK1D	0.425928981	0.661803188	0.32010525	1.368248287	0.265321052
CAMK1G	0.472066325	1.736400917	0.290683534	10.37240778	0.545104966

CAMK2A	0.939320221	0.407254323	0.000286915	578.0664918	0.808328363
CAMK2B	0.577727468	147.1571197	0.001896775	11416861.24	0.384895561
CAMK2D	0.741626244	0.675109954	0.008489629	53.68591095	0.860321098
CAMK2G	0.651378457	0.665942297	0.165989727	2.671726448	0.566268516
CAMK2N1	0.461287139	0.115559099	0.0042781	3.121457084	0.199446233
CAMK2N2	0.91627377	1.355260843	0.684288025	2.684150365	0.383273625
CAMK4	0.898540889	1.224795886	0.371981984	4.032789292	0.738754669
CAMKK1	0.691997452	0.930685288	0.466813704	1.855504882	0.838314891
CAMKK2	0.584906975	1.138634789	0.724445891	1.789628733	0.573606899
CAMKV	0.257970816	3.500641707	0.222142418	55.16502637	0.373142938
CAMLG	0.312771348	0.562338008	0.094884527	3.332724998	0.526047446
CAMP	0.376401737	67.689443	0.004788575	956831.6992	0.387338666
CAMSAP1	0.642971574	0.528116725	0.193183634	1.443741741	0.213406517
CAMTA1	0.593948119	0.008193737	4.21E-05	1.59429439	0.074015011
CAMTA2	0.795272589	0.757709618	0.223817275	2.565145452	0.655645706
CAND1	0.903159927	0.29671524	0.029958324	2.938746963	0.299021737
CAND2	0.398383922	0.958362793	0.353570867	2.597666632	0.93337929
CANT1	0.985622683	0.721826122	0.320807894	1.624127584	0.430791001
CANX	0.352064848	0.418436011	0.159177725	1.099957262	0.077267773
CAP1	0.057918345	0.094214133	0.002734538	3.245997231	0.19087419
CAP2	0.630431264	2.118902125	0.349228517	12.85618439	0.414327307
CAPG	0.521100346	1.342581927	0.43849183	4.110740738	0.605862975
CAPN1	0.865234984	0.861524417	0.41152832	1.803580178	0.692543023
CAPN10	0.745545077	0.825609853	0.403248049	1.690353201	0.600171033
CAPN11	0.468279633	0.601312296	0.130639719	2.76773772	0.513755644
CAPN12	0.818681615	1.115853178	0.475923378	2.616236923	0.800935654
CAPN13	0.244158817	0.574811741	0.190532549	1.734131721	0.325692515
CAPN14	0.172500306	0.647463635	0.244000549	1.718066459	0.382647392
CAPN2	0.691526037	0.003220159	3.71E-07	27.93024787	0.214871551
CAPN3	0.492288152	55.87891393	0.05350699	58355.98396	0.256630427
CAPN5	0.080088076	0.573429856	0.080553604	4.082024696	0.57866157
CAPN6	0.38308583	0.866538555	0.38205467	1.96539691	0.731722631
CAPN7	0.263348927	0.149694911	0.018900928	1.185580201	0.072060897

CAPN9	0.976028771	0.920208616	0.636606398	1.33015298	0.658241407
CAPNS1	0.92745126	2.636978679	0.000231542	30031.89611	0.838771043
CAPNS2	0.653349672	0.070168382	0.000711023	6.924668614	0.256787578
CAPS	0.15459395	1.19648142	0.915115222	1.564357966	0.189704283
CAPS2	0.365271295	0.946580904	0.640316397	1.399332285	0.783112068
CAPSL	0.845155638	17.47552327	0.076081019	4014.061825	0.302388159
CAPZA1	0.657090483	0.444362918	0.004767556	41.41711029	0.725913279
CAPZA2	0.953358068	0.082259103	0.00059983	11.28078789	0.319797562
CAPZA3	0.527873686	1.062409944	0.824704143	1.368630069	0.639430608
CAPZB	0.702717807	0.645602467	0.174623047	2.386870189	0.511888862
CARD10	0.465836017	0.734272681	0.170045975	3.17065058	0.678984497
CARD11	0.05549846	0.396951929	0.129566946	1.216134502	0.105788114
CARD14	0.875659791	0.231030591	0.010080528	5.294874624	0.359183422
CARD6	0.948935169	0.932320841	0.077632847	11.19657696	0.955933937
CARD8	0.292831756	2544.709408	0.082784071	78222125.42	0.136912962
CARD9	0.509557511	0.572831545	0.167375628	1.960476458	0.374773048
CARF	0.907080644	1.506457792	0.274365661	8.271498232	0.637230614
CARHSP1	0.553168905	1.468021189	0.010244102	210.3733728	0.879538805
CARM1	0.260630169	102.2752179	0.366934185	28507.07465	0.107189614
CARS	0.126520865	0.973563017	0.691765582	1.370153377	0.877866786
CASC1	0.979950392	0.002459563	8.45E-09	716.0468903	0.349325736
CASC2	0.351825971	0.680282677	0.245923607	1.881822271	0.458031195
CASC3	0.394566689	0.315209787	7.67E-06	12959.97098	0.831336494
CASC4	0.424398849	0.048567142	0.002128008	1.10843885	0.05803277
CASC5	0.24041113	1.27255761	0.735576564	2.201542232	0.388767717
CASK	0.879334259	1.126911253	0.32193125	3.944721023	0.851732244
CASKIN1	0.829624141	1.011913196	0.24273137	4.218524848	0.9870281
CASKIN2	0.304662209	4.001961245	0.647935116	24.71805185	0.135484763
CASP1	0.618205721	37.94113376	0.489531786	2940.625455	0.10139103
CASP10	0.624026906	1.074212096	0.101117811	11.41175448	0.952652628
CASP12	0.373140109	0.766050166	0.324920891	1.806079183	0.542503732
CASP14	0.698523842	0.323827444	0.012650291	8.289470538	0.495523567
CASP2	0.364353314	0.713359256	0.381916232	1.332442524	0.289329316

CASP3	0.674252856	0.777466438	0.113599147	5.320938395	0.797561185
CASP4	0.525381712	0.967366987	0.759193451	1.232622445	0.788431588
CASP5	0.561989414	1.971906707	5.61E-06	692931.9935	0.916997205
CASP6	0.761498862	0.610105556	0.17162754	2.168817369	0.445112269
CASP7	0.278436591	5.661826227	0.043888125	730.4088796	0.48441831
CASP8	0.732524243	34.25802136	0.077399767	15162.99166	0.255609219
CASP8AP2	0.28650461	12.9600416	0.283456463	592.551943	0.188994074
CASP9	0.509073558	0.491630257	0.066472894	3.636073227	0.486747856
CASQ1	0.265251556	0.01047841	5.11E-06	21.47203209	0.241320665
CASQ2	0.130781809	15.73819882	0.240042909	1031.860941	0.196575297
CASR	0.680335592	1.481001332	0.675050161	3.249188093	0.32724886
CAST	0.867403269	0.906942793	0.71774562	1.14601219	0.413214253
CASZ1	0.982437275	1.032997473	0.778825652	1.370118942	0.821752468
CAT	0.754587907	1.283668277	0.01182706	139.3249267	0.91683264
CATSPER1	0.393514415	45.06459514	0.002538505	800005.5646	0.445565231
CATSPER2	0.416874242	3.089326393	0.586191379	16.28126566	0.183477657
CATSPER3	0.145271035	0.683117614	0.230834671	2.021575319	0.49118404
CATSPER4	0.321614155	2.319263883	0.439216982	12.24675999	0.321748989
CAV1	0.145549492	0.00376634	2.41E-06	5.875063508	0.136768403
CAV2	0.139263496	0.618925705	0.244271873	1.568207682	0.311809596
CAV3	0.182069685	0.420112842	0.06840363	2.580196397	0.349042865
CBFA2T2	0.83500372	1.299932893	0.644178852	2.623224156	0.46400137
CBFA2T3	0.43669098	0.026234419	1.00E-05	68.81057877	0.364697954
CBFB	0.598081841	1.460542184	0.267808625	7.965327741	0.661610947
CBL	0.222690074	1.43670748	0.887928131	2.324657043	0.139987223
CBLB	0.747670395	0.047383392	8.61E-05	26.06987054	0.343553347
CBLC	0.945210105	129.9827856	0.045063559	374926.5453	0.231143494
CBLL1	0.77056237	1.50711826	0.180853289	12.55938145	0.704549785
CBLN1	0.058301164	23.29305948	0.254751096	2129.791108	0.171804904
CBLN2	0.527003038	1.286256088	0.576608023	2.869288422	0.538586766
CBLN4	0.76100804	0.989984177	0.412730239	2.374598656	0.982008549
CBR1	0.955657163	1.130553235	0.369614926	3.458060069	0.829675716
CBR3	0.400029252	0.978085911	0.701214442	1.364278873	0.896170498

CBR4	0.734166157	2.296637544	0.714815594	7.378887715	0.162656288
CBS	0.884136128	1.035180588	0.253534375	4.226641248	0.961580522
CBWD1	0.401657865	0.022181127	1.37E-05	35.92771538	0.312454673
CBWD2	0.225820333	0.843518172	0.612415855	1.161829663	0.297531077
CBWD3	0.371536915	0.666618279	0.060122345	7.391260753	0.741113113
CBWD5	0.925744088	0.890068222	0.725357262	1.092181028	0.264672135
CBX1	0.743591374	1.343706587	0.577802273	3.124853389	0.492651507
CBX2	0.228775089	1.421807379	0.675643711	2.992015153	0.353882592
CBX3	0.744798514	2.882898336	0.000559346	14858.61426	0.808173035
CBX4	0.417534468	0.634455419	0.108648721	3.704909502	0.613314547
CBX5	0.675660148	1.085272314	0.895076711	1.315882742	0.405178727
CBX6	0.794262803	0.00198246	4.58E-08	85.85985992	0.253238833
CBX7	0.869556086	1.152527314	0.900716057	1.474736903	0.259056681
CBX8	0.097175586	0.009799729	5.23E-05	1.835848459	0.083197952
CC2D1A	0.699663321	1.370029538	0.714576942	2.626702353	0.343121861
CC2D1B	0.306719277	0.020406429	6.69E-05	6.221993956	0.182345996
CCAR1	0.529867795	0.732523646	0.26264198	2.043050739	0.551997312
CCBE1	0.089160706	2.237944597	0.384733416	13.01783473	0.369882453
CCBL1	0.246208498	1.21995677	0.989973701	1.503367735	0.062123127
CCDC12	0.588295353	0.896831987	0.677808136	1.186630213	0.445951665
CCDC13	0.416652487	194.4972205	0.021768891	1737762.761	0.256193461
CCDC14	0.372537023	1.021636256	0.77261572	1.350918204	0.880631357
CCDC15	0.574784092	118.340329	0.002714492	5159135.807	0.381134041
CCDC17	0.509522019	1.428996451	0.786922656	2.594957513	0.240900719
CCDC18	0.332374934	0.902049722	0.75829201	1.073061155	0.244488301
CCDC22	0.395408854	388.7345202	0.046562232	3245431.37	0.195572828
CCDC23	0.240523283	0.099195847	0.003152815	3.120961636	0.189130791
CCDC24	0.411737817	2.960669449	0.316428182	27.70158944	0.341407274
CCDC25	0.722908038	1.131090454	0.497824497	2.569912939	0.768618427
CCDC26	0.74831029	0.88268562	0.008045585	96.83993027	0.95847977
CCDC27	0.250489157	0.588293535	0.28836396	1.200182171	0.144739894
CCDC28A	0.540339126	1.526505524	0.469226727	4.966083513	0.482196483
CCDC28B	0.207994208	1.340903351	0.738010767	2.436308354	0.335634468

CCDC3	0.127865291	1.694265408	0.847842613	3.38569356	0.135523623
CCDC33	0.829933545	1.360055215	0.000778985	2374.563226	0.935647564
CCDC34	0.964077812	1.246310083	0.210310932	7.385678006	0.808364288
CCDC36	0.840258257	1.848134412	0.003674125	929.6365139	0.846557985
CCDC37	0.610435434	1.174902003	0.483565893	2.854615546	0.721944814
CCDC38	0.633753171	1.236398406	0.27247603	5.610332105	0.78331749
CCDC40	0.538205671	1.301388861	0.83094825	2.038169005	0.249772654
CCDC42	0.097719035	1.936667949	0.59267142	6.328435322	0.273922627
CCDC43	0.509543306	0.954897531	0.349479801	2.609104421	0.928294704
CCDC47	0.189199025	0.81374754	1.03E-06	644820.2426	0.976274107
CCDC50	0.707671602	0.804099731	0.219152391	2.950350546	0.742359607
CCDC51	0.325212453	0.085979452	0.000246869	29.94494368	0.411282341
CCDC53	0.64848178	6.045580266	0.481491734	75.90792984	0.163374059
CCDC54	0.994077197	1.086198071	0.418562312	2.818758919	0.865057447
CCDC57	0.612104511	0.674218891	0.000235611	1929.324858	0.922668176
CCDC58	0.522572691	0.810368976	0.179570199	3.657053785	0.784484471
CCDC59	0.914778516	16.25183967	0.088064319	2999.19758	0.294953881
CCDC6	0.420550371	0.519416286	0.201526807	1.33874635	0.175087205
CCDC60	0.555316668	0.730165413	0.00201961	263.9824695	0.91666017
CCDC62	0.956679655	129.8632589	0.033254352	507135.6077	0.248773431
CCDC63	0.827389029	9.422240567	0.240901085	368.5272617	0.230498418
CCDC64	0.661992914	0.204444849	0.002061997	20.27048939	0.4984826
CCDC65	0.17955633	1.52079742	0.008256067	280.1363955	0.874826853
CCDC66	0.80383747	1.858085595	0.051861538	66.5711476	0.734377851
CCDC67	0.909699063	68.47323541	0.177514711	26412.36849	0.164221881
CCDC68	0.885037957	0.11369036	0.001288567	10.03090665	0.341482722
CCDC69	0.959011716	1.326069058	0.175525147	10.01827476	0.784442468
CCDC7	0.659845999	0.684817634	0.003339545	140.4308663	0.889137467
CCDC70	0.479042783	0.145142598	0.000843448	24.97649877	0.462452447
CCDC71	0.188309735	1.180433122	0.478225044	2.91373773	0.718978141
CCDC74A	0.638128508	1.263650932	0.250264573	6.380502267	0.776989301
CCDC74B	0.264126669	0.457201045	0.166227006	1.257514052	0.129497095
CCDC77	0.472343326	1.485661774	0.008262026	267.1488605	0.881208337

CCDC8	0.584940232	0.874128369	0.525090542	1.45517838	0.604912807
CCDC9	0.265642309	1.561659045	0.212228154	11.49130749	0.661577838
CCHCR1	0.997081878	0.238582769	0.000374038	152.1815722	0.663626999
CCIN	0.335407395	0.000779506	1.51E-07	4.022911194	0.10083394
CCK	0.164442075	0.594453535	0.139469962	2.533699727	0.481970851
CCKAR	0.837245193	6.704407954	0.706731735	63.60134093	0.097401114
CCKBR	0.594427475	0.938846341	0.647296845	1.361712881	0.739427633
CCL1	0.848162513	0.933485361	0.384197161	2.268093073	0.879219484
CCL11	0.441126344	0.998206504	0.768422353	1.296703851	0.989270161
CCL13	0.385283414	0.556764073	0.222595363	1.392599686	0.210583492
CCL14	0.456838454	0.022590102	0.000201698	2.530087865	0.115398029
CCL15	0.90112248	0.091500087	0.002017342	4.150146291	0.2191706
CCL16	0.582359608	4326.58462	0.100962778	185408273.9	0.123904974
CCL17	0.859234001	0.923146058	0.715056273	1.191792417	0.539468022
CCL18	0.197850649	0.970787929	0.359738448	2.619762241	0.953324069
CCL19	0.863124449	29.93864054	0.012408425	72234.97008	0.392336614
CCL2	0.718048523	1.733788286	0.239039231	12.57543294	0.586203947
CCL20	0.079200917	1.173829697	0.92328647	1.49236039	0.190742839
CCL21	0.867292374	24.79209974	0.001448006	424478.979	0.51859527
CCL22	0.378056297	1.002555553	0.808192214	1.243661619	0.981481022
CCL23	0.865473741	0.396588674	9.19E-06	17112.40597	0.865130013
CCL24	0.987473987	0.88353479	0.224290872	3.480452493	0.859492868
CCL25	0.535584976	0.971329064	0.66535162	1.418017364	0.880216515
CCL26	0.706039666	0.663371542	0.291050731	1.511976287	0.3288582
CCL28	0.863192421	0.950770929	0.217551256	4.155183358	0.946512101
CCL3	0.149552568	1.290959702	0.756097758	2.204181845	0.349451484
CCL3L3	0.609367444	1.447273042	0.107519903	19.48103739	0.780472565
CCL4	0.12558506	1.320866908	0.607446752	2.872168437	0.482569896
CCL4L1	0.438168591	0.78233931	8.88E-05	6892.723566	0.957760812
CCL5	0.536093223	2.900341359	0.285718797	29.44146512	0.367843083
CCL7	0.505784815	0.925792724	0.699533254	1.225234342	0.589701993
CCL8	0.180800908	0.692694775	0.205915233	2.330211532	0.553043944
CCM2	0.795281049	0.269653328	0.001143603	63.58233237	0.638201222

CCNA1	0.419071015	1.714445338	0.502771793	5.846236521	0.389058393
CCNA2	0.480810925	0.817769516	0.172924649	3.867273906	0.799669984
CCNB1	0.780751885	1.149800182	1.62E-05	81394.60498	0.980454882
CCNB1IP1	0.904074005	0.760832656	0.336735303	1.719054476	0.51101623
CCNB2	0.599723974	1.03774593	0.803946542	1.339537594	0.776047371
CCNB3	0.765621051	0.835273261	0.413578617	1.686937843	0.615742692
CCNC	0.901625572	0.91068192	0.480982046	1.724267186	0.773912055
CCND1	0.162702622	1.330864252	0.737648022	2.401144724	0.342454664
CCND2	0.438190037	1.819762561	0.554396891	5.973222128	0.32351342
CCND3	0.866196028	0.800321367	0.28972635	2.210756082	0.667443902
CCNDBP1	0.198838114	0.9485102	0.752969467	1.194831449	0.65358981
CCNE1	0.445365367	1.18400714	0.913642427	1.534378074	0.201572012
CCNE2	0.755158041	1.435092353	0.478183247	4.306905504	0.519430072
CCNF	0.635955789	1.208248785	0.356608716	4.093744945	0.761251037
CCNG1	0.20062012	1.601894889	0.942457657	2.722740078	0.081685591
CCNG2	0.372006853	0.719753042	0.018120623	28.58866565	0.861036212
CCNH	0.262779823	0.816447892	0.411055982	1.621645684	0.562455372
CCNI	0.519874669	0.835814975	0.313124185	2.231021132	0.720321518
CCNJ	0.822976398	0.641581456	0.302113857	1.362488864	0.248089442
CCNK	0.155792927	0.257497765	0.041499811	1.597720485	0.145165465
CCNL1	0.543802478	0.875179332	0.160247505	4.779724097	0.877671118
CCNL2	0.459709944	29.55469362	0.282770494	3089.006579	0.153438994
CCNT1	0.083021614	0.040185995	0.001379382	1.170752282	0.061716187
CCNT2	0.632105402	0.91146575	0.615760629	1.349176571	0.643173366
CCPG1	0.274227611	0.105069269	0.00023962	46.0710751	0.467881756
CCR1	0.258420548	1.43088417	0.877726796	2.33265011	0.150740841
CCR10	0.781086486	1.446815904	0.343623053	6.091780631	0.614552448
CCR2	0.250898266	0.910008417	0.083366102	9.933477698	0.938363586
CCR3	0.492029181	5.344084976	0.001788508	15968.19185	0.681447414
CCR4	0.094999233	0.610127833	0.225577941	1.650232161	0.33042614
CCR5	0.057772125	1.711591782	0.776470031	3.772903409	0.182656364
CCR6	0.754411173	1.087709457	0.2058772	5.746687155	0.921142205
CCR7	0.133153315	0.093611302	0.000151116	57.98924887	0.470223651

CCR8	0.548126158	1.037483376	0.599523802	1.795377851	0.895371496
CCR9	0.702333795	0.554713885	0.061204507	5.027530027	0.600280141
CCRL2	0.818075959	0.064566743	0.001937348	2.151841175	0.125618381
CCRN4L	0.669342371	0.080863802	0.000978422	6.683160475	0.264168509
CCS	0.558524342	0.925081592	0.381065741	2.245743607	0.863366251
CCT2	0.643844457	0.578581094	0.239698292	1.396572665	0.223591801
CCT3	0.94004711	0.486471423	0.000432369	547.3440065	0.840682033
CCT4	0.494078719	1.077328679	0.388977854	2.983812759	0.886049537
CCT5	0.742914636	1.083265876	0.357670908	3.280851005	0.887503862
CCT6A	0.556656805	0.828411187	0.344971817	1.989336696	0.673639277
CCT6B	0.486790447	2.53E-05	3.13E-10	2.051977945	0.066459291
CCT7	0.426311108	0.808263318	0.316894051	2.06153946	0.655895441
CCT8	0.901517193	1.198144106	0.039875216	36.00104108	0.917071026
CD109	0.190984817	0.824567864	0.495297028	1.372736205	0.458241714
CD14	0.301999239	0.077188323	0.000302292	19.70951637	0.365044901
CD151	0.57130376	6.266709352	0.013083331	3001.65492	0.560008449
CD160	0.771972743	0.765500654	0.453691357	1.291607703	0.316719854
CD163	0.059195202	0.442136027	0.138568016	1.410745938	0.167999197
CD164	0.611670081	0.801853213	0.358249669	1.79474995	0.591130582
CD164L2	0.721245077	0.003867681	3.19E-08	468.3327637	0.352246505
CD177	0.080905246	1060.746789	0.840053627	1339418.954	0.055859843
CD180	0.421879616	0.457995939	0.001660721	126.3067646	0.78534941
CD19	0.124244061	0.000154915	6.09E-09	3.943579266	0.090098955
CD1A	0.331988696	0.000444684	1.01E-07	1.958202627	0.071391837
CD1B	0.727314119	0.64819449	0.188352557	2.230689638	0.491713403
CD1C	0.570242381	6.149966056	0.481719388	78.51476072	0.162149927
CD1D	0.767756939	1.214713688	0.417045215	3.538056049	0.721391667
CD1E	0.57591135	0.100314019	0.000210579	47.78686532	0.464843923
CD2	0.94377116	1.474390289	0.15199763	14.30171459	0.737698084
CD200	0.859467505	55.13888274	0.003115186	975959.9741	0.421692904
CD200R1	0.563809166	0.555174207	0.063343748	4.865806152	0.595181996
CD207	0.062931062	0.739612121	0.196518549	2.783585029	0.655560109
CD209	0.059271199	1.165723286	0.341851213	3.975152724	0.806457984

CD22	0.503635992	1.349878683	0.813892748	2.238836093	0.245143838
CD226	0.418223164	1.342184138	0.883925761	2.038019866	0.167280975
CD24	0.858194013	1.856687988	0.019955121	172.7521645	0.789046871
CD244	0.209428899	1.159543092	0.940867832	1.429042568	0.165044756
CD248	0.911443909	3.12801593	0.017420952	561.6503298	0.666742445
CD274	0.375975651	7.187510913	0.001582607	32642.54153	0.646193768
CD276	0.486584871	0.486333168	0.001043362	226.6901102	0.818137312
CD28	0.801352991	0.018266292	2.30E-05	14.52557464	0.240127823
CD2AP	0.730041403	1.23705264	0.707676973	2.162426211	0.45533654
CD2BP2	0.536557776	1.952890447	0.88753023	4.297071769	0.096225122
CD300A	0.346321837	5.570098682	0.008246762	3762.203736	0.605409834
CD300C	0.504252508	1.046629938	0.175525251	6.240892524	0.960100613
CD300E	0.540413797	0.075433256	1.25E-05	453.827705	0.560501202
CD300LB	0.89148191	0.930309353	0.290065808	2.983721166	0.903303945
CD300LF	0.549743113	1.209534649	0.947674168	1.543752184	0.126458295
CD300LG	0.2762102	1.151600866	0.855641505	1.549930137	0.351688239
CD302	0.699946589	0.685375765	0.205617022	2.284538185	0.538543477
CD320	0.55899282	0.602558746	0.255724728	1.41979638	0.246694386
CD33	0.966164558	0.913566195	0.187431922	4.452833782	0.910934359
CD34	0.27083591	0.748660077	0.292585104	1.915654294	0.545931792
CD36	0.149054261	0.822797504	0.264214906	2.562291968	0.736470483
CD37	0.475280849	0.977459182	0.328202484	2.911088426	0.967339461
CD38	0.687862766	2.851650255	0.57088383	14.24442023	0.20163974
CD3D	0.398941535	4.014450709	0.000864044	18651.61968	0.746981235
CD3E	0.720690123	0.986352363	0.624776482	1.557182467	0.952965151
CD3EAP	0.162522141	0.577432161	0.229139795	1.455128738	0.244202587
CD3G	0.763247938	1.057930926	0.443505622	2.523570814	0.898970663
CD4	0.983096164	0.903835519	0.33162282	2.463396952	0.843323435
CD40	0.70122492	0.892422353	0.391126947	2.0362127	0.786834016
CD40LG	0.064505651	0.000981848	7.35E-07	1.310875342	0.059262212
CD44	0.491149708	0.029786158	3.27E-06	271.1638053	0.449996297
CD46	0.485702147	0.989009928	0.723602902	1.351764393	0.944736066
CD47	0.243885318	0.003100924	7.82E-08	123.0403392	0.284998121

CD48	0.922275773	0.525789852	7.45E-05	3708.617559	0.88693115
CD5	0.145282632	0.033062734	0.000602894	1.813162008	0.095175695
CD52	0.311546957	2.56953445	0.743216045	8.883698541	0.135942221
CD53	0.077708515	1.432522444	0.940288889	2.182436245	0.094260935
CD55	0.132602772	0.482135882	0.021726756	10.69902066	0.644590777
CD58	0.521085395	1.115509735	0.666093461	1.868149204	0.677776682
CD59	0.810341159	1.68660008	0.344271665	8.262718427	0.519101784
CD5L	0.431217314	1.155231585	0.913147732	1.461494091	0.22909345
CD6	0.502447424	0.699199544	0.288709494	1.693328458	0.427848959
CD63	0.666137217	0.901201171	0.753127473	1.078387897	0.255994967
CD68	0.125183075	0.027853817	3.24E-05	23.94123356	0.298920149
CD69	0.200060405	0.198162183	0.027591609	1.423195392	0.107586272
CD7	0.147612535	0.519994835	0.198266804	1.363791734	0.183757996
CD72	0.09883094	0.425202636	0.09132174	1.97978359	0.275848456
CD74	0.261488493	1.418971004	0.614261429	3.277885631	0.412696007
CD79A	0.617582624	2.465659148	0.593770939	10.23875477	0.214100227
CD79B	0.215544149	0.442049708	0.050149473	3.896510419	0.462250337
CD80	0.51423668	2.822107751	0.087261545	91.26920838	0.558590537
CD81	0.369969748	7.742040883	0.455002475	131.7337823	0.15695331
CD82	0.129703227	0.669840784	0.259085785	1.731807382	0.40833494
CD83	0.211754709	51.06490188	0.000367384	7097823.106	0.515076128
CD84	0.73168591	1.09483729	0.664822713	1.802989981	0.721845431
CD86	0.834490152	0.970321449	0.669432326	1.406450925	0.873606964
CD8A	0.56307933	7.4797308	0.00280141	19970.78752	0.617171579
CD9	0.568672484	3.156289909	0.040439697	246.3462066	0.605151012
CD96	0.249635724	1.189890859	0.942588666	1.50207647	0.143581747
CD97	0.304440529	0.950885513	0.42198801	2.142675232	0.903296668
CD99	0.055071481	0.227190965	0.021498777	2.400868363	0.217981799
CD99L2	0.26306901	2.06578821	0.943972162	4.520769889	0.0694214
CDA	0.351096396	0.936429806	0.314968327	2.78409194	0.905952224
CDADC1	0.943154766	0.966241928	0.358304263	2.605672222	0.945906779
CDAN1	0.80067914	0.450704222	0.13554557	1.498642084	0.193593245
CDC14A	0.643309657	2.425197848	0.200496645	29.33507738	0.486097369

CDC14B	0.097663547	0.671993205	0.237368295	1.902422845	0.454052035
CDC14C	0.952379929	0.865370846	0.482807608	1.551066488	0.627204355
CDC16	0.160012323	1.171126979	0.900240963	1.523523653	0.239213945
CDC20	0.581115042	0.42654881	0.00955713	19.03750333	0.660198009
CDC23	0.775006049	1.075318175	0.56709978	2.038987173	0.823970578
CDC25A	0.204118403	0.023453467	0.000220973	2.489292128	0.11484774
CDC25B	0.205911275	0.032017785	0.000188157	5.448319254	0.189145902
CDC25C	0.443896959	1.99239624	0.260204122	15.25587969	0.506872223
CDC26	0.286126508	1.601868296	0.642742999	3.992236464	0.31188462
CDC27	0.714414964	1.531891657	0.000873142	2687.640385	0.910897639
CDC34	0.107157673	0.028720073	0.000175175	4.708676779	0.172420982
CDC37	0.213552002	0.243025749	0.005331829	11.07715785	0.46790235
CDC37L1	0.967682775	1.487602357	0.552734954	4.00365629	0.431715354
CDC40	0.410872435	1.983803212	0.778578832	5.054690702	0.15115016
CDC42	0.318795109	1.44300466	0.864094438	2.409762588	0.161016539
CDC42BPA	0.125116461	0.001101956	2.69E-07	4.510273571	0.108498099
CDC42BPB	0.750151665	1.967747457	0.535383962	7.232248878	0.308098356
CDC42BPG	0.56942848	149.8709599	0.001290058	17411082.7	0.399842466
CDC42EP1	0.221740513	0.981546332	0.679250999	1.418375833	0.921007361
CDC42EP2	0.978278239	2.539602308	0.122464106	52.66506305	0.546851374
CDC42EP3	0.576484391	0.65571645	0.216812683	1.983113061	0.454814639
CDC42EP4	0.684226501	1.940327289	0.770411846	4.88682775	0.159572545
CDC42EP5	0.944455335	1.013027371	0.803553014	1.277108587	0.912798965
CDC42SE1	0.986081633	0.054578462	0.000257766	11.55622836	0.287183209
CDC42SE2	0.394160096	4.187630082	0.962150792	18.22608872	0.056320861
CDC5L	0.982922238	1.66745643	0.45978504	6.047197511	0.436644799
CDC6	0.16238166	0.202400911	0.003673403	11.15209094	0.434814234
CDC7	0.932283408	0.850341713	0.499070686	1.44885494	0.550998846
CDC73	0.332760441	0.031655356	7.34E-05	13.6540972	0.264647197
CDCA2	0.800068239	0.934089167	0.586071078	1.488765791	0.774346545
CDCA3	0.765693304	1.374128311	0.330010086	5.721730012	0.662337324
CDCA4	0.503281094	0.807728623	0.02086807	31.26429723	0.908863834
CDCA5	0.088105611	0.374671591	0.057519801	2.440530033	0.304523319

CDCA7	0.208296717	0.209526369	0.000747751	58.71114633	0.586745844
CDCA7L	0.382111782	0.893835832	0.428666493	1.863785736	0.764675383
CDCA8	0.646071369	1.046691267	0.55291705	1.981423091	0.88854158
CDCP1	0.776409227	2.228620173	0.343829689	14.44537235	0.400689945
CDCP2	0.870763679	0.921183224	0.442448473	1.917914928	0.826325566
CDH1	0.910863208	1.16670096	0.325802975	4.17795796	0.812741864
CDH10	0.512859134	0.598594208	0.195473929	1.83305788	0.368807206
CDH11	0.915482313	1.209810516	0.464436522	3.151434945	0.696599592
CDH12	0.63658161	1.078486188	0.896934185	1.296786852	0.421743449
CDH13	0.639350608	1.34699567	0.565616026	3.20782519	0.501054079
CDH15	0.636985334	0.00622669	2.28E-06	16.97400423	0.208256697
CDH16	0.122549197	0.653373591	0.280102281	1.524075587	0.324691648
CDH17	0.629916471	0.978776007	0.711477407	1.34649739	0.895124548
CDH18	0.310511258	0.89565922	0.667038995	1.202636495	0.463651072
CDH19	0.735484835	1.832537143	0.534939208	6.277708431	0.33497414
CDH2	0.867954751	0.671474899	0.409778837	1.10029728	0.113961096
CDH20	0.953976566	0.852319144	0.072947767	9.958466876	0.898619289
CDH22	0.488806688	1.067763828	0.901670165	1.264453054	0.447210043
CDH23	0.244723645	1.062565535	0.900536122	1.253748172	0.472202458
CDH24	0.480468942	0.147356596	0.001446383	15.01259335	0.416963179
CDH26	0.336351762	1.567126748	0.516680538	4.753200605	0.427457961
CDH3	0.288805369	0.241179153	0.005110432	11.38208828	0.469542259
CDH4	0.142794451	0.764245957	0.542903749	1.075829526	0.12330952
CDH5	0.73357239	1.383617296	0.003062388	625.1320072	0.917088391
CDH6	0.873346112	0.868851894	0.270315312	2.792677962	0.813441237
CDH7	0.129047252	1.388458721	0.550408513	3.502521442	0.486937954
CDH8	0.323781455	0.769909437	0.45675718	1.29775856	0.326313837
CDH9	0.377482278	0.000463925	3.98E-08	5.405129773	0.108108088
CDIPT	0.544846186	0.747789987	0.285585466	1.958047352	0.554001859
CDK10	0.759885502	0.461756689	0.038690287	5.510924224	0.541318624
CDK2	0.685479378	0.546802487	0.000842861	354.7356339	0.855011976
CDK2AP1	0.832410116	1.358728526	0.636621591	2.899906682	0.42806281
CDK2AP2	0.350164383	1.24528012	0.512510617	3.025737466	0.628189061

CDK3	0.130609888	0.482154581	0.115105413	2.019653406	0.318203693
CDK4	0.397760107	0.016345284	2.27E-05	11.77634312	0.220429641
CDK5	0.357159892	0.647214681	0.330130131	1.268853715	0.205260195
CDK5R1	0.06884782	0.533311336	0.222352844	1.279142538	0.159010391
CDK5R2	0.192726128	1.173461891	0.846445028	1.626818949	0.337192536
CDK5RAP1	0.325492492	1.060844861	0.560403347	2.008181829	0.856049273
CDK5RAP2	0.904324184	1.701793261	0.157196774	18.42340804	0.661754823
CDK5RAP3	0.45807224	1.352929512	0.46088543	3.971525562	0.582217597
CDK6	0.262824408	0.003919156	1.09E-06	14.1006184	0.184659111
CDK7	0.06423162	2.148414718	0.474357257	9.730399884	0.321067351
CDK8	0.980986752	1.093047638	0.474903697	2.515779819	0.834305463
CDK9	0.476618955	4.146316199	0.727190408	23.64159075	0.10931329
CDKAL1	0.070104017	0.299396578	0.007612728	11.77479503	0.519759922
CDKL1	0.458279688	1.052070486	0.592712162	1.867436469	0.862350795
CDKL2	0.920531238	1.347004224	0.286955802	6.322995976	0.705750194
CDKL3	0.717814683	0.804678536	0.325319886	1.99037186	0.638136845
CDKL4	0.467127314	0.774937528	0.349635837	1.717581863	0.530070993
CDKL5	0.614789474	584.7348602	0.004622995	73959596.42	0.287810736
CDKN1A	0.817332045	1.820874786	0.928003808	3.57281399	0.081386956
CDKN1B	0.34906782	0.032008538	7.23E-05	14.17256145	0.268244738
CDKN1C	0.09358179	1.650193928	0.800264693	3.402799128	0.174929024
CDKN2A	0.363896524	0.010285569	1.84E-05	5.760234293	0.156297396
CDKN2B	0.386900238	0.754056869	0.186292101	3.052205434	0.692313372
CDKN2C	0.211615823	1.143485413	0.797905232	1.638739585	0.465210149
CDKN2D	0.921340019	0.756311074	0.211866619	2.699842217	0.667052932
CDKN3	0.925695336	3.605239345	0.005932133	2191.075461	0.694964978
CDO1	0.088668923	1.408418159	0.765741679	2.590484186	0.270683421
CDON	0.924244332	1.041683922	0.025506223	42.54277002	0.982785697
CDR1	0.900799613	5.659318059	0.008717619	3673.925166	0.599855546
CDR2	0.134313241	1.0029924	0.881011363	1.141862405	0.963978428
CDR2L	0.085561891	0.002648208	1.32E-08	530.0211799	0.340710221
CDRT1	0.179564347	0.352701529	0.073163116	1.700288019	0.194095327
CDRT15	0.271366255	1.015719641	0.749716327	1.376102338	0.919809672

CDS1	0.050785765	1.311009071	0.805286541	2.134326971	0.276132309
CDS2	0.89436798	1.573876634	0.014499591	170.8384449	0.849582986
CDSN	0.268858584	0.017181583	5.43E-06	54.34137664	0.322992326
CDT1	0.380635374	0.216330389	0.036560872	1.280025205	0.091451209
CDV3	0.315308889	0.357407019	0.054442169	2.346338868	0.283876026
CDX1	0.561360403	7.335410218	0.000167851	320571.8849	0.714722519
CDX2	0.745468826	0.100437568	0.00312093	3.232275203	0.194431662
CDX4	0.30592574	48.27432891	0.052538989	44355.83684	0.265426896
CDY2B	0.625843837	0.952763432	0.685673473	1.323892776	0.773118614
CDYL	0.952730805	1.659972832	0.219031449	12.58042994	0.623821238
CDYL2	0.316325447	1.263254531	0.649956702	2.455258948	0.490673692
CEACAM1	0.458586	5.639942915	0.585257782	54.35033423	0.134517096
CEACAM16	0.664949647	0.993940888	0.808044382	1.222604241	0.9541247
CEACAM19	0.50957148	14.73167008	0.020625021	10522.27315	0.422362852
CEACAM20	0.215170447	33.46388303	0.461359969	2427.240208	0.108262903
CEACAM21	0.515882847	1.845400968	0.368572321	9.239719154	0.455969242
CEACAM3	0.620559672	0.590106247	0.282546303	1.232454218	0.160400465
CEACAM4	0.522889884	25.58144912	0.016742327	39087.19173	0.386138246
CEACAM5	0.397422275	0.776502753	0.311360268	1.93652366	0.587459412
CEACAM6	0.162025802	1.184119681	0.882991026	1.587943	0.258984734
CEACAM7	0.966055318	1.297887167	0.582418063	2.892271386	0.523632727
CEACAM8	0.729048483	1.181661948	0.572715519	2.438077743	0.651485156
CEBPA	0.522739851	61.53646118	0.06145139	61621.65054	0.242546936
CEBPB	0.460800044	0.917248642	0.715520821	1.175849879	0.495472397
CEBPD	0.920797555	0.698927593	0.21957532	2.224748123	0.544275728
CEBPE	0.42933188	0.86092101	0.000243416	3044.931805	0.971345395
CEBPG	0.107688048	14.19946581	0.176849837	1140.090556	0.235730929
CEBPZ	0.364698394	0.926958761	0.525194915	1.636064099	0.793588455
CECR1	0.946668132	0.713321926	0.236637887	2.150239663	0.548458652
CECR2	0.136617058	2.198503822	0.25084549	19.26851086	0.476898586
CECR5	0.540440185	1.808107713	0.811627284	4.028023162	0.147265247
CECR6	0.893319668	1.156118229	0.601243599	2.223074576	0.663656723
CECR7	0.540925953	1.076327871	0.835339916	1.386838656	0.569516749

CEL	0.851986545	1.268966327	0.561870954	2.86591704	0.566600065
CELP	0.139329796	6.103241771	0.780716024	47.71204762	0.084703377
CELSR1	0.424428434	0.80866849	0.327149369	1.998917891	0.645561482
CELSR2	0.846362289	0.913529462	0.736846898	1.13257731	0.409538867
CELSR3	0.129685842	1.050053082	0.448683642	2.457436317	0.910361904
CENPA	0.404706675	0.707764466	0.213577501	2.34542748	0.571780537
CENPB	0.910954316	1.103282434	0.335690309	3.626056798	0.871380158
CENPE	0.087099429	1.151109587	0.634995415	2.086713148	0.642886562
CENPF	0.488068692	0.285026025	0.067328843	1.206612661	0.088220741
CENPH	0.442691406	2.37697482	0.6634552	8.516037401	0.183582858
CENPJ	0.73400426	1.024463878	0.761982165	1.377363258	0.872851902
CEP135	0.809886656	1.045798678	0.455833897	2.399327652	0.915825332
CEP152	0.543377087	0.66929574	0.22380067	2.001588233	0.472512342
CEP164	0.462289759	0.9135973	0.349532314	2.387933802	0.853746591
CEP170	0.885047606	0.669087227	0.263988849	1.695820558	0.397068744
CEP192	0.73022111	0.751565585	0.22753675	2.482459768	0.639442771
CEP250	0.54626563	5.369429268	0.016580206	1738.866866	0.568747931
CEP290	0.294242803	13.21430926	0.029486571	5921.949079	0.407279553
CEP350	0.712871013	0.855871586	0.1995869	3.670161576	0.834039726
CEP55	0.310027269	0.644948564	0.004917807	84.58213391	0.860071605
CEP57	0.094644331	1.64282104	0.620264089	4.351148189	0.31784218
CEP63	0.096987704	0.710209411	0.020342814	24.79486866	0.85026811
CEP68	0.850053806	3.13605646	0.219465869	44.81266346	0.399608524
CEP70	0.288384706	0.440271703	0.123904931	1.56441855	0.204737743
CEP72	0.429993095	0.470350298	0.183818653	1.203519881	0.115600762
CEP76	0.674313553	2.251110693	0.03130555	161.8722329	0.709907025
CEPT1	0.148987138	2.82870295	0.00894439	894.5898094	0.723314311
CER1	0.488268427	1.681701829	1.95E-05	144859.5328	0.928562227
CERK	0.842607884	1.044460291	0.489434857	2.22889172	0.910444208
CERKL	0.934376168	0.123398659	0.00037729	40.35947958	0.478787925
CES1	0.98754849	0.949248825	0.232342234	3.878215836	0.942179935
CES2	0.950926846	0.83702085	0.382709694	1.830640598	0.655908609
CETN1	0.3835443	1.719403213	0.685791437	4.310854948	0.247811068

CETN2	0.410166554	1.354941262	0.638228446	2.876502662	0.429039637
CETN3	0.476204886	0.755064097	0.000117839	4838.150558	0.949907601
CETP	0.930405265	2.025139086	0.459875281	8.918044702	0.35085102
CFB	0.479259821	1.410327799	0.641544957	3.100366511	0.392272114
CFC1	0.667818852	0.486994914	0.084350335	2.811655042	0.421211149
CFD	0.221587562	1.890711403	0.000181337	19713.49197	0.892665624
CFDP1	0.832623207	1.028042039	0.26743936	3.951813346	0.967889368
CFH	0.864246754	1.24429954	0.02328601	66.48976434	0.914251029
CFHR1	0.495055554	0.35182467	0.001985607	62.33890887	0.692497248
CFHR3	0.95580013	1.41324009	0.677234086	2.949124377	0.35675732
CFHR4	0.180740583	0.755394966	0.342766725	1.664751893	0.486566978
CFHR5	0.548953628	0.002363619	3.49E-07	16.00337365	0.179005678
CFI	0.434119622	1.062880935	0.157608968	7.167840092	0.950066272
CFL1	0.680742509	12.4252482	0.000133048	1160386.756	0.666088022
CFL2	0.28492422	0.623303652	0.272729779	1.424514197	0.262312624
CFLAR	0.485203253	0.889960663	0.495374692	1.598850314	0.69653376
CFTR	0.240257127	3.997779156	0.003119345	5123.588432	0.704280773
CGA	0.700564911	2.550273447	0.000589593	11031.16355	0.82652069
CGB	0.222674326	0.629515282	0.263949068	1.501386205	0.296675498
CGB1	0.620383883	0.215552602	0.004168415	11.14642426	0.44589915
CGB2	0.223368998	0.892420312	0.537715886	1.481105605	0.659690859
CGB5	0.965271043	0.392252821	0.023567864	6.528477635	0.514219707
CGB7	0.923923701	2.569767267	0.247841621	26.64485395	0.428976793
CGB8	0.090360905	1.391491386	0.990622006	1.954578302	0.056700008
CGGBP1	0.775873195	0.026322378	2.18E-05	31.80921719	0.315136423
CGN	0.548476811	0.878726638	0.559378792	1.38038931	0.574777963
CGNL1	0.61489031	0.029867944	9.12E-05	9.776788375	0.23471853
CGREF1	0.11956989	0.351635692	0.157833294	0.783406699	0.010551322
CGRRF1	0.826914955	0.854600371	0.683354558	1.068759673	0.168474596
CH25H	0.733584425	1.701115139	0.066120288	43.76557948	0.748483525
CHAC1	0.747393013	1.025387277	0.845881858	1.242985717	0.798469022
CHAC2	0.540135336	2.216441213	0.663609781	7.402862029	0.19583053
CHAD	0.800489039	0.831215474	0.005888848	117.3267046	0.941646374

CHAF1A	0.745489022	0.99079349	0.860605463	1.140675701	0.897606039
CHAF1B	0.197768428	19.14197974	0.882635582	415.1377941	0.060048754
CHAT	0.47062022	0.584369467	0.256096849	1.333431765	0.201842473
CHCHD1	0.558452332	0.6104204	0.240992846	1.546158197	0.297891814
CHCHD2	0.09835669	73.17743243	0.622172818	8606.831521	0.077584924
CHCHD3	0.664754245	0.451049221	0.025160142	8.086019482	0.588749953
CHCHD4	0.940818298	1.307393242	0.423807479	4.033145175	0.640970749
CHCHD5	0.892056568	0.61991962	0.237091106	1.620897304	0.329523948
CHCHD6	0.957148662	0.216138937	0.031701119	1.473640094	0.117801351
CHCHD7	0.627073439	1.405282447	0.354212409	5.575238772	0.628460044
CHD1	0.246826504	14.02421062	0.036691496	5360.328877	0.38404056
CHD1L	0.990009997	2.370149376	0.369645299	15.19729339	0.362701279
CHD2	0.213751509	2.500277903	0.087052388	71.81181047	0.592696502
CHD3	0.504047163	14.32977205	0.000286085	717765.8279	0.629668254
CHD4	0.706960916	0.095584255	0.000122477	74.59622824	0.489607773
CHD5	0.348940332	0.723214122	0.469352815	1.114382719	0.141830771
CHD6	0.656371028	1.713736881	0.50264186	5.84291586	0.389362242
CHD7	0.99637679	0.739450079	0.361282326	1.513460197	0.408810324
CHD8	0.497989101	1.076414374	0.874301466	1.32524987	0.487697776
CHD9	0.294875609	0.869884962	0.000726656	1041.345942	0.969251599
CHDH	0.142532405	1.332503175	0.571592182	3.106348836	0.506218816
CHEK1	0.505452559	0.192975663	0.006679655	5.575079279	0.337720354
CHEK2	0.481795935	1.179345444	0.532005641	2.614362647	0.684637236
CHERP	0.461091598	0.807279465	0.010348073	62.97792081	0.923276282
CHFR	0.064467526	12.76150273	0.502531492	324.0711367	0.122827982
CHGA	0.625860761	1.669021743	0.288245954	9.664085612	0.567540686
CHGB	0.187446807	0.036033087	8.68E-07	1495.639014	0.540176724
CHI3L1	0.193219649	1.070284233	0.849996852	1.347661859	0.56346691
CHI3L2	0.714587455	4.129897301	0.013799238	1236.014061	0.625867497
CHIA	0.083765105	1.458080035	0.958894669	2.217133393	0.077788308
CHIC1	0.274606968	188.044643	0.016051646	2202938.369	0.273280061
CHIC2	0.221502599	1.313420966	0.485822016	3.550836678	0.591071242
CHID1	0.851635139	4.043281082	0.00526591	3104.519913	0.680225282

CHIT1	0.760099283	4.631765662	0.001981516	10826.6857	0.698507132
CHKA	0.694432307	0.067406203	0.000523144	8.685178381	0.276607606
CHKB	0.246225228	0.394522966	0.003501483	44.45212998	0.699611767
CHL1	0.096747582	0.526725674	0.203874841	1.360834585	0.185580424
CHM	0.49159063	0.015893254	1.16E-07	2169.751495	0.49236776
CHML	0.410371872	2.418666423	0.594494697	9.840200914	0.2173491
CHMP1B	0.194922619	0.868570143	0.125352737	6.018329655	0.886549255
CHMP2A	0.290232857	0.602418773	0.177173724	2.048319414	0.41699281
CHMP2B	0.613822625	0.940710945	0.202710255	4.365526955	0.937790319
CHMP4A	0.162156995	0.224109031	0.020261331	2.478852875	0.222591736
CHMP4B	0.5249497	0.290758797	0.025374808	3.331677572	0.320829566
CHMP4C	0.940405246	0.65394725	0.241098342	1.773745114	0.404128877
CHMP5	0.66100666	1.146173807	0.724431725	1.81344128	0.560013933
CHMP6	0.364968835	0.699087717	0.307496508	1.589363206	0.39295309
CHMP7	0.098456326	1.364117539	0.952093345	1.954447713	0.090571296
CHN1	0.094174286	0.06860288	9.10E-05	51.70193205	0.427952864
CHN2	0.50083316	0.547704774	0.200910016	1.493108833	0.239376086
CHODL	0.82621901	0.479848743	8.27E-06	27826.54921	0.895605173
CHORDC1	0.519879822	3.492499115	5.32E-06	2290863.452	0.854792396
CHPF	0.307465893	0.997719177	0.796445787	1.24985727	0.984152491
CHPT1	0.268479123	1.116612705	0.482057431	2.586463463	0.796897915
CHRAC1	0.110358142	1.83824279	0.449793897	7.512633182	0.39665483
CHRD	0.339362613	0.337522439	0.053772285	2.118589472	0.246494848
CHRDL1	0.641113632	3.076612089	0.339777011	27.85809999	0.317448979
CHRDL2	0.344109365	0.386568583	0.085820409	1.741255618	0.215818331
CHRFAM7A	0.164414911	0.845783623	0.427583293	1.673007219	0.630328252
CHRM1	0.618680027	0.068374384	1.23E-05	381.5199372	0.542193124
CHRM2	0.609557285	1.082554573	0.855474932	1.369910863	0.509000869
CHRM3	0.679738134	5.551176602	0.008636034	3568.25391	0.60336863
CHRM4	0.687083569	1.029269658	0.484775312	2.185334118	0.940135456
CHRM5	0.673192997	0.941108354	0.372399253	2.378320921	0.897896149
CHRNA1	0.079838352	0.676787373	0.31906368	1.435579091	0.308890079
CHRNA10	0.963879991	0.544346096	0.119155633	2.486770158	0.432663994

CHRNA2	0.224381342	0.003991022	1.29E-05	1.237249338	0.059129685
CHRNA3	0.077708515	1.600067369	0.945495322	2.707803544	0.079917923
CHRNA4	0.790399211	0.550814743	0.066926912	4.533256808	0.579215898
CHRNA5	0.820182156	1.015280673	0.811997703	1.26945537	0.894165744
CHRNA6	0.41033272	0.698857151	0.225845603	2.16254517	0.534137108
CHRNA7	0.37407482	0.587670916	0.227650241	1.517051352	0.271929252
CHRNA9	0.520466708	1.055338853	0.688949998	1.61657609	0.804482479
CHRNB1	0.795048925	1.052112857	0.306678168	3.609456356	0.935626697
CHRNB2	0.482064437	1.028187924	0.904544535	1.168732292	0.670658748
CHRNB3	0.175700448	1.9883004	0.457311659	8.644735824	0.359372046
CHRNB4	0.46204556	0.076392049	3.81E-06	1529.840599	0.610805675
CHRND	0.857757765	1.160644148	0.747302969	1.802608706	0.50719423
CHRNE	0.235721901	0.949594765	0.556126106	1.621449178	0.849731555
CHRNG	0.722194732	1.156775633	0.814947423	1.641983063	0.415115229
CHST1	0.29310011	1.030895475	0.86844923	1.223727817	0.727995735
CHST10	0.978618567	0.932430136	0.468108744	1.85731621	0.842272377
CHST11	0.814883682	1.148700464	0.803903889	1.641381231	0.446479356
CHST12	0.073112536	308.5203793	0.021916432	4343080.24	0.239570387
CHST13	0.231106851	1.489398053	0.405544978	5.469939662	0.548374304
CHST2	0.170413927	2.4355277	0.78261904	7.579415881	0.124342028
CHST3	0.090360905	1.632620192	0.821066713	3.246324141	0.162178606
CHST4	0.61071291	32.66741681	0.009310644	114617.2232	0.402539867
CHST5	0.98952985	0.975083268	0.606653908	1.567264907	0.917002274
CHST6	0.757684109	0.725170152	0.131633573	3.994966783	0.712050582
CHST7	0.76033267	360.8016846	0.513180966	253668.5189	0.078322219
CHST8	0.613360667	1.125139124	0.399602002	3.167997258	0.823351423
CHST9	0.835485045	0.942898063	0.085152265	10.44078807	0.961774784
CHSY1	0.797277923	0.881840609	0.273726151	2.840952017	0.833148564
CHTF18	0.072021329	1.263107874	0.632887568	2.520892464	0.507663281
CHUK	0.164857503	3.905168913	0.987512608	15.4431894	0.052131011
CHURC1	0.801401793	0.845010583	0.301310823	2.369788376	0.748906749
CIAPIN1	0.402643297	0.799468846	0.082639831	7.734169209	0.846734635
CIB1	0.377417195	1.125885664	0.537312376	2.359183568	0.753405261

CIB2	0.221724305	4000.7622	0.040653038	393724524.8	0.157367917
CIB3	0.233251407	1.676524355	0.494536463	5.683572645	0.406795105
CIB4	0.830912783	0.220571944	0.010216903	4.761910775	0.334888445
CIC	0.503006782	0.943570451	0.00034974	2545.674677	0.988502816
CIDEA	0.169191342	5.94848534	0.150106374	235.7293493	0.342207097
CIDEB	0.103503078	0.43743975	0.077127673	2.480997107	0.350424343
CIDEC	0.771930341	1.104266054	0.750702031	1.624350896	0.614473319
CIITA	0.573981336	1.42225947	0.565861462	3.574765445	0.453809418
CILP	0.280760191	1.468873666	0.830197331	2.598887957	0.186588051
CILP2	0.924928344	3.246784513	0.01566339	673.0094718	0.66521657
CINP	0.749757399	0.937743369	0.714811996	1.230201273	0.642572488
CIRBP	0.152162029	2.429727172	0.272772492	21.64285004	0.426232603
CIRH1A	0.524774866	1.108983149	0.473219399	2.598886743	0.811830714
CISH	0.797234029	1.121680017	0.509376596	2.470011517	0.775566237
CIT	0.466364893	1.042906014	0.121595185	8.944868625	0.969437055
CITED1	0.764686164	0.002566903	3.88E-09	1697.044173	0.383004373
CITED2	0.513272567	0.033340808	0.000345674	3.215770059	0.144591884
CITED4	0.578027837	1.010492088	0.449153007	2.273377322	0.979871739
CIZ1	0.248408266	0.317780393	0.051172715	1.973402799	0.218548915
CKAP2	0.249238137	0.98468486	0.300868142	3.222688409	0.979645862
CKAP4	0.494085534	4.886844901	0.000134717	177269.6499	0.767091433
CKAP5	0.429380796	0.880095129	0.473409205	1.636147816	0.68641586
CKB	0.689298971	0.964008597	0.721232293	1.288506608	0.804432691
CKLF	0.687898386	0.799535755	0.334886908	1.908875531	0.614349481
CKM	0.815871672	0.856171509	0.481153431	1.523484209	0.597410307
CKMT1A	0.844252412	0.341237695	0.015488	7.518283053	0.495604651
CKMT1B	0.197774918	1.074533571	0.290377098	3.976286014	0.914249435
CKMT2	0.191004938	0.558898529	0.250211612	1.248413544	0.155939872
CKS1B	0.609929745	1.240371948	0.227570371	6.760645351	0.80337569
CKS2	0.135253721	2.562811947	0.658933688	9.967626788	0.174453033
CLASP1	0.716367845	0.129039962	0.00459789	3.621511793	0.228761235
CLASP2	0.882936212	0.852016164	0.393242738	1.846013855	0.684764113
CLC	0.438794628	0.841121637	0.122900012	5.756595107	0.86004939

CLCA1	0.090360905	1.407777529	0.982731107	2.016663111	0.062184902
CLCA2	0.421759402	2.078459638	0.022736878	189.9994542	0.750809626
CLCA4	0.281480873	0.022944027	2.45E-06	214.8011056	0.418487241
CLCC1	0.249863901	0.780815969	0.50273671	1.212709488	0.270713244
CLCF1	0.590869605	0.100915291	0.003919054	2.598560036	0.166424711
CLCN1	0.751875476	0.675532401	0.235158398	1.940581445	0.466272562
CLCN2	0.980555387	1.04444999	0.772309536	1.412485191	0.777651247
CLCN3	0.419280421	0.560266928	0.097419948	3.222122756	0.516286567
CLCN4	0.49225552	0.920715654	0.584406799	1.450560324	0.721709237
CLCN5	0.508114216	0.004921627	4.80E-08	504.147937	0.366637331
CLCN6	0.843546872	1.096668931	0.676592734	1.777557878	0.708046862
CLCN7	0.642196068	0.023751139	0.000252467	2.234416954	0.106702998
CLCNKA	0.944323971	0.33858805	0.02258173	5.076753181	0.433085138
CLCNKB	0.24756478	0.888510434	0.45934021	1.718662492	0.725462054
CLDN1	0.985135097	0.99909335	0.703391922	1.419105752	0.99595799
CLDN10	0.800043364	0.933770169	0.562417501	1.55031934	0.791077592
CLDN11	0.566563374	0.71213263	0.258152994	1.9644664	0.511989366
CLDN12	0.618184325	3.260899719	0.567614563	18.73360494	0.185139827
CLDN14	0.267057456	1.09176363	0.562585469	2.118696429	0.795221973
CLDN15	0.686637763	0.936818977	0.797554021	1.100401695	0.4267228
CLDN16	0.656668775	1.067534357	0.349522093	3.260536678	0.908668911
CLDN17	0.509087713	2.49430752	0.648824299	9.588990438	0.18340942
CLDN18	0.181271428	1.22927816	0.039930512	37.84386181	0.906021687
CLDN19	0.969812679	0.906434494	0.499281767	1.645610851	0.746797385
CLDN2	0.053403504	0.402150547	0.116763411	1.385066271	0.148823986
CLDN20	0.329114412	1.173350848	0.83958085	1.639808972	0.349222954
CLDN23	0.726136795	2.021491924	0.022515265	181.4959579	0.759047862
CLDN3	0.390321089	0.949051836	0.55157287	1.632965356	0.850205676
CLDN4	0.247335225	3.171372119	0.747532158	13.45440596	0.117506209
CLDN5	0.419626473	1.563842042	0.840127649	2.910988509	0.158401926
CLDN6	0.879228956	1.014398645	0.424937405	2.421543971	0.974310666
CLDN7	0.931374302	0.11046743	0.001009967	12.08262897	0.357723124
CLDN8	0.507929158	2.073875108	0.002109538	2038.815247	0.835640538

CLDN9	0.686982232	250.0974587	0.003862155	16195296.23	0.328611586
CLDND1	0.378493037	1.575538728	0.732903196	3.386971564	0.244350012
CLEC10A	0.336653391	1.312898462	0.807074846	2.135740421	0.272820025
CLEC11A	0.077708515	1.356960673	0.824666953	2.2328314	0.229635786
CLEC12A	0.931942757	1.072678953	0.9095966	1.26500048	0.404377178
CLEC12B	0.836099371	0.697623481	0.311187337	1.563940632	0.382004322
CLEC14A	0.431762998	0.951646223	0.344954255	2.625364147	0.923739274
CLEC1A	0.223362264	0.658617145	0.371749701	1.16685109	0.152386945
CLEC1B	0.605464903	1.048679911	0.635952453	1.729263799	0.852239594
CLEC2A	0.473635192	0.959858332	0.030552575	30.15549478	0.981416537
CLEC2B	0.202296815	0.561722105	0.19750478	1.597590313	0.279486046
CLEC2D	0.382760386	2.27241369	0.783702129	6.589064628	0.13072663
CLEC2L	0.481596436	0.876703979	0.298610255	2.573956705	0.81075039
CLEC3A	0.993656516	0.829296291	0.607206439	1.132617003	0.23922034
CLEC3B	0.194087974	0.84149154	0.269388534	2.628575164	0.766493362
CLEC4A	0.917797927	0.597718638	0.020891433	17.10115209	0.763601338
CLEC4C	0.286179356	1.231401454	0.805748547	1.881914086	0.33610536
CLEC4D	0.760724651	1.155754014	0.392849425	3.40020185	0.792613394
CLEC4E	0.976040124	0.160113634	0.000150852	169.9437138	0.606330113
CLEC4F	0.677494913	0.583237208	0.017632908	19.29152277	0.762632246
CLEC5A	0.300935228	0.541777153	0.215568256	1.361622018	0.19240889
CLEC6A	0.73110013	1.453194069	0.668921336	3.156982579	0.345065022
CLEC7A	0.20595485	3.428145896	0.692192483	16.9782027	0.131227141
CLEC9A	0.806270876	0.944972771	0.730474829	1.222456275	0.666563017
CLGN	0.978919456	0.164072059	0.003166264	8.502019669	0.369529533
CLIC1	0.094729796	3.32386522	0.70167189	15.74536497	0.130145592
CLIC2	0.878120891	0.501788784	0.098572345	2.554387672	0.406256863
CLIC3	0.901059693	0.805556808	0.235253098	2.758398404	0.730622331
CLIC4	0.729091412	1.01568028	0.478611252	2.155416167	0.967672147
CLIC5	0.535570531	0.725222262	0.173296146	3.034962647	0.660017044
CLIC6	0.949446221	0.416494127	5.34E-06	32502.77157	0.878877161
CLK1	0.577327555	1.762200225	0.593158832	5.235275048	0.307811212
CLK2	0.238951828	0.671401546	0.159241863	2.830788501	0.587378751

CLK3	0.764215205	0.139675623	4.37E-06	4460.334512	0.70990062
CLK4	0.884036194	0.908254325	0.384971447	2.142823642	0.826079088
CLLU1	0.051973215	1.34800266	0.98461518	1.845503916	0.062430435
CLLU1OS	0.451623156	0.469249042	0.169641871	1.297997138	0.144971238
CLMN	0.13837665	0.101976017	0.003156942	3.294045106	0.197880244
CLN3	0.484974447	1.045119007	0.506432514	2.156800181	0.904969392
CLN5	0.985852699	0.228126836	0.006195304	8.400210377	0.421841215
CLN6	0.382780151	0.971709041	0.777625879	1.214232301	0.80069283
CLN8	0.745921213	0.688931891	0.20414394	2.324963216	0.548222696
CLNS1A	0.834335183	58.21030976	0.002321878	1459353.263	0.431654961
CLOCK	0.748102544	0.652321341	0.000553527	768.7486445	0.905749696
CLPB	0.660855631	0.748026328	0.45671985	1.225134812	0.248779152
CLPP	0.080476643	4.069234745	0.245265364	67.51328914	0.327432601
CLPS	0.881507185	1.357293312	0.758727016	2.428073725	0.303251883
CLPTM1	0.288315584	0.001528011	7.80E-07	2.994252966	0.09365807
CLPX	0.353815604	1.105624082	0.846016225	1.444894997	0.462124987
CLSPN	0.590442482	1.204162125	0.709279138	2.044338181	0.491478557
CLSTN1	0.17623513	0.574281643	0.224151967	1.471320595	0.247896237
CLSTN2	0.482926691	1.029588953	0.815260738	1.300263047	0.80656538
CLSTN3	0.388134585	0.649668075	0.326864216	1.291265874	0.218472296
CLTA	0.353623938	0.889666403	0.405637444	1.951265398	0.770477513
CLTB	0.866961674	1.313729659	0.313221667	5.51010929	0.709127548
CLTC	0.002847646	0.260082797	0.114638214	0.590056833	0.001272619
CLTCL1	0.535722864	0.356101658	0.01358007	9.337830738	0.535572393
CLU	0.112645488	0.580410685	0.22971884	1.466473376	0.249989907
CLUAP1	0.76207868	0.211117312	2.03E-05	2196.671986	0.741734921
CLUL1	0.285276602	0.371116979	0.069914988	1.969932581	0.244472933
CLYBL	0.734931884	0.384424771	0.000839855	175.9617359	0.759716522
CMA1	0.420926319	0.953667351	0.788740161	1.153081156	0.624353123
CMAS	0.119210072	1.208359324	0.649654095	2.247553375	0.550008127
CMIP	0.350712446	1.522276017	0.052373396	44.2462102	0.806905284
CMKLR1	0.07915337	3.660811677	0.530161425	25.27822945	0.188076217
CMTM1	0.350315944	1.167474864	0.419372403	3.25008882	0.766908863

CMTM2	0.758104476	0.915441098	0.191843044	4.368323111	0.911769341
CMTM3	0.71963527	2.393081789	0.727870724	7.867936244	0.150743773
CMTM4	0.540313162	1.225849208	0.73367307	2.048196047	0.436858856
CMTM5	0.729343302	1.976526458	0.816577479	4.784183912	0.13086964
CMTM6	0.458498476	1.414645218	0.901048537	2.220991446	0.131753848
CMTM7	0.558676248	1.936877529	0.340614811	11.01389146	0.455987368
CMTM8	0.709157052	1.140855951	0.367576033	3.540906327	0.819613266
CMYA5	0.893257169	1.766436422	0.291847835	10.69152229	0.535679316
CNBD1	0.905149234	0.096841288	3.74E-05	251.0580957	0.56046735
CNDP1	0.240191947	0.585867702	0.244235299	1.405370009	0.231044964
CNDP2	0.962163313	1.107907261	0.498421303	2.462692688	0.801476554
CNFN	0.152734744	0.76761259	0.360091417	1.636331945	0.493463358
CNGA1	0.967883883	2.882352763	0.000416063	19968.01558	0.814501842
CNGA2	0.339181636	0.249587888	0.020293335	3.069683435	0.278364373
CNGA3	0.444897833	1.082567292	0.809573451	1.447616569	0.592572646
CNGA4	0.937894175	0.99127678	0.092502703	10.62271285	0.994223134
CNGB1	0.937251479	0.490730438	0.162062049	1.485951604	0.207914712
CNGB3	0.44317086	0.921582164	0.554192089	1.532525821	0.752979141
CNIH2	0.683264387	1.088049561	0.818772847	1.445885573	0.560775749
CNIH3	0.675709322	1.628010686	0.258324631	10.26003126	0.603844011
CNIH4	0.745429737	0.133608062	0.002707115	6.594147923	0.311625436
CNKSR1	0.74195182	1.156000245	0.15747039	8.486272014	0.88666263
CNKSR2	0.998868638	1.01743262	0.755401539	1.370356141	0.90943731
CNKSR3	0.830364133	71.69972782	0.000861421	5967871.402	0.459827481
CNN1	0.898823648	0.712604298	0.168167492	3.019637619	0.645581666
CNN2	0.208760956	37.6470004	0.000696376	2035246.353	0.514056016
CNN3	0.952787274	0.875196985	0.269369601	2.843564232	0.824526218
CNNM1	0.342195093	1.039545319	0.066096107	16.34974452	0.977991521
CNNM2	0.905039378	0.026901559	7.13E-05	10.15365173	0.23235246
CNNM3	0.256028041	0.897332778	0.744640895	1.081334802	0.254997736
CNNM4	0.394759397	0.858243215	0.006611883	111.4026645	0.950902895
CNOT1	0.563046908	0.909101075	0.625219151	1.321880118	0.617817152
CNOT10	0.892984748	0.877852742	0.423465705	1.819806015	0.726148387

CNOT2	0.935240874	1.123706288	0.882957709	1.430097738	0.343081576
CNOT3	0.637141954	0.911150123	0.723690099	1.147168585	0.428519709
CNOT4	0.359216057	1.029318111	0.822339966	1.28839144	0.800825109
CNOT6	0.546848451	6.218937501	0.231031775	167.4020104	0.276667116
CNOT6L	0.54802697	1.096915047	0.86451305	1.391792316	0.446373352
CNOT7	0.834088448	1.29118361	0.446895718	3.730523806	0.636859442
CNOT8	0.432156217	0.323624286	0.026629474	3.932960926	0.37597701
CNP	0.515406165	20.06734224	0.037556027	10722.59913	0.349348154
CNR1	0.373116341	0.037573821	5.92E-06	238.385464	0.462593642
CNR2	0.652282103	0.011473816	1.97E-07	669.7997721	0.424938012
CNTF	0.711323225	0.429489821	0.000207804	887.6719123	0.828213251
CNTFR	0.837132548	0.747023072	0.283676854	1.967180128	0.554935904
CNTN1	0.665142531	2.89751486	0.000131176	64002.65887	0.834875651
CNTN2	0.871522887	1.072131795	0.621927656	1.848231982	0.802069919
CNTN3	0.406600447	0.176132415	0.002365207	13.11624486	0.429755123
CNTN4	0.73686704	0.719180326	0.296294072	1.745631755	0.466249413
CNTN5	0.169605134	1.077876876	0.797278441	1.457230624	0.625948443
CNTN6	0.699763416	5.152939527	0.000170866	155401.1596	0.755374831
CNTNAP1	0.801510687	0.884646893	0.058857385	13.29654936	0.929366373
CNTNAP2	0.353814193	0.001046108	7.61E-08	14.38448353	0.158076793
CNTNAP3	0.133843555	1.053330294	0.90124511	1.231079865	0.513722824
CNTNAP3B	0.732224614	1.51679954	0.007594636	302.9349644	0.877490504
CNTNAP4	0.938148446	1.203509181	0.305122402	4.747059995	0.791339759
CNTNAP5	0.973942539	1.0274721	0.075071096	14.0626549	0.983802641
CNTROB	0.387982239	0.152194049	0.020569368	1.126093354	0.065231908
COASY	0.754621864	0.841135705	0.351437773	2.013185063	0.697623703
COBL	0.29087206	1.142710412	0.412425472	3.166116489	0.79751538
COBLL1	0.853272015	1.291150523	0.520935668	3.200144996	0.581093901
COCH	0.388841403	0.168419696	0.025847371	1.097411173	0.062496173
COG1	0.278182816	1.410487955	0.940298494	2.115792255	0.096428144
COG2	0.200577214	2.04671484	0.817784807	5.122425367	0.125966302
COG3	0.381605456	2.396561445	0.487136119	11.79035292	0.282280065
COG4	0.280644257	0.754895112	0.221565848	2.571996701	0.653028574

COG5	0.363101239	0.766771913	0.525420546	1.118987772	0.168506674
COG6	0.942427642	1.638553102	0.336302749	7.983450246	0.541072711
COG7	0.789334804	2.688353976	0.734938999	9.833805404	0.135034301
COG8	0.204470732	0.84454481	0.453085858	1.574218052	0.594875503
COIL	0.785579638	4.749836517	0.193780685	116.4251581	0.339789139
COL10A1	0.202474423	1.65653553	0.550106218	4.988327481	0.36951491
COL11A1	0.722339487	0.97451396	0.429340594	2.21194425	0.950777923
COL11A2	0.926484557	0.793720288	0.376057239	1.675255333	0.544405198
COL12A1	0.348844214	0.548594324	0.175400488	1.715820382	0.30208113
COL13A1	0.462115527	0.372643862	0.159861844	0.868646605	0.022249324
COL14A1	0.114995091	0.661947118	0.227016757	1.930139401	0.449884971
COL15A1	0.775539606	8.367328021	0.045223401	1548.140496	0.425128418
COL16A1	0.530334933	1.961844557	0.580049464	6.63535493	0.278398535
COL17A1	0.704185143	1.523909002	0.655393342	3.543366244	0.327806945
COL18A1	0.107390324	0.994882537	0.849977161	1.164491597	0.949065208
COL19A1	0.82958602	0.91212715	0.389741831	2.134684739	0.832100701
COL1A1	0.655913748	0.795820864	0.202057406	3.134410461	0.744021045
COL1A2	0.680664638	0.13672888	2.25E-07	83207.77408	0.769669918
COL20A1	0.166993291	2.109583932	0.783926816	5.676989579	0.139413752
COL21A1	0.46587334	0.148997207	0.00421795	5.263259853	0.295187534
COL22A1	0.930850639	0.431345216	0.069211399	2.688266657	0.367753872
COL23A1	0.080407438	0.199446379	0.005718811	6.955791206	0.373649581
COL24A1	0.137249616	1.894238364	0.883393279	4.061768486	0.100715659
COL25A1	0.530499155	2.88358295	0.243277634	34.17926468	0.401204185
COL27A1	0.681870552	0.920054726	0.604157283	1.401126367	0.697811746
COL2A1	0.726184868	2.9146265	0.219987569	38.61603484	0.417123185
COL3A1	0.372521674	1.338676579	0.162860589	11.00361357	0.786094474
COL4A1	0.907920728	0.990648259	0.853660643	1.149618388	0.901523188
COL4A2	0.950920324	1.828735297	0.719407234	4.648650482	0.204760061
COL4A3	0.845271948	0.954708275	0.419611355	2.172171654	0.912009215
COL4A3BP	0.82472651	0.050317432	5.02E-05	50.45123172	0.396510894
COL4A4	0.415217234	0.667956998	0.026101912	17.09325197	0.807277006
COL4A5	0.705541839	1.178094251	0.820691417	1.691142414	0.374218162

COL4A6	0.262279565	0.354422957	0.026538307	4.733370323	0.432825153
COL5A1	0.25874628	0.004431373	8.89E-07	22.08221872	0.212207935
COL5A2	0.086907423	2.120010797	0.953237533	4.714927414	0.065397391
COL5A3	0.387872821	1.425328245	0.176473801	11.51196722	0.739501652
COL6A1	0.933860208	1.000818615	0.285128969	3.51292927	0.998980874
COL6A2	0.47134601	0.02130717	5.07E-06	89.5335014	0.365933703
COL6A3	0.765947131	6.990993449	0.008651749	5649.029682	0.569137842
COL7A1	0.769440077	0.952208086	0.682452485	1.328591015	0.773225789
COL8A1	0.070751629	7.73E-05	2.22E-09	2.68833498	0.075963214
COL8A2	0.948587288	1.022170339	0.024778155	42.16747358	0.990781291
COL9A1	0.386064148	22.11735231	0.012737749	38403.74708	0.415899428
COL9A2	0.548116935	1.507867992	0.313295788	7.257250083	0.608453702
COL9A3	0.849189796	7.097902332	4.07E-05	1237071.24	0.750273356
COLEC10	0.526992573	0.67811578	0.187326678	2.454754527	0.553987856
COLEC11	0.076611884	0.581241944	0.250528591	1.348517535	0.20636721
COLEC12	0.92963931	0.943855409	0.70265965	1.267844302	0.70114754
COLQ	0.616964004	1.23376828	0.86744161	1.754797269	0.242494904
COMMD1	0.452807543	0.242089068	0.052532881	1.115627304	0.06881951
COMMD10	0.788407539	3.220871386	0.129528032	80.09086786	0.475606413
COMMD2	0.829238472	1.650568206	0.002297304	1185.901364	0.881291778
COMMD3	0.434531836	0.841256074	0.507860227	1.393516846	0.502030095
COMMD4	0.320905908	1.023008809	0.428484639	2.442437667	0.959139845
COMMD5	0.250312078	0.707708311	0.3864772	1.29593946	0.262677912
COMMD6	0.224590734	0.762180935	0.206084103	2.818848071	0.684033354
COMMD7	0.338838751	1.180897974	0.81401881	1.713129977	0.381059015
COMMD8	0.648602255	1.407378241	0.389395041	5.086642884	0.602177968
COMMD9	0.891317231	1.624239796	0.059955948	44.00155437	0.773231796
COMP	0.959610784	1.042694366	0.833919812	1.303736315	0.713802713
COMT	0.474580953	54.96864043	0.008652694	349203.5531	0.369816539
COMTD1	0.565697412	0.798762667	0.187591402	3.401124957	0.761152821
COPA	0.344365848	0.478743605	0.001283734	178.5381162	0.807378574
COPB2	0.742150899	1.029448601	0.843719614	1.256062328	0.774945226
COPE	0.818148674	1.564541171	0.541752931	4.518275651	0.408129866

COPG2	0.786351902	0.090389145	0.00133307	6.128857402	0.263888464
COPS2	0.053694039	0.938425035	0.829995574	1.06101957	0.310354834
COPS3	0.560537714	0.016282455	4.83E-06	54.87046037	0.320428323
COPS4	0.751402775	1.766150834	0.158986001	19.61989574	0.643350061
COPS5	0.932969789	0.785215685	0.038209868	16.13624178	0.875421264
COPS6	0.774573005	0.913626406	0.679983155	1.227549836	0.548871276
COPS7A	0.863937264	0.0044972	5.35E-06	3.780632459	0.11574115
COPS7B	0.962297133	1.192681615	0.164960939	8.623189459	0.861413272
COPS8	0.692414192	48.49999951	0.000940213	2501825.668	0.483234174
COPZ1	0.47972883	270.047529	0.13275838	549311.224	0.149741685
COPZ2	0.778783591	1.032654671	0.269695047	3.95400539	0.962586137
COQ10A	0.791538723	1.072867869	0.734876957	1.566310458	0.715617994
COQ10B	0.377769213	0.713104571	0.130231356	3.904728825	0.696713086
COQ2	0.377923676	1.224739236	0.625189335	2.399251094	0.554586059
COQ3	0.988518897	1.08839408	0.012335266	96.03373538	0.970439527
COQ4	0.460384171	0.009869029	2.16E-06	45.03111972	0.282684554
COQ5	0.371299636	1.243157768	0.376124467	4.108855899	0.721213582
COQ6	0.878688802	0.66430596	0.054328026	8.122923699	0.748827591
COQ7	0.277643698	0.816546912	0.219450804	3.038261187	0.762412527
COQ9	0.069262951	1.133609387	0.23383881	5.495538767	0.876261764
CORIN	0.371597887	0.390627648	0.101378081	1.505157303	0.171991891
CORO1A	0.380441463	8.224436424	0.491019037	137.7570916	0.142830969
CORO1B	0.606880655	0.004530202	1.21E-05	1.699861786	0.074336536
CORO1C	0.97391592	0.941498342	0.394790992	2.245287114	0.891864835
CORO2A	0.936515611	0.907996496	0.447860642	1.84087986	0.788967034
CORO2B	0.512350741	0.356259354	0.0405213	3.132197833	0.352083316
CORO6	0.788004132	799.6987283	0.008241226	77599869.73	0.253908407
CORO7	0.181104038	0.000141634	2.51E-11	797.8214491	0.263804427
CORT	0.01449994	1.76460989	1.347616492	2.310633687	3.64E-05
COTL1	0.316548393	0.940637954	0.743568599	1.189936963	0.609919702
COX10	0.303182472	1.047902819	0.502130137	2.186883909	0.900795237
COX11	0.208298512	2.098043011	0.076409059	57.60814897	0.661080283
COX15	0.320717759	1.465672881	0.023014176	93.34233806	0.85684833

COX17	0.748201661	0.57651736	0.142228827	2.336883976	0.440544864
COX4I1	0.481167747	1.031936537	0.528937741	2.013267223	0.926543348
COX4I2	0.674184713	0.704194175	0.094178244	5.265435155	0.732611328
COX5A	0.607009696	1.242443247	0.831360975	1.856792981	0.28960759
COX5B	0.708404723	1.012373671	0.774805961	1.322783383	0.928187681
COX6A1	0.421246399	0.555249029	0.137288227	2.245651291	0.409240888
COX6A2	0.509259481	0.938613145	0.580750749	1.516992679	0.795914555
COX6B1	0.615990147	0.933729787	0.736762563	1.183354528	0.570551483
COX6B2	0.155585131	2.437853644	0.867840416	6.848183463	0.09083981
COX6C	0.83212384	0.169783732	6.10E-05	472.8012281	0.661268189
COX7A1	0.123801289	0.679261356	0.007899849	58.40567431	0.864867631
COX7A2	0.466415022	1.447287916	0.125949712	16.63078285	0.766642456
COX7A2L	0.443698907	1.231539002	0.243419675	6.230754829	0.801212491
COX7B	0.434226619	5.962101114	0.53393733	66.57457289	0.146982253
COX7B2	0.294424284	4.300647237	0.001698458	10889.62332	0.71523563
COX7C	0.276979269	0.342794924	0.021112866	5.565722706	0.451539765
COX8A	0.33127893	0.778593204	0.26638327	2.275696135	0.647431859
COX8C	0.13860937	1.256575159	0.477466851	3.306996347	0.643650463
CP	0.618440542	0.002649883	3.53E-06	1.990264645	0.079047949
CPA1	0.957704676	0.656735155	0.100275592	4.301156984	0.661017668
CPA2	0.141808085	1.090951178	0.792883463	1.501071127	0.592908222
CPA3	0.109529581	0.024747956	0.000528536	1.1587889	0.059447739
CPA4	0.460172172	0.695507889	0.00233657	207.0262144	0.900566156
CPA5	0.497878188	1.872236074	0.129672085	27.03178494	0.645243564
CPA6	0.58887784	1.181122	0.845369567	1.650224036	0.329293344
CPAMD8	0.801409643	1.107451726	0.831584061	1.474835055	0.485022373
CPB1	0.982335927	0.891078887	0.031917405	24.87738497	0.945872561
CPB2	0.267631915	0.060984945	0.002888535	1.287560428	0.072250258
CPD	0.989998125	0.106616355	0.000409397	27.76533659	0.430242801
CPE	0.002543812	1.563003882	1.198971121	2.03756462	0.000962212
CPEB1	0.546105188	0.996291505	0.044031033	22.54311801	0.998137242
CPEB2	0.385904815	8.611715718	0.105227628	704.7735371	0.33802941
CPEB3	0.502681845	1.208260973	0.577203477	2.529254651	0.615723346

CPEB4	0.97736085	1.55144602	0.558591998	4.309021184	0.399421563
CPLX1	0.199507936	0.51917478	0.187072873	1.440842002	0.208146932
CPLX2	0.855887666	0.882310912	0.148910672	5.227782098	0.890294031
CPLX3	0.637371854	0.896104191	0.749334587	1.071621057	0.229358712
CPLX4	0.993261018	0.400024565	0.063059985	2.537578332	0.33103298
CPM	0.056978733	3.345576396	0.177727316	62.97783423	0.420006562
CPN1	0.365541583	0.704621591	0.323891307	1.532895683	0.377334969
CPN2	0.428328553	1.907693135	0.265773261	13.69322516	0.520693909
CPNE1	0.957461034	1.107244215	0.290817368	4.215668966	0.881278208
CPNE2	0.312782262	1.520544536	0.862437418	2.680838791	0.147491539
CPNE3	0.712240725	0.276789829	0.020254074	3.782577829	0.335659553
CPNE4	0.595023371	1.575557549	0.590171112	4.206206535	0.364198293
CPNE5	0.152665177	4.112160908	0.230233801	73.44650214	0.33636006
CPNE6	0.838791361	0.634485622	1.61E-06	249402.2143	0.944815018
CPNE7	0.562355743	3.183763066	0.899690587	11.2664814	0.072490022
CPNE8	0.849234894	1.175287107	0.472040586	2.926230972	0.728571347
CPNE9	0.252182037	1.090543803	0.885602603	1.34291135	0.414441323
CPO	0.989821233	0.974915535	0.773457351	1.228846425	0.829688077
CPOX	0.26459158	6.081463317	0.506690394	72.99170564	0.154512554
CPS1	0.119626378	0.124772958	0.005348657	2.910691822	0.1952768
CPSF1	0.357968652	0.952847916	0.371789405	2.442025348	0.91987748
CPSF2	0.129968213	0.773657676	0.336647846	1.777959393	0.545529139
CPSF3	0.232376945	1.292976729	0.671710722	2.488852368	0.441885612
CPSF3L	0.681960494	0.06885927	3.99E-05	118.9400189	0.481731675
CPSF4	0.655121736	1.133336209	0.571029577	2.24935978	0.720432398
CPSF6	0.05091551	83606.64549	0.362962089	19258405704	0.072004112
CPT1A	0.826586798	0.680289136	0.175801554	2.632475639	0.576850979
CPT1B	0.784647732	1.250342393	0.556810845	2.807696928	0.588294826
CPT1C	0.712409964	1.136688555	0.675269498	1.913400315	0.629667458
CPT2	0.746202555	0.336990925	0.004378144	25.93858759	0.623552362
CPVL	0.419635705	1.303348162	0.536894859	3.163964796	0.558217519
CPXCR1	0.160938861	0.832040282	0.384077539	1.802477262	0.641074498
CPXM2	0.763415702	0.984846237	0.729262999	1.330003184	0.920653331

CPZ	0.857166225	0.722019749	0.115900031	4.49794975	0.727117424
CR1	0.764273144	0.090768336	1.20E-05	687.3581836	0.598543245
CR2	0.90364252	1.267733794	0.430996986	3.728910007	0.66649227
CRABP1	0.640349182	0.980471536	0.006919339	138.9329871	0.993774182
CRABP2	0.615896487	1.114346663	0.119569796	10.38530232	0.924261373
CRADD	0.344277137	0.39152206	0.111719697	1.372090391	0.142768037
CRAMP1L	0.644765805	2.410220475	0.572913447	10.13968649	0.230104359
CRAT	0.536544901	0.92742486	0.284165883	3.026812587	0.900648143
CRB1	0.476644811	0.955401192	0.475053759	1.92144872	0.898163438
CRB2	0.545439405	1.635674758	0.474810211	5.634739629	0.435565836
CRB3	0.408518092	5.70690564	0.002412889	13497.83454	0.660362594
CRBN	0.876314317	1.185267881	0.481187723	2.919567314	0.711721321
CREB1	0.638147193	1.070128202	0.124339176	9.210084891	0.950789601
CREB3	0.673361058	1.038179174	0.901193258	1.19598764	0.603779759
CREB3L1	0.248299055	2.952892642	0.271191969	32.15277716	0.374106016
CREB3L2	0.552101615	0.693962662	0.310431651	1.551337228	0.37341037
CREB3L3	0.937489205	1.352128114	0.790973967	2.311391414	0.270119551
CREB3L4	0.555758762	0.4098967	0.077456319	2.169161998	0.294134007
CREB5	0.524915294	0.623287238	0.313176165	1.240474292	0.178210938
CREBBP	0.65461344	1211.175421	0.00226245	648388180.4	0.291483434
CREBL2	0.524986317	10.22844939	0.096585528	1083.197238	0.328357529
CREG1	0.751030745	0.360012904	0.026443606	4.901347051	0.443173478
CREG2	0.092249999	1.439167786	0.982921561	2.107191457	0.061287337
CRELD1	0.664896819	0.83069593	0.465304945	1.483018254	0.530471878
CRELD2	0.154792876	13.56728866	0.372219581	494.5234771	0.155227674
CREM	0.322323766	0.756766357	0.333894946	1.715196134	0.504393445
CRH	0.173071184	0.317732638	0.056200129	1.796330921	0.194550713
CRHBP	0.949836161	0.895865431	0.653153672	1.228768825	0.495177469
CRHR1	0.965617466	1.493427655	0.301322071	7.401801515	0.623350657
CRHR2	0.335003121	0.79688017	0.363235776	1.748225386	0.571106047
CRIM1	0.697992521	1.06946189	0.782099085	1.462408994	0.674036786
CRIP1	0.336558077	0.251635905	0.005640028	11.22700596	0.476454915
CRIP2	0.566887948	0.176572418	0.002370879	13.15031835	0.430428819

CRIP3	0.394541143	0.872050831	0.656977554	1.157532165	0.343376999
CRIPT	0.372606043	653267.3085	0.505092384	8.44911E+11	0.062204095
CRISP1	0.198249432	0.791206566	0.416989889	1.501254219	0.473586358
CRISP2	0.074680387	0.000650885	3.60E-07	1.176231447	0.055168931
CRISP3	0.764032014	0.655379924	0.289400094	1.484183501	0.31098286
CRISPLD1	0.635213188	0.873599013	0.400404172	1.906012196	0.73423553
CRISPLD2	0.731568981	1.267411869	0.611341528	2.627553946	0.524085047
CRK	0.190978285	0.027908389	0.000319095	2.440899768	0.116696442
CRKL	0.655743648	0.951053949	0.007508187	120.4689779	0.983791569
CRLF1	0.121399399	0.624841334	0.189465147	2.060678174	0.43988323
CRLF2	0.765120512	0.787677384	0.238159691	2.605124563	0.695744982
CRLF3	0.324288186	0.865950747	0.627422301	1.195161049	0.381303687
CRMP1	0.432840036	1.05674987	0.19966249	5.593039983	0.948233706
CRNKL1	0.506588021	0.975053632	0.599488208	1.585902064	0.918920296
CRNN	0.984131894	0.79494787	0.287162279	2.200644596	0.65869331
CROCC	0.102712587	1.521310359	0.837575831	2.763194833	0.168237274
CROT	0.350271278	1.925379279	0.786908284	4.710949729	0.15127836
CRP	0.647144645	0.981259071	0.768958334	1.25217365	0.879114013
CRTAC1	0.862124476	9.399473801	0.000170819	517213.6576	0.687444936
CRTAM	0.20605094	1.08794789	0.67503892	1.753425732	0.729227004
CRTAP	0.881272393	1.089081856	0.838402526	1.414713401	0.52258353
CRTC1	0.354734139	0.800723318	0.26898741	2.383597917	0.689668608
CRTC2	0.464099468	1.123298048	0.830687467	1.51898103	0.450156996
CRX	0.430282948	0.527586872	0.000243053	1145.216574	0.870417194
CRY1	0.246543774	0.103164859	0.000397404	26.78130122	0.423230455
CRY2	0.947545839	0.925886569	0.428143425	2.002286823	0.844863207
CRYAA	0.540169222	1.212069759	0.672555445	2.184374703	0.522174064
CRYAB	0.384815919	19.46074492	0.058794409	6441.438915	0.315990469
CRYBA1	0.94772011	64.90904767	0.023076054	182578.1992	0.303086908
CRYBA2	0.192272943	0.404431427	0.064607379	2.531673346	0.333359783
CRYBA4	0.452137362	1.842572281	0.276910151	12.2605567	0.527361162
CRYBB1	0.851925024	0.725792112	0.253131272	2.081031649	0.550951835
CRYBB2	0.64552859	1.826270886	0.032982093	101.123521	0.768700049

CRYBB3	0.375406353	1.034018373	0.71782099	1.489499486	0.857436471
CRYGA	0.616153175	0.806935472	0.331191255	1.96606899	0.636849484
CRYGB	0.581245278	0.182495821	0.002464136	13.51578188	0.438659969
CRYGC	0.417549823	1.71975856	0.106488152	27.77369551	0.702468551
CRYGD	0.388209257	0.614742709	0.246684707	1.531949845	0.296305822
CRYGN	0.185710074	1.114822497	0.894287024	1.389743077	0.333795935
CRYGS	0.682609013	12.12680968	0.029085992	5056.025331	0.417533717
CRYL1	0.723353236	0.004940072	7.02E-09	3475.195824	0.439494188
CRYM	0.352994192	1.132805436	0.91946247	1.395650392	0.241488248
CRYZ	0.81745056	0.496486665	0.112046677	2.199967146	0.356585669
CRYZL1	0.423960426	132.9286058	0.198935365	88822.89104	0.140643353
CS	0.132480994	0.657448927	0.232772587	1.856915791	0.428558323
CSAD	0.696164549	1.677976141	0.086560952	32.52741401	0.732199252
CSAG1	0.711091318	0.000122617	5.94E-10	25.29076831	0.149147497
CSAG2	0.074924172	0.607100426	0.15260989	2.415118228	0.478707509
CSDC2	0.959525737	0.718165787	0.24855707	2.075024854	0.54084557
CSDE1	0.723112152	0.933064934	0.532900404	1.633720233	0.808457432
CSE1L	0.514936213	0.886889218	0.598544569	1.314141882	0.549636917
CSF1	0.193420164	0.377936447	0.001865105	76.58331548	0.719551906
CSF1R	0.728715014	0.746329167	0.174460915	3.192733597	0.69317605
CSF2	0.623323928	1.062704808	0.884641626	1.276609054	0.515705152
CSF2RA	0.220145223	0.007941116	6.77E-06	9.308583918	0.179854368
CSF2RB	0.485385551	0.310972246	0.024838892	3.893238863	0.365017201
CSF3	0.886499843	0.559485635	2.57E-05	12158.10965	0.909256473
CSF3R	0.100278354	0.065676768	0.001421751	3.033890225	0.163790782
CSH1	0.738330354	3.434398897	0.427616289	27.58336407	0.245741059
CSH2	0.220061366	0.71898886	0.290235166	1.781124556	0.475975701
CSHL1	0.079034277	0.547301641	0.283371732	1.05705352	0.072693232
CSK	0.113437925	0.009149542	3.69E-05	2.268893399	0.095174864
CSMD1	0.585100019	1.618056597	0.001334053	1962.521705	0.894328795
CSMD2	0.96932401	0.581394143	0.202987397	1.665222345	0.31243507
CSMD3	0.26736076	31.86514584	0.000299549	3389719.919	0.557780314
CSN1S1	0.331940997	0.330619461	0.065250924	1.675213486	0.181287822

CSN2	0.845469169	1.5974688	0.482820554	5.285414104	0.442908562
CSN3	0.219024516	2.218463667	0.329175493	14.95123772	0.413057508
CSNK1A1	0.857032104	1.10797825	0.841014786	1.45968397	0.46601089
CSNK1A1L	0.092816708	4.495290575	0.586888456	34.43181943	0.147915571
CSNK1D	0.695282508	1.084305635	0.846801927	1.388422337	0.521087073
CSNK1E	0.200005882	0.378063162	0.094641015	1.510251702	0.168659392
CSNK1G1	0.81480308	0.118798377	0.001043133	13.52948364	0.377900171
CSNK1G2	0.371919966	0.871288582	0.149751016	5.069373224	0.878122902
CSNK1G3	0.636583188	0.878774871	0.612311102	1.261197569	0.483274885
CSNK2A1	0.392051541	1.236889675	0.721082238	2.121666556	0.439987757
CSNK2A2	0.059093174	0.861748689	0.700084152	1.060745055	0.160423904
CSNK2B	0.633382869	0.097783153	0.003697505	2.585944849	0.164108199
CSPG4	0.718569011	0.522938768	4.10E-06	66630.16092	0.913923837
CSPG5	0.056547296	0.001177316	2.15E-07	6.442871199	0.124598105
CSPP1	0.247533212	162.0416698	0.005111462	5136984.515	0.335966098
CSRP1	0.074340085	8.112944164	0.082102078	801.6832763	0.371701769
CSRP2	0.325219394	1.09436582	0.798458213	1.499936412	0.575044357
CSRP2BP	0.570740602	0.540929502	0.117167711	2.497315365	0.431101399
CSRP3	0.959016484	0.299447151	0.000610808	146.8032036	0.702832238
CST1	0.150046394	0.527566062	0.053046674	5.246812496	0.585322584
CST11	0.647634558	1.041597707	0.666941981	1.626716887	0.857796362
CST2	0.428947871	1.654723617	0.007377382	371.1493351	0.855300912
CST3	0.266312424	0.786379918	0.011883682	52.03718669	0.910544525
CST4	0.407031363	5.347638965	0.031139779	918.3508622	0.523083636
CST5	0.127585537	0.60961703	0.018883456	19.68034433	0.780104377
CST6	0.927351304	1.053774312	0.803978329	1.381181882	0.704367137
CST7	0.827841015	1.110641512	0.85740176	1.438677438	0.42675052
CST8	0.162006516	0.223814307	0.012212919	4.101627561	0.313065613
CST9	0.424107978	0.783898604	0.369324669	1.663839632	0.526035084
CST9L	0.277024104	0.106800244	0.00243057	4.692847125	0.246485004
CSTA	0.611487693	0.60739485	0.138716451	2.659587253	0.50815143
CSTB	0.092579199	3.917610552	0.901969727	17.01572901	0.068413287
CSTF1	0.992000488	0.169387224	0.001405607	20.41255611	0.467677354

CSTF2	0.50231539	0.948355143	0.606673296	1.482474148	0.816040596
CSTF2T	0.675462459	1.060579751	0.867934799	1.295983765	0.565239426
CSTF3	0.099427518	0.694021689	0.326085973	1.477113841	0.343253113
CSTL1	0.143905774	1.987210807	0.93473084	4.224752864	0.07433192
CTAG1A	0.38529781	1.277036695	0.589893037	2.764607512	0.534888604
CTAG1B	0.869625893	1.554255638	0.588085553	4.107753668	0.373816875
CTAG2	0.654094094	0.564985893	0.268944029	1.186897735	0.13166983
CTAGE1	0.589466063	1.115373109	0.403936494	3.079833565	0.833120379
CTAGE5	0.17467111	0.469596194	0.196920424	1.119846184	0.088252064
CTAGE6	0.93278876	0.937346281	0.371812901	2.363064994	0.890914644
CTBP1	0.330401717	1.211585121	0.475023543	3.09024369	0.687861567
CTBP2	0.393240431	0.874806989	0.00069276	1104.693413	0.970716209
CTBS	0.603794492	1.456573043	0.629720125	3.369123752	0.379390831
CTCF	0.458884157	0.952788241	0.738367243	1.229476852	0.710047477
CTCFL	0.854450756	1.206004874	0.474281598	3.066633324	0.694040135
CTDP1	0.334218369	0.671455801	0.253388689	1.779293678	0.423087586
CTDSP1	0.476675456	0.127450772	0.005057966	3.211508034	0.210833742
CTDSP2	0.730544507	1.744369791	0.259247041	11.73716756	0.567297498
CTDSPL	0.763859778	0.882713775	0.079320426	9.82324039	0.919170324
CTDSPL2	0.853765856	0.960841145	0.711803948	1.297008409	0.794114694
CTF1	0.186346441	0.589881835	0.280336558	1.241224408	0.164337457
CTGF	0.605442108	0.786211772	0.121878873	5.071666105	0.800356863
CTH	0.849234015	4.383667254	0.001919073	10013.44979	0.708003984
CTHRC1	0.507892406	0.940508123	0.13994545	6.320716586	0.949687584
CTLA4	0.901223996	0.849524039	0.209804015	3.439834515	0.819217956
CTNNA1	0.621650776	1.166396414	0.149124384	9.123126326	0.8833959
CTNNA2	0.311403263	1.090982951	0.946773742	1.257157595	0.228655818
CTNNA3	0.240944423	1.219208237	0.900718587	1.650314258	0.199466826
CTNNAL1	0.184510419	0.397310243	0.010220849	15.44445361	0.621125064
CTNNB1	0.13323021	0.893838039	0.51371631	1.555228875	0.691249901
CTNNBIP1	0.00542909	0.159994864	0.073127071	0.350053079	4.48E-06
CTNNBL1	0.956324275	1.25569083	0.303637638	5.192898579	0.753253655
CTNND1	0.684409466	0.98124671	0.347562562	2.770278534	0.971481235

CTNND2	0.136725738	0.018065651	8.96E-05	3.641356495	0.138182277
CTNS	0.221866172	12.3266098	0.071885905	2113.701002	0.338593246
CTPS2	0.575092287	0.825997957	0.528758871	1.290328472	0.400930603
CTRB1	0.645010028	7.19573706	0.013723516	3772.985974	0.536789196
CTRB2	0.782472054	0.625195126	0.137863153	2.835195169	0.542572315
CTRC	0.221097349	1.319538406	0.570190146	3.053685896	0.517179955
CTRL	0.959580003	0.003287352	2.76E-07	39.18051447	0.232489427
CTSB	0.55435285	4.312099109	0.000456833	40702.4046	0.754316299
CTSC	0.761723164	4.449789954	0.000196806	100609.9999	0.77041551
CTSD	0.926029453	0.095963262	0.00529847	1.738038897	0.112753427
CTSE	0.773035305	0.975507436	0.835961179	1.138348025	0.752890107
CTSF	0.38413584	0.453590229	0.161401822	1.27473218	0.133733403
CTSG	0.414810502	0.890915052	0.369652147	2.147233923	0.796908052
CTSH	0.363430108	2.174130588	0.775560046	6.094748998	0.139760207
CTSK	0.681480617	0.627172733	0.187580868	2.096938998	0.448712218
CTSL	0.691532902	0.680306101	0.210958792	2.193871074	0.519046256
CTSO	0.650312235	1.448822401	0.325754133	6.443775041	0.626316372
CTSS	0.838433725	0.054344668	2.70E-07	10925.59843	0.640174621
CTSW	0.494028701	1.017781343	0.589611188	1.756884679	0.949545091
CTSZ	0.462991401	0.303839351	0.044180808	2.089557781	0.225943401
CTTN	0.114499069	2.831034657	0.5706782	14.04426739	0.202836394
CTTNBP2	0.926034471	1.263768977	0.473443218	3.373397198	0.640271183
CTTNBP2NL	0.709774665	2.322177677	0.453253213	11.8973435	0.312163338
CTXN1	0.178667916	0.051588288	6.45E-05	41.24843584	0.384701824
CUBN	0.308125219	1.117591458	0.294679598	4.238537982	0.870155318
CUEDC1	0.82078893	1.112389731	0.184797187	6.696048418	0.907415821
CUEDC2	0.2082319	0.774197711	0.550867126	1.088070185	0.140514937
CUL1	0.822619181	0.965643911	0.687618346	1.356083893	0.840079629
CUL2	0.137250735	0.052611652	0.000400527	6.910866795	0.236714442
CUL3	0.501057106	0.986737694	0.700369941	1.390195696	0.939152002
CUL4A	0.367927231	0.353593308	0.069289242	1.804439252	0.211238636
CUL4B	0.109230494	1.495806573	0.961222448	2.327699804	0.074314057
CUL5	0.536725461	0.486268905	0.109885475	2.151853528	0.342056099

CUL7	0.068270297	0.678700525	0.430102249	1.070988126	0.095854943
CUTA	0.45100722	1.136462606	0.922168721	1.400554177	0.230171962
CUTC	0.710928961	0.889990495	0.344919107	2.296431443	0.809572364
CUZD1	0.764103073	1.085726351	0.593970036	1.984614774	0.789267992
CWF19L1	0.663900203	1.570771141	0.574637591	4.29370096	0.378782863
CWF19L2	0.532624135	0.001053706	9.49E-08	11.70452002	0.149193747
CX3CL1	0.967142787	0.603977448	0.213624365	1.70761775	0.341673338
CX3CR1	0.501954134	3.111170311	8.18E-05	118313.4329	0.832936747
CXADR	0.397534042	0.549777111	0.273943711	1.103346639	0.092327312
CXCL1	0.286264387	1.113198871	0.466896532	2.654146349	0.808858776
CXCL10	0.7965452	0.71565752	0.305172466	1.678282748	0.441701617
CXCL11	0.175444884	1.106905376	0.634684859	1.930469105	0.72040763
CXCL12	0.652320912	0.83181923	0.355284272	1.947520013	0.671383614
CXCL13	0.924040581	0.994632487	0.205885826	4.805060187	0.994656431
CXCL14	0.425174687	16911.85049	0.013137964	21769788352	0.174974258
CXCL16	0.790418956	0.855730688	0.359790996	2.035278865	0.724511679
CXCL2	0.30456461	0.882393159	0.371698261	2.094757412	0.776683223
CXCL3	0.329513979	339.5663957	1.99E-05	5799138306	0.492794349
CXCL5	0.68905106	2.137994724	0.001877158	2435.07577	0.832407758
CXCL6	0.701470918	0.720058552	0.275847951	1.879601848	0.502297649
CXCL9	0.66323979	1.304656546	0.327414869	5.198690905	0.706150808
CXCR3	0.809270499	0.86628879	0.401316713	1.869985085	0.714653906
CXCR4	0.411494713	1.391045351	3.19E-05	60579.82813	0.951708476
CXCR6	0.056299802	672.2799875	0.012079055	37416864.4	0.242880517
CXorf21	0.514398894	0.364036734	0.035842411	3.697372454	0.392897646
CXorf22	0.91033867	0.006314548	9.15E-06	4.359563627	0.128881705
CXorf23	0.249870719	0.810544319	0.41430192	1.585756815	0.539582858
CXorf36	0.685695323	1.088101463	0.427937161	2.766679087	0.859247784
CXorf38	0.305425054	2.048994054	0.074740144	56.17298005	0.671108333
CXorf40A	0.076552176	20.70091474	0.247009419	1734.864493	0.179890245
CXorf40B	0.467181602	11.88928631	0.714954841	197.7119682	0.084342607
CXorf56	0.466146082	1.158141241	0.345399582	3.883302716	0.812005616
CXXC1	0.994788179	0.532271186	0.214695398	1.319602646	0.173422878

CXXC4	0.317272282	0.990318663	0.81559861	1.202467784	0.921747031
CXXC5	0.907048316	0.865680325	0.267320392	2.803386673	0.809876319
CYB561	0.573275157	1.371442673	0.610296889	3.081868906	0.444506381
CYB561D1	0.127455755	0.658468165	0.081512709	5.319174506	0.695058227
CYB561D2	0.316293927	0.366643983	0.086528965	1.553558515	0.173209035
CYB5A	0.961658881	22.28535415	0.006566948	75626.76471	0.454265581
CYB5D1	0.325183639	4.161411024	0.716163849	24.18069795	0.112258266
CYB5D2	0.820845013	0.963211546	0.856163013	1.083644667	0.532912253
CYB5R1	0.536935045	0.797662083	0.546615364	1.16400826	0.2410439
CYB5R2	0.397329732	0.743229865	0.423428156	1.30456755	0.301245454
CYB5R3	0.368182838	1.6148864	0.653689469	3.989444849	0.298967886
CYB5R4	0.940189853	0.4190896	3.49E-06	50267.16196	0.884118136
CYBA	0.786553638	1.248250098	0.543817318	2.865168603	0.600928201
CYBB	0.70103803	2.23087627	6.48E-05	76774.9094	0.88033199
CYBRD1	0.897086498	1.007095874	0.710445093	1.42761504	0.968318935
CYC1	0.310393034	1.337035287	0.322002191	5.551711782	0.6892496
CYCS	0.570637082	1.315585877	0.767421983	2.255299221	0.3185849
CYFIP1	0.349057729	0.463181068	0.012762923	16.80936993	0.674485394
CYFIP2	0.932648466	0.503626667	0.038378026	6.608985556	0.601516788
CYGB	0.263713474	1.327029026	0.337745813	5.213998123	0.685287574
CYHR1	0.474904887	0.787918688	0.165319878	3.755240243	0.764800553
CYLC1	0.53023809	0.562010954	0.154099804	2.049686664	0.382745293
CYLC2	0.725189686	5.166143527	0.589830757	45.24863889	0.138033967
CYLD	0.524079128	1.137055611	0.80379209	1.60849488	0.467973233
CYP11A1	0.78667306	0.998174694	0.838478505	1.188286539	0.983613188
CYP11B1	0.14122894	4.517587455	0.308346053	66.18731193	0.27090695
CYP11B2	0.313481763	1.471660709	0.505290533	4.286217733	0.478682683
CYP17A1	0.601290403	1.428529469	0.928611479	2.197578308	0.104604556
CYP19A1	0.97012889	0.004083916	1.66E-06	10.04295901	0.16732163
CYP1A1	0.723663327	29.28435018	0.009126804	93962.04275	0.412318037
CYP1A2	0.587071526	1.20243873	0.026485063	54.59148529	0.924554679
CYP1B1	0.388900716	1.269665598	0.136505211	11.80944464	0.833801468
CYP20A1	0.40759614	0.571092482	0.210272196	1.551068709	0.271804777

CYP21A2	0.290626545	1.864874304	0.901093157	3.859485714	0.093089688
CYP24A1	0.908121261	0.687647146	0.126183517	3.747388003	0.665101304
CYP26A1	0.791066252	1.078983755	0.843660836	1.379945463	0.544771245
CYP26B1	0.93538198	1.021668013	0.825701172	1.264144419	0.843599537
CYP26C1	0.530370755	0.48964671	0.121310395	1.976367334	0.315848375
CYP27A1	0.590225511	0.320946943	0.068269196	1.508834829	0.150123087
CYP27B1	0.400101628	0.124576873	2.67E-06	5817.038138	0.704169656
CYP2A13	0.744209096	0.000440158	1.98E-08	9.792343544	0.130222736
CYP2A6	0.746501351	0.932719907	0.754707283	1.152720326	0.519180354
CYP2A7	0.117861461	777.8289869	0.529657369	1142281.725	0.073591215
CYP2B6	0.631148278	1.504857712	0.6062175	3.735617549	0.378308408
CYP2C18	0.942932476	0.588797804	0.005662116	61.22849494	0.823122726
CYP2C19	0.189041808	0.629634152	0.093696056	4.231119011	0.634114791
CYP2C8	0.007170617	2.642643692	1.163316098	6.003153994	0.020269688
CYP2C9	0.132994806	51.04490568	0.218317339	11934.83947	0.157615994
CYP2D6	0.632597373	1.009128211	0.84682417	1.202539775	0.91909978
CYP2E1	0.935201136	0.114970388	3.10E-05	426.796283	0.605994089
CYP2F1	0.145658351	0.23446592	0.05390962	1.019748746	0.05312672
CYP2J2	0.41924191	0.49004967	0.062178231	3.862262954	0.498321841
CYP2R1	0.903124727	1.30957714	0.168017661	10.20721439	0.796844445
CYP2S1	0.062195327	1.166139525	0.266251269	5.107511395	0.838388822
CYP2U1	0.139909937	0.406742182	0.142634123	1.15988516	0.09246272
CYP2W1	0.819542237	1.411022573	0.126895289	15.68998124	0.779348127
CYP39A1	0.126494975	1.438606644	0.839292727	2.465872764	0.185919356
CYP3A4	0.351608961	0.074965558	4.57E-06	1230.185712	0.600853997
CYP3A43	0.313682745	0.28893428	0.028547142	2.924391417	0.29311656
CYP3A5	0.92335585	0.909757993	0.153364137	5.396695878	0.917076175
CYP3A7	0.22637439	11.88712075	0.262937649	537.4036029	0.203015568
CYP46A1	0.871873156	0.622276535	3.56E-05	10889.23693	0.924184025
CYP4A11	0.078753861	1.965088303	0.651022728	5.931547193	0.230726585
CYP4A22	0.830793431	1.50401667	0.575662715	3.929499137	0.404875315
CYP4B1	0.171965378	1.077094176	0.855804419	1.355603966	0.526782399
CYP4F11	0.316069179	0.932436707	0.842166157	1.032383223	0.178136918

CYP4F12	0.861337707	0.034426717	2.97E-05	39.90552386	0.349340522
CYP4F2	0.520280685	0.735400088	0.353256696	1.530935708	0.411333186
CYP4F3	0.287414616	0.522925055	0.175929823	1.554316422	0.243431649
CYP4F8	0.089682949	2.305602852	0.963831349	5.515284927	0.060493488
CYP4V2	0.215941164	1.072698396	0.369785027	3.111758898	0.897239766
CYP4X1	0.221715495	0.625476401	0.24823986	1.575978683	0.319631418
CYP4Z1	0.35700109	14.17083957	0.103949201	1931.834894	0.290415662
CYP4Z2P	0.298442481	22.48536027	0.705406133	716.7380641	0.078004431
CYP51A1	0.777893389	0.876930308	0.748218388	1.027783835	0.104893276
CYP7A1	0.97753377	0.239766505	9.73E-05	590.71032	0.72003346
CYP7B1	0.659505845	0.845311162	0.507682482	1.407476103	0.518265759
CYP8B1	0.331624632	1.39008815	0.944096581	2.046766297	0.095208726
CYR61	0.4675149	1.037630511	0.873356592	1.232803515	0.67442978
CYS1	0.846239529	0.683353603	0.000360412	1295.663344	0.921239506
CYSLTR1	0.14583143	3.062502122	0.299332097	31.33282176	0.345511393
CYSLTR2	0.621537736	2.387187204	0.54728962	10.4125175	0.246923451
CYTL1	0.910840696	37.97773969	0.000138465	10416430.68	0.569170195
CYYR1	0.748430226	0.203921445	0.013021127	3.193575873	0.257318585
D4S234E	0.16698007	0.89267968	0.565302603	1.40964681	0.626234006
DAAM1	0.868716426	0.55795615	0.095609755	3.256101493	0.516796426
DAAM2	0.62114536	1.103861758	0.703369218	1.732391394	0.667393475
DAB1	0.463306925	0.071800271	0.000427777	12.05130974	0.313617559
DAB2	0.531069013	1.472211186	0.505949252	4.283840259	0.477874559
DAB2IP	0.095470681	1.269784937	0.693125464	2.326207691	0.439361364
DACH1	0.384571052	1.440059628	0.422290252	4.910773384	0.560126506
DACH2	0.408376927	0.664517518	0.301112947	1.466504636	0.311567726
DACT1	0.478862826	1.053207807	0.631382227	1.756854465	0.842598421
DACT2	0.975954239	1.716549041	0.694325967	4.243742489	0.242001843
DAD1	0.341267705	1.007200804	0.343975603	2.949201775	0.989556451
DAG1	0.239917082	0.863607767	0.071728398	10.39781163	0.908045113
DAK	0.58856706	1.632588933	0.681185331	3.912806842	0.271724577
DALRD3	0.082900449	0.948522924	0.852977247	1.054771087	0.329261304
DAO	0.271412827	0.763688917	0.420141336	1.388153729	0.376565525

DAOA	0.679629168	1.002396872	0.784845496	1.280251328	0.984699023
DAP	0.144075081	9598.034486	0.428982018	214746218.1	0.072758343
DAP3	0.367731389	12.72558646	0.061875167	2617.213957	0.349271046
DAPK1	0.773212429	0.458951974	0.13438418	1.567423442	0.213947919
DAPK2	0.858120328	1.193385699	0.000885067	1609.109586	0.961650793
DAPK3	0.512187576	0.664262016	0.043951911	10.03924555	0.767801736
DAPP1	0.848740217	1.075284071	0.570215733	2.027716467	0.822542811
DARS	0.146236944	1.616782338	0.389493661	6.711239206	0.508247595
DARS2	0.895560995	3.509416979	0.058085658	212.0318144	0.548528208
DAXX	0.937672587	0.472625719	0.021692476	10.29735263	0.633570226
DAZ1	0.198296793	0.107080756	0.005495723	2.086402078	0.140328703
DAZ2	0.765148821	3.249149547	0.73664928	14.33107052	0.119636428
DAZ3	0.190674137	2.906176659	0.971480631	8.693804598	0.056363952
DAZ4	0.951177836	2.981414503	0.651019329	13.6537151	0.159398192
DAZAP1	0.378613441	2.856395731	0.601225419	13.57061147	0.186816951
DAZAP2	0.772833842	155.8160046	0.177365131	136885.0078	0.144329785
DAZL	0.140356645	71.02446837	0.029877195	168840.317	0.282451691
DBF4	0.733343657	0.962276686	0.639717625	1.447476799	0.853543939
DBF4B	0.456398819	0.000687762	4.91E-09	96.26932111	0.228389586
DBH	0.973267088	0.937164638	0.762708029	1.151525256	0.536906586
DBI	0.966854327	1.053158219	0.858963618	1.291256359	0.61844981
DBN1	0.425318452	1.733387835	0.630386637	4.766334202	0.286479984
DBNL	0.669929894	1.084626267	0.322920742	3.643042976	0.895448126
DBP	0.980165059	0.592665299	0.048157092	7.293882221	0.682934081
DBR1	0.183959317	9756.699771	0.09518368	1000099919	0.118659225
DBT	0.12546612	6.749268738	0.060151314	757.3006391	0.427876134
DBX1	0.109538445	3.627383312	0.509050762	25.84793239	0.198426449
DBX2	0.345771541	119.6908223	0.164042585	87330.32931	0.154865079
DCAKD	0.65045702	0.057725487	0.001805624	1.845473499	0.106668162
DCBLD1	0.763595874	0.38142875	6.23E-05	2337.059797	0.828500561
DCBLD2	0.959182276	5.403818129	0.005953513	4904.877622	0.62732333
DCC	0.541039992	0.711365047	0.022665935	22.32602483	0.84642184
DCD	0.620005235	0.038657615	4.21E-05	35.51938269	0.350075678

DCDC1	0.301805709	0.352855685	0.078949479	1.577048206	0.172686041
DCDC2	0.304359961	1.151537467	0.863236135	1.536124919	0.337214921
DCHS1	0.36778645	0.956366511	0.638075983	1.433429448	0.828929504
DCHS2	0.24979621	1.173282567	0.614637443	2.23968129	0.628063978
DCK	0.296573986	1.260193862	0.805845199	1.970711711	0.310707424
DCLRE1A	0.745683902	2.620323787	0.393994869	17.42686847	0.319021263
DCLRE1B	0.537073743	1.053999646	0.801418222	1.386186668	0.706732341
DCLRE1C	0.141821448	0.072168392	0.002906941	1.79166942	0.108688503
DCN	0.622457945	0.751049347	0.391467585	1.440924213	0.38914881
DCP1A	0.088937294	309.1856119	0.009110988	10492357.75	0.281359399
DCP1B	0.53684781	0.256180003	0.031994602	2.051227048	0.199461072
DCP2	0.937358303	0.959846953	0.201201167	4.579029956	0.959001051
DCPS	0.132444527	694.7700075	0.039250822	12297968.17	0.189795082
DCST1	0.449813183	0.292670293	0.005650009	15.16031157	0.541808617
DCST2	0.974435267	2.184299378	0.550884081	8.660921485	0.266293807
DCT	0.302612904	0.86415981	0.30580505	2.441987723	0.782963308
DCTD	0.683659879	0.786538013	0.391822604	1.578882996	0.499444456
DCTN1	0.569381039	1.387705622	0.380841028	5.056511126	0.619433285
DCTN2	0.456460929	1.449036944	0.468268769	4.48398058	0.519874642
DCTN3	0.851382751	0.137756005	0.002414647	7.859003753	0.336681187
DCTN4	0.268009729	1.321918815	0.972000622	1.797806825	0.075248994
DCTN5	0.708466651	3.594117723	0.00465843	2772.968847	0.706068357
DCTN6	0.683670308	1.224192566	0.6853675	2.18663336	0.494314161
DCUN1D1	0.679717339	0.979818926	0.148687562	6.45679515	0.983092157
DCUN1D2	0.387617612	0.739550935	0.412397197	1.326234974	0.311308876
DCUN1D3	0.226230025	1.237329856	0.889361948	1.721442182	0.206225394
DCUN1D4	0.72957453	1.245845148	0.723171268	2.146282909	0.428317256
DCUN1D5	0.833313456	0.88738675	0.181700425	4.333810686	0.882616932
DCX	0.599482905	0.933487155	0.75235926	1.158220964	0.531728386
DCXR	0.326349119	0.677928168	0.250823887	1.832307944	0.443533308
DDA1	0.831861362	2.071122485	0.656017112	6.538775089	0.214507884
DDAH1	0.087715682	0.398226747	0.106760038	1.485429809	0.17042976
DDAH2	0.099624536	36.96737409	0.027683861	49364.02356	0.325542708

DDB1	0.750872672	0.929377615	0.737150154	1.171732443	0.535600291
DDB2	0.523613053	0.795285051	0.376055887	1.681873186	0.548896753
DDC	0.877204506	1.044504369	0.738448925	1.477406682	0.80558796
DDHD1	0.510389908	0.051493563	0.000943418	2.810616451	0.146066192
DDHD2	0.730401525	1.192875543	0.319775978	4.449840376	0.792882711
DDI1	0.577074156	2.707693444	0.075663651	96.89730421	0.585263907
DDI2	0.194274587	0.959893002	0.41501277	2.220159577	0.923775462
DDIT3	0.560223061	0.872832163	0.081115874	9.391946971	0.910662626
DDIT4	0.527934358	1.376580593	0.716324832	2.645411749	0.33758371
DDIT4L	0.377814543	0.240807609	2.94E-06	19703.00085	0.805155998
DDN	0.294624991	2.078175621	1.291593431	3.343787455	0.002574795
DDO	0.142148488	0.026934487	1.98E-05	36.72980745	0.326374445
DDOST	0.506247958	0.516154501	0.137599588	1.936164721	0.326860858
DDR1	0.530076556	0.934397624	0.420437115	2.07664568	0.867742045
DDR2	0.435335305	26.33452343	0.007425804	93391.51793	0.432850094
DDT	0.664800226	14.71355463	0.696272278	310.9253329	0.08409779
DDX1	0.87433541	0.560062449	0.224015683	1.400214229	0.214994428
DDX10	0.66607882	1.217923246	0.234689694	6.320418275	0.814472077
DDX11	0.881495268	9.140323376	0.018237355	4581.010371	0.485443542
DDX17	0.512710374	0.987374115	0.026397395	36.93196459	0.994513691
DDX18	0.780621761	36.16337565	0.118125116	11071.22499	0.219230645
DDX19A	0.17302951	0.000591507	6.89E-09	50.80897855	0.199737488
DDX19B	0.216379113	0.000176956	2.96E-09	10.57219654	0.123634521
DDX20	0.798730614	0.875210112	0.415862104	1.84193927	0.725524396
DDX21	0.093572837	8.324419194	0.216898786	319.4852135	0.254815258
DDX23	0.5445919	0.962241664	0.154612718	5.988569587	0.967088217
DDX24	0.820560919	0.910293056	0.386266686	2.14523664	0.829850847
DDX25	0.844874758	0.935261893	0.648906464	1.347982887	0.719698081
DDX26B	0.016376302	2.622709369	1.143883329	6.013379386	0.022757025
DDX27	0.320844511	0.813685869	0.453715311	1.459251378	0.489037476
DDX28	0.278182816	2.17948182	0.715149068	6.642169042	0.170596912
DDX31	0.057441746	1.302438895	0.980921074	1.729341046	0.067731487
DDX3X	0.258131081	0.016708208	1.31E-09	213473.8028	0.62404924

DDX3Y	0.145315025	0.385814885	0.079350502	1.875893926	0.237870682
DDX4	0.649804635	1.084029397	0.908150581	1.293970138	0.371700318
DDX41	0.329930939	1.904520872	0.453256693	8.002528828	0.379084024
DDX42	0.754703018	1.008982409	0.842456309	1.20842528	0.922593661
DDX43	0.663116977	0.917159878	0.593972187	1.416198032	0.696452022
DDX46	0.074850644	0.916627464	0.712530764	1.179185447	0.498150184
DDX47	0.941869371	0.953893517	0.24355633	3.735944138	0.945970608
DDX49	0.755525862	0.439797208	0.002606315	74.21266134	0.753567589
DDX5	0.670757429	0.701703094	0.268639214	1.832894107	0.469599749
DDX50	0.583930818	1.076990831	0.446068336	2.600294968	0.869004388
DDX51	0.456568094	1.447582123	0.424840075	4.93243017	0.554273754
DDX52	0.086722596	6.660424728	0.401243061	110.5595632	0.185876474
DDX53	0.48537494	0.590946889	0.262731996	1.329180419	0.203405748
DDX54	0.159246693	1.213685652	0.938859029	1.568960638	0.13931289
DDX55	0.860869939	0.498858747	0.00655683	37.95432182	0.753024528
DDX56	0.754152052	1.315587443	0.258527357	6.694727949	0.741092017
DDX58	0.912977118	0.00791669	5.01E-08	1251.58704	0.428222042
DDX59	0.70256956	0.740055033	0.30566472	1.791771886	0.504610228
DDX6	0.559427388	0.84638148	0.604783097	1.184493438	0.330749728
DEAF1	0.876059855	0.913527819	0.796421871	1.047853037	0.196308603
DECR1	0.748478285	0.419994118	0.100826061	1.749498665	0.233398826
DECR2	0.172557008	0.013324903	0.000107064	1.658387743	0.079355104
DEDD	0.597630748	1.809355283	0.93117965	3.515719596	0.080190524
DEDD2	0.874047058	2.092438163	0.475055264	9.216396053	0.329054523
DEF6	0.421011453	0.452882138	3.75E-06	54732.91957	0.894455499
DEFA3	0.954557674	0.206178399	0.005997927	7.087370964	0.381628115
DEFA4	0.122212395	0.500793049	0.101945253	2.460081965	0.394472064
DEFA5	0.37111766	0.754398058	0.398945114	1.426553202	0.385921207
DEFA6	0.508743504	0.933841579	0.376413614	2.31676024	0.882619814
DEFB1	0.350717564	1.558116038	0.571257044	4.24979545	0.386347495
DEFB103A	0.139662024	1.20245214	0.936108865	1.544575853	0.14897876
DEFB104A	0.882526576	3.375850752	0.414413687	27.49998046	0.255601191
DEFB105A	0.511288752	1.419607819	0.691020706	2.9163907	0.34016397

DEFB106A	0.396572132	9.652153821	0.868342045	107.2896031	0.065026287
DEFB108B	0.825014524	1.374189232	0.120394671	15.68504678	0.79805176
DEFB114	0.052228296	1.127936157	0.65053371	1.955686467	0.668110603
DEFB118	0.404431754	0.006444154	4.89E-07	84.9598642	0.29731424
DEFB119	0.560477943	1.758893941	0.561865217	5.506138841	0.332125341
DEFB121	0.765369078	2.428321095	0.965870127	6.105109968	0.059276275
DEFB123	0.467527697	1.297868765	0.906908358	1.857368846	0.153967435
DEFB125	0.862156648	0.51882934	0.001278027	210.6246221	0.830449612
DEFB126	0.675783537	0.606803158	0.2279236	1.615497793	0.317356146
DEFB127	0.900281806	1.002202376	0.202093114	4.970033779	0.997851436
DEFB129	0.507270547	0.674217253	0.11787466	3.85637512	0.657739924
DEFB134	0.426360157	1.199859703	0.157922246	9.116279313	0.860211938
DEFB136	0.113842369	2.221825312	0.256464968	19.24827301	0.468635511
DEGS1	0.506212104	2.733551809	0.017613603	424.2349333	0.696021251
DEGS2	0.892353762	1.057073875	0.799296741	1.397985404	0.697141796
DEK	0.47971397	1.102700282	0.872655165	1.393388774	0.412827946
DENND1A	0.703020491	0.93681433	0.32446493	2.704825722	0.903967468
DENND1B	0.283969986	0.186700556	0.029431171	1.184359877	0.075001219
DENND1C	0.935674408	0.676106612	0.161337567	2.833315012	0.592378203
DENND2A	0.466566264	0.527746451	0.14380407	1.936776304	0.335303675
DENND2C	0.21135556	30.89812383	0.030172235	31641.47601	0.332013644
DENND2D	0.332121058	0.567010501	0.283968193	1.132172248	0.107809111
DENND3	0.167603467	1.965528074	0.610212811	6.331070965	0.257506091
DENND4A	0.094995193	1.775660039	0.531541238	5.931747801	0.350810563
DENND4C	0.118909217	0.014472727	5.83E-06	35.93101709	0.288255623
DENR	0.382392422	2.089867793	0.248452371	17.57901274	0.497527916
DEPDC1	0.486028247	0.61128162	0.297883762	1.25439942	0.179601966
DEPDC1B	0.354808758	25.52086895	0.518250372	1256.75694	0.103235891
DEPDC4	0.995486785	1.067699378	0.749166795	1.521666429	0.717070928
DEPDC5	0.722723578	0.78657396	0.302105267	2.047956996	0.622922271
DERA	0.703331302	1.693144492	0.015675258	182.8830057	0.825539271
DERL1	0.637854431	4.326859358	0.001039353	18012.84614	0.730472552
DERL2	0.780539863	0.838022273	0.000114859	6114.306013	0.968940795

DERL3	0.389143188	0.055215067	4.03E-06	755.7653949	0.551131706
DES	0.365356092	0.002123677	4.79E-08	94.11556086	0.259549398
DET1	0.747315989	0.912340009	0.670762651	1.240922241	0.558837163
DEXI	0.616860794	2.046908323	0.166365158	25.18456226	0.575904099
DFFA	0.13933752	0.664082035	0.209324403	2.106801415	0.487099418
DFFB	0.202181919	0.868239525	0.214989498	3.50640324	0.84274533
DFNA5	0.383800443	2.251266389	0.479786835	10.5634419	0.303552845
DFNB31	0.530903814	0.061425589	0.000660305	5.714184188	0.227689389
DGAT1	0.696360297	1.041694946	0.80370775	1.350152914	0.75756228
DGAT2	0.527179946	1.601590727	0.621298097	4.12860247	0.3296285
DGAT2L6	0.552043332	0.28332794	0.027924203	2.874736379	0.286078763
DGCR14	0.083278979	0.006579706	1.64E-06	26.37135277	0.235275396
DGCR2	0.171595594	1.961450016	0.743408412	5.175198591	0.173525825
DGCR6	0.840334525	1.864212879	0.482190897	7.207290056	0.366660773
DGCR6L	0.102749284	0.001848465	2.27E-06	1.503534449	0.065667632
DGCR8	0.396988222	0.99884653	0.597021292	1.671120284	0.996493022
DGKA	0.375540024	0.983055874	0.80868184	1.195029744	0.863801261
DGKB	0.098033052	1.152902653	0.883453029	1.504533329	0.294826226
DGKD	0.804353092	0.043778657	0.000101464	18.88924863	0.312172673
DGKE	0.155827535	0.586919192	0.188217783	1.830189115	0.358447425
DGKG	0.465794068	0.90089086	0.620292818	1.308421309	0.583592097
DGKH	0.910725344	2.652262904	0.244048438	28.82418991	0.422950278
DGKI	0.415949578	0.338373942	0.028142782	4.068429486	0.393094942
DGKK	0.283412079	0.235961309	0.002670289	20.8508295	0.52766931
DGKQ	0.3160686	3.427800261	0.913585734	12.86120633	0.067849987
DGKZ	0.381564287	0.375667128	0.084281121	1.674465046	0.19916307
DGUOK	0.722456387	0.990702774	0.666185312	1.47330175	0.963204771
DHCR24	0.103920024	0.523011209	0.217605182	1.2570506	0.147433965
DHCR7	0.94282191	1.086837494	0.498551142	2.369297026	0.834115506
DHDDS	0.566581737	0.605439513	0.063701051	5.754332084	0.66227406
DHDH	0.901197136	2.715714097	0.000439977	16762.45931	0.822483163
DHFR	0.782139345	1.063461823	0.322473723	3.507110712	0.919499935
DHFRL1	0.123318327	1.772669344	0.473708552	6.633523059	0.395177289

DHH	0.795112064	1.137397334	0.741506418	1.744654753	0.555315117
DHODH	0.39972938	0.025098178	3.70E-06	170.2933842	0.412994968
DHPS	0.274169261	0.078360607	0.000214237	28.66166658	0.397757748
DHRS1	0.571315865	1.791302338	0.574043675	5.589755984	0.315377581
DHRS2	0.434888614	0.009772671	4.35E-08	2197.917683	0.461681697
DHRS3	0.154333178	0.379719474	0.030684673	4.698987046	0.450595658
DHRS4	0.562303196	1.127884591	0.748928188	1.698592296	0.564577349
DHRS4L2	0.545375547	1.059608836	0.317293696	3.53858554	0.925021017
DHRS7	0.829015808	0.882069061	0.619565017	1.255793671	0.486283378
DHRS7B	0.694981454	0.613858483	0.188972056	1.994063274	0.416901555
DHRS9	0.801424682	1.104650636	0.440996099	2.767038142	0.831763099
DHRSX	0.960696511	0.917277638	0.656659883	1.281330393	0.612634465
DHTKD1	0.894100305	1.315696735	0.476060733	3.636212316	0.596819524
DHX15	0.195710445	0.402310871	0.119868627	1.350261872	0.140516104
DHX16	0.67435702	0.464240454	0.074443475	2.895071704	0.411255541
DHX29	0.510411275	67.19263087	0.00224824	2008170.408	0.423568217
DHX30	0.362344408	1.354978892	0.620914932	2.956874935	0.445459908
DHX32	0.239702604	0.823776261	0.554231482	1.22441137	0.337706418
DHX33	0.876087916	0.376187443	0.023827228	5.939297301	0.487394038
DHX34	0.449437794	75.79675523	4.72E-05	121719517.7	0.552743213
DHX35	0.504673238	141.6117507	0.00309413	6481267.851	0.365662104
DHX36	0.075484893	0.500660501	0.078312012	3.200798073	0.464849942
DHX37	0.316989491	0.955500012	0.450092455	2.028428302	0.905656858
DHX38	0.290088723	2.581909366	0.911670722	7.312131246	0.074123049
DHX40	0.093222283	5.957671466	0.793397527	44.73652628	0.082744665
DHX57	0.225610463	0.011396165	5.90E-05	2.200904671	0.09567253
DHX8	0.06962691	1.575852877	0.892249762	2.783203084	0.117087446
DHX9	0.508197443	0.891156504	0.702181711	1.130989174	0.34329592
DIABLO	0.607399008	0.954150082	0.624757334	1.457209592	0.828025739
DIAPH1	0.364822329	0.819176445	0.421486126	1.592104714	0.556337653
DIAPH2	0.995737716	0.990561919	0.850057145	1.154290534	0.903292755
DIAPH3	0.86058109	0.934032686	0.68198728	1.279227757	0.67062159
DICER1	0.92463562	1.019737855	0.311128752	3.342234642	0.974255977

DIDO1	0.783408261	1.084925762	0.871929133	1.349953642	0.464797812
DIO1	0.47015429	1.151272754	0.305538412	4.338010879	0.835126318
DIO2	0.762010921	0.924980919	0.740182787	1.155916776	0.492856438
DIO3	0.661728451	1.128024527	0.442448206	2.875905737	0.800821388
DIP2A	0.131477368	0.780632825	0.385681123	1.580029646	0.491201714
DIP2B	0.328251705	1.11658675	0.836871864	1.489793149	0.453531816
DIP2C	0.683292501	0.024656196	1.40E-05	43.52132471	0.331679055
DIRAS1	0.737492322	2.683598119	0.242256226	29.72761108	0.421098983
DIRAS2	0.242686844	0.6256904	0.253037032	1.547158823	0.310039121
DIRAS3	0.139050926	0.400461916	0.142244694	1.127421642	0.083119943
DIRC1	0.816977386	1.12240254	0.773378526	1.628940317	0.543427124
DIRC2	0.202546877	1.053207887	0.764168017	1.451574561	0.751457494
DISC1	0.431674594	1.03493156	0.912559815	1.173713016	0.592797226
DISC2	0.204527	0.8161673	0.191741065	3.474107451	0.783417616
DISP1	0.174514321	0.758768491	0.383847658	1.499890935	0.427201596
DISP2	0.807785104	0.924582801	0.665485383	1.284556172	0.640229461
DIXDC1	0.513852268	2.347806745	0.784755605	7.02409321	0.126895389
DKC1	0.570279544	0.293280191	0.002019739	42.58633472	0.629139521
DKFZP434I0714	0.405797182	0.740907935	0.373294268	1.47054111	0.391226965
DKFZP434L187	0.110839436	0.017534739	9.78E-05	3.142613305	0.126654549
DKK1	0.40787701	1.196625681	0.233781217	6.125013125	0.829406147
DKK2	0.446059282	0.776700134	0.226096201	2.668169995	0.688173678
DKK3	0.497696633	0.564563996	0.029069053	10.96466784	0.705625208
DKK4	0.147194634	0.383156665	0.145844426	1.006613921	0.051584205
DKKL1	0.65151806	4.707185368	0.021223844	1043.995358	0.574066306
DLAT	0.953855249	0.863365394	0.600989539	1.240287484	0.426684964
DLC1	0.135648979	0.47548871	0.162653753	1.390004902	0.174372579
DLD	0.907342468	0.981676848	0.782445874	1.231637186	0.873048388
DLEC1	0.304745798	9.615277524	0.026441978	3496.469169	0.451828564
DLEU1	0.361512932	0.778825788	0.34629553	1.751595255	0.545524441
DLEU2	0.003123082	2.236812115	1.231254809	4.063601133	0.00821937
DLEU7	0.331095288	1.051173586	0.802470655	1.37695491	0.717107746
DLG1	0.194100615	1.815925373	0.691454122	4.769058209	0.225887628

DLG2	0.692078579	0.499754398	0.008856858	28.19898969	0.736040099
DLG3	0.190592233	0.263136929	0.009034166	7.664353958	0.437696232
DLG4	0.455906784	1.278000759	0.113789831	14.35353167	0.842439956
DLG5	0.167691398	2.05648359	0.73893463	5.723273188	0.167394396
DLGAP1	0.17254931	2.123981599	0.618383868	7.295303233	0.231495536
DLGAP2	0.33718079	1.472200747	0.607991019	3.564814232	0.391356455
DLGAP3	0.408089975	2.132166284	0.487450875	9.326340963	0.314609334
DLGAP4	0.325016438	1.133640473	0.668468798	1.922514151	0.641614921
DLK1	0.75248223	1.427917035	0.407862195	4.999107747	0.577403524
DLL1	0.687680026	9.558001313	0.00655402	13938.83386	0.543636656
DLL3	0.209462284	0.696177914	0.258175784	1.877262384	0.474268779
DLL4	0.38388867	0.853465218	0.204409859	3.563442978	0.827976225
DLST	0.113869211	17.46832957	0.023983839	12722.83955	0.39497843
DLX1	0.942899115	0.060000417	0.000251071	14.33879194	0.31398189
DLX2	0.958049938	117.2643426	2.132432502	6448.469542	0.019787527
DLX3	0.684702927	0.650632541	0.258513533	1.637526277	0.361404185
DLX4	0.702149145	0.453175878	0.143728387	1.428864405	0.176741501
DLX5	0.147793988	0.000906924	2.51E-07	3.278768074	0.093759713
DMAP1	0.652946513	0.786683962	0.399027475	1.550949984	0.488452188
DMBT1	0.918881842	1.883817912	0.627789337	5.652803761	0.258650963
DMBX1	0.390308381	1.024146396	0.585437887	1.791609093	0.933360201
DMC1	0.83142136	0.026347364	7.10E-08	9770.748823	0.578354696
DMD	0.192869304	0.882807441	0.003084058	252.7024596	0.965552034
DMGDH	0.091989511	1.121665025	0.919353781	1.368496496	0.257891046
DMP1	0.087804616	1.331261473	0.969849848	1.827352052	0.076639344
DMPK	0.077033842	0.522900664	0.239199615	1.14308338	0.104198431
DMRT1	0.266694932	0.677654713	0.050007357	9.182967106	0.769826855
DMRT2	0.702977923	0.832784285	0.293484523	2.363087696	0.730946707
DMRT3	0.763570577	1.374343879	0.198155173	9.532030204	0.747604869
DMRTA1	0.564336072	0.936832697	0.773514031	1.134634237	0.504379971
DMRTA2	0.932489238	0.101394351	0.000609691	16.86234511	0.380378002
DMRTB1	0.635124412	0.89298291	0.560556706	1.422547387	0.633767102
DMRTC1	0.748493579	1.617604946	0.01635131	160.0266716	0.83743824

DMTF1	0.095309875	528.238251	0.005861827	47602167.76	0.281450315
DMWD	0.211490953	0.095366806	0.004542167	2.00231043	0.130288179
DMXL1	0.624676827	117.0838322	0.002943362	4657471.014	0.37809605
DMXL2	0.676761721	0.941177249	0.731160509	1.21151868	0.63794148
DNAH1	0.562690808	1.768987665	0.014136415	221.3657019	0.81693122
DNAH10	0.471412501	0.43328975	0.152209342	1.233432883	0.117137856
DNAH11	0.109706193	0.016702068	1.31E-05	21.35006155	0.262181643
DNAH17	0.734168219	2.156974218	0.000524427	8871.653725	0.856332568
DNAH3	0.33589931	257.3098047	4.81E-05	1376765464	0.482580156
DNAH5	0.250771884	2.160418157	0.806412517	5.787864785	0.12551329
DNAH7	0.333299636	1.808779339	0.561579223	5.825861363	0.320664149
DNAH8	0.100873323	0.493966059	0.14314063	1.704634583	0.264415703
DNAH9	0.994024878	0.511273638	0.183680869	1.423124436	0.199003512
DNAI1	0.188152023	0.486889546	0.242206468	0.978757636	0.043359196
DNAI2	0.628738317	0.908851217	0.300429995	2.749427648	0.865623271
DNAJA1	0.515810406	0.873302721	0.361528728	2.10953538	0.763364275
DNAJA2	0.987355067	0.247781892	0.011022811	5.569891733	0.379646127
DNAJA3	0.130886337	0.505464014	0.203228816	1.257173439	0.142199266
DNAJA4	0.411011651	0.722705834	0.336642326	1.551509364	0.40476514
DNAJB1	0.509484872	1.255719812	0.477563022	3.3018307	0.644337622
DNAJB11	0.439723101	1.6359259	0.64364897	4.157939615	0.301043199
DNAJB12	0.596283262	0.577532915	0.122948782	2.71287167	0.486716294
DNAJB13	0.655436162	0.837167531	0.567260516	1.235498425	0.370776734
DNAJB14	0.706698506	0.244833242	0.002157183	27.78776895	0.559979532
DNAJB2	0.082625587	0.001295162	2.56E-08	65.41279121	0.228842703
DNAJB4	0.327978649	0.448945089	0.075814907	2.658470507	0.377498314
DNAJB5	0.11738007	1.426712263	0.80862315	2.517251553	0.219933009
DNAJB6	0.665106974	1.185148749	0.06638328	21.15860448	0.908036683
DNAJB7	0.796420263	0.98618327	0.752617951	1.292232586	0.919637445
DNAJB8	0.312792155	0.615219747	0.225406258	1.679169603	0.343007907
DNAJB9	0.438245041	2.27764911	0.76163792	6.811222674	0.140808386
DNAJC1	0.09158147	0.488808579	0.06999875	3.413401328	0.470386498
DNAJC10	0.528308534	1.87886215	0.114911033	30.72048759	0.658225361

DNAJC11	0.070372677	0.490111414	0.014493214	16.57390841	0.691393334
DNAJC12	0.562431666	1.05382908	0.843591552	1.316461416	0.644214944
DNAJC13	0.587131579	0.716895819	0.427039273	1.203494964	0.207964903
DNAJC14	0.875238018	1.129904047	0.650773671	1.96179288	0.664385044
DNAJC15	0.824738252	0.724763689	0.210238786	2.4985038	0.610190497
DNAJC16	0.608107811	0.254774263	8.44E-06	7688.209406	0.795001597
DNAJC17	0.592885597	1.255895713	0.449204871	3.511257652	0.664027451
DNAJC18	0.782790267	0.674415494	0.247008606	1.841378187	0.442102676
DNAJC19	0.752209276	0.963896484	0.502858389	1.847630375	0.911804513
DNAJC3	0.186776835	0.756056933	0.106066488	5.389280781	0.780201266
DNAJC4	0.172798379	2.163662181	0.745228922	6.281873791	0.155833205
DNAJC5	0.66846854	0.509020234	0.037963714	6.824980241	0.610155924
DNAJC5B	0.403946081	0.808680112	0.246556591	2.652387112	0.726043139
DNAJC5G	0.890335631	0.541652201	0.121555352	2.413609117	0.421267695
DNAJC6	0.139834304	0.873109676	0.708692194	1.075672221	0.202411572
DNAJC7	0.07609481	0.392851052	0.11132444	1.386325852	0.14643604
DNAJC8	0.826341386	1.268886643	0.436746545	3.686516427	0.661658107
DNAJC9	0.333449913	1.076092648	0.516459042	2.242143702	0.844765588
DNAL4	0.49128725	1.417215851	0.771853287	2.60217946	0.260717178
DNALI1	0.653809794	0.073387887	5.43E-06	992.1938769	0.59043127
DNASE1	0.823090144	1.09199842	0.631068262	1.889590428	0.75308727
DNASE1L1	0.935264413	0.311910113	0.000541482	179.6696331	0.719409675
DNASE1L2	0.86321654	0.927270249	0.580971668	1.479986308	0.751590654
DNASE1L3	0.172497508	0.728013539	0.491980913	1.077285112	0.112368643
DNASE2	0.614099568	1.030252489	0.656467008	1.616867529	0.896873105
DNASE2B	0.444791665	261.7465838	0.005594921	12245261.5	0.310226147
DNER	0.514113017	0.285341353	0.000728257	111.8007473	0.680589147
DNHD1	0.483970223	0.513826803	0.232364404	1.136223875	0.100063085
DNM1	0.133986157	0.81564113	0.433417886	1.534940008	0.527586276
DNM1L	0.486741168	2.161214251	0.182530347	25.58942725	0.541094091
DNM2	0.6775581	0.737522898	0.425666419	1.277855148	0.277626557
DNM3	0.941234217	1.265710689	0.369443106	4.33632005	0.707623265
DNMBP	0.090405885	0.57171887	0.246809364	1.324351962	0.192058818

DNMT1	0.604768955	0.541510605	0.089026753	3.293770987	0.505476981
DNMT3A	0.375836705	9.34554778	0.000475552	183658.8568	0.657703229
DNMT3B	0.085895546	2.33298815	0.79928067	6.809665135	0.121133871
DNMT3L	0.329830473	1.376736884	0.895945178	2.115536189	0.144656683
DNPEP	0.776597915	1.377779359	0.626993357	3.027585445	0.424976414
DNTT	0.071626345	6.944578608	0.121591354	396.633235	0.347726717
DNTTIP1	0.734089442	2.336980304	0.016259549	335.893507	0.737704619
DNTTIP2	0.25317381	0.484476585	0.166791279	1.407253199	0.18285627
DOC2A	0.317736455	0.626937341	0.137132501	2.86620916	0.547110634
DOC2B	0.588236307	1.409303969	0.273204918	7.269772784	0.681896374
DOCK1	0.068383295	0.006780176	4.53E-05	1.014119917	0.050644912
DOCK10	0.68232215	1.275693081	0.507177509	3.208724375	0.60488491
DOCK11	0.935833141	32.21840319	0.012668221	81939.32878	0.385401981
DOCK2	0.260010065	1.102240762	0.483665488	2.511931751	0.816827495
DOCK3	0.311880502	2.080582855	0.611231949	7.082131468	0.241082497
DOCK4	0.159856244	2.295263791	0.043889885	120.0330308	0.680676394
DOCK5	0.43994085	2.148247301	0.055324784	83.41589701	0.682121826
DOCK6	0.149586279	0.512787659	0.08451741	3.111207316	0.467791655
DOCK7	0.14520419	0.851838063	0.583273333	1.244061823	0.406624017
DOCK8	0.946342797	0.974386319	0.131104872	7.241749952	0.979772324
DOCK9	0.421410386	0.530044606	0.197034041	1.42588196	0.208656432
DOK1	0.838008856	1.666349151	0.811490787	3.421751104	0.164234794
DOK2	0.96880357	0.685421381	0.141919565	3.310343226	0.638274712
DOK3	0.99528004	0.805344243	0.250166946	2.592586113	0.716665327
DOK4	0.077708515	1.806907226	0.709511831	4.601633937	0.214816438
DOK5	0.397072599	0.013466423	3.94E-07	460.8298794	0.418723361
DOK6	0.210276625	3.873675047	0.444514553	33.75673138	0.220210901
DOLPP1	0.885086145	0.759755713	0.215709703	2.675951685	0.668860872
DONSON	0.137964902	0.842681592	0.601789535	1.18000102	0.319041346
DOPEY1	0.217430286	0.490744085	0.134430008	1.79148808	0.28127977
DOPEY2	0.115704435	4.036330354	0.906352692	17.9753013	0.067109359
DOT1L	0.178431914	1.379948759	0.542284514	3.511548879	0.499171204
DPAGT1	0.070998866	1.886971367	0.890905518	3.996676267	0.097260466

DPCR1	0.335934119	1.147700132	0.99949914	1.317875665	0.050835937
DPEP1	0.777059831	0.1876121	0.000382614	91.99436853	0.596519837
DPEP2	0.582568064	0.794238425	0.268873391	2.346140213	0.676780159
DPEP3	0.476630918	0.143839339	0.001728663	11.96864573	0.390022906
DPF1	0.984704594	1.501178229	0.52035655	4.330753743	0.452335785
DPF2	0.391213037	2.346587858	0.330410162	16.66557271	0.393781273
DPF3	0.885827453	1.346326263	0.524739393	3.454275457	0.536188375
DPH1	0.183492539	0.368088039	0.08543093	1.585945555	0.17988274
DPH2	0.185894593	1.69468453	0.798718569	3.595704128	0.169321144
DPH5	0.534377255	0.598198602	0.056258407	6.360677152	0.670094573
DPM1	0.066609967	1.773102865	0.700382698	4.488822721	0.226852677
DPM2	0.414464344	0.302254013	0.047891947	1.907575163	0.203056844
DPM3	0.501446993	1.656287559	0.428082021	6.408324443	0.464824725
DPP10	0.763739983	1.619358695	0.611432622	4.288816937	0.332046215
DPP3	0.843497488	0.310836256	0.058020647	1.6652551	0.172423802
DPP4	0.609991591	2.880292282	0.018061943	459.3129106	0.682677523
DPP6	0.857135282	5.076128624	0.003544541	7269.512229	0.661270867
DPP7	0.429655172	1.373826364	0.521478522	3.619322368	0.520479713
DPP8	0.112302771	1.860942234	0.941380488	3.678752684	0.074061823
DPP9	0.122938872	0.034894658	8.58E-05	14.19957299	0.273731656
DPPA2	0.564927299	0.349870591	0.023865363	5.129166809	0.443338479
DPPA3	0.131904	0.000520608	2.30E-08	11.79467982	0.139495609
DPPA4	0.901689092	0.50077914	0.191048584	1.312649072	0.159533919
DPPA5	0.654502027	0.07441639	5.14E-07	10782.53311	0.668290231
DPRX	0.846567707	0.781145438	0.102483197	5.954031602	0.81161138
DPRXP4	0.074024247	1.049505119	0.773967236	1.423136462	0.755826448
DPT	0.166667616	0.762593788	0.562400038	1.034049156	0.081079232
DPY19L1	0.84753225	0.956355927	0.095257337	9.601535049	0.969751696
DPY19L2	0.849054989	1.077850093	0.502566153	2.311657511	0.847290454
DPY19L3	0.185953453	0.609044181	0.197581519	1.877376059	0.387960075
DPY19L4	0.812013591	0.753312651	0.136563624	4.155425389	0.745088225
DPYD	0.515841936	1.871363058	0.628686098	5.570346959	0.260159679
DPYS	0.781467657	0.65585411	0.214306011	2.007151419	0.459825705

DPYSL2	0.209888976	6.352418958	0.077831035	518.4721836	0.410409354
DPYSL3	0.72054708	2.299982946	0.302480001	17.48850016	0.42099018
DPYSL4	0.813825749	1.080775451	0.417554733	2.797419076	0.872810927
DPYSL5	0.928351612	0.140375732	3.01E-06	6557.153733	0.720403679
DQX1	0.241604271	1.067956515	0.750875937	1.518934168	0.714504981
DR1	0.908925742	5.328674191	0.035293418	804.534389	0.513369033
DRAP1	0.472845494	1.002175125	0.7279135	1.379772434	0.989373681
DRD1	0.492199873	0.67780891	0.003556427	129.1815848	0.884568856
DRD2	0.889330862	1.788539768	0.48103659	6.649960866	0.38553835
DRD3	0.796565475	1.163319125	0.517105061	2.617091747	0.714595089
DRD4	0.450939882	0.911702673	0.615793213	1.349806635	0.644279284
DRD5	0.82429366	0.746581018	0.237691764	2.3449833	0.616744749
DRG1	0.779785638	1.022924454	0.630318794	1.660071775	0.92689844
DRG2	0.262736741	7.414224814	0.034749629	1581.908381	0.464067699
DRP2	0.522445604	0.053279366	0.001435787	1.977098005	0.111771274
DSC1	0.638708582	1.15208317	0.483463777	2.745387956	0.749314639
DSC2	0.09613752	1.077866766	0.982949972	1.18194903	0.110864879
DSC3	0.604349034	0.725085144	0.195439818	2.690078571	0.63081276
DSCAM	0.530070089	0.127131749	1.84E-06	8765.698226	0.716721827
DSCAML1	0.783380317	1.10815352	0.729345121	1.683708012	0.630389345
DSCR10	0.588140451	0.861582233	0.297780009	2.492860237	0.783431563
DSCR3	0.917131558	4.588477823	0.000803298	26209.61678	0.729943601
DSCR4	0.797686375	1.154351446	0.499912042	2.665523427	0.736740157
DSCR8	0.278052167	0.003363942	8.41E-07	13.4487397	0.178372321
DSCR9	0.62678983	0.865837153	0.003142919	238.5279377	0.959920691
DSG1	0.550746702	1.183428158	0.276413658	5.066689587	0.820440632
DSG2	0.735344529	1.010853441	0.821570268	1.243745933	0.918719156
DSG3	0.564029958	0.107152451	0.006528252	1.75876288	0.117705518
DSG4	0.944047131	0.133002272	4.37E-05	404.6507189	0.622017333
DSP	0.826747774	0.994204762	0.559300806	1.767283542	0.984200929
DSPP	0.871265112	0.829389947	0.237183773	2.900230803	0.76961569
DST	0.610955964	0.791587447	0.385918057	1.623688436	0.52372391
DSTN	0.989382439	0.964874791	0.231909226	4.014430047	0.960793332

DTL	0.536083636	4.266993615	0.000200559	90782.36223	0.775366098
DTNA	0.511237805	1.356076263	0.04615069	39.84648576	0.859820135
DTNB	0.513121766	3.572276336	0.414760153	30.76756081	0.246492879
DTNBP1	0.879741126	2.701291832	0.316974525	23.02070667	0.363351685
DTWD1	0.597924465	1.5000248	0.417716887	5.38660148	0.534176396
DTWD2	0.100881778	1.63408844	0.877349357	3.043536769	0.121717725
DTX1	0.26588505	1.586794647	0.817215072	3.081094977	0.172644096
DTX2	0.239092741	0.187860803	0.022862385	1.543657029	0.119718761
DTX3	0.823155184	0.781262022	0.302278833	2.019229536	0.610397725
DTX3L	0.796478454	5.473256091	0.019109093	1567.658499	0.555927138
DTX4	0.637473683	1.736499042	0.911085493	3.309710173	0.093542532
DTYMK	0.933044457	0.61464931	0.000237251	1592.382477	0.903398929
DUOX1	0.779006816	0.993917701	0.831118942	1.188605321	0.946704409
DUOX2	0.364826549	1.077808563	0.788068229	1.474074522	0.639033739
DUPD1	0.531346086	0.617115012	0.111206108	3.424550534	0.580896995
DUS1L	0.123858669	2.42841603	0.634450158	9.294984552	0.195124566
DUS3L	0.205962559	1.362426666	0.874242966	2.123215731	0.171862939
DUS4L	0.993756835	0.475555408	0.04366693	5.179043855	0.541814152
DUSP1	0.966236081	0.307281287	0.004308987	21.91275912	0.587819426
DUSP10	0.397208797	1.107382428	0.910442883	1.346922321	0.307305198
DUSP11	0.956868514	1.424359915	0.490197196	4.138744941	0.515723783
DUSP12	0.953086472	1.593182525	0.01710742	148.370158	0.840441309
DUSP13	0.480351376	0.583435222	0.331096343	1.028089454	0.062304199
DUSP14	0.312768849	0.910775438	0.699303674	1.186196968	0.488125044
DUSP15	0.564895987	31.66927569	0.002480106	404395.304	0.473813859
DUSP16	0.195500883	0.473925226	0.201291214	1.115821778	0.087428247
DUSP18	0.400979917	0.510955001	0.181039209	1.442090991	0.20465058
DUSP19	0.340128801	1.228380736	0.479073569	3.149660781	0.668531696
DUSP2	0.143891587	0.828544084	0.555658482	1.23544465	0.356154816
DUSP21	0.777456844	3.983338668	0.016053251	988.397132	0.623227691
DUSP22	0.349544	0.693566308	0.31097107	1.546877733	0.371289902
DUSP23	0.571254439	0.377898259	0.032410195	4.406239893	0.437430203
DUSP26	0.343627841	0.476817661	0.156131954	1.456172657	0.193530897

DUSP27	0.87831408	0.897526536	0.699017984	1.152407952	0.396604118
DUSP3	0.593982769	0.989142239	0.405798895	2.411052323	0.980840376
DUSP4	0.849554086	0.187458457	0.002792753	12.58280811	0.435352418
DUSP5	0.993056792	0.919456523	0.303691409	2.78374782	0.88189179
DUSP6	0.954417729	0.733948994	0.226888262	2.374213285	0.605572999
DUSP7	0.143670404	0.902909074	0.792230577	1.029049899	0.125826246
DUSP8	0.46621378	0.612003787	0.23422881	1.59907159	0.316336368
DUSP9	0.860016737	0.883075847	0.358451636	2.175531848	0.786926943
DUT	0.304148107	1.313631218	0.061077845	28.25291192	0.861669021
DUX4	0.39405936	2.03502718	0.496935923	8.333741707	0.323261507
DUXA	0.259287446	0.544779715	0.083060351	3.573124076	0.526776909
DUXAP3	0.977224285	0.779503975	0.240917956	2.52213018	0.677563095
DVL1	0.685938108	2.482423042	0.009157839	672.9124968	0.750415725
DVL2	0.701056735	1.014288246	0.537615863	1.913598011	0.96506118
DVL3	0.131916719	0.018815722	0.000234443	1.51009462	0.075774934
DYDC1	0.245266825	0.343945184	0.054963037	2.152324481	0.254000111
DYM	0.156570694	0.026145141	5.04E-06	135.5027587	0.403687175
DYNC1H1	0.342798697	1.243520433	0.696366753	2.220587158	0.461294443
DYNC1I1	0.338322608	1.069234454	0.854288591	1.338262421	0.558803362
DYNC1I2	0.96837345	1.10714162	0.819319482	1.49607399	0.507578174
DYNC1LI1	0.089874262	0.592304848	0.33282636	1.05407827	0.074932268
DYNC1LI2	0.11216319	0.033610856	3.83E-05	29.53156655	0.326563865
DYNC2H1	0.9437837	1.832565386	0.484355955	6.933528658	0.372295634
DYNC2LI1	0.659396135	0.223630119	0.009433981	5.301094874	0.353766098
DYNLL2	0.491864401	1.539996701	0.113238136	20.94338461	0.7457582
DYNLRB1	0.958265412	1.085056227	0.31998137	3.67942364	0.895757092
DYNLRB2	0.992597445	1.069351425	0.836093718	1.367684561	0.593284142
DYNLT1	0.671102296	0.438507596	0.162701284	1.181852448	0.10317193
DYNLT3	0.546693978	0.139943102	7.45E-06	2629.970886	0.695318148
DYRK1A	0.882847163	2.921555025	0.016530141	516.3588042	0.684687747
DYRK1B	0.140684773	0.308198811	0.042893362	2.214480315	0.242077522
DYRK2	0.341823629	0.489862371	0.130600863	1.837393233	0.290042592
DYRK3	0.935091132	1.682555748	0.971405342	2.914328059	0.063389065

DYRK4	0.951874075	0.659250344	0.297514834	1.460804526	0.304715726
DYSF	0.567097106	1.192961053	0.921710824	1.544037497	0.180064314
DYX1C1	0.745430533	0.917880743	0.499333382	1.687259632	0.78265151
DZIP1	0.838317487	0.712944879	6.48E-06	78416.03098	0.95444278
DZIP3	0.863667692	1.014702008	0.765829613	1.344450709	0.919028035
E2F1	0.083301328	10.81703007	0.81040932	144.3815325	0.071708117
E2F2	0.05320921	4.534548197	0.06269757	327.957323	0.488883631
E2F3	0.50302321	0.649789618	0.274268285	1.539465442	0.327279337
E2F4	0.515073772	0.92230694	0.182984728	4.648749116	0.921930418
E2F5	0.089014883	1.696269129	0.922880818	3.117768734	0.088841047
E2F6	0.730542148	1.046080862	0.843827974	1.296810729	0.681102091
E2F7	0.64300435	0.933294373	0.400070181	2.177213968	0.873092686
E2F8	0.105707206	0.547223681	0.137032295	2.185278713	0.393435514
E4F1	0.213928635	0.634847563	0.213644462	1.886458577	0.413520303
EAF1	0.100841	0.004911406	2.78E-06	8.689630831	0.163529097
EAF2	0.566378656	0.02818271	0.000128028	6.203838978	0.194700972
EBAG9	0.920767579	0.920482449	0.263732409	3.212680391	0.896628311
EBF2	0.206625471	0.003615352	1.12E-05	1.171486378	0.056611007
EBF3	0.416130259	0.432539543	0.072853471	2.568037662	0.356434445
EBI3	0.356250567	0.523432785	0.077902458	3.516986331	0.505384701
EBNA1BP2	0.462933727	1.098045469	0.456818894	2.639347598	0.834425216
EBP	0.648752733	160.4466789	0.00095013	27094330.18	0.40832523
EBPL	0.817670428	0.857838809	0.393160301	1.87172362	0.7000831
ECD	0.937887199	0.505684726	0.136802557	1.869241678	0.306690921
ECE1	0.469183021	0.810265049	0.476238745	1.378572105	0.437787528
ECE2	0.850773591	0.397609272	0.093912946	1.683400854	0.210346942
ECEL1	0.496771397	225.0106226	0.028784343	1758934.717	0.236325027
ECH1	0.38885826	2.512846456	0.837891263	7.536058182	0.100107561
ECHDC1	0.625676568	0.980041446	0.266182791	3.60835212	0.975815365
ECHDC2	0.257504708	0.682057368	2.57E-05	18090.82756	0.941306129
ECHDC3	0.541172384	0.769309723	0.183328631	3.228287063	0.720043313
ECHS1	0.767933152	0.968437464	0.557483188	1.682330771	0.909378398
ECM1	0.414382466	1.013324714	0.770413962	1.332824983	0.924583162

ECM2	0.158231772	22.57243767	0.472060612	1079.342207	0.11421223
ECT2	0.081168364	0.509835265	0.205809609	1.26297309	0.145522475
EDA	0.653066473	2715.700535	0.077897696	94675834.29	0.138427518
EDA2R	0.84800961	0.877819987	0.079717079	9.666284114	0.915211707
EDAR	0.365869773	0.74643723	0.372095627	1.497379971	0.410312784
EDARADD	0.827376696	0.966606308	0.504105733	1.853436081	0.918554685
EDEM1	0.262394717	0.563226705	0.230705106	1.375020806	0.207443236
EDF1	0.563365363	0.633416578	1.99E-06	202035.2446	0.94369909
EDIL3	0.645150043	0.266674731	0.078849566	0.901912536	0.033501253
EDN1	0.934077664	0.920491179	0.735159438	1.152544559	0.470133773
EDN2	0.431191246	0.003152841	1.83E-07	54.26699597	0.24711862
EDN3	0.108427126	0.099421882	0.006497214	1.521376884	0.097219427
EDNRA	0.274295912	1.300739728	0.832200495	2.033072377	0.24854999
EDNRB	0.583356296	1.453972262	0.60356501	3.502581001	0.404050502
EEA1	0.457964968	0.89139638	0.47194756	1.68363516	0.723087612
EED	0.579037713	1.489216828	0.689688547	3.215606189	0.310573997
EEF1A1	0.668306085	1.16282929	0.016084894	84.06470902	0.944933453
EEF1A2	0.425530939	450.8856297	0.015827848	12844313.86	0.242910936
EEF1B2	0.217936308	0.034948586	3.78E-06	322.8731241	0.471588367
EEF1D	0.891309417	1.227651108	0.50776451	2.968161843	0.648863504
EEF1E1	0.682970776	1.000357686	0.761550371	1.31405031	0.997949606
EEF1G	0.578934433	0.805383771	0.159806638	4.058924118	0.793101853
EEF2	0.802623841	3.773725578	0.502014558	28.3677127	0.196916186
EEF2K	0.209793388	0.004443526	3.43E-07	57.53890075	0.262230438
EEFSEC	0.628127397	1.039782732	0.51810679	2.086728354	0.912594661
EFCAB1	0.192976916	2.374212447	0.679794935	8.292036981	0.175389456
EFCAB2	0.770729135	2.986451953	0.333614251	26.73415553	0.327908695
EFCAB3	0.410835956	0.666933823	1.01E-05	43915.13601	0.942955811
EFEMP1	0.858138862	0.094526595	0.000441024	20.26028429	0.3890473
EFEMP2	0.884181777	0.043971971	0.00026853	7.200449829	0.229735139
EFHB	0.586252136	0.834800741	0.614351946	1.134353495	0.248433217
EFHC1	0.734167041	0.584163847	0.001526413	223.561617	0.85938159
EFHC2	0.521478916	1.499944882	0.000699549	3216.119801	0.91749083

EFHD1	0.902770773	1.243414387	0.724823713	2.133041885	0.428828506
EFHD2	0.89500958	1.30147341	0.450297773	3.761584309	0.626545128
EFNA1	0.340584791	1.345344603	0.152496088	11.86884283	0.789436087
EFNA2	0.722025059	0.997073189	0.739655314	1.344078689	0.984652256
EFNA3	0.13626785	1.223023	0.883171656	1.693651794	0.225499823
EFNA4	0.118626386	1.178810777	0.683172523	2.03403211	0.554486887
EFNA5	0.246191469	1.904362847	0.226345244	16.02241683	0.553335135
EFNB1	0.54995288	1.076788468	0.694873304	1.668611239	0.740604697
EFNB2	0.167512056	0.62726665	0.350580052	1.122321273	0.116136977
EFNB3	0.714392021	8.903789502	0.043225781	1834.032058	0.421194426
EFS	0.996299214	0.527935698	0.001081566	257.6967393	0.839728141
EFTUD1	0.169561566	1.958502694	0.843880741	4.545349381	0.117628178
EFTUD2	0.261805348	1.990225827	0.786103302	5.038776493	0.146454714
EGF	0.346910875	0.86520761	0.471509827	1.587632252	0.640155602
EGFL6	0.502293624	0.753486489	0.370044195	1.534254276	0.435302077
EGFL7	0.908583292	2.245653161	0.906442552	5.563461364	0.080506749
EGFL8	0.31083009	1.3520188	0.323700429	5.647057201	0.679235226
EGFR	0.480549748	0.207255853	0.03593767	1.195263601	0.078332542
EGLN1	0.377273647	6.280672913	0.482923372	81.68346065	0.160364901
EGLN2	0.276549333	0.619050679	0.208985996	1.8337293	0.386727736
EGLN3	0.608223229	0.864670882	0.431615066	1.732228072	0.681682328
EGR1	0.163909289	5.278157511	0.468710037	59.43748696	0.178113899
EGR2	0.36873944	1.245373776	0.272150749	5.69888508	0.777333186
EGR3	0.546952468	0.620844603	0.254541668	1.514282607	0.294715639
EGR4	0.750725427	0.026194119	7.73E-05	8.874988981	0.220417749
EHBP1	0.5917398	0.917186373	0.269380773	3.122831793	0.890012861
EHD1	0.877772866	0.313550004	0.048722508	2.017827268	0.222110474
EHD2	0.179418654	169.6614603	0.078027212	368909.9041	0.190399545
EHD3	0.868781085	0.553035385	0.193646155	1.579417561	0.268591586
EHD4	0.21431461	1.945948346	0.47587213	7.957421175	0.354184898
EHF	0.136824621	14.04128666	0.131834104	1495.498698	0.267321092
EHHADH	0.589771288	0.604066113	0.160051053	2.279871712	0.456972917
EHMT1	0.434647048	1.747496653	0.415636051	7.34715996	0.446188696

EHMT2	0.906634557	1.741251314	0.835712461	3.627989625	0.138664926
EI24	0.019314314	2.081166706	1.038750879	4.169676238	0.038715807
EID3	0.36026122	132.9198046	0.003249038	5437816.394	0.366793812
EIF1	0.509588399	16.40587422	0.053679628	5014.056898	0.33795257
EIF1AX	0.797734767	1.324899807	0.086780919	20.22748222	0.839682234
EIF1AY	0.150990847	0.694159942	0.373656003	1.289576568	0.248009878
EIF1B	0.892767925	4.866590808	0.13629361	173.7697472	0.38569426
EIF2AK1	0.084955234	0.596881979	0.16450024	2.16576035	0.432590996
EIF2AK2	0.787658108	1.061968131	0.794642861	1.419224115	0.684473476
EIF2AK3	0.785398361	0.872370999	0.476897423	1.595796336	0.657668943
EIF2AK4	0.341063056	0.082534253	1.87E-05	363.5093083	0.560082661
EIF2B1	0.652732769	12.03036566	9.35E-05	1547348.55	0.678579783
EIF2B2	0.565186058	0.578697229	0.21083625	1.588391386	0.288346272
EIF2B3	0.40290244	1.301957516	0.955249406	1.774503457	0.094884111
EIF2B4	0.949606228	2.468243371	0.008554884	712.1341695	0.754579648
EIF2B5	0.711715821	1.015094845	0.702891409	1.465969752	0.936320595
EIF2S1	0.36250046	1.385402715	0.714035042	2.688020294	0.335062901
EIF2S2	0.651704319	0.980156582	0.796056847	1.206832061	0.850230111
EIF2S3	0.890633412	0.592410823	0.099389424	3.531065654	0.565410685
EIF4A1	0.150121606	2.579196545	0.68242883	9.747909997	0.162502612
EIF4A2	0.76333879	1.277821781	0.337584026	4.836806184	0.718114945
EIF4B	0.365216468	33.71391343	0.364101956	3121.729889	0.127842661
EIF4E	0.613263419	1.132606196	0.893387767	1.43587907	0.303636126
EIF4E2	0.596307125	0.543212621	0.06461567	4.566693372	0.574258713
EIF4E3	0.01892268	0.308127555	0.121625778	0.780612395	0.013057994
EIF4EBP1	0.942641817	0.78308892	0.306405549	2.001361456	0.609545966
EIF4EBP2	0.80217458	1.031641886	0.695144923	1.531026043	0.877092714
EIF4EBP3	0.221060702	1.348577322	0.95195805	1.910442159	0.092394663
EIF4ENIF1	0.224418277	1.125187637	0.303186958	4.175797093	0.860066999
EIF4G1	0.972964507	1.163079939	0.752927589	1.796660083	0.495934457
EIF4G2	0.469867158	7.200368112	0.00028519	181792.3729	0.702674363
EIF4G3	0.648539733	1.5753929	0.525930543	4.718993456	0.416804657
EIF5	0.594577464	0.027740278	7.26E-07	1059.925102	0.505449422

EIF5A	0.380020855	0.094430537	0.00074588	11.95517576	0.339359071
EIF5A2	0.977062493	1.137323426	0.854364044	1.513996972	0.377994409
EIF5B	0.852698061	1.582901147	0.309009487	8.108411382	0.581635915
ELAC1	0.508190999	1.504828437	0.267704381	8.458989783	0.64269907
ELAC2	0.270019654	30.02595029	0.024336344	37045.73277	0.348867192
ELAVL1	0.429758785	0.749074819	0.419695701	1.336952187	0.328329142
ELAVL2	0.318413941	0.94411277	0.110990679	8.030844858	0.958009373
ELAVL3	0.098528594	1.030030061	0.829580655	1.278913533	0.788734726
ELAVL4	0.651671912	5.052856429	0.431830237	59.12359969	0.196758856
ELF1	0.313549067	1.281331116	0.85795142	1.913639153	0.225768227
ELF2	0.388286341	0.154061027	0.002866601	8.279772114	0.357514375
ELF3	0.257018982	1.146274649	0.929867755	1.413045634	0.200958447
ELF4	0.910111532	1.42748766	0.633616633	3.21601567	0.390422434
ELF5	0.574244194	1.444389283	0.756649977	2.757233154	0.265010094
ELK1	0.723304951	2.76289847	0.002148102	3553.652705	0.780846664
ELK3	0.551358685	18.99366753	0.016517362	21841.22399	0.412909445
ELK4	0.693913074	3.142009987	0.007096975	1391.047095	0.712667117
ELL	0.097764309	1.18892882	0.824109352	1.715247781	0.354738561
ELL2	0.576526001	1.726302192	0.612438525	4.865989215	0.301776211
ELL3	0.090155674	1.130674797	0.932015662	1.371678126	0.212840855
ELMO1	0.928237769	0.771296645	0.291466651	2.041051736	0.600965894
ELMO2	0.385939696	22.3829159	0.002009323	249335.1403	0.513249183
ELMO3	0.750893239	2.237388431	0.256064017	19.54943557	0.466518303
ELMOD1	0.47510004	1.023185094	0.766058322	1.36661623	0.87664843
ELMOD2	0.098261754	3.262650799	0.548471555	19.40828133	0.193672483
ELN	0.489820542	0.596077105	0.219224694	1.620747679	0.310687801
ELOF1	0.778947553	1.017931136	0.841550831	1.23127892	0.854750344
ELOVL1	0.67330977	0.58631089	0.00185852	184.9645868	0.855692638
ELOVL2	0.076974186	1.213964673	0.766357861	1.923005299	0.408727026
ELOVL3	0.556145618	0.74980866	0.231201422	2.431702284	0.631461436
ELOVL4	0.284493978	0.681641979	0.296880751	1.565058652	0.366138136
ELOVL5	0.728135657	0.659698427	0.132832233	3.276328372	0.610962661
ELOVL6	0.636438003	2.630534718	0.268988801	25.72491077	0.405788995

ELOVL7	0.512136213	0.888518889	0.674430946	1.170565825	0.400725868
ELP3	0.388061551	1.054437961	0.407234691	2.73021783	0.913040891
ELP4	0.407348162	4.474223997	0.01137231	1760.300221	0.623071441
ELSPBP1	0.711101025	0.011573999	6.39E-07	209.5830077	0.372710202
EMB	0.497518131	0.504871031	0.247026113	1.031853496	0.060932386
EMCN	0.658462186	0.092113896	0.00638949	1.327957196	0.079838439
EMD	0.741146885	0.610580603	0.070413498	5.294562631	0.654404802
EME1	0.180338692	0.671333199	0.315252376	1.429610999	0.301486941
EME2	0.722187101	0.011420006	1.90E-05	6.853080652	0.170603401
EMG1	0.405954079	1.189117324	0.575669632	2.456269937	0.639798966
EMID1	0.191000707	2.482218071	0.809126346	7.614888058	0.111916996
EMILIN1	0.724471319	20.07309742	1.72E-05	23491490.11	0.673956699
EMILIN2	0.664294923	0.897283967	0.271256652	2.968106086	0.859061777
EMILIN3	0.966471312	0.974855427	0.630783601	1.50660718	0.908718041
EML1	0.534377031	0.941466521	0.656691024	1.349735534	0.742776711
EML2	0.97504415	1.620850837	0.311488721	8.434197651	0.566031848
EML3	0.533217639	1.027335503	0.533164937	1.979534214	0.935769246
EML4	0.340139142	4.695693252	0.379813634	58.05356405	0.228029576
EML5	0.563478251	2.24504652	0.386245453	13.04930281	0.367797806
EMP1	0.81588152	0.706374592	0.346447217	1.440234009	0.338906771
EMP2	0.633114955	2.333404927	0.042197697	129.0302308	0.678971186
EMP3	0.55884581	5.161221739	0.000603933	44107.86175	0.722362246
EMR1	0.977234933	0.40796902	0.13466803	1.235918584	0.112873451
EMR2	0.286133309	1.192887862	0.890980241	1.597096531	0.236155957
EMR3	0.989975959	1.219594972	0.634902554	2.342740451	0.551155378
EMX1	0.38724479	1.870662545	0.842749644	4.152334423	0.123698304
EMX2	0.927382379	1.099512385	0.707670595	1.708319511	0.673051507
EN1	0.166379802	0.628810304	0.267427735	1.478539235	0.287552045
EN2	0.662429086	1.077154017	0.570968105	2.032093851	0.818485963
ENAH	0.801847515	13.15801155	0.533245338	324.6784464	0.115130817
ENAM	0.722211848	1.325516389	0.475623143	3.694087898	0.589965036
ENC1	0.741508552	0.971465891	0.901109635	1.047315377	0.450417264
ENDOG	0.850774667	0.67347084	0.23483588	1.931404057	0.462091248

ENG	0.347513175	1.481947863	0.599560939	3.662962887	0.394227189
ENO1	0.735556425	1.220477065	0.657928014	2.264023168	0.527394285
ENO2	0.123540406	0.315360836	0.054445061	1.826657095	0.197851446
ENO3	0.503998245	1.086081221	0.793008892	1.487464303	0.606819404
ENOSF1	0.136139206	0.272244416	0.040571164	1.826839908	0.180392295
ENPEP	0.467830149	3.107161081	0.334420736	28.86917271	0.31884028
ENPP1	0.610259098	1.014360696	0.796133128	1.29240649	0.908157827
ENPP2	0.825297645	0.011275519	8.81E-06	14.42706309	0.219169762
ENPP3	0.681938788	0.00067662	8.60E-08	5.321130135	0.110779192
ENPP4	0.49668255	0.978791483	0.766515152	1.249854963	0.863542936
ENPP5	0.148233679	0.927225777	0.55598803	1.546342	0.772159205
ENPP6	0.638876772	0.999013072	0.867072366	1.151030937	0.989098861
ENPP7	0.717601175	0.865050624	0.000278077	2691.030724	0.971818201
ENSA	0.595405411	2.257766854	0.035840311	142.2284302	0.70004597
ENTPD1	0.77023322	0.931065435	0.29025603	2.986614413	0.904398942
ENTPD2	0.5639757	1.119441695	0.275562289	4.547609603	0.874646264
ENTPD3	0.40368864	0.289966377	1.01E-06	83622.39125	0.846958381
ENTPD4	0.394869362	1.176818786	0.460098383	3.010013739	0.734011201
ENTPD5	0.29003683	4.161233149	0.984715347	17.58463638	0.052499233
ENTPD6	0.426744845	7.979533377	2.93E-05	2176309.509	0.745010255
ENTPD7	0.35413476	0.5698673	0.226432891	1.434194204	0.232401259
ENTPD8	0.782959407	0.106678804	0.000294038	38.70377763	0.456750396
ENY2	0.611653937	0.995645753	0.474495687	2.089187517	0.990792554
EOMES	0.092078972	1.256562042	0.918602627	1.718858753	0.153062215
EP300	0.667410125	2.071772589	0.001572741	2729.147424	0.842462896
EP400	0.122892455	1.312956593	0.975294149	1.767523179	0.072646928
EP400NL	0.916437522	0.988251592	0.275885511	3.540023554	0.98551652
EPAS1	0.732886181	1.918158146	0.249744844	14.73235891	0.531173867
EPB41	0.901986167	0.828079007	0.524292874	1.307885106	0.418539386
EPB41L1	0.383561142	1.151846091	0.399323411	3.322493449	0.793668914
EPB41L2	0.221985448	0.933887871	0.788140089	1.106588243	0.42948728
EPB41L3	0.899934813	0.974069734	0.72934675	1.300906388	0.858745944
EPB41L4A	0.747450269	1.248782735	0.570435711	2.733802055	0.578381547

EPB41L4B	0.080165314	1.351025314	0.993131189	1.837893542	0.05535636
EPB41L5	0.909724504	0.914143794	0.62936267	1.327785895	0.637399929
EPB42	0.361753895	1.134147661	0.865940201	1.485426957	0.360508925
EPC1	0.870560595	1.36545369	0.002016209	924.7373677	0.925376227
EPC2	0.511576572	0.989666211	0.100095675	9.785030257	0.992910348
EPDR1	0.146968002	0.027225758	1.19E-05	62.51701354	0.361434542
EPGN	0.157934824	0.007020878	4.49E-05	1.097559567	0.054373514
EPHA1	0.16013598	0.031968718	0.000756605	1.350769589	0.07145912
EPHA10	0.116666202	1.29580935	0.635234993	2.643308209	0.4761917
EPHA2	0.941749052	0.884826829	0.453319277	1.727079694	0.719896888
EPHA3	0.725710542	1.449183294	0.676740264	3.103306148	0.339614157
EPHA4	0.743346004	0.874119825	0.635852664	1.201670625	0.407354213
EPHA5	0.373788812	0.719907219	0.257401125	2.013458192	0.531138898
EPHA6	0.275491125	1.383901445	0.121182959	15.80406375	0.79372007
EPHA7	0.689325171	1.19677778	0.245066829	5.844434611	0.824307272
EPHA8	0.875602378	0.957117092	0.472436499	1.939039704	0.903157481
EPHB1	0.833445997	1.01776114	0.890273795	1.163504693	0.796538729
EPHB2	0.054489448	1.488267113	0.818088786	2.707455522	0.192805948
EPHB3	0.909635976	1.065836533	0.102300238	11.10464192	0.957474798
EPHB4	0.083022817	0.008303296	2.23E-05	3.097174015	0.112787487
EPHB6	0.737481699	1.010237593	0.758178221	1.346095109	0.944549351
EPHX1	0.857586477	1.52660195	0.480818057	4.84697585	0.472950201
EPHX2	0.234809789	1.648137397	0.320682165	8.470558009	0.549680435
EPM2A	0.982900038	0.734422768	0.280324244	1.924117569	0.529913929
EPM2AIP1	0.439093499	0.931293454	0.170692958	5.081097114	0.934467757
EPN1	0.157275404	0.260638901	0.00711836	9.543298133	0.464190625
EPN2	0.749063371	0.579508413	0.20939931	1.60377797	0.293504298
EPN3	0.168399559	1.005494463	0.463004932	2.183603339	0.988950846
EPO	0.447081009	1.1003964	0.769851812	1.572864048	0.599649079
EPOR	0.763570826	0.616788005	0.113373815	3.35551419	0.576057174
EPRS	0.395619042	0.051786243	0.000106851	25.09860208	0.348023366
EPS15	0.144527353	1.681851034	0.931950058	3.035165753	0.084348925
EPS15L1	0.820305656	0.519158945	0.040592937	6.639726824	0.614166946

EPS8	0.511668555	5.675775597	0.061414995	524.5368629	0.452168032
EPS8L1	0.240182574	0.497924475	0.150991967	1.641999827	0.252050394
EPS8L2	0.772401663	1.734216424	0.071305041	42.17803627	0.735269903
EPS8L3	0.865152118	0.1249457	0.000231761	67.35997632	0.516922289
EPSTI1	0.081153418	0.839536185	0.645655819	1.091635798	0.191713885
EPX	0.934317671	0.909569364	0.524440069	1.577523299	0.735832781
ERAL1	0.379559987	0.736308398	0.428365196	1.26562583	0.268034429
ERAS	0.598819272	1.443415744	0.000873995	2383.821644	0.922660528
ERBB2	0.176514611	0.450965359	0.058012405	3.505625316	0.446587776
ERBB2IP	0.371220746	0.455504919	0.036424665	5.696270102	0.541794302
ERBB3	0.353284645	3.221313212	0.006391911	1623.436177	0.712530499
ERBB4	0.764282113	0.65446641	0.050473975	8.486081801	0.745733598
ERCC1	0.164659729	0.287063828	0.062390013	1.320814614	0.109010396
ERCC2	0.287782459	0.452504202	0.168636799	1.214207425	0.115357928
ERCC3	0.248776591	0.614571972	0.0017522	215.5568891	0.870655625
ERCC4	0.681241027	0.64598185	0.03869683	10.78363647	0.760936155
ERCC5	0.770852613	0.048282321	9.84E-06	236.9427203	0.484582504
ERCC6	0.956581517	4.873239963	0.004847461	4899.156286	0.653416318
ERCC8	0.983333725	0.109548782	0.001219745	9.838891125	0.335221465
EREG	0.430066216	0.576849043	0.172442705	1.929654371	0.371852236
ERF	0.734161439	0.820685827	0.647258051	1.040582231	0.102778049
ERG	0.665756148	1.728766941	0.665514964	4.49071065	0.261045256
ERGIC1	0.956075111	0.095378727	0.000516204	17.6230899	0.377521548
ERGIC2	0.873307665	1.526498302	0.098609856	23.63046811	0.76218705
ERGIC3	0.482495054	0.957616885	0.865622977	1.059387427	0.400672355
ERH	0.611338175	9.672598111	0.09082362	1030.119193	0.340697568
ERICH1	0.969777593	2.218576335	0.099371413	49.53216241	0.615047457
ERMAP	0.980314951	0.881542625	0.509622628	1.524887943	0.65203329
ERN1	0.843609742	1.003120491	0.430060721	2.339787549	0.994247248
ERN2	0.855579467	4.41205668	0.429339754	45.33995273	0.211778899
ERO1L	0.33749368	0.368039044	9.69E-05	1397.808897	0.812119771
ERO1LB	0.999796927	0.952846292	0.646857868	1.403578902	0.806907395
ERRFI1	0.922043823	0.032571425	3.08E-08	34455.67122	0.628507776

ESAM	0.607967529	0.997661716	0.868872098	1.145541332	0.973518158
ESCO1	0.671651737	342.4185654	0.005091983	23026484.36	0.303482764
ESCO2	0.701254859	0.024306185	2.95E-05	20.00830233	0.277826486
ESD	0.774007729	0.953314849	0.787376473	1.154224481	0.624137577
ESM1	0.152194119	1.652737408	0.824276457	3.313865046	0.156916784
ESPL1	0.756214223	0.712740667	0.206538889	2.459581637	0.59206318
ESPN	0.548900715	1.47587329	0.663352825	3.283625075	0.340081294
ESPNP	0.559307631	0.783061524	0.454250267	1.349884404	0.378777704
ESR1	0.502991329	1.225582656	0.661335314	2.271242464	0.518106708
ESR2	0.075102831	2.025186083	0.742335162	5.524968887	0.16817609
ESRRA	0.717551799	0.071228821	0.000100877	50.29421236	0.429906656
ESRRB	0.733267427	1.375398051	0.393435443	4.808208905	0.617674691
ESRRG	0.20988101	1.129917048	0.841426763	1.517318669	0.416752216
ESX1	0.687854435	0.048344866	0.001010203	2.313620447	0.12479621
ETF1	0.280981034	2818.977699	0.617837646	12862012.09	0.064609387
ETFA	0.288982554	0.809560078	0.365240482	1.794399999	0.602902278
ETFB	0.360087093	0.002036076	5.90E-08	70.32310859	0.245131883
ETFDH	0.530090184	0.11675986	0.000486518	28.02129105	0.442466822
ETHE1	0.595765107	0.970450417	0.251324135	3.747248595	0.965291267
ETNK1	0.463202532	0.236449792	0.014351458	3.895667179	0.313110771
ETNK2	0.596581723	0.117298107	0.000122845	112.0020006	0.540440952
ETS1	0.134311696	2.102932835	0.643518535	6.872104321	0.21856532
ETS2	0.421009115	0.832314624	0.472945753	1.464750721	0.524482483
ETV1	0.832586268	0.953686148	0.439703199	2.068479993	0.904447749
ETV2	0.973111966	1.058329049	0.609147243	1.838735035	0.840580802
ETV3	0.855561794	525.5648057	0.3698765	746785.3869	0.090755989
ETV4	0.412749021	0.891191786	0.611510016	1.298789519	0.548856143
ETV5	0.058285808	3493.402806	0.067084476	181917843.1	0.140919833
ETV6	0.956597388	0.239522056	0.021545953	2.662718902	0.244834962
ETV7	0.32190043	0.739605523	0.419111697	1.305180301	0.297929839
EVC	0.83163994	0.897632419	0.64795466	1.243519043	0.516079482
EVC2	0.429095403	0.482172316	0.07475911	3.109857002	0.443084175
EVI2A	0.903872496	1.126581064	0.920607917	1.378637822	0.247282541

EVI2B	0.840088981	3.664097237	0.546441079	24.56917876	0.18105421
EVI5	0.387037537	0.977607784	0.492649776	1.939952127	0.94835801
EVI5L	0.243038877	0.76626768	0.376784026	1.558362662	0.462303263
EVL	0.966818484	1.199088692	0.250128232	5.748306304	0.820390144
EVPL	0.869053299	0.011763897	1.14E-06	121.8448758	0.346284591
EVX1	0.18453114	0.561060712	0.204019504	1.542936416	0.262837076
EWSR1	0.939842786	1.403835032	0.464589037	4.241927033	0.547693423
EXO1	0.21945087	0.445548691	0.146419644	1.355785535	0.154482674
EXOC1	0.135862529	0.320540028	0.075270165	1.365028353	0.123795476
EXOC2	0.236816319	0.00223538	1.84E-06	2.713716741	0.092096423
EXOC6	0.798198112	1.106510323	0.484225267	2.528503113	0.810301718
EXOC7	0.930268533	0.979890089	0.717627372	1.337998834	0.898286946
EXOC8	0.451969772	9.215322349	0.004389878	19344.99499	0.569324507
EXOSC1	0.10106598	9.764350745	0.182197451	523.2924224	0.261957379
EXOSC10	0.532008378	1.944205707	0.850227639	4.445792699	0.115145111
EXOSC2	0.230041279	1.034772393	0.904440143	1.18388587	0.618727983
EXOSC3	0.390481233	0.000209298	2.41E-08	1.815524358	0.067090842
EXOSC4	0.717122985	0.854456364	0.487039468	1.499048285	0.583398139
EXOSC5	0.25916894	11.95593176	0.116099897	1231.218184	0.294029903
EXOSC6	0.311430002	0.270216428	9.53E-06	7664.502333	0.802477683
EXOSC7	0.863845952	0.774672087	0.273072149	2.197649398	0.6312879
EXOSC8	0.063046414	0.516264438	0.161042797	1.655019506	0.265997988
EXOSC9	0.969677419	0.913668652	0.267532307	3.120334946	0.885439346
EXPH5	0.102110499	0.610418026	0.235049078	1.585244109	0.310707898
EXT1	0.450468617	0.850878279	0.218994394	3.305992594	0.815604544
EXT2	0.284102083	2.663505208	0.621978511	11.40595674	0.186804125
EXTL1	0.396005537	0.623140737	0.196038781	1.980752867	0.42277879
EXTL2	0.341444296	0.701415662	0.140572427	3.499860844	0.665414508
EXTL3	0.760995299	0.054553572	5.72E-07	5205.236205	0.619060008
EYA1	0.558000016	11.43622754	0.009447961	13842.91228	0.501076402
EYA2	0.870954817	1.268910505	0.219570083	7.333120479	0.790171643
EYA3	0.256855742	0.009123592	3.48E-07	239.3259239	0.365589386
EYA4	0.573664275	1.20837011	0.414947667	3.518897532	0.728543929

EZH1	0.766764331	0.377775168	0.042938587	3.323678955	0.380267506
EZH2	0.593567571	0.914286578	0.493555251	1.693670456	0.775731611
F10	0.120372287	1.999386266	0.836241642	4.780371174	0.119269216
F11	0.390774894	0.704123323	0.322384229	1.537884332	0.378793763
F11R	0.139500506	2.206362754	0.058101761	83.78466507	0.66976988
F12	0.780608682	1.059038896	0.45205782	2.481017545	0.894933627
F13A1	0.309841382	4.408423905	0.79200906	24.53785228	0.090314984
F13B	0.29255878	0.529391289	0.194813608	1.438580908	0.212403293
F2	0.526684856	22.01659434	0.131140503	3696.267873	0.2368891
F2R	0.810198265	1.300099055	0.332082383	5.089874207	0.706261015
F2RL1	0.420237312	1.655052643	0.742403215	3.689638185	0.218039857
F2RL2	0.174665214	1.117881729	0.793788622	1.574297648	0.523520577
F2RL3	0.543911713	1.0717418	0.11873709	9.673729438	0.950784182
F3	0.739531377	0.836299203	0.635790301	1.100042507	0.201174689
F5	0.787439889	1.091636219	0.654471144	1.820813102	0.736950942
F7	0.776669453	1.039228079	0.840886022	1.284353612	0.721758678
F8	0.104539021	1.398143625	0.807170349	2.42180055	0.231816468
F8A1	0.423060727	1.006355473	0.592313164	1.709824125	0.981310452
F8A3	0.970571815	1.121368786	0.764238959	1.645385829	0.558178661
F9	0.302146433	2.637057924	0.003906309	1780.216403	0.770500584
FA2H	0.157890684	3.542521473	0.891332444	14.07943632	0.072404651
FAAH	0.220846928	0.125909085	0.004785845	3.31249734	0.214212288
FABP1	0.192601785	2.802095545	0.830205116	9.457589814	0.096885311
FABP2	0.298779704	0.882077454	0.498256162	1.561567509	0.666780372
FABP3	0.806207466	1.14408198	0.334878438	3.9086529	0.829977216
FABP4	0.945774229	10.1594554	0.020920853	4933.571995	0.46256491
FABP5	0.399298918	1.012437816	0.463000628	2.2138854	0.975296769
FABP6	0.165714058	0.561599001	0.190632378	1.654458919	0.295264817
FABP7	0.270640484	1.562260702	0.63656034	3.834135349	0.330089994
FADD	0.107706776	0.206209093	0.021816453	1.949088191	0.168310807
FADS1	0.611842367	1.710383894	0.616545103	4.744848428	0.302552121
FADS2	0.122515669	1.982939978	0.556692779	7.063233264	0.290862888
FADS3	0.143011462	3.02517126	0.333524542	27.43924358	0.325139951

FADS6	0.902450201	0.82428184	4.02E-06	169211.9979	0.975298735
FAF1	0.871037142	1.543815342	0.229481991	10.38585118	0.655230418
FAH	0.640204726	1.336799952	0.428748089	4.16802817	0.616856194
FAHD1	0.790433935	1.206215045	0.560813402	2.594365133	0.631358859
FAHD2A	0.76737254	1.317894978	0.373151556	4.654535528	0.668093261
FAIM	0.147119101	0.773065665	0.385651368	1.549665243	0.468195589
FAIM2	0.095422046	0.784503723	0.317430835	1.938835249	0.599064252
FAIM3	0.633535851	0.61568218	0.146303202	2.59095181	0.508281916
FAM101A	0.331432455	0.029644182	0.000759334	1.157299852	0.059859181
FAM101B	0.505013215	0.001047193	4.01E-07	2.735571782	0.087399426
FAM102B	0.11653494	0.958742813	0.733482629	1.253182755	0.757827215
FAM103A1	0.374340274	0.812654302	0.167895106	3.933450051	0.79653603
FAM104A	0.237744108	1.202362791	0.638705865	2.263446073	0.568016219
FAM105A	0.562389879	1.334076783	0.705805045	2.521604053	0.374888304
FAM106A	0.112540958	0.913908038	0.406554767	2.054404402	0.827559995
FAM107A	0.86823769	1.183819482	0.577364855	2.427284156	0.645072628
FAM107B	0.092829451	1.142008128	0.569376524	2.290545027	0.70845182
FAM109A	0.875136419	1.177801067	0.288801934	4.803345097	0.819505057
FAM109B	0.518076291	3.123389184	0.725798822	13.44113507	0.126126004
FAM111A	0.950227184	3.164916336	0.000112031	89410.21515	0.82561457
FAM111B	0.798445318	0.760327365	0.394309993	1.466099547	0.413414669
FAM19A1	0.94795578	1.046107078	0.181720407	6.02210856	0.959745101
FAM19A2	0.525756673	1.03018988	0.878453228	1.208136251	0.714465345
FAM19A3	0.445135671	1.207132193	0.499718871	2.915975798	0.67569828
FAM19A4	0.351773156	1.128472303	0.782393719	1.627632877	0.517774882
FAM19A5	0.670966781	1.044023553	0.537187555	2.029058879	0.898881998
FAM20A	0.79462424	1.195645541	0.264192665	5.411082316	0.816561881
FAM20B	0.568027845	0.585672232	0.001240395	276.5343995	0.864777078
FAM20C	0.969463997	51.09696549	0.000403843	6465131.651	0.511652468
FAM21C	0.52872352	1.142752251	0.914341317	1.428222352	0.240852572
FAM24A	0.465930864	33.68025809	0.096050885	11809.9879	0.239464741
FAM24B	0.677206403	1.277659558	0.585022047	2.790346031	0.538680357
FAM27L	0.746843408	0.613669877	0.133225237	2.826722079	0.530935212

FAM32A	0.563052278	1.582322444	0.522805181	4.789057968	0.416701617
FAM35A	0.884008869	0.077794731	0.000410793	14.73251897	0.339832999
FAM3A	0.906670569	0.995633561	0.732171937	1.35389809	0.977738407
FAM3B	0.501043149	0.353804389	0.014002086	8.939921385	0.52832686
FAM3C	0.771001499	1.086780158	0.192512884	6.135127631	0.924920699
FAM3D	0.649811272	1.029019345	0.792648382	1.335877086	0.829898343
FAM41C	0.479348484	71.23089133	0.042867403	118361.2622	0.259530736
FAM43A	0.752543866	0.41865422	0.000692923	252.9449039	0.789863096
FAM43B	0.283478981	0.917925953	0.690767787	1.219784812	0.554945906
FAM45A	0.80246864	1.935560663	0.217771012	17.20336899	0.553541931
FAM45B	0.189501616	1.217734096	0.411421776	3.604272831	0.721984632
FAM46A	0.933377599	0.648337737	0.000697428	602.7029939	0.901103693
FAM46B	0.337586484	0.268273626	4.11E-05	1749.904689	0.769053949
FAM46C	0.658369826	0.618749621	0.291290466	1.31432758	0.211704714
FAM46D	0.587107436	0.903411215	0.217233007	3.757034129	0.888904417
FAM47A	0.973371994	0.609192761	0.106755864	3.476303824	0.577004716
FAM47B	0.23296316	0.475888404	0.146000806	1.551154272	0.218038123
FAM49A	0.699998168	1.126564787	0.343149399	3.698529629	0.844230046
FAM49B	0.496471948	0.946777916	0.798994796	1.121895194	0.52763695
FAM50A	0.950274573	0.002980221	1.60E-06	5.541892867	0.129987381
FAM50B	0.194364849	0.54721131	0.18349791	1.63184539	0.279466332
FAM53A	0.765060152	0.610963071	0.202922753	1.839497383	0.380944183
FAM53B	0.593541849	0.050657459	4.11E-09	624115.4304	0.720300066
FAM53C	0.443131062	1.07660451	0.728962633	1.590036606	0.710638442
FAM57A	0.489202458	0.02023682	1.07E-05	38.41444888	0.311216198
FAM57B	0.422879256	0.761600204	0.346973752	1.671696682	0.497175984
FAM58A	0.914149449	1.079966362	0.496043309	2.351261113	0.846333321
FAM60A	0.890023026	1.20305624	0.645905568	2.240798637	0.560193953
FAM63A	0.283646121	5.185891348	0.214783581	125.2119407	0.310981025
FAM63B	0.692685209	1.627451929	0.488874877	5.417745746	0.427380591
FAM64A	0.793416691	0.860178467	0.515055084	1.436558959	0.564892102
FAM65A	0.357284782	0.890723229	0.736492312	1.077252073	0.232912564
FAM66E	0.855813857	0.490070098	0.005545346	43.30995789	0.755107707

FAM69A	0.611267234	0.431008356	0.057151539	3.250449718	0.414246397
FAM69B	0.463018031	0.065226119	0.000146458	29.04888556	0.380327891
FAM71A	0.763656548	0.051428013	2.52E-05	105.0258138	0.445391584
FAM71B	0.469786517	0.829504212	0.516629667	1.331857773	0.439080785
FAM71C	0.980170731	1.976876272	0.311841577	12.53213195	0.46950352
FAM72A	0.796030213	44.87577028	0.001546872	1301875.139	0.468104332
FAM73A	0.931431093	0.595478198	0.234160392	1.514322218	0.276343224
FAM73B	0.511298401	1.246891151	0.631809051	2.46077124	0.524674577
FAM76A	0.427457124	0.887446382	0.719827723	1.094096624	0.263575373
FAM76B	0.133762508	2.054468538	0.345988922	12.19935294	0.428240845
FAM78A	0.239723427	2.184072111	0.103087439	46.27305747	0.616055706
FAM78B	0.364108036	0.955328873	0.368165018	2.478924425	0.925160532
FAM81A	0.012761385	1.743021047	1.00547449	3.021580757	0.047769431
FAM81B	0.955193758	0.603900577	0.000347226	1050.311814	0.894600272
FAM83A	0.456034198	0.091000033	3.27E-05	253.3693893	0.553661805
FAM83E	0.218371584	0.401007232	0.070108703	2.293678159	0.304434239
FAM83F	0.709788302	1.620442551	0.977967422	2.684991341	0.061000316
FAM84A	0.513827863	0.637219532	0.117840909	3.445736589	0.600754944
FAM84B	0.124819674	1.169624514	0.728753702	1.877206936	0.51627111
FAM86B1	0.208630175	0.005585402	3.04E-06	10.25839565	0.176107871
FAM87B	0.751444274	0.241268352	0.000274125	212.3497667	0.681055994
FAM89A	0.276020266	1.370445259	0.681984272	2.753905458	0.376135815
FAM89B	0.05757041	2.538987343	0.681083985	9.464995315	0.165172333
FAM8A1	0.659570521	1.060605137	0.814738879	1.380667211	0.661905807
FAM90A1	0.406363201	0.36054832	0.107652179	1.207547235	0.098095084
FAM91A1	0.100505953	2.082103832	0.568895522	7.620303194	0.267916555
FAM92A1	0.077708515	1.302696771	0.848540415	1.999926989	0.226645716
FAM92B	0.674859664	0.58964621	0.135963573	2.557175023	0.480392046
FAM96A	0.28051067	0.025520499	0.000492385	1.322738385	0.068590816
FAM96B	0.164995061	3.310480556	0.84568233	12.95909957	0.085570052
FAM98A	0.285836993	1.654374499	0.471808466	5.800987438	0.431600772
FAM98B	0.692618647	2.19932115	0.396917574	12.18644333	0.36694489
FAM98C	0.73187146	0.71233312	0.22008207	2.305587525	0.571367118

FAM99A	0.101142944	2.363502971	0.694650142	8.041668681	0.168581011
FAM9A	0.633682052	0.221051609	0.000166686	293.1491915	0.680747984
FAM9B	0.692072813	2.560602119	0.000404437	16211.8862	0.833252435
FAM9C	0.337043404	0.010645141	3.45E-06	32.86166191	0.267824202
FANCA	0.17531582	0.386128538	0.101537389	1.468377795	0.162630031
FANCB	0.734773203	0.769751208	0.303435855	1.952692514	0.581652386
FANCC	0.238086683	0.124385907	0.000353635	43.75094544	0.485924919
FANCD2	0.831504749	0.432988556	0.02137858	8.769482768	0.58551562
FANCE	0.514374369	2.794725087	0.083687286	93.32944922	0.565871674
FANCF	0.328380223	2.750576888	0.740550885	10.21627733	0.130706691
FANCG	0.942710883	317.549338	0.19013129	530357.638	0.128131754
FANCL	0.35596923	1.064657868	0.755735467	1.499858648	0.72011235
FANCM	0.271555401	0.952084255	0.879444068	1.030724364	0.225276079
FANK1	0.65538162	0.892327285	0.732197775	1.087476652	0.258924764
FAP	0.135194552	0.600968965	0.300589367	1.201518536	0.149700952
FARP1	0.908118892	0.056276546	0.00036546	8.665930186	0.262844902
FARP2	0.582440126	0.851787946	0.332736082	2.180535099	0.73801282
FARS2	0.994359131	1.144822943	0.336365811	3.896411372	0.82865236
FAS	0.774165749	0.787428157	0.456362474	1.358663646	0.39051556
FASLG	0.926576384	1.705113318	0.170995976	17.00280614	0.649261326
FASN	0.106872385	1.205294783	0.440110022	3.300846249	0.71640722
FASTK	0.06295877	383.1567153	0.237648819	617756.3556	0.114423629
FAT2	0.965757203	0.107863514	0.001788708	6.504437438	0.287009635
FAT3	0.7613604	0.801815671	0.33390943	1.925397463	0.621175881
FAT4	0.655862108	1.209395389	0.459192071	3.185240558	0.700396058
FATE1	0.025137241	0.608672529	0.370025073	1.001235526	0.050571088
FAU	0.429353034	1.16556021	0.5351346	2.538670841	0.6996939
FBF1	0.872787621	1.236434841	0.234492017	6.51950174	0.802435793
FBL	0.316241467	3.173047027	0.704143271	14.29854953	0.132763635
FBLIM1	0.662009438	0.652363997	0.245006074	1.737013197	0.392615947
FBLN1	0.726524497	0.836149618	0.336932559	2.075033016	0.699589471
FBLN2	0.786885878	1.351538625	0.811908556	2.249830529	0.246626336
FBLN5	0.368311401	0.533867811	0.270285153	1.054496842	0.070735914

FBN1	0.556360327	1.726514212	0.295689913	10.08100442	0.544127681
FBN2	0.494691744	1.710264137	0.293796015	9.955898888	0.550437447
FBN3	0.741175577	1.074760734	0.54907157	2.103752405	0.83335572
FBP1	0.306674446	1.212439018	0.227769705	6.45392402	0.821354213
FBP2	0.305277069	415.0355406	0.075524469	2280777.378	0.170057099
FBXL12	0.758774431	1.010382478	0.807411023	1.264378021	0.928068411
FBXL13	0.196539061	2.435215288	0.592940374	10.00146686	0.216894642
FBXL14	0.780816396	0.994466918	0.34062466	2.903384773	0.991901771
FBXL15	0.567423727	0.922342531	0.70251709	1.210953807	0.560583854
FBXL16	0.585727393	1.196855451	0.943674439	1.517963095	0.138372235
FBXL17	0.474605022	0.137057298	0.007980173	2.353921669	0.170726949
FBXL18	0.762573887	0.532599529	0.120053934	2.362790191	0.407225623
FBXL19	0.192508017	4.362907767	0.005905962	3223.008422	0.662007729
FBXL2	0.528000243	0.132953981	3.73E-05	474.1444244	0.628736643
FBXL20	0.599225616	1.653809637	0.685605774	3.989298833	0.262799165
FBXL21	0.964895718	0.8001239	0.015070253	42.48092408	0.912384422
FBXL22	0.059852549	1966.772621	0.2309507	16749005.51	0.100474375
FBXL3	0.956540746	3.623162296	0.351432607	37.353691	0.279489386
FBXL4	0.14301015	0.332571951	0.071250198	1.552333954	0.1613578
FBXL5	0.00906649	0.189770359	0.077269495	0.466067355	0.000288681
FBXL6	0.655485088	0.193476368	0.01835783	2.039081166	0.17162317
FBXL7	0.319392542	10.11677699	0.005236645	19544.79841	0.548860599
FBXL8	0.508166368	1.425741995	0.034507495	58.90720937	0.851806735
FBXO10	0.906157907	1.130869206	0.783947948	1.63131387	0.510610869
FBXO11	0.681968809	0.453048769	0.074763729	2.745357804	0.389061665
FBXO15	0.890081897	0.083588691	0.000255272	27.37106343	0.400946073
FBXO16	0.203547358	320.6109334	0.131858644	779557.3137	0.14688431
FBXO17	0.274814322	0.814971895	0.256622591	2.588155572	0.728567257
FBXO18	0.474018731	0.55487928	0.067292034	4.575445235	0.584242836
FBXO2	0.356855183	0.000216566	1.03E-08	4.537151753	0.096499403
FBXO21	0.513456567	0.777099036	0.239443516	2.522026582	0.674588921
FBXO22	0.401393102	64.44590038	0.000369627	11236396.77	0.498707622
FBXO24	0.939970527	0.792237212	0.449027771	1.397775017	0.421424766

FBXO25	0.824376035	0.183379159	0.005253025	6.401629003	0.349401614
FBXO27	0.589057905	0.170089418	0.024683542	1.172052613	0.07205692
FBXO28	0.712816054	1.251554534	0.459455941	3.40922515	0.660757003
FBXO3	0.95142071	1.029375897	0.652396382	1.624188557	0.900976155
FBXO30	0.760577221	0.270281035	0.000493879	147.9144227	0.684229111
FBXO31	0.40836574	0.77278879	0.263062308	2.27019415	0.63921665
FBXO32	0.553830415	0.382251814	0.000225511	647.9345337	0.799886096
FBXO33	0.972997412	0.629540511	0.238355747	1.662730013	0.350368759
FBXO34	0.780207025	21.66194245	0.02415293	19427.85997	0.375288725
FBXO36	0.582821853	0.000478071	5.39E-09	42.42629453	0.188423807
FBXO38	0.189263275	0.006674916	4.34E-05	1.027563143	0.051249693
FBXO39	0.054227946	0.528401361	0.234520561	1.190548058	0.123771303
FBXO4	0.530673552	0.913410395	0.287641232	2.900552689	0.877902246
FBXO40	0.174805293	0.6457591	0.056479408	7.383307141	0.72499744
FBXO42	0.802048114	0.949090611	0.000265161	3397.083547	0.990014658
FBXO43	0.547693106	0.415527186	0.136234277	1.267396474	0.122712431
FBXO44	0.338458767	1.10471933	0.754140719	1.618271984	0.60914542
FBXO46	0.995884492	0.987777858	0.338821242	2.879704618	0.982028323
FBXO47	0.27325008	2321.286129	0.402253916	13395442.73	0.079452724
FBXO5	0.139029594	1.183697901	0.908056334	1.54301079	0.212446041
FBXO6	0.349984657	0.884722446	0.367837182	2.127935524	0.784445982
FBXO7	0.2676311	0.174173088	6.48E-06	4680.659232	0.736973653
FBXO8	0.248957567	6.255585366	0.032140672	1217.533594	0.495401037
FBXO9	0.603411562	1.180758719	0.640536006	2.176600753	0.594400863
FBXW10	0.937977264	1.013612166	0.88266426	1.163986884	0.848082522
FBXW11	0.70865933	0.957898708	0.851761765	1.07726124	0.472831074
FBXW12	0.336429637	0.40656731	0.091054444	1.815364198	0.238437889
FBXW2	0.53777021	0.204099215	0.016123247	2.583629055	0.219803826
FBXW4	0.358715975	0.380899827	0.08878541	1.634104946	0.193933982
FBXW5	0.480366855	4.96362156	0.625301169	39.40107614	0.129580959
FBXW7	0.077865799	0.056238041	0.001671222	1.892457361	0.108628281
FBXW8	0.084622192	7.697447213	0.00253232	23397.78922	0.617925752
FBXW9	0.487178065	3.619174005	0.298955009	43.81401909	0.312043936

FCAMR	0.464407067	3.996414698	0.000947553	16855.34376	0.744950213
FCAR	0.974073185	0.250972753	6.26E-06	10062.36278	0.798232082
FCER1A	0.473340853	1.040796959	0.430711291	2.515045075	0.929219285
FCER1G	0.74277451	0.005636403	1.56E-06	20.36226627	0.215365175
FCER2	0.667886021	2.182909125	0.800495117	5.952681218	0.127207411
FCGBP	0.316962676	3.567800387	0.786890563	16.17658185	0.099104135
FCGR1A	0.721899122	0.000929386	1.80E-09	480.9269825	0.29835715
FCGR2A	0.131995036	0.670028675	6.15E-06	73008.90249	0.946051841
FCGR2B	0.954777354	0.601041086	0.289119533	1.249484542	0.172741692
FCGR3A	0.2103766	2.883555797	0.207789855	40.0158807	0.430027512
FCGR3B	0.664775739	1.461498624	0.358098659	5.964775832	0.59693264
FCGRT	0.730984331	0.49824168	0.160117927	1.550387122	0.229033251
FCHO1	0.532379715	1167.187575	0.315544639	4317382.284	0.092028015
FCHO2	0.430982196	1.34222874	0.619453364	2.908335149	0.455640009
FCHSD1	0.798246746	0.961122452	0.788459005	1.171597206	0.694703938
FCHSD2	0.342874557	1.023709093	0.709162775	1.477771174	0.900439812
FCN1	0.570206552	0.623137625	0.03701813	10.48946837	0.742649969
FCN2	0.11942953	0.574343382	0.229576794	1.436862651	0.235921911
FCN3	0.310988002	0.816661404	0.078925477	8.450197247	0.865107601
FCRL1	0.531664815	27.40098244	0.00230708	325439.0244	0.489203222
FCRL2	0.675424212	0.052974975	7.09E-07	3959.453923	0.607859929
FCRL3	0.194682963	0.824873597	0.35483109	1.917578451	0.654653965
FCRL4	0.792098583	1.232326	0.511840998	2.966990482	0.641219743
FCRL5	0.707050985	0.899094505	0.591228849	1.367272471	0.618952204
FCRL6	0.82263693	0.717559087	0.000541225	951.344602	0.927907881
FDFT1	0.774153274	7.581240794	0.579953808	99.10308574	0.122453912
FDPS	0.61727538	0.685117068	0.251704038	1.86483062	0.459177363
FDX1	0.523131179	0.202155349	1.08E-05	3785.495486	0.75009494
FDXR	0.533205091	2.102363005	0.134579211	32.84259273	0.596217222
FECH	0.613494153	0.833262188	0.416893386	1.665475867	0.605681127
FEM1A	0.360540094	1.169609002	0.641370628	2.132909051	0.609294004
FEM1B	0.941575624	1.124029346	0.787959611	1.603434941	0.518860679
FEM1C	0.774520216	13.16427659	3.19E-06	54315055.9	0.740160464

FEN1	0.169851007	0.058132059	0.001935905	1.745610559	0.101209548
FER	0.390345069	1.227805013	0.737075077	2.045253189	0.430549735
FER1L4	0.773006687	0.810410949	0.173811187	3.77861701	0.788996196
FERD3L	0.604536356	0.79719859	0.392442083	1.619412443	0.530784583
FES	0.388568457	2.403548652	0.011529309	501.0747815	0.747542852
FETUB	0.915635574	1.248976361	0.511835291	3.04774207	0.625221002
FEV	0.997564812	0.455795108	0.082242848	2.526045558	0.368481874
FEZ1	0.272046056	0.535134679	0.186547145	1.535103223	0.244894113
FEZ2	0.819029313	1.106671963	0.879526631	1.392479534	0.387179098
FFAR1	0.287841749	0.33532371	0.100928806	1.11407233	0.074483604
FFAR2	0.391948292	2.203390436	0.203181332	23.89456439	0.515965606
FFAR3	0.364211009	1.817057435	0.508842055	6.488649458	0.357770921
FGA	0.350815659	1.431870552	0.709242716	2.890763956	0.316586386
FGB	0.994447913	0.925357524	0.317797306	2.694442434	0.886873383
FGD1	0.689147973	0.878121608	0.008288811	93.02873216	0.956432583
FGD2	0.913867092	0.091342605	1.29E-06	6489.321397	0.674575525
FGD3	0.363047313	2.234627077	0.748057813	6.67536397	0.14984318
FGD4	0.079704525	1.127616241	0.438567707	2.899252198	0.803147152
FGD5	0.679042295	1.247156462	1.99E-05	78257.63068	0.968741662
FGD6	0.874756392	0.000329115	1.16E-10	931.6473577	0.290072476
FGF1	0.885490827	0.017790602	3.99E-06	79.36510491	0.34734538
FGF10	0.92872683	1.017501274	0.693599054	1.492661843	0.929290603
FGF11	0.670524108	0.558555179	0.059344123	5.257199422	0.610655772
FGF12	0.709319039	0.999990759	0.790874677	1.264399466	0.999938404
FGF13	0.361907461	0.659131883	0.179576162	2.419334691	0.529816855
FGF14	0.347917975	0.053185087	7.97E-06	355.1087593	0.513762893
FGF16	0.94203578	0.913152519	0.436222124	1.911520479	0.809525448
FGF17	0.156478619	1.632666561	0.555988946	4.794340107	0.372432034
FGF18	0.304175379	0.113482692	0.01069597	1.204034921	0.070937741
FGF19	0.081892849	1.07448512	0.57100413	2.021908795	0.823749437
FGF2	0.499546376	1.78697347	0.400093686	7.981316104	0.447093166
FGF20	0.360162346	1.186103075	0.897905452	1.566802496	0.229474675
FGF21	0.400357014	2.706409028	0.006758051	1083.840584	0.744704299

FGF22	0.516965199	0.660595286	0.149307282	2.922738432	0.584764296
FGF23	0.47346114	5.156947064	0.283370269	93.84930572	0.267813339
FGF3	0.365948006	0.602759209	0.16376422	2.218547283	0.446402406
FGF4	0.160695576	0.409354704	0.060928063	2.750313479	0.358096891
FGF5	0.582386603	1.020728654	0.866611234	1.202254188	0.805935688
FGF6	0.05111798	0.659749495	0.386971081	1.124811173	0.126542463
FGF7	0.402937291	1.984264221	0.319144407	12.33706251	0.462355386
FGF8	0.553251376	3.170964719	0.22652287	44.38853022	0.391383595
FGF9	0.248687453	0.858640329	0.37838111	1.948467288	0.715466689
FGFBP1	0.476132864	0.317212456	0.000453941	221.6669717	0.731142718
FGFR1	0.223726288	0.563087606	0.226749232	1.398318528	0.215889947
FGFR1OP	0.192670786	1.60456454	0.932457579	2.761120099	0.087739436
FGFR1OP2	0.468042717	1.588749643	0.371076864	6.802163312	0.532681323
FGFR2	0.378713248	0.521289633	0.064422825	4.218114978	0.541416304
FGFR3	0.850348598	0.866052378	0.247152161	3.034756876	0.822149618
FGFR4	0.663527501	3.198205256	0.055207642	185.2735682	0.574562709
FGFRL1	0.143805651	1.432101842	0.608824649	3.368647585	0.410548081
FGG	0.436657428	0.436375582	0.018225088	10.44843531	0.608795708
FGL1	0.440828316	1.568125584	0.840389492	2.926045448	0.157484967
FGL2	0.090052544	0.023145088	5.56E-05	9.637850731	0.221051953
FGR	0.192057736	0.687681899	0.196792358	2.403072959	0.557511788
FH	0.896096171	1.006691819	0.873321637	1.160429759	0.926715302
FHIT	0.06993476	0.043548532	0.000710871	2.667817902	0.135539866
FHL1	0.855190017	0.777168088	0.482306947	1.252294292	0.300345365
FHL2	0.637845682	1.03326262	0.609317675	1.752175729	0.90334884
FHL3	0.771878724	0.987622682	0.24765527	3.938533429	0.98592044
FHL5	0.07671023	0.591276218	0.25379773	1.377504697	0.223319259
FHOD1	0.169895061	6.765643243	0.351213923	130.3306203	0.205262832
FHOD3	0.902675925	0.471121507	0.094776374	2.341886111	0.357625683
FIBCD1	0.551954549	0.010106306	9.10E-06	11.226632	0.199107113
FIBP	0.385375652	1.011445282	0.822777766	1.243375309	0.913963408
FIGF	0.60562664	1.283147887	0.470169765	3.501859587	0.626460309
FIGLA	0.796140514	13.64020464	0.045726031	4068.911708	0.368762211

FIGN	0.596483926	1.537125213	0.630549658	3.74713377	0.344345129
FIGNL1	0.718865243	0.711188516	0.170368178	2.968800351	0.640170042
FILIP1	0.256095276	1.706928437	0.392265023	7.427643355	0.476052769
FIP1L1	0.744585387	10.83191536	0.24275796	483.3225252	0.218910359
FIS1	0.791053168	6.115273618	0.654016132	57.17989142	0.112362556
FJX1	0.301690518	31.95327071	0.021945667	46524.51485	0.351218148
FKBP10	0.906078624	0.12520312	0.002118777	7.39852474	0.318100135
FKBP11	0.051862904	2.777762871	1.461632313	5.27900656	0.0018175
FKBP14	0.238170353	1.498467548	0.828100285	2.711513369	0.18135086
FKBP1A	0.052332022	0.78179038	0.24712563	2.473220604	0.675264605
FKBP2	0.126059109	25.42176121	0.620336819	1041.798461	0.087651408
FKBP3	0.238424405	1.197766296	0.884692593	1.621630058	0.243047853
FKBP4	0.623205495	2.189261842	0.583951292	8.207649301	0.245182421
FKBP5	0.251100684	0.246914859	0.013260917	4.597490977	0.348507394
FKBP6	0.407677716	1.176120351	0.50181134	2.756532128	0.708935163
FKBP7	0.955605934	1.4579801	0.322058028	6.600381885	0.624569154
FKBP8	0.650035868	1.322016138	0.265891679	6.573077708	0.732993059
FKBP9	0.932874253	1.13310351	0.604629791	2.123487105	0.69658476
FKBPL	0.65970302	0.574643721	0.21932544	1.505595545	0.259606088
FKRP	0.704056281	1.040100196	0.416610695	2.596689024	0.932876798
FLAD1	0.431479697	0.679043568	0.228422462	2.018628829	0.486222346
FLCN	0.473811915	3.966302266	2.91E-05	539720.657	0.819296142
FLG	0.181954316	0.71038562	0.329085517	1.533485076	0.383769482
FLG2	0.408871342	1.147101652	0.958960892	1.372154183	0.133228371
FLI1	0.285353667	0.495671931	0.230775399	1.064631085	0.07195606
FLII	0.299145098	1.581697283	0.71111487	3.518090257	0.260963841
FLJ13224	0.171629627	1.130125285	0.905324292	1.410746592	0.279689391
FLJ20021	0.692651265	0.617981017	0.306887755	1.244430682	0.177769318
FLJ26850	0.630922917	0.087081609	0.000128011	59.23867543	0.463267299
FLJ27354	0.485581815	0.000301407	5.33E-10	170.2978931	0.230256451
FLJ30679	0.335331958	0.732317578	0.337902392	1.587112278	0.429846493
FLJ33360	0.552827354	1.070137522	0.894701766	1.279973238	0.458074422
FLJ35934	0.922324337	0.821152638	0.077652465	8.683454608	0.869926342

FLJ39080	0.402763982	0.776946273	0.413304406	1.460534903	0.433213751
FLJ40194	0.928674757	1.3558856	0.447390047	4.109223649	0.590454256
FLJ40288	0.669704325	0.562134497	0.120583977	2.620540466	0.463324449
FLJ42102	0.653829753	16.2673153	0.037660314	7026.642093	0.36766674
FLJ42393	0.862486596	0.905180712	0.651569275	1.257505766	0.552563162
FLJ43879	0.707907895	28.9658765	0.04028594	20826.67037	0.31587068
FLJ45079	0.273533326	0.500091561	0.20711613	1.207494414	0.12337883
FLJ46284	0.608598094	1.391205951	0.181768722	10.64789356	0.750510126
FLNA	0.902192415	0.674705936	0.219351297	2.075338078	0.492481848
FLNB	0.207626049	0.977794586	0.789807133	1.210526232	0.836684582
FLNC	0.825976549	1.024874381	0.905626198	1.159824548	0.697051176
FLOT1	0.521271637	0.954764234	0.439596407	2.073662862	0.906878115
FLOT2	0.86160781	0.250664886	0.017788146	3.532289789	0.305335181
FLRT1	0.896906579	2.120592572	0.365361269	12.30812688	0.402152255
FLRT2	0.432745171	1.646603761	0.294500357	9.206453844	0.570103117
FLRT3	0.567900349	1.355489923	0.401327313	4.578190594	0.624279601
FLT1	0.480682311	0.004984112	1.36E-05	1.820277415	0.078238744
FLT3	0.451221157	17.65576312	0.014192458	21964.19966	0.429728238
FLT3LG	0.722075978	0.08563496	5.39E-05	136.1783978	0.513471599
FLT4	0.419821232	0.516434229	0.158605944	1.681553075	0.272596038
FLYWCH1	0.263866039	1.315515577	0.523615944	3.30505832	0.559598217
FMN2	0.474426761	0.871686442	0.349229881	2.175750971	0.768564579
FMNL1	0.376364675	1.438047016	0.790056242	2.617508867	0.234511173
FMNL2	0.927031889	3.015918297	0.788795896	11.53119992	0.106690324
FMNL3	0.954986086	3.053708297	0.418814617	22.26554181	0.270747657
FMO1	0.299930087	72.21681693	0.040800515	127823.5975	0.262040667
FMO2	0.509165983	3.405951069	0.766964028	15.12522396	0.107144531
FMO3	0.235298194	1.456235631	0.452643539	4.684971799	0.528412656
FMO4	0.827084795	2.227117449	0.667837254	7.427037196	0.192574401
FMO5	0.902257059	0.037845679	6.24E-06	229.4114162	0.461241074
FMOD	0.954013569	1.127879584	0.907064568	1.402449617	0.279020923
FMR1	0.257382349	0.576849074	0.196905992	1.689917363	0.315751514
FMR1NB	0.114381481	0.428905087	0.050251208	3.660799054	0.439058674

FN1	0.311607937	1.666942019	0.909553298	3.055011401	0.098280951
FN3K	0.473852278	0.959455559	0.733169374	1.255582956	0.762973046
FN3KRP	0.359784453	1.113880796	0.945589539	1.312123682	0.196871671
FNBP1	0.707628721	0.603950428	0.221481105	1.646894976	0.324511409
FNBP1L	0.775075063	0.193827879	6.34E-05	592.5377067	0.688624344
FNBP4	0.918891384	0.810939965	0.201727522	3.259959879	0.767825843
FNDC1	0.200996725	1.285490154	0.614459801	2.68932961	0.504877504
FNDC3A	0.643308312	0.692469871	0.420712533	1.139767618	0.148343413
FNDC3B	0.672007678	1.079902545	0.50242209	2.321135018	0.84390684
FNDC4	0.619979192	0.658895916	0.222398781	1.952096259	0.451533808
FNDC5	0.323102372	0.837616142	0.392455382	1.787721185	0.646886374
FNDC7	0.130018576	0.390249107	0.106453875	1.43061364	0.155701157
FNDC8	0.602698726	0.904674646	0.288913661	2.832805524	0.863423723
FNTA	0.94601894	0.003981861	5.59E-06	2.834368491	0.099134252
FNTB	0.661676796	0.707928712	0.238795542	2.098711971	0.533309612
FOLH1	0.806503133	0.666916639	0.009090382	48.9283931	0.853356063
FOLR1	0.883177559	0.178650427	0.009347944	3.41422403	0.252542183
FOLR2	0.749176841	0.00782268	7.47E-06	8.192720255	0.171572584
FOLR3	0.069363748	2.096597697	0.721470948	6.092721976	0.173778622
FOS	0.437569976	1.067691966	0.709959474	1.605677754	0.753056551
FOSB	0.103414075	2.945121748	0.973040082	8.914064564	0.055927945
FOSL1	0.963494523	1.682043456	0.447842145	6.317561269	0.441192176
FOSL2	0.709296423	0.04912836	0.000918139	2.628791697	0.137814977
FOXA1	0.167850106	1.831753468	0.538500513	6.230858999	0.33253477
FOXA2	0.506642502	0.911198886	0.668639009	1.241751376	0.555948774
FOXA3	0.330553439	0.814208137	0.251220777	2.638853751	0.731904709
FOXB1	0.779077484	0.119750818	0.000355763	40.3084494	0.474694167
FOXC1	0.453926367	0.899780663	0.46748579	1.731828559	0.751922064
FOXC2	0.69617124	3118.996455	0.010264294	947765025.7	0.211647145
FOXD1	0.514202392	1.114256415	0.229068906	5.420060639	0.893369608
FOXD2	0.055337465	0.039827644	0.000173561	9.139377249	0.245163693
FOXD3	0.435030859	1.017949376	0.774845835	1.33732529	0.898324978
FOXD4	0.172082061	1.144240079	0.94069575	1.39182659	0.177583195

FOXD4L1	0.77366845	2.568711471	6.38E-06	1033656.438	0.886070253
FOXD4L4	0.056187781	1.154721984	0.928833357	1.435545837	0.195230118
FOXE1	0.103877077	0.440662859	0.172971728	1.122632915	0.08588426
FOXE3	0.473418796	0.864359157	0.309145481	2.416715748	0.781113267
FOXF1	0.385416323	8.065251974	0.23027184	282.4847774	0.249902641
FOXF2	0.181394014	0.958128191	0.779457097	1.177755178	0.684589407
FOXH1	0.211271604	0.800946332	0.277878836	2.308614202	0.681108539
FOXI1	0.827852247	1.994159722	0.320506854	12.40745071	0.459287805
FOXJ1	0.50678819	0.920314214	0.003533698	239.6861051	0.976657127
FOXJ2	0.905671304	0.936236245	0.016561581	52.92600265	0.974467313
FOXJ3	0.118270672	27.67056989	0.127688426	5996.318247	0.2262945
FOXK2	0.845653009	0.856025736	0.650437448	1.126595744	0.267283904
FOXL1	0.810993996	1.5761571	0.383018163	6.486040211	0.528451359
FOXL2	0.31695026	1.775213141	0.54504287	5.781896926	0.340783061
FOXM1	0.162478934	1.880414827	0.492612422	7.177975558	0.355493589
FOXN1	0.157294665	0.856771731	0.328782887	2.232652089	0.751747312
FOXN4	0.367480148	0.083190602	0.004926965	1.404653044	0.084646899
FOXP1	0.372223849	0.823024186	0.333425088	2.031547222	0.672672728
FOXP2	0.221584851	0.956877023	0.833967019	1.097901495	0.529728039
FOXP3	0.921816975	1.481390513	0.701541289	3.128137842	0.302791152
FOXP4	0.691586173	0.7670057	0.217235171	2.708114622	0.680247368
FOXQ1	0.419862256	4.970882665	0.083489821	295.9603222	0.441838652
FOXR1	0.514058719	1.476635723	0.386133463	5.646889652	0.568998821
FOXR2	0.369872502	0.72261616	0.076849274	6.794782679	0.776309752
FOXRED1	0.462937543	1.1130487	0.426294114	2.906156496	0.826863424
FPGS	0.961787706	0.835787059	0.332658879	2.09986882	0.702734161
FPGT	0.368950455	0.683355605	0.367583819	1.270390205	0.22878946
FPR1	0.131859114	2.501821507	0.804264086	7.782407497	0.113248427
FRAS1	0.09581218	0.75952081	0.386717172	1.491715141	0.424460344
FRAT1	0.535153639	1.078166727	0.350611304	3.315476363	0.895526291
FRAT2	0.398619705	0.41841959	2.20E-05	7945.504071	0.86238602
FREM1	0.424624768	6.735296348	0.009677985	4687.361535	0.567894519
FREM2	0.158934741	1.178598788	0.012594621	110.2927279	0.943429682

FREM3	0.946615355	1.481222686	0.348289418	6.299418039	0.594778931
FRG1	0.641360835	1.011010433	0.880057609	1.161449075	0.877044112
FRG2	0.846115249	0.9635265	0.772786235	1.201345566	0.741311444
FRK	0.216065302	0.723005592	0.181000451	2.888042999	0.646226736
FRMD1	0.118427596	8.90E-05	6.63E-10	11.9405135	0.121552487
FRMD3	0.070252164	0.344922969	0.072693924	1.63661346	0.180289657
FRMD4A	0.893698636	0.773962616	0.000401197	1493.078788	0.947069854
FRMD4B	0.083708814	6.855826466	0.08937118	525.9229696	0.384643518
FRMD5	0.050826919	230.7991971	0.001819803	29271447.93	0.364070871
FRMD6	0.933017072	0.971457118	0.493827452	1.911049962	0.933148544
FRMPD1	0.459636891	0.884372861	0.390331421	2.00372124	0.768406574
FRMPD2	0.568272927	1.152794345	0.661508545	2.008945785	0.615840299
FRMPD4	0.68753413	0.773161869	0.258846811	2.309394012	0.644940506
FRRS1	0.736347545	1.153473838	0.333898415	3.984750557	0.821409381
FRS2	0.653219362	0.868893193	0.529529146	1.42574849	0.578080164
FRS3	0.369168655	102.9472439	0.002268129	4672632.658	0.396968989
FRY	0.149537521	0.775459667	0.339510902	1.771188173	0.546210972
FRYL	0.429904945	0.220441965	0.000267061	181.9609431	0.658999061
FRZB	0.919623154	1.32921107	0.054255487	32.56448641	0.861567022
FSBP	0.177457797	0.646444303	0.078726085	5.308154666	0.684661655
FSCN1	0.552117715	1.388726499	0.672367243	2.868315357	0.374891587
FSCN2	0.151860178	0.495567654	0.053617225	4.58038061	0.536081516
FSCN3	0.258637979	2.450516908	0.731511018	8.209080892	0.14619596
FSD1	0.522209174	0.859227626	0.669973071	1.101942966	0.231997784
FSD2	0.237824278	1.227121706	0.758394546	1.98554656	0.404505148
FSHB	0.333830348	0.743449668	0.388090217	1.424198254	0.371418162
FSHR	0.348649478	0.676492258	0.299216796	1.529465532	0.347712248
FSIP1	0.50799264	1.60E-05	2.23E-10	1.148459213	0.052904296
FSIP2	0.497019833	0.640805126	0.3484976	1.17828992	0.152134488
FST	0.282985348	0.72848797	0.368313163	1.440879055	0.36264362
FSTL1	0.831565214	1.424539376	0.000948723	2138.99391	0.924458464
FSTL3	0.058922093	1.448000016	0.945616515	2.217287889	0.088614133
FSTL4	0.270956666	1.196664347	0.933498703	1.53401987	0.156517305

FSTL5	0.88189591	1.303752976	0.703518298	2.416101797	0.399390919
FTCD	0.439693002	1.114534723	0.861043614	1.442653574	0.410155778
FTH1	0.842281344	1.464327872	0.490774476	4.369127214	0.494092475
FTHL17	0.067962974	1.011545166	0.389170698	2.62924117	0.981208933
FTL	0.701301342	0.256948624	0.004614185	14.30861549	0.507606887
FTMT	0.372308647	0.45844986	0.098503372	2.133696247	0.320206771
FTSJ1	0.736665562	0.951204209	0.286757464	3.155242883	0.934829345
FTSJ2	0.258796376	1.743220618	0.953577911	3.186753895	0.070991801
FTSJ3	0.168448832	0.063356665	2.88E-06	1392.786672	0.588607276
FUBP1	0.946995275	1.087272064	0.941359736	1.255801046	0.25510307
FUBP3	0.69295399	1.15659274	0.647377121	2.066348534	0.623179281
FUCA1	0.496237659	0.391050671	0.011738692	13.02705868	0.599657811
FUCA2	0.717140406	5.784835612	0.002435639	13739.44468	0.6580577
FUK	0.811176783	0.048548077	0.000564168	4.177682622	0.183208502
FUNDC1	0.600216155	0.996387336	0.326253184	3.042997803	0.994930625
FUNDC2	0.104016159	1.396595819	0.742994837	2.6251594	0.299553383
FURIN	0.995220066	0.884806185	0.376591563	2.078862254	0.778853027
FUT1	0.200192671	0.845844658	0.302349612	2.36631091	0.749752679
FUT10	0.3403529	0.000416467	2.85E-08	6.092191382	0.111680998
FUT11	0.071656184	0.322279997	0.057764621	1.798062472	0.196693826
FUT2	0.609844925	2.851236845	0.835020766	9.735747747	0.094483272
FUT3	0.693612982	1.111380012	0.390456904	3.16338505	0.843148929
FUT4	0.111740645	4.557114866	0.451884949	45.95704275	0.198339053
FUT5	0.901912245	15.59916893	0.296060652	821.906152	0.174401199
FUT6	0.242704943	0.269737917	0.044368486	1.639869865	0.154776878
FUT7	0.196176116	2.597871902	0.851987898	7.921401743	0.093276741
FUT8	0.945332862	1.377999788	0.47548974	3.993531839	0.554785078
FUT9	0.776336299	0.916127354	0.786471216	1.067158354	0.260537771
FXN	0.095015108	1.679933531	0.662801107	4.257954063	0.274293805
FXR1	0.711018641	0.76117809	0.375897723	1.541355665	0.448414147
FXR2	0.443771242	3.520280344	0.147655354	83.92769603	0.436692935
FXYD1	0.337986017	1.507781006	0.10283635	22.10700358	0.764387058
FXYD2	0.956385559	1.161177814	0.53104655	2.539012665	0.708127037

FXYD3	0.975424533	2.302294976	0.062502847	84.80513093	0.650408001
FXYD4	0.682721155	0.220842982	4.76E-05	1024.830541	0.725873392
FXYD5	0.787338186	0.172578578	0.000132241	225.22064	0.631231958
FXYD6	0.191310439	0.420925256	0.101362051	1.747972446	0.233581123
FXYD7	0.463520159	0.915869133	0.270031132	3.106368748	0.887846178
FYB	0.381489078	0.013281296	6.05E-07	291.4429003	0.396829648
FYCO1	0.510043054	1.210825423	0.396388813	3.698636685	0.737041606
FYN	0.392564562	1.765095763	0.630921621	4.938114256	0.279026784
FYTTD1	0.679926076	1.312784163	0.30361873	5.67620534	0.715623276
FZD1	0.852211417	1.22057325	0.214913312	6.932092971	0.822036613
FZD10	0.937646715	0.718788989	0.007973363	64.79795189	0.885685534
FZD2	0.685219922	12.50463289	1.25E-07	1253737038	0.788130308
FZD3	0.910602214	0.095907414	0.002096278	4.387887119	0.229426926
FZD4	0.568754967	0.916344768	0.393348141	2.134718955	0.839550087
FZD5	0.68064698	0.750062592	0.147147252	3.82333943	0.729275889
FZD6	0.44278266	0.266443113	0.012915516	5.496639048	0.391750094
FZD7	0.98074467	0.136271723	0.005925265	3.134034257	0.212802719
FZD8	0.968393983	1.227592745	0.002100015	717.6064424	0.949699496
FZD9	0.26047323	0.478584524	0.14878627	1.539410498	0.216364574
FZR1	0.392099356	1.217628964	0.01220271	121.4992663	0.933181584
G0S2	0.419329062	1.103017769	0.470457701	2.586094766	0.821566743
G3BP2	0.400601974	1.716748602	0.094629202	31.14499229	0.714756834
G6PC	0.767433391	0.533064494	0.164139975	1.731191656	0.295196174
G6PC2	0.69215823	2.642844599	0.664074176	10.51784248	0.167871577
G6PC3	0.213744132	24047.37405	0.802855978	720273889.8	0.055084332
G6PD	0.111768223	2.706666349	0.252599031	29.00265566	0.410583095
GAA	0.474373079	0.56032388	0.15970069	1.965945483	0.365751684
GAB1	0.661353471	1.267768095	0.45473881	3.534415593	0.650155032
GAB2	0.946626457	0.966213386	0.311819064	2.993942367	0.952502548
GAB3	0.722782348	0.718849269	7.65E-05	6756.75828	0.943619199
GABARAP	0.935960633	0.891885093	0.328624081	2.420574346	0.822283184
GABARAPL1	0.805495084	0.769240232	0.215131025	2.750558795	0.68653516
GABARAPL2	0.53024321	0.139487261	0.003649269	5.331669785	0.289313385

GABBR1	0.436326669	1.426392575	0.404587684	5.028812935	0.580655365
GABBR2	0.811276915	1.147117008	0.848910963	1.550077083	0.3715575
GABPA	0.995926377	0.438174726	0.000306249	626.9307693	0.823864608
GABPB2	0.232612156	0.224546493	0.000365127	138.0920805	0.648468614
GABRA1	0.826484557	0.432855469	0.000288959	648.4088682	0.822404113
GABRA2	0.363340849	0.532431343	0.137912913	2.055522779	0.360441559
GABRA3	0.745346091	0.578003906	0.148518028	2.249481225	0.429144553
GABRA4	0.283524217	1.246390777	0.907810883	1.711248452	0.173226551
GABRA5	0.214068972	0.46027175	0.158295192	1.33832292	0.154203417
GABRA6	0.702859089	0.063027043	9.57E-05	41.49588504	0.403826637
GABRB1	0.204202478	0.932194845	0.782833636	1.110053515	0.430650068
GABRB2	0.059400318	62.09055727	0.006497991	593296.8202	0.377275925
GABRB3	0.357208141	1.377717311	0.487913873	3.890245993	0.545172276
GABRD	0.102186083	3.56E-05	1.25E-10	10.17166701	0.110027495
GABRE	0.309306156	0.199340068	0.005967238	6.659104749	0.36765587
GABRG1	0.664629552	1.102746338	0.579647179	2.09791323	0.765660131
GABRG2	0.770556733	1.041402944	0.551717688	1.965715649	0.900395924
GABRG3	0.141552923	0.87621847	0.659936518	1.163382818	0.360909537
GABRP	0.974834248	0.023689421	2.45E-05	22.89685051	0.285883563
GABRQ	0.475912152	1.077142083	0.502939945	2.306905781	0.848337654
GABRR1	0.372099095	1.064109847	0.453621261	2.496200827	0.886416902
GABRR2	0.75992654	0.471369781	0.005037389	44.10806678	0.745344859
GABRR3	0.264997961	2.276434351	0.180346724	28.73439146	0.52484877
GAD1	0.166444102	1.40830825	0.813341234	2.438499418	0.221569804
GAD2	0.132749688	0.013493341	9.76E-05	1.864669229	0.086862862
GADD45A	0.148302249	0.712223878	0.350906117	1.445579964	0.347407405
GADD45B	0.145059486	0.837066628	0.046670836	15.01324175	0.903888079
GADD45G	0.523110684	0.362051188	9.30E-07	141005.9513	0.877064933
GADD45GIP1	0.661761045	0.053679182	8.28E-06	348.1990837	0.513708162
GADL1	0.254093284	1.258311556	0.331205993	4.780553507	0.73582339
GAGE1	0.703106183	0.834613607	0.206912335	3.366545896	0.799446959
GAK	0.17795646	0.417200111	9.65E-05	1804.058951	0.837840635
GAL	0.29343235	0.452240003	0.208181782	0.982415554	0.044986133

GAL3ST1	0.13485821	62.90959467	0.241044393	16418.62334	0.144613865
GAL3ST2	0.740571254	0.701053429	0.005113884	96.10618555	0.887498351
GAL3ST3	0.061933883	0.82831267	0.60281253	1.138167911	0.245334857
GAL3ST4	0.333832177	0.225448472	0.021563285	2.357109009	0.213515981
GALC	0.098523517	0.593284499	0.213229134	1.650742988	0.317332406
GALE	0.183029375	2.060506534	0.548645982	7.738482223	0.284252746
GALK1	0.702976749	1.086126944	0.941638041	1.252786833	0.256655711
GALK2	0.752481114	1.869135812	0.356753787	9.792940717	0.459177831
GALM	0.485093532	3.206406147	0.599237908	17.15685915	0.173340436
GALNS	0.766017127	0.96731658	0.501225662	1.866826536	0.921091099
GALNT1	0.203172291	0.395346547	0.147629657	1.05872286	0.064830495
GALNT10	0.829775761	0.836976269	0.306221041	2.28765885	0.728673647
GALNT11	0.256216781	3.419458924	0.599336667	19.50940094	0.166424588
GALNT12	0.778381623	3.419254823	0.586949917	19.91874129	0.171510757
GALNT13	0.905053734	0.052084339	0.000144116	18.82359797	0.325473122
GALNT14	0.000496403	1.543123009	1.176455246	2.024070724	0.001724692
GALNT2	0.682277775	1.060462759	0.916392049	1.227183567	0.43069864
GALNT3	0.797724577	1.140567758	0.539724312	2.410294999	0.730446434
GALNT4	0.761494648	0.364687607	9.72E-05	1368.901545	0.810167434
GALNT5	0.343330299	3.738647015	0.455254031	30.70259797	0.219635027
GALNT6	0.384793658	0.799898316	0.484779776	1.3198515	0.382213855
GALNT7	0.935369354	0.704741193	0.352277504	1.409854853	0.322623754
GALNT8	0.992647328	0.907444805	0.594600515	1.384889609	0.652501791
GALNT9	0.125316768	1.01327012	0.801593262	1.280844519	0.91220344
GALNTL5	0.135535589	0.309151326	0.055529785	1.721140133	0.180209683
GALP	0.905731245	0.976530447	0.879638868	1.084094563	0.655989046
GALR1	0.786653088	0.916252074	0.504217295	1.664992199	0.774105578
GALR2	0.5440554	0.697483641	0.242283015	2.007913884	0.504252189
GALR3	0.961985396	10.21636755	0.000143997	724836.1084	0.683425035
GALT	0.125864166	11.77088391	0.335853046	412.5426571	0.174237983
GAMT	0.442295054	0.005200029	5.89E-08	459.1100209	0.365412161
GAN	0.065307165	108.0240346	0.021318051	547385.4956	0.282012842
GANAB	0.559476386	0.562058932	0.089607762	3.525478558	0.538558632

GANC	0.323725886	0.083606843	0.001262281	5.537677479	0.246069456
GAP43	0.551984281	0.786804937	0.050096035	12.35750522	0.864506538
GAPDH	0.62754391	1.025079807	0.741531914	1.417051093	0.880817984
GAPDHS	0.960480677	0.722975127	0.140162993	3.729180032	0.698361905
GAPVD1	0.93111422	1.018581742	0.101943266	10.17731537	0.987491808
GARNL3	0.513138424	0.801341477	0.421714087	1.522709775	0.498936976
GARS	0.941104828	1.348497949	0.137076537	13.26592246	0.797699825
GART	0.795627585	1.185715259	0.175761712	7.9990156	0.861161036
GAS1	0.362745566	66.42401746	0.282949952	15593.39403	0.131899985
GAS2	0.341883414	3.081503658	0.72767829	13.04926218	0.126444697
GAS2L1	0.426900988	0.888973906	0.006037422	130.8960439	0.963146298
GAS2L2	0.263643675	0.796342301	0.368896785	1.719074516	0.561898866
GAS2L3	0.484058057	0.207994092	0.033045334	1.309157366	0.094335321
GAS5	0.753497025	0.133484588	0.001609872	11.06804296	0.371639995
GAS6	0.708280514	0.829958495	0.151603755	4.543628243	0.829870685
GAS7	0.099321413	0.000329166	2.70E-08	4.012529932	0.094816965
GAS8	0.457193314	0.011082944	1.18E-06	104.1278045	0.334728172
GAST	0.994904265	0.969683127	0.734240446	1.280623225	0.828251757
GATA1	0.340619796	1.107344354	0.650818553	1.884106582	0.706906824
GATA2	0.054768631	0.580307975	0.272885904	1.234059146	0.157465102
GATA3	0.994479222	0.474829566	0.002422021	93.08885383	0.78211822
GATA4	0.513555077	1.055972904	0.632149243	1.763948603	0.835196414
GATA5	0.193550741	0.6751271	0.154236912	2.95517199	0.602003469
GATA6	0.545051906	1.075469225	0.348721286	3.316786503	0.899243428
GATAD1	0.740306398	0.991551107	0.409819179	2.399042423	0.98498345
GATAD2A	0.186741864	2.255415315	0.916976569	5.547468079	0.076525171
GATAD2B	0.982611707	0.228075557	1.49E-05	3484.739423	0.763645782
GATM	0.424669449	0.267101295	0.037934132	1.88071003	0.184950696
GATS	0.58848904	1.948743595	0.572991496	6.627675326	0.285390699
GBA	0.128781427	0.332957624	0.077173533	1.43651295	0.140385146
GBA2	0.068835383	0.300191754	0.042961533	2.097576213	0.225075181
GBA3	0.951109638	0.87264823	0.024415211	31.1901846	0.940488979
GBAS	0.592551508	0.479128338	0.100605433	2.281824708	0.355495885

GBE1	0.755425839	1.114671811	0.550689757	2.25624906	0.762846303
GBF1	0.222905365	1.142332795	0.848297329	1.538286365	0.38080596
GBGT1	0.562630594	0.465213387	0.042290829	5.117504171	0.531649087
GBP1	0.114578364	36.37451679	5.175262273	255.6595979	0.000303526
GBP2	0.884826541	1.028049818	0.037932723	27.86212913	0.986889604
GBP3	0.415241397	2.391879986	0.075790105	75.48597312	0.620483468
GBP4	0.11732224	0.337482954	0.047762424	2.384609789	0.276222341
GBP5	0.769271263	0.727057392	0.314143298	1.682711215	0.456583786
GBP6	0.052337402	0.429647974	0.150177274	1.229196515	0.115213578
GBP7	0.239984914	87.15379064	0.00129349	5872316.376	0.430937212
GBX2	0.5107623	1.134251926	0.614900925	2.092251579	0.68675669
GC	0.316224249	0.033297692	9.21E-06	120.3909649	0.415700217
GCA	0.51920463	1.374117189	0.120013618	15.73319832	0.798336981
GCAT	0.152775804	0.048358582	0.000763368	3.063468594	0.152415154
GCC1	0.662052875	1.007993561	0.842934943	1.205372999	0.930463347
GCC2	0.848854554	1.552559612	0.553279832	4.356640549	0.403364248
GCDH	0.093745626	1.298825301	0.697003848	2.420283855	0.41032783
GCG	0.597347925	2.167477632	0.000430319	10917.39693	0.858834624
GCGR	0.922550609	0.696985014	0.20278467	2.395585967	0.566593548
GCH1	0.935264299	0.028324187	1.25E-05	64.36398402	0.366081857
GCHFR	0.082092787	0.625065185	0.263743526	1.481387967	0.285817955
GCK	0.1889109	1.950073126	0.327674328	11.60538032	0.463006423
GCKR	0.086245835	0.526334704	0.129526923	2.138769411	0.369603801
GCLC	0.345164083	1.208745976	0.01502317	97.25423199	0.932511727
GCLM	0.942160072	0.957931265	0.586076391	1.565721334	0.863870001
GCM1	0.713504681	0.956441802	0.369191973	2.477792011	0.926937954
GCM2	0.319594511	1.042733274	0.666510381	1.63132145	0.854596803
GCN1L1	0.455270051	0.795347926	0.199741323	3.166987753	0.745338225
GCNT1	0.425409707	0.955563113	0.875342483	1.043135549	0.309626388
GCNT2	0.192758718	0.172153719	0.00220041	13.46881094	0.428978894
GCNT3	0.979331644	1.031773712	0.385775515	2.759524523	0.95031016
GCNT4	0.392733282	0.33334639	0.084994066	1.307383224	0.115126849
GCSH	0.658265837	0.802739214	0.498809142	1.291857331	0.36541074

GDA	0.08457828	0.699594031	0.356801802	1.37171899	0.298371285
GDAP1	0.298091303	0.112197943	0.005798146	2.171104083	0.147865007
GDAP1L1	0.343043124	1.010057788	0.779941485	1.308068303	0.939526526
GDAP2	0.752377468	1.056331158	0.319215896	3.495551222	0.92848153
GDF10	0.69211861	1.008340656	0.851708593	1.193777879	0.923176369
GDF11	0.896378172	0.108745129	2.32E-05	510.7950167	0.607008684
GDF15	0.988046461	0.990528834	0.819256035	1.197607744	0.921735446
GDF2	0.084276206	1.245075354	0.356311966	4.350717302	0.731313619
GDF3	0.350928508	2.491481606	0.64747267	9.587247278	0.184263329
GDF5	0.992273047	20.674489	0.06724175	6356.683106	0.300043078
GDF6	0.475059549	1.255055964	0.052853687	29.80237612	0.888204061
GDF7	0.716280403	0.601398959	0.244398131	1.479883281	0.268377425
GDF9	0.780853182	0.957114976	0.335492282	2.730522061	0.934687644
GDI1	0.30724602	0.823263801	0.036653658	18.49101342	0.902508131
GDI2	0.553108636	0.982075696	0.301063619	3.203551048	0.97608105
GDNF	0.375905591	2.189016417	0.639685896	7.490852775	0.21196829
GDPD1	0.351560367	1.234636167	0.624915118	2.439253619	0.544047677
GDPD2	0.103020073	130.0993391	0.090164518	187721.7164	0.189628997
GDPD3	0.975644009	1.009815084	0.829008826	1.230055064	0.922701752
GDPD4	0.917621013	14.44140492	0.000955501	218266.8176	0.586571395
GDPD5	0.799397676	5.380954918	0.133780533	216.4341483	0.371967497
GEM	0.925950273	0.809031129	0.468366574	1.397476686	0.447313681
GEMIN4	0.539745685	11.85366003	0.243944942	575.9875775	0.212056144
GEMIN5	0.596930783	0.990208605	0.512527599	1.913093234	0.976638028
GEMIN6	0.159003786	0.083363425	0.001640734	4.235580695	0.215086713
GEMIN7	0.341440793	0.00168749	1.16E-06	2.460316306	0.085843594
GFAP	0.260458508	0.306523132	0.001021895	91.94333597	0.684496896
GFER	0.520916396	2.069175308	0.615874599	6.951880243	0.239582246
GFI1	0.935995896	42.31611823	0.001125931	1590376.227	0.485922908
GFI1B	0.688958763	0.990426444	0.432406861	2.2685684	0.981849947
GFM1	0.084880191	0.473753948	0.177612256	1.263667314	0.13558012
GFM2	0.160078559	0.581308434	0.228548645	1.478545173	0.254731889
GFOD1	0.773455252	0.455966339	0.005875612	35.3844486	0.723552209

GFPT1	0.425007109	1.732950151	0.535350132	5.609630129	0.358930835
GFPT2	0.524762129	0.905872751	0.382140532	2.147391787	0.822380199
GFRA1	0.952922891	1.180155256	0.594391191	2.343181479	0.635956568
GFRA2	0.87644129	0.005127803	1.88E-06	13.98248313	0.191405181
GFRA3	0.737400969	1.07424735	0.465224108	2.480540776	0.866788875
GFRA4	0.397481023	0.024140315	8.78E-06	66.39910519	0.356737746
GGA1	0.433194712	32.76493755	6.12E-05	17530128.38	0.604111298
GGA2	0.753599847	1.066170735	0.862464377	1.317990709	0.553670463
GGA3	0.836851222	1.573210312	0.027193772	91.01314422	0.826761653
GGCX	0.719832073	0.973139014	0.025831659	36.66042289	0.988266827
GGH	0.441428895	0.597641302	0.140721002	2.538179234	0.485408872
GGN	0.593130905	0.997485875	0.786571312	1.264955962	0.983429833
GGPS1	0.989680676	0.771236639	0.261888796	2.271215733	0.637373089
GGT1	0.470401307	0.663219773	0.001998684	220.0750385	0.889720363
GGT2	0.812885162	0.375494816	0.001570083	89.80184617	0.725953035
GGT6	0.215616304	0.40896202	0.027985467	5.976313901	0.513476608
GH1	0.393483622	0.152325622	0.009842664	2.357399883	0.178179799
GH2	0.109797635	0.753393987	0.453102536	1.252702101	0.275051032
GHITM	0.951333357	0.940863874	0.722843512	1.224642422	0.650385491
GHR	0.673671305	1.116667755	0.014413281	86.51374185	0.960345445
GHRH	0.997331056	0.692094902	0.255148281	1.877321498	0.469763688
GHRHR	0.852095147	1.412317786	0.534408898	3.732425744	0.486266707
GHRL	0.104762962	0.05562019	0.001273488	2.429237381	0.13378142
GHSR	0.369175479	15.45089296	0.095211662	2507.361891	0.291740316
GIF	0.929960826	0.018229398	0.000223052	1.489837591	0.074665164
GIMAP1	0.318879836	0.000969133	1.18E-07	7.939147045	0.131215624
GIMAP2	0.531878371	0.876126677	0.474998763	1.615999903	0.672015608
GIMAP4	0.596099974	1.34831728	0.485281447	3.746196149	0.56650533
GIMAP5	0.111011268	1834.038551	0.000447482	7516944471	0.333411976
GIMAP6	0.3477873	0.90662848	0.0914653	8.986743578	0.933249266
GIMAP7	0.456757294	0.783303376	0.177470505	3.457274081	0.747139541
GIMAP8	0.829059018	0.998709547	0.509064633	1.959320477	0.997003451
GIP	0.392199549	1.471236143	0.729266336	2.968100515	0.280915012

GIPC1	0.274558997	2.49776105	0.800262834	7.795951531	0.114960605
GIPC2	0.722537297	3.124742952	0.124324598	78.53649782	0.488560592
GIPC3	0.172136763	0.409551155	0.095714516	1.752421227	0.228748603
GIPR	0.544991354	0.79194876	0.291237228	2.153511907	0.647660574
GIT1	0.794099323	1.78728231	0.548163086	5.827422774	0.335549851
GIT2	0.732584945	0.702856755	0.297026388	1.663177546	0.422352057
GJA1	0.954657492	9.252012753	0.313352912	273.1735903	0.197706332
GJA10	0.32624514	0.051016838	7.57E-06	343.9705537	0.508278551
GJA3	0.949232914	0.758312271	0.130955941	4.391076077	0.757508974
GJA4	0.14475447	2.455208188	0.484405757	12.44421059	0.278069141
GJA5	0.480510171	0.24440259	0.02674778	2.233180676	0.211958781
GJA8	0.846589828	1.059112465	0.676271992	1.65868057	0.801871308
GJB1	0.106617447	0.817104038	0.541129226	1.233825447	0.336729374
GJB2	0.943592099	1.259571065	0.463417052	3.423523714	0.651017614
GJB3	0.751905007	2.101108856	0.739502894	5.96976491	0.163453179
GJB4	0.49107462	20.82630348	0.682454887	635.5510454	0.081701135
GJB5	0.717730268	0.789119915	0.352091316	1.768604374	0.565164748
GJB6	0.263558271	0.843033898	0.679352211	1.046152706	0.121068605
GJB7	0.670704401	1.155805516	0.547797449	2.438650259	0.70387385
GJC1	0.488065199	1.043559763	0.374996705	2.904070791	0.934923801
GJE1	0.548844266	0.005106456	1.13E-06	23.1330439	0.219211312
GK	0.961917514	1.595315088	0.006595256	385.8880353	0.867532428
GK2	0.79318432	12.95974593	2.34E-05	7165718.741	0.704147462
GKAP1	0.976733518	0.507597341	0.123106891	2.092937767	0.348178253
GKN1	0.591513888	0.950815465	0.201098503	4.495558314	0.949264575
GLA	0.297040202	1.218586822	0.952091829	1.559675021	0.116400547
GLB1	0.918700583	0.954678503	0.178792402	5.097593812	0.956722887
GLB1L	0.29268077	8.268618038	0.006199753	11027.8665	0.565025824
GLCCI1	0.420183302	1.186734721	0.725843697	1.940279022	0.494894625
GLCE	0.245323466	3.379602163	0.051148824	223.3034892	0.568998067
GLDC	0.220489781	1.920154638	0.189816429	19.42399746	0.580561584
GLDN	0.673412404	1.317279958	0.441204754	3.932927901	0.621460786
GLG1	0.254843852	0.015889842	3.73E-06	67.75467487	0.33138561

GLI1	0.926147724	1.056996196	0.553712805	2.017726426	0.866553813
GLI2	0.271259021	0.149739549	0.007892423	2.840944047	0.206016892
GLI3	0.342599445	0.96381349	0.825072289	1.125884915	0.642088721
GLI4	0.370059205	0.545012284	0.156556396	1.897325161	0.340253229
GLIPR1	0.595420927	1.024031427	0.247417231	4.23834815	0.97386007
GLIS1	0.255426316	1.089799288	0.818740906	1.45059625	0.555623563
GLIS2	0.478356535	47.32969367	0.004419209	506900.6502	0.41522523
GLIS3	0.419025157	0.049388761	8.56E-05	28.49893847	0.353774452
GLMN	0.189964514	0.351204416	0.000689913	178.7826966	0.742110942
GLO1	0.327523988	1.691225763	0.728511887	3.926146752	0.221395416
GLP1R	0.832634793	0.5152591	0.212356495	1.250218126	0.142600328
GLP2R	0.295442117	0.155960947	0.022531549	1.079544824	0.059778392
GLRA1	0.925639323	1.035786738	0.772136895	1.389461082	0.814519276
GLRA2	0.582884661	9.753262368	0.011093718	8574.774204	0.510211167
GLRA3	0.417173258	15.50808979	0.005477447	43907.47117	0.499056676
GLRB	0.172798379	1.391050557	0.692038482	2.796118575	0.354151231
GLRX	0.853495635	1.514467784	0.013294883	172.5184571	0.86360107
GLRX2	0.37500433	1.086392949	0.260940885	4.523053725	0.909344786
GLRX5	0.773581946	0.050470675	3.86E-05	65.97504017	0.414672173
GLS	0.998023893	0.787904349	0.521923213	1.189434093	0.256622997
GLS2	0.84487916	1.028134313	0.794231394	1.330922165	0.833140737
GLT1D1	0.090360905	1.575131735	0.900490011	2.755210998	0.111259002
GLT6D1	0.12591799	9.448827579	0.006256623	14269.73308	0.547608748
GLT8D1	0.643961863	0.992136264	0.770468593	1.277578833	0.95120487
GLT8D2	0.148118662	1.022575673	0.52254205	2.001104039	0.948036314
GLTP	0.762744443	1.374297574	0.628331555	3.00588727	0.425896276
GLTSCR1	0.194129157	1.599153785	0.880682945	2.903761045	0.122951164
GLTSCR2	0.113343562	0.622055361	0.129016707	2.999246225	0.554200292
GLUD1	0.873610312	0.855964162	0.365426422	2.004985416	0.720246324
GLUD2	0.689118433	0.041648587	1.40E-07	12402.75184	0.621123196
GLUL	0.811536082	1.071863197	0.480667253	2.390199676	0.865321884
GLYAT	0.961126661	1.693302669	0.261283592	10.97380018	0.580698536
GLYATL1	0.693102751	0.054653346	5.01E-05	59.64232988	0.415391499

GLYATL2	0.277669659	1.962282817	0.244932129	15.72090141	0.525469817
GLYCTK	0.9287832	1.061938545	0.804939613	1.400991397	0.67076993
GM2A	0.624969543	23.38740949	0.001857632	294445.239	0.512838271
GMCL1	0.916829681	0.043918941	4.73E-05	40.75718734	0.369996071
GMDS	0.463961768	0.56567135	0.230894986	1.385842461	0.212684417
GMEB1	0.611901462	1.067800516	0.72901502	1.564025308	0.736204944
GMEB2	0.129020477	0.003630437	1.19E-06	11.10099008	0.170025929
GMFB	0.216551019	2.939633044	0.781146796	11.06250768	0.11078269
GMFG	0.123159437	0.060293969	0.001949872	1.864410689	0.108680057
GMIP	0.371877349	0.532961324	0.208268824	1.363851621	0.189291302
GML	0.154937898	0.339344528	0.099553194	1.156715357	0.084116552
GMNN	0.712552874	0.675945509	0.190410877	2.399560034	0.544594785
GMPPA	0.407064832	0.014169873	1.04E-05	19.3129171	0.247707904
GMPPB	0.895427591	0.948967498	0.586600128	1.535184309	0.830995052
GMPR	0.911447145	7.150360471	0.148285891	344.7910968	0.31984029
GMPR2	0.850164435	2.132304666	0.434996367	10.45232451	0.350503375
GMPS	0.774509095	1.056071421	0.939520943	1.187080346	0.360521978
GNA11	0.410725877	1.672945812	0.545014714	5.135178222	0.368502625
GNA12	0.451697002	0.664314298	0.081589672	5.408938103	0.702266501
GNA13	0.174059293	0.783124676	0.418834384	1.464264352	0.44390064
GNA14	0.555498558	1.046535706	0.863000425	1.269103643	0.643842058
GNA15	0.746742362	1.578335596	0.233040182	10.68975846	0.640073919
GNAI1	0.58940478	1.078454712	0.262385904	4.432648802	0.916588944
GNAI2	0.728371462	1.06340429	0.301766482	3.747363446	0.923791662
GNAI3	0.343085504	1.270157982	0.461778719	3.493667495	0.643194403
GNAL	0.941590613	0.536419094	0.194086774	1.482560808	0.229829829
GNAO1	0.934815344	1.324270592	1.03E-05	170421.8793	0.9626815
GNAQ	0.552152956	1.071419563	0.533880244	2.150182355	0.846094008
GNAS	0.178494241	0.472172652	0.193050933	1.154861099	0.100083978
GNAT1	0.254756351	1.17531792	0.205178421	6.732541398	0.856057263
GNAT2	0.329634433	1.825510248	0.715429541	4.658023569	0.20792323
GNAT3	0.765261138	0.869578965	0.082253319	9.193155829	0.907536409
GNAZ	0.465290523	2.214034977	0.000492155	9960.175311	0.853072542

GNB1	0.791105039	0.817993248	0.243939603	2.742945156	0.744849099
GNB1L	0.562017512	0.023203438	5.57E-06	96.61451514	0.37612536
GNB2	0.697933775	1.342547621	0.710456419	2.537008699	0.364311473
GNB2L1	0.915425082	737.1928332	0.087150661	6235790.641	0.152402889
GNB3	0.401292379	0.022159218	2.01E-05	24.44139925	0.286531762
GNB4	0.534322698	0.935438747	0.707755327	1.23636745	0.639082293
GNB5	0.733390352	0.798122555	0.10876589	5.856611987	0.824509928
GNE	0.755641335	0.832542983	0.313862869	2.208377882	0.712713582
GNG10	0.460820141	0.922455759	0.387815506	2.194148029	0.85513406
GNG11	0.627788547	0.357233072	0.027076497	4.713145424	0.43417378
GNG12	0.017726109	0.677162098	0.480124461	0.955061747	0.026280821
GNG13	0.372638172	0.829734206	0.365664377	1.882761616	0.655262912
GNG2	0.743197137	0.349699233	0.077602945	1.575836504	0.171349875
GNG3	0.262131423	0.312438169	0.005482541	17.80517676	0.572760708
GNG4	0.341340674	7.198761243	0.174395012	297.1539321	0.298385423
GNG5	0.639210473	0.901099402	0.276552541	2.936079087	0.862811193
GNG7	0.50344388	1.256995733	0.543677162	2.906206814	0.592736155
GNG8	0.555073913	0.749864301	0.107737091	5.219154019	0.771207646
GNGT1	0.207630255	0.405063435	0.122957815	1.334412022	0.137361346
GNGT2	0.850370789	0.257235976	0.025347687	2.610508252	0.250809017
GNL1	0.308319093	0.375036446	0.006632111	21.20777905	0.633811
GNL2	0.095171073	0.664011147	0.293900083	1.500206459	0.324813045
GNL3	0.507814829	1.186361286	0.490051803	2.872049633	0.704811514
GNL3L	0.148141447	0.602514858	0.127062022	2.857062616	0.523475713
GNLY	0.195574028	0.656501548	0.339497791	1.269505411	0.211028202
GNMT	0.284333243	0.125340878	0.007251818	2.166399618	0.153211058
GNPAT	0.488380857	1.63591935	0.99126218	2.699822683	0.054150688
GNPDA1	0.475234416	0.033980178	0.000408572	2.826070614	0.133775192
GNPDA2	0.9152182	0.036979137	0.00015138	9.033288055	0.239829981
GNPNAT1	0.145151288	8.458732213	0.373324105	191.6569267	0.179887888
GNPTAB	0.726256139	0.469988857	4.03E-07	548600.3583	0.915637707
GNPTG	0.313149097	0.158520084	3.00E-06	8363.921562	0.739890241
GNRH1	0.615475261	1.313760697	0.100948226	17.0975482	0.834886539

GNRH2	0.511400693	1.37181514	0.78755721	2.389511206	0.264203085
GNRHR	0.515615747	0.855493692	0.583649845	1.253952972	0.423707106
GNRHR2	0.778277138	1.104245342	0.689093637	1.769509557	0.680215607
GNS	0.826494271	10.88422039	0.884663602	133.9110745	0.062284392
GOLGA1	0.362763176	2.203164794	0.449964131	10.78738232	0.329749133
GOLGA2	0.916877836	0.879821636	0.42947368	1.802406405	0.726400919
GOLGA3	0.463526232	0.505879424	0.140848001	1.816951533	0.296212337
GOLGA4	0.565063852	0.974455112	0.748797681	1.268116595	0.847316998
GOLGA5	0.486646821	1.03012467	0.395033164	2.686247468	0.951604317
GOLGA7	0.085506371	1297.581595	0.136749139	12312457.78	0.124993064
GOLGA8A	0.058472876	0.539622751	0.134275268	2.168625073	0.384724386
GOLGA8B	0.986637506	0.774966939	0.359885586	1.668790806	0.514773873
GOLGA8F	0.941195086	0.989475292	0.302219223	3.23957339	0.986049853
GOLGA8G	0.136241564	1.114223567	0.83137013	1.493310997	0.469127149
GOLGB1	0.469640095	0.359941625	0.100143394	1.293724607	0.117481714
GOLPH3	0.255651762	0.710702406	0.350624028	1.440568442	0.343467208
GOLPH3L	0.876427435	1.121360023	0.33828699	3.717105108	0.851400676
GOLT1A	0.136225116	0.480085496	0.022246293	10.36047139	0.639643305
GOLT1B	0.698040053	0.387540172	7.50E-05	2001.649368	0.827967422
GOPC	0.397747451	0.928706834	0.36681684	2.35129986	0.875990578
GORASP1	0.815861606	1.047473527	0.450440444	2.435839862	0.914218514
GORASP2	0.50395577	1.820557129	0.410785841	8.068506582	0.430263448
GOSR1	0.797739872	1.030085567	0.258108116	4.110976021	0.966517001
GOSR2	0.654808814	0.922889999	0.302205232	2.818369309	0.887965586
GOT1	0.33223289	0.843834827	0.20774335	3.427581274	0.812320783
GOT1L1	0.271820099	4.857387382	0.851565931	27.70685313	0.075224047
GOT2	0.387315509	22.63388483	0.000975969	524906.9252	0.54301086
GP1BA	0.178964293	1.327005964	0.967356518	1.820367977	0.07939779
GP5	0.152243755	0.021370044	8.65E-05	5.281371383	0.171315014
GP6	0.859536948	5.391808187	0.012906901	2252.40712	0.584238757
GP9	0.376863598	2.199373003	0.132640476	36.46881966	0.582262605
GPA33	0.422232163	0.768771506	0.273138019	2.163776507	0.618445633
GPAA1	0.632092118	1.139117833	0.387748515	3.346471713	0.812736137

GPAM	0.503388278	0.248047798	0.000712812	86.31691539	0.640561785
GPBAR1	0.710517568	1.549843776	0.483334341	4.969677362	0.461115383
GPBP1	0.066609967	2.123509955	0.67011278	6.729157638	0.200647342
GPBP1L1	0.669812398	0.660025395	0.257328423	1.69290868	0.387298346
GPC1	0.232978216	0.989291099	0.358597401	2.72923584	0.983409398
GPC2	0.137863798	1.47230552	0.712411527	3.042740694	0.296292948
GPC3	0.301638255	1.298146447	0.508852091	3.311736799	0.585007449
GPC4	0.858786592	0.746269797	0.268416994	2.074826191	0.574816939
GPC5	0.418088159	0.494239485	0.000281167	868.7801431	0.853337397
GPC6	0.133660554	76.84390914	0.000456189	12944155.55	0.479493193
GPD1	0.290233169	1.141732451	0.456229429	2.857231268	0.77701866
GPD1L	0.585786725	0.154277336	6.10E-07	39033.37248	0.768421729
GPD2	0.709899444	1.194030374	0.814200763	1.75105281	0.364000222
GPHA2	0.565071037	10.2023291	0.051449854	2023.086781	0.389471598
GPHB5	0.286515296	1.336312215	0.501929213	3.557733421	0.561722674
GPHN	0.21148805	0.962398163	0.507939609	1.823465245	0.906427432
GPI	0.901677189	0.157968153	0.01108727	2.250683726	0.173369115
GPKOW	0.92553849	0.643141576	0.036955556	11.19266309	0.762011335
GPLD1	0.556215962	0.796048082	0.568761094	1.114162969	0.183602059
GPM6A	0.143901927	71.98065474	0.013472503	384576.9893	0.32882856
GPM6B	0.340688045	0.380646412	1.42E-05	10231.04562	0.852746775
GPNMB	0.644065578	0.144005299	0.005558082	3.731057768	0.243196757
GPR1	0.082320521	0.718898923	0.37103241	1.392912446	0.32809235
GPR101	0.428918349	0.139950946	0.010061262	1.94670078	0.143186494
GPR107	0.274635786	0.922522904	0.549340372	1.549218938	0.760443487
GPR108	0.556198579	1.160024734	0.230046625	5.84949848	0.857290849
GPR110	0.613119108	1.0493352	0.011681061	94.26406829	0.983258278
GPR111	0.732380974	0.85607976	0.474289818	1.545199848	0.60604249
GPR112	0.812054019	0.455753952	0.031064906	6.686376811	0.566357847
GPR113	0.873060484	1.148296535	0.09882332	13.34285202	0.912013015
GPR114	0.928565985	0.977406828	0.440132611	2.170536978	0.95523058
GPR115	0.473506733	11.53448688	0.002577685	51613.90616	0.568577312
GPR116	0.886768467	1.364047473	0.011233809	165.6273069	0.899109562

GPR119	0.953651958	2.290507119	0.373375117	14.05134573	0.37052634
GPR12	0.391492781	0.914854905	0.779403423	1.073846319	0.276376742
GPR123	0.61355673	2.27619059	0.324477361	15.96735005	0.407932967
GPR124	0.087708695	0.446556689	0.000694098	287.2978141	0.806964422
GPR125	0.648866839	1.051647175	0.91039737	1.214812144	0.49377953
GPR126	0.060686155	0.625725903	0.147485209	2.654726595	0.524876661
GPR128	0.315562932	1.942557936	0.515070904	7.326236653	0.326892828
GPR132	0.125020081	0.776506964	0.4680038	1.288372156	0.327505861
GPR133	0.903724242	1.020356924	0.259777247	4.007773068	0.976967343
GPR135	0.844647658	0.003018671	2.02E-07	45.11001301	0.236705919
GPR137	0.854453615	0.649964466	0.107260321	3.938584231	0.639288603
GPR137B	0.429430823	0.839723463	0.253778926	2.77854235	0.774787552
GPR139	0.637172072	0.826816549	0.301058376	2.270741028	0.712173248
GPR141	0.343470377	1.015611832	7.01E-05	14713.01562	0.997471503
GPR142	0.562630283	1.796014001	0.50621115	6.372175506	0.364783997
GPR143	0.485751584	0.004974117	3.01E-07	82.16460361	0.284499044
GPR144	0.304464696	3.067740224	0.846828775	11.11326204	0.087857079
GPR146	0.212256143	5.087728264	0.153736765	168.3720794	0.362200852
GPR148	0.656184365	0.576672535	0.197270998	1.685758248	0.314509746
GPR149	0.591505452	0.889412269	0.077886043	10.15655895	0.924855619
GPR15	0.558315916	1.009329887	0.422281357	2.412483538	0.983334736
GPR150	0.845731889	0.373644509	0.035438799	3.939473805	0.412705306
GPR151	0.224654878	1.432781847	0.540211269	3.800112916	0.469922651
GPR152	0.122223533	1.884177174	0.754449385	4.705582232	0.174915097
GPR153	0.181747328	1.413851076	0.874507752	2.285828639	0.157687512
GPR155	0.511956867	1.047648628	0.76824796	1.428663278	0.768667382
GPR156	0.847788461	0.645510803	0.180538521	2.308007149	0.500730327
GPR157	0.655026868	0.938186142	0.296393956	2.969673369	0.913572133
GPR158	0.830030245	21.96327943	0.161005571	2996.080452	0.21803042
GPR160	0.924767756	0.749841426	0.097923324	5.741861475	0.781637371
GPR161	0.331776495	1.891316099	0.450934134	7.932592179	0.383650091
GPR162	0.391681917	0.904497914	0.734674162	1.11357731	0.344127797
GPR17	0.663759553	212.7426685	0.002509671	18034014.3	0.354555346

GPR171	0.991704546	0.815353361	0.312120974	2.129946906	0.676923355
GPR173	0.082005977	3.288123194	0.767600242	14.08513643	0.108793758
GPR174	0.723546667	0.522422896	7.75E-06	35220.69855	0.908878956
GPR176	0.882923466	0.069430095	0.000289568	16.64736293	0.340041598
GPR179	0.099382419	2.221929087	0.456310197	10.8193262	0.322897455
GPR18	0.313914169	0.005706745	9.37E-06	3.476465413	0.114313125
GPR19	0.451505324	0.891601938	0.564942942	1.407140361	0.622130276
GPR20	0.343124599	1.002039347	0.481329455	2.086061515	0.995655017
GPR21	0.349624384	0.180074396	0.007308032	4.437143823	0.294361751
GPR22	0.26162144	3.046635775	2.34E-05	397260.3921	0.852930242
GPR25	0.295997253	1.066329976	0.941298508	1.207969213	0.312843246
GPR26	0.19288107	0.006637613	1.38E-05	3.188954309	0.111417128
GPR27	0.195687908	0.076223656	0.00443749	1.30930895	0.076028561
GPR3	0.717671912	0.090766151	4.59E-05	179.5322019	0.535502258
GPR31	0.778579913	0.915940717	0.509514119	1.646563589	0.769197398
GPR32	0.355174156	12.66397613	0.000514781	311543.0125	0.622614495
GPR34	0.297312597	2.773679194	0.754915066	10.19094282	0.124411992
GPR35	0.663823759	0.30780236	0.037652507	2.516227998	0.271693891
GPR37	0.475743739	8.23686723	0.587575982	115.4675886	0.117526024
GPR37L1	0.588198776	1.213000872	0.444280174	3.311809087	0.706317084
GPR39	0.824407616	5.317078562	0.001908844	14810.7079	0.679702691
GPR4	0.467861892	0.93780859	0.31696988	2.774664114	0.907639099
GPR42	0.528991552	0.588827065	0.182574803	1.899042515	0.375359963
GPR45	0.732271359	0.026200289	3.06E-07	2242.17671	0.529666822
GPR50	0.426137638	1.749372754	0.230659103	13.26765342	0.588500993
GPR52	0.660305408	2.058210827	0.346211985	12.2359479	0.427379311
GPR55	0.749222555	1.042835167	0.847301374	1.283492769	0.69217324
GPR56	0.331037504	1.110990031	0.496867825	2.484159343	0.797672154
GPR6	0.645907166	8.550754899	0.041598965	1757.625687	0.42965735
GPR61	0.39276935	0.929452961	0.503864354	1.714514631	0.81484106
GPR62	0.763234897	0.574710065	0.084890247	3.890808057	0.570283524
GPR63	0.112543615	0.361663914	0.124074239	1.054213898	0.062428283
GPR64	0.055495503	2.402145018	0.667700625	8.642047756	0.179721192

GPR65	0.804486931	0.72457449	0.313506593	1.674632059	0.451014959
GPR68	0.922049096	1.189576258	0.895198026	1.580758259	0.231405118
GPR75	0.840220727	1.559567417	0.365221751	6.659654093	0.548492834
GPR78	0.42557885	0.81203282	0.594832473	1.108542878	0.189825361
GPR82	0.223415004	1.611283544	0.67140075	3.866892699	0.285512772
GPR83	0.596122464	1.201058841	0.943820985	1.528406725	0.13627977
GPR84	0.553962185	0.159298459	0.000154159	164.6089794	0.60393542
GPR85	0.067519044	0.476381161	0.180165753	1.259612372	0.134985325
GPR87	0.220925874	2.699781668	0.153875703	47.36823897	0.496829842
GPR88	0.157598633	22.04071189	0.000614926	790002.9335	0.563230195
GPR89A	0.677155918	0.85489069	0.610817631	1.196491481	0.360681976
GPR97	0.324671656	0.530268325	0.078472788	3.583210213	0.515206784
GPRASP1	0.452609389	10.92388637	0.295658062	403.6125126	0.194187974
GPRASP2	0.861165495	1.111035364	0.201971763	6.111743362	0.903656699
GPRC5A	0.271479321	10.10665018	0.410245541	248.9835176	0.157082859
GPRC5B	0.783342907	0.022839083	0.000129691	4.022052769	0.152017782
GPRC5C	0.535416964	2648.741108	0.022204474	315964678.5	0.186314451
GPRC5D	0.278673315	0.664204526	0.245363492	1.798016683	0.420651523
GPRC6A	0.419595393	0.31507244	0.023234819	4.272494741	0.38525598
GPS1	0.746233104	4.576190031	0.035683832	586.8628529	0.539142556
GPS2	0.481184233	0.136524489	4.46E-06	4176.299603	0.705528887
GPSM1	0.317822945	0.335026961	0.068759232	1.632407187	0.175915145
GPSM2	0.35690095	158.8472115	0.166526824	151521.7554	0.147661041
GPSM3	0.439431835	0.494048511	0.024733837	9.868421637	0.644423418
GPT	0.100055063	0.93112576	0.245551438	3.530808808	0.916428422
GPT2	0.611334276	0.384639143	0.015631447	9.464720164	0.558782915
GPX1	0.439480588	2.488813268	0.770299429	8.041277517	0.127554045
GPX2	0.114183299	1.443240394	0.748168352	2.784056328	0.273744726
GPX3	0.159064628	0.091911999	0.003198123	2.641492164	0.163600196
GPX4	0.635812828	0.002248387	4.56E-08	110.8689415	0.26874063
GPX5	0.473447706	14.35379986	0.32709506	629.8828552	0.167352563
GPX6	0.145715495	0.758595392	0.429687169	1.339269611	0.340753906
GPX7	0.240252181	0.457698811	0.088902577	2.356379403	0.349901121

GRAMD1A	0.820542124	0.031183725	0.000104469	9.308287067	0.232989443
GRAMD1C	0.105517287	1.08773403	0.273460926	4.32663393	0.904974722
GRAMD2	0.815405239	0.625661728	0.19111234	2.048285305	0.438336616
GRAMD3	0.670605837	1.034350649	0.341714007	3.130925988	0.952341016
GRAP	0.729299589	1.060752719	0.835825173	1.346210149	0.627635595
GRAP2	0.230263888	1.817087733	0.829934778	3.978394348	0.135243594
GRASP	0.419232942	1.031299954	0.69323789	1.534220231	0.879124281
GRB10	0.571270204	0.913853439	0.569248683	1.467070777	0.70914389
GRB14	0.363320363	1.508191684	0.01072018	212.1832027	0.870663971
GRB2	0.742976859	0.581487316	0.181778867	1.860103454	0.360794355
GRB7	0.851300884	0.80181869	0.327443048	1.96343522	0.628823701
GREB1	0.907536644	0.479636987	0.04146858	5.547613187	0.556379586
GREM1	0.718657901	1.401826744	0.000109656	17920.76017	0.944184109
GREM2	0.438611947	0.988839533	0.791661847	1.235127886	0.921210686
GRHL1	0.862808154	1.308031322	0.433388427	3.947834861	0.633761479
GRHL2	0.761981215	0.603287036	0.172534109	2.109468383	0.42879514
GRHL3	0.771027035	0.732577809	0.299747847	1.790405676	0.494914547
GRHPR	0.172798379	1.69656678	0.777870065	3.70028231	0.183978068
GRIA1	0.883109363	1.701470992	0.368580997	7.854456847	0.49584747
GRIA2	0.203027472	0.173635147	0.006250256	4.823668932	0.301960734
GRIA3	0.930033443	0.844158313	0.522352216	1.364219841	0.489082349
GRIA4	0.658602838	0.944535491	0.645472362	1.38216188	0.768937545
GRID1	0.920287515	1.103163385	0.856103585	1.421521268	0.447872199
GRID2	0.33589451	0.898755976	0.144402047	5.593842487	0.908901758
GRIK1	0.68040164	0.944384759	0.397936499	2.241218325	0.89674866
GRIK2	0.855263917	10.53799028	0.000249407	445252.6743	0.664767289
GRIK3	0.369478481	4.484515546	0.881547383	22.81315793	0.070597371
GRIK4	0.292495705	0.587671947	0.154278822	2.238533532	0.435957758
GRIK5	0.391558325	1.580704598	0.548086011	4.558822846	0.39685149
GRIN1	0.364755624	43.08799467	0.000792665	2342194.181	0.498740458
GRIN2A	0.426442528	0.425002459	0.145098047	1.244862306	0.118638016
GRIN2B	0.528342679	0.000664747	2.24E-07	1.976234754	0.072970005
GRIN2C	0.180723826	15.66708494	0.01310979	18723.22554	0.446610844

GRIN2D	0.877658559	1.108040989	0.291702731	4.208924718	0.880239887
GRIN3A	0.57746998	8.91372246	0.012247498	6487.402155	0.515291913
GRIN3B	0.945926009	0.247700766	3.60E-07	170499.002	0.838759054
GRINA	0.508576386	0.493606728	0.018167996	13.41081303	0.675172797
GRIP2	0.814990423	0.705057073	0.206534732	2.406885617	0.576931519
GRIPAP1	0.901861077	1.236746709	0.678961274	2.252768282	0.48738233
GRK1	0.795948358	1.182659217	0.899290942	1.555317371	0.229973041
GRK4	0.788921297	21.15795533	0.006625377	67567.33189	0.45848207
GRK5	0.274066836	2.093180983	0.183255508	23.90873087	0.552217243
GRK6	0.628310672	1.063680657	0.652303732	1.734493436	0.804558964
GRK7	0.683939377	1.585851703	0.62106503	4.049375672	0.334998576
GRM1	0.422988332	115.8852291	0.00034134	39343125.81	0.464516785
GRM2	0.602601033	1.611748663	0.301022466	8.629700587	0.577142821
GRM3	0.062664686	0.062892773	0.001315105	3.00774534	0.160943064
GRM4	0.934352996	1.047276944	0.333416375	3.289547484	0.936951006
GRM5	0.895889594	0.896788883	0.049181253	16.35237515	0.941376682
GRM6	0.214202654	0.999571769	0.419871436	2.379642041	0.999227762
GRM7	0.614353981	0.863569477	0.245963868	3.031958504	0.818936629
GRM8	0.143612137	24.92427446	0.0052423	118501.3078	0.456618705
GRN	0.296350599	0.064863269	0.000216095	19.46937439	0.34727398
GRP	0.324861141	1.310355353	0.52994676	3.240006883	0.55840812
GRPEL1	0.072788714	0.543408213	0.21513263	1.372606684	0.197032172
GRPEL2	0.560188174	1.365187299	0.203487484	9.15897295	0.748562612
GRPR	0.360768744	0.996497221	0.594362531	1.670708802	0.989381492
GRSF1	0.052049863	0.812191771	0.642811597	1.026203441	0.081297678
GRTP1	0.662359204	0.977722798	0.28824843	3.316381883	0.971161146
GRWD1	0.183088124	1.075450995	0.004234706	273.1227943	0.979458822
GSC	0.832811285	1.111416069	0.59637084	2.071271087	0.739450771
GSG1	0.987631128	0.679182552	0.103228533	4.468618571	0.687333899
GSG2	0.307293197	2.258860096	0.795375948	6.415141104	0.125997334
GSK3A	0.465783793	0.540912719	0.21240228	1.377511431	0.197596565
GSK3B	0.229464939	0.514524129	0.194478098	1.361259091	0.180678626
GSN	0.829405303	0.848345015	0.048990534	14.69037388	0.909999005

GSPT1	0.57563847	1.632485313	0.507176214	5.254600317	0.411239
GSPT2	0.620211667	0.629763417	0.215001858	1.844644346	0.399052059
GSR	0.106560531	0.462550605	0.191404377	1.117806526	0.086788636
GSS	0.307352679	0.344495574	0.052783226	2.248388533	0.265520824
GSTA1	0.270106753	1.00662833	0.091094328	11.12364087	0.995699712
GSTA2	0.330725683	2.96225897	0.711700131	12.32960038	0.135559608
GSTA3	0.798624394	0.785480563	0.200251202	3.081028774	0.729141551
GSTA4	0.061141951	6.790041335	0.482640923	95.52580211	0.155626082
GSTA5	0.137226809	0.226691512	0.044073617	1.165981948	0.07570233
GSTK1	0.301740619	1.373900034	0.525399096	3.592699951	0.517187547
GSTM1	0.877085561	0.602183401	0.221625861	1.636202774	0.319976244
GSTM2	0.152316651	0.905092863	0.637980945	1.284040058	0.576269885
GSTM3	0.735559017	1.74920488	0.543092877	5.633875609	0.348764322
GSTM4	0.102303231	0.686868225	0.435048782	1.084448406	0.106955978
GSTM5	0.101837749	1.520221303	0.830093143	2.784112637	0.174855754
GSTO1	0.552332673	126.6798409	0.106119056	151224.3195	0.180439199
GSTO2	0.551204743	1.433084509	0.50801982	4.042620242	0.496474764
GSTP1	0.779530035	0.625898621	7.98E-05	4906.103444	0.918423813
GSTT2	0.747434419	1.117647324	0.826337437	1.511653092	0.470354277
GSTZ1	0.322465606	1.266828766	0.865452046	1.854354762	0.223741159
GTDC1	0.396269779	1.129988782	0.932631126	1.369110049	0.212097693
GTF2A1	0.261224279	0.356043237	0.079199101	1.600608896	0.178108314
GTF2A2	0.502029096	2.240715566	0.827593016	6.066757636	0.11237819
GTF2B	0.171101608	0.741308007	0.420331681	1.307390294	0.30111056
GTF2E1	0.083545249	804.0582594	0.715310375	903817.0105	0.061973562
GTF2E2	0.354203557	0.77365137	0.328715221	1.820835801	0.556762332
GTF2F1	0.666257872	0.955554545	0.34837345	2.620993329	0.929629965
GTF2F2	0.861382056	1.135211588	0.691140885	1.864605867	0.616443134
GTF2H1	0.099283215	0.900572609	0.759458405	1.067907102	0.22844394
GTF2H2	0.111759212	0.173706154	0.026685265	1.130729919	0.067038858
GTF2H3	0.102717126	1.045146971	0.957110271	1.141281444	0.325334233
GTF2H4	0.417567152	0.791782143	0.323994935	1.934965316	0.608581362
GTF2H5	0.989737834	0.741345997	0.274945848	1.998916844	0.554260762

GTF2I	0.900103524	0.735762668	0.309001764	1.751921081	0.488171487
GTF2IP1	0.270847978	0.398775545	1.09E-05	14610.77364	0.863857985
GTF2IRD1	0.635441401	0.927923944	0.402825167	2.137510059	0.860526117
GTF2IRD2	0.705240936	1.126672952	0.443507306	2.862166922	0.802018838
GTF2IRD2B	0.549208917	1.144352236	0.902155895	1.451569566	0.266431074
GTF3A	0.076004682	0.020880805	0.000123442	3.532096646	0.1394283
GTF3C1	0.212246104	0.180968476	0.000988501	33.13056919	0.520165878
GTF3C2	0.473090858	1.006156676	0.032212151	31.42762054	0.997211015
GTF3C3	0.891344553	6.959061385	0.000757533	63929.29254	0.676911758
GTF3C4	0.989887622	1.520805066	0.128625608	17.98124099	0.739392438
GTF3C5	0.253217464	1.975201906	0.058842165	66.303178	0.704170563
GTPBP1	0.727252271	1.27001685	0.475491919	3.39215607	0.63345725
GTPBP2	0.554713056	1.078625175	0.417632578	2.785779485	0.875762908
GTPBP3	0.363281533	0.502937351	0.001036918	243.9401407	0.827567116
GTPBP4	0.186288649	0.797195713	0.438757275	1.448456905	0.456923786
GTPBP6	0.843622279	1.110149705	0.471412394	2.614340192	0.811016265
GTPBP8	0.655751712	0.003341798	2.12E-07	52.55587977	0.247525973
GTSCR1	0.152997566	1.353510488	0.455309853	4.023612988	0.586057633
GTSE1	0.654054562	0.734959755	0.170066369	3.176206113	0.68007098
GUCA1A	0.616259718	0.994104406	0.46135571	2.14204257	0.987955012
GUCA1B	0.448657768	0.677909463	0.297248577	1.546050258	0.355402814
GUCA1C	0.093469697	0.271922293	0.035672767	2.072778213	0.208894056
GUCA2A	0.603200185	0.894638897	0.426906169	1.874835302	0.768040028
GUCA2B	0.948768679	1.156789758	0.576182655	2.32246239	0.682116733
GUCY1A2	0.810643739	6.16E-06	4.42E-12	8.579403273	0.096473718
GUCY1A3	0.639442958	0.333603844	0.007772371	14.31886426	0.567090277
GUCY1B2	0.191374503	0.000418362	5.74E-09	30.48047222	0.173265883
GUCY1B3	0.810759833	0.884628586	0.429503985	1.822026716	0.739487309
GUCY2C	0.600918424	0.98623963	0.827820245	1.174975623	0.876748869
GUCY2D	0.05373854	0.450568521	0.123471582	1.644200139	0.22739905
GUCY2F	0.217714155	0.905175715	0.410785361	1.994577102	0.804791299
GUF1	0.673949589	4.131563946	0.270854504	63.0221036	0.307521627
GUK1	0.52345713	1.240266553	0.70925485	2.168841173	0.450155065

GULP1	0.550761035	1.09713136	0.747611576	1.610056961	0.635734063
GUSB	0.832375954	1.019338877	0.824897088	1.25961379	0.859213558
GYG2	0.848182208	0.917450399	0.643245746	1.308543802	0.634375311
GYLTL1B	0.78977583	0.807629796	0.370448951	1.760744323	0.591074583
GYPA	0.215940736	0.933677215	0.449771715	1.938212463	0.853895673
GYPB	0.371367407	0.584304423	0.211453763	1.614592499	0.300134717
GYPC	0.887654834	1.188405502	0.180899293	7.80714844	0.85737054
GYPE	0.277554643	0.256440879	0.019508204	3.370988166	0.300486626
GYS1	0.318467347	0.707392096	0.291679192	1.715595734	0.443771501
GYS2	0.517099339	0.874781089	0.301718619	2.536276866	0.805431103
GZMA	0.178061436	38.12478721	0.081497938	17834.79963	0.245768488
GZMB	0.557585147	0.126545694	0.00026127	61.29215835	0.512279636
GZMH	0.670490678	81.97765677	0.017088056	393276.8088	0.308224587
GZMK	0.629362724	0.992536702	0.780355317	1.26241096	0.95132192
GZMM	0.825606108	1.038196941	0.801479974	1.344828223	0.776480027
H19	0.679670056	0.801861921	0.387038392	1.661288784	0.552401971
H1F0	0.279362214	0.00462337	7.22E-08	296.2260581	0.341028167
H1FOO	0.912796181	1.429496827	0.020205467	101.1340709	0.86939054
H1FX	0.685710756	0.929977153	0.393483774	2.197949604	0.868610655
H2AFB1	0.308754655	2.029054453	0.751312989	5.479822699	0.162750404
H2AFB2	0.562914585	0.921614389	0.348078208	2.440178849	0.869487047
H2AFB3	0.152089726	0.000116404	7.25E-09	1.86940247	0.066751341
H2AFJ	0.924664586	0.120133378	0.001229881	11.73448782	0.364652144
H2AFV	0.618835767	0.13730543	2.13E-06	8833.967549	0.725225051
H2AFX	0.470050705	0.535370261	0.216893985	1.321481165	0.175322755
H2AFY	0.701103986	0.974380454	0.478128269	1.985695742	0.94303858
H2AFY2	0.497182338	0.183828134	0.009687885	3.488148588	0.259341196
H2AFZ	0.666988452	0.88104529	0.61195609	1.268458336	0.495814801
H2BFM	0.51447663	2.050481042	0.233200333	18.02944469	0.517374125
H2BFS	0.644095846	0.989916866	0.604311242	1.621574006	0.967896657
H2BFWT	0.820549497	1.199177396	0.365039943	3.939367337	0.76470029
H3F3A	0.694585808	1.207298724	0.21225524	6.867063491	0.831793721
H3F3B	0.26801612	1.189167875	0.571525726	2.47428973	0.643040297

H6PD	0.384077092	0.309304404	0.014111402	6.779568246	0.456309137
HAAO	0.225421167	0.352396977	0.121328144	1.023535226	0.05521208
HABP2	0.220383622	0.212106575	3.09E-06	14577.39614	0.784949473
HABP4	0.997321372	1.196074985	0.000155504	9199.738527	0.968716201
HACE1	0.198365787	0.104027863	0.00014373	75.29266582	0.500538377
HADHA	0.890769866	1.036573509	0.523027491	2.054355951	0.918024793
HAGH	0.928220416	1.006960083	0.477968641	2.121412417	0.985444402
HAGHL	0.507587319	2.16242344	0.004093442	1142.333184	0.809479845
HAL	0.533068328	1.06460907	0.444959848	2.547179203	0.888138594
HAMP	0.248634405	0.919479866	0.675232805	1.252076643	0.594102467
HAND1	0.613539347	0.523296929	0.136102804	2.012006125	0.345942855
HAND2	0.067872938	0.67288553	0.398760891	1.135454721	0.13778349
HAO1	0.068759317	1.448868711	0.953775198	2.200959456	0.082191566
HAO2	0.508704048	0.914659282	0.256738674	3.258572567	0.890546524
HAP1	0.55479809	0.964894486	0.801043987	1.162259981	0.706646818
HAPLN1	0.215021542	0.897835622	0.48590692	1.658977823	0.730826336
HAPLN2	0.114100545	9.581219934	0.822716158	111.5813449	0.071205234
HAPLN3	0.656517814	1.304665565	0.858925432	1.981722944	0.212419249
HAPLN4	0.486477139	1.070177111	0.771918764	1.483678208	0.684084518
HARS	0.880268805	0.080167044	1.28E-05	503.6479916	0.571682629
HARS2	0.305579178	0.064952056	0.002881231	1.464224535	0.085420387
HAS1	0.534043454	1.273531949	0.60564014	2.677965871	0.523731478
HAS2	0.541951758	0.784575925	0.272986825	2.254905094	0.652412184
HAS3	0.91151044	1.045949597	0.924270196	1.183647989	0.476492751
HAT1	0.203679386	0.004450171	3.34E-06	5.937092281	0.140261479
HAVCR1	0.462591712	1.403418151	0.344070658	5.724354761	0.636568185
HAVCR2	0.75392594	0.633825623	0.125184621	3.209139567	0.581636293
HAX1	0.852889948	1.061218252	0.30538816	3.687713954	0.925510397
HBA1	0.945828195	0.984125858	0.832544879	1.163305102	0.851269395
HBB	0.212254496	1.174565269	0.902880723	1.528002022	0.23061364
HBBP1	0.821953276	0.957620447	0.74131205	1.23704575	0.74026702
HBD	0.930625505	0.950504865	0.670842744	1.346753029	0.775246201
HBE1	0.320745954	1.360203259	0.003087894	599.1633901	0.921105701

HBEGF	0.632080688	0.896558309	0.434171443	1.851381092	0.767887742
HBG1	0.263183625	0.25099446	2.00E-07	314350.6178	0.846988394
HBG2	0.976146601	0.997573616	0.756319003	1.315784893	0.986278888
HBP1	0.231101315	2.841587922	0.939954632	8.590437926	0.064276619
HBQ1	0.73498138	1.867970147	3.88E-05	90016.72832	0.909573204
HBS1L	0.580074054	0.420642917	0.129630235	1.364962916	0.149327235
HBZ	0.891836774	0.294848908	3.18E-06	27308.44289	0.834208384
HCCS	0.306152137	0.001809427	5.38E-07	6.089506143	0.127515067
HCFC1	0.969296339	0.36160877	0.002604047	50.21450135	0.686134024
HCFC1R1	0.716811106	0.794389075	0.350833381	1.798728503	0.580931858
HCFC2	0.057772125	1.20794543	0.640812706	2.277002543	0.559158925
HCG18	0.883491843	1.102991614	0.544042707	2.236204042	0.785740933
HCG22	0.398104097	3.03472791	0.504468107	18.2560074	0.225295008
HCG27	0.784912124	14.34474444	0.48423575	424.9411431	0.123435038
HCG4	0.080501995	1.129348166	0.901156507	1.415322721	0.290858658
HCG9	0.521667728	0.914858314	0.365026562	2.292889948	0.849447574
HCK	0.754973708	3.654310116	0.012000925	1112.74605	0.656936607
HCLS1	0.175216159	0.422800941	0.167761998	1.065560956	0.067953007
HCN1	0.16101671	0.848657036	0.078094538	9.222396144	0.892758977
HCN2	0.158728331	0.635951713	0.055421198	7.29747096	0.716186929
HCN3	0.306197896	0.000638333	1.33E-09	306.1053304	0.270329692
HCN4	0.848676093	0.892599887	0.660946654	1.205444573	0.458612404
HCP5	0.596277201	14.95680358	0.047287744	4730.738954	0.35703818
HCRT	0.357958226	1.034472464	0.341514836	3.133489869	0.952205243
HCRTR1	0.948307701	1.040695076	0.76064827	1.423846321	0.803050164
HCRTR2	0.505311579	1.39359794	0.458572158	4.235135487	0.558398184
HCST	0.851691998	1.196591588	0.425951062	3.361492804	0.733433645
HDAC1	0.172175962	0.774671899	0.34197129	1.754874072	0.540562568
HDAC11	0.938273929	1.797051159	0.651145568	4.959555938	0.257777048
HDAC2	0.085786582	304.8250266	0.898377712	103428.9871	0.054364916
HDAC3	0.116208875	2.023373751	0.278286169	14.71162346	0.486257778
HDAC4	0.462450863	1.804361089	0.514727647	6.325129339	0.356405103
HDAC5	0.844116968	0.850629858	0.187899719	3.850836821	0.833685756

HDAC6	0.915136005	1.04995738	0.45190088	2.439496248	0.9097638
HDAC8	0.264500461	0.001609217	1.03E-06	2.515825229	0.08651114
HDAC9	0.343664441	0.128335424	0.001574939	10.45753448	0.360475211
HDC	0.521865231	0.016247332	5.48E-05	4.813095982	0.155953038
HDDC2	0.073683652	2.861362163	0.072671944	112.6623693	0.574816465
HDDC3	0.724079846	2.824137925	0.224674489	35.49915722	0.421472055
HDGF	0.845937669	0.745518217	8.06E-05	6895.807696	0.949744173
HDGFL1	0.766313255	4.012940458	0.000471168	34178.20788	0.763463207
HDGFRP3	0.388819788	0.703356898	0.372231601	1.329040643	0.278440111
HDHD2	0.605160048	1.075770908	0.222323381	5.205404126	0.927656442
HDHD3	0.747910559	1.048387793	0.56895695	1.931810419	0.879558288
HDLBP	0.752629052	0.026453352	0.000206762	3.384468878	0.142260205
HEATR1	0.223191255	0.128644758	0.00444846	3.720270435	0.232235082
HEBP1	0.516809512	2.131999683	0.00043234	10513.5402	0.86147485
HEBP2	0.238145753	1.976531449	0.329815066	11.84505188	0.455786855
HECA	0.584781406	0.775279758	0.249431255	2.409716865	0.660004557
HECTD1	0.999986965	0.807792522	0.000450767	1447.597338	0.95546387
HECTD2	0.799347628	0.716152828	0.33612142	1.525861916	0.387000618
HECTD3	0.125813567	1.098370442	0.9058427	1.331818016	0.339961351
HECW1	0.970840238	0.84302171	0.455944247	1.558711637	0.586067552
HECW2	0.284102083	1.353150191	0.786474974	2.328129314	0.274662441
HEG1	0.623067222	0.710534677	0.234470339	2.153191444	0.545756518
HELB	0.961062389	0.00617405	2.04E-06	18.6926077	0.213508566
HELLS	0.712063796	0.004475708	1.34E-07	149.9722953	0.308927509
HELT	0.484890861	1.014820055	0.53607776	1.921101417	0.963963417
HELZ	0.704185659	0.911590468	0.76775031	1.08237948	0.290755392
HEMGN	0.57844189	1.151852284	0.756492675	1.753835467	0.50987001
HEMK1	0.382001876	7.070480279	0.026427452	1891.657636	0.49279176
HEPH	0.387935666	0.069916664	0.001520893	3.214125481	0.173144823
HERC1	0.843883946	0.971373342	0.093633382	10.0772412	0.980585902
HERC2	0.565317271	12.770002	0.002821799	57790.41546	0.553128943
HERC2P2	0.416517315	0.907371853	0.735741759	1.119038943	0.363548187
HERC2P4	0.148366646	0.000390049	8.40E-08	1.812166401	0.068460535

HERC3	0.724577706	1.035596948	0.641608265	1.671519987	0.8861357
HERC4	0.974565653	1.196495447	0.387377685	3.695621639	0.75520718
HERC5	0.926484645	0.583472731	0.112391569	3.029056623	0.521438747
HERC6	0.246775358	1.021650878	0.482000022	2.165498901	0.9554339
HERPUD1	0.870420781	0.52748306	0.173025496	1.60807734	0.260719847
HES1	0.794597494	1.202028277	0.287197535	5.030934478	0.801099569
HES2	0.736581078	0.837284308	0.203118107	3.451415646	0.805876473
HES3	0.662476476	1.150553644	0.295079232	4.486163531	0.839917129
HES4	0.547468657	0.537185939	0.152079351	1.897487927	0.334477633
HES5	0.063244282	433.280318	0.245138584	765819.1998	0.111510733
HES6	0.145189599	0.734684468	0.257153535	2.098984437	0.564860901
HES7	0.119083076	0.838976592	0.540597138	1.302044855	0.433653041
HESX1	0.932144887	1.165139965	0.492854566	2.754465989	0.72770906
HEXA	0.472790051	26.97316041	0.000391113	1860206.657	0.562169716
HEXB	0.871435122	0.79770712	0.333772948	1.906495575	0.611156909
HEXDC	0.578542699	0.670603111	0.203081114	2.214428127	0.512083298
HEXIM1	0.947851406	0.859248767	0.566907249	1.302344335	0.474641171
HEXIM2	0.636957278	2.370968225	0.841572179	6.679748296	0.10234683
HEY1	0.608641236	0.571333497	0.1710388	1.908467345	0.362989704
HEY2	0.729336268	1.958174084	0.005564063	689.1449554	0.822265021
HEYL	0.307537213	1.028838751	0.819116293	1.292257502	0.806887703
HFE	0.669901114	1.099441595	0.812126387	1.488403578	0.539591375
HFE2	0.820722816	1.028853977	0.858265356	1.233348752	0.75843626
HFM1	0.956165798	1.036675179	0.808185753	1.329762895	0.776766317
HGD	0.291716772	3.161049468	0.934692893	10.69039234	0.064122855
HGF	0.824015275	0.041526553	5.48E-08	31458.68613	0.645088896
HGFAC	0.24348794	1.466531081	0.985591883	2.182154143	0.058973745
HGS	0.531481644	0.99530059	0.790770252	1.252732081	0.967986061
HHAT	0.999037763	1.502659292	0.762521707	2.961207437	0.239349621
HHEX	0.350484333	2.212339571	0.014212105	344.3857577	0.757838844
HHIP	0.143620543	0.834233775	0.556858882	1.249770839	0.37949031
HHLA1	0.677195501	0.028598719	3.16E-06	259.1133096	0.444528818
HHLA2	0.401167995	1.011963886	0.46678706	2.193871669	0.97596772

HHLA3	0.279012631	1.111789548	0.182832165	6.760714122	0.908398549
HIAT1	0.229407228	0.813750582	0.385319428	1.7185482	0.58895961
HIATL1	0.623219359	0.826404736	0.038205969	17.87534251	0.903242125
HIATL2	0.695264442	1.408640486	0.257607768	7.702671538	0.692647166
HIBADH	0.766595759	1.0666069	0.573770464	1.98276201	0.838477456
HIBCH	0.117255498	0.590142046	0.273824734	1.271863317	0.178256498
HIC1	0.838553665	1.135019399	0.883588159	1.45799717	0.321551619
HIC2	0.303596797	2.04273456	0.857134231	4.868274225	0.106951953
HIF1A	0.45411822	0.380326621	0.000259978	556.3876588	0.794883197
HIF1AN	0.684420235	0.304182841	0.000119807	772.3007874	0.766050404
HIF3A	0.711855127	2.191737939	0.225567199	21.29616016	0.498799665
HIGD1A	0.404676211	0.854018257	0.623178239	1.1703669	0.326350387
HIGD1B	0.294676092	1.501078917	0.891679144	2.52696043	0.126382272
HIGD2A	0.527873701	0.775364563	0.521121877	1.153646069	0.209493011
HILS1	0.691220675	260.3378192	0.0183858	3686311.263	0.254069215
HINT1	0.418098201	0.799995571	0.43068718	1.485980878	0.479995422
HINT2	0.573360494	0.365688566	0.004150638	32.21868976	0.659755584
HINT3	0.183942152	1.343469858	0.740832043	2.436329904	0.330950142
HIP1	0.167033344	0.91461753	0.127103133	6.581468183	0.929370538
HIP1R	0.741474848	1.056087763	0.414033953	2.69379203	0.909059502
HIPK1	0.803979125	50.32317537	0.02761741	91696.57873	0.306333149
HIPK2	0.309482563	0.704757934	0.399289168	1.243919908	0.227422814
HIPK3	0.743203913	0.943924633	0.573425834	1.553808112	0.820475775
HIPK4	0.645343856	2.215396547	0.132089935	37.15636518	0.580331217
HIRA	0.806066096	0.977838707	0.066412971	14.39731617	0.986969611
HIRIP3	0.456989862	79.6618932	0.000114489	55429221.13	0.523598998
HIST1H1A	0.639671519	1.398167121	0.523896734	3.73140577	0.503365451
HIST1H1B	0.100374347	1.32048936	0.747616741	2.332334276	0.338151497
HIST1H1C	0.755408204	1.046904212	0.684261911	1.601738182	0.832682557
HIST1H1D	0.334610297	9.230278567	7.20E-05	1183426.571	0.711112144
HIST1H1E	0.256274738	0.043768588	0.00050632	3.783553657	0.16909005
HIST1H1T	0.212591818	0.000109022	3.19E-10	37.26976598	0.160490343
HIST1H2AA	0.239798901	0.32758484	0.075919749	1.413490284	0.134639606

HIST1H2AB	0.20547272	1.326490987	0.675468926	2.604973036	0.411914906
HIST1H2AC	0.968835364	0.792163777	0.291600024	2.152000676	0.647723273
HIST1H2AD	0.780630463	0.895262433	4.45E-05	18021.82828	0.982542315
HIST1H2AE	0.171545331	0.421723413	0.123284706	1.442600972	0.168830263
HIST1H2AG	0.77938516	0.961562924	0.754654911	1.225200079	0.751204763
HIST1H2AH	0.134546898	0.740672584	0.132844861	4.129598038	0.732048853
HIST1H2AI	0.748135856	0.560063135	0.219179883	1.43111088	0.225856762
HIST1H2AJ	0.103074195	0.430423811	0.094907301	1.95205906	0.274467407
HIST1H2AL	0.620835043	1.22767555	0.488590644	3.084764871	0.662582369
HIST1H2AM	0.964935732	0.753044648	0.328405379	1.726756861	0.502942819
HIST1H2BA	0.331621533	0.729336036	0.228057065	2.332447162	0.5946458
HIST1H2BB	0.259389475	7.324055328	0.093745333	572.2075436	0.370554344
HIST1H2BC	0.805154981	0.850240851	0.046393082	15.58226951	0.912939677
HIST1H2BD	0.140308103	0.908467941	0.352832796	2.339107959	0.84231173
HIST1H2BE	0.072853459	0.980034932	0.784141519	1.224866231	0.859311326
HIST1H2BF	0.270803572	0.089906336	0.00129458	6.243840363	0.265530282
HIST1H2BG	0.248247207	25.26006017	0.003227973	197669.1339	0.480203037
HIST1H2BH	0.59480416	0.018230694	1.13E-05	29.41508642	0.287936523
HIST1H2BI	0.61920949	0.012751441	6.23E-08	2611.135209	0.484498163
HIST1H2BJ	0.174862124	0.68943823	0.34086138	1.394482043	0.3007932
HIST1H2BK	0.629527804	1.141017896	0.843821898	1.542887001	0.391493624
HIST1H2BL	0.251078287	0.781616382	0.172620602	3.539115032	0.749152961
HIST1H2BM	0.269477766	0.835371132	0.00017171	4064.08998	0.966875788
HIST1H2BN	0.794049693	1.014103103	0.765183073	1.343998763	0.922362862
HIST1H2BO	0.512862385	0.386664645	0.000457019	327.1409616	0.78232583
HIST1H3A	0.627274717	0.712811339	0.247508925	2.052855286	0.530472487
HIST1H3B	0.538073715	1.22362717	0.150601137	9.941913353	0.850236716
HIST1H3C	0.764383585	16.06802569	0.001644585	156988.843	0.55357897
HIST1H3D	0.586999841	0.557791997	0.218721182	1.422504716	0.221649327
HIST1H3E	0.892025285	1.518682628	0.66831534	3.451060879	0.318420561
HIST1H3F	0.578632966	0.844570519	0.455643714	1.565476136	0.591604508
HIST1H3G	0.822840082	0.879557899	0.399324914	1.9373249	0.750074862
HIST1H3H	0.549926137	1.247439098	0.58248782	2.671479556	0.569340387

HIST1H3I	0.337965577	0.741159439	0.357859248	1.535009413	0.420039049
HIST1H3J	0.51418642	1.109061831	0.917275566	1.340947247	0.285254006
HIST1H4A	0.101770505	7.806421721	0.758799285	80.3113831	0.084010182
HIST1H4B	0.107417759	0.565369954	0.182336851	1.753036666	0.323294011
HIST1H4C	0.579222186	4.005235049	0.001487144	10787.05569	0.730601933
HIST1H4D	0.242477351	0.720287015	0.309328262	1.677225939	0.446768555
HIST1H4E	0.685742793	0.92566775	0.388559312	2.20522519	0.861554632
HIST1H4F	0.939805224	0.922470278	0.584737765	1.455270149	0.728632646
HIST1H4G	0.19864111	0.096935542	0.000627062	14.98496555	0.364194967
HIST1H4H	0.061309462	1.955710139	0.736841381	5.190808016	0.178047136
HIST1H4I	0.079895078	0.461174031	0.184803238	1.150853677	0.09715019
HIST1H4K	0.156264183	1.217457512	0.505670471	2.931163435	0.660718415
HIST1H4L	0.666801315	1.205381794	0.891706795	1.629398	0.224497965
HIST2H2AB	0.131975068	1.058499642	0.909974371	1.231267085	0.461119135
HIST2H2AC	0.322049418	0.593148794	0.211697113	1.66192862	0.320411081
HIST2H2BE	0.746939481	1.261616944	0.614756871	2.589116751	0.526364717
HIST2H2BF	0.340129502	86.73423406	0.066996112	112287.5213	0.222224796
HIST2H3C	0.452199659	0.547549893	0.21363135	1.403403037	0.209756028
HIST3H2A	0.998444369	1.165276327	0.468654194	2.897379212	0.742053164
HIST3H2BB	0.957029868	0.745966413	0.303620764	1.832766248	0.52280988
HIST3H3	0.541089657	1.153308795	0.746522027	1.781757442	0.520407756
HIVEP1	0.770143795	4.470165247	0.347418563	57.51672319	0.250619496
HIVEP2	0.277139417	1.224087109	0.567308417	2.641225134	0.606338483
HIVEP3	0.799310518	1.800310165	0.686083329	4.724086063	0.232271846
HK1	0.490997561	0.081259714	2.26E-05	292.8079791	0.548022792
HK2	0.350261782	39.73235026	0.2919435	5407.414982	0.141877768
HK3	0.162994529	0.580578098	8.85E-05	3806.970965	0.903483174
HKDC1	0.989735879	0.300046657	0.009422092	9.554990163	0.495400148
HKR1	0.602314138	1703.514817	0.057356761	50594954.78	0.156782599
HLA-A	0.69904087	0.655763824	0.257206357	1.671911218	0.376891924
HLA-B	0.443648554	3.420926069	0.733577618	15.95296105	0.117446002
HLA-C	0.300983728	0.868599271	0.31014882	2.432589277	0.788615273
HLA-DMA	0.988978052	0.889740965	0.121270756	6.527863837	0.90852921

HLA-DMB	0.780119764	0.254332751	0.021265721	3.041756701	0.279543428
HLA-DOA	0.754321055	1.580978563	0.808802501	3.09036287	0.180428726
HLA-DOB	0.993442491	0.216698417	0.000175203	268.0222192	0.673794236
HLA-DPA1	0.441591655	0.038431633	0.000229207	6.44392112	0.212388338
HLA-DPB1	0.695054151	0.478230531	0.003021933	75.68150571	0.775266598
HLA-DPB2	0.215832972	0.795297209	0.596505925	1.060337583	0.118586402
HLA-DQA1	0.994974598	1.101201167	0.266491685	4.550400931	0.894058858
HLA-DQA2	0.896695106	0.894194094	0.646479967	1.236825762	0.499224261
HLA-DQB1	0.622366675	0.236576373	0.001034846	54.08377478	0.602985626
HLA-DQB2	0.724353425	1.108776355	0.686860023	1.789862511	0.672580105
HLA-DRA	0.119867361	0.30056618	0.083446135	1.082614894	0.065980554
HLA-DRB1	0.848456688	0.870756741	0.219167659	3.45953096	0.84412365
HLA-DRB5	0.381036531	0.707300114	0.047820431	10.46150024	0.801084842
HLA-DRB6	0.787314469	1.89567324	4.32E-05	83265.67089	0.906653489
HLA-E	0.685841427	0.105076381	0.001255002	8.797634435	0.318582154
HLA-F	0.544778593	1.969343313	0.022969465	168.8464674	0.765397369
HLA-G	0.989933858	0.92091902	0.695650668	1.219134661	0.564890021
HLA-H	0.900699408	0.813213445	0.407002275	1.624846219	0.558233859
HLCS	0.585199578	0.682732739	0.154485589	3.017265217	0.614697243
HLF	0.325258861	0.59339392	0.269315292	1.307450243	0.195372435
HM13	0.195398261	1.342414145	0.654142135	2.754868767	0.422076908
HMBOX1	0.780023671	0.067065832	0.000205046	21.93574251	0.360377573
HMBS	0.374793663	0.004871707	2.36E-07	100.4659197	0.293504268
HMCN1	0.578766196	0.883889711	0.320088842	2.44076306	0.811758934
HMG20A	0.76694405	0.812526066	0.277749106	2.376960331	0.704637425
HMG20B	0.869562585	1.084011491	0.799621943	1.469545606	0.603340079
HMGA1	0.667766347	0.442485645	0.078816891	2.484157184	0.354313059
HMGA2	0.689479993	1.277303165	0.237207641	6.877954567	0.775696246
HMGB1	0.799237017	0.808044583	0.468088183	1.394899661	0.444180929
HMGB2	0.654615789	0.929139402	0.628801662	1.372928988	0.712171531
HMGB3	0.327881269	5.947160806	0.454925314	77.74621584	0.174012415
HMGB4	0.942276173	0.268815339	0.004817463	14.99994543	0.522022713
HMGCL	0.02887385	0.410662316	0.182639787	0.92336692	0.031332581

HMGCLL1	0.751382372	0.965162855	0.483942618	1.924896264	0.919810503
HMGCR	0.484367903	1.035819736	0.577010944	1.859449176	0.906153005
HMGCS1	0.076602174	3.58297312	0.552321097	23.24317584	0.180988008
HMGCS2	0.718708966	3.013227705	0.000602736	15063.87048	0.79962848
HMGN1	0.689553709	0.12918433	0.000408525	40.85089152	0.485927342
HMGN2	0.188394754	2.362913486	0.220309115	25.34330071	0.477493901
HMGN3	0.217472649	0.334571999	0.034408595	3.253211126	0.345439398
HMGN4	0.828256868	0.294645807	0.000499325	173.8668709	0.707376346
HMHA1	0.875372515	3.502267483	0.089200789	137.508622	0.503282593
HMMR	0.972249313	0.627452358	0.131551759	2.992711494	0.558724895
HMOX1	0.123284028	4.527004081	0.844275023	24.27380342	0.078001759
HMOX2	0.533937383	0.833804153	0.352571287	1.971883108	0.678969466
HMP19	0.055045331	2.012236497	0.255691641	15.83585487	0.506489706
HMX1	0.934590948	1.024935029	0.614456965	1.70962634	0.924833262
HMX2	0.884773222	2.58273796	0.049013939	136.0946611	0.639004522
HMX3	0.605967655	2.430101097	0.031978469	184.6677316	0.687785135
HN1	0.588349549	0.854427625	0.124943542	5.843011596	0.872579038
HNF4A	0.646872074	0.960171384	0.828824202	1.112333694	0.588148463
HNF4G	0.158370588	0.00071926	3.85E-08	13.42506024	0.149199049
HNMT	0.542904081	1.856648253	0.428161536	8.051033186	0.408414544
HOMER1	0.058656071	33.33874558	0.024250152	45833.60852	0.341529261
HOMER2	0.332161315	0.887530494	0.31203695	2.524413786	0.822983572
HOMER3	0.223766411	0.704383692	0.386817536	1.282662596	0.251825499
HOOK1	0.938366143	0.792546978	6.75E-05	9304.212748	0.961214156
HOOK2	0.790216452	1.035929571	0.54826348	1.957361952	0.91341621
HOOK3	0.600303908	0.827687936	0.172079075	3.981119271	0.813439774
HORMAD1	0.550052005	1.469140171	0.650228794	3.319405203	0.354979628
HORMAD2	0.547839217	1.135982277	0.246431641	5.236566729	0.87010653
HOXA1	0.929397931	0.921276756	6.81E-05	12466.31262	0.986521353
HOXA10	0.584670803	1.081655812	0.672836911	1.73887502	0.745896546
HOXA11	0.196444967	1.118543007	0.31583054	3.961423297	0.862156013
HOXA13	0.969143228	0.861748475	0.689639639	1.076809384	0.19055108
HOXA2	0.336200615	0.472442499	0.151900893	1.469391719	0.195248109

HOXA3	0.753497525	1.122907601	0.223543063	5.640620038	0.888057147
HOXA4	0.59852306	1.611300041	0.412188278	6.298791013	0.49282931
HOXA5	0.08731448	1.014063573	0.099492434	10.33570982	0.990593178
HOXA6	0.071980265	0.419051454	0.054275813	3.235402856	0.404259971
HOXA7	0.813660305	2.06720353	0.722030595	5.918489413	0.176019272
HOXA9	0.878105975	0.389477131	3.72E-06	40811.96009	0.872976519
HOXB1	0.142776947	0.698601792	0.04006685	12.18075454	0.805739075
HOXB13	0.534269271	0.680094245	0.227310362	2.034787052	0.490520434
HOXB2	0.549174519	0.340397894	0.071639694	1.617409564	0.17533285
HOXB3	0.893388523	1.345868099	0.457668524	3.957800996	0.589378906
HOXB4	0.174293238	0.522156406	0.005234619	52.08541534	0.782010197
HOXB5	0.8862569	0.765660592	0.193543791	3.028958654	0.703539033
HOXB6	0.77457153	1.114343617	0.036375012	34.13776699	0.950557254
HOXB7	0.578464216	0.92640754	0.795360976	1.079045813	0.325942173
HOXB8	0.464615628	0.809248998	0.31521583	2.077573137	0.659960086
HOXB9	0.626365747	0.556740263	0.098879659	3.13471672	0.506562953
HOXC10	0.573449227	0.846959938	0.348469329	2.058548854	0.713938338
HOXC11	0.37017216	0.629509997	0.173469857	2.284447815	0.481584686
HOXC12	0.29949051	1.309023685	0.910649958	1.881670331	0.145826249
HOXC13	0.891812548	0.651856934	0.273749963	1.552210121	0.333688747
HOXC4	0.332267588	0.594645634	0.236720696	1.493757985	0.268703374
HOXC5	0.808589597	1.411137361	0.61187731	3.254424737	0.419212372
HOXC6	0.110727432	2.627524172	0.820792761	8.411238015	0.103673278
HOXC8	0.082610029	1.775292512	0.964451088	3.267831352	0.06522768
HOXC9	0.466039008	0.3536134	0.081661031	1.53123755	0.16447488
HOXD1	0.089867435	207.3150482	0.615057473	69878.88299	0.072448182
HOXD10	0.229017969	0.940738998	0.794560658	1.113810321	0.478324706
HOXD11	0.499697713	1.020787133	0.885623599	1.176579274	0.776487166
HOXD12	0.421918328	1.776255289	0.675218116	4.672686913	0.244356278
HOXD13	0.11884674	0.646199664	0.307207097	1.359258981	0.249763267
HOXD3	0.289157552	1.00862625	0.820080299	1.240521097	0.935164077
HOXD4	0.218847864	0.352456214	0.070925101	1.751500968	0.202376141
HOXD8	0.271875588	1.072237549	0.223522585	5.143522115	0.930525679

HOXD9	0.17429324	2.504861636	0.823528173	7.618842955	0.105690514
HP	0.135188457	49.95261404	0.007261087	343648.7969	0.385663462
HP1BP3	0.924629716	0.908108301	0.042104813	19.58590068	0.950949285
HPCA	0.852310754	1.66963415	0.262817652	10.60689104	0.58685647
HPCAL1	0.687789381	1.445564797	0.711455473	2.937158629	0.308313822
HPCAL4	0.737908468	1.010474205	0.765702471	1.333492	0.941308501
HPD	0.224970333	0.011367268	2.11E-06	61.38200052	0.307244411
HPN	0.77753173	0.966725326	0.298301667	3.132928706	0.955015532
HPR	0.698137431	0.657582519	0.138406356	3.124240691	0.598049559
HPRT1	0.193005256	9.99E-05	9.21E-09	1.08374381	0.052016188
HPS1	0.282011496	0.394206313	0.10924939	1.422420911	0.155088687
HPS3	0.195020104	4022.492598	0.471176608	34340513.57	0.072331014
HPS4	0.909994208	0.453454262	0.06616589	3.10765516	0.420624317
HPS5	0.355791699	0.064373555	9.05E-05	45.79138218	0.412978492
HPS6	0.211568297	0.883435452	0.654101993	1.193175081	0.418968465
HPSE	0.744528945	3.310851831	0.210204039	52.14809333	0.3946942
HPSE2	0.828665357	56.98381996	0.000897755	3616971.663	0.47366319
HPX	0.217214857	0.042381513	2.95E-07	6092.513083	0.60188539
HR	0.674903208	0.668863287	0.05249415	8.522437124	0.756758925
HRAS	0.92872646	1.41633936	0.563678961	3.558793783	0.459023683
HRASLS	0.190565578	0.159486005	0.018276582	1.391714609	0.096730446
HRASLS2	0.541478022	1.140973466	0.311194167	4.18330608	0.84229958
HRASLS5	0.52751076	0.974104384	0.801789385	1.183452125	0.791662513
HRC	0.59580467	1.262320624	0.356306313	4.472144607	0.71813352
HRG	0.175248209	0.008561767	2.26E-05	3.247690616	0.11613991
HRH1	0.604098172	0.817473464	0.343244327	1.946901413	0.648970882
HRH2	0.52732664	0.697509587	4.28E-05	11378.47009	0.941972155
HRH3	0.104492523	5.460813916	0.30377828	98.16530859	0.249457753
HRH4	0.585708137	1.246872424	0.287645814	5.404879083	0.768109808
HRK	0.061140344	0.865989983	0.512065323	1.464537077	0.591461854
HRNR	0.214718067	0.845146284	0.505657222	1.412562126	0.52088398
HRSP12	0.718246924	0.735044425	0.258164228	2.09281631	0.56420463
HS1BP3	0.958902954	0.031942601	7.19E-05	14.19035076	0.26821851

HS2ST1	0.365612244	0.875229867	0.507694931	1.508833895	0.631500167
HS3ST1	0.217528434	0.399800612	0.104888338	1.523911362	0.179309108
HS3ST2	0.58493505	0.037252524	1.12E-05	124.0398438	0.426585265
HS3ST3A1	0.71187698	1.952108623	0.483931564	7.87451854	0.347217129
HS3ST3B1	0.552811267	1.050988325	0.290754776	3.798996789	0.939536438
HS3ST4	0.081811184	0.452027138	0.12308554	1.660053114	0.231575009
HS3ST5	0.467532274	0.999761864	0.770316451	1.297549576	0.998571442
HS3ST6	0.98166703	0.927739227	0.008225502	104.6380007	0.975182551
HS6ST1	0.514343012	1.174281719	0.945490502	1.458436179	0.146218115
HS6ST2	0.543998365	0.432663384	0.033176363	5.64249931	0.522565005
HS6ST3	0.719573908	0.853031075	0.090125645	8.07386193	0.889752755
HSBP1	0.984578817	0.759992376	0.289356163	1.99611581	0.577499904
HSD11B1	0.524606936	0.87743767	0.488628693	1.575627619	0.66156275
HSD11B2	0.1837232	0.555324877	0.057469632	5.366063909	0.611280219
HSD17B1	0.981346762	2.975100564	0.008063126	1097.740888	0.717703479
HSD17B12	0.403774073	0.074187834	0.000974272	5.649175308	0.23932229
HSD17B13	0.771865361	2.319855142	0.53213371	10.11348798	0.262635645
HSD17B2	0.105164539	1.350962369	0.809041749	2.2558778	0.250175498
HSD17B3	0.262929547	0.859184005	0.389537461	1.895060754	0.706876187
HSD17B4	0.67983629	0.03883546	3.56E-05	42.32324851	0.362636334
HSD17B6	0.325397691	2.043561452	0.323979531	12.89014588	0.446920299
HSD17B7	0.255254783	3.046944894	0.834958453	11.11896426	0.091628583
HSD17B8	0.305724979	1.268261939	0.780967613	2.059609539	0.336737564
HSD3B1	0.072875926	1.644124733	0.593955656	4.551090828	0.338501751
HSD3B2	0.568245551	0.002856808	1.05E-07	77.74178663	0.260850812
HSD3B7	0.419099569	0.714745781	0.2243524	2.277049552	0.569996985
HSDL1	0.054746509	1.959795075	0.909948702	4.220893694	0.085634969
HSDL2	0.435986365	1.25167804	0.106189271	14.75382497	0.858451251
HSF1	0.320792548	1.028795925	0.85885012	1.232369922	0.757946418
HSF2	0.360651292	0.004971799	5.76E-08	429.2162042	0.360387166
HSF2BP	0.149653806	11.19486992	0.033246531	3769.56959	0.415908042
HSF4	0.851092417	0.597510478	0.001057089	337.7375169	0.873454312
HSFY1	0.014888808	0.438771577	0.191645632	1.004565011	0.051273095

HSH2D	0.598483577	1.274544514	0.690613966	2.35220224	0.437786324
HSP90AA1	0.996867023	0.572360913	0.210201432	1.55849088	0.27493346
HSP90AB1	0.908681342	0.119807103	3.41E-05	420.5395732	0.610441677
HSP90AB2P	0.158066207	0.12630391	8.43E-06	1892.492015	0.673185803
HSP90AB6P	0.45504649	1.236740768	0.244608262	6.252968388	0.797195947
HSP90B1	0.832504451	190.8798985	0.014287005	2550229.021	0.278599135
HSPA12A	0.198689875	0.985680716	0.574241081	1.691913912	0.958272918
HSPA12B	0.233172553	0.782594958	0.123517583	4.958442779	0.794677714
HSPA14	0.096357391	0.388508349	0.046218203	3.265785538	0.38408201
HSPA1A	0.733795409	1.248511141	0.613637295	2.540230333	0.540247174
HSPA1B	0.582196917	0.076365555	0.000254075	22.95269433	0.376917355
HSPA1L	0.330109252	0.565109645	0.099563052	3.207504241	0.519392522
HSPA2	0.204086792	0.712599011	0.34831743	1.457857993	0.353523197
HSPA4	0.866058216	0.637127634	0.161965789	2.506280036	0.518860631
HSPA4L	0.820491967	0.753987373	0.246186839	2.309209385	0.620973549
HSPA5	0.756195753	0.354207757	0.002310922	54.29137614	0.686042151
HSPA6	0.694431886	1.350767721	0.610545637	2.988430881	0.458007556
HSPA8	0.67364312	1.591621983	0.157231729	16.11163702	0.693940275
HSPB1	0.76418684	1.112008757	0.9183354	1.34652707	0.276860055
HSPB2	0.395832393	1.333663404	0.256941131	6.922434215	0.731842303
HSPB3	0.365981727	0.277014597	0.052095936	1.472995632	0.132147733
HSPB6	0.546987253	1.451012954	0.411365723	5.118167298	0.562712066
HSPB7	0.170148913	1.710279117	0.208539047	14.02641235	0.617180555
HSPB8	0.238344954	0.503333027	0.157382505	1.609735055	0.247123088
HSPB9	0.594805274	0.150135931	0.000238387	94.55543404	0.564199885
HSPBAP1	0.76709312	0.669957714	0.111117204	4.039368558	0.662144283
HSPBP1	0.131392394	0.009673583	8.27E-05	1.131665609	0.056254943
HSPC047	0.953965715	0.582966609	0.248436048	1.36795795	0.214977488
HSPD1	0.410981746	63.52662338	0.007419447	543926.2434	0.3688782
HSPE1	0.347413938	1.163015106	0.553436784	2.44400838	0.690211896
HSPG2	0.697600201	0.404004123	0.056744591	2.876385742	0.365470261
HSPH1	0.439882048	2.80031552	0.389105831	20.15330118	0.306497575
HTATIP2	0.532620388	0.657491112	0.352239604	1.227274155	0.187895304

HTATSF1	0.699632359	0.83455962	0.470528554	1.480228463	0.53620931
HTN1	0.364442392	0.48428568	5.45E-05	4303.163581	0.875794598
HTN3	0.125398679	1.787666681	0.781569872	4.088888628	0.168778588
HTR1A	0.351272739	26.5704709	0.471829139	1496.28301	0.110770865
HTR1B	0.58330673	0.52613198	0.170495966	1.623585984	0.263988891
HTR1D	0.424377908	0.950212297	0.79310435	1.138442134	0.579691326
HTR1E	0.220332711	0.000148462	1.52E-09	14.52252141	0.132690039
HTR1F	0.521225176	1.235365775	0.625258023	2.440798107	0.54294454
HTR2A	0.395669581	1.238137267	0.83871817	1.827769979	0.282416288
HTR2B	0.810702006	0.385870615	4.52E-06	32934.96058	0.869437408
HTR2C	0.123807376	0.571349436	0.30570384	1.06783146	0.07938275
HTR3A	0.923142235	1.539736264	0.046772338	50.6878185	0.80869672
HTR3B	0.918258357	1.140874947	0.668125794	1.948129612	0.629263622
HTR3C	0.415048901	1.091760242	0.452897655	2.631809669	0.844956026
HTR3D	0.050368033	3.698205328	0.809229366	16.90092232	0.091615608
HTR3E	0.889244769	11.66690863	0.001235953	110130.9929	0.598823664
HTR4	0.658267342	0.925766682	0.259305979	3.305145344	0.905439102
HTR5A	0.273777653	8.7212396	0.007117473	10686.38019	0.550547863
HTR6	0.696535866	0.912238012	0.351353122	2.368495222	0.850335503
HTR7	0.568451872	0.837630953	0.380587821	1.843531438	0.659787319
HTRA1	0.389506903	14.33551127	0.05655408	3633.811771	0.345765154
HTRA2	0.250452754	0.95973913	0.406454119	2.266182467	0.925314032
HTRA3	0.381239	0.56261049	0.20690571	1.529830006	0.259766786
HTRA4	0.754294513	1.106167981	0.857715603	1.426588949	0.436910403
HUNK	0.670896663	0.75773766	0.296256721	1.938070336	0.562600527
HUS1	0.468291151	0.807803999	0.115468175	5.651317367	0.82973455
HUS1B	0.107549971	0.534708336	0.08872404	3.222497584	0.4945345
HUWE1	0.786986881	0.648224587	0.069291024	6.064207068	0.703935629
HYAL1	0.4847445	0.871758162	0.133991327	5.671727507	0.885788189
HYAL2	0.779075059	0.693930542	0.195615611	2.461662411	0.571684999
HYAL3	0.073067987	0.233132948	0.041180976	1.319807757	0.09971206
HYAL4	0.648612341	1.535827304	0.300332456	7.853848146	0.606332969
HYDIN	0.530103042	1.470677015	0.087556921	24.70268325	0.788719991

HYI	0.173028489	0.256838528	0.0213204	3.094033373	0.284403046
HYLS1	0.414861749	2.826139013	0.369815638	21.59741477	0.316700054
HYMAI	0.864199197	0.96840314	0.808640566	1.159729899	0.727065962
HYOU1	0.552448509	1.243760799	0.491958811	3.144452122	0.644823009
HYPK	0.32393333	1.942516072	0.723005877	5.219001409	0.187918408
IAPP	0.65814646	1.624111682	0.307722047	8.571822452	0.567740282
IARS	0.198703921	1.224077839	0.842064665	1.779396072	0.289449544
IARS2	0.914039214	1.356050612	0.020048958	91.7191436	0.887351973
IBSP	0.558649024	1.731074548	0.000551832	5430.314897	0.893728679
IBTK	0.484805418	0.548592032	0.169383972	1.776751453	0.316661979
ICA1	0.105226348	1.358482437	0.96369664	1.914995294	0.080312733
ICAM1	0.493955697	0.013181544	5.31E-06	32.73777419	0.277773816
ICAM2	0.081241773	0.526289787	0.164943829	1.679244026	0.278212127
ICAM3	0.831041751	1.227171491	0.500915103	3.006397409	0.654309221
ICAM5	0.53630161	0.912374644	0.46677511	1.783358777	0.788557782
ICK	0.077909559	1.210873296	0.46273215	3.168602267	0.696641791
ICMT	0.979638654	0.612897269	0.102466241	3.666017781	0.59165254
ICOS	0.449771369	1.412670853	0.590822062	3.377732601	0.437291339
ICOSLG	0.847276268	1.149618242	0.855588052	1.544694436	0.354902248
ICT1	0.434548555	2.480756186	0.163021911	37.75045466	0.513045617
ID1	0.418532018	4.921912349	0.008607195	2814.531563	0.622724594
ID2	0.621400885	0.878357025	0.057030983	13.52792848	0.925930617
ID3	0.055268198	1.280048678	0.345152662	4.747246062	0.711971093
ID4	0.853718791	1.293594502	0.998438926	1.6760031	0.051398113
IDE	0.222585588	0.000701674	1.84E-07	2.680311287	0.084405539
IDH1	0.49964983	119.9907881	0.008632609	1667837.438	0.325312878
IDH2	0.14607708	1.209806164	0.75080792	1.949407986	0.433931592
IDH3A	0.849638149	0.994506344	0.442345968	2.23590343	0.989366781
IDH3B	0.677465829	0.701583998	0.225277853	2.184946725	0.540884805
IDH3G	0.168479928	1.399859845	0.009538936	205.4325041	0.894863344
IDI1	0.513640747	44.59304336	0.173495437	11461.62433	0.179823173
IDI2	0.114870027	0.479533076	0.197044216	1.167006958	0.105315051
IDS	0.084372927	1.221499442	0.95818391	1.557175894	0.106279931

IDUA	0.780760991	0.678281625	0.198876082	2.313329782	0.535162429
IER2	0.69996029	0.906312368	0.239199661	3.433960172	0.884917298
IER3	0.965293276	1.000292425	0.744509851	1.343951237	0.998451739
IER3IP1	0.955876735	1.193611915	0.583769688	2.440533368	0.627682012
IER5	0.230773599	44.31415392	0.211464553	9286.399123	0.164456923
IER5L	0.094789543	2.06506447	0.614111507	6.944164402	0.241210697
IFI16	0.790727058	0.630837362	0.275721406	1.443325651	0.275275229
IFI27	0.585432545	1.420863528	0.682426457	2.958345392	0.347845338
IFI30	0.562856939	0.022314424	3.21E-05	15.53043855	0.254850877
IFI35	0.165108671	0.635463432	0.280291037	1.440694561	0.277625064
IFI44	0.325797905	0.62322596	0.196114693	1.980527777	0.422812477
IFI44L	0.673845642	2.204912788	0.871797025	5.576573749	0.094887537
IFIH1	0.378312164	5.318672701	0.001476635	19157.25516	0.689169718
IFIT1	0.645674266	1.05477029	0.393233666	2.829209351	0.915643705
IFIT2	0.818748475	0.023586428	5.00E-06	111.2454614	0.385271962
IFIT3	0.591108609	1.111836477	0.337674395	3.66086493	0.861582881
IFIT5	0.620656677	0.038144999	2.97E-07	4907.117614	0.586330545
IFITM1	0.836178532	4.130457831	0.000592673	28785.97789	0.753407433
IFITM2	0.682367215	0.945606483	0.509307797	1.755660576	0.859387292
IFITM3	0.478725142	0.031919537	2.08E-05	49.07694828	0.357553094
IFITM4P	0.708170248	0.02843942	0.000301507	2.682528852	0.124881839
IFITM5	0.508138683	0.883155655	0.298024535	2.617113088	0.822616876
IFNA1	0.098018565	0.594500585	0.267254796	1.322449403	0.202371183
IFNA10	0.666256313	1.840436247	0.526208629	6.43700121	0.339631921
IFNA13	0.472335524	0.004137969	2.11E-07	80.96533953	0.276405235
IFNA14	0.054737956	38.67894297	0.028798083	51950.00668	0.319903811
IFNA16	0.373644093	1.211618586	0.38251193	3.837840038	0.744183835
IFNA17	0.55357967	0.409288899	0.005884942	28.46542908	0.679788588
IFNA2	0.780100524	0.369754231	0.000569052	240.2561508	0.763351195
IFNA21	0.48168025	0.007669523	2.02E-08	2913.279948	0.457468147
IFNA4	0.353029012	0.044319971	0.000431175	4.555594693	0.187358605
IFNA5	0.570709548	271.1590459	0.00326219	22539220.32	0.332359837
IFNA6	0.804818397	0.701549135	0.240441318	2.046949302	0.516472097

IFNA7	0.335632455	4.345037317	0.111727563	168.9766494	0.431559699
IFNA8	0.279005608	2.739516267	0.653631015	11.48193584	0.168084834
IFNAR1	0.104016159	1.910920108	0.931877612	3.918557129	0.077160156
IFNAR2	0.510344716	2.120425798	0.858544716	5.237008021	0.103240753
IFNB1	0.235604826	1.214781226	0.888581844	1.660728764	0.222642462
IFNG	0.123356076	6.468570481	0.039471224	1060.073627	0.473001171
IFNGR1	0.032615795	0.379721423	0.164515537	0.8764422	0.023268251
IFNGR2	0.34658812	0.004037586	4.03E-06	4.043890938	0.117907187
IFNK	0.85335701	1.027634836	0.844629949	1.250291155	0.785290001
IFNW1	0.469865731	105.7309907	0.00092516	12083367.4	0.432819397
IFRD1	0.932322165	1.111841836	0.272326143	4.539381538	0.882573349
IFRD2	0.302325387	10.15379544	0.198774164	518.6768742	0.248113119
IFT122	0.662216403	1.034914957	0.349949797	3.060578914	0.950534301
IFT140	0.284841925	0.969789086	0.350362472	2.684336779	0.952907786
IFT172	0.116208205	29.05331267	0.093916707	8987.697777	0.249517616
IFT20	0.200945343	1.043394104	0.848629135	1.282858686	0.686974543
IFT52	0.907320663	0.881183076	0.363758861	2.134610856	0.779322463
IFT57	0.121893622	0.458795435	0.094558651	2.226060226	0.333593693
IFT74	0.771544543	1.249725903	0.391639609	3.987887833	0.706509061
IFT80	0.312683505	2.075249932	0.876491327	4.913525269	0.096876432
IFT81	0.371407943	0.93570702	0.707425883	1.237652804	0.641422289
IGBP1	0.13370778	1.464052054	0.674518543	3.177745727	0.334988663
IGF1	0.67120268	1.315638012	0.55372757	3.1259115	0.534413593
IGF1R	0.459452735	47.9476767	0.050884052	45180.75165	0.26802943
IGF2	0.672867848	7.442144764	0.007690887	7201.447912	0.567171299
IGF2BP1	0.074214854	1.159082167	0.70114078	1.916122281	0.564876565
IGF2BP2	0.953001532	1.865838707	0.399858586	8.706463235	0.42741899
IGF2BP3	0.330699696	0.825182014	0.416769166	1.633818939	0.581395618
IGF2R	0.46826665	0.455076467	0.120777849	1.714673615	0.244729938
IGFALS	0.533032395	0.00163559	7.95E-08	33.63446048	0.205454953
IGFBP1	0.625447478	0.969815515	0.695463266	1.352396566	0.856640822
IGFBP2	0.224666944	0.751548841	0.129034046	4.377338201	0.750713941
IGFBP3	0.657809632	1.327976189	0.730793755	2.413157949	0.351950581

IGFBP4	0.285815574	1.183736934	0.489160442	2.864567549	0.708336658
IGFBP5	0.854503523	1.064237832	0.360888752	3.138369251	0.910161073
IGFBP6	0.583393792	1.11715425	0.486775868	2.563877344	0.793803161
IGFBP7	0.680719079	1.097734042	0.704327482	1.71088032	0.680448761
IGFBPL1	0.186645645	0.996503817	0.359721053	2.760527492	0.994624764
IGFL1	0.879233023	0.252430293	0.015295411	4.166024217	0.335856184
IGFL2	0.585276412	1.04059629	0.768631504	1.408790341	0.796822858
IGFL3	0.715878756	1.190481751	0.604005747	2.346412773	0.614513493
IGFL4	0.527676619	1.342815025	0.012681289	142.1899784	0.901383557
IGHMBP2	0.68973911	0.755219232	0.281931911	2.023027781	0.576544147
IGJ	0.203321551	1.491534308	0.176604266	12.59694707	0.71342507
IGLL1	0.524342356	1.376957346	0.775243343	2.445698565	0.275107464
IGSF1	0.239111228	1.804936053	0.609053745	5.348943637	0.28670109
IGSF10	0.536366533	0.779210193	0.167914	3.615949391	0.750047423
IGSF11	0.841026353	1.644625647	0.669283873	4.041324806	0.278105303
IGSF21	0.690486652	0.01494699	8.71E-06	25.65115655	0.268673925
IGSF22	0.181762747	1.862764776	0.961324069	3.609493116	0.065314868
IGSF3	0.696322634	1.239745673	0.462709118	3.321675056	0.66910358
IGSF6	0.576270366	0.936024427	0.683902816	1.281090978	0.679675606
IGSF8	0.46040296	1.145930572	0.253174413	5.186767739	0.859649218
IGSF9	0.248895224	5.027992388	0.578800742	43.67773847	0.143133123
IGSF9B	0.620401329	1.110508681	0.652451446	1.890147596	0.699286573
IHH	0.974162434	0.880015584	0.232354649	3.332954301	0.850782912
IK	0.477741973	0.062067844	0.000197565	19.49947362	0.343408053
IKBKAP	0.338107933	0.681132971	0.327905469	1.414865465	0.303229299
IKBKB	0.906700912	3.710969918	0.000645576	21331.78966	0.766549461
IKBKE	0.261971137	1.310644458	0.882547535	1.946398155	0.180007958
IKBKG	0.690719094	9.272095761	0.141686107	606.7762174	0.296514489
IL10	0.133132973	1.221863245	0.816106465	1.82935665	0.330502755
IL10RA	0.659048321	0.853403668	0.195478619	3.725716023	0.833029873
IL10RB	0.791434034	1.196102407	0.439124318	3.257986205	0.726149204
IL11	0.93168218	0.999525258	0.827336338	1.207550904	0.996072409
IL11RA	0.707762511	141.9762995	0.000128588	156758795	0.485152322

IL12A	0.360718425	0.145715239	0.002532593	8.38387029	0.351560786
IL12B	0.533514838	0.302990746	0.017925148	5.121485814	0.407844199
IL12RB1	0.548873794	1.349845906	0.158445345	11.49976334	0.783736548
IL12RB2	0.065465643	9.891147961	0.027858079	3511.900699	0.444348226
IL13	0.137555432	1.189445492	0.304238024	4.650242465	0.803057774
IL13RA1	0.054092682	7536.140303	0.036158067	1570698198	0.153096025
IL13RA2	0.861616333	11.34290069	0.418539947	307.4052951	0.149134373
IL15	0.179166791	0.152029399	0.008306189	2.782616325	0.204088924
IL15RA	0.630575442	2.346534327	0.417969878	13.17373245	0.332565687
IL16	0.789132762	1.184006235	0.384348293	3.647396877	0.768579254
IL17B	0.314753696	1.005318138	0.192636686	5.246480186	0.994979858
IL17C	0.822677317	1.051582085	0.742639838	1.489046001	0.776870023
IL17D	0.164389867	0.65894729	0.309612519	1.402435318	0.279096309
IL17F	0.788138885	1.257029847	0.706240365	2.23737429	0.436785692
IL17RB	0.919351952	0.331514062	1.43E-05	7690.604346	0.829548488
IL17RC	0.862325176	1.081909081	0.394315305	2.968505776	0.878496217
IL17RD	0.909330944	1.165190366	0.72902005	1.862319958	0.522827988
IL17RE	0.300497385	2.938170354	0.254232203	33.95653628	0.388045624
IL18	0.424484697	11.29892928	0.107604012	1186.440922	0.307193275
IL18BP	0.317442344	0.056055682	0.000835758	3.759747811	0.179339622
IL18R1	0.149255882	9.34E-05	3.97E-10	21.96391428	0.141458183
IL18RAP	0.982662518	0.922703637	0.30515202	2.790025773	0.886686783
IL19	0.874464867	0.967329616	0.712643193	1.313036586	0.831280575
IL1A	0.215050408	0.298503988	0.000266813	333.9592686	0.735708384
IL1B	0.493009579	0.029952579	0.000222265	4.03642052	0.160846846
IL1F10	0.737087696	0.782853936	0.141055901	4.344804286	0.779497226
IL1R1	0.68546563	0.133725221	0.007072119	2.528582273	0.179771901
IL1R2	0.366273018	1.064705995	0.853636251	1.327964755	0.578088169
IL1RAP	0.17859105	0.493154287	0.140284333	1.733630162	0.270398232
IL1RAPL1	0.846032303	3.212246069	0.191227197	53.95950448	0.417533558
IL1RAPL2	0.077598798	0.014105211	4.72E-05	4.218112506	0.142899721
IL1RL1	0.324227275	0.732468279	0.286081319	1.87537509	0.516302314
IL1RL2	0.911320054	0.821985021	0.304133695	2.221586712	0.699172313

IL1RN	0.676867651	1.262262954	0.039308182	40.53374313	0.895315031
IL2	0.828538356	1.063882515	0.77586933	1.458810087	0.700641833
IL20	0.280680436	0.000428191	2.84E-09	64.51602801	0.202317414
IL20RA	0.869973197	1.197928742	0.319144169	4.496504739	0.78900633
IL21	0.825187204	0.937555405	0.331132767	2.65455498	0.903351754
IL21R	0.563217851	1.459109473	0.612061402	3.47840992	0.393989363
IL22	0.069839268	3.189192978	0.98985648	10.27517833	0.052030124
IL22RA1	0.831523476	1.652446334	0.28161858	9.696018249	0.577984345
IL22RA2	0.12036642	1.166436312	0.922456879	1.474945552	0.198502729
IL23A	0.800167304	1.024183593	0.662132873	1.584201714	0.914492283
IL23R	0.601015634	1.116785859	0.972818906	1.282058404	0.116738479
IL24	0.618156099	1.595922446	0.089681693	28.40009349	0.750304246
IL26	0.537871392	0.019001256	1.78E-05	20.23799506	0.265135012
IL27	0.420747892	0.826148867	0.368602321	1.85164854	0.642789595
IL27RA	0.559943938	1.455877466	0.361123192	5.869407573	0.597463899
IL2RA	0.356497803	0.834255151	0.377534603	1.843491039	0.654182431
IL2RB	0.459954379	0.248910059	0.056521088	1.096161092	0.065977321
IL2RG	0.597894994	1.785260225	1.16E-05	274499.5488	0.924226602
IL3	0.588366477	0.366469852	0.003388769	39.63095783	0.674416903
IL31	0.930082466	0.974310225	0.247896698	3.829338679	0.970271488
IL31RA	0.27631096	0.050144883	0.001140493	2.20475562	0.121046369
IL32	0.871043372	1.046827507	0.794049241	1.380075407	0.745524553
IL3RA	0.755005244	1.068526405	0.753282962	1.515696936	0.710195032
IL4	0.766604016	1.134031357	0.859151014	1.496858057	0.374496925
IL4I1	0.971575754	0.845821853	0.362683322	1.97255998	0.698331544
IL4R	0.081380667	0.813070049	0.467996051	1.41258223	0.46277143
IL5	0.205007965	0.619193186	0.240089879	1.596902809	0.321372414
IL5RA	0.415136002	0.59262421	0.234742599	1.496121517	0.268162076
IL6	0.991421417	0.838752052	0.464663362	1.514010063	0.55952928
IL6R	0.921442291	0.888591708	0.460747408	1.71372689	0.724476837
IL6ST	0.839363376	1.763186218	0.457885591	6.789524941	0.409697378
IL7	0.374815833	1.30366685	0.825015439	2.060018729	0.25596906
IL7R	0.737034501	11.31745536	0.017383825	7368.044389	0.462921716

IL9	0.333502541	0.047387706	8.29E-07	2709.583572	0.585326598
IL9R	0.248828314	11.33146811	0.475616718	269.9698407	0.133460344
ILDR1	0.179145913	0.218464798	0.041200548	1.158403702	0.073905033
ILF2	0.594825085	5.093556224	0.279838644	92.71169498	0.271466598
ILF3	0.836999431	0.320036913	0.002607673	39.27778485	0.642469866
ILK	0.355393229	1.118388896	0.401988665	3.111514906	0.83029592
ILKAP	0.324299045	0.282581494	0.001831031	43.61055842	0.623035247
ILVBL	0.549113789	1.151519764	0.450712799	2.942001575	0.768153023
IMMP1L	0.876040878	0.543634577	0.165016117	1.790967804	0.316369396
IMMP2L	0.315169551	1.685367259	0.774723502	3.666421362	0.188074404
IMMT	0.235961742	1.111137518	0.837026536	1.475014867	0.465924976
IMP3	0.337492487	0.979383708	0.704921499	1.360708176	0.901185897
IMP4	0.588404819	0.237347719	0.000256407	219.7052572	0.679834664
IMPA1	0.140004546	0.186812484	0.006961571	5.013079031	0.317541051
IMPA2	0.904729725	2.142704999	0.03743865	122.6322183	0.712083461
IMPACT	0.132382978	1.751738856	0.657839077	4.664649952	0.261913598
IMPAD1	0.333877204	10.26538287	0.009173042	11487.8017	0.515586567
IMPDH1	0.877450251	1.677248095	0.003356772	838.0554079	0.8704255
IMPDH2	0.917485575	0.959867695	0.49370353	1.866192841	0.903891559
IMPG1	0.645487029	5.15614619	0.220001993	120.8436488	0.308131893
IMPG2	0.42555419	0.028435333	0.000523851	1.54350668	0.080642526
INA	0.636454066	1.809750504	0.726666314	4.507153865	0.202611569
INADL	0.053203441	0.737500089	0.464437168	1.171108642	0.196870445
INCA1	0.953792275	0.983898548	0.324125644	2.986670041	0.977142027
INCENP	0.994237692	1.173459838	0.65772609	2.093588827	0.58813672
INE1	0.066013512	0.16985481	0.000111752	258.1657619	0.635312416
ING1	0.731069438	1.391258947	0.745713057	2.595638415	0.299362103
ING2	0.296037799	0.513509968	0.221599916	1.189948499	0.120095585
ING3	0.872999321	0.720727948	0.171690235	3.02549982	0.654561622
ING4	0.872140899	0.498103467	0.100501459	2.468691165	0.393434225
ING5	0.780591269	1.38217502	0.548124217	3.485355555	0.492802223
INGX	0.424519635	1.122860198	0.187162721	6.736464488	0.899126514
INHA	0.373059256	30.60271418	0.13474023	6950.604974	0.21650663

INHBA	0.129695181	0.627788152	0.291770169	1.350782246	0.233715776
INHBB	0.346380645	0.155800578	0.005681408	4.272500504	0.271146993
INHBC	0.133836085	1.21624078	0.915795206	1.615253744	0.176270917
INHBE	0.84472097	1.011850513	0.869803533	1.177095082	0.878680251
INMT	0.911455297	1.259050527	0.251789891	6.29575803	0.779082817
INPP1	0.283816865	2.360701042	0.406294744	13.71642013	0.338694273
INPP4A	0.71113734	39.84517103	6.86E-05	23149958.09	0.586325941
INPP4B	0.97340512	2.528827699	0.00036798	17378.5912	0.836940427
INPP5A	0.39170795	1.267930435	0.190527394	8.437881557	0.80608535
INPP5B	0.256627211	1.747368249	0.914148333	3.340044153	0.09133213
INPP5D	0.865975962	0.371709758	0.041637008	3.318397525	0.375593216
INPP5E	0.275153336	0.377647723	0.073746421	1.933894561	0.242591617
INPP5F	0.629999217	1.415638236	0.461774231	4.339851551	0.543112083
INPPL1	0.711935029	0.909737445	0.564139624	1.4670521	0.69801037
INS	0.948193849	2.910511127	0.876561553	9.663981942	0.081020946
INSIG1	0.181536918	0.197020924	0.000649513	59.76356942	0.577445228
INSIG2	0.888409271	1.011561013	0.708794008	1.443657357	0.949495542
INSL3	0.19356456	1.685476318	0.717900337	3.957137605	0.230581767
INSL4	0.238889565	0.696424606	0.335134237	1.447202877	0.332304779
INSL5	0.837520018	0.772524887	0.319049688	1.870538425	0.567306932
INSL6	0.851409622	1.005872501	0.909211173	1.112810223	0.909563892
INSM1	0.520102571	0.992713573	0.768035444	1.283118176	0.955455229
INSM2	0.564579566	0.769461096	0.111403455	5.31465006	0.790404915
INSR	0.197540777	1.553868334	0.850582083	2.838652316	0.151692186
INSRR	0.30047921	0.885649784	0.065435824	11.98694382	0.92720964
INVS	0.304553423	0.948140261	0.843806849	1.06537409	0.370623946
IPMK	0.679228237	2.478165679	0.159588538	38.48211919	0.516642969
IPO11	0.61316893	1.144040698	0.844736783	1.549392836	0.384521475
IPO13	0.77065944	1.176937803	0.56055777	2.471079103	0.666840949
IPO4	0.973133251	1.001215812	0.363303991	2.75921302	0.998125575
IPO7	0.469063626	4.266149064	0.647686828	28.10004318	0.13146323
IPO8	0.180197811	2.001447376	0.698873197	5.731785988	0.196167917
IPO9	0.954481243	0.840533624	0.388738822	1.817407302	0.658824694

IPP	0.096372408	1.276860207	0.898677776	1.814189725	0.172622302
IPPK	0.837362445	1.085764291	0.794593706	1.483631305	0.605464348
IQCB1	0.301024094	0.008650688	1.57E-07	476.2975325	0.393731784
IQCC	0.520720568	1.077185478	0.057552384	20.16125941	0.960324809
IQCD	0.674969585	0.063033179	9.78E-08	40630.78944	0.685472417
IQCE	0.894602445	0.893503783	0.6178759	1.29208634	0.549620823
IQCF1	0.609791136	1.057940639	0.438339929	2.553357157	0.900291868
IQCF2	0.718908247	0.797110605	0.036544602	17.386571	0.885354248
IQCG	0.786229959	1.045859183	0.81120873	1.348384689	0.72941797
IQCH	0.490053908	0.945506812	0.825725794	1.082663444	0.417500203
IQGAP1	0.06044704	0.800642087	0.633326535	1.012159946	0.063039561
IQGAP2	0.428883968	0.728754954	0.324085734	1.638713858	0.444076573
IQGAP3	0.462439323	0.689903919	0.214309904	2.220930574	0.533747279
IQSEC1	0.256837191	0.005711779	2.14E-06	15.23928677	0.199406057
IQSEC2	0.308391766	0.826977377	0.384981841	1.776425555	0.626259359
IQSEC3	0.490745598	0.036612686	0.000101289	13.23427671	0.27110142
IRAK1	0.308356383	0.931734619	0.813635117	1.066976316	0.306551026
IRAK1BP1	0.155806873	1.653475722	0.525420411	5.20341788	0.389937741
IRAK2	0.522561197	1.456333704	0.45446163	4.666857916	0.526944026
IRAK3	0.73365258	0.943478661	0.741150134	1.201041385	0.636610949
IRAK4	0.675556338	3.094356843	0.00016458	58178.64957	0.822014434
IREB2	0.13676315	1.215752782	0.010106467	146.2484137	0.936285427
IRF1	0.669318189	0.092826476	0.001110788	7.757333562	0.292479982
IRF2	0.754156089	0.962539575	0.378685199	2.446576829	0.936065022
IRF2BP1	0.185617459	0.698956044	0.394723185	1.237676349	0.21924193
IRF2BP2	0.857101231	0.826414999	0.308265522	2.215498335	0.704734668
IRF3	0.693549181	1.168510768	0.717110538	1.904054317	0.531882666
IRF4	0.98105309	8.204621042	0.226989857	296.5586534	0.250206817
IRF5	0.705259338	1.163355454	0.000412277	3282.733114	0.970225128
IRF6	0.07294191	0.016646531	0.000100774	2.749786166	0.11600447
IRF7	0.076026705	0.150967802	0.022789714	1.000068602	0.050008313
IRF8	0.439047164	0.388557228	0.001539643	98.05955704	0.737633966
IRGC	0.969755648	1.015392248	0.932244472	1.105956053	0.726021474

IRGM	0.473797529	1.608713977	0.80520744	3.214029742	0.17817118
IRGQ	0.052194641	1.338255489	0.617556281	2.900023542	0.460251365
IRS1	0.787499295	1.194910261	0.846458411	1.686805299	0.311384919
IRS2	0.936277013	53.47776885	0.018473913	154805.9587	0.327831704
IRS4	0.905000172	1.196988608	0.323854667	4.424150316	0.787480258
IRX1	0.737099439	1.038924108	0.336254581	3.209958657	0.947102757
IRX2	0.691132934	0.846155623	0.218900297	3.270801131	0.808659339
IRX3	0.76160605	3.790850844	0.000856323	16781.68389	0.75572424
IRX4	0.749820006	1.062773671	0.886427478	1.274202238	0.510748675
IRX5	0.920830775	0.056309697	4.70E-05	67.46675124	0.426349165
IRX6	0.731862229	0.043612829	5.00E-05	38.03338405	0.364545726
ISG20	0.700583469	1.306086032	0.903022899	1.889055888	0.156129973
ISG20L2	0.536787775	0.871123986	0.423865151	1.790326467	0.70737166
ISL1	0.297043857	0.681132626	0.432572563	1.072517524	0.097371185
ISL2	0.853437045	0.218921658	0.00131279	36.50749868	0.560641858
ISLR	0.316734472	1.558896949	0.558577195	4.350624624	0.39652223
ISLR2	0.82732466	0.13852652	0.000142475	134.6871899	0.573335319
ISOC1	0.545123406	0.957944527	0.065468597	14.01676163	0.974963153
ISOC2	0.144553617	0.73362152	0.528256864	1.018823552	0.064505664
ISYNA1	0.678663461	170.910221	0.002856122	10227260.05	0.359620679
ITCH	0.407073657	1.192088657	0.919686541	1.545173603	0.184360097
ITGA1	0.883680324	1.596388767	0.402868879	6.325773042	0.505525318
ITGA10	0.844411612	0.690312058	0.003200015	148.9151346	0.892479927
ITGA11	0.439370452	2.025544222	0.100306412	40.90296243	0.64528925
ITGA2	0.546822182	0.989466882	0.682846269	1.433770315	0.955376559
ITGA2B	0.445653933	0.376606524	0.033236111	4.267420888	0.430433891
ITGA3	0.759859411	0.000638794	9.56E-09	42.67521991	0.194375102
ITGA4	0.308208854	1.64199435	0.389689989	6.918693122	0.499187361
ITGA5	0.294876726	1.403306093	0.469672048	4.192857546	0.544030597
ITGA6	0.742506919	0.856570468	0.69993769	1.048254691	0.132947219
ITGA7	0.762982681	1.008016204	0.251864028	4.03430642	0.990997083
ITGA8	0.791527052	1.049658307	0.779855452	1.412803565	0.749189287
ITGA9	0.142783447	0.498862461	0.18631442	1.335719238	0.166385404

ITGAD	0.140632454	1.19840397	0.919613614	1.5617125	0.18035166
ITGAE	0.6803753	1.185069816	0.58713573	2.391934944	0.635586678
ITGAL	0.108252694	1.002517869	0.434597935	2.312579048	0.995295158
ITGAM	0.789338188	0.900770121	0.476454921	1.702966588	0.747747682
ITGAV	0.740661452	1.011392255	0.780061119	1.311325828	0.931873374
ITGAX	0.18050626	0.430448301	0.182872372	1.013197002	0.053613515
ITGB1	0.731756239	1.046935878	0.434310469	2.523712439	0.918618692
ITGB1BP1	0.682581405	1.12706557	0.516525314	2.459273079	0.763814192
ITGB1BP2	0.915414212	0.911028265	0.170474712	4.868595993	0.913226378
ITGB2	0.427135649	1.370443231	0.561716634	3.343526854	0.488611763
ITGB3	0.357638944	0.083821781	8.54E-06	822.8619822	0.597078383
ITGB3BP	0.578118704	0.976413809	0.741103631	1.286438072	0.865280625
ITGB4	0.260889211	0.087555069	0.004984728	1.537875297	0.095790153
ITGB5	0.438539941	0.238082846	0.054884555	1.032775829	0.055252164
ITGB6	0.77541988	1.018559421	0.413029192	2.511840117	0.968148501
ITGB7	0.790181888	1.083167919	0.897901463	1.306660908	0.403873509
ITGB8	0.645028836	3.56926126	0.058442158	217.9869177	0.544214622
ITGBL1	0.186792905	0.001588904	2.12E-06	1.19078078	0.056357279
ITIH1	0.410359758	0.636808795	0.231883731	1.748830932	0.381277859
ITIH2	0.811895267	1.243332881	0.906322133	1.705659164	0.176954293
ITIH3	0.139972255	1.35420952	0.547023483	3.352476593	0.512076604
ITIH4	0.217838829	0.044091535	0.00075158	2.586633758	0.132964465
ITIH5	0.895151666	0.937741668	0.396984936	2.215095223	0.883471502
ITK	0.550686425	1.425782166	0.479891933	4.236067841	0.523167767
ITLN1	0.520819347	0.85979126	0.438006828	1.687738555	0.660664322
ITLN2	0.320942068	6.220951803	0.057637225	671.4452554	0.444105156
ITM2A	0.633943396	1.124868896	0.558418967	2.265915214	0.74191895
ITM2B	0.487121938	1.775378058	0.101360224	31.09668777	0.694357656
ITM2C	0.390953626	1.274020965	0.802624702	2.022276931	0.304278948
ITPA	0.192589723	1.362306507	0.88730763	2.091584651	0.157541486
ITPK1	0.196080247	0.617397988	0.099585282	3.827676801	0.604425725
ITPKA	0.960736573	0.102838661	0.004125578	2.563468824	0.165670183
ITPKB	0.911372858	0.584559597	0.211834521	1.613098379	0.299879321

ITPKC	0.483201989	0.452745912	3.36E-05	6109.332601	0.87027061
ITPR1	0.479099866	1.049392964	0.795894622	1.383632408	0.732540327
ITPR2	0.524753933	1.160536049	0.836056886	1.610947704	0.373570336
ITPR3	0.117769278	0.806318942	0.371815503	1.748582912	0.585701498
ITSN1	0.917355489	0.288428535	0.000593172	140.2477303	0.693667946
ITSN2	0.771166859	1.064098491	0.432433407	2.618450793	0.892429997
IVD	0.210477015	0.655843898	0.336100337	1.27977027	0.216183835
IVL	0.306279908	1.106883111	0.846656164	1.447093015	0.457707399
IVNS1ABP	0.414953661	0.002887343	1.24E-06	6.709499983	0.139240856
IZUMO1	0.803610367	1.374719246	0.823699262	2.294348307	0.223299032
JAG1	0.333950768	0.792469425	0.246066802	2.552184145	0.696684857
JAG2	0.645810184	0.673809063	0.322309439	1.408642129	0.294027466
JAGN1	0.476302609	0.981369239	0.840835018	1.145391858	0.81149785
JAK1	0.623973453	0.063124221	0.001223782	3.256026818	0.169694057
JAK2	0.640022012	0.904391838	0.622392105	1.314162873	0.598145468
JAK3	0.413317308	0.605150162	2.50E-06	146425.4989	0.936704078
JAKMIP1	0.672662444	3.845604579	0.523527367	28.2481404	0.185544394
JAKMIP2	0.384330836	1.126091978	0.756212426	1.676887471	0.558863353
JAM2	0.111853359	0.868844875	0.49628919	1.52107165	0.622681706
JAM3	0.310980636	2.762662425	0.44567279	17.12535262	0.274953915
JARID2	0.855533384	1.135460389	0.888738005	1.450675326	0.309474993
JAZF1	0.275285618	1.01357614	0.032983104	31.14735951	0.993843455
JDP2	0.862579661	1.251237261	0.714235436	2.191986849	0.433330308
JMJD1C	0.553347407	0.749302162	0.278835382	2.013567023	0.567159468
JMJD4	0.935522083	3.854737814	0.000853318	17413.21246	0.753335019
JMY	0.101868872	0.241706726	0.03235038	1.805918259	0.166381832
JOSD1	0.788396199	1.247978335	0.813475797	1.914562093	0.310331577
JOSD2	0.981417796	1.023906288	0.577444378	1.815558567	0.935566997
JPH1	0.285986016	0.004954628	9.74E-07	25.21326215	0.222913009
JPH2	0.4495431	0.79481954	0.435761984	1.449732021	0.453934101
JPH3	0.467716279	0.916946358	0.258296112	3.255142388	0.893295868
JPH4	0.080170356	0.008056957	1.36E-06	47.75573159	0.276716382
JRK	0.299362304	0.884704952	0.606476938	1.290573148	0.524859194

JRKL	0.291144398	0.604741401	0.15453967	2.366461386	0.469973854
JSRP1	0.098485609	0.695072157	0.356652487	1.354610779	0.285324841
JTB	0.405182535	0.436113544	0.100223692	1.897705218	0.268694
JUN	0.304016302	3.81548184	0.422894517	34.42442757	0.232819248
JUNB	0.700049863	0.802842736	0.362028588	1.780402102	0.588914924
JUND	0.546515716	1.099796666	0.859624028	1.407071773	0.449224023
JUP	0.054614994	1.251722397	0.963279438	1.626536286	0.092952899
KAAG1	0.252209538	1.216516754	0.916657961	1.614465892	0.174681778
KAL1	0.159613488	1.75944814	0.518976513	5.964928433	0.364395275
KALRN	0.80588295	0.90353039	0.403125125	2.025096216	0.805402019
KARS	0.534527267	0.042460396	1.36E-06	1322.754741	0.549544223
KATNA1	0.821142266	2.072104931	0.003843071	1117.236353	0.820408895
KATNAL1	0.281017005	0.702146152	0.402923859	1.223579114	0.212071643
KATNAL2	0.413542766	0.984659308	0.307218124	3.155913919	0.979245672
KATNB1	0.790732987	1.036611163	0.791265012	1.358031362	0.794139503
KAZALD1	0.548317874	0.974903986	0.203287084	4.675347601	0.974651121
KBTBD11	0.521142959	1.461713127	1.37E-05	155612.3923	0.948750983
KBTBD2	0.522832712	0.823418072	0.002291879	295.8346636	0.948398919
KBTBD3	0.4119227	0.297576844	0.005855884	15.12188123	0.545337643
KBTBD4	0.947416683	0.03785555	0.001173712	1.220949614	0.064700135
KBTBD6	0.339013339	1.795165827	0.622560126	5.176400175	0.278867386
KBTBD7	0.667461127	0.20413318	0.006421841	6.488848647	0.36793754
KBTBD8	0.503801393	1.071419918	0.243181004	4.720519374	0.927352895
KCMF1	0.50838575	0.962919951	0.440393818	2.105422	0.924580195
KCNA1	0.14661857	1.859146143	0.611069208	5.656355022	0.274682898
KCNA10	0.094103627	0.632817215	0.356217468	1.124194246	0.118599927
KCNA2	0.184856822	1.522699048	0.775805478	2.988651741	0.221655227
KCNA3	0.217042229	0.616179514	0.152646698	2.487293849	0.49642849
KCNA4	0.793394799	35.39818077	0.002489496	503327.2491	0.464749847
KCNA5	0.384778426	1.111788834	0.132532524	9.326574115	0.922207832
KCNA7	0.712551405	1.337383057	0.909604842	1.966341163	0.1393516
KCNAB1	0.744421057	0.925462314	0.247631464	3.458690102	0.908317656
KCNAB2	0.618337988	0.731215398	0.25013241	2.13757169	0.567342982

KCNAB3	0.341441423	1.192007299	0.90442181	1.571038409	0.212462365
KCNB1	0.46242333	1.06189503	0.920055074	1.225601692	0.411672434
KCNB2	0.519707286	1.507065573	0.576003916	3.943109724	0.403249476
KCNC1	0.987698592	2.112118612	0.048462073	92.05229532	0.697843845
KCNC2	0.326599249	0.748698665	0.161726361	3.466037867	0.711260543
KCNC3	0.934206579	1.907235699	2.73E-05	133056.3775	0.909661937
KCNC4	0.53199819	1.426890684	0.216570966	9.401154099	0.711703069
KCND1	0.19708839	0.395179028	0.014384984	10.85621394	0.582851949
KCND2	0.03430045	0.370183804	0.135996674	1.007642651	0.051767514
KCND3	0.62017016	0.175970543	0.001351891	22.90542745	0.484293142
KCNE2	0.072811366	1.938849279	0.657954483	5.71336867	0.229843124
KCNE3	0.164019007	0.000527429	1.61E-07	1.729666294	0.067653726
KCNE4	0.199623634	1.089692122	0.846303988	1.403076125	0.505397347
KCNF1	0.689665065	9.370052264	0.004264688	20587.17324	0.568734087
KCNG1	0.523549463	0.841345181	0.223160328	3.171987239	0.798620247
KCNG2	0.47027852	4.959037513	0.000398025	61785.24888	0.739289749
KCNG3	0.405415253	1.104572572	0.609279336	2.002497861	0.743171348
KCNG4	0.854705883	159.0135881	0.003236038	7813665.697	0.357725953
KCNH1	0.947742214	0.859923038	0.593803912	1.245306096	0.424418615
KCNH2	0.101608262	0.008689476	4.34E-05	1.737843368	0.079169147
KCNH3	0.730930502	3.224190972	0.298845957	34.78517	0.33470627
KCNH4	0.363712061	0.756642779	0.440006172	1.301136965	0.313344547
KCNH5	0.138546693	0.008343918	1.44E-05	4.835501204	0.14035763
KCNH6	0.93714486	0.891925089	0.659170094	1.20686659	0.458516172
KCNH7	0.187328052	0.698557888	0.438934966	1.11174356	0.130240742
KCNH8	0.632521764	1.084322766	0.873509938	1.346013148	0.462989307
KCNIP1	0.369694512	2.009924999	0.331158757	12.19897833	0.447993428
KCNIP2	0.111740645	2.337231673	0.52772696	10.35128448	0.263509384
KCNIP4	0.780095588	0.008444185	1.30E-06	54.85503092	0.286473685
KCNJ1	0.873943626	1.019660995	0.52195743	1.991941268	0.954555343
KCNJ10	0.07936754	1.628291054	0.849736306	3.120181802	0.141764374
KCNJ11	0.892393135	0.508215027	0.002798694	92.28680874	0.798699913
KCNJ12	0.338633687	1.227018646	0.043793233	34.37916444	0.904235902

KCNJ13	0.854827715	0.899331049	0.374156234	2.161654041	0.812553798
KCNJ14	0.710406921	1.279038238	0.398835013	4.101793375	0.678922581
KCNJ15	0.977937163	3.101451741	0.28952541	33.22334615	0.349531403
KCNJ16	0.903230076	0.99886695	0.332717938	2.998741786	0.998387289
KCNJ2	0.747797258	0.729767122	0.321355717	1.65722912	0.451556937
KCNJ3	0.773904052	0.574865361	0.184123873	1.794825291	0.340564353
KCNJ4	0.732513622	0.98720933	0.742488164	1.312589625	0.929424862
KCNJ5	0.065753285	0.005608998	4.43E-06	7.094409798	0.154931218
KCNJ6	0.467703527	1.465336065	0.379677908	5.655345592	0.579231326
KCNJ8	0.207103929	1.028076296	0.360299563	2.933505839	0.958720404
KCNJ9	0.397597917	23.83994344	0.009398441	60472.03927	0.427795133
KCNK1	0.677348182	0.959220988	0.532572341	1.727661827	0.889701618
KCNK10	0.930481624	1.580318643	0.189902409	13.15100231	0.672071863
KCNK12	0.061441384	1.284640106	0.568911379	2.900803648	0.546688395
KCNK13	0.451203283	0.912176578	0.688589514	1.208363028	0.521704021
KCNK15	0.165914659	1.500515464	0.504464498	4.463241052	0.465602246
KCNK16	0.089768621	1.74615232	1.007526991	3.026269222	0.046956695
KCNK17	0.248289505	0.692929532	0.428745233	1.11989895	0.134225475
KCNK18	0.809040926	0.271530219	0.026325367	2.800669817	0.273527401
KCNK2	0.214766252	0.004469487	3.75E-08	532.896665	0.364289219
KCNK3	0.622376059	1.108893864	0.034011872	36.15342315	0.953636144
KCNK4	0.985486425	3.482649641	0.305700317	39.6756165	0.314793551
KCNK5	0.264684097	0.001678356	1.38E-06	2.048018873	0.078025373
KCNK6	0.089808828	0.460482186	0.156474704	1.355131778	0.159090399
KCNK7	0.621212703	0.981397823	0.750715845	1.282964376	0.890754301
KCNK9	0.953148803	0.949916323	0.714955176	1.262094535	0.723035289
KCNMA1	0.469759495	1.405558162	0.731169986	2.701962313	0.307275372
KCNMB1	0.920574278	0.250106454	0.002038843	30.68075471	0.572231782
KCNMB2	0.582682271	0.57041525	0.076280429	4.265491951	0.584457326
KCNMB3	0.826458497	0.008268049	9.43E-07	72.46493702	0.300539252
KCNMB4	0.084742126	0.045079272	0.000192176	10.5743906	0.265701745
KCNN1	0.604421133	2.599985591	0.036160519	186.9421452	0.661355984
KCNN2	0.505229178	0.93590984	1.40E-05	62520.14192	0.990676489

KCNN3	0.118166354	1.141547148	0.958049599	1.360190424	0.138700773
KCNN4	0.717059193	0.560581349	0.181034058	1.73586922	0.315558012
KCNQ1	0.346019709	0.888933178	0.712288873	1.109384444	0.297600656
KCNQ1DN	0.584260835	0.808667766	0.566208801	1.154951239	0.242889142
KCNQ2	0.423037789	0.840313214	0.625254037	1.129343044	0.248702653
KCNQ3	0.367908028	1.501145629	0.574168654	3.92469736	0.407414139
KCNQ4	0.111740645	2.156645942	0.741191892	6.275192392	0.158432397
KCNQ5	0.534933555	0.795314078	0.00038968	1623.189709	0.953033779
KCNRG	0.705594452	0.84067341	0.376300078	1.878106927	0.672166909
KCNS1	0.165489151	1.146909241	0.990451983	1.32808135	0.066987892
KCNS2	0.731368241	0.969340254	0.740037508	1.269693115	0.821110009
KCNS3	0.419965602	0.001739341	7.59E-08	39.86111646	0.214793727
KCNT1	0.344823003	0.642570878	0.178189498	2.317181083	0.499143773
KCNT2	0.116648091	1.224019267	0.808999183	1.851946449	0.338694205
KCNV1	0.92898925	0.712480104	0.439290216	1.15556386	0.169455012
KCNV2	0.151891277	0.048599915	8.55E-06	276.2491312	0.492974034
KCTD1	0.916976408	0.012948626	4.28E-07	391.6514657	0.408938919
KCTD10	0.751670995	0.25444041	0.017464916	3.7068557	0.316641156
KCTD11	0.567997717	12.2884687	0.523124593	288.6625197	0.119315291
KCTD12	0.071675365	0.810365272	0.544357096	1.206362293	0.300297515
KCTD13	0.103994099	0.061467944	0.003668211	1.030013825	0.052452413
KCTD14	0.986018906	1.878969698	0.027241648	129.6003503	0.770296409
KCTD15	0.2784629	5.393649121	0.229457351	126.783695	0.29549077
KCTD16	0.730039041	3.827146399	0.275103666	53.24192799	0.317718928
KCTD17	0.235309506	0.089986454	0.00109648	7.385048786	0.284241691
KCTD18	0.983821708	0.954924243	0.812297566	1.122593922	0.576274986
KCTD19	0.381363107	1.275472909	0.966738731	1.682803316	0.085298155
KCTD2	0.336298421	0.749569086	0.371037295	1.514278544	0.421722758
KCTD3	0.59606511	2.554195078	0.166072234	39.28358358	0.50127901
KCTD4	0.946223175	0.620828206	0.126057477	3.057554934	0.557855654
KCTD5	0.549580025	1.083975399	0.896550987	1.310580975	0.405114299
KCTD6	0.414417196	0.585137728	0.160223071	2.136934208	0.41741525
KCTD7	0.762374864	1.361238801	0.689539947	2.687257034	0.374151993

KCTD8	0.792702892	0.978649681	0.855406123	1.119649687	0.753321013
KCTD9	0.618512192	0.640612612	0.049470194	8.295591446	0.733247079
KDELC1	0.680642977	0.667403985	0.025320953	17.59128443	0.808599274
KDELC2	0.566107719	0.155004236	0.011109721	2.162638736	0.165633333
KDELR1	0.226575836	1.020908775	0.296509922	3.515075383	0.973830829
KDELR2	0.410992922	0.913285515	0.171804066	4.854893438	0.915255454
KDELR3	0.644520503	2.4871818	0.752743529	8.218035849	0.135127224
KDR	0.4276169	1.446723178	0.877284256	2.385780822	0.147902056
KEAP1	0.061790924	0.578991998	0.210690649	1.591108743	0.289368182
KEL	0.146192417	0.016010587	5.89E-05	4.352529664	0.148263699
KERA	0.858295856	0.582211105	0.139368073	2.432190992	0.458367205
KHDRBS1	0.758414664	675.2503482	0.315203913	1446565.268	0.095928017
KHDRBS2	0.793729138	1.109220063	0.421265397	2.920650865	0.833786295
KHDRBS3	0.781263086	0.959128483	0.074475595	12.35206572	0.974468291
KHK	0.97757877	0.164974909	1.39E-05	1951.347568	0.706475944
KHSRP	0.728550901	0.758131421	0.413503616	1.389983616	0.370636615
KIAA0020	0.522516692	12.09867737	0.165912854	882.2583117	0.254628788
KIAA0040	0.264997876	2.058872048	0.803481176	5.275735435	0.132526462
KIAA0100	0.821259721	0.994074271	0.798437118	1.237647442	0.957610076
KIAA0101	0.898343177	0.967864882	0.720352118	1.300422954	0.828402485
KIAA0125	0.492742763	3.152927866	0.91814155	10.82725657	0.068108537
KIAA0141	0.614473878	0.896600424	0.651578724	1.23376085	0.502761687
KIAA0195	0.374650039	0.78195606	0.400305805	1.527470428	0.471546984
KIAA0196	0.677906277	0.189233854	0.018645294	1.92056246	0.159130079
KIAA0226	0.759963527	0.625804651	0.101084141	3.874311593	0.614325508
KIAA0232	0.646555418	0.033758988	0.001064965	1.070147555	0.054666352
KIAA0319	0.373367129	0.845931526	0.270599298	2.644501124	0.773566474
KIAA0319L	0.408013651	0.714278412	0.369204559	1.381872564	0.317624818
KIAA0355	0.175128954	1.833850403	0.806504637	4.169854885	0.147931204
KIAA0391	0.954805958	9.549287254	0.003338627	27313.28792	0.578418999
KIAA0408	0.479944763	1.00700758	0.78821543	1.286531863	0.955444073
KIAA0513	0.352348479	0.790726515	0.540976935	1.155776488	0.225350942
KIAA0556	0.057338142	2.180224099	0.688063047	6.908345313	0.185307971

KIAA0586	0.253035902	0.024634458	2.77E-05	21.88697815	0.285005702
KIAA0753	0.345370905	1598.445983	0.017633426	144896942	0.205288332
KIAA0895	0.905283377	1.031621816	0.793975914	1.340397802	0.815730306
KIAA0907	0.410800751	1.595398272	0.51427899	4.949250694	0.418684962
KIAA0922	0.282058306	0.68663505	0.252517392	1.867070175	0.461356336
KIAA1024	0.547255817	0.207140492	1.93E-06	22248.37732	0.789957387
KIAA1033	0.351725339	15.87029078	0.001745546	144290.7248	0.552231184
KIAA1107	0.495519808	1.269490521	0.828903451	1.944262845	0.272576875
KIAA1109	0.761796243	0.028424916	0.00011639	6.941999087	0.204352312
KIAA1143	0.229198443	0.049380816	0.000758573	3.214542333	0.157977437
KIAA1161	0.063524434	6.277445419	0.526035896	74.91184777	0.146460464
KIAA1191	0.975101928	0.6314307	0.219141189	1.819396578	0.394487291
KIAA1210	0.414266615	0.008312115	1.34E-06	51.61455611	0.282404208
KIAA1211	0.6406612	2.623387259	0.001441707	4773.618736	0.801174098
KIAA1217	0.357807635	1.02818494	0.781452221	1.352820099	0.842626418
KIAA1244	0.904188556	0.01352183	2.14E-06	85.55951154	0.335215472
KIAA1257	0.483785759	0.477423582	0.132202778	1.724118657	0.259098342
KIAA1279	0.422823466	1.083990107	0.827103635	1.420661815	0.558942123
KIAA1324	0.456472234	61.10832195	0.012122056	308052.2856	0.344409754
KIAA1324L	0.9668731	0.937779335	0.676501203	1.299968243	0.699838615
KIAA1328	0.983066835	1.575862231	0.013351528	185.9968199	0.851786633
KIAA1377	0.713365189	1.083716443	0.760233736	1.544842426	0.656707268
KIAA1407	0.822823524	1.148706819	0.357139535	3.694710969	0.816082276
KIAA1429	0.909794353	1.134411922	0.925555655	1.390397649	0.224446619
KIAA1456	0.96707632	0.954357397	0.275590993	3.304890446	0.941236632
KIAA1462	0.268380934	0.549879777	0.118032845	2.561725664	0.446195744
KIAA1467	0.241890645	1.258044439	0.47695329	3.318303582	0.64272532
KIAA1468	0.300264779	0.890914959	0.178090772	4.456881481	0.888171996
KIAA1522	0.424762172	0.843675817	0.510617499	1.393976674	0.507018757
KIAA1524	0.90397377	1.786996051	0.696941439	4.581955824	0.226888357
KIAA1549	0.124653781	1.770000955	0.950109188	3.297414045	0.072060045
KIAA1586	0.293111985	0.298918465	0.014327748	6.236307749	0.435932939
KIAA1598	0.156087784	0.372619018	0.098534379	1.409101412	0.145772444

KIAA1614	0.948463077	0.912060452	0.306180577	2.716874719	0.868720388
KIAA1644	0.822470892	26.11663951	0.22680493	3007.337011	0.177889131
KIAA1671	0.629587087	0.929565579	0.233491294	3.700746823	0.917474853
KIAA1683	0.325383556	0.456917406	0.175137702	1.192053529	0.109400053
KIAA1715	0.287839233	0.010049725	3.81E-09	26477.49395	0.541958274
KIAA1755	0.595888007	0.660883078	0.239316428	1.825058344	0.424200121
KIAA1841	0.780229289	0.832534964	0.390787309	1.773636066	0.634811266
KIAA1875	0.270570712	0.784657037	0.294747146	2.088863871	0.627364473
KIAA1919	0.449714422	1.112509361	0.368753069	3.356384488	0.849904589
KIAA1958	0.19126633	0.000421262	3.78E-08	4.695055107	0.10211296
KIAA2013	0.615385283	1.386497683	0.765405815	2.511577241	0.281028755
KIAA2018	0.868024448	1.292486483	0.675389336	2.473419731	0.438464795
KIAA2022	0.34246563	0.766923718	0.189619805	3.101848938	0.709737626
KIAA2026	0.748203275	0.000136814	4.06E-09	4.610388974	0.094398703
KIDINS220	0.194963125	0.53911447	0.152072057	1.911228259	0.338660761
KIF11	0.457006348	0.65065749	0.150147155	2.819601668	0.565671341
KIF12	0.0908911	0.758315095	0.455169677	1.263356971	0.288092472
KIF13A	0.386042528	3.103992327	0.060724965	158.6623953	0.572545971
KIF13B	0.170947699	0.149951711	0.013228035	1.699837923	0.125597621
KIF14	0.550096225	0.268922248	0.002402603	30.10034739	0.585338645
KIF15	0.128878649	1.549527663	0.86119325	2.788033905	0.143924431
KIF17	0.178628772	0.493413087	0.050101767	4.859239323	0.544968527
KIF18A	0.977913111	0.660720362	0.234562185	1.861132889	0.432848035
KIF19	0.254483817	1.230666759	0.314109352	4.821698759	0.765779978
KIF1A	0.714283383	1.11548211	0.46061624	2.701381824	0.808644708
KIF1B	0.867408029	0.891721827	0.561451917	1.416270554	0.62730947
KIF1C	0.553547665	1.445832644	0.052448838	39.8565938	0.827525261
KIF20A	0.328825597	0.857096823	0.462833656	1.587211634	0.623783346
KIF21A	0.653305123	0.323961104	0.049106139	2.137223535	0.241623696
KIF21B	0.591977449	0.757266342	0.172102817	3.33203327	0.713018368
KIF22	0.447699541	4.729262973	0.276790315	80.80459136	0.283290989
KIF23	0.993844626	0.744833098	0.295175558	1.879479274	0.532749819
KIF24	0.440392036	0.692405934	0.233421623	2.053905594	0.507594437

KIF25	0.383548085	154.4523611	3.326596492	7171.152827	0.010059485
KIF27	0.591557151	0.682005908	0.297428903	1.563842836	0.366049585
KIF2B	0.916476579	0.70574357	0.181752497	2.740396942	0.614611336
KIF2C	0.910564787	0.17327929	0.000683452	43.93244303	0.534840196
KIF3A	0.707921889	0.83976202	0.285734372	2.468027368	0.750864974
KIF3B	0.839235136	0.588342635	0.058316936	5.935618022	0.652861116
KIF3C	0.289780332	28.1128678	0.005142113	153698.1682	0.447398267
KIF4A	0.91193233	4.055750005	0.131713646	124.8853752	0.42330403
KIF5A	0.609318803	0.911686771	0.432088257	1.923618049	0.808237879
KIF5B	0.215900762	1.866234293	0.570892447	6.100677034	0.301880118
KIF5C	0.405105335	0.001604876	6.99E-07	3.683764228	0.103161382
KIF6	0.509862527	1.169586467	0.79883809	1.712402704	0.420630799
KIF7	0.308301727	194.0562216	0.011776457	3197720.374	0.28760162
KIF9	0.283852353	0.683207763	0.263492143	1.771486775	0.433234363
KIFAP3	0.483653699	1.011325721	0.388215016	2.634570205	0.981607195
KIFC1	0.21339042	1.272954502	0.595194179	2.72249498	0.533795379
KIFC2	0.516879055	0.965349252	0.347011207	2.685501679	0.946139083
KIFC3	0.948754655	1.460224975	0.549892476	3.877588927	0.44738296
KIN	0.653192181	0.812084387	0.000354286	1861.438734	0.957948787
KIR2DL1	0.188615802	0.834911246	0.397753152	1.752536181	0.633416429
KIR2DL3	0.885207133	1.025001614	0.378086871	2.778801352	0.961294246
KIR2DL4	0.533443415	902.505369	0.313061888	2601772.911	0.094083055
KIR2DS4	0.672428128	0.92522766	0.636852112	1.344183693	0.683410242
KIR3DL1	0.606981362	0.882016074	9.71E-05	8009.510222	0.978460822
KIR3DL2	0.13833573	0.690174789	0.314913582	1.512609381	0.354318664
KIR3DL3	0.739213669	1.45455151	0.148014282	14.2940267	0.747924358
KIRREL	0.215532382	1.234867631	0.387574076	3.934468685	0.721230378
KIRREL2	0.439397903	1.163464516	0.815957787	1.658970232	0.402941885
KIRREL3	0.050613236	0.5397578	0.25429317	1.14567954	0.108316599
KISS1	0.343404684	0.573122129	0.2602368	1.262192642	0.166999656
KISS1R	0.297330489	0.279585578	0.019091147	4.094468297	0.352048635
KIT	0.841657035	0.387670931	8.90E-06	16889.03909	0.861969381
KITLG	0.424512091	0.43644606	0.036902336	5.161872719	0.510675761

KL	0.140527746	191.7581319	0.067796689	542374.2946	0.194884418
KLB	0.1331733	0.222074791	0.044573438	1.106426059	0.066279436
KLC2	0.354270308	0.134510541	1.77E-06	10215.05945	0.72642533
KLC3	0.587988566	0.27225927	0.002090377	35.46016969	0.600515251
KLC4	0.918695731	1.024806602	0.019164891	54.79961026	0.990370149
KLF1	0.307293197	1.55548625	0.772301006	3.132894372	0.216203872
KLF10	0.618792484	0.697555773	0.000310069	1569.278985	0.927128358
KLF11	0.437667483	1.577303818	0.031953501	77.85961731	0.818814264
KLF12	0.102528925	101.7312472	0.109922411	94150.47011	0.184713089
KLF13	0.522586221	0.0431828	9.12E-05	20.43723746	0.317377394
KLF14	0.48687217	0.501554133	0.120850236	2.081556115	0.34194827
KLF15	0.955857581	1.041960362	0.595377304	1.823518279	0.885541988
KLF16	0.811716927	0.58398557	0.004437768	76.84925226	0.828955331
KLF17	0.322593997	0.796601282	0.312437445	2.031042091	0.633933136
KLF2	0.775027559	1.16025134	0.762754918	1.76489609	0.48735244
KLF3	0.537632561	20.95087528	5.83E-05	7535230.747	0.641155995
KLF4	0.131392633	1.17560182	0.742897444	1.860337051	0.489661478
KLF5	0.805780697	0.505434405	0.193791179	1.318243377	0.162997837
KLF6	0.423672847	1.447541849	0.217368369	9.639753069	0.702209935
KLF7	0.394676327	1.201186742	0.85739029	1.682838733	0.28661727
KLF8	0.483035944	0.533065707	0.126545577	2.245507544	0.39120206
KLF9	0.972031353	0.922462586	0.588942149	1.44485706	0.724442798
KLHDC1	0.967998242	4.896305194	0.45801703	52.34260521	0.188836835
KLHDC2	0.747847654	2.858011625	0.140667709	58.06755863	0.494319861
KLHDC3	0.534362486	0.952874554	0.620126313	1.464169308	0.825674991
KLHDC4	0.425149181	0.928586627	0.726501181	1.186884683	0.55405217
KLHDC7A	0.764521361	0.804909763	0.316849634	2.04475454	0.648211597
KLHDC7B	0.131038452	0.398987378	0.122047502	1.304335817	0.128427121
KLHDC8A	0.090270966	0.446485056	0.152516423	1.307065173	0.14119922
KLHDC8B	0.288105528	5.618440364	0.024815919	1272.041227	0.532691332
KLHL1	0.665370359	1.026460084	0.725286869	1.452694581	0.882830087
KLHL10	0.536583503	1.184729067	0.860330203	1.631446805	0.299080616
KLHL11	0.359402707	0.774113833	0.378409158	1.583609207	0.483227819

KLHL12	0.66776896	1.4675185	0.007289333	295.4468629	0.887304703
KLHL13	0.359644957	0.500438239	0.162092105	1.545037809	0.228749473
KLHL14	0.166412108	1.118554729	0.804456793	1.555291338	0.505297126
KLHL15	0.274777125	0.031645805	0.000448319	2.233805913	0.111853489
KLHL17	0.287368639	1.053836418	0.009576792	115.9648446	0.982557169
KLHL18	0.51972513	0.130855133	9.81E-06	1746.182806	0.674763013
KLHL2	0.982741235	1.225968293	0.015856668	94.78651496	0.926826152
KLHL20	0.381399925	1.051754151	0.319599476	3.46116586	0.933829203
KLHL21	0.4206164	1.001056736	0.906328925	1.10568532	0.983386264
KLHL22	0.159697161	0.927087306	0.41368481	2.077646681	0.854104391
KLHL23	0.215265946	1.595020889	0.939007382	2.709341464	0.084139072
KLHL24	0.781823683	1.033065662	0.890832387	1.198008378	0.666884742
KLHL25	0.293059314	1.35600622	0.879265349	2.091237727	0.168254432
KLHL26	0.220459398	0.224142188	0.005700927	8.812552249	0.424696912
KLHL3	0.729241049	0.654788682	0.032074015	13.3674632	0.783199269
KLHL4	0.640558503	1.369923395	0.592029777	3.169925201	0.462138047
KLHL5	0.284682784	87.78267427	0.061524351	125247.9342	0.227225072
KLHL6	0.207958108	0.415047496	0.122618804	1.404877699	0.157503905
KLHL7	0.246435579	0.983323297	0.357586142	2.704032942	0.974005836
KLHL8	0.108561264	0.660367794	0.140824998	3.096649251	0.598669413
KLHL9	0.708843738	1.285829215	0.98971881	1.670531824	0.059757681
KLK1	0.111003192	0.63807264	0.403420871	1.009210784	0.05476094
KLK10	0.090082374	0.517989644	0.135826514	1.975411604	0.335466964
KLK11	0.410542308	1.179303429	0.000183662	7572.351717	0.970589264
KLK12	0.691322098	1.047243928	0.424230375	2.585198771	0.920246299
KLK13	0.647400234	1.398151757	0.786289153	2.486144348	0.253764848
KLK14	0.927312096	1.267441744	0.34299593	4.683462498	0.722294869
KLK15	0.156542848	0.136947261	0.010826111	1.732344359	0.12464343
KLK2	0.374442988	1.582203217	0.238971006	10.47560983	0.634256338
KLK3	0.235696556	0.282023926	0.062565276	1.271272176	0.099445002
KLK4	0.99914254	8.874999459	0.00333894	23590.00798	0.587363076
KLK5	0.460818766	2.561292028	0.0001644	39903.97416	0.848565448
KLK6	0.817684378	1.633231432	0.265706683	10.03905842	0.596477801

KLK7	0.767269788	0.753046411	0.077246029	7.34120457	0.807135102
KLK8	0.204739453	1.280861046	0.514731905	3.187300035	0.594603287
KLK9	0.365606787	0.101379066	0.004718101	2.178358551	0.14360615
KLKB1	0.807195939	1.125466278	0.965518056	1.311911606	0.130714213
KLRB1	0.097665354	2.164662854	0.97166481	4.822409152	0.058807428
KLRC1	0.706825513	0.187188681	7.52E-05	465.7274426	0.674475157
KLRC2	0.811692011	1.121596925	0.516787504	2.434230031	0.771620379
KLRC3	0.10462927	0.743625423	0.441108326	1.25361218	0.266271822
KLRC4	0.787070404	16.26896283	0.02815473	9400.876812	0.389975408
KLRD1	0.108818228	0.148158503	0.004050796	5.418920055	0.298449161
KLRF1	0.382266553	23.82363266	0.00011579	4901671.651	0.611491455
KLRG1	0.36474254	0.145607863	0.00138976	15.2556242	0.416880102
KLRK1	0.246771533	61.57725105	0.668444847	5672.506661	0.074193123
KMO	0.445163494	0.708767687	0.267156804	1.880362495	0.489262717
KNDC1	0.768816348	0.110998233	0.000636607	19.35354934	0.403833288
KNG1	0.937897076	0.143816982	0.00087079	23.75236573	0.456727394
KNTC1	0.099052287	16.22601017	0.000112194	2346674.712	0.6457582
KPNA1	0.847657993	1.356281309	0.852096299	2.158792369	0.198776211
KPNA2	0.835391812	1.014614557	0.338755331	3.038897419	0.979319085
KPNA3	0.340179423	0.538255046	0.19276876	1.502932806	0.23708205
KPNA4	0.449549926	0.949532118	0.205299688	4.391683463	0.947159678
KPNA5	0.517024371	0.756068205	0.351526459	1.626162455	0.474228269
KPNA6	0.512015408	0.883326614	0.52456908	1.487441668	0.640786058
KPNB1	0.522464277	1.548654894	0.404732565	5.925720301	0.522930435
KPTN	0.11203771	0.754603956	0.234371558	2.429591436	0.63695783
KRAS	0.482969983	1.114519789	0.353518456	3.513690272	0.853173424
KREMEN2	0.186755248	1.576354489	0.367423859	6.763016105	0.540210659
KRIT1	0.247203062	1.110414714	0.853221954	1.445134917	0.435909151
KRT1	0.546420884	46.68530498	0.500347071	4356.01171	0.096762956
KRT10	0.388881498	0.845977988	0.462653664	1.546899573	0.586994103
KRT12	0.069355422	0.184992842	0.000877883	38.9828093	0.53649248
KRT13	0.443776047	0.576433671	0.165096746	2.012612514	0.387829398
KRT14	0.187579339	5.273598157	0.769111554	36.15969282	0.090510911

KRT15	0.231299633	3.642164378	0.6457891	20.54132123	0.143053283
KRT16	0.706899131	1.138558437	0.494769554	2.620038573	0.760242134
KRT17	0.913084304	1.085455375	0.799444799	1.473789526	0.599238582
KRT18	0.359239959	0.973978929	0.827134065	1.146893827	0.751844528
KRT19	0.106403887	1.24383651	0.450532665	3.434000203	0.673662247
KRT20	0.147899833	2.374975788	0.811752562	6.948558286	0.114290512
KRT23	0.763415708	0.983889291	0.785713838	1.23204924	0.887449497
KRT24	0.684676479	0.890461819	0.568547327	1.39464599	0.612284779
KRT3	0.465524883	0.701674015	0.389965365	1.262538851	0.237159313
KRT4	0.506491178	0.935296199	0.759767567	1.151377103	0.528191132
KRT5	0.962172546	8.810166191	0.000529689	146536.9002	0.660810532
KRT6A	0.130260691	0.71245838	0.309183423	1.641734018	0.426029448
KRT6B	0.890042425	0.680003241	0.005249422	88.08672272	0.876503908
KRT6C	0.082610029	1.73539642	0.913795843	3.295704129	0.092087742
KRT7	0.517867236	0.590560514	0.019446518	17.93440484	0.762332273
KRT8	0.493903387	0.909376591	0.680434323	1.215349897	0.520893949
KRT9	0.17905279	0.515310001	0.068175106	3.895034622	0.520596055
KRTAP10-1	0.860730712	0.050257499	4.75E-07	5322.50167	0.612438636
KRTAP10-10	0.667491307	0.894756748	0.618820289	1.293735278	0.554463955
KRTAP10-11	0.48848492	1.25630327	0.244881961	6.445137499	0.784470005
KRTAP10-12	0.450154509	0.058039682	0.000695165	4.845766122	0.20733329
KRTAP10-2	0.98032325	0.795133591	0.258213485	2.448506624	0.689534516
KRTAP10-4	0.265626591	0.578349046	0.278443048	1.2012784	0.142038315
KRTAP10-5	0.141509449	1.49599005	0.514046744	4.353662884	0.459890533
KRTAP10-6	0.644036425	2.275589355	0.483408175	10.71207973	0.298201761
KRTAP10-7	0.665397486	0.832248188	0.036929113	18.75585391	0.90802338
KRTAP10-8	0.509863821	2.219428985	0.884188904	5.571055005	0.089537868
KRTAP10-9	0.47331452	7.614017253	0.185860826	311.9175783	0.283884208
KRTAP1-1	0.222106979	0.681869183	0.001028539	452.0448499	0.908032265
KRTAP11-1	0.819134392	0.141589345	0.001405972	14.25885219	0.406139586
KRTAP12-1	0.719860167	0.096799592	0.000354537	26.42927686	0.414569965
KRTAP12-2	0.982311682	0.424012925	0.085772175	2.096098886	0.292666576
KRTAP12-3	0.493183889	0.555620883	0.235284788	1.31208893	0.180108333

KRTAP12-4	0.52626417	0.569172021	0.020915193	15.48906559	0.738118311
KRTAP1-3	0.445179093	0.622965099	0.15281711	2.539542307	0.509200028
KRTAP13-1	0.29864581	0.044499699	0.00019223	10.30130946	0.262553066
KRTAP13-2	0.993826808	1.130084376	0.789817788	1.616943448	0.503455609
KRTAP13-3	0.355846828	0.548917188	0.117654853	2.560966014	0.445295053
KRTAP13-4	0.974722944	0.897198911	0.179403641	4.486898271	0.894915511
KRTAP1-5	0.252997324	11.87137218	0.171357121	822.4314029	0.252547461
KRTAP15-1	0.53542873	0.875031502	0.599664495	1.276847529	0.488693766
KRTAP17-1	0.840355137	3.878542592	0.000882072	17054.25989	0.751475613
KRTAP19-1	0.441751345	1.119367143	0.873012569	1.435240276	0.373928091
KRTAP19-3	0.375877356	0.573153907	0.220014111	1.4931106	0.254542873
KRTAP19-4	0.254767373	914.961878	0.390322777	2144776.806	0.0850083
KRTAP19-5	0.47260377	3283.206018	0.036552752	294900961.1	0.164123287
KRTAP19-6	0.309866258	0.651087949	0.017087194	24.80896021	0.817287411
KRTAP19-7	0.248890371	3.403831202	0.679743383	17.04476592	0.136149146
KRTAP20-1	0.130622895	0.94358212	0.083365803	10.68000532	0.962586847
KRTAP20-2	0.811045308	6.345700247	6.56E-06	6137808.108	0.792726273
KRTAP2-1	0.731979385	1.058130245	0.714108007	1.567885537	0.778225525
KRTAP21-1	0.51792239	0.214879257	1.38E-09	33511568.27	0.873073883
KRTAP21-2	0.799348601	2.006855325	0.609895458	6.603538762	0.251683285
KRTAP2-2	0.350349241	1.419482571	0.678475339	2.969792203	0.352346587
KRTAP22-1	0.250520498	0.087865873	0.000187721	41.12703764	0.438209945
KRTAP23-1	0.897435691	0.166180356	6.60E-05	418.2806933	0.653296551
KRTAP2-4	0.698610334	4.513275685	0.080996217	251.4889959	0.462531169
KRTAP26-1	0.190919077	3.018566098	0.020850359	437.0064416	0.663396764
KRTAP3-1	0.331624692	1.348968964	0.333376305	5.458448118	0.674688932
KRTAP3-3	0.174254491	15.79135559	0.173724687	1435.414365	0.230419169
KRTAP4-12	0.274957446	0.042061163	7.07E-05	25.01869301	0.330971202
KRTAP4-2	0.813844405	0.950711576	0.143266339	6.308896495	0.958252829
KRTAP4-3	0.186767592	1.180771768	0.321331206	4.338893767	0.802396597
KRTAP4-4	0.253603506	2.087371672	0.684942074	6.361297783	0.195538389
KRTAP4-5	0.208229498	1.399035148	0.908569466	2.154264939	0.127357103
KRTAP4-7	0.933544703	0.381037881	1.10E-05	13206.22921	0.856439918

KRTAP5-1	0.071706843	0.581512429	0.306021337	1.105010217	0.097902812
KRTAP5-10	0.378705919	0.907435596	0.446999096	1.842149948	0.788030974
KRTAP5-11	0.702175379	0.024420338	5.04E-06	118.3826904	0.391228412
KRTAP5-2	0.95971725	1.038167755	0.409858361	2.629670129	0.937039088
KRTAP5-3	0.851504774	1.059995592	0.873190515	1.286764613	0.555828123
KRTAP5-4	0.136872835	1.348054579	0.010520693	172.7311326	0.90399366
KRTAP5-6	0.96035385	2.345842621	0.247049616	22.27478713	0.457804405
KRTAP5-7	0.35076691	0.865831343	0.021526675	34.82488199	0.939077065
KRTAP5-9	0.383530581	0.485312633	0.159251362	1.478972287	0.203507753
KRTAP6-1	0.512136921	12.77129846	0.000608642	267983.6176	0.615895318
KRTAP6-2	0.972234186	0.659861112	0.086714533	5.021265406	0.688051844
KRTAP6-3	0.971036593	0.692425463	0.326745962	1.467357147	0.337446029
KRTAP7-1	0.513175666	0.966410049	0.370541009	2.520499374	0.944307818
KRTAP8-1	0.895073426	1.119497979	0.480530906	2.608106384	0.79363461
KRTAP9-2	0.79026895	1.229092084	0.728967411	2.072338665	0.438982983
KRTAP9-3	0.53036489	0.886779994	0.26152965	3.006843614	0.847059311
KRTAP9-4	0.519492398	1.064522035	0.798423846	1.419305257	0.670074399
KRTAP9-8	0.522268719	0.594934772	0.193835601	1.826018446	0.364091497
KRTCAP2	0.353379155	0.257930422	0.00049636	134.0320124	0.671035666
KRTCAP3	0.675817592	0.891690468	0.667064561	1.191956426	0.438842958
KSR1	0.475896024	1.652146479	0.040209931	67.88342945	0.791137036
KSR2	0.404732501	1.302235819	0.737021214	2.300908165	0.363192165
KTI12	0.398059032	0.04667107	1.64E-06	1326.945547	0.558073412
KTN1	0.961174392	0.10964784	0.00357136	3.366406406	0.205799098
KY	0.37435798	10.89417197	0.008200128	14473.30774	0.515140087
KYNU	0.661771753	1.572594827	0.313915139	7.878098829	0.581859383
L1CAM	0.784764509	2.371279479	0.429017678	13.10660761	0.322259577
L2HGDH	0.787016712	1.134249775	0.001295833	992.8154426	0.970927514
L3MBTL2	0.296411688	1.707182987	0.973254165	2.994565917	0.062123856
L3MBTL3	0.786777523	4.07763664	0.03549332	468.4577416	0.561447688
L3MBTL4	0.52852184	1.041320287	0.672694867	1.611946208	0.855883796
LACE1	0.642879525	1.624461228	0.203338894	12.97771532	0.647236802
LACRT	0.294702125	0.018251327	0.000234616	1.419811709	0.0715177

LACTB	0.62783907	0.007134215	3.16E-06	16.11272392	0.209661034
LACTB2	0.752505822	0.17378112	0.003632041	8.314850926	0.375226689
LAD1	0.953241926	1.324273642	1.39E-05	126269.9307	0.961705919
LAG3	0.873529503	0.988033561	0.3786051	2.578439429	0.98037538
LAIR1	0.474871581	1.517786598	0.113479986	20.30028582	0.752502351
LAIR2	0.815912033	79.36427046	0.709031101	8883.513596	0.06919942
LALBA	0.138374311	30.58160416	0.550657304	1698.396635	0.09514618
LAMA1	0.604686395	0.336674987	0.078105334	1.451245918	0.144188026
LAMA2	0.455069968	0.448610132	0.01528891	13.16320493	0.641960723
LAMA3	0.274823117	1.078075319	0.858593504	1.353663157	0.517450797
LAMA4	0.205901022	1.138101007	0.45689131	2.834971631	0.781163269
LAMA5	0.809633861	1.265783065	0.372668269	4.299284115	0.705586481
LAMB1	0.883615366	0.06635165	0.000755676	5.825968008	0.234786639
LAMB2	0.862273793	1.189588256	0.008568122	165.1610722	0.945011356
LAMB3	0.423932172	5.729506071	0.001621276	20247.77818	0.675388875
LAMB4	0.976962909	0.63655538	0.144933978	2.795774732	0.549674097
LAMC1	0.76531412	1.489501525	0.452034901	4.90806084	0.512530106
LAMC2	0.804420152	0.877566062	0.440336256	1.748941138	0.710496461
LAMC3	0.209926283	0.808164653	0.331042725	1.972948071	0.639982445
LAMP1	0.112540486	0.483669715	0.18960274	1.233823905	0.128460131
LAMP2	0.008224709	0.567353305	0.331883098	0.969889018	0.038291613
LAMP3	0.268064178	0.516285127	0.121055364	2.201887825	0.371669881
LANCL1	0.108993933	0.076180327	8.24E-06	703.9683455	0.580520716
LANCL2	0.898252634	1.16305295	0.067234417	20.11904369	0.91728485
LANCL3	0.75753893	0.800046292	0.262139107	2.44173438	0.695158504
LAP3	0.62003335	0.311393914	0.000302471	320.5796495	0.741669001
LAPTM4A	0.554969276	1.493890242	0.510701639	4.369886221	0.463598532
LAPTM4B	0.541859619	9.64E-05	7.89E-11	117.6604146	0.195941357
LAPTM5	0.330961424	0.236923104	7.16E-06	7835.783416	0.786226097
LARGE	0.185163479	1.014264097	0.814083327	1.263668748	0.899523965
LARP1	0.214613906	11.65219036	0.212041067	640.3171888	0.229663078
LARP4	0.930925819	0.512696933	0.175638564	1.496585592	0.221595287
LARP6	0.519441572	7.878074322	0.085654096	724.5894595	0.370932836

LARS	0.271584341	1.887172924	0.556427092	6.400518053	0.30811356
LARS2	0.232944292	31.41369971	0.00394595	250084.3758	0.451932064
LAS1L	0.063058888	1.839197055	0.988805771	3.420940599	0.05430315
LASP1	0.746707053	1.730371983	0.499773114	5.991092984	0.386842729
LAT	0.356601589	0.095747401	6.85E-08	133808.0557	0.745216303
LAT2	0.65798275	0.955960728	0.289435018	3.15739581	0.941103912
LATS1	0.948953258	1.036666185	0.824631178	1.303221133	0.757753446
LATS2	0.994769729	2.256300925	0.727162745	7.001037795	0.158986757
LAX1	0.767706702	0.959131362	0.42640999	2.157390752	0.919638029
LAYN	0.585163899	1.118136802	0.847984202	1.474355189	0.428732385
LBH	0.086391498	1755.508611	0.042199803	73029024.58	0.168617858
LBP	0.702895745	0.897434666	0.707310172	1.138664495	0.372982974
LBR	0.305193748	0.735833698	0.027632307	19.59486152	0.854651757
LBX1	0.139631748	1.214922045	0.931073331	1.585305396	0.151591242
LBX2	0.154329947	0.642697745	0.27113728	1.523436361	0.315400484
LCAT	0.21580885	0.432454692	0.089471311	2.090246125	0.297040741
LCE1A	0.651621993	1.189157581	0.560567177	2.522616041	0.651626292
LCE1B	0.774606417	5.872167829	0.25073894	137.5229352	0.271242117
LCE1C	0.471872684	1.285219544	0.625466023	2.640893698	0.494672996
LCE1D	0.357654983	1.078355068	0.456390367	2.54792769	0.86347382
LCE1E	0.403133451	11.20139136	0.030040227	4176.771716	0.423872975
LCE1F	0.614328029	0.712811716	0.39094853	1.299660962	0.269294172
LCE2A	0.41350091	0.069818856	0.00071952	6.774891986	0.254145421
LCE2B	0.222764202	0.768854117	0.351148841	1.68343615	0.510935292
LCE2C	0.290788771	0.672654992	0.260925327	1.734077496	0.411835968
LCE2D	0.389892637	0.914793263	0.398618519	2.099367379	0.833570629
LCE3A	0.054964205	0.007123859	4.22E-05	1.203809962	0.058879443
LCE3B	0.564778854	0.800399542	0.424546927	1.508995558	0.491331795
LCE3C	0.568900421	0.849511837	0.40292718	1.791068953	0.66825155
LCE3D	0.731684801	0.66927352	0.16814869	2.663874721	0.568833964
LCE3E	0.437978964	2.262519466	0.581126112	8.80874948	0.239074319
LCE4A	0.266926617	1.803913893	0.698919532	4.655908422	0.222656763
LCE5A	0.22893523	0.005392077	9.95E-06	2.922299269	0.103930715

LCK	0.152984309	0.174268532	0.002613952	11.61824152	0.414856576
LCMT1	0.561651506	0.000540118	1.47E-07	1.990064333	0.072539619
LCMT2	0.096593537	0.760607886	0.219592293	2.63453853	0.665960173
LCN1	0.949116109	1.006840979	0.801066575	1.265473792	0.95339347
LCN10	0.806261987	0.430085329	0.073884109	2.503561223	0.347810373
LCN12	0.679079731	1.444948521	0.713014462	2.928238263	0.307086078
LCN2	0.453612213	23.59819382	8.75E-05	6367312.016	0.620286971
LCN6	0.920025577	20.81502565	0.011699163	37033.87075	0.426605497
LCN8	0.381330921	1.265992429	0.453006222	3.538001799	0.652849124
LCN9	0.26775789	1.167647214	0.74845798	1.82161197	0.494571184
LCP1	0.07224585	1.071584395	0.673085832	1.706012906	0.770742893
LCP2	0.16947033	68.08292025	0.411357237	11268.26908	0.105406465
LCT	0.291086393	2.055843987	0.496574259	8.51130404	0.320108064
LCTL	0.741334939	0.830420498	0.423149631	1.629679319	0.589058822
LDB1	0.151112282	138.637151	0.019527696	984256.4092	0.275693961
LDB2	0.585271455	1.15671978	0.864212327	1.548231385	0.327673741
LDB3	0.233400338	295663.976	0.298591294	2.92765E+11	0.073716338
LDHA	0.34831248	0.969093241	0.00106782	879.4940966	0.99279157
LDHAL6A	0.296751041	1.234043259	0.716971335	2.124021829	0.447826272
LDHAL6B	0.847354987	0.820106593	0.090682605	7.416800906	0.859888024
LDHB	0.579573009	1.145168504	0.136018375	9.641424568	0.900760631
LDHC	0.413887354	0.011651787	5.29E-05	2.565305163	0.105732791
LDHD	0.260799822	0.41085059	0.067975792	2.483210589	0.332508667
LDLR	0.486232049	0.759825702	0.28039502	2.059006243	0.589186338
LDLRAD1	0.452159543	1.661990347	0.390806197	7.067983919	0.49155198
LDLRAD2	0.11189197	4.610811726	0.414615292	51.27544788	0.213643321
LDLRAD3	0.107269854	1.274016753	0.727238697	2.231892627	0.397231984
LDLRAP1	0.207364279	1.588140033	0.523035346	4.822214757	0.414344975
LDOC1	0.815117026	0.145819597	2.80E-06	7605.79984	0.7282764
LDOC1L	0.208843175	1.517634241	0.642963536	3.582183998	0.341092457
LECT1	0.80933199	0.931601204	0.501446979	1.730752879	0.822610114
LECT2	0.704769306	0.498758404	0.068622263	3.625061838	0.491844888
LEF1	0.949846394	0.645941972	0.250223383	1.66747418	0.36639802

LEFTY1	0.644063108	0.494359568	0.062847195	3.888660167	0.503208284
LEFTY2	0.428953028	1.56274916	0.000872185	2800.076641	0.90701055
LELP1	0.516359262	4.204326377	0.16725116	105.6875196	0.382687675
LEMD1	0.321139795	0.736796059	0.23397438	2.320204605	0.601747072
LEMD2	0.118585244	0.511836776	0.215469435	1.215842448	0.129209305
LEMD3	0.845360978	1.92135287	0.344000239	10.73137873	0.456831315
LENEP	0.819352317	0.992404447	0.84627714	1.163763665	0.92525301
LENG1	0.915163225	1.147761226	0.834850674	1.577953847	0.396127721
LENG8	0.623058433	0.320764369	0.086940368	1.183452316	0.087806064
LENG9	0.435583118	0.982001476	0.476846419	2.022300808	0.960698374
LEO1	0.78602184	0.932058339	0.597495148	1.453957828	0.756455699
LEP	0.437301144	2.158462267	0.879541226	5.29703352	0.093007812
LEPR	0.386212132	79.50011725	0.021443276	294743.6124	0.29667516
LEPROT	0.514123653	0.058237381	3.12E-06	1087.425458	0.57097017
LEPROTL1	0.622926409	0.897694441	0.714942281	1.127161355	0.352743824
LETM1	0.389518611	0.009145172	2.32E-07	360.4903697	0.384570401
LETM2	0.097665354	1.534667257	0.928665106	2.536117244	0.094681833
LETMD1	0.238921016	0.540082518	0.000516415	564.8350771	0.862130374
LFNG	0.539463458	239.7676801	0.000847047	67869371.3	0.39225229
LGALS1	0.891282094	0.24649234	0.000165104	368.0017222	0.707244383
LGALS12	0.195689123	1.083193108	0.902345341	1.300286328	0.391209345
LGALS13	0.195926304	0.579963266	0.241559304	1.392442283	0.222796991
LGALS14	0.933680962	2.305369911	0.76802409	6.920004851	0.136398629
LGALS2	0.40969519	0.9728513	0.589609761	1.605196716	0.914212839
LGALS3	0.936972803	1.105447646	0.746876865	1.636165955	0.61629513
LGALS3BP	0.085332986	0.000658927	2.19E-08	19.82457979	0.163849524
LGALS4	0.150798074	0.935640602	0.238992359	3.662976253	0.923890943
LGALS7	0.619500672	0.989629514	0.713583853	1.372461794	0.950182951
LGALS8	0.138370321	0.639330886	0.260301744	1.570269852	0.329206682
LGALS9	0.776756972	2.418826886	0.501786312	11.65979095	0.271040738
LGI1	0.47919474	1.065525854	0.320235189	3.54534849	0.917584991
LGI2	0.670320222	1.245703105	0.667709239	2.324029885	0.48987303
LGI3	0.108270686	0.393705992	0.124780071	1.24222087	0.111836772

LGI4	0.775071551	0.685217754	0.000215406	2179.710115	0.926803845
LGMN	0.990146866	1.124882945	0.366038522	3.456908397	0.837228396
LGR4	0.824272708	0.783699214	0.201511272	3.047891327	0.725046637
LGR5	0.294384431	0.856423624	0.345933541	2.120237956	0.737549029
LGR6	0.461339973	0.510733408	0.125104051	2.085053304	0.34918479
LHB	0.518808328	0.809686154	0.032971359	19.88367146	0.897151186
LHCGR	0.232143656	1.766838626	0.806719475	3.86964593	0.154734452
LHFP	0.209203193	0.306406858	0.04314221	2.176178791	0.236979143
LHFPL1	0.402548333	0.56879869	0.159595587	2.027198596	0.384214728
LHFPL2	0.859048548	2.786435745	0.093792218	82.78111234	0.55369884
LHFPL3	0.500793876	0.000442332	1.86E-09	105.3632375	0.221455162
LHFPL5	0.75655998	98.60920361	0.085682885	113485.616	0.201708429
LHPP	0.024523477	0.414822177	0.197755603	0.870152027	0.019915081
LHX1	0.830220612	1.080563304	0.000274599	4252.081442	0.98536278
LHX2	0.279965204	0.783804846	0.062910771	9.765418961	0.849876511
LHX3	0.931834653	1.015348329	0.7185718	1.4346962	0.931186787
LHX4	0.759778348	0.114349944	0.000411567	31.77102651	0.450062855
LHX5	0.915346906	2.33027055	0.612573941	8.864498584	0.214595784
LHX6	0.773156979	0.956840548	0.483572542	1.893291603	0.899170726
LHX8	0.148613374	0.199196895	4.82E-05	822.8794687	0.704093112
LHX9	0.921115317	1.061112613	0.172183129	6.539316486	0.949024438
LIAS	0.90311091	0.856811287	0.447698436	1.639776963	0.640765074
LIF	0.349536457	1.347009535	0.550651897	3.295066626	0.51396277
LIFR	0.629760296	0.485777675	0.000658113	358.5707088	0.830332342
LIG1	0.865657568	0.895283493	0.328535705	2.439712095	0.828783847
LIG3	0.811056296	0.353165147	0.007336863	16.99985651	0.598487982
LIG4	0.357808447	0.990978783	0.243112164	4.039448014	0.989914925
LILRA1	0.461516129	2.633368454	0.876188393	7.914541524	0.084608288
LILRA2	0.606818657	1.077886066	0.002804054	414.3423828	0.980295057
LILRA4	0.600805117	0.044373773	9.54E-06	206.3582867	0.46968251
LILRA5	0.68077776	2.011672297	0.576570037	7.01879246	0.272952688
LILRA6	0.632517765	0.982581841	0.330521124	2.92104499	0.974782868
LILRB1	0.833983659	597.7228681	0.001706328	209381014	0.326348509

LILRB2	0.78233496	1.727265721	0.514976055	5.79337008	0.376070366
LILRB3	0.721491162	0.623620756	0.011774714	33.02864427	0.815642829
LILRB4	0.77010535	0.827743452	0.404589137	1.693469149	0.604716994
LILRB5	0.063757363	7.164122224	0.430372505	119.2563342	0.169951385
LILRP2	0.524122719	0.895140379	0.227749774	3.518230926	0.873964793
LIM2	0.119303852	5.154467621	0.02828434	939.3373241	0.536930997
LIMA1	0.787148966	1.531849275	0.488774358	4.800911018	0.464333469
LIMD1	0.213369932	0.896646011	0.17586202	4.571618535	0.895568133
LIMD2	0.63184371	0.013196632	4.85E-06	35.89542967	0.283463909
LIME1	0.093824537	1.787286066	0.871571234	3.665095125	0.113007302
LIMK1	0.118353884	0.141896641	0.010037628	2.005917791	0.148491745
LIMK2	0.148147729	0.582444801	0.21853207	1.552366875	0.27983538
LIMS1	0.500800687	0.172238113	0.003748115	7.91490397	0.367776398
LIMS2	0.97197783	0.891084685	0.003173997	250.1678604	0.968020024
LIMS3	0.596452246	2.894476589	0.001400978	5980.104864	0.784939526
LIN28B	0.211783646	0.942512717	0.884483412	1.004349216	0.067833657
LIN7A	0.164360511	0.003786362	1.01E-05	1.415159989	0.065027304
LIN7B	0.486955457	4.981371892	0.00046585	53266.21347	0.734439033
LIN7C	0.360336244	3.517860273	0.082472016	150.05503	0.511261881
LIN9	0.071573964	0.528262105	0.144712818	1.92837687	0.334059959
LIPA	0.335559423	0.173879084	0.009422446	3.208714243	0.239540147
LIPC	0.359518398	0.705660525	0.364339325	1.366739033	0.3013057
LIPE	0.11544982	2.112258133	0.365869033	12.19462162	0.403196156
LIPF	0.47454612	0.326740839	0.042202817	2.529678917	0.284082118
LIPG	0.251384019	0.699509377	0.39106948	1.2512185	0.228373283
LIPH	0.70644127	0.370741426	0.093967857	1.462725761	0.156510877
LIPI	0.341471581	10.09206398	3.25E-05	3138514.868	0.720157728
LIPT1	0.554397896	2.639006744	0.780777952	8.919765956	0.118356525
LITAF	0.214219382	0.668368818	0.289138771	1.544991266	0.345969121
LIX1	0.506843956	1.918025784	0.376570422	9.76928268	0.432965369
LIX1L	0.985336675	0.305650601	0.042785476	2.183504721	0.237393465
LLGL1	0.586589143	2.671604474	0.654309768	10.90839662	0.170991879
LLGL2	0.509601329	0.926459758	0.308768986	2.77983775	0.891621634

LMAN1	0.544243405	1.307720794	0.908743328	1.881866555	0.14854919
LMAN1L	0.071137957	1.18517156	0.901727028	1.557712681	0.223145662
LMAN2	0.849640339	1.076866655	0.002042802	567.6721047	0.981523761
LMAN2L	0.596109886	0.50216198	0.243290434	1.036484048	0.062455915
LMBR1	0.109672573	0.849232882	0.612511184	1.177442155	0.326980426
LMBR1L	0.254099652	1.117817857	0.842965915	1.482286223	0.439204582
LMBRD1	0.859027759	1.0163385	0.682786238	1.512836506	0.936353841
LMBRD2	0.965781969	0.549374665	0.158963339	1.898629737	0.343808385
LMCD1	0.538881377	0.285886774	0.013380712	6.108138664	0.422810206
LMLN	0.412328695	1.334934407	0.679154491	2.623924148	0.40212412
LMNA	0.574735483	1.38696767	0.264300858	7.278369564	0.698943998
LMNB1	0.759944439	0.916524669	0.430314412	1.952101641	0.821231936
LMNB2	0.914556207	1.002907083	0.875148207	1.149316891	0.966695351
LMO1	0.578414018	0.051216928	0.000662484	3.959601979	0.180372087
LMO2	0.094831651	1.609625834	0.851474219	3.042834732	0.142898575
LMO3	0.224244074	119.7803542	0.131783189	108870.7394	0.168546612
LMO4	0.872646309	0.798700784	0.430461215	1.481952196	0.47603314
LMO7	0.159329266	1.588800436	0.856183205	2.948302198	0.142178291
LMOD1	0.130136158	1.869920001	0.699475469	4.99888983	0.212198951
LMOD3	0.151372026	1.29471088	0.911985987	1.838050461	0.148554164
LMTK2	0.193797677	0.005028879	1.15E-06	22.00628967	0.215982778
LMTK3	0.380171263	0.780620669	0.392985045	1.550615316	0.479394757
LMX1A	0.149798115	0.538697845	0.158283057	1.833395015	0.322209078
LMX1B	0.130267096	1.118590587	0.880946944	1.420340817	0.357723791
LNPEP	0.150204763	15.368539	0.142479943	1657.720981	0.252594318
LNX1	0.057682337	1.318958477	0.856890439	2.030191241	0.208357115
LNX2	0.598185404	0.795851523	0.442769752	1.430494392	0.445312303
LOC124685	0.434250514	1.508265769	0.626353365	3.631920508	0.359376923
LOH12CR1	0.563670195	1.069969619	0.544291467	2.103349134	0.844521768
LONRF1	0.129495391	2.055350722	0.781663039	5.404459954	0.144132857
LONRF2	0.37501359	1.317817954	0.336621894	5.159035079	0.691858687
LONRF3	0.315108058	0.841786547	0.595637689	1.189657075	0.32910772
LOR	0.558995551	1.913994436	0.728298942	5.030042599	0.187886921

LOX	0.294226407	0.134354549	0.006625846	2.724353328	0.191126847
LOXHD1	0.095665071	0.469593956	0.17008696	1.29650435	0.144616304
LOXL1	0.247501084	5.31718452	0.65773905	42.98429783	0.11710006
LOXL2	0.731154714	0.914719446	0.032066104	26.09333742	0.958418238
LOXL3	0.392934475	0.9008343	0.383329378	2.116984718	0.810671099
LOXL4	0.979591759	1.17223182	0.664535288	2.067802062	0.583178133
LPA	0.840880755	10.44285248	0.075147017	1451.197565	0.351418738
LPAL2	0.639305283	1.087951578	0.802947987	1.474116202	0.586505214
LPGAT1	0.374650446	4.898395683	0.007350005	3264.525764	0.631964656
LPHN1	0.294995467	0.158128564	2.84E-07	88108.47055	0.784685875
LPHN2	0.274293925	1.364926998	0.283687561	6.567174479	0.697919304
LPHN3	0.39615693	1.255492807	0.661319076	2.383512357	0.486645439
LPIN1	0.402709483	0.624801198	0.165695507	2.355987461	0.487360874
LPIN2	0.828966159	4.027099706	0.184038076	88.12052576	0.376241831
LPIN3	0.330336788	1.698874786	0.521039895	5.539260174	0.379479722
LPL	0.051145562	0.689292558	0.355749829	1.33555716	0.270213779
LPO	0.744036513	0.529995072	1.89E-05	14847.91617	0.903284517
LPP	0.307482606	15.4663898	0.000637451	375258.9436	0.59498343
LPPR2	0.280807614	1.10066962	0.376591849	3.216940611	0.860854231
LPPR4	0.21532475	0.832178267	0.409379832	1.691633575	0.611764411
LPXN	0.270470949	0.049928687	0.001161944	2.145434436	0.118261602
LRAT	0.57204363	2.337455138	0.690025642	7.918106494	0.17258545
LRBA	0.659475514	1.023621753	0.807452788	1.297662859	0.847036884
LRCH1	0.274679846	0.930884723	0.210277916	4.120957562	0.924827244
LRCH2	0.457872838	1.004080191	0.665744855	1.514359475	0.984504742
LRCH3	0.691518093	1.58776382	0.337083198	7.478847837	0.558747951
LRCH4	0.928805535	0.634897773	0.191468027	2.105287173	0.457618875
LRFN2	0.97545397	1.176781766	0.000210905	6566.064647	0.970498427
LRFN3	0.785763793	1.071481788	0.495020202	2.31924519	0.860890348
LRFN4	0.904260999	1.603892891	0.678653667	3.790552577	0.281662621
LRFN5	0.434760664	6.327979799	0.263816887	151.7845534	0.255104327
LRG1	0.394776368	2.356084037	0.552332625	10.05034239	0.246895127
LRIG1	0.057325605	1.31435922	0.837151942	2.063592131	0.234965184

LRIG2	0.086023549	0.500150866	0.187166473	1.336515478	0.167106457
LRIG3	0.185183929	1.269322236	0.921832812	1.747799511	0.143945422
LRMP	0.158892427	2.466596742	0.555259236	10.95722339	0.23535296
LRP1	0.227314582	2866.400425	0.003480474	2360669922	0.252014469
LRP10	0.121637461	0.813258644	0.569985352	1.160362491	0.254360676
LRP11	0.854565312	0.046398247	0.001588021	1.355647472	0.074545939
LRP12	0.107465386	0.078266433	4.63E-05	132.3746484	0.50174502
LRP1B	0.437271379	0.856180461	0.346952429	2.11281121	0.736182206
LRP2	0.362620531	0.000228153	5.16E-11	1009.156102	0.282807175
LRP2BP	0.775794622	1.539361631	0.38607968	6.137681815	0.541006265
LRP4	0.225419124	0.436801835	0.124043446	1.538137231	0.197194986
LRP5	0.359906734	2.638976738	0.938100981	7.423719155	0.065933027
LRP5L	0.617114498	0.90209957	0.424394861	1.917515292	0.788854907
LRP6	0.153767719	0.295714747	1.92E-05	4549.494446	0.804379899
LRP8	0.340035195	1.089265969	0.322420967	3.679972678	0.890510828
LRPAP1	0.412565277	0.409512005	0.015483569	10.83084123	0.59315467
LRPPRC	0.333045594	1.07184969	0.794449967	1.446109642	0.649769274
LRRC1	0.059301304	1.53334917	0.884985395	2.656721446	0.127442336
LRRC10	0.61358498	0.158124197	0.000387079	64.59477769	0.54768599
LRRC14	0.173693004	0.055414872	0.001874205	1.638458837	0.094088846
LRRC15	0.550824289	1.849945988	0.206524819	16.57089047	0.582377452
LRRC17	0.309923017	2.296791093	0.090176198	58.49935365	0.614688152
LRRC18	0.931726629	0.981746222	0.22317094	4.318777552	0.980554401
LRRC19	0.254391183	2.605061187	4.04E-05	168105.5918	0.865447012
LRRC2	0.182406232	1.584244005	0.788755199	3.182012707	0.195987725
LRRC20	0.656298772	0.116084899	0.000440928	30.56213507	0.448862494
LRRC23	0.610440525	0.967846971	0.744207247	1.258692067	0.807401563
LRRC24	0.935487441	0.010481773	6.17E-09	17800.95487	0.53343418
LRRC25	0.904096659	1.325266599	0.747079485	2.350929982	0.335579423
LRRC27	0.780184134	0.748053687	0.100296713	5.579288717	0.77706405
LRRC28	0.978726242	0.880165816	0.642965569	1.204873015	0.425624399
LRRC29	0.082444725	0.882952783	0.099219034	7.857419979	0.911128903
LRRC3	0.903656657	1.592739857	0.808836	3.136383955	0.178204069

LRRC30	0.829809512	1.054882633	0.350527678	3.174577755	0.924275801
LRRC31	0.900900253	1.046993383	0.841699623	1.302359078	0.680051519
LRRC32	0.804021249	0.25585162	0.004384971	14.92827587	0.51116426
LRRC34	0.659259545	2.10025006	0.103546799	42.59958151	0.628936214
LRRC36	0.436103285	1.352220732	0.576484518	3.171812685	0.487871662
LRRC37A	0.081331025	1.746556705	0.57772627	5.280113583	0.323178843
LRRC37B	0.811526767	0.733640538	0.327949827	1.641191414	0.450862606
LRRC38	0.239380271	0.290851467	0.000501368	168.7275217	0.703663545
LRRC39	0.804665052	0.657174389	0.205250024	2.10415652	0.47953767
LRRC3B	0.91699918	0.850428356	0.223824403	3.231231185	0.811972474
LRRC4	0.441457734	0.403137541	0.060773122	2.674206493	0.346680851
LRRC40	0.890311414	55882.33728	0.113427439	27531571182	0.102153771
LRRC41	0.549412365	0.968101238	0.707676882	1.324361486	0.839311006
LRRC42	0.914644756	1.303791788	0.305110017	5.571344544	0.720349402
LRRC43	0.20519069	1.637541409	0.443908817	6.040749274	0.458974382
LRRC45	0.723112466	0.387640799	0.075254932	1.99675137	0.257162986
LRRC46	0.671757641	0.361700309	0.070347262	1.859732855	0.223492122
LRRC47	0.740547609	1.214493602	0.571017947	2.583096937	0.613772833
LRRC48	0.459061083	0.949185332	0.462648416	1.947381126	0.886895263
LRRC49	0.982175484	0.021417204	0.000188846	2.428942908	0.111315298
LRRC4B	0.217918094	0.013311892	3.89E-07	455.5883099	0.417482381
LRRC4C	0.953381882	0.583088883	1.21E-05	28063.23722	0.921885499
LRRC52	0.478100341	1.179246555	0.842840605	1.649923402	0.335960238
LRRC55	0.391997134	2.639392631	0.747797389	9.315883639	0.131474728
LRRC56	0.341024338	0.597039473	0.243541509	1.46363605	0.259592366
LRRC57	0.282383934	2.036917134	0.261044997	15.89393191	0.497327139
LRRC58	0.399330862	0.180131503	0.003556923	9.122310806	0.392013217
LRRC59	0.417716976	0.599917619	0.215673503	1.668731413	0.327615784
LRRC6	0.733138762	0.800525037	0.279804151	2.290317471	0.678261275
LRRC61	0.572038458	1.551372363	0.402771652	5.975485605	0.523309941
LRRC7	0.667020125	0.87338107	0.139365742	5.47332853	0.88504118
LRRC8A	0.171594215	3.896284445	0.464785087	32.66247759	0.209955262
LRRC8B	0.703875836	0.418651841	0.010194553	17.19245167	0.645982739

LRRC8C	0.161525323	0.064654086	0.000332077	12.58790329	0.30854861
LRRC8D	0.088881249	0.099231575	0.009245036	1.065101927	0.056406802
LRRC8E	0.666762294	1.630636993	2.20E-05	120633.4993	0.931879621
LRRCC1	0.746975825	1.191376185	0.641978363	2.210942448	0.57884422
LRRFIP1	0.940826709	1.028694143	0.717240822	1.475392376	0.877806249
LRRFIP2	0.539790712	1.265026806	0.551455664	2.901942845	0.578923446
LRRIQ1	0.555799635	9.06960778	0.392197758	209.7354804	0.168853359
LRRK1	0.715422674	6097.546193	0.00572295	6496661170	0.218392869
LRRK2	0.152950573	1.188183759	0.90194864	1.565256138	0.220152243
LRRN1	0.445988486	0.002212331	9.41E-09	520.2447691	0.332623913
LRRN3	0.872447835	4.923946252	0.287171678	84.42770853	0.271572161
LRRTM1	0.327747841	2.49007208	0.959901708	6.45947279	0.06068018
LRRTM2	0.27290154	31.45185943	0.758602387	1304.002569	0.069587531
LRRTM3	0.239741084	0.007962559	2.33E-09	27182.56537	0.528902764
LRRTM4	0.982563921	0.481362131	0.07300822	3.173745377	0.447380495
LRSAM1	0.950504691	1.114297059	0.605338143	2.051180732	0.728125831
LRTM1	0.609755607	1.75251792	0.266871194	11.50861963	0.559030209
LSAMP	0.355988871	123.4066713	0.006353672	2396914.136	0.339152309
LSG1	0.844430275	0.235761964	0.000855022	65.00850553	0.614284798
LSM1	0.278029638	0.47894794	0.089276472	2.569446602	0.390386766
LSM10	0.610640345	0.903840296	0.494428299	1.652266431	0.742546616
LSM11	0.253196656	1.870140795	0.424772543	8.233645623	0.407788884
LSM12	0.841741344	1.084356946	0.889273994	1.322235885	0.423528972
LSM14A	0.74436842	0.972764531	0.41817764	2.262844165	0.948885486
LSM14B	0.77126341	0.790500684	0.277232485	2.254033579	0.660124393
LSM2	0.619228892	1.604302516	0.190633511	13.50122837	0.663608853
LSM3	0.660297872	3.454929555	0.604627939	19.74195611	0.163265047
LSM4	0.414223728	1.148665129	0.060353292	21.86179971	0.926534318
LSM5	0.740560315	0.805161786	0.229887789	2.82000843	0.734713344
LSM6	0.589763471	0.402757983	1.59E-05	10229.47183	0.86049855
LSM7	0.414980761	0.566910561	0.179971872	1.785765637	0.332304639
LSM8	0.385203535	1.23873788	0.686599198	2.234886874	0.477026242
LSP1	0.757079098	0.918819587	0.203672563	4.145032698	0.912294654

LSR	0.790179994	0.611183835	0.069337021	5.387391548	0.657483466
LSS	0.130376228	1.681890219	0.710506249	3.981322769	0.236977081
LST1	0.159265023	24147.63678	0.09373549	6220785371	0.112384365
LTA	0.31572879	74.86679555	0.007921219	707597.7974	0.355462746
LTA4H	0.104393196	2.672667546	0.383511214	18.62566607	0.32098052
LTB	0.829297841	0.965533076	0.201478113	4.627073906	0.965007364
LTB4R	0.832508646	0.817077628	0.290874712	2.295200726	0.701449904
LTB4R2	0.243922266	0.209889187	0.009957794	4.424019174	0.315468624
LTBP1	0.795699381	0.922144771	0.627340023	1.355486576	0.680048175
LTBP2	0.516066524	0.646070837	0.294709719	1.416334444	0.275353153
LTBP3	0.414505864	1.255391648	0.44303092	3.557332276	0.668651123
LTBP4	0.361274269	1.198002101	0.449336559	3.19406246	0.71804811
LTBR	0.594668777	1.219230879	0.953681831	1.558721041	0.113748594
LTC4S	0.135300736	0.579149513	0.257830439	1.300909854	0.185887677
LTF	0.545949159	0.818932127	0.608823941	1.101549699	0.186646295
LTK	0.935548001	0.56864673	0.142506932	2.269076309	0.424005331
LTV1	0.510672354	0.168744312	0.000132531	214.8526172	0.625685302
LUC7L	0.314572632	1.366598532	0.637439736	2.929832328	0.422155698
LUC7L2	0.926447569	0.166625908	0.001980753	14.01698928	0.428110469
LUM	0.622221989	3.253988876	0.822025086	12.88092515	0.092806016
LUZP1	0.232826655	0.065187153	5.20E-05	81.66480466	0.453100148
LUZP2	0.410719994	0.814928648	0.465764199	1.425847462	0.473361994
LUZP4	0.543065422	0.986125979	0.669000941	1.453577097	0.943736057
LXN	0.189921428	0.618539154	0.285619013	1.339514064	0.223024459
LY6D	0.32060559	0.887893747	0.495494146	1.591048676	0.689500806
LY6E	0.678776872	1.06540009	0.62245917	1.823537041	0.817287745
LY6G5B	0.68408679	1.292491357	0.941254287	1.774795537	0.112790088
LY6G5C	0.679533256	0.438805044	0.155621703	1.23729443	0.119380276
LY6G6C	0.459777256	4.052683887	0.010642755	1543.232665	0.644393773
LY6G6D	0.938482878	0.382156902	0.047402109	3.080957819	0.36636652
LY6G6E	0.065873817	0.026697066	0.000190008	3.751066287	0.151003636
LY6H	0.743654256	0.967869014	0.838955721	1.116591024	0.654289927
LY6K	0.364807774	0.936156381	0.749751794	1.168905199	0.56033394

LY75	0.943525603	0.314046605	0.023229211	4.245743503	0.383364382
LY86	0.063058888	1.354486565	0.748673968	2.450511081	0.315824781
LY9	0.779752014	0.109344752	1.50E-05	797.3021876	0.625758381
LY96	0.430453994	3.604614552	0.611473746	21.24906612	0.156615154
LYAR	0.966603198	1.42509514	0.250677269	8.10163667	0.689510938
LYG2	0.531894749	0.356911583	0.010322154	12.34101654	0.568741806
LYL1	0.190228086	0.595762894	0.351646363	1.009347636	0.05418247
LYN	0.496851507	1.713704134	0.188201704	15.60443818	0.632683923
LYNX1	0.447274742	0.510908181	0.220912358	1.181586997	0.116437713
LYPD1	0.109042697	1.093044494	0.405748178	2.944551153	0.860327394
LYPD2	0.896735335	18.30847465	0.071060659	4717.100133	0.304687674
LYPD3	0.163959985	1.372230292	0.887173911	2.122487993	0.155027419
LYPD4	0.305010901	0.02398504	0.000397899	1.445797766	0.074474772
LYPD5	0.559463997	0.069102428	0.000327786	14.5678831	0.327696902
LYPLA1	0.239154662	0.040079263	0.000392296	4.094729682	0.172954106
LYPLA2	0.177527255	0.051585337	0.000743773	3.577766378	0.170497978
LYPLA2P1	0.399018063	3.508719718	0.680348154	18.09531486	0.133670561
LYPLAL1	0.773008366	0.858096301	0.483852117	1.52180643	0.600604135
LYSMD1	0.608405887	0.652822797	0.240840951	1.769539617	0.401919096
LYSMD2	0.773489416	0.778421134	0.50058437	1.210464206	0.266130268
LYSMD3	0.41207793	0.023129024	4.85E-05	11.01947103	0.231215943
LYSMD4	0.129812411	0.987866407	0.407966641	2.392058421	0.978415466
LYST	0.897826491	6.529013327	0.00582802	7314.322638	0.600455382
LYZ	0.3673039	0.250299866	0.021386005	2.929487003	0.26977259
LYZL1	0.491414946	1.571361886	0.004622191	534.2007581	0.87921273
LYZL2	0.698244723	1.037571996	0.743704124	1.447559064	0.828137972
LYZL4	0.114251527	1.817883703	0.71007057	4.654046085	0.212726154
LYZL6	0.785668062	0.038822813	3.23E-05	46.5952954	0.36915653
LZIC	0.125346888	0.872284345	0.00091573	830.9003153	0.968855251
LZTFL1	0.756629967	0.795769027	0.390142386	1.623121113	0.529902963
LZTR1	0.734111261	0.812459904	0.003876545	170.2781903	0.939295096
LZTS1	0.217980408	0.691869193	0.324216753	1.476428889	0.34085097
LZTS2	0.688606567	0.776133961	0.050052103	12.03513731	0.856211302

M6PR	0.752837221	0.38629434	0.035147472	4.245634396	0.436734219
MAB21L1	0.917531654	0.934804308	0.697642164	1.252589277	0.65159442
MAB21L2	0.448555675	0.07337332	0.002137943	2.518141976	0.147609866
MACF1	0.82035167	1.119399356	0.557478449	2.247719026	0.751155936
MAD1L1	0.273409914	1.010722416	0.49149409	2.078478304	0.976869536
MAD2L1	0.360970384	0.816852331	0.138272676	4.825593514	0.823362308
MAD2L1BP	0.071618208	0.660229813	0.293403164	1.485680662	0.315718396
MAD2L2	0.449173814	2.698348294	0.550249729	13.2323255	0.221106405
MADCAM1	0.884431315	1.01781753	0.494600236	2.094524928	0.961744645
MADD	0.239810095	0.360230161	0.006555494	19.79496402	0.61743943
MAEA	0.097665354	1.186563223	0.880537762	1.598945943	0.261008969
MAEL	0.830979795	2.060396853	0.186409542	22.77370109	0.555397808
MAF	0.387344139	1.901989687	0.725202236	4.988353023	0.191267089
MAF1	0.403873379	1.441841221	0.045646579	45.54352521	0.835450516
MAFA	0.571356331	1.368754312	0.099202232	18.88554648	0.814658794
MAFB	0.176479416	0.584846245	0.181034869	1.889388112	0.369963957
MAFF	0.700688052	1.040832018	0.220365394	4.916068127	0.959704502
MAFG	0.579913693	1.506188848	0.629450068	3.604106127	0.357527829
MAFK	0.305663632	1.329280504	0.735470415	2.402525818	0.345910469
MAG	0.508889319	1.219591234	0.293979933	5.059538459	0.784491047
MAGEA1	0.79177858	0.439529144	0.128541788	1.502903226	0.190027663
MAGEA10	0.369156661	1.13960569	0.750378677	1.730727656	0.539900566
MAGEA11	0.546005302	0.750267255	0.21931338	2.566651218	0.647045163
MAGEA12	0.415532067	1.198914916	0.39105251	3.675713462	0.750955168
MAGEA2	0.339254296	0.474033007	0.125748993	1.78695102	0.270223046
MAGEA2B	0.911855474	0.638379984	0.250788281	1.624992211	0.346444563
MAGEA3	0.491340161	2.01650607	0.16079745	25.28831602	0.586743608
MAGEA4	0.821521342	1.318426204	0.726523526	2.392555224	0.363247523
MAGEA5	0.793209555	1.150398157	0.46399314	2.852231653	0.76232233
MAGEA6	0.265946366	0.715271847	0.180882997	2.828424034	0.632852557
MAGEA8	0.803397285	0.677816618	0.16467247	2.789994993	0.590107838
MAGEA9	0.513403982	1.278253706	0.805555851	2.028329301	0.297360132
MAGEB1	0.566724505	0.750556934	0.401403807	1.40341397	0.368861525

MAGEB10	0.949963832	0.956672063	0.164118329	5.576594904	0.960722614
MAGEB18	0.251135226	0.51959583	0.011335608	23.81696947	0.737272698
MAGEB2	0.342415844	2.118729759	0.384635827	11.67082077	0.388440948
MAGEB3	0.886909607	0.939400075	0.010502506	84.02494872	0.978247292
MAGEB4	0.926116976	2.109054714	0.003568401	1246.527862	0.818728462
MAGEB5	0.275240881	0.628592013	0.201916791	1.956884894	0.422967987
MAGEB6	0.593762411	0.000723641	1.71E-08	30.68957553	0.183469196
MAGEC1	0.361925955	0.787408473	0.315652446	1.964223978	0.608325357
MAGEC2	0.493273056	0.606865342	0.227910421	1.615922355	0.317534643
MAGEC3	0.427125137	0.703394391	0.455641215	1.085862413	0.112255018
MAGED1	0.463400791	3315.671696	0.073387295	149803570.7	0.138251344
MAGED2	0.531339018	1.267822414	0.20689303	7.769105	0.797520526
MAGED4	0.323935984	0.199148042	0.018348762	2.161450579	0.184703837
MAGEE1	0.271054781	0.556872531	0.210323378	1.474429612	0.238635728
MAGEE2	0.070322153	1.897693527	0.964670059	3.73313206	0.063486286
MAGEF1	0.228991332	1.138412468	0.431737255	3.001786234	0.793280644
MAGEH1	0.96684676	1.397931083	0.413424324	4.726890031	0.58992926
MAGEL2	0.279564633	6.551184811	0.757260921	56.675343	0.087748357
MAGI1	0.742599208	1.049156128	0.828711035	1.328241734	0.690083649
MAGI2	0.161177904	0.511789608	0.040370009	6.488197764	0.605217933
MAGI3	0.465425044	0.807006772	0.11212971	5.80809432	0.831378791
MAGOH	0.871177856	1.202746236	0.691194309	2.092897017	0.513639878
MAK	0.98972737	1.353399086	0.850226442	2.15435441	0.201996271
MAL	0.125599821	0.871516989	0.753392447	1.008162301	0.064231188
MAL2	0.97784135	0.579969334	0.099759825	3.371742376	0.544114107
MALAT1	0.739398011	1.147462442	0.541372122	2.432098001	0.719677233
MALL	0.72301008	10.71113385	0.000973682	117829.4299	0.617470419
MALT1	0.155640025	0.698676294	0.356457046	1.369445686	0.296351196
MAMDC2	0.815352722	4.361247223	1.66E-06	11470830.33	0.845183576
MAMDC4	0.213296354	1.554963081	0.633943394	3.814078996	0.334887747
MAML1	0.202047135	1.148696177	0.723906081	1.822754278	0.55622277
MAML2	0.905625415	0.257184993	0.017398224	3.801774286	0.323071631
MAML3	0.782289652	14.80070704	0.000210526	1040540.991	0.636052529

MAN1A1	0.130985102	0.481656563	0.223390977	1.038506782	0.062381166
MAN1A2	0.593439609	1.225697646	0.028696523	52.35249952	0.915392849
MAN1B1	0.395021663	0.535839543	0.164545762	1.74494932	0.300315777
MAN1C1	0.163142598	1.585645667	0.818995368	3.069946764	0.171438829
MAN2A1	0.693773446	1.742012601	0.33176428	9.14687955	0.511836241
MAN2A2	0.82175469	1.22016607	0.249390216	5.969782048	0.805961453
MAN2B1	0.572551969	1.123211862	0.446583136	2.825016859	0.804976011
MAN2B2	0.64276259	0.81320015	0.327973862	2.016302399	0.65536647
MAN2C1	0.806951566	0.004882268	7.59E-06	3.140553495	0.106721762
MANBA	0.153279882	13.79790443	0.164680819	1156.067648	0.245389893
MANBAL	0.533610058	0.423316421	0.02307401	7.766174542	0.562518636
MANEA	0.231420459	1.105300463	0.836720977	1.460091413	0.48088416
MANEAL	0.950658929	1.008867806	0.825263202	1.233320772	0.931355027
MANSC1	0.260609839	1.936319211	0.001167532	3211.331569	0.861319889
MAOA	0.229637236	34.90824258	0.000558007	2183817.904	0.528364309
MAOB	0.745505402	0.126837669	3.92E-05	409.9350046	0.616499816
MAP1A	0.166311405	1.291687409	0.808847753	2.062756996	0.283859287
MAP1B	0.080416986	7529.465482	0.108716474	521474328.7	0.116473658
MAP1LC3A	0.18438059	0.029672093	2.58E-06	340.9686987	0.460874882
MAP1LC3B	0.536427767	2.910860636	0.831571322	10.18927592	0.094635478
MAP1LC3C	0.640986608	71.92466317	0.108580221	47643.64175	0.197031003
MAP2	0.877000222	1.126525386	0.9355124	1.356539416	0.208837432
MAP2K1	0.629317362	18.00391707	0.000202485	1600816.051	0.619070244
MAP2K2	0.321698839	1.037754283	0.226820274	4.747961608	0.961903414
MAP2K3	0.790529374	0.999490355	0.811579104	1.230910166	0.996172181
MAP2K4	0.688723473	0.461659009	0.006147547	34.6689576	0.725758069
MAP2K5	0.233531727	1.381838255	0.897479377	2.127599824	0.141901699
MAP2K6	0.662844118	1.033798353	0.824284246	1.296566129	0.773608446
MAP2K7	0.92344589	0.336447733	5.52E-05	2051.933879	0.806489315
MAP3K1	0.587126167	1.304367661	0.510661155	3.331710234	0.578648486
MAP3K10	0.36718835	0.256232381	0.037111382	1.769134661	0.167197697
MAP3K11	0.316499575	1.27106678	0.313879992	5.147224418	0.73677371
MAP3K12	0.077639664	1.217892532	0.974163568	1.522600791	0.08359318

MAP3K13	0.879322423	0.736205448	0.366768459	1.47776737	0.388997021
MAP3K14	0.103588363	0.045276141	0.000798668	2.566684233	0.132996104
MAP3K15	0.939776865	1.029498245	0.76876206	1.378666679	0.845311619
MAP3K2	0.559859363	1.141280671	0.509488237	2.556529229	0.748093032
MAP3K3	0.284130041	13.38236695	0.007584177	23613.33865	0.496453883
MAP3K4	0.118398923	1.868045386	0.371871679	9.383864813	0.447976399
MAP3K5	0.189881933	0.478798722	0.214858892	1.066971046	0.071638923
MAP3K6	0.774928668	0.964260919	0.758784065	1.225380399	0.765972122
MAP3K7	0.271644339	0.725567039	0.272618295	1.931079232	0.520661952
MAP3K8	0.174149289	0.178348714	0.020048893	1.586534626	0.122091274
MAP3K9	0.914872735	193.7651252	0.047229705	794943.014	0.214690635
MAP4	0.66776819	0.94359358	0.493525713	1.80409819	0.860626368
MAP4K1	0.190050912	0.734759312	0.12609496	4.281465698	0.731792737
MAP4K2	0.69599604	6.331651872	0.003657209	10961.86006	0.627602659
MAP4K3	0.543831471	1.477915344	0.286473691	7.624552729	0.640761703
MAP4K4	0.338528437	0.598662593	0.273545485	1.310191247	0.199184016
MAP4K5	0.14046888	0.310916564	0.027906602	3.464023002	0.34220462
MAP6	0.373551937	0.000797689	5.09E-07	1.249235812	0.057344698
MAP6D1	0.726628877	1.425401922	0.430973314	4.714376903	0.561383539
MAP7	0.20986287	1.431891332	0.320462519	6.397979997	0.638337788
MAPK1	0.300102089	0.883675927	0.509404661	1.532932862	0.65992957
MAPK10	0.171670036	0.518321208	0.138575784	1.938700014	0.328879422
MAPK11	0.463062356	1.040929354	0.896913846	1.208069119	0.59751012
MAPK12	0.456486237	0.281704559	0.069233089	1.146235992	0.076835359
MAPK13	0.616844401	0.395322692	0.008841486	17.67576503	0.632194784
MAPK14	0.450698222	0.900828179	0.418170791	1.940574106	0.789672113
MAPK15	0.92541926	0.587838122	3.90E-06	88599.19891	0.930403569
MAPK3	0.962221929	0.019265747	3.23E-05	11.49520979	0.225847406
MAPK4	0.338221457	0.507250989	0.247797923	1.038360461	0.06331439
MAPK6	0.913650837	0.86596361	0.484697405	1.547136349	0.626932334
MAPK7	0.501609185	0.111827144	0.000286662	43.62382638	0.471723175
MAPK8	0.866884779	0.854347662	0.328386491	2.222716061	0.746935792
MAPK8IP1	0.668309737	1.840091067	0.342932056	9.873486803	0.47682293

MAPK8IP2	0.707105713	14.35189845	0.003350879	61469.5457	0.532394654
MAPK8IP3	0.859904425	0.863270104	0.438905239	1.697941167	0.670103836
MAPK9	0.910050023	0.987540404	0.764899938	1.274985134	0.923370045
MAPKAP1	0.658519433	1.275850269	0.089493101	18.18904346	0.85739626
MAPKAPK2	0.848179013	0.880952909	0.405486871	1.913941198	0.748836845
MAPKAPK3	0.600653668	5.796203257	0.001502874	22354.48704	0.676621344
MAPKAPK5	0.765226976	0.989962937	0.896101598	1.093655697	0.842666832
MAPKBP1	0.817040967	1.040365747	0.859435075	1.259386448	0.684769141
MAPRE1	0.401008949	0.750398141	0.475281915	1.184764981	0.217820601
MAPRE2	0.949582246	1.238933052	0.678731314	2.26150625	0.485301283
MAPRE3	0.361082003	1.070752986	0.917600367	1.249467631	0.385370276
MAPT	0.840957344	1.174873431	0.902205689	1.529947767	0.23164172
MARCKS	0.832847599	81.44891292	0.194960573	34027.01027	0.153010239
MARCKSL1	0.087013341	1.155098788	0.895235852	1.490392958	0.267487587
MARCO	0.959244894	1.34900233	0.615002362	2.959024874	0.455078284
MARK1	0.717596415	1.036790511	0.7460547	1.440825403	0.829625769
MARK2	0.923631612	0.134981281	0.000857854	21.23899094	0.43778422
MARK3	0.237240578	372.6189656	0.60098099	231030.4251	0.071114081
MARK4	0.398844354	0.401507824	0.056440526	2.856254978	0.361998851
MARS	0.803572212	0.698609942	0.15954194	3.059106909	0.63406666
MARS2	0.558678449	0.443774965	0.044012602	4.474541636	0.490774596
MARVELD1	0.509927132	0.001386206	2.73E-07	7.037175334	0.130596223
MARVELD2	0.516976613	0.946151236	0.394111744	2.271442492	0.90141077
MARVELD3	0.214605863	0.003170963	4.02E-07	24.98800677	0.208788582
MAS1	0.481838105	1.135302341	0.206602912	6.238592651	0.883942451
MAS1L	0.817997257	208.73515	0.012630847	3449520.409	0.281124333
MASP1	0.796951637	0.027231457	1.65E-05	44.88219649	0.340369517
MASP2	0.674865323	0.13343821	0.000159066	111.9395853	0.557616036
MAST1	0.345550689	4.225777423	0.577443074	30.92459781	0.155840183
MAST2	0.735307037	82.16348833	0.008661426	779414.2177	0.345384373
MAST3	0.418200463	0.100699263	0.001708648	5.934718219	0.269705897
MASTL	0.305399844	0.438004127	0.010674579	17.97238239	0.6631218
MAT1A	0.123989005	5.02E-05	4.85E-10	5.195753406	0.09290459

MAT2A	0.100819204	0.696046234	0.434308528	1.115521176	0.132148596
MAT2B	0.242011891	0.0744532	0.003395757	1.632413541	0.099171064
MATK	0.961671037	0.2156311	0.007905253	5.881756181	0.363069521
MATN1	0.263862186	1.119717095	0.877466302	1.428848459	0.363312536
MATN2	0.748151538	0.693210383	0.238812839	2.012206033	0.500357232
MATN3	0.877720323	0.430868896	0.002546327	72.90815724	0.747753382
MATN4	0.433097718	1.003561648	0.762622273	1.320622302	0.979751054
MATR3	0.664036046	0.000442138	1.07E-08	18.25214333	0.154336814
MAX	0.206946035	0.087015857	6.06E-07	12502.06579	0.686959146
MAZ	0.436702254	1.238628516	0.350988951	4.371079484	0.739417555
MB	0.829466922	0.036700117	5.42E-07	2487.131006	0.560351991
MBD1	0.62507784	72.04514788	1.92E-05	270572669	0.579738522
MBD2	0.659054869	0.688469012	0.168554281	2.81208865	0.60312568
MBD3	0.198455616	0.911563819	0.805097118	1.032109765	0.143958069
MBD3L1	0.993256909	0.406657961	0.096764964	1.708993526	0.219311617
MBD3L2	0.384717583	1.112496758	0.919016689	1.346710077	0.274119656
MBD4	0.429582419	0.484197962	0.09877319	2.373596178	0.371213034
MBD5	0.591746824	1.110583992	0.942696168	1.308371503	0.209740821
MBD6	0.971221567	0.206638465	0.002701255	15.80726523	0.476134186
MBIP	0.646771873	1.383629697	0.587851674	3.256656778	0.457185244
MBL2	0.766077018	4.269529276	0.384830087	47.36864618	0.23712943
MBNL1	0.6839941	1.625766257	0.494246504	5.347768578	0.42373916
MBNL2	0.571935522	3.771099067	0.125977255	112.8869505	0.444036842
MBNL3	0.70558836	0.838569119	0.331907358	2.118657963	0.709665548
MBP	0.365028424	1.034417558	0.370317481	2.889465765	0.948521143
MBTD1	0.890436163	1.178129544	0.444755407	3.120792238	0.741539075
MBTPS1	0.130912753	0.83245115	0.445868335	1.554214244	0.564838702
MBTPS2	0.83949079	1.894628892	0.190023341	18.89040899	0.586003886
MC1R	0.832245189	0.70200906	0.24809813	1.986378213	0.504961611
MC2R	0.585953845	1.873151503	0.154549723	22.70270362	0.621970022
MC3R	0.052230301	1.683326562	0.951952275	2.976607537	0.073349002
MC4R	0.177000086	0.731054489	0.43283329	1.234749446	0.241422367
MC5R	0.834521839	0.012693865	2.88E-06	55.91481612	0.307718569

MCAM	0.048314168	1.759337456	1.206296372	2.565926878	0.003345769
MCC	0.356136146	1.235613679	0.39179419	3.896793786	0.71808285
MCCC1	0.391059603	0.501726029	0.151925683	1.656922017	0.257834718
MCCC2	0.463208677	0.616750575	0.111340981	3.416363572	0.580035613
MCCD1	0.574672386	0.917468579	0.550869832	1.528035379	0.740680524
MCEE	0.277934885	0.407153053	0.0989497	1.675332107	0.213129622
MCEMP1	0.447752585	13.85657407	0.022293926	8612.419593	0.423124296
MCF2	0.859005055	0.260143907	3.00E-06	22587.12169	0.816476049
MCF2L	0.211329987	1.448237091	0.814556916	2.574885354	0.207174716
MCF2L2	0.511565375	1.818551123	0.507118369	6.521412728	0.358699101
MCFD2	0.099464251	0.735982538	0.174048563	3.112179083	0.676899529
MCHR2	0.879444808	1.066189939	0.618815415	1.836995263	0.817395011
MCL1	0.745796501	0.096998201	0.001257197	7.483829263	0.292701407
MCM10	0.571567845	1.939352935	0.77868365	4.830061355	0.154832624
MCM2	0.655795192	0.765289778	0.346688153	1.689323502	0.507889427
MCM3	0.944401647	1.163993648	0.874451508	1.549406916	0.298050699
MCM3AP	0.60193637	0.79837581	0.351681309	1.812447571	0.59036226
MCM4	0.281484185	1.071191914	0.736155644	1.558708579	0.719326214
MCM5	0.151372026	2.202168045	0.920127421	5.270513615	0.076227332
MCM6	0.85336345	0.226677837	1.63E-07	314850.1794	0.837047808
MCM7	0.706849534	1.234532269	0.402067593	3.790581359	0.712795707
MCM8	0.586435064	0.801831949	0.522115132	1.231403642	0.312976602
MCOLN1	0.957782269	2.114329698	0.397172798	11.25552931	0.38014615
MCOLN2	0.434063341	9.043731229	0.000423073	193321.4249	0.665090904
MCOLN3	0.99832132	0.72431581	0.186958009	2.806156295	0.640677156
MCPH1	0.705839636	0.896376711	0.393611387	2.041331206	0.794460606
MCRS1	0.554369391	1.174288317	0.767091065	1.797639307	0.459597341
MCTP1	0.964134718	2.00423464	0.399798038	10.04746423	0.397937702
MCTP2	0.147048665	0.502329412	0.12127106	2.080750667	0.342374351
MCTS1	0.550015688	1.093801618	0.807625744	1.481381678	0.562346248
MDC1	0.740518334	1.097313452	0.753278014	1.598475979	0.628503045
MDFI	0.72884621	2.094148677	0.000698252	6280.628871	0.856406641
MDFIC	0.139426888	0.524563348	0.160585476	1.713521749	0.285401172

MDGA1	0.896764409	0.217292519	0.00017068	276.6348556	0.675585456
MDH1	0.200393852	33.49589868	0.441294958	2542.461018	0.111917423
MDH1B	0.24301371	1.141484528	0.660100562	1.973921857	0.63581898
MDH2	0.954771596	0.745480559	0.249625053	2.226304036	0.598753265
MDK	0.857427883	0.265799504	2.76E-05	2560.282224	0.777089001
MDM1	0.232258102	0.848863287	0.355024707	2.02963024	0.712561454
MDM2	0.172798379	2.48180493	0.867244516	7.102213507	0.090179681
MDM4	0.393719396	1.333267204	0.771575692	2.303858785	0.302677359
MDN1	0.176654939	1.147609844	0.065031595	20.25182296	0.925104289
ME1	0.748957441	1.046268	0.605938017	1.806582023	0.871071249
ME2	0.563867825	3.083335014	0.002261498	4203.831612	0.759782486
ME3	0.71160714	0.481110255	0.000222568	1039.981879	0.851852247
MEA1	0.373245052	3573.324616	0.051187988	249446194.2	0.150530169
MECP2	0.276719653	0.930631723	0.773486368	1.119703515	0.44616039
MECR	0.332641683	1.091783806	0.917635773	1.298981484	0.321948729
MED12	0.525484714	0.102313748	0.00498734	2.098935258	0.139150601
MED12L	0.166567041	0.002381118	1.78E-06	3.18460339	0.100056025
MED18	0.444498056	0.92138645	0.325920862	2.604782602	0.877281339
MED19	0.495577068	0.173615437	0.011723284	2.571149937	0.20293219
MED25	0.345949234	19.30510356	0.515590219	722.8357128	0.109248908
MED28	0.771603173	0.674232068	0.29934135	1.518630426	0.341368058
MED31	0.067854228	1.388565095	0.36175702	5.329856547	0.632404895
MED4	0.533794218	0.070841807	0.001354072	3.706273919	0.189809892
MED6	0.407767934	1.567455739	0.003134961	783.7155529	0.887278152
MED8	0.467345634	0.463320366	0.105499839	2.034749656	0.308187537
MED9	0.615648422	1.074241636	0.680810024	1.695032464	0.758269261
MEF2A	0.915445181	0.845138496	0.285593505	2.500964011	0.76116002
MEF2B	0.075114279	0.901753575	0.810235979	1.003608247	0.05822516
MEF2C	0.267227912	3.164498654	0.655700104	15.27230463	0.151448027
MEF2D	0.1502691	1.409548157	0.930566197	2.135072188	0.105169891
MEFV	0.744223512	0.713494083	0.493867412	1.030790438	0.072112432
MEG3	0.185758799	1.091061955	0.953174903	1.24889586	0.206132216
MEGF10	0.624260975	0.930717905	0.516675476	1.676556871	0.811023647

MEGF11	0.895741939	0.85106881	0.307158634	2.358123911	0.756457679
MEIS1	0.575977997	1.009303892	0.616706866	1.651829101	0.970608081
MEIS2	0.925044171	0.397907986	0.006875488	23.0282965	0.656275325
MEIS3	0.575033036	1.073843612	0.654993361	1.760537085	0.777597996
MELK	0.41412577	0.748814159	0.259804065	2.158252005	0.592247105
MEN1	0.247509142	1.200526528	0.813488347	1.771708164	0.357365641
MEOX1	0.370493455	0.012434156	4.41E-05	3.502232975	0.127396903
MEOX2	0.745511846	0.229544157	0.002604031	20.23421515	0.519589639
MEP1A	0.787904991	19.17198217	0.225154175	1632.503153	0.192760992
MEP1B	0.332068316	0.822460052	0.506648065	1.335129025	0.429113616
MEPE	0.12149925	0.31980306	0.015837365	6.457765888	0.457180747
MERTK	0.664577074	4.173584883	0.314519631	55.38226895	0.278762261
MESDC1	0.23518449	6.813197118	0.513913845	90.32575291	0.145630388
MESDC2	0.258184383	0.01962725	0.000112905	3.411988123	0.135274497
MESP1	0.233531059	0.941640275	0.230935342	3.839543993	0.93317202
MESP2	0.325188987	0.426755108	0.093275152	1.952502022	0.272400809
MEST	0.281637304	0.366881995	0.103979682	1.294506729	0.119066156
MET	0.537405772	0.633063554	0.255606627	1.567914996	0.323143424
METAP1	0.878838714	1.142084254	0.637541698	2.045915502	0.655130151
METAP2	0.241554249	1.181495004	0.931737204	1.498201895	0.168685442
METRN	0.836062397	1.770607417	0.747357654	4.194846476	0.194206632
METRNL	0.481086298	0.336453392	0.056367033	2.008281771	0.232082018
METTL1	0.854035957	1.278923592	0.898255789	1.820912901	0.172335567
METTL2A	0.858845451	1.14304556	0.599763977	2.178445525	0.684509961
METTL2B	0.64156327	0.207067535	0.02178464	1.968220016	0.170499169
METTL3	0.17661284	0.030378894	0.000411079	2.245011086	0.111478642
METTL4	0.770706814	1.816907538	0.418486874	7.888307153	0.425382575
METTL5	0.341889781	0.663888933	0.029746627	14.81675602	0.795987556
METTL6	0.086180182	1.89036631	0.754406067	4.736818728	0.174258139
METTL7A	0.343458792	0.678472548	0.360340676	1.27747165	0.229563448
METTL7B	0.297608462	1.117207217	0.743958242	1.677717776	0.593169546
MFAP1	0.95443197	0.923103444	0.172408191	4.942456409	0.925533305
MFAP2	0.368402913	0.191042063	0.000174118	209.6106495	0.643055638

MFAP3	0.617303599	1.473557256	0.556703142	3.900410872	0.435038025
MFAP3L	0.071003785	0.025729563	0.000193871	3.414702377	0.142226072
MFAP4	0.145191704	1.583515471	0.518973693	4.831692396	0.419334032
MFAP5	0.708556363	1.003387977	3.56E-05	28275.5346	0.999483793
MFGE8	0.657141044	0.902212017	0.540923303	1.504809496	0.693390333
MFHAS1	0.505761116	1.184040359	0.828086621	1.693001114	0.354457739
MFI2	0.168905345	0.364316471	0.077110943	1.721240683	0.202480831
MFN1	0.265744274	1.063950835	0.890972853	1.27051164	0.493498034
MFN2	0.564192745	1.636535285	0.1565633	17.10648496	0.680798977
MFNG	0.747070522	0.148435149	1.90E-05	1162.5057	0.676674801
MFRP	0.479781671	1.000095582	0.915251641	1.092804568	0.998313999
MFSD1	0.257874208	1.666171498	0.879442636	3.15668964	0.117366715
MFSD3	0.432429517	0.603402941	0.11465173	3.175661714	0.551035952
MFSD4	0.431590217	0.276940811	0.024071965	3.186121814	0.302921606
MFSD5	0.326651299	1.222893534	0.663175631	2.255011381	0.519260463
MFSD7	0.923083149	1.452822454	0.59927743	3.522063368	0.408415028
MGA	0.473104394	0.946798981	0.710405484	1.261854433	0.709138944
MGAM	0.701102817	0.68677746	0.118443587	3.982176583	0.675206442
MGAT1	0.859828382	0.933014933	0.500559505	1.739087674	0.827247889
MGAT2	0.208497886	0.895331462	0.515647008	1.554587565	0.694520405
MGAT3	0.900289056	1.149561349	0.879421098	1.502683183	0.307814643
MGAT4A	0.560460625	27.82798992	0.037974999	20392.28545	0.323062378
MGAT4B	0.888861745	1.086335934	0.830049418	1.421753617	0.546384984
MGAT4C	0.949736229	1.080279332	0.344191263	3.390566698	0.894729045
MGAT5	0.093881855	1.460739323	0.918538111	2.322994925	0.109383224
MGAT5B	0.294447995	1.570059345	0.641390492	3.843347192	0.323328515
MGC15885	0.128203213	15.03719674	0.259218182	872.3048811	0.190768541
MGC27382	0.669828628	0.205685694	0.000474201	89.2165722	0.609758715
MGC39584	0.324773559	0.569778599	0.116190136	2.794106818	0.488068635
MGC45922	0.584239283	6.027304331	0.138766701	261.7947772	0.350533015
MGC50722	0.7039462	1.993567472	0.706465497	5.625626846	0.192413975
MGEA5	0.546839329	2.264398204	0.350864847	14.61388703	0.390295969
MGLL	0.683123446	1.05491383	0.836115054	1.330968967	0.652163893

MGMT	0.265497312	0.86428033	0.481530356	1.551263548	0.62502702
MGP	0.973612436	0.310036654	0.029313197	3.279162121	0.330495218
MGRN1	0.579766623	1.914676396	0.786337549	4.662101799	0.152552758
MGST1	0.290069241	0.291405458	0.065423168	1.297967424	0.10570855
MGST2	0.412014995	1.761169844	0.350748274	8.843148907	0.491805759
MGST3	0.482914033	0.945022682	0.782770065	1.140907031	0.556292561
MIA	0.562149284	1.233821419	0.257223543	5.918258018	0.792818181
MIA2	0.377552351	1.059232359	0.862145075	1.301374006	0.583808923
MIB1	0.895647326	0.876360607	0.520987742	1.474138164	0.618909791
MIB2	0.34738566	322.264651	0.100272536	1035722.337	0.160987678
MICA	0.071161885	6.271946506	0.000712646	55198.9249	0.691946687
MICAL1	0.353761591	0.779741899	0.496588868	1.224347681	0.279819161
MICAL2	0.666938159	12.86865336	0.088367799	1874.011131	0.314765613
MICAL3	0.82606568	8.282403059	0.000820741	83580.79685	0.653111031
MICALCL	0.848309624	1.183804655	0.859697971	1.630099765	0.301242696
MICB	0.132874481	1.048002894	0.843323649	1.302358906	0.672357259
MID1	0.187997912	2.568991744	0.617426081	10.68908293	0.194605279
MID1IP1	0.55423149	0.822205899	0.292442457	2.311642937	0.710507823
MID2	0.823679339	1.0855225	0.495794399	2.376709179	0.83738459
MIDN	0.654814446	0.103286739	0.000230962	46.19001923	0.465950883
MIER1	0.561768449	0.953235422	0.257171384	3.533277126	0.942880905
MIF	0.742653016	1.416968798	0.298683484	6.722168061	0.660842368
MIF4GD	0.340559793	0.703779536	0.14870741	3.330739434	0.657821857
MINA	0.24904752	0.767036584	0.278597004	2.111814248	0.607762709
MINK1	0.224687585	35.28293213	0.296034438	4205.204334	0.144040552
MINPP1	0.681087987	1.242214703	0.517260926	2.983208845	0.627516245
MIOX	0.210279891	1.902857572	0.946314345	3.826283476	0.071053098
MIP	0.963557291	0.629150002	0.287042116	1.378995286	0.247129745
MIPEP	0.875355429	0.96192818	0.754105106	1.227025009	0.754623266
MIPOL1	0.660271124	95.94497013	0.019092572	482147.5815	0.293907408
MIS12	0.387640011	0.056186822	2.46E-06	1284.884768	0.573993441
MITF	0.066609967	1.640805441	0.826509783	3.25736313	0.15696585
MIXL1	0.093610502	2.06415813	0.793190372	5.371659742	0.137501061

MKI67	0.838554684	1.318801905	0.256600793	6.777993343	0.740397383
MKKS	0.923840122	1.137322231	0.661845476	1.954386493	0.641335582
MKL1	0.217779575	0.914891994	0.648214953	1.291280549	0.612901441
MKL2	0.010080595	0.175027348	0.064512392	0.474863377	0.000620698
MKLN1	0.318077773	1.253688921	0.128104521	12.26916818	0.84596474
MKNK1	0.189009527	1.126066912	0.038557254	32.88685146	0.945018211
MKNK2	0.095601104	1.980257057	0.703045803	5.577756096	0.195970749
MKRN1	0.223475314	0.252048472	0.000591442	107.4127669	0.655520158
MKRN2	0.792401412	0.717984514	0.325675365	1.582869989	0.411422001
MKRN3	0.521093887	2.1554349	0.587722964	7.904914213	0.246730343
MKS1	0.976682913	1.217508921	0.386942681	3.830872235	0.736487712
MLANA	0.651381841	1.344243278	0.72379348	2.496554668	0.348974448
MLC1	0.912705886	0.844516813	0.188396505	3.785678755	0.825264824
MLF1	0.708745064	1.072336007	0.142989796	8.041864148	0.945835039
MLF2	0.759442836	0.790143432	0.000581434	1073.770683	0.94897848
MLH1	0.732116434	0.507619516	0.013559127	19.00399448	0.713746657
MLH3	0.914394268	1.283947193	0.20730374	7.952198025	0.788205017
MLKL	0.086030105	0.000598794	1.28E-08	28.08646136	0.176311548
MLLT1	0.696007991	1.659192219	0.000317677	8665.792027	0.90771396
MLLT10	0.913729498	0.161643326	0.004174239	6.259480409	0.328649812
MLLT11	0.385069407	0.517652544	0.111406453	2.405283969	0.400835266
MLLT3	0.035433415	2.304329699	1.097496741	4.838224262	0.027399106
MLLT4	0.676101692	0.779325161	0.551862422	1.100541878	0.156802422
MLLT6	0.214115361	0.766499457	0.519335858	1.131293762	0.18061587
MLN	0.417304587	0.746730519	0.000523823	1064.494271	0.937176419
MLNR	0.540448522	1.009125863	0.474481725	2.146204912	0.981175594
MLPH	0.896828102	50130.26729	0.61255644	4102550448	0.060786054
MLX	0.905935782	0.17966215	4.52E-05	713.8514095	0.684746106
MLXIP	0.368541298	1.996965543	0.209734398	19.01391194	0.547488706
MLYCD	0.653128483	0.863352575	0.073837647	10.09481886	0.906768528
MMAA	0.457596918	0.579524609	0.179988687	1.865943789	0.360492359
MMAB	0.084587087	0.383693139	0.007154747	20.57660694	0.637296495
MMACHC	0.112385585	3.039180301	0.691535114	13.35668532	0.141115687

MMD	0.585658134	0.196653937	0.000366689	105.4647173	0.612023905
MMD2	0.556752802	0.008876938	3.12E-05	2.524849591	0.10127526
MME	0.510251375	0.968189958	0.135684252	6.90862631	0.974278648
MMP1	0.890424538	0.122471584	0.001523243	9.846944252	0.348169362
MMP10	0.732046952	14.35749038	0.076489202	2694.988619	0.318513861
MMP11	0.434760023	2.259782423	0.544677494	9.375486705	0.261419903
MMP12	0.701007214	5.963947741	0.530737163	67.0174902	0.147971163
MMP13	0.710944667	0.170013279	0.002714451	10.64838485	0.401247618
MMP14	0.056888302	1727.118003	0.017565021	169822543.7	0.2037752
MMP15	0.134944568	0.086358526	0.000436026	17.10402001	0.364035714
MMP16	0.370867249	0.942152169	0.562505081	1.578031452	0.820857596
MMP17	0.996419366	2.89367765	0.001961053	4269.833079	0.775336674
MMP19	0.308599502	0.600576759	0.284516691	1.267737378	0.181027878
MMP2	0.986050435	11.54031658	0.472432841	281.9001883	0.133597229
MMP20	0.081593643	0.317823706	0.045902467	2.2005769	0.245617562
MMP21	0.539405947	0.823191239	0.420580774	1.611209687	0.570133794
MMP23A	0.999282624	1.705291188	0.713883115	4.073521246	0.2296149
MMP23B	0.498195591	19.33724212	0.874642989	427.5217861	0.060769195
MMP24	0.214993351	14.74419763	0.89564874	242.7194433	0.05972075
MMP25	0.952599138	1.12686092	0.201760007	6.293692939	0.891750207
MMP26	0.284351501	3.660629886	0.137171249	97.6896491	0.438682668
MMP27	0.144678308	0.00153619	4.13E-08	57.19495571	0.227653401
MMP28	0.988604862	7.088156679	1.97E-05	2545794.816	0.764118441
MMP3	0.858039271	1.231731931	0.201493882	7.529576277	0.821483343
MMP7	0.444444531	1.371435935	0.464335314	4.050599785	0.567577772
MMP8	0.69260202	0.183087513	0.00124208	26.98781843	0.505135452
MMP9	0.894534028	0.023849015	8.85E-05	6.428390787	0.190757086
MMRN1	0.289719863	16021.07929	0.295954709	867277911.7	0.081680645
MMRN2	0.291762227	0.938561017	0.373417803	2.359011213	0.892737558
MN1	0.533191841	0.395867281	0.000280225	559.2322531	0.802273708
MNAT1	0.787934864	1.124799539	0.120688767	10.48294742	0.917753088
MNDA	0.782789602	10.67796363	0.00390895	29168.67679	0.557474052
MNS1	0.659806648	0.602259373	0.233662797	1.552306818	0.293871793

MNT	0.145335579	2.202297202	0.360155152	13.46673216	0.392787599
MOAP1	0.395474048	8.171411381	0.005243473	12734.30114	0.575443181
MOBP	0.490107249	2.705399948	0.185463742	39.46425754	0.466726045
MOCOS	0.421166711	0.013167767	3.30E-05	5.246451655	0.156371925
MOCS1	0.45787536	0.284919072	0.068644406	1.18260005	0.083808234
MOCS2	0.400825141	4.478130679	0.158449718	126.5616285	0.379208234
MOCS3	0.894101929	15.67292401	7.99E-05	3076025.454	0.658076944
MOG	0.150533618	1.185668824	0.851866097	1.650271756	0.312703488
MOGAT1	0.07092565	1.455277805	0.616961441	3.432683713	0.391482565
MOGAT2	0.494283249	0.305698758	0.024281056	3.848750592	0.359103611
MOGAT3	0.139285893	1.179246013	0.769200288	1.807879146	0.449471659
MON1A	0.894295148	1.053166365	0.221310148	5.011787319	0.948108125
MON1B	0.429014774	0.194610482	0.015248353	2.483759419	0.207760697
MON2	0.137829781	0.6754614	0.384693431	1.1860044	0.171927077
MORC1	0.194882738	28.15010533	0.191551871	4136.886925	0.189898642
MORC2	0.380625295	1.187453962	0.860247241	1.639118203	0.296178152
MORC3	0.637233219	0.115254223	0.000447393	29.69096203	0.445574558
MORC4	0.551486153	1.106491351	0.006498024	188.4146798	0.969204475
MORF4	0.33242334	0.230771686	0.017768287	2.997226018	0.26233971
MORF4L1	0.570176749	0.528211897	0.136233298	2.048014779	0.355938587
MORF4L2	0.274708563	0.813537332	0.331239278	1.998081251	0.652616482
MORN1	0.094516066	390.6970477	0.033119899	4608835.938	0.212177883
MORN2	0.811518142	8.079742104	0.00988751	6602.49482	0.54141677
MORN3	0.900607078	1.129609216	0.919129229	1.388288981	0.246697251
MOS	0.760085784	0.06673781	0.001129145	3.94452157	0.193392205
MOSPD1	0.125548313	2.419020672	0.547010081	10.69753778	0.244179397
MOSPD2	0.680492993	1.027383577	0.470601206	2.242911836	0.945930956
MOSPD3	0.596102447	0.706922396	0.247084114	2.02254716	0.517840294
MOV10	0.337135793	0.23088564	0.02435996	2.188352503	0.201440646
MOV10L1	0.059911998	0.36677579	0.11606543	1.159040036	0.087534196
MOXD1	0.100978393	3.118054906	0.298978173	32.51831498	0.341781849
MPDU1	0.081246165	10.72059813	0.790016592	145.479507	0.07461548
MPDZ	0.431140092	0.688607865	0.136016579	3.486198471	0.652098373

MPEG1	0.298897767	15.94330848	0.036103471	7040.572027	0.372870523
MPG	0.368329975	1.600200059	0.001452282	1763.184074	0.89534468
MPHOSPH10	0.452100731	1.412702312	0.859605433	2.321678931	0.172846606
MPHOSPH6	0.476454038	1.127594612	0.391326265	3.24912924	0.824002793
MPHOSPH9	0.372866165	0.777090841	0.417276999	1.447168613	0.426648598
MPI	0.530516042	1.024826375	0.717099143	1.464607942	0.892919861
MPL	0.426740669	0.789283851	0.187789192	3.317384734	0.746684961
MPO	0.926460788	1.087512981	0.859260283	1.376398406	0.485189232
MPP1	0.342038804	3.85434357	0.938166399	15.83510598	0.061285447
MPP2	0.754264616	1.112245874	0.784260259	1.577398408	0.550671177
MPP3	0.64376925	1.069656047	0.506192793	2.260332577	0.859979344
MPP4	0.410815405	0.679151845	0.30817394	1.496710682	0.33720964
MPP5	0.842591522	1.76844537	0.195538349	15.99378866	0.611864064
MPP6	0.128582075	0.001133197	5.37E-07	2.389878178	0.082411328
MPP7	0.02514617	2.270620208	1.09195997	4.721524843	0.028128203
MPPE1	0.515123254	1.085469799	0.837836685	1.406293976	0.534758497
MPPED1	0.815582367	1.220451866	0.853833915	1.744487695	0.274389781
MPPED2	0.222682788	5.721133181	0.437222042	74.86211061	0.183718929
MPST	0.104817823	1.564384677	0.312959417	7.819861891	0.585723894
MPV17	0.66417773	0.827202607	0.330122442	2.07275867	0.685646486
MPZ	0.344877357	0.894258136	0.503449382	1.588436977	0.702998749
MPZL1	0.749623098	1.063605535	0.203164455	5.568182364	0.941798491
MR1	0.250173266	1.075401029	0.791538807	1.461062127	0.642005382
MRAP	0.687670859	1.650508446	0.508368498	5.358668249	0.404300014
MRAS	0.92888739	0.158279711	0.00167567	14.95072061	0.42697159
MRC1	0.945815028	1.440798084	0.438230311	4.737004879	0.547583491
MRC2	0.573859506	0.515503809	0.078469277	3.386601574	0.49025733
MRE11A	0.276505509	0.434703484	0.126653668	1.491998793	0.185486496
MRFAP1	0.097757579	0.474667749	0.128789223	1.749443532	0.262884512
MRFAP1L1	0.146836188	2.027290112	0.900649779	4.563266757	0.087787417
MRGPRD	0.189090887	5.333237035	0.000122331	232512.6189	0.758750995
MRGPRF	0.334660205	3.346218268	0.08830632	126.799268	0.514857049
MRGPRX1	0.742470511	1.164288328	0.528825736	2.563353518	0.705609288

MRGPRX2	0.509597966	0.718563465	0.154133305	3.349914891	0.673912614
MRGPRX3	0.376478361	2.261961005	0.277387328	18.44521026	0.445868546
MRGPRX4	0.180106589	1.403332733	0.652049998	3.020232752	0.386233689
MRM1	0.778887872	0.071849561	2.30E-05	224.4858598	0.521295294
MRO	0.218621819	0.019764392	0.00012958	3.014586481	0.126074526
MRPL1	0.970332674	1.187909278	0.813622613	1.734377132	0.372513055
MRPL10	0.923031624	0.828991989	0.165550824	4.15115854	0.819507029
MRPL11	0.496076439	0.497759621	0.003126576	79.24471718	0.78740348
MRPL12	0.77452388	0.236297776	2.66E-07	209966.5996	0.836453942
MRPL13	0.323576166	0.935973048	0.39514923	2.216999248	0.880453926
MRPL14	0.178421254	0.013706515	2.08E-07	902.7100432	0.448570137
MRPL15	0.054348687	9144.43833	0.367864459	227314029	0.077345231
MRPL16	0.699407142	0.558065556	0.207868517	1.498241143	0.247029918
MRPL17	0.65077883	1.536023609	0.519871025	4.538372813	0.437468706
MRPL18	0.884219064	0.945254425	0.013292286	67.21988302	0.979355124
MRPL19	0.074627101	14.85470082	0.452608832	487.5338729	0.129796188
MRPL2	0.880648375	0.831970619	0.047267153	14.6438925	0.899956684
MRPL20	0.339843093	1.142476973	0.782376075	1.668319975	0.490496667
MRPL21	0.604229563	1.885604342	0.619167346	5.742395422	0.264308743
MRPL22	0.845146595	1.221877636	0.504278125	2.960637957	0.657199808
MRPL23	0.809561058	0.042666172	3.91E-05	46.6021913	0.376854464
MRPL24	0.157105268	2.81488865	0.008869116	893.3920537	0.724726941
MRPL27	0.923080952	1.24286262	0.702052316	2.200274049	0.455623814
MRPL28	0.302492281	0.587489171	0.127307574	2.711099705	0.49542498
MRPL3	0.263318977	0.713792316	0.248732176	2.048385849	0.530761969
MRPL30	0.434857096	1.450475265	0.198722133	10.5870366	0.713846709
MRPL32	0.619884907	1.086403156	0.788458637	1.496935618	0.612353599
MRPL33	0.21470006	2.690118158	0.805112305	8.988479809	0.107884836
MRPL34	0.697353917	1.097742272	0.788227944	1.528793929	0.581067922
MRPL35	0.977377534	0.539294554	0.10994089	2.645408976	0.446645057
MRPL36	0.89450334	1.374861031	0.463643968	4.076927524	0.565951046
MRPL37	0.35588056	1.175524188	0.875199564	1.578905171	0.282662992
MRPL38	0.463013342	25.33800179	0.329993517	1945.536207	0.144457282

MRPL39	0.128538917	1.10218295	0.920003524	1.32043761	0.291215659
MRPL4	0.881651773	1.475431657	0.550636823	3.953419899	0.439260117
MRPL40	0.895006679	0.41208166	0.081460278	2.084590173	0.283789705
MRPL41	0.522523696	0.946823131	0.000721052	1243.285996	0.988099301
MRPL42	0.829626072	2.479756096	0.846478354	7.264438917	0.097714019
MRPL43	0.551736731	0.42646704	0.062499974	2.909987383	0.38441427
MRPL44	0.984618571	1.217155406	0.334156179	4.4334577	0.765731738
MRPL45	0.17718907	1.428142176	0.458847487	4.44502832	0.538436791
MRPL46	0.330380097	14.16770281	0.119455934	1680.316712	0.276617743
MRPL47	0.501744942	0.711072255	0.352230616	1.435490638	0.341427427
MRPL48	0.431246425	0.217996874	0.040588187	1.170848986	0.075723039
MRPL49	0.163049241	1.826100009	0.639572408	5.213860384	0.260598779
MRPL50	0.052179898	6.345448028	0.419237252	96.04277874	0.182572423
MRPL51	0.387715289	3.308533988	0.252023842	43.43397454	0.362393226
MRPL52	0.319445833	1.317197342	0.492614254	3.522043508	0.582990407
MRPL53	0.530788094	0.042701465	0.000127592	14.29098931	0.287671981
MRPL54	0.526402025	1.0497494	0.827428905	1.331804821	0.689262653
MRPL55	0.527345804	1.50451721	0.036972599	61.22296188	0.828970151
MRPL9	0.28397711	0.823874028	0.600595681	1.130158667	0.229643363
MRPS10	0.356825909	40.63866478	0.036391172	45381.91467	0.300846387
MRPS11	0.810440162	1.066096779	0.647540152	1.755199794	0.801345024
MRPS12	0.99380095	0.775221455	0.397117638	1.51332564	0.455660026
MRPS14	0.891109633	0.513973352	0.018650057	14.16449356	0.694050964
MRPS15	0.370464548	0.044351159	6.28E-05	31.3021057	0.351870439
MRPS16	0.083725797	0.544495464	0.233307841	1.270747305	0.15977057
MRPS17	0.340354585	0.219618113	0.004177497	11.54569726	0.453344768
MRPS18A	0.453677316	135.9829869	0.832436784	22213.54595	0.058834691
MRPS18B	0.406735376	0.871736654	0.549743315	1.382326578	0.559519253
MRPS18C	0.805032346	1.869395615	0.317169567	11.01820708	0.489425662
MRPS2	0.566285873	0.93428322	0.699744334	1.247434372	0.644870446
MRPS21	0.342700069	1.117890458	0.302840329	4.126527937	0.867174726
MRPS22	0.390835006	8.42E-06	2.55E-11	2.786996435	0.071560682
MRPS23	0.383501661	0.560921573	0.195702247	1.607712819	0.281847908

MRPS24	0.206853144	0.56991322	0.246926785	1.31537402	0.187637243
MRPS25	0.07203934	0.076122578	0.000481485	12.03494415	0.318795529
MRPS26	0.77432477	0.972684284	0.795370911	1.189526426	0.787371056
MRPS27	0.475333561	0.078888763	0.001175808	5.292903364	0.236624879
MRPS28	0.586447926	0.872856526	0.489851924	1.555324126	0.644526718
MRPS30	0.886268611	7.505520545	2.80E-06	20100472.21	0.789530792
MRPS31	0.887089384	0.853952881	0.43205641	1.687824796	0.649703699
MRPS33	0.065738731	29.91681413	0.0027888	320932.2383	0.47293425
MRPS34	0.213351386	8.823687419	0.006425854	12116.28155	0.55472371
MRPS35	0.208423864	0.900938795	0.516771989	1.570694096	0.712991367
MRPS36	0.47269089	0.020593063	0.000161341	2.628428503	0.116562649
MRPS5	0.172358095	0.424657117	0.160817472	1.121356182	0.083850237
MRPS6	0.541286829	0.749455902	0.428243981	1.311598467	0.312479916
MRPS7	0.259359856	0.528886026	0.214163454	1.306107198	0.167281599
MRPS9	0.677894283	1.118045621	0.7826987	1.597071786	0.539676829
MRRF	0.491520287	1.240672755	0.944444547	1.629813938	0.121304443
MRVI1	0.383034042	1.034353261	0.518855984	2.062010852	0.923555629
MS4A1	0.699681106	0.486070417	0.088742611	2.662356291	0.405737214
MS4A10	0.261479022	1.744917769	0.449626659	6.771702615	0.421028146
MS4A12	0.186257353	0.734701824	0.030229957	17.8560219	0.849795835
MS4A13	0.581997704	1.430537392	0.128523459	15.9226747	0.770878512
MS4A2	0.992964732	1.02037731	0.764125829	1.362563358	0.891256117
MS4A3	0.348904312	0.006074302	1.22E-05	3.024454372	0.107247817
MS4A4A	0.062741216	2.185992753	0.917907546	5.205932051	0.077314616
MS4A5	0.493560151	0.808486456	0.498355842	1.311613699	0.389150491
MS4A6A	0.271834589	0.439146798	0.006986018	27.6051283	0.696905057
MS4A6E	0.504402007	0.035489743	5.52E-05	22.83519033	0.311615967
MS4A7	0.747168715	0.074562956	2.27E-05	244.7783851	0.5297042
MSC	0.09274741	0.098434796	0.002542285	3.811298861	0.213960113
MSH2	0.584125034	1.064086745	0.84107452	1.346231011	0.604703043
MSH3	0.207882961	0.927710251	0.154610184	5.566556418	0.934585073
MSH4	0.380440143	0.659744688	4.31E-06	101050.3922	0.945566758
MSH5	0.890578661	0.174803266	0.00387012	7.895410375	0.369655254

MSH6	0.077708515	2.773041883	0.716051206	10.73912204	0.139819563
MSI1	0.364017524	0.001165125	2.81E-08	48.35940487	0.21311111
MSI2	0.75962787	2.515052631	0.014690233	430.5915165	0.725221288
MSLN	0.655418932	1.458763157	0.124923543	17.03433879	0.763318201
MSMB	0.372570616	0.129253268	0.000747985	22.33521211	0.43637675
MSN	0.706399033	0.9471746	0.219586299	4.085590621	0.941989062
MSR1	0.880590511	1.100740658	0.461226247	2.626975384	0.828776091
MSRA	0.385553349	0.033269376	4.56E-06	242.8920246	0.453377433
MSRB2	0.421642589	1.91679912	0.665531644	5.520577268	0.227990395
MSRB3	0.203782119	0.498998563	0.09336262	2.667015612	0.416288589
MST1	0.570015133	0.003323942	2.79E-06	3.960031122	0.114306628
MST1R	0.132382978	1.570069706	0.958075931	2.572989053	0.073450136
MSTO1	0.097859965	0.721758583	0.267579256	1.946845435	0.519542908
MSX1	0.7874953	1.323232971	0.633777638	2.762712644	0.455843084
MSX2	0.074207007	0.160706282	0.004195468	6.15581095	0.325666542
MT1A	0.011826737	0.461388077	0.211985414	1.004215119	0.051252013
MT1B	0.132287157	0.532872424	0.165085787	1.720033112	0.292410692
MT1E	0.940911582	1.163996849	0.31364976	4.319750368	0.820444076
MT1F	0.230038424	70.7691173	0.015321448	326879.5383	0.322477165
MT1G	0.726560335	8.40E-05	1.62E-09	4.358054948	0.090222758
MT1H	0.885888318	1.477749299	0.596945758	3.658193331	0.398446055
MT1JP	0.476797773	0.020370761	1.15E-05	36.18804708	0.307767017
MT1L	0.238662585	0.368413905	0.091070782	1.490366084	0.16140181
MT1M	0.960862902	0.212843828	1.18E-05	3837.092495	0.756983688
MT1X	0.497837546	1.358111578	0.71857058	2.566855797	0.345974706
MT2A	0.893065658	1.06847424	0.429676722	2.65696777	0.886684388
MT3	0.871760742	35139.0383	0.062955888	19612970981	0.121053145
MT4	0.125348673	0.392379951	0.074207304	2.074755678	0.270890282
MTA1	0.423566084	0.183468988	6.99E-05	481.3433606	0.672893064
MTA2	0.841812846	1.017392105	0.854249027	1.211691982	0.846675494
MTA3	0.146761169	1.390789455	0.852852405	2.268030549	0.186151102
MTAP	0.668129753	1.10476408	0.690492687	1.767583777	0.677780027
MTBP	0.70498235	1.21541624	0.759507355	1.944993195	0.4160803

MTCH1	0.051791998	0.469524993	0.117717114	1.87274145	0.284124657
MTCH2	0.995784818	0.243751224	0.004149407	14.31883072	0.496981049
MTCP1	0.724723063	0.047161188	0.000746644	2.978900428	0.14876309
MTDH	0.189151657	1.217006054	0.579568826	2.55552692	0.603857562
MTF1	0.782480751	0.026540319	9.33E-07	755.1894838	0.487976909
MTF2	0.780352015	0.726169277	0.164006124	3.215256888	0.6733936
MTFMT	0.832914637	0.327933893	0.000398659	269.7559813	0.744764069
MTFR1	0.936709416	39.23791891	0.002526733	609329.9039	0.456098343
MTG1	0.460504452	3.526070063	0.337001313	36.89353607	0.292804546
MTHFD1	0.937489064	1.426840173	0.138493482	14.70013503	0.76516661
MTHFD1L	0.204786597	1.945601895	0.71115172	5.322867999	0.194924628
MTHFD2	0.938248519	0.052858561	1.05E-06	2663.168339	0.594573817
MTHFD2L	0.547808026	1.212857354	0.26024782	5.652393014	0.805876709
MTHFR	0.765724692	0.838777721	0.0006814	1032.504278	0.961376442
MTHFS	0.989956126	2.350898551	0.215324909	25.66690511	0.48338166
MTIF2	0.338478929	0.253061135	0.005413253	11.8302126	0.483620799
MTIF3	0.20010365	0.967584435	0.700049988	1.337361123	0.841829304
MTL5	0.64599622	0.493815685	6.65E-06	36680.35236	0.901865688
MTM1	0.101962114	0.001011215	2.60E-08	39.33058826	0.200902153
MTMR1	0.588911948	1.213335875	0.107274574	13.72351237	0.87584128
MTMR10	0.231218893	0.845069309	0.584653917	1.221478412	0.370472271
MTMR11	0.490414367	0.002326993	1.38E-07	39.15450873	0.221991324
MTMR12	0.172798379	1.266175915	0.978484753	1.638453172	0.072721519
MTMR2	0.945650384	4.515433715	0.261953652	77.83492036	0.299373114
MTMR3	0.889074798	0.872426073	0.420046946	1.812005204	0.714388433
MTMR4	0.917376179	1.054933167	0.485370609	2.292854094	0.892601062
MTMR6	0.861652647	0.275658908	0.015793923	4.811206899	0.377119313
MTMR7	0.675563545	1.544384245	0.26034351	9.161444806	0.63231886
MTMR8	0.208193171	1.759543158	0.581701995	5.322299306	0.317033087
MTMR9	0.849238608	0.040604072	0.00010989	15.00304521	0.288173478
MTNR1A	0.128964641	1.575435673	0.829408498	2.992491111	0.16496583
MTNR1B	0.027226845	0.660867628	0.436272347	1.001085733	0.050601651
MTO1	0.462401202	0.322514743	0.039247341	2.650262598	0.292338678

MTPN	0.588725098	1.191245481	0.383688572	3.698483351	0.762080449
MTR	0.266841443	0.389807667	0.10381297	1.463690106	0.162830527
MTRF1	0.151820204	0.484972909	0.04871982	4.827577794	0.537096513
MTRF1L	0.135233475	1.345528233	0.75810467	2.388121716	0.310633809
MTRR	0.691745951	1.124027778	0.318185392	3.970761945	0.855915554
MTSS1	0.122465372	74.47033262	0.000663595	8357247.784	0.467515716
MTTP	0.871670571	0.971741113	0.84104201	1.12275104	0.697307681
MTUS1	0.912704615	1.238867795	0.000455405	3370.174041	0.957664558
MTX1	0.18876448	0.068584603	8.82E-05	53.36134705	0.430121151
MTX2	0.770245352	1.274668707	0.029048874	55.93264282	0.899901018
MTX3	0.854482715	1.172715119	0.649683366	2.116816931	0.596991446
MUC1	0.375532114	1.697659587	0.692869974	4.159579984	0.247066852
MUC12	0.895174859	1.75945987	0.722319978	4.285772411	0.213554354
MUC13	0.286884855	0.273087181	0.036806038	2.026205842	0.204312405
MUC15	0.408386187	1.639970532	0.623651775	4.312508124	0.315955846
MUC17	0.674938453	2.167425226	0.011594491	405.1693129	0.771934923
MUC20	0.73721811	1.142669175	0.510266777	2.558843538	0.745760309
MUC4	0.24643687	1.516695772	0.589218197	3.904098815	0.38788655
MUC5AC	0.39333834	0.707318192	0.257278899	1.94457854	0.502163722
MUC5B	0.584517672	3.527216929	0.423328181	29.38915909	0.243901293
MUC6	0.217810895	0.968471854	0.666467906	1.40732618	0.866577134
MUC7	0.881043974	4.632423446	0.00050394	42583.13824	0.741966531
MUM1	0.610556598	0.303949825	0.006637005	13.91975585	0.541629091
MUM1L1	0.603182685	1.043838626	0.235961395	4.617700607	0.954902233
MUS81	0.244302332	0.494644977	0.033620819	7.277444696	0.607860888
MUSK	0.183328646	0.014065496	6.44E-05	3.071086353	0.120743177
MUSTN1	0.493452806	0.929449967	0.62418442	1.384009619	0.718729717
MUT	0.333247421	1.18658005	0.766099801	1.837844382	0.443455344
MUTYH	0.486389816	2.946267834	0.000462069	18786.15634	0.808973294
MVD	0.237997951	1.174349556	0.883120892	1.561617318	0.269066731
MVK	0.416689723	0.842339119	0.504809218	1.405551179	0.511318177
MVP	0.060724382	0.175070709	0.001779637	17.22247127	0.456703425
MX1	0.670097368	0.669057686	0.189530626	2.361825078	0.532306561

MX2	0.264132204	1.29163076	0.361468399	4.615368944	0.693689956
MXD1	0.115008387	0.477614479	0.129551768	1.760806466	0.266974177
MXD3	0.53683113	0.65395486	0.152466649	2.804921361	0.567533671
MXD4	0.608759613	0.810734641	0.328101911	2.003312492	0.649404874
MXI1	0.173804413	0.370361706	0.141599344	0.968703591	0.042889854
MXRA5	0.742336635	0.93910598	0.615425756	1.433024265	0.770765653
MXRA7	0.865618473	0.76143413	0.322920458	1.795432652	0.533450457
MXRA8	0.135661611	1.149587865	0.405232578	3.261219194	0.793292557
MYADM	0.912778985	8.829870599	0.005500272	14175.04703	0.563007855
MYADML	0.086664713	4.742281682	0.230934495	97.38361311	0.312755507
MYB	0.893169842	1.506049353	0.21555386	10.52258887	0.679719958
MYBBP1A	0.114970553	0.567699036	0.219766299	1.466476873	0.24229812
MYBL2	0.482799394	1.280831107	0.627117931	2.615980573	0.496947211
MYBPC1	0.930051024	1.028808341	0.641251274	1.650595713	0.90626451
MYBPC2	0.252355624	74.06069558	0.000620136	8844807.777	0.470456993
MYBPC3	0.340348895	2.979396176	0.034113606	260.2129378	0.632144162
MYBPH	0.282121485	1.150924899	0.71922057	1.841755058	0.557883577
MYBPHL	0.649614104	1.140827892	0.785309307	1.657293843	0.48924228
MYC	0.025914635	2.214087845	1.479799429	3.312736098	0.000110504
MYCBP	0.150700602	0.963716957	0.71799987	1.293524429	0.805600975
MYCBP2	0.070182336	1.270059697	0.453524363	3.556703378	0.649101411
MYCBPAP	0.749859358	0.898533873	0.455704387	1.771681694	0.757420178
MYCN	0.204186126	0.0211703	2.42E-07	1855.004367	0.506739979
MYCT1	0.430691972	0.977418086	0.85140894	1.122076678	0.745674653
MYD88	0.529011273	5.194835691	0.001675054	16110.71901	0.687917026
MYEF2	0.190647417	0.7573992	0.023755046	24.14870283	0.875004455
MYEOV	0.731575243	0.91192449	0.289252429	2.875019159	0.874950742
MYEOV2	0.127045568	1.06100576	0.892657528	1.261103152	0.501721507
MYF5	0.548770076	1.536605499	0.000100107	23586.26322	0.930393312
MYF6	0.557866554	0.002375786	8.61E-09	655.5391416	0.344492308
MYH1	0.850186437	1.154836545	0.381035347	3.500062282	0.799137933
MYH10	0.849961131	0.816524636	0.223351696	2.985034336	0.75924611
MYH11	0.584159314	15.8290872	0.000996244	251504.6556	0.575758759

MYH13	0.28856664	1.681206678	0.3831001	7.377852148	0.491157959
MYH14	0.351789668	2.786917737	0.606802875	12.79972591	0.187599506
MYH15	0.210281365	0.308538462	0.005908766	16.11097485	0.560108596
MYH16	0.706698317	3.068872822	0.000117128	80407.45283	0.828969307
MYH2	0.841314915	0.991231613	0.226608932	4.335840164	0.990667365
MYH3	0.903287614	1.134304423	0.519423165	2.477068043	0.751827378
MYH4	0.626771702	0.987790118	0.831807993	1.173022291	0.888583585
MYH6	0.192317628	0.933227263	0.659489173	1.320587449	0.69644102
MYH7	0.968060256	0.601128069	0.142466688	2.53641719	0.488393961
MYH7B	0.159060984	2.046616069	0.293961084	14.24895188	0.469450748
MYH8	0.12049436	42.37264321	0.318458405	5637.91334	0.133251381
MYH9	0.099480193	1.891252841	0.705019924	5.073384716	0.205615682
MYL1	0.387971709	1.049175972	0.758818991	1.450636098	0.771511539
MYL2	0.415918431	16.62173777	0.000191163	1445266.565	0.62811657
MYL3	0.461016777	0.470218747	0.055630069	3.974571202	0.488393496
MYL4	0.765204735	1.148948737	0.451704795	2.922446726	0.770669676
MYL5	0.41963964	0.053859908	0.000984114	2.947717623	0.15254888
MYL6	0.643209339	0.319366405	0.001574911	64.76234252	0.673655625
MYL7	0.263883097	4.45E-05	2.25E-10	8.77469931	0.107214557
MYL9	0.171851822	0.598252175	0.24494227	1.46118375	0.25949726
MYLIP	0.300813146	1.406612022	0.743107951	2.662543682	0.294651829
MYLK	0.406345941	0.368309054	0.043580611	3.112658503	0.359016424
MYLK2	0.491046711	1.247163839	0.855794001	1.817514075	0.250347374
MYLPF	0.303002722	1.14247336	0.769579293	1.696050543	0.508786276
MYNN	0.272136639	1.077697401	0.87665117	1.324850439	0.477518033
MYO10	0.251666831	0.93072351	0.356637454	2.428926752	0.883376331
MYO15A	0.913704483	5.655696875	0.513099268	62.34058233	0.157064892
MYO18A	0.495287647	0.742583962	0.278364134	1.980969793	0.552180266
MYO18B	0.227239336	0.674075567	0.209482486	2.169049448	0.5083252
MYO1A	0.968115956	0.907437529	0.376811702	2.185290063	0.828513728
MYO1B	0.267769283	0.367875677	0.000495378	273.190528	0.76684067
MYO1C	0.291729522	1.112724148	0.970261875	1.276103969	0.126495528
MYO1D	0.341939178	1.62720148	0.722724091	3.66361754	0.239692294

MYO1E	0.516491489	172.1922594	0.008539502	3472119.988	0.308629094
MYO1F	0.307732541	0.0483088	5.79E-05	40.28253659	0.377247704
MYO1G	0.859995588	0.347580463	0.105921171	1.140585749	0.08133346
MYO3A	0.991355116	0.621415745	0.238656595	1.618046747	0.329864995
MYO3B	0.428910513	0.163551481	0.012337837	2.1680532	0.169715657
MYO5A	0.829270457	0.780623582	0.469430791	1.298110795	0.339853622
MYO5B	0.231533946	0.843262108	0.625570776	1.136707485	0.263166847
MYO5C	0.31393083	0.533728028	0.246617261	1.155091933	0.110949213
MYO6	0.105777808	1.785659333	0.839723876	3.797175886	0.132022467
MYO7A	0.909588996	5.154534489	0.00026264	101162.2048	0.745058315
MYO9A	0.700167698	1.549787992	0.43739713	5.491217604	0.497268529
MYO9B	0.616436678	2.500686163	0.028068601	222.7909821	0.689063476
MYOC	0.549805709	1.455023351	0.839745393	2.521112912	0.181157693
MYOCD	0.375888976	1.014664649	0.761139326	1.352635864	0.920941446
MYOD1	0.37104376	0.955423507	0.578384725	1.578247208	0.858668941
MYOG	0.391304575	1.068131967	0.82088535	1.389848044	0.623663525
MYOM1	0.239970423	2.241594158	0.462871677	10.85558831	0.315911994
MYOM2	0.954396064	0.776129848	0.04922788	12.23651188	0.857064439
MYOM3	0.98722157	1.265684777	0.327041352	4.898334549	0.732923108
MYOT	0.245359416	1.707679703	0.630161415	4.627655545	0.292757209
MYOZ1	0.314945447	0.003057241	2.03E-06	4.600729745	0.120874133
MYOZ3	0.425228706	0.065741469	3.98E-05	108.6045293	0.471520012
MYRIP	0.882303934	0.681515607	0.174522453	2.661339641	0.58117454
MYSM1	0.482476061	3.355082675	0.212847996	52.88553326	0.389607518
MYT1	0.121930587	0.737900366	0.326186877	1.669279142	0.465542098
MYT1L	0.372047211	0.993126754	0.789373304	1.249473151	0.953055112
N4BP1	0.250980282	1.007988689	0.575167301	1.766514185	0.977824358
N4BP2	0.214907729	0.933690809	0.692204015	1.259424257	0.653182934
N4BP3	0.186379515	0.999210012	0.432038768	2.310951522	0.998525981
NAALAD2	0.700552114	0.133294067	7.79E-05	228.0939249	0.595749855
NAALADL1	0.313027083	0.39520318	0.100858907	1.548554886	0.182749398
NAALADL2	0.84655751	0.601768859	0.250318087	1.446662382	0.256434953
NAB1	0.377747155	1.855137628	0.606978186	5.669949424	0.27832144

NAB2	0.909427956	1.410939441	0.662257003	3.006008387	0.372351733
NACA	0.835117814	1.060934633	0.357573868	3.147831529	0.915107718
NACAP1	0.969959469	0.739110301	0.092422966	5.910695807	0.775652401
NADK	0.596077807	1.713684142	0.000656402	4473.952566	0.893252149
NADSYN1	0.276734607	1.30627474	0.063089143	27.04670912	0.862805716
NAGA	0.408978713	2.720987174	0.369033443	20.06260228	0.326096784
NAGK	0.665321561	0.348257671	0.002181656	55.59236252	0.683611126
NAGLU	0.238966122	7.409304298	0.204835898	268.0086383	0.273990392
NAGPA	0.949332938	2.365363387	0.000139749	40035.77256	0.862412169
NAGS	0.343117726	0.236405657	0.050207087	1.113142335	0.068095587
NANOG	0.234104263	0.000219205	3.73E-11	1287.269246	0.289356106
NANOS1	0.541561438	1.252901655	0.801901564	1.957550187	0.322033412
NANOS2	0.25689971	0.937845053	0.207482865	4.239161356	0.933554556
NANOS3	0.870044802	1.040201108	0.761194487	1.421474226	0.804618578
NANP	0.719818056	0.968721761	0.533295163	1.759666908	0.91689602
NANS	0.090351666	0.556809157	0.042777685	7.247620788	0.654726233
NAP1L1	0.950942242	1.957875106	0.274445707	13.96733429	0.502739812
NAP1L2	0.251978662	0.013196097	6.41E-05	2.717531334	0.111345626
NAP1L3	0.771212009	1.011316766	0.725207141	1.410302716	0.947120441
NAP1L4	0.677793752	1.032327329	0.5250708	2.029630508	0.926507272
NAP1L5	0.369105329	1.172730483	0.673324315	2.042547334	0.573554442
NAPA	0.994147845	5.103642787	0.79432443	32.79160089	0.085914545
NAPB	0.158343519	1.174561223	0.801332127	1.721625804	0.409536689
NAPG	0.783858625	0.782160107	0.424359204	1.441642901	0.430974781
NAPSA	0.977581017	1.044350254	0.212624061	5.129558012	0.9573833
NAPSB	0.580443381	0.055843828	0.00045449	6.861615898	0.239845719
NARF	0.849149618	0.137096445	0.001048384	17.92799823	0.424205597
NARFL	0.69789864	1.636384301	0.422328827	6.340447082	0.476060131
NARS	0.826786331	0.855117759	0.599316012	1.220101526	0.388118274
NARS2	0.250674786	1.418384066	0.463592303	4.339617693	0.540145482
NASP	0.258783914	0.664462545	0.130090827	3.393863229	0.623213234
NAT1	0.174142486	1.011279367	0.080056123	12.77461268	0.99308426
NAT10	0.578830447	0.533226014	0.007645691	37.18826318	0.771554585

NAT2	0.993002187	0.468014898	0.000230221	951.4240377	0.845109728
NAT6	0.095488131	0.969681315	0.853866502	1.101204757	0.635198963
NAT8	0.406914936	1.730745354	0.501052169	5.978378437	0.385760065
NAT9	0.382472857	0.753245557	0.187232977	3.030336213	0.68991425
NAV1	0.37468278	1.646956957	0.516278437	5.253884384	0.399242355
NAV2	0.785779251	0.704038842	0.218037502	2.273327696	0.557357109
NAV3	0.633302922	1.467363366	0.490623972	4.388605874	0.492691043
NBEA	0.240951902	0.417157925	0.030563405	5.69376145	0.512066447
NBEAL1	0.176227274	0.003575175	3.15E-07	40.55077367	0.236932629
NBEAL2	0.577386055	0.941188728	0.758040677	1.168586658	0.583039355
NBL1	0.616950034	0.766453437	0.151427381	3.879423052	0.747855978
NBN	0.986710964	1.183171664	0.609761293	2.295808547	0.618966925
NBPF1	0.587708492	0.82490065	0.570962373	1.191779204	0.30518383
NBPF10	0.420725315	0.106799784	0.003780807	3.016867173	0.189456499
NBPF11	0.211033735	0.869599156	0.234620478	3.223089039	0.83441923
NBPF12	0.189238023	0.077611067	0.00268106	2.24667786	0.1366024
NBPF14	0.965830448	0.740648804	0.310594406	1.766163979	0.498334196
NBPF15	0.164119813	16.80764778	0.170663803	1655.28377	0.228214145
NBPF3	0.617382274	1.52882428	0.804923167	2.903759978	0.194648573
NBPF4	0.83228937	0.010531447	9.17E-06	12.0884504	0.205274395
NBPF9	0.374319199	1.382576106	0.373631728	5.116044889	0.627493484
NBR1	0.355905688	0.464787958	0.127499236	1.6943462	0.245656468
NBR2	0.216664814	1.605340976	0.901356193	2.859157866	0.10798749
NCALD	0.329123444	4.203674022	0.68342776	25.85624452	0.121312284
NCAM1	0.724943113	1.467212216	0.626285868	3.437266906	0.377445121
NCAM2	0.577670167	0.978664872	0.755407607	1.2679048	0.870327704
NCBP1	0.766566997	1.002599241	0.423767689	2.372066735	0.995286087
NCBP2	0.156943709	1.185597952	0.96445906	1.45744134	0.106011302
NCDN	0.190108201	1.258898236	0.467373883	3.390914269	0.648808993
NCF1	0.933212877	0.765312519	0.469433453	1.24768111	0.283457966
NCF2	0.440304776	0.051862552	1.15E-05	233.6360165	0.49057347
NCF4	0.996820412	58.36567733	0.008305752	410143.7405	0.36818881
NCK1	0.135475498	0.848091666	0.088973985	8.083930097	0.886107225

NCK2	0.512797576	0.517404126	0.189988873	1.409066883	0.197368528
NCKAP1	0.132153707	2.662027667	0.795118066	8.912376165	0.112265152
NCKIPSD	0.281089528	1.153153404	0.000710192	1872.400105	0.969862263
NCL	0.19939317	2.706101851	0.697538367	10.49832894	0.150087868
NCLN	0.495224116	1.123661931	0.260719598	4.842812536	0.87570006
NCOA1	0.544644866	0.790448841	0.339464393	1.8405741	0.585556073
NCOA2	0.742254231	0.94378306	0.712328534	1.250443329	0.686910196
NCOA3	0.995994491	0.939355719	0.34345002	2.569192361	0.903003887
NCOA4	0.687209488	0.068339514	2.74E-07	17044.09295	0.672144993
NCOA5	0.461620824	1.105988684	0.012723099	96.14096132	0.964728955
NCOA6	0.68186499	0.897042762	0.143930904	5.590777894	0.907349282
NCOA7	0.513325162	0.848094535	0.321261874	2.238872391	0.739387056
NCOR1	0.493482847	0.027616476	2.10E-07	3623.515942	0.550529454
NCOR2	0.069623793	1.892257976	0.725502741	4.935391761	0.192264326
NCR1	0.813622027	0.930112434	0.36127958	2.394569714	0.880638637
NCR2	0.328503853	0.743739584	0.324637874	1.703894131	0.483935605
NCR3	0.966800112	0.452058088	0.10844704	1.884389984	0.275690102
NCSTN	0.167404405	0.042051617	0.000758282	2.332031347	0.121940269
NDE1	0.286041325	1.207818945	0.480712873	3.034715079	0.687915831
NDEL1	0.826593819	0.006167299	6.26E-07	60.75053031	0.278095214
NDFIP1	0.14670675	0.470788595	0.06294784	3.521040627	0.463055399
NDFIP2	0.861766117	1.253671387	0.419449013	3.74703933	0.685698263
NDN	0.2235131	0.049826079	8.09E-05	30.68492956	0.360083552
NDNL2	0.314040933	0.495945548	0.162292366	1.515548708	0.218526381
NDOR1	0.390000223	0.393108224	0.000210253	734.9913749	0.808076079
NDP	0.378785596	0.562328957	0.253717768	1.246321287	0.156281322
NDRG1	0.427697143	0.001567752	1.64E-07	15.01840419	0.167362695
NDRG2	0.950956952	1.193675003	0.523709591	2.720706354	0.673628451
NDRG3	0.10515532	0.062093673	0.001468955	2.624739114	0.145720808
NDRG4	0.176084318	0.525427704	0.212162664	1.301238716	0.164265852
NDST1	0.418051558	3.299250185	0.873126914	12.46674637	0.07841939
NDST2	0.151753351	2.330394447	0.708921999	7.660558263	0.163501105
NDST3	0.082856453	2.597271145	0.453605589	14.87154826	0.283698894

NDST4	0.129176544	22.4773742	0.062661982	8062.82116	0.299716565
NDUFA1	0.05476462	4.006954219	0.356071278	45.09120248	0.261070076
NDUFA10	0.344188606	0.002717908	1.60E-06	4.604177842	0.119368816
NDUFA11	0.436446556	1.037660446	0.828782922	1.299181212	0.747174273
NDUFA12	0.621989319	0.059730927	0.001196839	2.981004937	0.157811537
NDUFA13	0.074388044	4604.274595	0.071270868	297447541.6	0.135548123
NDUFA2	0.790260516	2.215764975	0.255344635	19.22740388	0.470496694
NDUFA3	0.214974233	0.909621611	0.77467145	1.068080509	0.247632793
NDUFA4	0.261805348	2.08822331	0.600010519	7.267666908	0.24719756
NDUFA5	0.372701292	1.292968616	0.532506946	3.139429175	0.570247876
NDUFA6	0.39079801	0.789385894	0.181551751	3.432245006	0.752466498
NDUFA7	0.817570495	0.687162769	0.168668864	2.799524809	0.600615694
NDUFA8	0.434769512	0.301526644	7.88E-06	11535.95391	0.823780992
NDUFA9	0.317858189	1.959743629	0.248721617	15.44134016	0.522934945
NDUFAB1	0.924842849	0.503716028	3.52E-06	72164.22987	0.909867299
NDUFAF1	0.124858569	0.166701869	0.010633252	2.613453766	0.202015093
NDUFB1	0.325867533	0.543751691	0.143219997	2.064417734	0.370745596
NDUFB10	0.007731678	0.447053773	0.222423277	0.898543885	0.023801496
NDUFB11	0.969915703	1.074091301	0.330090567	3.495016938	0.905487072
NDUFB2	0.053545251	0.297116965	0.071938542	1.227137617	0.093520726
NDUFB3	0.54995288	1.434918464	0.932761151	2.207415045	0.100337116
NDUFB4	0.161226709	0.793304141	0.412403066	1.526010624	0.48786599
NDUFB5	0.645292787	0.062741118	0.000637697	6.17290986	0.236986381
NDUFB6	0.165411225	1.420781364	0.397755033	5.075032416	0.588728548
NDUFB7	0.320695953	1.164973073	0.890737473	1.523638897	0.264830284
NDUFB8	0.553870726	0.013905156	2.06E-05	9.379053874	0.198290615
NDUFB9	0.434149679	0.612952531	0.183234726	2.050434508	0.426921288
NDUFC1	0.782188423	1.088067823	0.333564923	3.54920889	0.888724936
NDUFC2	0.611590381	0.772973601	0.002445172	244.3542454	0.930129131
NDUFS1	0.598498466	0.488739732	0.181429132	1.316583086	0.156781076
NDUFS2	0.352943871	1.389476579	0.394715607	4.891230869	0.608470476
NDUFS3	0.421530633	3.890108423	0.561887714	26.93232683	0.168809052
NDUFS4	0.441953902	0.843529477	0.567795147	1.253166714	0.399482632

NDUFS5	0.385596758	0.993698631	0.514517591	1.919151039	0.984982118
NDUFS6	0.380933743	0.014448084	2.77E-06	75.38789885	0.331947416
NDUFS7	0.146777869	4.037472275	0.648845871	25.12335071	0.134595651
NDUFS8	0.8908917	1.14714855	0.399507594	3.293929364	0.798658594
NDUFV1	0.078372021	2.505630635	0.871703358	7.202203383	0.08817891
NDUFV2	0.208323943	0.571909778	0.212704416	1.537724513	0.268176844
NDUFV3	0.851868041	116.6496782	0.002942867	4623773.012	0.378308428
NEB	0.085118786	1.426961608	0.731120156	2.785068108	0.297377829
NEBL	0.525612484	0.834436088	0.371549378	1.874000133	0.661048367
NECAP1	0.326052832	0.75369668	0.373871305	1.519396322	0.429229745
NECAP2	0.257716004	0.496414889	0.036004667	6.844327773	0.600863071
NEDD1	0.758891676	1.285637998	0.727543436	2.271843826	0.387063167
NEDD4	0.299793844	0.151263921	0.012100599	1.890879411	0.142749641
NEDD4L	0.692885764	1.133357077	0.581069269	2.210576832	0.713423808
NEDD8	0.619254318	1.051751598	0.702487765	1.574662904	0.806426336
NEDD9	0.463993238	1.055061139	0.428306389	2.598966618	0.90723428
NEFH	0.972028578	1.02717512	0.647822943	1.628668354	0.909232516
NEFL	0.297609007	0.847366771	0.629489981	1.140654286	0.274767726
NEGR1	0.552479851	0.016805708	4.63E-06	61.02747829	0.328589496
NEIL1	0.1761531	0.868700315	0.551231441	1.369007972	0.544159068
NEIL2	0.488183896	0.817060165	0.289590618	2.305279493	0.702626758
NEIL3	0.803205322	1.09494119	0.421263721	2.84595172	0.852359876
NEK1	0.268742771	0.0147958	3.80E-05	5.760549233	0.166186844
NEK10	0.515691753	1.230340295	0.708635615	2.136129215	0.461479356
NEK11	0.574171008	0.675350377	0.302493494	1.507794847	0.338129586
NEK2	0.553883724	1.299671884	0.527324225	3.203241811	0.569008287
NEK3	0.772712406	1.10327368	0.674925406	1.803477542	0.695078726
NEK4	0.841774849	0.011033263	1.49E-05	8.183430852	0.18136708
NEK6	0.732929652	1.259820995	0.38435867	4.129343409	0.702960432
NEK7	0.31966488	0.220978404	0.004324448	11.29195098	0.451938597
NEK8	0.881589617	1.418673532	0.338950092	5.937849374	0.632090256
NEK9	0.316032247	1.904674466	0.731317071	4.960618267	0.187080295
NELL1	0.386988682	1.927423232	0.583271115	6.369182737	0.281939225

NELL2	0.490908116	0.941494163	0.687194777	1.289898133	0.707443845
NENF	0.728661407	0.601994259	0.165609027	2.188268926	0.440877075
NEO1	0.402904729	1.654069502	0.155856853	17.55422281	0.676259717
NES	0.056695813	0.297188772	0.054750558	1.613155544	0.159753119
NET1	0.536506406	0.877860205	0.434348091	1.774241806	0.716712422
NETO1	0.937622815	3.21537066	0.403813056	25.60246213	0.269884691
NETO2	0.432698188	1.823502308	0.680866989	4.883715497	0.232001786
NEU1	0.285010555	0.167756194	2.90E-05	969.7050858	0.686257717
NEU2	0.704477005	0.923363677	0.412591704	2.066450856	0.846183164
NEU3	0.499902432	0.760277443	0.416324373	1.388392868	0.372400173
NEU4	0.090360905	1.793044373	0.809893145	3.969669508	0.149872213
NEURL2	0.894497305	1.337302922	0.493653646	3.6227406	0.567572463
NEUROD1	0.647581614	0.152088151	0.000636816	36.32256232	0.500247972
NEUROD2	0.745354673	1.18496161	0.736478868	1.906550315	0.484299461
NEUROD4	0.916294705	0.542713641	0.043913508	6.707232171	0.633778967
NEUROD6	0.42001989	1.875270203	0.304854265	11.53547364	0.497552658
NEUROG1	0.550694598	0.867033162	0.018653167	40.30128095	0.941932068
NEUROG2	0.408587062	1.710613965	0.473723523	6.177020976	0.412507164
NEUROG3	0.971025967	0.994379583	0.791050663	1.249971464	0.96148438
NEXN	0.572263296	1.030800276	0.549996791	1.931918924	0.924593659
NF1	0.658302324	0.758161533	0.218121544	2.635268853	0.663159796
NF2	0.540451794	1.170857738	3.03E-08	45174382.42	0.985879594
NFAM1	0.994345912	1.182253746	0.597971919	2.337440729	0.63022851
NFASC	0.919025354	1.044917532	0.814195882	1.341019616	0.729967821
NFAT5	0.268199717	0.066153846	0.003651425	1.198526948	0.066145005
NFATC1	0.200320872	6.235743096	0.154319197	251.9744324	0.332146394
NFATC2	0.967233507	1.770518656	0.592483283	5.290843477	0.306398463
NFATC2IP	0.854764359	0.157162659	0.015968033	1.546846816	0.112722644
NFATC3	0.275231853	1.495739754	0.887689618	2.520292415	0.130421573
NFATC4	0.187547344	1.99618023	0.77580311	5.136271639	0.151712804
NFE2	0.863174415	0.854987858	0.537383856	1.36030182	0.508457597
NFE2L1	0.173699685	1.294762954	0.511813703	3.275432247	0.585393244
NFE2L2	0.578730958	0.988113383	0.829812075	1.176613461	0.893218004

NFE2L3	0.147531202	0.865778333	0.530844004	1.412038407	0.56361253
NFIA	0.112231588	0.282557365	0.03693699	2.16148267	0.223425529
NFIB	0.803782364	0.040160299	0.000168394	9.57782308	0.249725332
NFIC	0.954298017	0.626230369	0.200867581	1.952353252	0.419809293
NFIL3	0.989613816	2.407343705	1.03E-05	564896.076	0.889257235
NFIX	0.395294554	0.333522514	0.091481394	1.215955095	0.096171138
NFKB1	0.850672079	0.999170332	0.668236987	1.493992956	0.996773433
NFKB2	0.458299368	1.027248493	0.795404425	1.32667035	0.836794022
NFKBIA	0.731733069	1.625307783	0.00037307	7080.772505	0.909550676
NFKBIB	0.987389612	1.505080151	0.677460073	3.343763497	0.315451128
NFKBIE	0.503823995	6800.562193	0.286868661	161215400.7	0.08597866
NFKBIL1	0.202643589	0.27488745	0.040435802	1.868717979	0.186641404
NFKBIZ	0.396465656	1.927784069	0.001135234	3273.644645	0.862671184
NFRKB	0.382157746	3.170428824	0.283546893	35.44958233	0.348888484
NFS1	0.495529623	0.88410088	0.358524339	2.180143107	0.789086179
NFX1	0.962962483	1.228513383	0.583315354	2.587357114	0.588122837
NFXL1	0.365542744	0.892888717	0.73101462	1.09060782	0.266957664
NFYA	0.221550548	0.658909183	0.031889856	13.61440191	0.787161034
NFYB	0.561924427	0.08753331	0.004625229	1.65658401	0.104477962
NFYC	0.469622494	0.352180593	0.009210012	13.46699377	0.574565794
NGB	0.989201285	2.141404035	0.182491771	25.12776998	0.544472875
NGEF	0.540500397	3.005522513	0.001969045	4587.586597	0.768587818
NGFR	0.502828499	0.57265106	0.124081753	2.6428482	0.474947281
NGFRAP1	0.642464258	1.046603613	0.432640989	2.531843144	0.919502155
NGLY1	0.247918126	0.70841792	0.384138863	1.306444097	0.269621722
NGRN	0.629083841	1.486533576	0.391626773	5.642571516	0.560215251
NHLH1	0.592636556	1.653174101	0.60935922	4.485013958	0.323545199
NHLH2	0.234412077	1.709512788	0.486429946	6.007923644	0.403063068
NHLRC1	0.408500509	1.165410279	0.004152532	327.0730336	0.957555074
NHLRC2	0.708547727	0.309581513	0.021605058	4.43603127	0.388020914
NHP2L1	0.105572271	0.097968559	1.15E-05	833.6947865	0.614841953
NHS	0.889451809	0.924214553	0.082455305	10.3592187	0.94903653
NICN1	0.330587809	0.62476165	0.240313051	1.624244368	0.334571032

NID1	0.439146108	1.270503489	0.00163389	987.9362415	0.943798219
NID2	0.01804926	0.712964466	0.526529821	0.965412233	0.028701387
NIF3L1	0.296989619	1.093540436	0.871343119	1.372399299	0.440351826
NIN	0.061677469	0.508643557	0.226899792	1.140231401	0.100727567
NINJ1	0.691685585	0.029509456	0.000476246	1.828482769	0.094262723
NINJ2	0.718914104	0.704149258	0.324292013	1.528949706	0.375249214
NIP7	0.64451877	1.040046201	0.424598688	2.547572875	0.931543685
NIPA1	0.57439043	0.995784038	0.324343437	3.05720954	0.994110064
NIPA2	0.068164953	0.588063139	0.064073634	5.397200577	0.638777496
NIPBL	0.30027504	0.015925597	0.000118423	2.141681456	0.097839075
NIPSNAP1	0.612715322	1.225384593	0.528979512	2.838611638	0.635344239
NIPSNAP3A	0.42334764	0.963923774	0.491572319	1.890157371	0.914834999
NIPSNAP3B	0.706006367	0.013169195	2.81E-05	6.168449124	0.167569922
NISCH	0.914079954	0.925724318	0.050849867	16.85285665	0.958424531
NIT1	0.779402685	1.278934225	0.264310147	6.18845991	0.759727048
NIT2	0.58980931	3.627697309	0.011461296	1148.228575	0.660898523
NKAP	0.594841617	0.794151957	0.195379316	3.227963657	0.747354104
NKD1	0.202487826	0.000423669	3.31E-08	5.42867902	0.107527508
NKD2	0.077708515	1.626816533	0.887370494	2.98244313	0.11558732
NKG7	0.960732418	1.108885943	0.681421298	1.804504844	0.677393893
NKIRAS1	0.438826231	11.02515952	0.00508617	23898.95241	0.540258447
NKIRAS2	0.300324273	1.700924577	0.751153285	3.85160323	0.202743463
NKPD1	0.789624699	1.587217016	0.305920596	8.23500571	0.582343579
NKRF	0.179431854	0.651319621	0.032917777	12.88717797	0.778308965
NKTR	0.986590251	0.798227945	0.281541227	2.263142276	0.671675897
NKX1-1	0.561635423	1.049852962	0.149933176	7.351216516	0.960924246
NKX2-2	0.996105888	0.968071185	0.635289493	1.475172862	0.879984691
NKX2-3	0.817783671	22.08342912	0.187066653	2606.973679	0.203604919
NKX2-5	0.74757996	0.233464757	0.0013616	40.03070754	0.579415836
NKX2-6	0.949255926	0.783905397	0.261773588	2.347477749	0.663512945
NKX2-8	0.587625598	54.61754564	0.097991372	30442.23405	0.214990275
NKX3-1	0.228918949	0.908514482	0.106189875	7.772855615	0.930191981
NKX6-1	0.662193466	0.577567974	0.096558232	3.454752192	0.547510584

NKX6-2	0.2021638	3.886392645	0.020887378	723.1184204	0.610680605
NLE1	0.983292196	1.016697326	0.740007296	1.396842245	0.918618838
NLGN1	0.179832055	0.546815422	0.183410713	1.630259765	0.278780271
NLGN2	0.575535969	1.48083991	0.66652737	3.290017693	0.335074159
NLGN3	0.523838497	8.392885937	4.71E-05	1494121.683	0.730177539
NLGN4X	0.729754102	0.506590397	0.031221601	8.219752404	0.632423629
NLGN4Y	0.689081365	0.880249183	0.379144102	2.043652059	0.766616836
NLK	0.679454788	0.750411347	0.24040953	2.342324738	0.621019157
NLN	0.701441124	1.671226397	0.494429874	5.648925801	0.408541957
NMB	0.481372159	2.09368037	0.111890453	39.1766891	0.621002637
NMBR	0.172827609	0.149471062	0.002786494	8.017818088	0.349562872
NMD3	0.268563596	1.197265382	0.806961087	1.776348844	0.37108962
NME1	0.987182043	0.765751142	0.26697076	2.19640088	0.619582178
NME1-NME2	0.096418551	1.479285901	0.992328223	2.205204617	0.054585859
NME2	0.686576193	269.9882259	0.031236132	2333632.14	0.226088261
NME3	0.105393134	0.607683501	0.302362647	1.221312362	0.16193317
NME4	0.966068509	0.678063503	0.149831155	3.068588196	0.613996457
NME5	0.426378051	12.95465151	0.000598849	280242.3764	0.615004005
NME6	0.913150851	0.992960248	0.712088236	1.384617811	0.966781796
NME7	0.241856597	7.90E-05	6.37E-10	9.792810324	0.114414275
NMI	0.464025383	0.170434251	0.006935213	4.188455959	0.278740592
NMNAT1	0.351614612	0.084066798	0.000412043	17.15166799	0.361479931
NMNAT2	0.930322449	1.115730229	0.434012602	2.868243771	0.820174556
NMNAT3	0.596392494	0.808886999	0.407569275	1.605366788	0.544205811
NMS	0.54693702	1.323653514	0.002685649	652.3780555	0.929370931
NMT1	0.341511701	1.110088325	0.384647125	3.203705444	0.846853155
NMT2	0.315636482	0.968645531	0.372841944	2.516546699	0.947857684
NMU	0.970633944	0.930614019	0.784714219	1.103640575	0.408509155
NMUR1	0.380017239	0.97166559	0.415070052	2.274637771	0.947191441
NMUR2	0.095070227	1.13346148	0.836095425	1.536588872	0.419712219
NNAT	0.38129504	0.331974995	0.029530682	3.731962489	0.371743368
NNMT	0.804714568	24.01729885	0.006769291	85212.85754	0.445943648
NNT	0.278512808	2.61534284	0.514175463	13.30288718	0.246685079

NOBOX	0.149701747	0.668493842	0.147940321	3.020704653	0.60072769
NOC2L	0.11313724	0.030178359	0.000886243	1.027634003	0.051796663
NOC3L	0.090360905	1.322107016	0.982581548	1.77895358	0.065194147
NOC4L	0.081310981	0.023322441	0.000169887	3.201744102	0.134503079
NODAL	0.41254854	0.91368814	0.373460361	2.235380527	0.843244529
NOG	0.870877001	0.681458286	0.254173393	1.827041728	0.445946561
NOL10	0.98194481	1.034296565	0.833222023	1.283894754	0.759805572
NOL11	0.560032794	0.771658844	0.182132229	3.269368502	0.724928303
NOL3	0.192732601	1.376892155	0.763025792	2.48462375	0.288264408
NOL4	0.066578852	1.985813557	0.417110046	9.454232807	0.388864746
NOL6	0.70498565	1.10449939	0.582754093	2.093368225	0.76061208
NOL7	0.654559038	1.274836247	0.535635625	3.034166103	0.583111706
NOL8	0.737818266	0.966419662	0.744915881	1.253788498	0.797052178
NOL9	0.680409914	0.637667449	0.204776362	1.985677302	0.43753807
NOLC1	0.232971518	1.754457935	0.001046839	2940.396877	0.882019566
NOM1	0.243217375	0.720576697	0.330577098	1.570679816	0.409781884
NOMO1	0.512775492	0.537156522	0.102287354	2.820848488	0.462688257
NOMO2	0.384572836	0.735746559	0.244437602	2.214565166	0.585189518
NOMO3	0.266674764	1.196670219	0.916207956	1.562985349	0.187604749
NONO	0.163985529	0.626139682	0.214147291	1.830753497	0.392405706
NOS1	0.961828904	1.036066338	0.604278899	1.776387458	0.897514104
NOS1AP	0.355724807	0.899171946	0.293885648	2.751104701	0.852230048
NOS3	0.418223775	0.554926066	0.181710012	1.694694397	0.301185762
NOSIP	0.963928765	0.808173947	0.119001675	5.488537274	0.827502458
NOSTRIN	0.307093759	0.298781754	0.073189967	1.219710037	0.092331315
NOTCH1	0.85061598	1.07270859	0.584983694	1.967069732	0.820525844
NOTCH2	0.532681263	245.9695918	0.001234875	48993646.22	0.376543727
NOTCH2NL	0.401428938	0.614130229	0.223624706	1.686557564	0.344203057
NOTCH3	0.118176027	1.725444397	0.846882818	3.515431303	0.133027986
NOTCH4	0.381249001	2.831173106	0.653673995	12.26229163	0.164073024
NOTUM	0.439085735	1.901408627	0.516920846	6.994020069	0.333550279
NOV	0.97042727	0.764241255	0.154153769	3.788844743	0.742027254
NOVA1	0.361060448	0.811255499	0.397628606	1.655151252	0.565331246

NOVA2	0.337134231	3.262752361	0.641156918	16.60366234	0.154290942
NOX1	0.210051636	0.020159125	3.14E-05	12.94065174	0.236538175
NOX3	0.277449177	1.11414143	0.874280216	1.419809237	0.382231173
NOX4	0.654488419	2.459328737	0.30248848	19.99513451	0.399987433
NOX5	0.357348713	0.916239576	0.694529475	1.208724744	0.53600489
NOXA1	0.208587917	0.744105103	0.436211298	1.26932156	0.278035995
NOXO1	0.730087738	0.170662922	1.03E-05	2835.660595	0.721401233
NPAS1	0.639571317	1.236783761	0.50235411	3.044931935	0.643862597
NPAS2	0.951352978	1.039419644	0.578761272	1.866733744	0.897027817
NPAS3	0.415342626	0.325304525	0.017766312	5.95638723	0.449033082
NPAS4	0.133127001	0.778021129	0.546656904	1.107306745	0.163346311
NPAT	0.990092769	0.916944194	0.727564684	1.155617738	0.462581094
NPB	0.309549611	51.76229329	0.446282425	6003.675822	0.10367334
NPBWR1	0.980719473	0.702389111	0.055539877	8.882815219	0.784948467
NPBWR2	0.374157823	0.145421728	0.000128676	164.3466822	0.590886513
NPC1	0.891891044	1.627865914	0.324656157	8.162319975	0.553612041
NPC1L1	0.416529027	0.63003354	0.175466064	2.26221671	0.478744785
NPC2	0.002741643	0.611409875	0.418744999	0.89272	0.010846857
NPDC1	0.156243218	0.430884046	0.122969524	1.509813604	0.188176744
NPEPL1	0.103192407	1.773445532	0.230456601	13.6472943	0.58212726
NPEPPS	0.486355542	0.702170043	0.266534354	1.849828221	0.474352247
NPFFR1	0.747916726	21.90482	0.000304594	1575283.363	0.588525971
NPFFR2	0.854464814	1.802448089	0.383317711	8.475525696	0.455718532
NPHP1	0.672764649	1.010999702	0.702079002	1.455848124	0.953111517
NPHP3	0.78817745	0.666181737	0.264277573	1.679287805	0.38919346
NPHP4	0.974674289	0.667138212	0.232125645	1.917381403	0.452386693
NPHS1	0.147992289	7.114935269	0.24438654	207.1403112	0.253957051
NPHS2	0.753074577	1.186983395	0.441676709	3.18995671	0.733973751
NPL	0.812442781	2.232854852	0.046885362	106.3368315	0.683622834
NPLOC4	0.789776665	1.557282292	0.297166529	8.16083879	0.60019682
NPM1	0.129032964	0.030341132	4.49E-06	205.1190925	0.437270768
NPM2	0.133352069	1.353825472	0.934358472	1.961606241	0.109351586
NPM3	0.990966317	0.079203868	0.001342025	4.674467431	0.222932978

NPNT	0.78357959	1.033041578	0.422002482	2.528835607	0.943263915
NPPA	0.333491568	0.626823136	0.103138184	3.809522593	0.611940315
NPPB	0.140403432	0.99947742	0.437246732	2.284648556	0.999011247
NPPC	0.217454901	2.996407506	0.835307594	10.74868468	0.09221161
NPR1	0.993309312	1.059084528	0.820683464	1.366738927	0.659080995
NPR2	0.309383804	0.596860638	0.201487976	1.76805896	0.351635118
NPR3	0.544612674	1.215960638	0.307782093	4.803919093	0.780287857
NPTX1	0.393096052	0.711789911	0.001527388	331.7067868	0.913639679
NPTX2	0.796097863	1.134853389	0.093358824	13.79507757	0.920928726
NPTXR	0.179215635	1.746498682	0.598317999	5.098054304	0.307626375
NPY	0.053720335	1.22782396	0.905478521	1.664922625	0.186524559
NPY1R	0.598799654	0.960540897	0.505886277	1.823806765	0.90205779
NPY2R	0.768425983	4.233234742	0.529415489	33.84917282	0.17370929
NPY5R	0.908892611	60.01280464	0.000310279	11607410.81	0.509714043
NQO1	0.690108315	1.25557811	0.900398569	1.750865055	0.179745657
NQO2	0.934731854	1.037247126	0.000142677	7540.673386	0.993568148
NR0B1	0.904562843	0.950600911	0.249441547	3.622660718	0.94083714
NR0B2	0.540449318	0.03804585	0.000200167	7.231404481	0.222087528
NR1D1	0.444411641	5.217997347	0.04426608	615.0871377	0.497205511
NR1D2	0.354204089	0.068731316	0.001285551	3.674684421	0.187204569
NR1H2	0.919014117	1.232889524	0.594784171	2.555576718	0.573473338
NR1H3	0.60822114	1.351847529	0.315868614	5.785607235	0.684444056
NR1H4	0.220356527	1.352891003	0.442989293	4.13173432	0.595698027
NR1I2	0.373122307	0.364977825	0.067148233	1.983802212	0.243250843
NR1I3	0.69857287	1.058291295	0.829260293	1.350577706	0.648876125
NR2C1	0.174496337	1.122179819	0.865919228	1.454278304	0.38346803
NR2C2	0.687689529	1.018820571	0.741949174	1.399011403	0.90825519
NR2E1	0.243050862	0.70629642	0.382963095	1.302618033	0.265528893
NR2E3	0.362683313	1.265251901	0.028648704	55.8790499	0.903108703
NR2F1	0.221746341	1.751367456	0.741511196	4.13653628	0.201263621
NR2F2	0.87596053	1.285203155	0.528114257	3.127632184	0.580284865
NR2F6	0.401441096	1.019431533	0.69735397	1.49026276	0.920868987
NR3C1	0.529927249	1.087910749	0.512589705	2.308961313	0.826299299

NR3C2	0.917244417	0.993111234	0.674508936	1.462204382	0.97206236
NR4A1	0.107884176	56.6067446	0.012450953	257355.6772	0.347588915
NR4A2	0.077420122	1.321104254	0.091583985	19.05700478	0.837967643
NR4A3	0.259478456	0.633466967	0.334761724	1.198704542	0.160617422
NR5A1	0.089254341	0.737779292	0.264241859	2.059924521	0.561577478
NR5A2	0.365806285	1.100596914	0.897871272	1.349094914	0.356103699
NR6A1	0.765612294	1.139117511	0.220250685	5.891417328	0.876538159
NRAP	0.075500723	0.46432409	0.18602098	1.158992177	0.10021565
NRAS	0.120528086	0.54006135	0.160237613	1.820210975	0.320324981
NRBF2	0.152243429	2.510694221	0.406552622	15.50496819	0.321672901
NRBP1	0.654827577	0.837155317	0.314293467	2.229855526	0.722140134
NRBP2	0.496834077	0.82475979	0.340237177	1.999278022	0.66976935
NRCAM	0.495993703	0.115830303	0.004325366	3.101854891	0.198754913
NRD1	0.908146918	0.08018486	0.001097967	5.855925846	0.249060128
NRF1	0.329650966	0.825081509	0.599234421	1.13604872	0.238685155
NRG1	0.461213758	1.900945548	0.00774789	466.3971731	0.819028455
NRG2	0.292020254	1.100420316	0.977908334	1.238280553	0.112058191
NRG3	0.119149171	0.332504913	0.078643706	1.405827915	0.134419929
NRG4	0.780952865	1.404804886	0.572872891	3.444877215	0.45766717
NRGN	0.433545329	0.107186948	0.010155254	1.131339713	0.063264156
NRIP1	0.59006426	0.759005336	0.271335876	2.123158602	0.599305336
NRIP2	0.111952052	1.732892266	0.781972071	3.84018268	0.175673692
NRIP3	0.99094045	1.081158416	0.308283372	3.791652833	0.902987255
NRK	0.812346884	1.035134434	0.456186361	2.348827996	0.934170667
NRL	0.594550661	0.973028661	0.68281083	1.386598943	0.879741251
NRM	0.066609967	2.748784616	0.928408612	8.138460554	0.06787559
NRN1	0.19391014	1.176978231	0.95979068	1.443312366	0.117431468
NRP1	0.412378575	0.951692195	0.356431154	2.541074267	0.921286251
NRP2	0.718907518	1.288685446	0.432107744	3.843277983	0.649167552
NRTN	0.732822389	1.000448359	0.76712313	1.304740896	0.99736027
NRXN1	0.419695333	1.161838012	0.351801311	3.837016872	0.805613075
NRXN2	0.949952217	1.078750685	0.069331441	16.78463669	0.956830471
NRXN3	0.211131642	0.633167243	0.184568455	2.172097926	0.467444203

NSD1	0.41943846	111.1766928	0.209128655	59103.60303	0.141216184
NSDHL	0.103109124	0.564410067	0.103485537	3.078292216	0.50870227
NSF	0.81412244	1.435701299	0.004931938	417.9367316	0.900576833
NSFL1C	0.804121657	1.577824673	0.364983755	6.820935638	0.5414878
NSMAF	0.780529455	0.83827368	0.542623402	1.295010058	0.426627051
NSMCE1	0.796379446	1.142238197	0.402243593	3.243577076	0.802785054
NSUN2	0.871311585	0.006776293	1.71E-06	26.84593732	0.23737387
NSUN3	0.126422532	0.607264625	0.318393415	1.158222213	0.130003504
NSUN4	0.754914692	0.933414906	0.000475084	1833.915345	0.985790744
NSUN5	0.395464748	0.313908322	0.026629208	3.700389311	0.357319534
NSUN6	0.477735398	0.860010733	0.395532068	1.869932986	0.703532566
NT5C	0.223625242	1.547688263	0.819508051	2.922898629	0.178184035
NT5C1A	0.850654182	0.723545669	0.249005694	2.102435196	0.552126375
NT5C1B	0.292710748	0.001963514	8.05E-08	47.90532151	0.226553399
NT5C2	0.241170763	0.873497005	0.597274185	1.277465255	0.485577685
NT5DC1	0.449484734	0.029514518	2.64E-05	32.99470102	0.32527022
NT5DC2	0.763968442	2.894589527	5.71E-06	1468108.567	0.874004231
NT5DC3	0.192918879	0.002877973	5.74E-06	1.442575396	0.065118235
NT5E	0.421343044	0.001910718	2.04E-07	17.85869231	0.179584533
NT5M	0.41525721	1.352120275	0.714240436	2.559683194	0.354211521
NTAN1	0.701704155	1.124370754	0.297049178	4.255893251	0.86296034
NTF3	0.207488344	0.348277965	0.112750391	1.075805946	0.066805429
NTHL1	0.157405521	1.347068168	0.79899228	2.271101605	0.263597177
NTN1	0.842007742	0.885626659	0.127202315	6.166040153	0.902362851
NTN4	0.837251273	1.094908283	0.479793895	2.498623179	0.829463791
NTNG1	0.663603526	1.138817324	0.438628123	2.956729926	0.789442297
NTNG2	0.734174636	0.966037651	0.278928547	3.345762754	0.956524543
NTRK1	0.815300404	0.671173503	0.056070095	8.034119959	0.752906071
NTRK2	0.247794459	31.50788551	0.634008352	1565.826137	0.083398108
NTRK3	0.563099801	2.189942993	0.458952989	10.44954587	0.325528588
NTS	0.398830447	3.988471868	0.037250466	427.052589	0.561799078
NTSR1	0.483805447	0.35776498	0.090325936	1.417043509	0.143297084
NTSR2	0.248687231	0.43304785	0.155728236	1.204216042	0.108748665

NUAK1	0.349604062	0.8875915	0.679893097	1.158739035	0.380637229
NUAK2	0.488122274	0.498268134	0.194207971	1.278377668	0.147312805
NUBP1	0.03691432	0.401123052	0.160296419	1.003763552	0.050945669
NUBP2	0.113225002	0.134118066	0.017250638	1.042724089	0.054860193
NUBPL	0.884843341	0.988602265	0.639201469	1.528992793	0.958909369
NUCB1	0.83777475	135.4759206	0.810612807	22641.79013	0.060166864
NUCB2	0.947096187	0.737410793	0.000511276	1063.563458	0.934585743
NUCKS1	0.09950164	0.564138222	0.150932469	2.108571701	0.394778674
NUDC	0.282660539	1.415076966	0.004726026	423.7053854	0.905004789
NUDCD1	0.71169812	0.909678579	0.589719053	1.403236188	0.668611747
NUDCD2	0.237092229	59.3938476	2.90E-05	121802889.2	0.581785342
NUDCD3	0.395573752	3.926653293	0.618153263	24.94301497	0.147051729
NUDT1	0.805737391	0.886927222	0.296230586	2.655498562	0.830189397
NUDT10	0.382543801	0.062005439	0.000552676	6.956472653	0.248272029
NUDT11	0.208883293	0.851924857	0.366539557	1.980075409	0.709578533
NUDT12	0.12709926	1.698917127	0.890163181	3.242461006	0.108022967
NUDT13	0.58029204	1.415959556	0.344261125	5.823897374	0.629773881
NUDT14	0.898090783	0.210372891	0.004579483	9.664136885	0.424695471
NUDT15	0.501027859	0.850758461	0.02889093	25.05249803	0.92538658
NUDT16	0.977056853	0.188909507	5.76E-05	619.2472022	0.686587187
NUDT16L1	0.810657124	0.798676418	0.3618279	1.762948686	0.577895721
NUDT17	0.93123409	4.467778218	0.403816915	49.43092147	0.222250886
NUDT18	0.572730696	0.018902918	3.04E-05	11.74863438	0.226573462
NUDT19	0.26119564	0.439693096	0.05416671	3.569166712	0.441846296
NUDT2	0.961145881	23.54025607	0.034052296	16273.31236	0.343721695
NUDT21	0.697963449	0.017261393	6.59E-05	4.520628725	0.153031562
NUDT22	0.717709399	1.119113928	0.68359996	1.832089024	0.654532542
NUDT3	0.299559104	0.446410884	0.04062176	4.905810992	0.509584902
NUDT4	0.371388864	16.2128988	0.003416003	76949.01437	0.518919694
NUDT4P1	0.375571115	0.579332067	0.318840058	1.052645785	0.073201038
NUDT5	0.767213032	0.990928954	0.86115088	1.140264981	0.898756219
NUDT6	0.999961328	133.5670158	0.489026617	36480.93398	0.087257766
NUDT8	0.286105541	1.742104505	0.459693259	6.602072245	0.414150098

NUDT9	0.471095618	1.844059583	1.61E-05	211689.7503	0.918004213
NUFIP1	0.272384804	1.403957667	0.941704788	2.093115757	0.095875684
NUFIP2	0.267470846	0.887953324	0.26744063	2.948172487	0.846103919
NUMA1	0.852655615	0.912876123	0.132825118	6.273985131	0.926152099
NUMB	0.087690224	0.00365844	3.31E-06	4.040804562	0.116562552
NUMBL	0.234531294	0.660676205	0.351183704	1.242919425	0.198611615
NUP107	0.52221016	1.337839783	0.372421812	4.805882004	0.655528413
NUP133	0.479796348	0.953511755	0.256859522	3.539618317	0.943290959
NUP153	0.355010504	3.333925957	0.452674363	24.55421202	0.237214931
NUP155	0.418594739	1.387554222	0.641027111	3.00347159	0.405789423
NUP160	0.83632537	0.634926724	0.001426342	282.6333822	0.883929243
NUP188	0.453795158	0.36623502	0.025895952	5.179500207	0.457390833
NUP205	0.44968919	1.104909843	0.306616196	3.98160886	0.878767859
NUP210	0.722240505	0.293228674	0.003995616	21.51935106	0.575659527
NUP210L	0.124115543	0.583968189	0.235678065	1.446968966	0.245277314
NUP214	0.437949388	0.465185717	0.154298952	1.402457686	0.174066344
NUP35	0.675641852	1.421621791	0.282946195	7.142730861	0.6692858
NUP37	0.34421178	1.139053576	0.827722521	1.567485499	0.424141318
NUP43	0.73579382	439439.8557	0.197859665	9.75982E+11	0.081392624
NUP50	0.939330288	1.270438326	0.208356228	7.746413685	0.795249861
NUP54	0.123680646	7.387293819	0.188077522	290.1575346	0.285619709
NUP62	0.453328909	2.490928935	0.679820411	9.127008924	0.168363624
NUP85	0.471462134	0.900017293	0.198764173	4.075337698	0.891263825
NUP88	0.64866801	0.366582059	0.000490589	273.9208014	0.766255833
NUP93	0.09264669	0.292292004	0.068338712	1.250164268	0.097147252
NUP98	0.829852726	1.454326374	0.748532319	2.825616407	0.269050374
NUPL1	0.503620634	1.562980172	0.494621108	4.938946156	0.446794894
NUPL2	0.98115181	1.106798182	0.534725103	2.290900895	0.784557582
NUSAP1	0.22176219	0.914706583	0.761870117	1.09820311	0.339205605
NUTF2	0.063048273	1.523503899	0.696768643	3.33118339	0.291525869
NVL	0.888057411	3.406359807	0.000135235	85801.16597	0.812624143
NXF1	0.17232116	1.882890921	0.239092268	14.82807558	0.547843419
NXF2	0.78756419	12.48019154	0.024618104	6326.855175	0.427020983

NXF3	0.950174507	0.729713344	0.250204468	2.128185675	0.563948224
NXF4	0.453445502	0.717475639	0.174420466	2.951323919	0.645427053
NXF5	0.223723051	0.95293487	0.474903739	1.912145117	0.892079785
NXN	0.066618182	1.33179196	0.947614403	1.871721049	0.098924911
NXPH1	0.443417878	0.220466885	1.24E-05	3932.235204	0.762090528
NXPH2	0.585477092	0.921514179	0.758383037	1.119735466	0.410925345
NXPH3	0.212318143	0.000637523	6.81E-08	5.970076747	0.114793054
NXPH4	0.455482882	0.532191313	0.146949423	1.927381457	0.33673709
NXT1	0.246314293	0.132740658	1.42E-05	1239.116807	0.665047021
NXT2	0.406171092	1.765058048	1.148057082	2.71365419	0.009621745
NYX	0.260516805	0.149338144	0.001122296	19.87165466	0.446043323
OAF	0.285932336	0.651782328	0.345367684	1.230051979	0.186509996
OAS1	0.53812899	1.067951771	0.668280047	1.706651264	0.78342163
OAS2	0.543619409	0.206300428	0.022005316	1.934072066	0.166880415
OAS3	0.419169255	2.102064337	0.017517022	252.2503261	0.761017204
OASL	0.597867213	1.066825324	0.837161696	1.359493963	0.600985007
OAT	0.876605256	0.908324322	0.367218177	2.246765348	0.835156791
OAZ1	0.327167124	0.768688922	0.45004889	1.31292993	0.33547003
OAZ2	0.126686916	0.527512882	0.078232997	3.556936973	0.511287
OAZ3	0.464707924	244.8937528	0.012998381	4613878.553	0.273405114
OBFC1	0.65191514	1.206563541	0.379955674	3.831487931	0.750095641
OBP2A	0.41862419	1.065158896	0.971284434	1.168106308	0.179919754
OBP2B	0.619201996	2193.902927	0.318137961	15129316.94	0.08800755
OBSCN	0.838778932	0.004116794	7.37E-09	2299.423498	0.415916983
OBSL1	0.735712725	0.929784937	0.403151184	2.144356917	0.864417124
OCA2	0.18914833	80.82037215	0.293747727	22236.53821	0.125392285
OCIAD1	0.170354964	4.433515378	0.007923111	2480.851097	0.644578459
OCIAD2	0.717757422	0.918238575	0.38011744	2.218162052	0.84965969
OCLM	0.957984586	1.04368412	0.581827074	1.872165442	0.885963819
OCLN	0.737798164	0.891251406	0.601924474	1.319649064	0.5653537
OCM	0.917886862	1.290334261	0.451262058	3.689569013	0.634408182
OCRL	0.147112743	1.586881543	0.125210366	20.1116977	0.721551247
ODC1	0.827932397	0.983361795	0.839733461	1.151556374	0.83501651

ODF1	0.84685788	0.651821237	0.026829124	15.83618335	0.792601123
ODF2	0.086595413	0.847495792	0.136651568	5.256062028	0.858942245
ODF2L	0.064499748	96.093585	0.552749717	16705.53018	0.082794441
ODF3	0.681079754	0.84603693	0.387562856	1.846870709	0.674669677
ODF3L1	0.257988478	0.315237571	0.003288604	30.21790206	0.619978134
ODF4	0.288632898	1.04959011	0.822470243	1.339427669	0.697255231
OFCC1	0.28243718	2.317578167	0.8939693	6.008224847	0.083745703
OFD1	0.609268484	0.995367992	0.808228342	1.225838527	0.965149843
OGDH	0.735756421	1.074573898	0.423532921	2.726373807	0.879653279
OGDHL	0.698369461	0.112347296	0.001174397	10.74756942	0.347486876
OGFOD1	0.459493405	0.626291219	0.246137147	1.593585917	0.326084407
OGFOD2	0.818981233	1.073260282	0.583498107	1.974106889	0.820124916
OGFR	0.68214034	0.612916804	0.31613738	1.188303039	0.147278696
OGFRL1	0.146600873	0.000146308	8.23E-09	2.600727417	0.076972727
OGG1	0.891548434	0.841986523	0.238821448	2.968499325	0.7890635
OGN	0.235897503	330047.481	0.024415002	4.46166E+12	0.12931605
OGT	0.413836677	0.911733207	0.430841193	1.929382462	0.80907892
OIP5	0.959856628	0.463296989	0.10446226	2.054752599	0.311360309
OIT3	0.66695665	1.927707985	0.007142487	520.2751083	0.818252714
OLFM1	0.96739421	1.27926989	0.559674538	2.924077013	0.559275659
OLFM3	0.968658231	1.91762665	0.746502502	4.926027657	0.176181271
OLFM4	0.16089548	191.7968191	0.059990789	613194.4704	0.201731977
OLFML1	0.411886463	0.285566054	0.053312194	1.529630752	0.143300762
OLFML2A	0.137384452	0.83211761	0.5227211	1.324644668	0.438482461
OLFML2B	0.182751904	0.002378646	6.89E-08	82.07244705	0.257131432
OLFML3	0.135343452	1.380595665	0.670811817	2.841399542	0.38115272
OLIG1	0.321967348	37.67267884	9.54E-06	148711137.2	0.639580915
OLIG2	0.577262208	1.905170112	0.619472546	5.859296234	0.260798029
OLIG3	0.891592522	1.397989498	0.548163852	3.565311047	0.483056907
OLR1	0.828326732	0.966114599	0.880141174	1.060486029	0.468482427
OMA1	0.477609931	1.032828961	0.608315593	1.753589213	0.904802785
OMD	0.368119861	0.387351546	0.088206299	1.701026145	0.209011026
OMG	0.495035311	0.339262087	2.40E-05	4796.024603	0.824547032

OMP	0.150018739	1.730349742	0.572536644	5.229552139	0.331203335
ONECUT1	0.223871944	0.986922681	0.008041628	121.1217954	0.995720248
ONECUT2	0.343440052	2.24736333	0.552657435	9.138829263	0.257887766
OPA1	0.492506489	0.791316628	0.54858708	1.141445048	0.210499649
OPA3	0.108606273	0.003147996	6.97E-06	1.422619752	0.064753857
OPCML	0.571423319	1.026120652	0.644735664	1.633108964	0.913397061
OPHN1	0.983051442	2.569790329	0.016209408	407.406761	0.71499705
OPLAH	0.705657596	1.76561496	0.665007858	4.687758422	0.253826469
OPN1LW	0.802412943	2.282296809	0.445357692	11.69594422	0.322291275
OPN1MW	0.851599006	0.647324032	0.092043953	4.552481605	0.662111058
OPN1SW	0.790462176	0.004673246	2.76E-08	790.8833084	0.382351617
OPN3	0.242500242	0.02294975	0.000187775	2.804907894	0.123721654
OPN4	0.402751778	179.9980069	0.010086503	3212142.277	0.29848687
OPN5	0.240427166	1.179421963	0.44242349	3.144128191	0.741497857
OPRD1	0.923824467	1.066627296	0.818722397	1.389596513	0.632692763
OPRK1	0.793658188	59.43803009	2.70E-05	130739258.5	0.583529612
OPRL1	0.569251076	0.927961236	0.407076352	2.115357601	0.858849497
OPRM1	0.77669076	1.038878171	0.699205251	1.543563714	0.850249463
OPTC	0.490543033	1.655236772	0.527660949	5.192366004	0.38761279
OPTN	0.52555819	1.47089539	0.379088998	5.707190818	0.57698215
OR10A2	0.582351834	1.028323338	0.795421521	1.329419509	0.831205499
OR10A3	0.494723632	1.177837895	0.495140986	2.801832498	0.711237763
OR10A4	0.090187871	0.040412325	0.000512971	3.183721644	0.149817265
OR10A5	0.394748377	1.089889088	0.610377541	1.94610408	0.771054672
OR10A6	0.99591487	0.838218416	0.385443017	1.822863773	0.656158137
OR10A7	0.861753155	0.435335395	0.053558538	3.538500368	0.436623643
OR10AD1	0.534156697	1.332643255	0.529656489	3.352999693	0.541868747
OR10AG1	0.453631036	0.619134607	0.347946034	1.101687112	0.102976198
OR10C1	0.13079871	0.573804041	0.232945907	1.413422892	0.22717269
OR10G2	0.703705743	0.026031857	0.000178449	3.797480854	0.151257266
OR10G3	0.921004967	18.70005104	0.000226463	1544144.348	0.612165436
OR10G4	0.081873349	2.59188695	0.882069085	7.616045129	0.083311578
OR10G7	0.334618148	1.010515265	0.852192261	1.198252023	0.904234354

OR10G8	0.691018247	1.160196527	0.926272951	1.453195821	0.19589179
OR10G9	0.437013693	0.755557981	6.05E-05	9434.027712	0.953554604
OR10H1	0.978791091	0.959775129	0.526522372	1.749533061	0.893383477
OR10H2	0.338207001	0.715876968	1.09E-05	46900.91073	0.952894674
OR10H3	0.414450686	1.138383841	0.597752833	2.167982648	0.693328666
OR10H4	0.278560387	1.784350687	0.01216618	261.701475	0.820015946
OR10H5	0.85740683	1.029556615	0.530024117	1.999884135	0.931478806
OR10J1	0.253620006	0.008917161	1.97E-06	40.38254313	0.271818867
OR10J3	0.282514907	0.253257838	0.043141008	1.486741645	0.128310708
OR10J5	0.342392202	1.699715875	0.158700849	18.20427599	0.661049705
OR10K1	0.729710462	1.079367354	0.853820376	1.364495294	0.523087358
OR10K2	0.176618842	0.038713669	4.83E-06	310.5642743	0.478388779
OR10P1	0.128099528	0.571096997	0.14789318	2.205319947	0.416410975
OR10Q1	0.169544244	1.364396724	0.865284437	2.151406336	0.181149014
OR10R2	0.766862842	0.906943313	0.016155318	50.91488553	0.962091348
OR10S1	0.335286653	0.25206519	0.056438921	1.125763196	0.07110309
OR10T2	0.736925791	0.863303701	0.387726721	1.922212838	0.718917249
OR10V1	0.497489537	1.02126868	0.359855422	2.898357656	0.968456336
OR10W1	0.7470565	33.5868347	0.006688102	168668.9969	0.418944917
OR10Z1	0.197525807	0.808386357	0.004638163	140.8938071	0.93561214
OR11A1	0.180275257	1.572686025	0.835797766	2.959258131	0.160366603
OR11G2	0.422776026	0.833410676	0.003031272	229.1359628	0.949295881
OR11H1	0.533827008	1.200528529	0.417716225	3.450353762	0.734381824
OR11H12	0.554031288	2.663259369	0.000485156	14619.94642	0.823560615
OR11H4	0.177751931	38.36975084	0.069568268	21162.49008	0.25746651
OR11H6	0.857522725	0.516260664	0.143600884	1.856012761	0.311206156
OR11L1	0.220025667	3.816948037	0.514571348	28.31306555	0.190161495
OR12D2	0.425832826	1.051954155	0.260203973	4.252846455	0.94334728
OR12D3	0.538180684	0.345874369	0.025848989	4.627998444	0.422414036
OR13A1	0.380634655	3.742334483	0.723674427	19.35271837	0.115444225
OR13C2	0.926381772	0.135964316	0.003722773	4.965732136	0.277049543
OR13C3	0.495899813	1.075484583	0.54967448	2.104276494	0.831718003
OR13C4	0.91453959	0.863957559	0.271704633	2.747184166	0.804321468

OR13C5	0.458500189	0.991842165	0.619204344	1.588733816	0.972815771
OR13C8	0.431852163	0.838449763	0.300299881	2.34098663	0.736613026
OR13C9	0.284472571	0.778183552	0.066980022	9.041048658	0.841151711
OR13D1	0.480103186	0.183718993	0.004536633	7.440025407	0.369594283
OR13F1	0.814344286	0.36276105	2.66E-05	4950.340958	0.834654093
OR13G1	0.277262008	0.597606127	0.271750097	1.31419671	0.20039675
OR13H1	0.680651973	0.684334956	0.262670779	1.782894669	0.437518491
OR13J1	0.161508292	0.642856279	0.244169834	1.692527653	0.371025822
OR1A1	0.382765972	0.491673651	0.155799419	1.551629528	0.225988669
OR1A2	0.640256666	1.126617946	0.490733688	2.586470072	0.778585352
OR1C1	0.493236696	1.350643851	0.711931477	2.562379767	0.357571036
OR1D2	0.877802921	1.857727406	0.000687077	5022.947037	0.877915165
OR1D4	0.111740645	1.551404207	0.91963934	2.617172744	0.099768072
OR1D5	0.43525041	1.298729415	0.68365068	2.467192885	0.424657404
OR1E1	0.682339071	1.6436621	0.267369631	10.10445761	0.59174707
OR1E2	0.213767404	0.536686566	0.184911515	1.557677307	0.252315567
OR1F1	0.735103452	511.7140496	0.000978394	267633835	0.353152251
OR1G1	0.347666745	1.376396169	0.117838951	16.07674201	0.798917556
OR1I1	0.312429996	0.410348106	0.112232839	1.500323519	0.17809321
OR1J1	0.762189085	1.239966945	0.700251251	2.195666232	0.460658545
OR1J2	0.361584524	0.679404303	0.256625187	1.798694087	0.436483235
OR1J4	0.135189443	2.041585422	0.819382144	5.086846313	0.12545009
OR1K1	0.412411123	2.156658625	0.645602305	7.204398727	0.211697939
OR1L1	0.577433792	1.420139156	0.585778347	3.44293235	0.437571913
OR1L3	0.56583576	0.708001346	0.277324322	1.807507909	0.470233315
OR1L4	0.827183672	0.673770625	0.015014791	30.23464432	0.838777538
OR1L6	0.937936724	0.593771241	0.069088222	5.103102592	0.634828094
OR1L8	0.233097703	0.387574358	0.096577519	1.555371118	0.181245416
OR1M1	0.679993381	1.207251278	0.680312564	2.1423324	0.519817411
OR1N1	0.964005265	0.218113394	0.000248163	191.7021698	0.659734611
OR1N2	0.957752735	3.000332504	0.74593909	12.06800295	0.121813038
OR1Q1	0.908475639	0.902581921	0.003635647	224.0740663	0.970940088
OR1S2	0.116279344	0.543305395	0.196043981	1.505686381	0.240770243

OR2A1	0.410584754	0.217100373	0.023347578	2.018734987	0.179427495
OR2A12	0.836673198	3.850550633	0.002531883	5856.012572	0.718364202
OR2A14	0.548817829	0.833399263	0.35633295	1.94917234	0.674195925
OR2A2	0.741616596	0.707756407	0.00091912	544.9984888	0.91881218
OR2A20P	0.773486985	1.393110252	0.914103482	2.12312524	0.123026327
OR2A25	0.935375476	1.844591871	0.409837352	8.30212071	0.425021011
OR2A42	0.22139789	1.125450628	0.808662072	1.566339218	0.483464446
OR2A5	0.064638818	5125.514215	0.225628356	116434372.4	0.095107643
OR2A7	0.60485265	0.322554462	0.006907733	15.0615815	0.563958494
OR2A9P	0.090346986	9.645583745	0.43695127	212.9237109	0.151126355
OR2AE1	0.54713355	0.782906085	0.095130421	6.443174875	0.819972168
OR2AG1	0.464075951	241.4812962	0.020982423	2779146.039	0.250125232
OR2AG2	0.12264774	3.671060666	0.176017829	76.56432582	0.401412722
OR2AK2	0.208080404	0.703728354	0.243104804	2.037119741	0.517046112
OR2AT4	0.98905028	0.522368975	0.158826017	1.718039346	0.285050835
OR2B11	0.292017934	2.365594854	0.530537855	10.54785999	0.258939573
OR2B2	0.857407131	1.473952306	0.636352904	3.414041778	0.365333724
OR2B3	0.307064195	1.08378522	0.842959407	1.393412771	0.530305911
OR2B6	0.066609967	1.753392271	0.866959009	3.546170493	0.118127226
OR2C1	0.290698722	0.537060712	0.139264025	2.071132209	0.366688984
OR2C3	0.420136425	0.664068997	0.277024583	1.591871838	0.358762555
OR2D2	0.97378824	0.121407869	0.000174851	84.29956101	0.527624767
OR2D3	0.167671724	0.767742631	0.216658215	2.720546486	0.682203084
OR2F1	0.257981352	0.616044669	0.002608562	145.4866749	0.862059593
OR2F2	0.198556209	0.43231749	0.116507663	1.604172695	0.210017462
OR2G2	0.263013982	0.505035173	0.097873609	2.606019421	0.414538968
OR2G3	0.667174788	0.646428043	0.011510654	36.30282176	0.831885615
OR2G6	0.129685666	0.123180222	0.003101632	4.892058443	0.264937013
OR2H1	0.84184827	0.004370036	3.44E-07	55.45229507	0.25974313
OR2H2	0.947308745	1.84697108	0.220103656	15.49861657	0.571862745
OR2J2	0.626205682	6260.490486	0.014721611	2662326987	0.186160085
OR2J3	0.086476094	21.24103172	0.000898539	502127.9446	0.552011401
OR2K2	0.608593352	18.11569234	0.008186118	40089.61196	0.461032475

OR2L13	0.886001309	17.61544262	0.260876362	1189.466982	0.181951681
OR2L1P	0.112451036	0.869921899	0.020043353	37.75636267	0.942254043
OR2L2	0.158973617	0.061457671	0.002240759	1.685609701	0.098751281
OR2L3	0.934365905	1.18066393	0.651725118	2.138888432	0.583832784
OR2M1P	0.196146066	3.069222492	0.011386248	827.3248905	0.694528091
OR2M2	0.051993202	5.977812832	0.990937177	36.06106128	0.051171936
OR2M3	0.217102352	0.169288935	0.010314901	2.778382885	0.213439965
OR2M4	0.379595051	1.279359582	0.484603834	3.377523713	0.618915403
OR2M5	0.865293592	0.580696089	0.021762232	15.49509902	0.745647094
OR2M7	0.234955697	0.529831511	0.193597171	1.450028572	0.216243658
OR2S2	0.966845086	0.449804559	0.020465244	9.886231614	0.612330901
OR2T1	0.569526528	0.649225219	0.023314376	18.07869044	0.799107205
OR2T10	0.500242724	0.911032291	0.490024898	1.693750337	0.768378982
OR2T11	0.374308272	0.955049798	0.760921156	1.198705161	0.691593116
OR2T12	0.701041971	0.842555209	0.427463451	1.660725095	0.620723899
OR2T2	0.489837292	1.036786114	0.19594811	5.485765831	0.966100753
OR2T27	0.565279173	0.807387396	0.131950314	4.940302036	0.816924864
OR2T29	0.998350672	0.436772893	0.086707696	2.200157181	0.315324325
OR2T3	0.140152645	1.094572415	0.582079524	2.058290528	0.779129548
OR2T33	0.88246348	0.064058803	0.000244718	16.76838574	0.333349475
OR2T34	0.60154597	0.590894814	0.057738248	6.047233738	0.657492783
OR2T35	0.223562334	0.654105403	0.167694894	2.551382866	0.541037593
OR2T4	0.655274618	0.648452531	0.174689561	2.40707391	0.517434826
OR2T6	0.069752183	0.865121269	0.159873506	4.68141864	0.86644129
OR2T8	0.794500799	0.742257977	0.434323517	1.268517321	0.2756762
OR2V2	0.275712918	0.928277921	0.149787257	5.752825129	0.936263697
OR2W1	0.880465582	0.130157989	0.001339698	12.64546252	0.382512449
OR2W3	0.758080274	0.700010086	0.129408849	3.786558068	0.678804279
OR2W5	0.901870132	0.246392215	0.008272123	7.339001438	0.418548269
OR2Y1	0.442496403	22.1490259	0.052333485	9374.100594	0.315421677
OR2Z1	0.112506431	0.051091772	4.28E-05	61.01429455	0.410665426
OR3A1	0.965141507	0.542805449	0.0004664	631.7278228	0.865295586
OR3A2	0.194828682	1.50807643	0.307170422	7.404015353	0.612820992

OR3A3	0.338078183	1.51150033	0.662338444	3.449344166	0.326436352
OR4A16	0.250967116	1.151260185	0.898123597	1.475743448	0.266207279
OR4A47	0.635623108	0.670014847	0.219234395	2.047670925	0.482325893
OR4A5	0.47533787	1.363683943	0.447173481	4.158640825	0.585576682
OR4B1	0.104653572	0.528715351	0.196237869	1.424495304	0.207567302
OR4C11	0.195742369	0.501939629	0.070848232	3.556099886	0.490201726
OR4C12	0.340577416	156.9415126	0.073319632	335935.1079	0.196301169
OR4C13	0.616220307	0.019726883	5.72E-06	68.05748125	0.344891759
OR4C15	0.184581189	0.54116475	0.130264208	2.248194574	0.398085237
OR4C3	0.57635549	0.858439872	0.380659758	1.935899443	0.712960615
OR4C45	0.212490248	1.161992285	0.343342126	3.93259658	0.809273701
OR4C46	0.62522081	2.112774662	0.681124182	6.55360196	0.195289552
OR4C6	0.345689495	0.202865357	0.017176139	2.396018895	0.205400208
OR4D1	0.321716125	297.2069504	0.055745652	1584553.554	0.193398344
OR4D10	0.83638023	0.3815588	0.063204192	2.303440851	0.293560295
OR4D11	0.530784257	0.533970907	0.154591123	1.844380996	0.321170957
OR4D2	0.498746242	0.654165015	0.226452605	1.889719341	0.432976953
OR4D5	0.095913766	44.58775458	0.416993173	4767.626876	0.111152658
OR4D6	0.423674698	13.31528577	0.000471607	375942.1005	0.620512088
OR4D9	0.960953802	12.31125594	3.49E-05	4339630.259	0.70006373
OR4E2	0.154288759	1.596823584	0.675200634	3.776426491	0.286569029
OR4F15	0.544101	1.005566636	0.793867409	1.273719323	0.963289312
OR4F17	0.697235125	16.593999	0.005330673	51655.91544	0.493662027
OR4F21	0.484837442	1.222077272	0.902052698	1.655638148	0.195469908
OR4F29	0.385362734	3.381000151	0.295724683	38.65474441	0.327125746
OR4F4	0.111694335	0.002216226	2.71E-06	1.811147234	0.074040227
OR4F5	0.970113389	0.809699762	0.345127913	1.899625266	0.627552173
OR4F6	0.273584472	0.616659605	0.147334479	2.580991698	0.508063332
OR4K1	0.608561736	1.828682926	0.094917738	35.23136262	0.689233664
OR4K13	0.884473809	0.973193491	0.402857219	2.35097083	0.951851509
OR4K14	0.829379257	0.578395956	0.041697898	8.022991461	0.683242416
OR4K15	0.819153347	1.029731882	0.340049384	3.118216938	0.95866544
OR4K17	0.48320465	5.286391277	0.085020922	328.6947727	0.429398929

OR4K2	0.787518574	0.618675185	0.243637881	1.571015898	0.312542003
OR4L1	0.210768431	0.04053152	0.000517845	3.1723847	0.149583438
OR4M1	0.540604372	0.782618682	0.335173962	1.827385391	0.571040705
OR4M2	0.830703004	1.219347524	0.923961325	1.609167336	0.161156656
OR4N2	0.332247462	1138.682519	0.562158407	2306463.561	0.070033805
OR4N5	0.077929662	0.77667296	0.445688629	1.353458104	0.372453182
OR4P4	0.069326323	43.01942007	0.00582584	317665.8918	0.407821854
OR4Q3	0.299292945	1.03604053	0.290290658	3.697604278	0.956501862
OR4S1	0.928638235	1.141357502	0.548284617	2.375950205	0.723750041
OR4S2	0.554013368	1.658442362	0.293999115	9.35523589	0.566573695
OR4X2	0.886987174	0.956279243	0.805071368	1.135886864	0.610702484
OR51A2	0.293055532	0.993598447	0.762461169	1.294804134	0.962084569
OR51A4	0.274800994	0.188331628	0.003310676	10.71346176	0.418081343
OR51A7	0.457801221	596.6402406	0.095937363	3710541.622	0.151565095
OR51B2	0.553449551	1.460449389	0.745442829	2.861268947	0.269682316
OR51B4	0.785692484	0.381389742	5.96E-05	2441.596792	0.829327753
OR51B5	0.428336868	1.045246626	0.915703595	1.19311589	0.512139746
OR51B6	0.767727002	0.575033119	0.176457004	1.873901747	0.358608856
OR51D1	0.984592134	0.825706735	0.267650374	2.547321727	0.738986979
OR51E1	0.6565554	0.863968055	0.308293338	2.421203141	0.780930154
OR51E2	0.234414409	0.021770636	1.16E-05	40.78977997	0.31952886
OR51F1	0.282641541	0.119645868	0.001574418	9.09233595	0.336589636
OR51F2	0.615870568	0.85691557	0.437112432	1.679897985	0.652996958
OR51G1	0.732125513	0.479859404	0.02933483	7.849544256	0.606591087
OR51G2	0.191014759	1.487179449	0.903508381	2.447905033	0.118548914
OR51I1	0.805468283	0.976542996	0.446905739	2.133864348	0.952540361
OR51I2	0.902920589	0.656670098	0.293867711	1.467380055	0.305271888
OR51L1	0.906660536	0.922375839	0.032436725	26.22882522	0.962268031
OR51M1	0.092405896	1.07569226	0.540748868	2.139835895	0.835282819
OR51Q1	0.588049349	1.933025809	0.488654206	7.646693166	0.347549492
OR51S1	0.599540006	0.953001714	0.456037904	1.991528028	0.898139677
OR51T1	0.608630154	0.313651052	3.65E-05	2692.173284	0.80189302
OR51V1	0.412815203	1.301532558	0.264159843	6.412734718	0.746015472

OR52A1	0.189171623	0.30445603	0.069977527	1.324617746	0.11291435
OR52A4	0.632163535	1.000564295	0.249113766	4.018761892	0.999365503
OR52A5	0.571253162	3.215279285	0.015639133	661.0354321	0.667341009
OR52B2	0.400627771	2.096013634	0.711545637	6.174267568	0.179412185
OR52B4	0.616735983	3.108014377	0.47615885	20.28682942	0.236118132
OR52B6	0.75209565	0.727289501	0.289190543	1.829070941	0.498573176
OR52D1	0.905075139	1.294607235	0.584486252	2.867488992	0.52452136
OR52E2	0.805074158	1.102641281	0.400006449	3.039495485	0.850199274
OR52E4	0.219143975	3.486048169	0.47119601	25.79082074	0.221327204
OR52E5	0.517650464	0.572062146	0.183156049	1.786755613	0.336480118
OR52E6	0.211214204	0.538488227	0.142901752	2.029153359	0.360448583
OR52E8	0.620076469	0.834343844	0.419577924	1.659118865	0.605578133
OR52H1	0.913968577	1.622521408	0.598185937	4.400932145	0.341785033
OR52I1	0.628893709	0.394391855	0.00365652	42.53906786	0.696845296
OR52I2	0.426495723	1.302083455	0.170761857	9.928571614	0.798972847
OR52J3	0.138605673	0.7898713	0.294477772	2.118654546	0.639373916
OR52K1	0.177065147	28.22054254	0.002287853	348098.8457	0.487098885
OR52K2	0.840058396	1.010032507	0.531615156	1.918992818	0.975680815
OR52L1	0.765937119	17.99194897	0.00166477	194447.3854	0.541970773
OR52M1	0.405616985	227.1735039	0.058580479	880972.6669	0.198109648
OR52N1	0.932045358	0.018806881	5.12E-05	6.903811923	0.187254836
OR52N2	0.517014017	0.931624815	0.627877656	1.382315147	0.724988815
OR52N4	0.734259895	8.426789213	0.00127175	55837.04417	0.634942581
OR52N5	0.281988869	1.227701168	0.909619715	1.657011314	0.179979715
OR52W1	0.258546005	3.346013634	0.842087229	13.29530583	0.086199127
OR56A1	0.903803327	0.004302844	4.22E-06	4.382715013	0.123119092
OR56A3	0.309636498	50.89554672	0.304900285	8495.750254	0.132308418
OR56A4	0.66389264	1.191098096	0.491776466	2.884877118	0.698415454
OR56B1	0.717794207	0.744875389	0.000508516	1091.095613	0.936878123
OR56B4	0.883506776	1.285751368	0.855322184	1.932788149	0.226841236
OR5A1	0.517002111	0.467458968	0.121196039	1.803011794	0.269544857
OR5A2	0.152984012	0.825911592	0.529703021	1.287759236	0.398673203
OR5AC2	0.366184591	0.634105823	0.000376648	1067.548661	0.904333679

OR5AK2	0.408354836	119.1226229	0.007797075	1819938.894	0.330816944
OR5AN1	0.646631529	3.663826692	0.28805235	46.60134187	0.316947561
OR5AP2	0.116602087	0.580583284	0.270008071	1.248395826	0.163927834
OR5AR1	0.008062474	2302.172041	1.990801321	2662242.61	0.03145251
OR5AS1	0.832455181	0.562558444	0.011541697	27.41988483	0.771738282
OR5AU1	0.793235244	2.07417711	0.072897142	59.01754961	0.669333927
OR5B12	0.828649011	1.336223664	0.561108778	3.182081178	0.512649571
OR5B17	0.591142968	0.750766939	0.248040891	2.272411601	0.611939459
OR5B2	0.31706479	1.401803155	0.985096523	1.994781263	0.060581259
OR5B21	0.494020388	0.482741692	0.186926395	1.246691466	0.132460717
OR5B3	0.415911833	0.906168354	0.268894481	3.053766972	0.873704206
OR5C1	0.148158058	0.000382411	8.54E-09	17.13227394	0.149851138
OR5D14	0.299422337	200.7824218	0.02424209	1662958.112	0.249368886
OR5D16	0.782044478	490.8819422	0.001869686	128879977.5	0.330431492
OR5D18	0.113724574	1.686614504	0.454943792	6.252791079	0.434277132
OR5F1	0.052134867	0.196739007	0.001684805	22.97372336	0.503218378
OR5H1	0.733853946	1.264858005	0.399827207	4.001392967	0.689258969
OR5H14	0.636891977	2.804196559	0.195110455	40.30290606	0.448305005
OR5H15	0.923953456	1.336938564	0.458013948	3.902511557	0.595217245
OR5H2	0.190011099	0.941889641	0.012394747	71.57516718	0.978384061
OR5I1	0.939726964	0.826386037	0.443452511	1.539993272	0.548218125
OR5J2	0.721945847	0.006874805	2.29E-08	2064.282833	0.439006379
OR5K1	0.611059424	0.93341687	0.398676132	2.18540059	0.873866675
OR5K2	0.305938261	0.986685108	0.55924543	1.740823348	0.963092758
OR5K3	0.344778025	4.071618716	0.000122814	134985.3139	0.791489399
OR5K4	0.700086984	0.880146143	0.573138706	1.35160516	0.559674341
OR5L1	0.461507781	1.025311464	0.768377766	1.368159835	0.865140527
OR5L2	0.218678419	1.198565307	0.861407294	1.667688218	0.282493469
OR5M1	0.277607827	0.815394205	0.567007319	1.172591054	0.27089213
OR5M10	0.650219793	13.51942667	0.428829243	426.2183619	0.139122693
OR5M11	0.845230779	1.239785695	0.679792186	2.261085961	0.483265406
OR5M3	0.857294661	0.239920646	0.009638193	5.972272668	0.384119391
OR5M8	0.649409797	0.004984114	5.85E-07	42.49085987	0.250948356

OR5M9	0.14835396	0.040121994	0.000889103	1.810559342	0.098017972
OR5P2	0.634358382	0.9270758	0.026644567	32.25684011	0.966648846
OR5P3	0.24553104	1.309215901	0.615641793	2.78416166	0.484004201
OR5T1	0.631951211	1.253549234	0.345910811	4.542748103	0.730850827
OR5T2	0.62243665	5.192903274	8.31E-05	324611.8791	0.770006107
OR5T3	0.533963315	0.003509684	3.56E-06	3.455777928	0.107981919
OR5V1	0.234749762	0.543544397	0.124430755	2.37433673	0.417688071
OR5W2	0.977770747	2.303949698	0.000273929	19377.93754	0.856359785
OR6A2	0.874686209	0.260401336	0.032907153	2.060611413	0.202341423
OR6B1	0.108857793	1.519420477	0.418812415	5.512345154	0.524613939
OR6B2	0.903105991	1.986724159	0.262364882	15.04421194	0.506305347
OR6B3	0.956535336	1.976425499	0.531831613	7.344914548	0.309055881
OR6C1	0.439312014	0.948284975	0.535701289	1.678630262	0.855392749
OR6C2	0.790306376	1.984990249	0.372116619	10.58857919	0.422172697
OR6C3	0.890988079	0.78793808	0.346222902	1.793198584	0.570000062
OR6C4	0.944053383	0.931498909	0.508402563	1.706699142	0.818336086
OR6C6	0.540621906	0.730892245	0.261480923	2.04299215	0.550008332
OR6C65	0.846077464	2.384039963	0.458047662	12.40841733	0.301945813
OR6C68	0.200313334	0.008639252	5.21E-06	14.31587068	0.209010198
OR6C70	0.959562619	0.566696029	0.000269874	1189.98035	0.884305138
OR6C74	0.464587337	1.904810742	0.80638496	4.499468791	0.141754348
OR6C75	0.523523635	0.545819112	0.158795772	1.876111055	0.336479613
OR6C76	0.393722154	1.60825794	0.518842392	4.985123882	0.41040015
OR6F1	0.274039588	0.593158552	0.157784736	2.22985491	0.439501513
OR6K2	0.557641627	1.036993887	0.371028306	2.898313428	0.944773304
OR6K3	0.145400035	0.473733893	0.171774256	1.306504286	0.148899626
OR6K6	0.757756539	0.849674751	0.446906868	1.615430939	0.619236058
OR6M1	0.604632928	0.579453577	0.132326983	2.537399694	0.468948135
OR6N1	0.638023456	0.021029728	4.59E-05	9.636615029	0.216727247
OR6N2	0.763297266	1.107057676	0.31037624	3.948680792	0.875439132
OR6Q1	0.87148813	0.454034153	0.035953524	5.733708121	0.541697954
OR6S1	0.839750613	2.645915539	0.037816238	185.1286501	0.653480672
OR6T1	0.923238299	0.545852828	0.153793487	1.937372744	0.348904933

OR6V1	0.516783264	1.394819393	0.644245565	3.019844054	0.398475757
OR6W1P	0.575378112	1.962146162	0.876178474	4.39410197	0.10129331
OR6X1	0.935562912	0.682747646	0.348355274	1.338129155	0.26632128
OR6Y1	0.634558008	0.573332246	0.149707031	2.195687555	0.41680589
OR7A10	0.953195736	0.74095489	0.364847179	1.504778386	0.406854186
OR7A17	0.844634437	1.044031901	0.193581929	5.630704347	0.960028893
OR7A5	0.476697505	0.015506113	4.89E-06	49.17148454	0.311084315
OR7C1	0.312191188	0.005464674	1.62E-06	18.40798109	0.208723034
OR7C2	0.594252797	0.493622117	0.005482499	44.44374559	0.758481285
OR7D2	0.210022863	0.039967302	0.000563062	2.836961991	0.138742376
OR7D4	0.307087644	1.096389023	0.964564162	1.246230098	0.159145533
OR7E156P	0.946880281	0.088030389	3.52E-07	22027.42458	0.701593236
OR7E24	0.759802567	0.941397955	0.275828381	3.212976509	0.923188982
OR7E91P	0.606561598	0.730674406	0.32334781	1.651117067	0.450612562
OR7G1	0.268122128	2.101161367	0.001847786	2389.281115	0.836148738
OR7G2	0.439993163	0.229382986	0.041960003	1.253969269	0.089349564
OR7G3	0.425268384	3.153359922	0.029250213	339.9523553	0.630559796
OR8A1	0.490056219	0.993157393	0.789245211	1.249753047	0.953304091
OR8B2	0.327146356	0.004014197	6.20E-06	2.600197087	0.094792083
OR8B3	0.122433407	3.005577925	0.628338229	14.37680892	0.168181714
OR8B8	0.723935273	1.340459623	0.551700618	3.256896838	0.517696206
OR8D1	0.358348566	0.993667929	0.776969768	1.270803568	0.959636465
OR8D2	0.280911521	3.654670066	0.003802529	3512.560962	0.711498614
OR8D4	0.508265435	1.83976917	0.710464614	4.764136777	0.209185141
OR8G1	0.558025732	0.776247473	0.353310001	1.705471506	0.528247284
OR8G5	0.892128924	0.91214177	0.581926705	1.429737802	0.68840673
OR8H1	0.698759742	0.725657444	0.180469536	2.917826119	0.651501697
OR8H2	0.681669455	0.066070289	0.000448114	9.741453861	0.286215451
OR8H3	0.473380276	0.930011886	0.291515998	2.966979907	0.902435916
OR8I2	0.077708515	1.301644162	0.920610646	1.840384459	0.135734629
OR8J1	0.09701424	30.35241631	0.000639184	1441320.108	0.534473399
OR8J3	0.097017032	1.238435396	0.595640877	2.574910976	0.56690433
OR8K1	0.444383062	0.1895772	0.028417002	1.264718716	0.085901974

OR8K5	0.260995464	24.53509097	0.263722029	2282.595396	0.166462025
OR8S1	0.112008644	1.410195848	0.953651588	2.085302803	0.085035132
OR9A2	0.205965003	0.887140293	0.313600413	2.509620097	0.821428642
OR9A4	0.991676043	1.079131776	0.851654352	1.367368565	0.528352323
OR9G1	0.904774068	0.355363016	0.016788634	7.521926662	0.506482684
OR9G4	0.597979003	0.75971137	0.27683304	2.084871688	0.593653397
OR9I1	0.780275758	0.005246309	1.22E-07	225.3848784	0.334751246
OR9K2	0.876870211	1.317896121	0.001366936	1270.616026	0.937241521
OR9Q1	0.925097423	1.897647419	0.401242506	8.974786242	0.419050431
OR9Q2	0.676229367	2489.523885	0.010827476	572407566.2	0.214431234
ORAOV1	0.339848499	1.533420034	0.663621716	3.543249027	0.317112855
ORM1	0.405653812	1.741241292	0.564982732	5.366396288	0.33417614
ORM2	0.350044006	1.193825688	0.625246748	2.279451715	0.591356683
ORMDL1	0.093714256	0.267850769	0.062794933	1.142513123	0.075084861
ORMDL2	0.7902325	0.854303624	0.605223531	1.205892775	0.370575681
ORMDL3	0.890807939	0.017168087	5.31E-06	55.52137984	0.324233863
OS9	0.539073353	1.266839561	0.151723023	10.57771222	0.827085417
OSBP	0.458140393	0.998937254	0.770218918	1.295574044	0.993604984
OSBP2	0.07778012	0.47058797	0.209370498	1.057708891	0.068124056
OSBPL10	0.383387923	1.085101131	0.276197241	4.263056582	0.906869397
OSBPL11	0.963627147	0.641911385	0.124484216	3.310060013	0.596315663
OSBPL1A	0.975670628	1.676599292	0.232800178	12.07466938	0.607948449
OSBPL2	0.325950579	0.810068758	0.277017359	2.368845746	0.700431261
OSBPL3	0.758035932	1.338879943	0.124399589	14.41001143	0.809768673
OSBPL5	0.472734638	3.406765154	0.350251646	33.1363148	0.290929534
OSBPL6	0.856746596	0.582404497	0.219396069	1.546039546	0.277800955
OSBPL7	0.680579105	6.07781595	1.38E-05	2681706.121	0.785517344
OSBPL8	0.457794263	0.32325333	0.039239909	2.66291941	0.293882671
OSBPL9	0.095183537	3.146779961	0.289356576	34.22152781	0.346449441
OSCAR	0.137417855	0.665851132	0.32381811	1.369156681	0.26884768
OSGEP	0.608505402	0.690102385	0.347381947	1.370944304	0.289555281
OSGEPL1	0.640361651	0.697944956	3.29E-06	148150.638	0.954175447
OSM	0.081985227	0.911455215	0.19634905	4.230988693	0.905775395

OSMR	0.470007084	34.2745001	0.003564964	329524.0554	0.45004013
OSR1	0.913797214	0.675704255	0.218872139	2.086040926	0.495514491
OSR2	0.214200286	0.020676273	0.000188252	2.270938337	0.105693482
OSTF1	0.649015257	0.005183826	6.12E-06	4.390915062	0.126058258
OSTM1	0.63049572	0.464986296	0.024018818	9.001785811	0.612507715
OSTN	0.235276128	0.84254258	0.339041077	2.093781688	0.71220882
OTC	0.771517921	0.983936453	0.790788466	1.224260323	0.88452076
OTOA	0.282643275	1.724003228	0.92407264	3.216399885	0.086936287
OTOF	0.326871532	1.533635173	0.055906747	42.07071511	0.800198734
OTOG	0.718948817	0.695184775	0.008890353	54.36025964	0.870148609
OTOP1	0.835960848	0.198962904	2.06E-07	192195.6531	0.818372487
OTOP2	0.882202151	0.086482369	0.00059633	12.54205888	0.33505653
OTOP3	0.842355458	0.45652492	2.88E-05	7236.07449	0.873738054
OTOR	0.864970652	0.786740741	0.000166096	3726.519931	0.955701647
OTOS	0.130234033	0.670607546	0.236829627	1.898894515	0.451801988
OTP	0.330176707	1.238265675	0.707782915	2.166344865	0.453933217
OTUB1	0.624882056	0.900688722	0.31572456	2.569455397	0.844954546
OTUB2	0.700484204	1.013742425	0.875433417	1.17390276	0.855290693
OTUD1	0.764220561	11.74778391	0.383825335	359.5657034	0.158129576
OTUD3	0.879705121	0.982000761	0.709820189	1.35854898	0.91266504
OTUD4	0.886889218	1.032847663	0.645169223	1.653479827	0.892915078
OTUD5	0.932447011	3.985328818	0.2869681	55.34707793	0.303018767
OTUD6A	0.531251481	2.046848062	0.706409903	5.930815764	0.186951726
OTUD6B	0.550366329	0.993395647	0.663992853	1.486213153	0.974282445
OTX1	0.109395775	0.647037671	0.01000475	41.84589669	0.83784269
OTX2	0.185078009	0.538506482	0.109838426	2.640143717	0.445417674
OVCH1	0.575089784	0.414543292	0.08269657	2.078032274	0.284322964
OVCH2	0.659235643	0.614339444	0.305361263	1.235955565	0.171937013
OVGP1	0.224119366	0.513762007	0.205316703	1.285581721	0.154691375
OVOL1	0.938004996	2.341795153	0.645077392	8.50131256	0.195823416
OVOL2	0.429460134	5.160177615	0.006099554	4365.471804	0.633254314
OXA1L	0.249746806	1.118906655	0.901002763	1.389509727	0.309318743
OXCT1	0.955828673	6.797351381	0.000886013	52148.22902	0.674542254

OXCT2	0.086535264	1.937490401	0.93629057	4.009299222	0.074660427
OXER1	0.999132631	0.019956167	1.33E-05	30.01646295	0.294349315
OXGR1	0.164611997	1.05431658	0.84187305	1.320369443	0.645008283
OXR1	0.514194431	1.127237069	0.300838381	4.223741019	0.858952088
OXSM	0.56102197	0.72269858	0.416913858	1.252760557	0.247239991
OXSR1	0.255847119	1.41399536	0.961530298	2.079375849	0.078307625
OXT	0.826811238	1.018412823	0.76658108	1.352974533	0.899819239
OXTR	0.792486509	0.834153452	0.121356899	5.733600525	0.853719503
P2RX1	0.358576418	0.434437225	0.000201849	935.0349896	0.831387318
P2RX2	0.706952102	2.396938955	0.04027524	142.6513245	0.674990178
P2RX3	0.623097383	1.102109084	0.277851404	4.371561262	0.890005594
P2RX4	0.254606377	0.340269005	0.042223003	2.742178128	0.311294003
P2RX5	0.526789817	0.765180821	0.356743227	1.641241219	0.491815239
P2RX7	0.282927915	374.5335254	0.268344728	522743.1243	0.108735525
P2RY1	0.284381443	1.560301024	8.06E-05	30201.34046	0.929609413
P2RY10	0.779744056	0.876583191	0.368023627	2.08790424	0.766104148
P2RY11	0.466991301	1.086113981	0.767033726	1.53792922	0.641594147
P2RY12	0.842150032	1.067283813	0.890521579	1.279132099	0.480892387
P2RY13	0.592683353	0.001255264	2.23E-09	707.2465336	0.322765386
P2RY14	0.812063811	5.464132378	0.104442141	285.8687346	0.400304194
P2RY2	0.502418566	1.550140881	0.636579501	3.774763005	0.334376035
P2RY4	0.099088539	4.34349161	0.473749655	39.82255012	0.193898774
P2RY6	0.290895006	1.30269817	0.497226678	3.412975603	0.590494476
P2RY8	0.83012934	1.247351001	0.56898244	2.734503581	0.581022364
P4HA1	0.630905577	0.797113882	0.009962518	63.77810861	0.919217511
P4HA2	0.226351988	1.401767003	0.649040908	3.027468233	0.389966908
P4HA3	0.811148616	0.941681928	0.578014481	1.534156811	0.809324516
P4HB	0.249438783	0.897127053	1.84E-05	43761.09617	0.984274806
PA2G4	0.663521383	1.616379118	0.726156117	3.597961087	0.239523977
PABPC1	0.100382374	0.130795061	0.00903341	1.893786281	0.135783717
PABPC3	0.408631325	42.64489153	0.116288905	15638.52349	0.212860755
PABPC4	0.583399694	0.69325729	0.319008595	1.506560253	0.354918071
PABPC5	0.736685314	0.463039121	0.017103374	12.53584363	0.647315651

PABPN1	0.60048966	1.21677136	0.463145999	3.196686453	0.690543932
PACRG	0.728555138	0.961125476	0.740583758	1.247343288	0.765601728
PACS1	0.384276607	1.030499544	0.668632899	1.588209782	0.891719204
PACS2	0.176636785	1.051564995	0.752644277	1.469205271	0.768255103
PACSIN1	0.669825818	0.145225393	0.005523618	3.818225077	0.247377159
PACSIN2	0.140950139	0.202544088	0.016394716	2.502276147	0.213170079
PACSIN3	0.763372393	4.043267853	0.004326184	3778.853023	0.688927712
PADI1	0.805146633	1.024112591	0.845989801	1.239739058	0.806919288
PADI2	0.337049147	1.600636244	0.861098617	2.975311231	0.136968992
PADI3	0.673798461	0.920366406	0.441250577	1.919712665	0.824907735
PADI4	0.375054155	2.327081523	0.881893461	6.140547192	0.087991804
PADI6	0.765540644	0.66261198	0.096896675	4.531163076	0.674795036
PAEP	0.101214129	0.009323496	7.09E-06	12.25900399	0.20197069
PAF1	0.680313786	0.800987011	0.329247526	1.948625701	0.624683542
PAFAH1B1	0.260324244	0.18465629	0.025658873	1.328894903	0.093428754
PAFAH1B2	0.550692562	1.325129953	0.227311463	7.724948707	0.754302714
PAFAH1B3	0.200742421	0.797900394	0.605674241	1.051134413	0.108414431
PAFAH2	0.052683658	0.000601486	3.13E-08	11.57702795	0.140642659
PAG1	0.91863827	1.191201112	0.228656836	6.205631607	0.835410391
PAGE1	0.382544241	1.343784109	0.362539898	4.980846911	0.658444912
PAGE2	0.385392168	0.689183318	0.13554793	3.504101073	0.653681461
PAGE2B	0.075715415	0.869363738	0.205442012	3.678864418	0.849152166
PAGE3	0.66530416	0.94719162	0.762027386	1.177348718	0.624943049
PAGE4	0.326911215	0.541165318	0.215264895	1.360462892	0.191724387
PAGE5	0.659032381	28.06889881	0.353999591	2225.604493	0.135033791
PAH	0.812828116	0.210362916	0.002747884	16.10423059	0.481221053
PAICS	0.262377674	1.193747012	0.889648969	1.601791244	0.237792018
PAIP1	0.345693305	0.724042821	0.205831489	2.546928119	0.614845814
PAIP2	0.177341708	0.867601894	0.469548786	1.603098694	0.650273847
PAK1	0.662699834	2.713103666	0.026460342	278.1873163	0.672665349
PAK1IP1	0.919677658	1.247393883	0.646193797	2.407933203	0.510061437
PAK2	0.563675649	0.064364525	3.65E-05	113.3894981	0.471915283
PAK3	0.894403587	1.313340765	0.216688272	7.960116869	0.766856288

PAK4	0.197376769	0.222132568	0.022964971	2.148614875	0.193807538
PAK6	0.652973327	0.22422275	7.06E-05	712.514939	0.716311195
PAK7	0.731037151	554.4049309	0.211615251	1452470.113	0.115661411
PALLD	0.38961409	1.032722641	0.761710239	1.400159797	0.835753429
PALM	0.949261227	1.447975992	0.398835209	5.256894139	0.573648236
PALM2	0.087451393	1.9952458	0.986692816	4.034696246	0.054520725
PALM2-AKAP2	0.248869497	0.223558773	0.032892769	1.519438077	0.125493218
PALMD	0.513904698	274.354576	0.467581083	160978.3547	0.084305162
PAM	0.966708809	1.123471504	0.209951648	6.011804304	0.891786985
PAN3	0.232321215	0.000260096	1.26E-08	5.378047749	0.103495931
PANK1	0.702894529	0.175503733	1.74E-05	1775.086191	0.711504116
PANK2	0.704540175	0.231995313	0.002178545	24.70539654	0.539584387
PANK3	0.369951057	1.029728693	0.001167053	908.5626291	0.993245586
PANK4	0.312040229	19.0630085	0.64098786	566.934752	0.088564473
PANX1	0.499811613	29.76976862	0.245171713	3614.769072	0.165790865
PANX2	0.079930723	1.903363594	0.087675751	41.32035291	0.681899659
PANX3	0.289234336	0.763478614	0.027370735	21.29645355	0.873735508
PAOX	0.496724703	1.07375971	0.888090998	1.298245246	0.462514364
PAPD4	0.05345801	0.070901458	0.002445171	2.055895592	0.123450076
PAPD5	0.532211978	1.321116163	0.342585689	5.094631706	0.685928454
PAPLN	0.111952052	1.615103163	0.997515965	2.615054112	0.05119422
PAPOLA	0.439150178	0.963162954	0.203882279	4.550090763	0.962212061
PAPOLB	0.136435362	0.446762277	0.007640386	26.12387936	0.697908598
PAPOLG	0.804213446	0.863032649	0.522561929	1.425334132	0.56498748
PAPPA	0.580314838	1.022150221	0.761573751	1.371884301	0.883987366
PAPPA2	0.103150106	0.052776888	0.000931158	2.991330452	0.153278801
PAPSS1	0.214466037	0.002744902	1.06E-07	70.95908215	0.255215918
PAPSS2	0.147041465	0.013556687	9.94E-05	1.849352511	0.086378183
PAQR3	0.431027718	2.470804684	0.099329416	61.46090507	0.581199049
PAQR4	0.129506099	1.183880101	0.891498777	1.572152569	0.243468467
PAQR5	0.602881559	1.306272915	0.5308287	3.214500136	0.560887042
PAQR6	0.695712538	0.589284805	0.167444197	2.073864531	0.41006932
PAQR7	0.862524921	1.401622638	0.166883995	11.77192589	0.755833215

PAQR8	0.673822017	5.83064257	0.002736323	12424.11415	0.652075947
PAQR9	0.230676778	0.03816332	4.23E-05	34.42489902	0.346868875
PARD3	0.252866149	0.859958102	0.343109271	2.15537148	0.747586145
PARD6A	0.949797602	1.261570197	0.881992062	1.804505314	0.203248596
PARD6B	0.866424669	1.048450012	0.380066869	2.892247441	0.927185667
PARD6G	0.641636461	1.209880713	0.538794226	2.716828188	0.644361481
PARG	0.68342946	1.054158195	0.50787547	2.188035386	0.887430122
PARK2	0.309613454	742.3960975	0.006709542	82144501.87	0.264651135
PARK7	0.608271061	1.074944591	0.820557486	1.408196127	0.59990851
PARN	0.455895141	0.813199489	0.098818523	6.691998485	0.847518368
PARP1	0.292321489	0.312694426	0.053970262	1.811697783	0.194640232
PARP10	0.165348194	0.478308955	4.68E-06	48878.90535	0.900273356
PARP12	0.174178842	0.065219087	1.47E-05	288.6603714	0.523897457
PARP14	0.412943089	0.812313518	0.453791569	1.454088829	0.484097102
PARP15	0.096865283	1.53088958	0.738665581	3.172779352	0.252084323
PARP16	0.105245162	0.000797357	9.12E-08	6.970666194	0.12340262
PARP2	0.242941127	3.033961561	0.665102369	13.83985861	0.151770637
PARP3	0.086560191	1.670232049	0.8133291	3.429946249	0.162358384
PARP4	0.464642939	7.830786586	0.192973022	317.7709401	0.276050229
PARP6	0.227939335	0.565119714	0.183930859	1.736306197	0.318991209
PARP8	0.340501042	0.930345469	0.720775285	1.200849572	0.579280231
PARP9	0.688205896	1.242843237	0.310715785	4.971293331	0.758562253
PARS2	0.592340497	0.901201811	0.442074488	1.837167098	0.7746807
PART1	0.161595622	2.04749979	0.516583113	8.115355083	0.30777427
PARVA	0.307340218	0.005306481	2.90E-06	9.7025764	0.171622125
PARVB	0.271282456	0.72294476	0.373450558	1.399513578	0.33573795
PARVG	0.85558028	0.824171323	0.048743234	13.93543898	0.893378845
PASD1	0.196439219	0.559206034	0.189720741	1.648272013	0.291939125
PASK	0.616278261	0.630015257	0.151279681	2.623744458	0.525599555
PAWR	0.156511877	1.666090513	0.895772306	3.098842843	0.106892754
PAX1	0.711590576	1.073964669	0.714840941	1.613505948	0.731157905
PAX2	0.453189596	0.837274247	0.614952358	1.139971504	0.259336175
PAX3	0.888302997	1.025887231	0.800264186	1.315121466	0.840164

PAX4	0.131980414	3.633796663	0.302987284	43.58096495	0.308708981
PAX5	0.694782304	2.343523478	0.668539891	8.215070437	0.183262143
PAX6	0.558564859	1.198160359	0.593763517	2.417777793	0.613763311
PAX7	0.368456569	0.001387556	3.32E-08	58.06867061	0.225545828
PAX8	0.241705447	1.045030802	0.869973745	1.25531303	0.637731113
PAX9	0.302595253	1.205621442	0.702629736	2.068689934	0.497257765
PAXIP1	0.064128118	0.724681762	0.206214992	2.54668029	0.615537377
PBK	0.600316757	0.520210852	5.75E-06	47035.5366	0.910634872
PBOV1	0.159947965	0.488061374	0.231398348	1.029410567	0.059586781
PBX1	0.952193824	1.197497353	0.58068766	2.469485765	0.625501791
PBX2	0.190345988	0.451680973	0.157635683	1.294222845	0.13893461
PBX3	0.133795826	0.298169244	0.002353855	37.76990662	0.624228403
PBX4	0.817730105	3.492342875	0.003222627	3784.632827	0.725776917
PBXIP1	0.424767228	0.951843655	0.827661787	1.094657695	0.488964064
PC	0.571570721	1.154039155	0.833155256	1.598509236	0.388758788
PCBD1	0.900955141	0.898836893	0.288815353	2.797315839	0.853916983
PCBD2	0.511375233	0.006484744	2.10E-06	20.06687456	0.219213633
PCBP1	0.709644012	0.260900582	0.002613924	26.04096624	0.567269193
PCBP2	0.886734735	1.853349142	0.664430867	5.169692157	0.238457825
PCBP3	0.372253974	12.03810953	0.002769798	52320.08273	0.560478697
PCBP4	0.59290448	0.46969775	0.016016744	13.77408429	0.661104212
PCCA	0.544388277	0.000267867	3.09E-08	2.318771034	0.075380434
PCCB	0.219670681	1.235036761	0.602461786	2.531805062	0.56435275
PCDH1	0.297471945	0.688522765	7.08E-05	6699.126025	0.936511213
PCDH10	0.367331691	4.069881786	0.009640898	1718.090824	0.649062082
PCDH11X	0.760828068	0.878425954	0.519326424	1.485832649	0.628834493
PCDH11Y	0.189467517	2.423413316	0.780533364	7.524255043	0.125690606
PCDH12	0.378281037	1.093942429	0.817606716	1.463674423	0.545570583
PCDH15	0.958399626	0.783363893	0.168369694	3.644711673	0.755603614
PCDH17	0.173759502	1.166004548	0.802110813	1.69498601	0.421014889
PCDH18	0.756956446	0.943766059	0.723971227	1.230289742	0.668753968
PCDH19	0.987294466	1.696919691	0.495037699	5.816802324	0.40016629
PCDH20	0.54491533	0.010596671	2.02E-05	5.556419779	0.154675457

PCDH7	0.923445333	1.028926824	0.581813847	1.819637698	0.921905641
PCDH8	0.71095146	1.056373683	0.141612345	7.880141795	0.957341514
PCDH9	0.110890465	5.551106816	0.692369411	44.50628004	0.106567296
PCDHA1	0.786623131	0.74487907	0.447388421	1.24018594	0.257480062
PCDHA10	0.135358518	63.8644662	0.363405678	11223.46263	0.114991736
PCDHA11	0.873837715	17.54752452	0.464033321	663.5635906	0.122174148
PCDHA12	0.817107869	8.185765221	0.001677241	39950.59111	0.627549747
PCDHA13	0.230478634	0.482853657	0.134785565	1.729767231	0.263454698
PCDHA2	0.773188157	0.818799719	0.047934663	13.98639194	0.890189283
PCDHA3	0.487462506	0.759489488	0.405370592	1.422955424	0.390441878
PCDHA4	0.423065319	23.96626604	0.059089405	9720.556682	0.299847208
PCDHA5	0.559882504	1.431195762	6.88E-05	29787.88472	0.943662723
PCDHA6	0.222368988	2.713912143	0.716425947	10.28064261	0.141773451
PCDHA7	0.472237654	1.181612851	0.255202961	5.470974642	0.831000926
PCDHA8	0.079319311	2.277133095	0.365647079	14.18125683	0.377863534
PCDHA9	0.389287957	6.555558328	0.01257808	3416.685529	0.555808111
PCDHAC1	0.733442244	1.266258475	8.85E-05	18112.71502	0.961432782
PCDHAC2	0.706653954	6.938433249	0.038244717	1258.784474	0.465390617
PCDHB1	0.299531374	0.667327567	0.286244684	1.555753198	0.348973575
PCDHB10	0.433304817	0.693372569	0.200752392	2.394818389	0.562563485
PCDHB11	0.417259559	0.057385751	0.001493808	2.204516535	0.124709812
PCDHB12	0.624493363	0.184142012	0.000118612	285.876548	0.651736307
PCDHB13	0.910130563	0.979865954	0.210828105	4.554123789	0.979299393
PCDHB14	0.096266523	1.511810843	0.8533766	2.678268918	0.156615915
PCDHB15	0.910577062	1.409110691	0.341058305	5.821857768	0.635629729
PCDHB16	0.64143783	1.055717686	0.637520488	1.74824159	0.83312768
PCDHB19P	0.3069407	0.723191875	0.314161887	1.664767462	0.44616259
PCDHB2	0.856351763	0.477755178	0.022841812	9.992640244	0.63396657
PCDHB3	0.052213383	0.259160325	0.057023417	1.177833194	0.080451416
PCDHB4	0.673011041	0.115944275	1.53E-05	877.2892452	0.636338067
PCDHB5	0.635331941	2.312242596	0.14554042	36.73526452	0.55247381
PCDHB6	0.779819202	1.94033649	0.31691855	11.87972649	0.473373878
PCDHB7	0.769386647	0.055652468	0.00136784	2.264298022	0.126579345

PCDHB8	0.363609798	8.872690747	0.44113806	178.458057	0.154003318
PCDHB9	0.966475773	0.047847523	3.88E-09	590323.7356	0.715203129
PCDHGA1	0.791228213	6.55945286	0.000488693	88043.86995	0.69811778
PCDHGA10	0.338061704	1.128551332	0.91233534	1.39600874	0.265078343
PCDHGA11	0.079572597	0.373646015	0.08283533	1.685408204	0.2002609
PCDHGA12	0.162410875	0.567312435	0.167573487	1.920610499	0.362277131
PCDHGA2	0.071742715	6.437635817	0.216649382	191.2913604	0.281878668
PCDHGA3	0.290366187	1.863730824	0.369065097	9.411598696	0.45113271
PCDHGA4	0.932914978	1.372860518	0.625265583	3.014312723	0.429683509
PCDHGA5	0.368292779	1.022368911	0.736413563	1.419363037	0.894859474
PCDHGA6	0.353876911	1.391547466	0.378663764	5.113783088	0.618784203
PCDHGA7	0.822206112	0.849111388	0.416600698	1.730650366	0.652555173
PCDHGA8	0.122652594	0.022145627	7.39E-05	6.6338499	0.190333809
PCDHGA9	0.161468018	0.814182055	0.001125112	589.1788966	0.951205605
PCDHGB1	0.212775534	1.213276707	0.774689577	1.900168032	0.398325408
PCDHGB2	0.129987767	1.81736185	0.583464088	5.660681035	0.302757281
PCDHGB3	0.842309136	2.693739255	0.03587349	202.2727978	0.652914392
PCDHGB4	0.795603076	0.25952687	0.003581451	18.80639783	0.537062596
PCDHGB5	0.82233593	2.790577761	0.929725422	8.375939887	0.067245774
PCDHGB6	0.526657074	4.743312251	0.008460609	2659.266139	0.629745986
PCDHGB7	0.624858177	0.027173445	1.93E-06	382.6363627	0.459442572
PCDHGB8P	0.856617637	0.020764758	3.96E-07	1087.808484	0.484652672
PCDHGC3	0.115305692	20.78459224	0.002672398	161652.3197	0.506820626
PCDHGC4	0.952289908	0.846060466	0.50385372	1.420686766	0.527301606
PCDHGC5	0.216633149	1.840120106	0.819822177	4.130215186	0.139313296
PCF11	0.398985028	2.038871178	0.551386146	7.539173246	0.285648092
PCGF1	0.206926938	0.732167805	0.227049794	2.361022601	0.601770613
PCGF2	0.219400598	1430.279328	0.189718345	10782821	0.110700739
PCGF3	0.51453184	1.196484726	0.336120469	4.259114909	0.781844098
PCGF5	0.939031217	0.50445447	6.66E-05	3819.709182	0.88064727
PCGF6	0.143155647	0.671297471	0.328649912	1.371186415	0.274093862
PCK1	0.212969479	11.81976033	0.800537911	174.5160752	0.072176379
PCK2	0.915784514	0.5425603	0.217437514	1.353821949	0.189979892

PCM1	0.584844881	1.015788958	0.262003187	3.938223875	0.981922499
PCMT1	0.626931818	1.020143694	0.431466687	2.411989586	0.963768617
PCMTD1	0.602918547	0.637506985	0.119245102	3.40823354	0.598648587
PCMTD2	0.569378154	0.722541574	0.313969591	1.662792642	0.444744346
PCNA	0.369669443	0.786486844	0.06759376	9.151163646	0.847881642
PCNP	0.122915711	2.758669648	0.824547174	9.229621383	0.099585453
PCNX	0.796830831	0.841063725	0.274284273	2.579032994	0.762070965
PCNXL2	0.683208701	0.915455663	0.430622815	1.946155759	0.818435275
PCNXL3	0.363970305	0.331163023	0.054157156	2.025013051	0.231605725
PCOLCE	0.335760087	0.041932059	0.000387436	4.538293612	0.18447906
PCOLCE2	0.678005019	0.970767917	0.271325262	3.473286417	0.963617531
PCP2	0.713556496	1.21362267	0.153033393	9.624565971	0.854597743
PCP4	0.638329304	0.974292344	0.452466074	2.097937563	0.94693861
PCSK1	0.896163021	1.704158067	0.465697553	6.236139093	0.420605388
PCSK1N	0.755144332	10.64016098	0.000527437	214647.4361	0.640092677
PCSK2	0.449655673	0.997458358	0.761596949	1.306364446	0.985249738
PCSK4	0.535337306	0.007426846	2.16E-07	254.8254743	0.35751016
PCSK5	0.609795305	1.266681451	0.55266107	2.903193272	0.576412115
PCSK6	0.772989583	0.47428345	0.070401649	3.195163656	0.443419742
PCSK7	0.337464934	0.780825005	0.398703509	1.529175626	0.470639094
PCSK9	0.7640992	1.275695133	0.488419855	3.331965428	0.619131246
PCTP	0.874694523	0.772042971	0.395272689	1.507947212	0.448792425
PCYOX1	0.776007721	1.622524711	0.699830071	3.761750957	0.259293021
PCYT1A	0.798458391	0.814842567	0.383384171	1.731861823	0.594526127
PCYT1B	0.774873442	0.746146808	0.17405577	3.198601581	0.693349462
PCYT2	0.299379272	0.481217749	0.131971339	1.754703135	0.267818898
PDAP1	0.441990383	0.016103251	4.75E-05	5.461324028	0.164871097
PDC	0.849574468	1.072804948	0.265539056	4.334241732	0.921417479
PDCD1	0.059446826	1.101063676	0.574095554	2.111741172	0.772003764
PDCD10	0.154134397	0.000911832	3.81E-08	21.82054582	0.173607141
PDCD11	0.796696558	0.929736645	0.670813957	1.28859905	0.66177794
PDCD1LG2	0.228985613	1.121679195	0.415747949	3.026266806	0.820613102
PDCD2	0.957898006	1.631342535	0.097167948	27.38843952	0.733811446

PDCD4	0.179971851	0.947616241	0.268510208	3.344291997	0.933353977
PDCD5	0.234579252	0.772887964	0.266256428	2.24353571	0.635634655
PDCD6	0.572918218	1.862559911	0.521852058	6.647725863	0.33801608
PDCD6IP	0.956052714	0.860603024	0.243254084	3.04470763	0.815864743
PDCD7	0.097766772	112.4575473	0.046814627	270144.1995	0.234402184
PDCL	0.924361805	1.309188899	0.555119117	3.087581601	0.538268699
PDCL2	0.419145071	0.389620861	0.136943709	1.108516893	0.077253443
PDCL3	0.996525813	0.587473029	0.073004183	4.72746281	0.617109881
PDDC1	0.854452487	1.25402211	0.621747203	2.529277886	0.527151634
PDE10A	0.894245868	1.066987381	0.733549964	1.551989813	0.734490055
PDE11A	0.73497182	0.197439941	0.010548656	3.695497208	0.277732346
PDE1A	0.92926286	1.155286697	0.212417178	6.283330591	0.867326002
PDE1B	0.752410159	0.79738732	0.123183074	5.161638845	0.8121886
PDE1C	0.470444253	0.577813737	0.097618882	3.420124349	0.545459935
PDE2A	0.914563998	0.964352964	0.716280041	1.298342248	0.810930116
PDE3A	0.492573598	1.292515858	0.528797277	3.159239495	0.573638868
PDE3B	0.346448672	5.40E-05	4.70E-11	62.0237682	0.167521143
PDE4A	0.070485627	1.703795901	0.592869115	4.89639348	0.322498389
PDE4B	0.649761801	1.73096012	0.677152066	4.424741631	0.251872838
PDE4C	0.071135142	66.22928123	0.199564612	21979.43637	0.156833127
PDE4D	0.449164649	2.015144002	0.491241919	8.266406415	0.330577958
PDE4DIP	0.736959408	0.007645788	4.44E-06	13.15504786	0.199811534
PDE5A	0.086907423	1.044574243	0.168597366	6.471841006	0.962621687
PDE6A	0.383023996	5.677396002	0.043549784	740.1374383	0.484668396
PDE6B	0.955154214	1.600389209	0.009996915	256.2035961	0.855909771
PDE6C	0.453568509	0.835781305	0.584998706	1.194071683	0.324364444
PDE6D	0.818891502	0.773342539	0.400065394	1.494902314	0.444661167
PDE6G	0.810113282	1.501519365	0.496552839	4.540423955	0.471541468
PDE6H	0.998271601	1.095313938	0.048617097	24.67676388	0.954316992
PDE7A	0.269378746	0.702265445	0.139263183	3.541329045	0.66853579
PDE7B	0.416601766	0.219585654	0.023327716	2.066977258	0.185090107
PDE8A	0.588201922	0.053862078	1.92E-06	1513.842298	0.576198293
PDE8B	0.762564621	1.373469511	0.503132856	3.749344682	0.535686904

PDE9A	0.757111563	0.007639647	3.40E-09	17180.56754	0.513627646
PDF	0.981441879	0.932855983	0.696998971	1.24852449	0.640227
PDGFA	0.066100528	1.097141215	0.923242197	1.303795308	0.292377443
PDGFB	0.22474116	0.409099553	0.114639995	1.45989577	0.168501883
PDGFC	0.29278725	1.563041994	0.018538971	131.7818716	0.843512182
PDGFD	0.274537647	3.988304267	0.05714647	278.3473913	0.523057264
PDGFRA	0.963587562	0.782634671	0.001174923	521.3254659	0.94110122
PDGFRB	0.922923309	1.13350104	0.001369456	938.2005595	0.970839241
PDGFRL	0.397109516	2.419072872	0.20696445	28.27496976	0.48129288
PDHA1	0.390836927	0.000523143	2.69E-08	10.17904585	0.133749991
PDHA2	0.488464372	1.276077139	0.376643099	4.323384312	0.695370863
PDHB	0.099551478	0.655001937	0.409696935	1.047182686	0.077163418
PDHX	0.610148081	0.63553714	0.334522228	1.207415898	0.166255434
PDIA2	0.406273039	0.643217568	0.23086425	1.792087078	0.398630986
PDIA3	0.760294704	1.061582024	0.000378781	2975.222012	0.988227825
PDIA4	0.587636774	0.068512326	4.37E-06	1075.096023	0.586539512
PDIA5	0.530292149	1.637248638	0.059358431	45.15926515	0.770821457
PDIA6	0.631848234	0.43042515	0.00234335	79.06025078	0.751297143
PDIK1L	0.181167338	2.031610425	0.004554981	906.1379671	0.819850632
PDILT	0.618172181	0.14779311	0.007314768	2.986124092	0.212524714
PDK1	0.272242077	1.716512424	1.053282757	2.797363655	0.030136278
PDK2	0.55905832	0.717697519	0.08486925	6.069215045	0.760730647
PDK3	0.303002722	1.11548805	0.768171719	1.619837803	0.565810153
PDK4	0.799572467	1.252469184	0.881955254	1.778637917	0.208390071
PDLIM1	0.133694282	0.348113563	0.07299694	1.660111401	0.185509272
PDLIM2	0.788923906	0.922476028	0.539412711	1.577571319	0.768184807
PDLIM3	0.195799742	0.17120469	0.000784485	37.36342933	0.520682222
PDLIM4	0.723774275	0.932049336	0.314768138	2.759859908	0.898898865
PDLIM5	0.865169763	0.929177165	0.713625513	1.209836515	0.585433227
PDLIM7	0.248991745	0.761789691	0.441786103	1.313584853	0.327694822
PDP2	0.159887199	0.753784817	0.538615671	1.054910916	0.099303788
PDPK1	0.849423813	1.121595921	0.131204948	9.587880842	0.916520957
PDPN	0.799805182	0.234540405	0.009308173	5.909774138	0.378411102

PDPR	0.490519137	0.911074713	0.287456681	2.887590328	0.874271788
PDRG1	0.558287209	0.763158904	0.437023084	1.332679059	0.341975116
PDSS1	0.812356832	47.24703215	0.002921929	763975.3857	0.435541373
PDSS2	0.517031211	0.71756541	0.105089938	4.899613851	0.734900087
PDXK	0.761867115	0.911587069	0.248690194	3.341470657	0.888919606
PDXP	0.601438093	1.285622733	0.863465187	1.914177706	0.216044091
PDYN	0.740129874	52.18252268	0.20081414	13559.88019	0.163298526
PDZD11	0.555792152	277.3709958	0.009578147	8032312.853	0.283188446
PDZD3	0.955810717	1.212425833	0.756296687	1.943650456	0.423736929
PDZD4	0.077708515	2.769932798	0.892487693	8.596788245	0.077879148
PDZD7	0.35231182	0.03981854	0.001316797	1.204070272	0.063854037
PDZD8	0.342508949	0.533748324	0.228016676	1.249414205	0.147948245
PDZK1	0.074448469	1.08462311	0.755816653	1.556471779	0.659356233
PDZK1IP1	0.646932537	0.825001513	0.178471184	3.813654846	0.805468508
PDZRN3	0.240589826	0.586718022	0.16774746	2.052120712	0.403905015
PDZRN4	0.098802017	0.358668596	0.113855634	1.129879634	0.079878978
PEA15	0.339887314	0.058153353	0.000859162	3.936177085	0.185901216
PEBP1	0.232000187	1.05047577	0.342111995	3.225549994	0.931441712
PEBP4	0.438230228	0.013520258	6.45E-05	2.831972262	0.114516131
PECAM1	0.242492058	1.08507277	0.895665268	1.314534522	0.404179896
PECR	0.503406165	6.798893985	0.018735933	2467.18211	0.523875489
PEF1	0.083879629	13.82474863	0.030267174	6314.552971	0.400589843
PEG10	0.787571241	1.119091699	0.202833313	6.174361668	0.89725923
PEG3	0.756362859	0.998313403	0.28953525	3.442170337	0.997867355
PELI1	0.251688485	1.243751521	0.310346026	4.984493818	0.758101059
PELI2	0.886346147	0.972279067	0.115542018	8.181669328	0.979362531
PELI3	0.326012276	1.536994378	0.944509718	2.501140722	0.083600686
PELO	0.486335266	1.704343866	0.421972148	6.883838257	0.454110741
PELP1	0.949371261	1.174664457	0.136997009	10.07201979	0.883260769
PEMT	0.747910351	1.263073354	0.440248528	3.623758394	0.664064379
PENK	0.863757659	1.602104029	0.538786834	4.763919898	0.396614156
PEPD	0.374916954	4.537192638	0.704411539	29.22455962	0.111547627
PER1	0.829114264	0.250309956	0.000437332	143.2666406	0.668999714

PER2	0.734095944	2.175262519	0.772725597	6.123476492	0.141100929
PER3	0.74447393	1.272289261	0.503938675	3.212136799	0.610298208
PERP	0.607778106	1.181986384	0.00957587	145.8971092	0.945747456
PES1	0.597366891	0.796004868	0.122694943	5.1642206	0.81099833
PEX1	0.544693894	0.065731913	0.000363928	11.87235013	0.304540878
PEX10	0.451735993	0.97209943	0.460882865	2.050363277	0.940760673
PEX11A	0.675405272	1.757249675	0.57462897	5.373774352	0.322906362
PEX11B	0.102146166	68.40845521	0.016439654	284660.2857	0.32032413
PEX11G	0.875378457	1.811169958	0.476959928	6.877593744	0.382938682
PEX12	0.868448563	0.848803318	0.596179012	1.208474399	0.363118427
PEX13	0.118002943	0.008842371	7.86E-09	9948.104223	0.505984728
PEX14	0.653980322	0.040284837	0.000376665	4.308518708	0.177891659
PEX16	0.963870906	0.837853243	0.349313282	2.009651777	0.691859026
PEX19	0.69718849	0.029007259	3.53E-05	23.8403593	0.301212834
PEX26	0.840871499	0.990913378	0.763494369	1.286072777	0.945291616
PEX3	0.073033237	0.744426094	0.094668076	5.853823542	0.779090318
PEX5	0.203043128	1.036854721	0.552375023	1.946264166	0.910312166
PEX5L	0.252634691	4.166516432	0.281083412	61.76052537	0.299548685
PEX6	0.103970106	1.385226953	0.912639339	2.102532327	0.125871114
PEX7	0.174829029	1.78165504	0.358127314	8.863592802	0.48047894
PF4	0.144323993	0.020119513	9.50E-05	4.261134261	0.152865077
PF4V1	0.739169391	0.042990585	6.02E-05	30.72534467	0.347997563
PFAS	0.220807823	3.078594437	0.409098933	23.16736355	0.274838658
PFDN1	0.377152219	8.539975411	0.009851689	7402.911495	0.534340542
PFDN2	0.062985821	1.501097693	0.758416513	2.971051191	0.243566953
PFDN4	0.485017998	0.924934811	0.429456498	1.992063015	0.841990943
PFDN5	0.606463777	0.867564677	0.590105269	1.275481697	0.469988622
PFDN6	0.05868443	574.0365879	0.094703933	3479454.257	0.152842644
PFKFB1	0.417347198	0.327614755	0.002289905	46.87156523	0.659456715
PFKFB2	0.920292785	1.119322325	0.674299889	1.858049345	0.662883353
PFKFB3	0.629170819	1.441725367	0.477002351	4.357571887	0.516810645
PFKFB4	0.139863972	0.742691072	0.399612099	1.380313633	0.346852048
PFKL	0.30533662	0.736368104	0.447970254	1.210433013	0.227497802

PFKM	0.729697595	2.189757621	0.287743172	16.66429965	0.449083177
PFKP	0.933764688	1.016553879	0.201429913	5.130230034	0.984139595
PFN1	0.310494724	1.63897481	0.018462514	145.4968907	0.829097714
PFN2	0.813890818	0.583268432	0.21411429	1.588880703	0.291709893
PFN3	0.950018443	0.996127588	0.466361833	2.127683057	0.992005112
PFN4	0.221639962	1.168522169	0.484534796	2.818051605	0.728780486
PGA5	0.346774869	0.010203326	1.75E-05	5.962665648	0.158353075
PGAM1	0.113635469	0.527430175	0.233113215	1.19333685	0.124618634
PGAM4	0.741667571	1.029719106	0.324529103	3.267261485	0.96035269
PGAM5	0.18152252	0.000596883	2.02E-08	17.62215361	0.157472214
PGAP1	0.062530029	1.665422234	0.795660345	3.485948792	0.175914356
PGBD1	0.541059701	3.59971966	0.062972277	205.7727974	0.534938369
PGBD2	0.422139682	0.130722698	2.52E-08	677921.8207	0.796465016
PGBD3	0.26218077	11059.58064	0.034366527	3559112145	0.150143249
PGBD4	0.568691378	0.596089785	0.075904134	4.68120793	0.622704922
PGBD5	0.172720347	1.544725742	0.383574634	6.220895242	0.540667913
PGC	0.285685889	0.772040321	0.509026092	1.170954234	0.223464673
PGD	0.702449337	0.787716641	0.247981177	2.502195983	0.685740023
PGF	0.75583512	0.998266934	0.646731429	1.540882084	0.99375121
PGGT1B	0.685007579	0.963224299	0.290402461	3.194880117	0.951161465
PGK1	0.181495519	103.4197176	0.245761966	43520.31428	0.132393012
PGK2	0.739151503	0.835088025	0.195880355	3.560193713	0.807544366
PGLS	0.421551188	0.855620669	0.295290878	2.479205369	0.773907971
PGLYRP1	0.06216004	353.4149319	0.002091869	59708386.63	0.339379917
PGLYRP2	0.423060884	1.504244022	0.661656483	3.419826051	0.32988176
PGLYRP3	0.138059476	0.408039762	0.097444143	1.708634728	0.219894844
PGLYRP4	0.924773486	5.528148545	0.005769119	5297.243244	0.625437708
PGM1	0.110499857	7.11E-06	9.27E-12	5.454002785	0.086419836
PGM2	0.492310726	0.366287854	6.20E-06	21648.07534	0.857809952
PGM2L1	0.546579225	1.709798014	0.365372321	8.001178732	0.495728946
PGM3	0.993617842	0.567849055	0.121270059	2.65896258	0.472490178
PGM5	0.536293122	2.374332226	0.697639219	8.080757734	0.166425518
PGM5P2	0.308679393	1.48189623	0.423837095	5.181274741	0.537982515

PGPEP1	0.55733314	0.852306577	0.087444275	8.307307755	0.890587004
PGR	0.919399457	0.719970634	0.161277708	3.214069197	0.666893948
PGRMC1	0.343835728	914.6350059	0.001872393	446784983.1	0.307619153
PGRMC2	0.793586381	5.201349414	0.023280751	1162.077451	0.55018412
PHACTR1	0.320605156	1.374162391	0.914052756	2.065878872	0.126524375
PHACTR2	0.985031956	0.944533455	0.565465582	1.577714854	0.827428146
PHACTR3	0.539866058	1.719017526	0.639953243	4.617558061	0.282558433
PHACTR4	0.658866548	0.795271346	0.191868954	3.296294168	0.7521832
PHB	0.738894974	1.047103838	0.58137878	1.885907237	0.878142086
PHB2	0.485861196	3.484720267	0.731999655	16.58918178	0.116859316
PHC1	0.332500626	1.066180184	0.470465658	2.416202257	0.87798618
PHC2	0.261345261	1.054481261	0.737436265	1.507832991	0.771253887
PHC3	0.491307007	0.496826122	0.072455275	3.406738758	0.476390207
PHEX	0.515099786	0.712822761	0.226704778	2.241312661	0.562472195
PHF1	0.318781682	0.945456573	0.444520099	2.010905992	0.884186129
PHF10	0.484526357	1.260181369	0.592047902	2.682311816	0.548507363
PHF11	0.166978604	1.330318919	0.848811153	2.084973106	0.213143615
PHF12	0.517422444	0.616879603	0.174083523	2.185964749	0.454222555
PHF13	0.413028267	1.128292254	0.47522433	2.678826249	0.7843892
PHF14	0.543316014	1.36001931	0.972326713	1.902295289	0.072486524
PHF19	0.59231184	0.11773981	0.001250274	11.08770181	0.356263271
PHF2	0.64442349	0.950499961	0.357731265	2.525499625	0.918897375
PHF20	0.068248122	0.708868241	0.471192789	1.066430119	0.098677026
PHF20L1	0.670567849	1.971299564	0.077335854	50.24864595	0.681236389
PHF21A	0.143749169	0.598346451	0.293184682	1.221136361	0.158224924
PHF21B	0.716614684	0.865549681	0.436439517	1.716563742	0.679378816
PHF23	0.832238906	0.910741525	0.778681451	1.065198258	0.242102992
PHF3	0.19190125	4.145110137	1.90E-05	904968.1401	0.82066065
PHF5A	0.843535323	0.935990813	0.613237182	1.428613314	0.75914089
PHF6	0.756929127	0.993040631	0.774692428	1.272930596	0.956039015
PHF7	0.76370655	1.0384479	0.349410469	3.086267114	0.945876291
PHF8	0.248578422	0.304580513	0.053595864	1.730903881	0.17990076
PHGDH	0.441566606	0.002154138	1.48E-07	31.27497673	0.209175919

PHIP	0.111952052	0.949366684	0.259527414	3.472839675	0.937411421
PHKA1	0.925390933	4.501965982	0.03893682	520.5278154	0.534760412
PHKA2	0.183462382	0.521441843	0.221019255	1.230216775	0.13705114
PHKB	0.936469921	1.315619991	0.695217446	2.489661286	0.399284663
PHKG1	0.510157516	0.23380084	0.009387704	5.822811684	0.375644936
PHKG2	0.342168317	1.079332306	0.439218345	2.652344192	0.867826131
PHLDA1	0.899888762	0.878835945	0.331635917	2.328917284	0.795055793
PHLDA2	0.840532012	1.591732419	0.542465201	4.670552304	0.397368298
PHLDA3	0.097862176	0.578810468	0.276678032	1.210871551	0.146532346
PHLDB1	0.712418863	2.284446587	0.440786776	11.83950268	0.325061678
PHLDB2	0.469825833	0.851086401	0.116425628	6.221551706	0.873773717
PHLDB3	0.431781117	0.570953493	0.047182746	6.909048653	0.659526411
PHOSPHO1	0.192043529	1.579402801	0.006458457	386.2398317	0.870605603
PHOSPHO2	0.05775158	2717.460543	0.313796814	23533036.29	0.087374595
PHOX2A	0.8897372	1.109163776	0.506510111	2.42886421	0.79558005
PHOX2B	0.266550973	2.904808123	0.007088769	1190.32093	0.728263569
PHPT1	0.925366261	0.999893453	0.618047033	1.617655073	0.999653638
PHTF1	0.944687455	0.282776078	0.000115417	692.8099132	0.751067797
PHTF2	0.608783446	0.316791943	0.000141823	707.6218056	0.770161331
PHYH	0.91561519	0.102143873	1.26E-05	829.1856054	0.619384458
PHYHD1	0.409749321	0.023959174	0.000129278	4.440381588	0.161374856
PHYHIP	0.188305285	1.817519986	0.889075607	3.715520786	0.101485836
PHYHIPL	0.246044911	1.15436011	0.738826781	1.803599029	0.52837879
PI15	0.744638149	1.540027764	0.348387111	6.807615552	0.569063067
PI16	0.395651298	0.010785494	6.43E-06	18.09974361	0.231858371
PI3	0.697064796	1.101025228	0.338758373	3.57852868	0.872856473
PI4K2B	0.578836425	1.303106091	0.372468479	4.559004532	0.678624139
PIAS1	0.348336289	0.684496185	0.225236137	2.080194744	0.503867541
PIAS2	0.821790897	0.874204075	0.399396459	1.913469056	0.736590131
PIAS3	0.692866741	3.810655478	0.407918071	35.59806784	0.240619031
PIAS4	0.898987883	8.494142562	0.732147735	98.54631034	0.087142567
PICALM	0.975786143	0.630751464	0.003281037	121.2565988	0.863627244
PIGA	0.313254102	0.972950169	0.780407404	1.212997244	0.807438576

PIGB	0.918860958	1.155383629	0.873003501	1.529101921	0.312441029
PIGC	0.437086508	0.997920661	0.411544793	2.419774617	0.996325063
PIGF	0.429539863	0.161335161	0.007855977	3.313277998	0.236778669
PIGG	0.205957275	0.489816905	0.018121174	13.2397931	0.671352242
PIGH	0.380643279	1.164169983	0.886814592	1.528269564	0.273595397
PIGK	0.434408407	1.665532428	0.872926021	3.177815991	0.121703862
PIGL	0.067281481	2.007974906	0.47173254	8.54713823	0.34552689
PIGM	0.5667657	0.913991625	0.297592274	2.807131645	0.875176082
PIGN	0.406268674	0.064575057	1.21E-05	345.8777081	0.531672931
PIGO	0.816577707	0.485068021	0.132788128	1.771927871	0.273733951
PIGQ	0.923431546	1.387247823	0.72354742	2.659751757	0.324328634
PIGR	0.993279077	0.808631822	0.30595795	2.137174152	0.668390811
PIGS	0.650734536	0.818295106	0.486273785	1.377016201	0.450139125
PIGT	0.270713067	1.031989002	0.787123429	1.353029603	0.819761933
PIGV	0.132583923	0.427500501	0.130458909	1.400875418	0.160526329
PIGW	0.816650821	0.840388028	0.518091217	1.363180874	0.481062509
PIGX	0.263672512	7.664473914	0.432092144	135.9528544	0.165119478
PIGZ	0.898202808	0.889919665	0.338859947	2.337121922	0.812861679
PIK3AP1	0.065399769	70.66551902	0.007429701	672115.2368	0.362267953
PIK3C2A	0.149393932	0.800791756	0.601295886	1.066475677	0.128586833
PIK3C2B	0.439436673	0.779616538	0.394891091	1.539163483	0.473155216
PIK3C2G	0.754652814	0.900304501	0.359177964	2.256675728	0.822754904
PIK3C3	0.640316447	0.736153549	0.367985604	1.472671871	0.386577616
PIK3CA	0.137660196	0.661115393	0.434984012	1.00480374	0.052680975
PIK3CB	0.898190434	0.811673002	0.162505612	4.054094228	0.799288142
PIK3CD	0.383675048	0.474998243	0.196961515	1.145519877	0.097422568
PIK3CG	0.153371433	0.515892885	0.171628955	1.550702615	0.238525227
PIK3R1	0.191091046	0.851247247	0.010604697	68.33027702	0.942618567
PIK3R2	0.65956126	0.628787511	0.09999992	3.953740511	0.620895871
PIK3R3	0.341378328	1.408206616	0.66185771	2.996181572	0.374206191
PIK3R4	0.474444743	0.072581365	0.000342295	15.39040251	0.337190688
PIK3R5	0.430498061	0.400577131	0.041340208	3.881500479	0.429804233
PILRA	0.23895583	1.397616413	0.963677914	2.026954867	0.077579136

PILRB	0.155377774	0.05647508	0.000727745	4.382626894	0.195516698
PIM1	0.061394515	2.564552107	0.8347546	7.878875426	0.100059464
PIM2	0.558656094	0.321138126	0.018355829	5.618362281	0.436627798
PIM3	0.076217648	3.366564742	0.664397272	17.05870666	0.142613272
PIN1	0.796930138	0.713864028	0.020462225	24.90451753	0.852458435
PIN4	0.087770856	1.156242232	0.414602312	3.224526395	0.781447598
PINK1	0.417758097	0.380807524	0.082513491	1.75746255	0.215969317
PINX1	0.240737114	1.065303205	0.572161505	1.983480029	0.841898862
PIP	0.819348082	1.066323866	0.888889583	1.279176412	0.489214647
PIP5K1A	0.505687426	1.160128586	0.866754913	1.552801507	0.317999504
PIP5K1B	0.876738353	1.14540103	0.47764287	2.746703869	0.760969421
PIP5K1C	0.897322648	0.991689233	0.244841726	4.016666404	0.990670135
PIP5KL1	0.485709038	4.112705936	0.235996612	71.67200395	0.332173867
PIPOX	0.521265764	0.236596535	0.03509371	1.595098377	0.138767669
PIR	0.281858345	1.414981935	0.585376174	3.42032007	0.440815489
PISD	0.285309442	0.094526207	0.001973493	4.527608825	0.232109745
PITPNA	0.833336835	0.384574695	0.000243883	606.4289556	0.799210043
PITPNB	0.943392861	1.53594677	0.376127545	6.2721609	0.549962201
PITPNC1	0.621597172	0.998648734	0.70173887	1.421182915	0.994007078
PITPNM1	0.24230194	1.415883303	0.786592259	2.548620973	0.246230442
PITPNM2	0.217546226	0.085394859	0.000553586	13.17280619	0.33852035
PITPNM3	0.104036403	1.374370474	0.327619561	5.76551106	0.66380813
PITRM1	0.661513355	41.12227295	0.830743578	2035.575571	0.061926651
PITX1	0.890764934	0.787358804	0.420414418	1.474578083	0.455187704
PITX2	0.745868015	1.200796396	0.223375336	6.455108292	0.831140481
PITX3	0.79623444	0.549759401	0.177513497	1.702605184	0.299597183
PIWIL1	0.285102091	0.633795206	0.233533923	1.720077145	0.370661544
PIWIL2	0.27952703	0.851423444	0.191292132	3.78960637	0.832779611
PIWIL3	0.250124236	12.43934732	0.144274217	1072.522626	0.267614403
PIWIL4	0.976071367	0.942962316	0.171182867	5.194316139	0.946215479
PJA1	0.281401873	0.868493352	0.472947783	1.594849855	0.649336285
PJA2	0.737827818	1.877964985	0.000246638	14299.30685	0.890087098
PKD1	0.501951432	0.812181644	0.09129207	7.225589502	0.852013869

PKD1L1	0.367347493	0.219065711	0.021343803	2.248417805	0.201247446
PKD1L2	0.524186745	0.944916435	0.693095739	1.288230498	0.72011436
PKD1L3	0.641406052	1.040406964	0.858095498	1.261452429	0.686950883
PKD2	0.616793798	0.026413607	1.90E-05	36.70852322	0.325035941
PKD2L1	0.870806516	2.638632554	0.405517739	17.16911761	0.309919554
PKD2L2	0.954299674	4.90756244	1.79E-06	13467657.1	0.833424915
PKDREJ	0.868165475	1.023399401	0.715595552	1.463600956	0.899168593
PKHD1	0.875762752	0.820623915	0.219209032	3.072061425	0.769120036
PKHD1L1	0.911410987	0.005700748	3.22E-06	10.0821149	0.175636633
PKIA	0.19204448	0.010736346	3.02E-07	381.125861	0.396331422
PKIB	0.146291104	0.51478427	0.262282561	1.010371576	0.053610098
PKIG	0.330836706	1.342541757	0.74900899	2.406404188	0.322506871
PKLR	0.073542645	1.21936962	0.783209813	1.898421401	0.379886256
PKMYT1	0.793598934	2.465176772	0.014115872	430.5151272	0.731949465
PKN1	0.166988401	2.753690917	0.007956386	953.0475349	0.734185712
PKN2	0.127333678	1.814331676	0.360095888	9.141452431	0.470280603
PKN3	0.545408443	0.085245548	1.60E-05	453.977607	0.573818193
PKNOX1	0.189662026	0.387862423	0.083734992	1.796587733	0.225936547
PKNOX2	0.206413442	36.27698445	0.026233383	50165.83679	0.330419599
PKP1	0.544014441	0.626573411	0.212562485	1.846959208	0.396670424
PKP2	0.61972848	1.042229608	0.597438607	1.818165988	0.884170331
PKP3	0.422078931	0.330171884	0.029567921	3.686883295	0.368056916
PKP4	0.54370291	3.356486524	0.367832863	30.62804579	0.283091733
PLA1A	0.684158143	0.088848837	3.67E-07	21507.6456	0.701918739
PLA2G10	0.677011824	0.432174236	0.000389531	479.4862973	0.814592889
PLA2G12A	0.532701918	1.984988381	0.958135913	4.112338155	0.065053876
PLA2G12B	0.967287203	0.991360244	0.382625665	2.568555183	0.98574711
PLA2G1B	0.867446113	1.01942125	0.72526692	1.432878923	0.91182594
PLA2G2A	0.604954256	1.069323961	0.851300309	1.343184914	0.564520252
PLA2G2D	0.348960686	1.165824813	0.893826143	1.520594922	0.257676617
PLA2G2E	0.300708733	1.167097364	0.592465701	2.299063481	0.65509316
PLA2G2F	0.661407904	0.01110844	5.88E-06	20.99390377	0.242367841
PLA2G3	0.696091226	0.807228926	0.371972883	1.751790438	0.588006912

PLA2G4A	0.100470112	0.776462245	0.323362452	1.86445153	0.571328806
PLA2G4B	0.820325973	0.004419786	1.68E-06	11.62716477	0.177219523
PLA2G4C	0.391746622	1.636186821	0.634196077	4.221261233	0.308578147
PLA2G4D	0.441002761	1.870947575	0.45562371	7.682753886	0.384723819
PLA2G4E	0.11036365	0.410474847	0.07174333	2.34850542	0.317029862
PLA2G4F	0.987630009	0.661497602	0.185848552	2.354492801	0.523491818
PLA2G5	0.397194249	2197.821217	0.157862689	30598858.65	0.113934628
PLA2G6	0.54161538	0.352466804	0.039031186	3.18291247	0.353008038
PLA2G7	0.645147562	1.746144491	0.446617482	6.826917223	0.422974534
PLA2R1	0.267910106	0.019697483	4.03E-05	9.634797942	0.213877465
PLAA	0.844925447	0.695458346	0.142420245	3.396022181	0.653518713
PLAC1	0.981779076	0.070347413	0.000151163	32.73790028	0.397052942
PLAC4	0.349968145	0.882816622	0.608697207	1.280382396	0.511154605
PLAC8	0.439349838	0.591740158	0.276992519	1.26413672	0.17549336
PLAC8L1	0.561236228	0.124109599	0.002365971	6.510304065	0.301723734
PLAC9	0.878090332	73.37231494	0.019935763	270042.1624	0.305187349
PLAG1	0.303194742	1.060183673	0.600499842	1.871756399	0.840300916
PLAGL1	0.428540387	0.704791373	0.41813725	1.187961318	0.189058272
PLAGL2	0.948524727	0.062012438	4.20E-07	9164.915955	0.647091629
PLAT	0.627217685	1.497828777	4.18E-06	536363.7745	0.950627111
PLAU	0.751320083	0.478474218	0.003754716	60.97333629	0.765669859
PLAUR	0.328796277	58938.87153	0.020352832	1.70678E+11	0.147913201
PLB1	0.483625801	1.245877091	0.173914437	8.925134393	0.826784514
PLCB1	0.033105954	0.327876985	0.116934772	0.919344312	0.034020637
PLCB2	0.96050081	24.29996161	0.000657304	898348.8601	0.552154833
PLCB3	0.92615911	1.134810396	0.75767589	1.699664267	0.53948791
PLCB4	0.010465829	1.899903349	1.224044305	2.94893961	0.004220247
PLCD1	0.099715712	23.26593074	0.22372796	2419.471995	0.184154097
PLCD3	0.471123245	0.005764603	6.31E-06	5.268429328	0.138273799
PLCD4	0.197147667	1.41893675	0.693828841	2.901841754	0.337768705
PLCE1	0.923802824	1.357377555	0.679518068	2.711441998	0.386753333
PLCG1	0.758270436	0.780221755	0.087071925	6.991300429	0.824453445
PLCG2	0.477070483	0.588828482	0.254695018	1.361310414	0.215491182

PLCL1	0.308445561	2.147410509	0.793221043	5.8134765	0.132563367
PLCL2	0.316720837	4.167320379	0.824067493	21.07419511	0.084353559
PLCXD1	0.846855335	1.025586304	0.670870776	1.567853758	0.907125988
PLCXD2	0.920820614	1.932263638	0.720751697	5.180206695	0.190486616
PLCXD3	0.447678091	0.024578293	2.26E-05	26.74454	0.298904819
PLCZ1	0.800614335	0.778167869	0.039490092	15.33410544	0.869013373
PLD1	0.893650985	0.299903231	0.000140489	640.207488	0.758159693
PLD2	0.683528764	0.038006319	4.53E-06	319.1517599	0.478132328
PLD3	0.001897011	0.40600305	0.219443529	0.75116581	0.004086015
PLD4	0.143023955	0.000845373	5.70E-07	1.25441651	0.057547959
PLD5	0.126631964	4.592029691	0.12783779	164.949165	0.404155184
PLEK	0.969378627	0.786149257	0.128545108	4.807889337	0.794540549
PLEK2	0.105587934	1.459937717	0.950285326	2.242924393	0.084131191
PLEKHA1	0.182688772	6.68E-05	3.41E-10	13.06729539	0.121973625
PLEKHA2	0.446217729	1.324047761	0.610604664	2.871092505	0.477215663
PLEKHA3	0.057958439	0.689299706	0.440377857	1.078923649	0.103597031
PLEKHA4	0.394705454	1.102781363	0.853237542	1.425308516	0.454806857
PLEKHA5	0.715287807	1.104676122	0.4703599	2.594416176	0.819236365
PLEKHA6	0.86770731	1.072774371	0.830051846	1.386473454	0.591442217
PLEKHA7	0.145731528	1.869956246	0.634011698	5.515255277	0.25670411
PLEKHA8	0.509476192	1.135763599	0.598920017	2.153808385	0.696605922
PLEKHB1	0.382451549	4.428194217	0.000392494	49959.74953	0.754621565
PLEKHB2	0.825965832	0.669397437	9.83E-05	4556.853696	0.928974575
PLEKHF1	0.757533213	7.489297761	4.35E-06	12881623.18	0.783426828
PLEKHF2	0.952407416	0.756244016	0.291567002	1.961487443	0.565598272
PLEKHG1	0.141238393	1.267707054	0.384879361	4.175545215	0.696518583
PLEKHG2	0.388075078	1.08306787	0.91194672	1.286298843	0.363109116
PLEKHG3	0.210500481	341.7376313	0.518335678	225306.9077	0.078145762
PLEKHG4	0.334327927	0.586336852	0.152738739	2.250842885	0.436652655
PLEKHG5	0.596989444	1.565651902	0.607073306	4.037841644	0.353702377
PLEKHG6	0.469863198	1.126691918	0.781342976	1.624683037	0.522992248
PLEKHH1	0.769115419	0.801771891	0.588750557	1.091868462	0.160867622
PLEKHH2	0.361454032	2.33618259	0.833659417	6.546737175	0.106544017

PLEKHH3	0.30571576	1.045883968	0.931188782	1.174706241	0.449055548
PLEKHJ1	0.632486736	144.3886744	0.001971228	10576195.32	0.384274079
PLEKHM1	0.483928682	1.120298903	0.5684825	2.207754208	0.742761858
PLEKHM2	0.231498945	0.917788874	0.455453285	1.849446352	0.810353071
PLEKHN1	0.754598071	0.02305383	2.84E-06	186.8855869	0.411673531
PLEKHO1	0.315998567	1.09617106	0.186456159	6.444362045	0.919075158
PLG	0.05985343	42.4261251	0.002754112	653559.6145	0.446186563
PLGLB2	0.481413295	1.279781377	0.023546915	69.55647225	0.903680596
PLK1	0.276038034	1.060669179	0.786167432	1.431017187	0.699891155
PLK2	0.646095574	1.282787503	0.556789889	2.955412465	0.558662219
PLK3	0.17084619	0.000717818	3.00E-08	17.15080352	0.159300604
PLK4	0.66196916	1.129233426	0.973867402	1.309385783	0.107543529
PLN	0.719437777	4.134916345	0.069866099	244.7185887	0.495377391
PLOD1	0.625383384	1.022225848	0.70303496	1.48633531	0.908367328
PLOD2	0.292621194	1.77198518	0.964540312	3.25536573	0.06523877
PLOD3	0.392295472	0.130942807	0.010225109	1.676854397	0.11813746
PLP1	0.993671527	1.381443867	0.63961004	2.983672923	0.410810554
PLP2	0.920513153	0.966823518	0.49965063	1.870802637	0.920203449
PLRG1	0.570510763	0.630196143	0.170259737	2.332595988	0.489254952
PLS1	0.863099754	0.736534807	5.62E-05	9652.383383	0.949592913
PLS3	0.965873473	0.891028981	0.301662371	2.631858406	0.834609669
PLSCR1	0.228088902	1.430463686	0.678526196	3.015692496	0.346816431
PLSCR2	0.198730947	51.99839033	0.108876608	24833.91661	0.209334625
PLSCR4	0.225137276	0.77332121	0.246019818	2.430802927	0.659996799
PLSCR5	0.624929845	1.016092511	0.480618327	2.148157764	0.966662145
PLTP	0.233035657	203.8148797	0.596826704	69602.2898	0.074010538
PLVAP	0.773617932	2.538122025	0.02128758	302.6207514	0.702586257
PLXDC1	0.113407081	0.715696704	0.203121339	2.521752636	0.602681745
PLXDC2	0.661529556	0.650452372	2.99E-06	141445.34	0.94531591
PLXNA1	0.920767863	0.964438366	0.795370155	1.169444636	0.712713371
PLXNA2	0.070800021	2.261530416	0.819815575	6.238622415	0.11497642
PLXNA3	0.137039058	0.778245267	0.19302966	3.137682037	0.724498395
PLXNB1	0.505728003	0.644920611	0.234474952	1.773846592	0.395499026

PLXNB2	0.490663907	0.088434625	4.97E-07	15733.17175	0.69414279
PLXNB3	0.417501688	0.563964428	0.102592426	3.100188669	0.510079902
PLXNC1	0.274333728	0.746941949	0.259963122	2.146159312	0.587947909
PLXND1	0.103472549	0.357634336	0.002062694	62.00741276	0.69586579
PMAIP1	0.59190524	0.591617422	0.115679571	3.025695646	0.528456993
PMCH	0.471201787	1.046185248	0.895614827	1.222069509	0.569035904
PMCHL1	0.869039034	0.907420982	0.702827365	1.171571967	0.456119817
PMCHL2	0.561632368	262.4930293	0.681372439	101123.2426	0.066702863
PMF1	0.059895695	0.002026421	3.41E-07	12.05175388	0.161937093
PMFBP1	0.968661366	0.992358261	0.780872877	1.261120659	0.949980044
PML	0.652759703	1.153667857	0.450621858	2.953584028	0.765680556
PMM1	0.484414274	1.134702739	0.380061931	3.38773816	0.820856493
PMM2	0.308692245	0.349549056	0.003582429	34.10661856	0.652888462
PMP2	0.32855081	0.862128208	0.187458526	3.964957277	0.848871412
PMP22	0.528573811	2.524273926	0.928481536	6.8627739	0.069593355
PMPCA	0.184590707	1.820083607	0.606820131	5.459120701	0.28523677
PMPCB	0.836643489	1.015285069	0.28651923	3.597677442	0.981250658
PMS1	0.668988774	1.094573738	0.841520271	1.423722884	0.500527293
PMS2	0.976352987	0.102335977	0.00080353	13.0333031	0.35665876
PMS2CL	0.751347218	1.490349188	0.238910725	9.296948485	0.669241056
PMS2L2	0.773239909	0.738272726	0.285329939	1.910232832	0.53157872
PMVK	0.164453055	2.256100891	0.385052593	13.21895068	0.367070932
PNCK	0.386078019	0.929422796	0.640813174	1.348016502	0.699640048
PNKD	0.2328674	0.211610432	0.02096459	2.135933776	0.187976119
PNKP	0.891993834	1.20320527	0.471056458	3.073310846	0.699027563
PNLDC1	0.465021781	83.94151445	0.10575897	66624.87226	0.19343935
PNLIP	0.216272394	1.133034336	0.73549651	1.74544242	0.571040587
PNLIPRP1	0.484642063	0.759061721	0.142732264	4.036751604	0.746451339
PNLIPRP2	0.149612898	0.173728382	0.005943792	5.077827887	0.309445614
PNLIPRP3	0.383797234	0.503218025	0.018322186	13.82086058	0.684536859
PNMA1	0.242347888	0.327553425	0.052926852	2.027160913	0.230089182
PNMA2	0.947798923	0.930152922	0.296600565	2.917002056	0.901186206
PNMA3	0.064024491	14.8698266	0.591356297	373.9061276	0.100867405

PNMA5	0.286662255	0.584854762	0.153240312	2.232148237	0.432491274
PNMA6A	0.144598246	0.272371738	0.04264533	1.739612836	0.169213256
PNMT	0.785943424	1.206994517	0.571426612	2.549471329	0.621925044
PNN	0.732125268	1.321893084	0.456457407	3.828180457	0.606983646
PNOC	0.546979878	0.668945038	0.285500623	1.567378239	0.354714403
PNPLA1	0.111874393	0.467821907	0.106920877	2.046909293	0.313092091
PNPLA2	0.456132105	0.124848812	0.000148652	104.8574391	0.544746445
PNPLA4	0.089159069	5.131277703	0.611492263	43.05861654	0.131866777
PNPLA5	0.962771196	1.151109649	0.564174184	2.348660153	0.698920506
PNPO	0.962257884	0.897871173	0.064880558	12.42548881	0.935950892
PNPT1	0.050150103	97.46983224	0.012202572	778554.5889	0.31784465
PNRC1	0.626947199	1.031166436	0.000712332	1492.708323	0.993405265
PNRC2	0.919629149	1.422073307	0.320571617	6.308395329	0.643184892
PODN	0.188411265	5.141069396	0.546706718	48.34510658	0.152180598
PODXL	0.519842225	1.053343042	0.942020396	1.177821169	0.361818974
PODXL2	0.338906118	0.635687091	0.206644219	1.955525688	0.429408158
POF1B	0.249274908	0.779944125	0.041327189	14.71943421	0.868302348
POFUT1	0.878687086	0.817812488	0.427727035	1.563654415	0.543066352
POFUT2	0.107748955	0.006053781	8.68E-06	4.221414287	0.126304434
POGK	0.20143302	0.538909891	0.222953711	1.302619582	0.169796327
POGZ	0.936627091	0.941833321	0.763184782	1.162300437	0.57654708
POLA2	0.153234434	3.218225412	0.978345254	10.58621663	0.054363603
POLB	0.294940386	0.940683793	0.766941853	1.15378499	0.557252263
POLD1	0.986015668	2.002277397	0.00215575	1859.73061	0.842168013
POLD2	0.096114044	2.11578285	0.862609228	5.189530699	0.101607348
POLD3	0.942796119	1.314416765	0.839956677	2.056881599	0.231457778
POLD4	0.489683371	2.37310823	0.42917981	13.12187233	0.321938964
POLDIP2	0.144321359	3.324397536	0.294447545	37.53340505	0.331377054
POLDIP3	0.100881778	2.125257034	0.843145583	5.356984076	0.10998564
POLE	0.905499741	0.882371839	0.105258616	7.396829783	0.908161414
POLE2	0.957709644	0.897615035	0.566801794	1.421507058	0.645161849
POLE3	0.311786001	2.439265859	0.331760432	17.93468224	0.381019181
POLE4	0.591005784	0.876398293	0.314518028	2.442066587	0.800782569

POLG	0.447288567	1.077119122	0.392843388	2.953303116	0.885216765
POLG2	0.705858254	94.15493322	0.053831994	164681.8327	0.232869635
POLH	0.308570518	0.760215953	0.366250926	1.577957223	0.461863561
POLI	0.601685743	40.21502574	0.056194876	28779.28411	0.270663784
POLK	0.39957084	1.061939882	0.479471265	2.351999789	0.882239936
POLL	0.41811067	0.705552383	0.226817356	2.194735769	0.546931466
POLM	0.469009265	391.3033179	0.050709757	3019503.464	0.191180162
POLN	0.713956108	5.180416288	0.000767647	34959.7077	0.714629987
POLQ	0.931020794	0.602398171	0.189479133	1.915163695	0.390422553
POLR1A	0.446289086	0.160144023	0.001144695	22.40430821	0.467477621
POLR1B	0.383434141	35.35058065	0.001291304	967752.9849	0.494026143
POLR1C	0.799209636	1.262098696	0.706478941	2.254692996	0.431700344
POLR1D	0.360946331	0.395198973	0.018232622	8.566087086	0.554184349
POLR2A	0.69424759	1.313768671	0.561697845	3.072805309	0.529026885
POLR2B	0.10971164	0.266686335	0.007607498	9.348881673	0.466441878
POLR2C	0.523251629	0.786479923	0.528847403	1.16962032	0.235547265
POLR2D	0.520983586	0.007227221	5.48E-06	9.525058205	0.178617103
POLR2E	0.743228585	264.1107408	0.078434628	889332.7497	0.178403388
POLR2F	0.732176962	0.906235284	0.358637238	2.289952918	0.835096382
POLR2G	0.054518174	0.485224792	0.130824208	1.799690611	0.279559661
POLR2H	0.648722495	1.45709382	0.444806375	4.773138425	0.534065939
POLR2I	0.254158763	5.160211936	0.983696714	27.06910255	0.052315826
POLR2J	0.54886404	2.515981999	0.624649432	10.1339489	0.194293052
POLR2J2	0.522769498	0.039753577	4.00E-07	3951.252321	0.582782681
POLR2J3	0.408580209	3.136843718	0.268062641	36.70704908	0.36233167
POLR2K	0.802872654	1.018294281	0.425698635	2.435815285	0.967502623
POLR2L	0.875517975	0.218801389	0.00131023	36.53864665	0.560607502
POLR3A	0.173538451	0.825210449	0.266761216	2.552740971	0.73880655
POLR3B	0.508563291	0.758435269	0.196962903	2.92046903	0.687720887
POLR3C	0.622975222	0.862620364	0.630538703	1.180124057	0.355381852
POLR3D	0.840812389	0.894638715	0.275073406	2.909690334	0.853210722
POLR3E	0.130371796	1.130498381	0.815841285	1.56651375	0.461119734
POLR3F	0.117850733	0.544894389	0.125161889	2.372206893	0.418518225

POLR3G	0.013362507	1.885297834	0.997073009	3.564782008	0.051063582
POLR3GL	0.300013963	1.654981589	0.625671302	4.377640547	0.310057206
POLR3H	0.674415605	4.752338242	0.775396936	29.12665463	0.091995135
POLR3K	0.349237621	1.884462884	0.764446041	4.645455888	0.1686756
POLRMT	0.719809534	1.656858891	0.289078416	9.496320846	0.570846153
POM121	0.600540686	2.383434887	0.353639068	16.06372815	0.372292511
POMC	0.36401316	1.952823283	0.012371207	308.2576054	0.795515228
POMGNT1	0.606643537	0.009827023	1.27E-05	7.617546057	0.173260455
POMT1	0.134319317	1.159777977	0.445206484	3.021260931	0.761558247
POMT2	0.83506881	0.862824273	0.044917299	16.57414276	0.922052301
POMZP3	0.788206936	0.06417662	0.000553669	7.438810652	0.257449733
PON1	0.15862141	1.621699841	0.844375395	3.114622229	0.146514275
PON2	0.683596527	0.742548168	0.299407135	1.841565269	0.520658973
PON3	0.836832071	0.376018748	0.036405419	3.883765166	0.411621328
POP1	0.071268392	0.746635435	0.46322842	1.203433228	0.230277066
POP4	0.427813975	0.614479251	0.309649749	1.219393045	0.163710645
POP5	0.185425762	0.513009047	0.119838026	2.196116635	0.368316479
POP7	0.379308131	1.73276728	0.514422958	5.836602741	0.374975991
POPDC2	0.283816614	0.860658184	0.379115445	1.953844189	0.719796349
POPDC3	0.278230315	0.714973269	0.342424648	1.492844566	0.371735752
POR	0.961647233	0.846649632	0.390910569	1.833707389	0.672886285
PORCN	0.115719521	0.020100295	8.84E-05	4.569689906	0.158197003
POSTN	0.705236958	0.435562569	0.07630646	2.486221361	0.349699419
POT1	0.648093725	60.01104735	0.443977633	8111.502787	0.101921565
POU1F1	0.698383893	1.195636377	0.535609334	2.669009395	0.662761729
POU2AF1	0.489610974	1.182812241	0.059727672	23.4237291	0.912243674
POU2F1	0.705078923	39.71480533	9.50E-05	16594078.91	0.577163829
POU2F2	0.346857649	1.213612103	0.591335949	2.490723488	0.597659639
POU2F3	0.150121318	0.255177977	0.000313506	207.7016491	0.689579855
POU3F1	0.995073082	1.026250784	0.277912714	3.789645519	0.968988758
POU3F2	0.168051212	0.486602383	0.140012935	1.691142891	0.257084367
POU3F3	0.081686729	69.71193757	0.732965295	6630.265133	0.06780618
POU3F4	0.811088684	3.25006541	0.00523923	2016.121683	0.719397446

POU4F1	0.959354483	0.945080097	0.743653694	1.201064954	0.644168073
POU4F2	0.996243024	0.02145856	1.67E-07	2762.052196	0.522193118
POU4F3	0.577344808	0.807391761	0.196586762	3.315998738	0.76659969
POU5F1	0.830186662	0.690903426	3.79E-05	12597.95327	0.941116663
POU6F1	0.288728369	0.82278251	0.234124015	2.891506275	0.760983901
POU6F2	0.224387114	0.00657234	2.39E-06	18.05158957	0.213571425
PPA1	0.289925442	0.85043234	0.294020212	2.459814445	0.764963186
PPA2	0.265529714	1.235576734	0.532721208	2.865757628	0.6221401
PPAN	0.681933462	0.276635427	2.19E-05	3493.338616	0.789697312
PPAP2A	0.26994893	1.417930982	0.147042137	13.67314374	0.762647334
PPAP2B	0.075528509	0.416696139	0.155414562	1.117241975	0.081920603
PPAP2C	0.222690079	0.14670435	0.000250647	85.86630061	0.554951405
PPAPDC1A	0.140213707	0.330468767	0.02454379	4.449582009	0.403909998
PPAPDC1B	0.266249504	0.622197261	0.155378942	2.491518013	0.502652104
PPAPDC2	0.764634292	1.194036463	0.482703947	2.953618017	0.701147303
PPAPDC3	0.381179846	0.484674444	0.11874625	1.978246192	0.312834315
PPARA	0.459065498	20.95318481	0.199201853	2203.975252	0.200284707
PPARD	0.944280934	0.599836195	0.253374341	1.420046952	0.245076835
PPARG	0.815089945	501.9940113	0.094366691	2670412.454	0.155410673
PPARGC1A	0.68319088	0.212323213	8.52E-06	5288.515437	0.764149427
PPARGC1B	0.568981542	2.408895169	0.784962236	7.392426877	0.124354787
PPAT	0.343523097	0.006131193	8.24E-06	4.562034454	0.13102604
PPBP	0.670097737	0.807192889	0.392210024	1.661253717	0.560807032
PPCDC	0.340411687	1.218379474	0.871297947	1.703720924	0.248246793
PPCS	0.809662181	1.062801472	0.904786535	1.248412664	0.458304127
PPEF1	0.81006002	2.132284232	0.09339839	48.68002575	0.635188748
PPEF2	0.598249874	0.461242482	0.196568524	1.082292441	0.075364836
PPFIA1	0.501711959	1.118189192	0.419602979	2.979833629	0.823238756
PPFIA2	0.394067975	0.001597142	1.52E-07	16.7762913	0.172863494
PPFIA3	0.938675669	1.67081001	0.253511521	11.01175234	0.593661983
PPFIA4	0.104931266	24.46523284	0.054865295	10909.40307	0.304291568
PPFIBP1	0.720919442	1.0982261	0.318851367	3.782641977	0.881956239
PPFIBP2	0.85429703	1.03128246	0.549505541	1.935455485	0.923599753

PPHLN1	0.378526097	0.856777443	0.199892818	3.672305961	0.835098617
PPIA	0.973514736	0.068641319	0.000733063	6.427320255	0.247419696
PPIB	0.531099349	0.008815871	7.73E-08	1005.473264	0.425830891
PPIC	0.287329688	1.229087244	0.875536564	1.72540533	0.233295193
PPID	0.345700232	0.785440291	0.497143452	1.240922411	0.300690436
PPIE	0.437372548	0.623408401	0.116266946	3.342635619	0.58127196
PPIF	0.146055915	1.737263403	0.003348772	901.2509167	0.862525047
PPIG	0.233374943	2.298172605	0.281889035	18.7364412	0.437020626
PPIH	0.490540512	1.040802571	0.803450102	1.348272891	0.762016705
PPIL1	0.217413946	0.322669525	0.064565084	1.612568528	0.168236372
PPIL2	0.690553488	0.425256082	0.025888502	6.985446047	0.549325978
PPIL3	0.405930765	1.535608052	0.374331978	6.299467394	0.551457662
PPIL4	0.995915284	0.468851719	0.051616631	4.258742368	0.50104085
PPIL6	0.716842607	1.510558821	0.148271758	15.38922839	0.727622835
PPL	0.39515025	4.147872862	0.18003362	95.5646466	0.374130047
PPM1A	0.705478496	0.932026846	0.812851501	1.068674954	0.313239226
PPM1B	0.45502863	0.03510653	0.000575077	2.143136351	0.110354957
PPM1D	0.500174769	1.003764804	0.688112047	1.46421471	0.984436789
PPM1E	0.845486557	3.516540672	0.032256923	383.3613782	0.599350071
PPM1F	0.525822864	0.975354851	0.668935445	1.422135861	0.896809794
PPM1G	0.880568553	1.151181798	0.70498362	1.879787692	0.573624706
PPM1H	0.524164778	0.888958552	0.270442548	2.922052436	0.846282038
PPM1J	0.459335547	0.537924745	0.16184179	1.787937664	0.311642837
PPM1K	0.725966584	1.671046902	0.648830971	4.303736834	0.28744142
PPM1L	0.127800295	3.795433299	0.945604408	15.23397502	0.059960576
PPM1M	0.576918588	0.299058173	0.011212406	7.976503177	0.471205969
PPME1	0.198826739	0.770280607	0.33017162	1.797041835	0.545939825
PPOX	0.188805067	0.990170919	0.346573113	2.828951267	0.985286372
PPP1CA	0.702345841	0.926421136	0.2160837	3.971868861	0.918039515
PPP1CB	0.835485819	1.022941497	0.881719184	1.186782964	0.76475586
PPP1CC	0.78084165	0.695717959	0.271514256	1.782681639	0.449806277
PPP1R10	0.567469471	116.1100009	0.001016749	13259450.96	0.423602507
PPP1R11	0.632390655	0.453610311	0.120536449	1.707054714	0.242365701

PPP1R12A	0.143254387	0.877704689	0.461879095	1.667894327	0.690459265
PPP1R12B	0.545502379	0.646157103	0.272352548	1.533009346	0.321814919
PPP1R12C	0.986969238	0.911985284	0.403163589	2.062976869	0.824924483
PPP1R13B	0.64942674	0.886452345	0.669101733	1.174407001	0.401016602
PPP1R13L	0.132388841	1.861430756	0.765755541	4.524844127	0.170360662
PPP1R14A	0.869159983	1.768008254	0.744363532	4.19936369	0.196673568
PPP1R14B	0.360464144	0.608653148	0.241738066	1.532479601	0.2919433
PPP1R14C	0.299401429	46.53178367	0.006173221	350741.8168	0.399197022
PPP1R14D	0.541464798	0.393441037	0.010715058	14.44657188	0.611874831
PPP1R15A	0.102406255	0.894758487	0.182296713	4.391701513	0.891033262
PPP1R15B	0.943810162	20.73264169	0.00079953	537618.6877	0.55877455
PPP1R16A	0.432241589	1.072084158	0.804082881	1.429410412	0.635319475
PPP1R16B	0.687787919	8.490333306	0.001200147	60064.08433	0.636258472
PPP1R1B	0.98096708	0.94030752	0.812125359	1.088721368	0.410430554
PPP1R1C	0.574606258	1.171292856	0.720228094	1.904850652	0.523969403
PPP1R2	0.183334886	58.7125233	0.297061446	11604.19985	0.131058647
PPP1R2P9	0.992829139	1.190447504	0.172535171	8.213776079	0.859587058
PPP1R3A	0.189292809	1.39468734	0.988170117	1.968439183	0.058454637
PPP1R3B	0.067524997	0.431899127	0.181455711	1.028002124	0.057756085
PPP1R3C	0.257365654	65224.2281	0.135387203	31422467004	0.096823574
PPP1R3D	0.096274986	2.245989661	0.676021333	7.461997589	0.186555986
PPP1R3E	0.220078639	1.047377094	0.793456536	1.38255686	0.743845658
PPP1R3F	0.205767063	1.57209802	0.481205509	5.136042996	0.453862648
PPP1R3G	0.070371696	0.475989413	0.176929961	1.28054016	0.141500195
PPP1R7	0.05863319	0.862302828	0.653935188	1.13706401	0.293821097
PPP1R8	0.512616143	0.111553604	0.002808479	4.43094141	0.242995674
PPP1R9A	0.953389663	1.376511354	0.563605396	3.361897387	0.483057894
PPP1R9B	0.642085477	0.890707734	0.331095936	2.396164322	0.818693109
PPP2CA	0.730420477	0.720831082	0.297287256	1.747795907	0.468827341
PPP2CB	0.112340156	0.166190524	0.00897164	3.078510921	0.228214367
PPP2R1A	0.132376218	1.453881324	0.990368669	2.134327318	0.056061697
PPP2R1B	0.272717059	1.027248251	0.270342429	3.903342044	0.968515383
PPP2R2A	0.084615367	1.827195467	0.733990465	4.54861941	0.195192501

PPP2R2B	0.599013303	1.065088098	0.037536313	30.22173928	0.970531048
PPP2R2C	0.234911045	1.37709349	0.942648381	2.011764428	0.098013702
PPP2R2D	0.677203771	0.660850528	0.241111901	1.811289357	0.420695721
PPP2R3A	0.283868002	0.966056489	0.338192136	2.759570789	0.948584509
PPP2R3B	0.175768801	0.458353346	0.190268752	1.104163389	0.082022084
PPP2R4	0.738352688	0.809635702	0.344313036	1.903819784	0.628343311
PPP2R5A	0.61346648	0.764031452	0.403560367	1.446485107	0.408541568
PPP2R5B	0.942456099	1.186433833	0.120217373	11.70900019	0.883643274
PPP2R5C	0.399830419	2.191838201	0.047139619	101.9133128	0.688713345
PPP2R5D	0.094681659	0.026356869	0.00039047	1.77909639	0.090666189
PPP2R5E	0.350509349	0.971562414	0.707841354	1.333538255	0.8582903
PPP3CA	0.082596284	1.085753015	0.768324558	1.534325041	0.641002333
PPP3CB	0.937720994	1.029243929	0.710728304	1.490503557	0.878737652
PPP3CC	0.360391115	1.789742102	0.379345211	8.443962652	0.462113873
PPP3R1	0.223157541	164.4936458	0.138358924	195564.9757	0.157809061
PPP3R2	0.771113891	1.145860207	0.507088252	2.58928423	0.743406942
PPP4C	0.997108045	1.430464903	0.57739481	3.543900642	0.439275515
PPP4R1	0.066609967	6.339551248	0.314582015	127.7565406	0.228116111
PPP4R1L	0.968410596	0.897491572	0.63793646	1.26265102	0.534627671
PPP4R2	0.209119984	1.898051676	0.931007894	3.869569947	0.0778561
PPP5C	0.793973849	1.149099918	0.618985072	2.133218846	0.659718907
PPP6C	0.249302589	3.942905449	0.044989231	345.5605471	0.547767569
PPRC1	0.708161204	0.522560348	0.225801531	1.209333334	0.129521093
PPT1	0.851160217	4.399913595	0.001251899	15463.90079	0.72209489
PPT2	0.857770729	2038.984339	0.866741407	4796652.264	0.054372461
PPWD1	0.894224617	1.655481073	0.642379137	4.266355216	0.296641893
PPY	0.833854697	1.433782819	0.508866556	4.03982763	0.495402037
PPY2	0.288181008	7.672983554	0.236932031	248.4876205	0.25079717
PQBP1	0.945347835	0.952678202	0.004644339	195.419801	0.98576023
PQLC1	0.961610583	0.814107875	0.198607684	3.337089575	0.775088169
PQLC2	0.827805735	25.94601415	0.319938161	2104.143029	0.146552174
PQLC3	0.301619748	0.297070181	0.024108184	3.660611312	0.343503538
PRAF2	0.888922157	1.373928156	0.423652232	4.45572674	0.596656694

PRAM1	0.394667577	27.6158563	1.55E-05	49347158.84	0.651422478
PRAME	0.259859556	1.588777429	0.540532447	4.6698653	0.400006524
PRAMEF1	0.810259228	1.076456678	0.000618827	1872.508273	0.984559477
PRAMEF10	0.465214379	0.95310127	0.798129891	1.138163151	0.595734003
PRAMEF2	0.434783337	2.083743848	0.592295245	7.330783868	0.25266217
PRAMEF4	0.576078939	0.001606962	8.16E-09	316.3537132	0.300963187
PRAMEF5	0.780921261	1.304586506	0.852936447	1.995395973	0.220082926
PRAMEF7	0.864440698	0.830784659	0.341218025	2.022762867	0.683035373
PRAMEF8	0.567028325	0.996994304	0.773251862	1.285477205	0.981478582
PRAP1	0.305627459	0.129446427	0.001159487	14.45154035	0.395427982
PRB1	0.365804637	1.387119225	0.702719877	2.738075023	0.345611183
PRB2	0.817924762	0.457216264	0.184857005	1.130856317	0.090302391
PRB3	0.160846479	0.578265708	0.119467313	2.799018574	0.496038266
PRB4	0.239952548	1.371726267	0.622994181	3.020305824	0.432531829
PRC1	0.477042418	1.374047901	0.517511151	3.648245319	0.523605263
PRCC	0.252835658	0.124601928	0.00101302	15.32609472	0.396305285
PRCP	0.65273786	0.603066957	0.258281034	1.408116381	0.242442486
PRDM1	0.26586155	1.313993523	0.497110584	3.473229169	0.58189484
PRDM10	0.355583027	1.271819296	0.746974986	2.165433048	0.375854698
PRDM11	0.487747772	0.888395988	0.411458694	1.918169293	0.763161638
PRDM12	0.965705933	1.193450131	0.438216134	3.250275614	0.729371196
PRDM13	0.007597559	0.152062833	0.022966311	1.006827119	0.050830357
PRDM14	0.951384701	1.028127802	0.484517507	2.181648261	0.942390546
PRDM15	0.56976553	1.092117519	0.383488834	3.11018358	0.868924332
PRDM16	0.159248281	0.394536234	0.142515736	1.092222129	0.073426999
PRDM2	0.3897197	0.993057544	0.7046745	1.399459305	0.968250432
PRDM4	0.217851013	19.56791273	0.349589504	1095.293778	0.147569568
PRDM5	0.340410752	1.497365967	0.625866315	3.58240216	0.36437495
PRDM6	0.992990781	0.306809999	0.000313192	300.5584081	0.736689015
PRDM7	0.101011667	2041.413817	0.072527773	57458959.47	0.144835494
PRDM8	0.664528262	0.12467184	0.000679159	22.88576182	0.433702763
PRDM9	0.846847376	0.000906207	7.31E-09	112.3560902	0.24164696
PRDX1	0.911643894	1.162147279	0.892006384	1.514099363	0.265583588

PRDX2	0.070149921	0.693811131	0.439550336	1.095150761	0.116490062
PRDX3	0.744341744	0.891216949	0.470039576	1.689788882	0.724223461
PRDX4	0.938376639	0.951060771	0.255728852	3.537014236	0.940313928
PRDX5	0.257540116	2.122793546	0.859730542	5.241470696	0.102629407
PRDX6	0.23155964	0.81392464	0.125340268	5.285398956	0.829224585
PREB	0.674968035	29.33090457	0.062678041	13725.73156	0.281465404
PRELP	0.700723805	0.311423178	0.029396846	3.299142914	0.33267162
PREP	0.757647999	0.073651976	4.65E-07	11670.39278	0.669390531
PREPL	0.567069785	0.904881731	0.008607135	95.1316546	0.966433372
PREX1	0.867861426	0.050852867	7.38E-05	35.03102992	0.371646184
PRF1	0.86162641	1.221458774	0.522644306	2.854640373	0.644173505
PRG2	0.158609272	4.575068583	0.016455166	1272.017104	0.596399896
PRG3	0.685215177	1.175270761	0.419823574	3.29009957	0.758473967
PRG4	0.723774332	8.63226591	0.085028561	876.3645278	0.360513909
PRH1	0.978725137	1.693165918	0.278083229	10.30918274	0.567758424
PRH2	0.13320939	2.27951613	0.339613011	15.30033778	0.396314817
PRICKLE1	0.977811575	1.2187966	0.079170896	18.76276802	0.887202279
PRICKLE2	0.19308442	5832.776456	0.123906371	274572493.6	0.114205657
PRIM1	0.322684287	0.871436573	0.605098791	1.255004492	0.459634518
PRIMA1	0.728631289	1.072530869	0.153789377	7.479856473	0.943666873
PRKAA1	0.154415745	0.508323575	0.173120786	1.492558245	0.218239919
PRKAA2	0.996048136	0.772063222	0.112871603	5.281059224	0.792021375
PRKAB1	0.199938272	2.80865031	0.949942362	8.304205476	0.061885304
PRKAB2	0.343451813	0.052584018	0.000105439	26.22444821	0.35274014
PRKACA	0.76057764	0.799930323	0.037780408	16.93704629	0.886035856
PRKACB	0.491461262	0.310611411	0.069175258	1.394710351	0.127056935
PRKACG	0.791729159	0.580098393	0.23519723	1.430774274	0.237102366
PRKAG1	0.103911723	0.517637271	0.191955834	1.395885393	0.193260577
PRKAG2	0.832402189	1.030757817	0.709550953	1.497371926	0.87366392
PRKAG3	0.551426195	2.410978812	0.001850084	3141.921352	0.809960748
PRKAR1A	0.19037502	409.1222682	0.008929633	18744447.68	0.272079103
PRKAR1B	0.975322925	0.032607529	5.80E-05	18.33201271	0.289317089
PRKAR2A	0.92388186	1.773396329	0.008305389	378.6619129	0.834181411

PRKAR2B	0.338958197	0.637029204	0.200172837	2.027279089	0.445179723
PRKCA	0.998449034	1.32161365	0.400378449	4.36252911	0.647193219
PRKCD	0.175797918	1.307326028	0.596884397	2.863370785	0.502899914
PRKCDBP	0.874376891	0.937139111	0.265952543	3.30220461	0.919527199
PRKCE	0.42953877	1.036311132	0.675331474	1.590242428	0.870321423
PRKCG	0.136383693	2.055411555	0.568427541	7.43228707	0.271938021
PRKCH	0.082019742	0.513880916	0.148080012	1.783316953	0.294301533
PRKCI	0.111898826	1.431007308	0.750587817	2.728237616	0.276357413
PRKCQ	0.183241617	1.644842251	0.944087844	2.86573548	0.078944509
PRKCSH	0.766824611	0.357634218	0.003313053	38.60554793	0.666851224
PRKCZ	0.234049576	0.483923219	0.172409477	1.358287753	0.168075155
PRKD1	0.554017639	0.90729935	0.266544479	3.088385523	0.87630228
PRKD2	0.851707453	0.016694255	3.27E-08	8514.576631	0.541621469
PRKD3	0.463471871	0.807331306	0.001475322	441.7909315	0.946954415
PRKDC	0.405907947	0.905705719	0.759925889	1.079451116	0.26867562
PRKG1	0.911908664	2.670799757	0.022712026	314.0702394	0.686295864
PRKG2	0.506652657	1.337348096	0.395297777	4.524437111	0.640172653
PRKRA	0.954118933	0.994235399	0.575144862	1.71870444	0.983483637
PRKRIP1	0.391027905	1.619678127	0.683918134	3.83577669	0.27295796
PRKRIR	0.342947843	0.718283183	0.290951575	1.773252922	0.472980062
PRKX	0.38436238	1.428997492	0.340834784	5.991271812	0.625456352
PRKY	0.738768454	1.074491916	0.837193676	1.37905112	0.572551279
PRL	0.076689923	0.93664093	0.760849536	1.153048257	0.537115179
PRLH	0.687328425	0.806780302	0.073566384	8.847715811	0.860518343
PRLHR	0.968767079	0.300420203	0.023487665	3.842540278	0.355078944
PRLR	0.09264391	0.469234197	0.214318749	1.0273517	0.058427959
PRM1	0.605626251	0.068643196	0.000229804	20.50394573	0.356938433
PRM2	0.337766354	2.194627969	0.530635285	9.076652189	0.277862542
PRM3	0.77875184	1.82913877	0.730453401	4.580372456	0.197285777
PRMT1	0.063728711	130.9511023	0.045580895	376214.4502	0.2301993
PRMT2	0.236611885	1.072664208	0.901806744	1.275892547	0.428124817
PRMT3	0.317706317	0.450393889	0.079014504	2.567309094	0.369072067
PRMT5	0.16226936	1.521945783	0.814192015	2.844929601	0.188204901

PRMT6	0.126906086	0.306555628	0.032990175	2.848616383	0.298544559
PRMT7	0.276532855	0.271167994	0.01052917	6.983654115	0.431075343
PRMT8	0.801627567	1.077946233	0.478514084	2.428283972	0.856256282
PRND	0.445198318	64.85813665	0.308725284	13625.63453	0.126216702
PRNP	0.176041122	0.109191388	0.007304558	1.6322355	0.10851403
PRNT	0.715201047	1.068558927	0.81258079	1.405174962	0.635078785
PROC	0.331431502	1.369506586	0.775458946	2.418630021	0.278531761
PROCA1	0.428473724	0.655089225	0.181408389	2.365612167	0.518503458
PROCR	0.422577136	1.875430441	0.203139091	17.31443871	0.579233117
PRODH	0.051025041	0.067709659	0.002697645	1.699481588	0.101536503
PRODH2	0.969195158	1.376038251	0.525094834	3.605979621	0.516069762
PROK1	0.689580709	0.408913092	0.001018155	164.2282888	0.770029798
PROK2	0.400934829	0.020977711	6.45E-05	6.824407389	0.19044214
PROKR1	0.326492855	1.333368508	0.228321531	7.786701383	0.749315718
PROKR2	0.697905682	0.881521556	0.546045181	1.423106148	0.605816727
PROL1	0.193051683	1.201407349	0.367231879	3.93043115	0.761562894
PROM1	0.853615415	0.864892851	0.326980919	2.287716501	0.769926189
PROM2	0.831151849	0.954074072	0.384933106	2.364715636	0.91913869
PROP1	0.967001423	0.706241709	3.91E-05	12769.63389	0.944560068
PROS1	0.959738148	0.948615517	0.692033683	1.300328902	0.7430305
PROSC	0.391626847	0.77046797	0.34381771	1.726557052	0.526478277
PROX1	0.145343	0.535288876	0.187457619	1.52852779	0.24305728
PROZ	0.097779735	0.383104245	0.105339067	1.393299438	0.145262262
PRPF18	0.661645775	1.009617628	0.476402834	2.139634112	0.980072427
PRPF19	0.404167341	0.380554262	0.053144733	2.725040416	0.336108153
PRPF3	0.80265435	14.03324584	0.002691706	73162.50736	0.54526396
PRPF31	0.75177179	15.2745695	0.003446723	67691.09731	0.52454002
PRPF38A	0.098835814	1.357907825	0.961366439	1.918013347	0.082500912
PRPF38B	0.255556271	0.048885775	1.11E-06	2158.651473	0.580194115
PRPF39	0.235648161	48.14192844	0.071601708	32368.57539	0.243513838
PRPF4	0.540901958	0.622517775	0.282492858	1.371816557	0.239691003
PRPF40B	0.995434786	1.768922175	0.744202658	4.204615004	0.196646898
PRPF4B	0.783092807	0.82659578	0.545158647	1.253324306	0.369862272

PRPF8	0.45411324	0.186067958	9.68E-05	357.8120694	0.662924968
PRPH	0.850923174	0.498580147	0.071121567	3.495172745	0.483620923
PRPS1	0.169093393	0.003374666	6.52E-06	1.74565283	0.074226908
PRPS1L1	0.302452554	0.56423456	0.153391329	2.07548003	0.389142848
PRPS2	0.597949758	1.17831149	0.062051458	22.37526756	0.913010257
PRPSAP1	0.845149192	4.656584283	0.000319459	67876.47248	0.753155964
PRPSAP2	0.118528487	1.659596152	0.265812217	10.36167343	0.587753446
PRR11	0.509606297	0.939635131	0.577190519	1.52967547	0.802262559
PRR3	0.518043447	0.136908146	0.001156487	16.20756642	0.414289393
PRR4	0.449206234	0.813667118	0.58896484	1.124097967	0.211106059
PRR5	0.766911191	0.947077646	0.479618623	1.87014437	0.87553413
PRR7	0.656241421	0.122071757	0.000843689	17.66234025	0.407313367
PRRG1	0.964111453	0.386168307	0.008375948	17.80406943	0.626403051
PRRG2	0.752335024	0.723373855	0.217089943	2.410382197	0.597969416
PRRG3	0.55990697	0.465530121	0.056844456	3.812478949	0.476078713
PRRG4	0.068709978	0.267336127	0.065900215	1.084497288	0.064829431
PRRT1	0.819623519	0.823173554	0.251415023	2.695203697	0.74778873
PRRT2	0.10804841	0.788247721	0.584357734	1.063277567	0.119192438
PRRT3	0.493855916	5.678400758	0.653458904	49.34393728	0.115424026
PRRX1	0.724991029	0.109831136	0.00646522	1.865811071	0.126414425
PRRX2	0.841191592	3.017826105	0.01471172	619.0489216	0.684266318
PRSS1	0.854484426	0.178287012	0.002523319	12.5970034	0.427335461
PRSS12	0.605299171	0.77683798	0.262539159	2.298618041	0.648221809
PRSS16	0.433548733	1.004835177	0.839318547	1.202992281	0.958110198
PRSS21	0.79892206	0.71505569	0.000460076	1111.347005	0.928722281
PRSS22	0.636782043	0.845012481	0.399460534	1.787526006	0.659549018
PRSS23	0.468615971	1.757305741	0.137007816	22.53976133	0.66495984
PRSS27	0.831630757	0.998752597	0.344023567	2.899530281	0.998168567
PRSS3	0.511994469	14.39578372	0.001428827	145041.106	0.570671166
PRSS33	0.703673329	0.228463231	2.38E-05	2190.129617	0.752289535
PRSS35	0.90442596	0.82348783	0.432526913	1.567838177	0.554426392
PRSS36	0.251026919	0.542111962	0.128288963	2.290807977	0.405023566
PRSS8	0.478061991	1.203044733	0.064368575	22.48483247	0.901520747

PRTFDC1	0.756144278	6.081547201	0.0001801	205359.0555	0.734363884
PRTG	0.77159701	1.185459594	0.389807333	3.605151394	0.764328196
PRTN3	0.395935399	1.43728435	0.404048496	5.112718703	0.575285314
PRUNE	0.759813081	88.55767299	0.007302794	1073898.709	0.350015625
PRX	0.495299914	9.511533248	0.000617687	146464.5225	0.647043531
PRY	0.910175753	1.13625587	0.958615248	1.346815007	0.140835111
PSAP	0.379004166	0.165283639	0.003719427	7.34486268	0.352424423
PSAT1	0.175137124	0.622325068	0.313010963	1.237300082	0.176156963
PSCA	0.663136194	0.806369213	0.076885577	8.457129858	0.857563249
PSD	0.351917937	0.53507978	0.185367684	1.544553858	0.247605309
PSD2	0.775829683	1.024921636	0.816784509	1.286097311	0.831682258
PSD3	0.789009074	1.003777298	0.562203313	1.792178808	0.989828993
PSD4	0.95627198	0.284493611	0.000299615	270.135366	0.719325645
PSEN1	0.836106137	1.599213161	0.001079633	2368.845507	0.899694555
PSEN2	0.294676092	1.650672257	0.469435258	5.80424852	0.43467872
PSENEN	0.989536641	2.013693102	0.27617624	14.68250819	0.489845076
PSG1	0.194336163	0.307572199	0.050180083	1.885223235	0.202466497
PSG11	0.705539597	1.319223084	0.618010303	2.816052638	0.473945428
PSG2	0.376052312	0.000426657	2.47E-08	7.36351321	0.119028146
PSG3	0.824355283	5.698123312	0.794949798	40.84359709	0.083342689
PSG4	0.086110033	0.681330586	0.304151782	1.526249048	0.351095767
PSG5	0.741424985	1.614236779	0.476300683	5.470830661	0.441924516
PSG6	0.094486934	0.883891513	0.466344504	1.675294124	0.705193895
PSG7	0.622214424	0.150725064	0.001986718	11.43496309	0.39158464
PSG8	0.225752396	3.064784624	0.276251201	34.00131745	0.361668466
PSG9	0.556113267	6.14278289	0.229911358	164.1231728	0.278827773
PSIP1	0.829546194	0.355487031	0.041609746	3.037053595	0.344669763
PSKH1	0.885825583	1.0898039	0.370400159	3.206457966	0.875885369
PSKH2	0.384911484	0.98488468	0.405476195	2.392243602	0.973166535
PSMA1	0.823858437	0.283073819	0.010962613	7.309460425	0.446768841
PSMA2	0.175197302	0.004605587	1.27E-06	16.72560625	0.198286975
PSMA3	0.096879215	1.13712653	0.834253419	1.549956783	0.416107481
PSMA4	0.289974807	14639.77705	0.167091754	1282666959	0.098569355

PSMA5	0.42614845	0.442897574	0.035984886	5.451129123	0.524850383
PSMA6	0.794964247	1.112247432	0.850949171	1.45378172	0.436196928
PSMA7	0.573266541	0.058676657	0.000383018	8.988994006	0.269344635
PSMA8	0.920890729	1.179999322	0.803478873	1.732962056	0.398616181
PSMB1	0.7041677	0.905164048	0.332991883	2.460486266	0.845166404
PSMB10	0.808665047	0.617236498	1.20E-05	31741.33628	0.930530595
PSMB2	0.062028919	16.89893385	0.311031593	918.1509913	0.165434907
PSMB3	0.21219094	20.07626568	0.711179605	566.7435355	0.078410936
PSMB4	0.803098778	1.461890597	0.0010526	2030.32915	0.918080633
PSMB5	0.555369303	0.147136251	0.009904444	2.185794223	0.163930374
PSMB6	0.405220985	1.039563065	0.814308276	1.327128066	0.755501305
PSMB7	0.132459685	2.305997385	0.208201147	25.54080038	0.495889453
PSMB8	0.271968519	0.680012359	0.353646056	1.307569535	0.247656446
PSMB9	0.732515939	0.01458236	2.52E-05	8.447106929	0.192723272
PSMC1	0.624200269	0.742219103	0.467017526	1.179589986	0.207236348
PSMC2	0.553125581	1.050437506	0.486165448	2.26963672	0.90037781
PSMC3	0.565683864	3.758505306	0.030725174	459.765081	0.589280359
PSMC4	0.224040054	1.322262483	0.476472727	3.669418987	0.591677327
PSMC5	0.640016409	0.953380755	0.311249178	2.920280367	0.93338375
PSMC6	0.516661624	0.988823525	0.705882993	1.385175694	0.947891714
PSMD1	0.670179483	1.063045825	0.844868858	1.337564304	0.60191591
PSMD10	0.580748304	0.807268323	0.259802372	2.508376427	0.711287355
PSMD11	0.962123553	0.290354029	1.17E-07	718687.611	0.869227625
PSMD12	0.659095618	0.741716806	0.406544265	1.353219975	0.33007989
PSMD13	0.860943841	3.291309467	0.002481928	4364.638243	0.745378445
PSMD14	0.443530611	1.095046493	0.786338285	1.524950324	0.591011315
PSMD2	0.664094881	0.832996856	0.328043703	2.115217444	0.700746106
PSMD3	0.8379382	1.176353009	0.368808945	3.752095557	0.783737477
PSMD4	0.43186902	1.236195858	0.563769558	2.710646889	0.59658912
PSMD5	0.450861749	0.564486237	0.231235594	1.378008923	0.209184691
PSMD6	0.695951748	1.609196128	0.609256594	4.250281748	0.337043562
PSMD7	0.860991553	0.242085143	0.032045263	1.828826197	0.16917757
PSMD8	0.298602054	1802.599114	0.428089844	7590377.606	0.078287932

PSMD9	0.405111526	0.062440236	0.001363802	2.858761058	0.15514659
PSME1	0.242976614	2.967291824	0.724030528	12.16084188	0.13071968
PSME2	0.339223899	0.28984302	0.010660395	7.880474767	0.462396755
PSME3	0.635184233	0.059283723	4.75E-06	739.8864109	0.557119186
PSME4	0.245725913	1.150593912	0.941031584	1.406824566	0.171476253
PSMF1	0.319107033	29.68261518	0.894980553	984.4433395	0.057714769
PSORS1C1	0.697485858	7.028127435	0.001193337	41391.99089	0.659756259
PSORS1C2	0.735723323	4.053860469	0.007040224	2334.270187	0.666015542
PSPC1	0.054834111	0.000795052	3.75E-08	16.8425057	0.160222956
PSPH	0.593549936	1.044399378	0.450855371	2.419334734	0.919267383
PSPN	0.11283379	1.507841651	0.663359309	3.42738304	0.326953185
PSRC1	0.469848667	1.152146695	0.393639609	3.372226719	0.796043703
PSTPIP1	0.254158763	2.969895103	0.641207226	13.75573538	0.16399441
PSTPIP2	0.05950383	0.124613275	0.007016504	2.21313477	0.155968801
PTAFR	0.413204934	0.603802514	0.092573017	3.938269366	0.597985773
PTBP1	0.619426729	1.046721092	0.732606222	1.495516978	0.801949103
PTBP2	0.92066171	0.969613326	0.725089658	1.296598277	0.835134425
PTCD1	0.814905269	0.949891464	0.599642567	1.504719383	0.826626535
PTCD2	0.432981573	0.520178246	0.040567225	6.670049756	0.615586834
PTCH2	0.289896981	1.557026121	0.398389484	6.085327143	0.524348607
PTCHD1	0.243372399	2.114462515	0.037524886	119.1463101	0.715832536
PTCHD2	0.109366982	0.480436301	0.172056919	1.341527214	0.161759761
PTCHD3	0.073354118	1.25223819	0.952062684	1.647055925	0.10769452
PTCRA	0.6087393	1.045119857	0.861363969	1.268076627	0.654650905
PTDSS1	0.526439632	0.714962487	0.177376381	2.881845685	0.63709577
PTDSS2	0.976495364	1.146165187	0.767800465	1.710984425	0.504532899
PTEN	0.810756718	1.394764061	0.59611193	3.263425354	0.44298389
PTER	0.318947514	1.277074798	0.758369877	2.150560155	0.357684169
PTF1A	0.750125124	7.808081322	0.154469505	394.6807094	0.304516318
PTGDR	0.269976646	2.660096688	0.881500666	8.027350023	0.082538686
PTGDS	0.516203884	1.229908206	0.820072168	1.844562288	0.316962491
PTGER1	0.988711007	1.470698193	0.122234838	17.69506315	0.761184125
PTGER2	0.445642942	1.206749158	0.554875788	2.624449581	0.635439198

PTGER3	0.445474088	1.051352969	0.480114811	2.302247377	0.900346949
PTGER4	0.070515863	0.689107117	0.314966099	1.507681684	0.351259305
PTGES	0.925704056	1.194714917	0.385679367	3.700855823	0.757779321
PTGES2	0.160908514	2.081873045	0.480784651	9.014837235	0.326787424
PTGES3	0.16392851	1.031258168	0.441168476	2.41062874	0.943360079
PTGFR	0.101300889	1.000593338	0.574971676	1.741280606	0.998325713
PTGFRN	0.986946225	0.938924172	0.311699383	2.828297548	0.910811813
PTGIR	0.891445052	0.988579911	0.276644002	3.532663762	0.985896892
PTGIS	0.156192053	0.484148639	0.100945068	2.322054059	0.364515641
PTGS1	0.442425684	0.753883149	0.151081663	3.761805322	0.730484428
PTGS2	0.060115521	33.49879394	0.438804532	2557.332738	0.112384565
PTH	0.375786418	0.876953727	0.497968287	1.544371114	0.649296483
PTHLH	0.257955294	0.887837105	0.018942928	41.61208464	0.951673395
PTK2	0.494061427	0.820916045	0.49012123	1.374972377	0.453322053
PTK2B	0.06970129	0.436635818	0.130616856	1.459618945	0.178371813
PTK6	0.111740645	3.871921881	0.899350139	16.66956884	0.069134805
PTK7	0.189437844	0.742023358	0.129670778	4.246127543	0.737436796
PTMA	0.686609906	0.357954377	0.105883086	1.210120904	0.098314364
PTMS	0.260876292	69.62517179	0.367554614	13188.96393	0.112766333
PTN	0.615347491	0.387308848	0.000975997	153.6973214	0.75602728
PTOV1	0.126566632	0.049649735	0.000118444	20.81227998	0.329728032
PTP4A1	0.102830913	0.740602715	0.443745339	1.23605215	0.250534946
PTP4A2	0.692115002	1.374411738	0.415223543	4.549375047	0.602540877
PTP4A3	0.304577894	3.209600469	0.955093449	10.7858924	0.059339605
PTPDC1	0.43076888	1.203416287	0.563413361	2.570423174	0.632501598
PTPLA	0.633339379	1.049869605	0.196686081	5.603986728	0.954583603
PTPLAD1	0.408560518	24.8405615	0.058899766	10476.33187	0.297558955
PTPLAD2	0.705506577	49.92183762	0.009724568	256277.6999	0.369669825
PTPLB	0.485833965	13.49825141	0.048950797	3722.161875	0.364026943
PTPN1	0.766458692	0.598348365	0.012208732	29.32497505	0.795918962
PTPN11	0.146600963	79.83232368	0.002139369	2979008.554	0.414808297
PTPN12	0.629266034	40.57870687	0.001077133	1528717.339	0.490916431
PTPN13	0.637608312	0.530004123	2.57E-05	10950.65378	0.900338687

PTPN14	0.198088908	10.18267367	0.118684589	873.633584	0.306935365
PTPN18	0.84131979	0.909214248	0.162955837	5.072972931	0.913591648
PTPN2	0.402678618	3.499401166	0.067201637	182.2248535	0.534526454
PTPN21	0.312528863	1.37317116	0.655852663	2.875034503	0.400272812
PTPN22	0.995580521	0.750043162	0.273000592	2.06067225	0.576988158
PTPN23	0.376318191	0.672140956	0.173302584	2.606847818	0.565642141
PTPN3	0.207250133	2.986435311	0.480885658	18.546604	0.240309012
PTPN4	0.090360905	4.098829712	0.829038789	20.26492032	0.083624046
PTPN5	0.671131369	0.812836774	0.305724284	2.161109396	0.677882075
PTPN6	0.506437296	1.200320157	0.00186253	773.554323	0.955879314
PTPN7	0.253035068	1.459483666	0.511767687	4.162225603	0.479497874
PTPN9	0.794070253	8.894812284	7.70E-05	1027516.541	0.713283024
PTPRA	0.671503308	0.151954414	0.003759602	6.141646096	0.318142318
PTPRB	0.408445667	10.191161	0.000880689	117930.1814	0.626745754
PTPRC	0.917426835	1.425587546	0.319047751	6.369892431	0.642473246
PTPRD	0.90262816	1.281176728	0.292113498	5.619096076	0.742540727
PTPRE	0.890476579	0.969076762	0.055982239	16.77513788	0.982773459
PTPRF	0.36545684	0.929033675	0.671306801	1.285706577	0.657021206
PTPRG	0.257937067	0.55535631	0.150111391	2.054611775	0.37823876
PTPRH	0.91530727	0.731304473	0.010393755	51.45457178	0.885351858
PTPRJ	0.240041079	520.7240786	0.021230093	12772132.55	0.225147176
PTPRK	0.256089318	0.828552761	0.294047168	2.334658354	0.721967076
PTPRM	0.882482075	0.92089245	4.98E-05	17029.16002	0.986883365
PTPRN	0.241694988	0.273481896	0.042695099	1.751778306	0.171219523
PTPRN2	0.471503673	1.710512345	0.260648673	11.2252729	0.576014335
PTPRO	0.884276229	2.006636059	0.48051313	8.37976742	0.33957885
PTPRQ	0.518473848	0.128344972	1.97E-05	834.7924967	0.64674523
PTPRR	0.795999616	1.069590376	0.418109346	2.73618273	0.888359094
PTPRS	0.811663744	18.08851446	0.00796881	41059.37585	0.462739289
PTPRT	0.148533913	0.856016091	0.65169632	1.124394178	0.263852691
PTPRU	0.199816651	28.00647848	0.189333542	4142.7569	0.191158863
PTPRZ1	0.486131505	0.453854866	0.172258454	1.195785957	0.109993539
PTRF	0.309019569	0.288013726	0.065274686	1.270812795	0.100273695

PTRH1	0.091413599	9.473181128	0.589059514	152.3465093	0.112617748
PTRH2	0.684132518	1.926013541	0.464813064	7.980688252	0.366158942
PTS	0.062218479	0.755093907	0.236242605	2.413480025	0.635622776
PTTG1	0.938697765	0.929056167	0.301681333	2.861116241	0.89797172
PTTG1IP	0.052369937	1.205661726	0.921372385	1.577668512	0.172845142
PTTG2	0.822630508	0.658202012	0.017688646	24.49197514	0.820686857
PTX3	0.466527663	1.113644512	0.858488619	1.444636623	0.417523969
PUM1	0.203969154	0.456479721	0.156306734	1.33310786	0.151526158
PUM2	0.121730463	7.00103587	0.44885951	109.1978719	0.165001386
PURA	0.668721254	0.941947817	0.737987651	1.20227715	0.63097828
PURB	0.489981815	0.266631016	3.00E-06	23679.8656	0.820126443
PURG	0.277700138	5.254130328	0.001641429	16818.20524	0.687047214
PUS1	0.198271284	0.030594761	7.67E-06	121.9982043	0.409766759
PUS3	0.595511045	2.869624173	0.002103348	3915.064011	0.774698507
PUS7	0.374551529	0.874988536	0.371127019	2.062918887	0.760229272
PUS7L	0.62073957	1.411155293	0.505179281	3.941886253	0.511102654
PUSL1	0.686429265	1.026074162	0.924973573	1.138225153	0.626716486
PVALB	0.409427595	0.68000749	0.413650453	1.11787666	0.128360593
PVR	0.282553579	11.51931317	0.807747179	164.2773622	0.071466712
PVRL1	0.094759818	0.019192446	0.000192634	1.912175969	0.09221056
PVRL2	0.187401284	0.530347456	0.15520069	1.812288489	0.311734597
PVRL3	0.5669677	1.257127902	0.362789849	4.356159822	0.718182986
PVRL4	0.63821169	1.126774413	0.909490223	1.395969464	0.274832712
PVT1	0.705480824	1.832968055	0.169091899	19.86950242	0.618260607
PWP1	0.371848187	2.075807101	0.538419859	8.00300183	0.288799144
PXDNL	0.668021332	1.326318263	0.993329649	1.770932878	0.055544743
PXK	0.580604563	1.330307438	0.854285069	2.071577677	0.206580865
PXMP2	0.89266636	0.324912039	0.016315266	6.47049409	0.461388292
PXMP4	0.666000569	1.159643747	0.000473667	2839.069958	0.970323514
PXN	0.097665354	1.230794596	0.864475141	1.75234112	0.249306098
PXT1	0.592812632	1.449288222	0.190772569	11.01015919	0.719842993
PYCARD	0.085483213	3.413157673	0.790653874	14.73419114	0.099933232
PYCR1	0.237291254	0.637876359	0.249267605	1.632327029	0.348321072

PYCR2	0.537029302	0.321124989	0.049338912	2.090059446	0.234600364
PYCRL	0.675651954	1.081829443	0.794122865	1.473770616	0.618048385
PYDC1	0.1564708	1.058112408	0.672799184	1.664095164	0.806837987
PYGB	0.762646234	0.210458577	1.02E-05	4354.400802	0.758555882
PYGL	0.256751992	0.167112454	0.014609805	1.911495254	0.150180413
PYGM	0.172292846	10.31085761	0.486963821	218.319678	0.134138003
PYGO1	0.12373711	0.243820049	0.004605977	12.90675434	0.485850074
PYGO2	0.646964152	0.001554138	4.75E-07	5.086324949	0.117333756
PYHIN1	0.469746392	0.590413397	0.279072886	1.249092967	0.168135689
PYY	0.726951842	0.239142387	0.027236067	2.09975549	0.196800514
PYY2	0.215673678	0.569058013	0.268612707	1.205553622	0.141047207
PZP	0.72154588	1.154997153	0.387678625	3.441041977	0.795859682
QARS	0.659745507	1.935928472	0.022056628	169.9180424	0.772319722
QDPR	0.785621765	1.444198522	0.001244326	1676.175798	0.918688219
QKI	0.076297699	12.56710517	0.001056528	149482.257	0.597043944
QPCT	0.970044947	0.990584699	0.597089176	1.643402837	0.970783317
QPCTL	0.516743033	0.884151753	0.693216672	1.127676748	0.321229977
QPRT	0.394252303	0.030493165	0.000357461	2.601213726	0.123913033
QRICH1	0.547824573	0.002266118	6.51E-09	788.9263149	0.349601311
QRICH2	0.687285929	0.60216932	0.104311294	3.476209296	0.570681195
QRSL1	0.282283532	2.916066127	0.818545213	10.38848133	0.098724106
QSER1	0.33514801	1.219537471	0.862093706	1.725185597	0.262086753
QTRT1	0.306440715	140.6650921	0.049837575	397023.0892	0.222398972
QTRTD1	0.831029332	1.060733668	0.541348632	2.078431251	0.86359553
R3HCC1	0.533211825	0.426308585	0.131959327	1.377235049	0.154158457
R3HDM1	0.249889963	9.50709469	0.069612846	1298.393253	0.369338707
R3HDM2	0.881940952	0.854967295	0.586608026	1.246094571	0.414927822
R3HDML	0.985317928	4.869293524	0.402005594	58.97932704	0.213545233
RAB10	0.220469149	1.260802013	0.08918613	17.8236427	0.863844755
RAB11A	0.615130898	1.142081072	0.591951432	2.203473298	0.691946816
RAB11B	0.253948326	1.761471485	0.751116922	4.130890546	0.192963926
RAB11FIP1	0.283379153	12.09219421	0.069227357	2112.187537	0.344029414
RAB11FIP2	0.53091627	0.391021825	0.002032901	75.2117781	0.726390726

RAB11FIP3	0.49857232	0.791792632	0.084249449	7.441420432	0.838179358
RAB11FIP4	0.276277064	0.057598494	0.000208589	15.9049029	0.319609715
RAB11FIP5	0.669826376	0.842694403	0.325419836	2.182208269	0.72442392
RAB12	0.522138698	1.132650735	0.658912228	1.946993292	0.652234135
RAB13	0.666438253	1.010562739	0.820643709	1.244434141	0.921196132
RAB14	0.919046427	0.38885928	0.006330344	23.8867821	0.653021872
RAB15	0.440207365	1.043405691	0.917340349	1.186795541	0.517798386
RAB17	0.45539587	0.000319272	9.87E-09	10.32734082	0.128690466
RAB18	0.129828537	1.62090655	0.604314079	4.347636661	0.337334096
RAB1A	0.561766597	0.614064127	0.006172751	61.08698835	0.83539915
RAB1B	0.460349589	3.557548628	0.155009197	81.64775074	0.427295604
RAB20	0.325112269	65.16237738	0.2486082	17079.62743	0.141539261
RAB21	0.307102785	1.172867221	5.19E-05	26497.37236	0.975131674
RAB22A	0.186684489	1.21228924	0.526613651	2.790746497	0.650891731
RAB23	0.156519814	0.009727326	5.04E-05	1.878584645	0.084496651
RAB24	0.24070054	2.175335633	0.004788731	988.1710671	0.803398571
RAB25	0.550888332	1.053321988	0.340487357	3.258526901	0.928161441
RAB26	0.497724889	1.415175843	0.381433339	5.250518143	0.603676674
RAB27A	0.341340278	0.742822916	0.31351792	1.759981963	0.499354099
RAB27B	0.974463206	14.564446	0.008241526	25738.32602	0.482598645
RAB28	0.81454558	8.927036505	0.000573404	138980.4628	0.656698622
RAB2B	0.96446726	0.713545653	0.037864219	13.446663	0.821753748
RAB30	0.472366407	0.764814666	0.33320516	1.755499445	0.527075595
RAB31	0.682816229	1.46243048	0.404531097	5.28686899	0.562120063
RAB32	0.745868995	0.662591822	0.066078981	6.643987503	0.726385694
RAB33A	0.128692279	19.87393628	0.042117926	9377.796698	0.341264687
RAB33B	0.812302111	0.96298349	0.430080793	2.156193016	0.926924658
RAB34	0.444792758	0.740781801	0.216176795	2.538467079	0.63301075
RAB35	0.779444632	0.953768984	0.082998823	10.96009854	0.969690322
RAB36	0.457405747	0.939123376	0.735781349	1.198661418	0.613918005
RAB37	0.608239656	0.869312042	0.256941867	2.94114554	0.821815029
RAB38	0.374698638	1907.956462	0.538037	6765887.588	0.070089091
RAB39B	0.428665934	0.040613953	7.10E-07	2322.799591	0.566503435

RAB3A	0.131174862	1.021400835	0.751747439	1.387779475	0.892300292
RAB3B	0.250850701	0.338156141	0.019479613	5.870217997	0.456536029
RAB3C	0.678734537	0.585338526	0.073634586	4.652992714	0.612615653
RAB3D	0.766715976	0.503782276	0.140415509	1.807468307	0.292870364
RAB3GAP1	0.771581114	0.032986627	5.47E-06	199.085768	0.442420409
RAB3GAP2	0.265779929	2.281303938	0.563053405	9.243079982	0.247949392
RAB3IL1	0.527852885	1.111869101	0.420925038	2.936990644	0.830569512
RAB3IP	0.410103897	3.292377521	0.62932279	17.22446719	0.158119673
RAB40A	0.268087621	3.686000054	0.206709085	65.72810495	0.37481329
RAB40B	0.384867602	1.417522494	0.00116791	1720.483034	0.923284252
RAB40C	0.819501201	36.73601218	0.843551818	1599.824174	0.061261241
RAB41	0.120244381	7.485989014	0.009542792	5872.498298	0.553871576
RAB42	0.898856339	4.678494566	0.009779736	2238.129073	0.62405675
RAB43	0.519104899	1.045214866	0.315776494	3.459643569	0.942273091
RAB4A	0.134536811	1.225691393	0.837226125	1.794400994	0.29536385
RAB4B	0.87455677	3.77368175	0.429503625	33.15612048	0.231012467
RAB5A	0.083625104	1.334687828	0.85590118	2.081305226	0.202822046
RAB5B	0.148941492	2.315878925	0.00139604	3841.791173	0.824306544
RAB5C	0.544728635	0.211597119	1.27E-08	3515211.011	0.85472893
RAB6A	0.130486945	0.513977674	0.127795988	2.067146665	0.348596275
RAB6B	0.18804825	0.077485011	0.00418929	1.433161113	0.085759989
RAB7B	0.524068327	1.425530822	0.654342663	3.105617652	0.372171475
RAB8A	0.808497943	2.468210731	0.822414622	7.407533929	0.107116452
RAB8B	0.002763785	0.484731937	0.25455773	0.923032472	0.027546263
RAB9A	0.771926575	0.520164682	0.065791244	4.112573054	0.535543341
RAB9B	0.685094851	0.720992666	0.329798912	1.576204185	0.412364671
RABAC1	0.105748879	1.1536311	0.931833794	1.428221129	0.18955941
RABEP1	0.980404682	0.008208426	2.17E-05	3.106864577	0.112812494
RABEP2	0.419355184	0.97678833	0.271412503	3.515370264	0.971327399
RABEPK	0.89002766	6.948269698	0.555809281	86.86154304	0.132526488
RABGAP1	0.175068378	1.200960064	0.875785594	1.646870063	0.255674149
RABGAP1L	0.54313076	0.774211297	0.510157391	1.174937664	0.229186764
RABGEF1	0.239162634	0.45419488	0.168702593	1.222820499	0.118318284

RABGGTA	0.861366794	1.051967705	0.631187448	1.753260549	0.845871718
RABGGTB	0.9895286	1.440349722	0.201236629	10.30929274	0.716331126
RABIF	0.173808881	1.250025072	0.765549855	2.041098529	0.372367638
RABL2A	0.592033481	0.428375844	0.040893955	4.487359146	0.479351757
RABL2B	0.172800832	104.8860272	0.025715371	427801.6728	0.272667056
RABL3	0.52322628	0.874596753	0.693496027	1.102990428	0.257677071
RAC1	0.76239453	0.67538755	0.065461671	6.968174458	0.741703311
RAC2	0.691995147	7.940197063	2.22E-06	28400916.7	0.78784219
RAC3	0.846088785	1.149631944	0.133663631	9.887907381	0.898935466
RACGAP1	0.222632373	0.810592802	0.446156887	1.4727122	0.490640776
RAD1	0.089987191	0.551219652	0.132264356	2.297241029	0.413420828
RAD17	0.874821786	0.823586047	0.453135533	1.496889842	0.52433057
RAD18	0.578285094	0.921766896	0.448099507	1.896128419	0.824809451
RAD21	0.629892214	1.741487978	0.710951323	4.26580594	0.224893406
RAD23A	0.086904349	0.212477965	0.033379199	1.352545524	0.10096735
RAD23B	0.557639397	3.334897648	0.12842641	86.59856151	0.468555066
RAD50	0.543487624	0.65740733	0.274212397	1.576093575	0.347116091
RAD51	0.440419538	1.588591592	0.302383088	8.345781704	0.584485505
RAD51AP1	0.377970071	0.808750861	0.375025703	1.744088335	0.58826186
RAD51C	0.49640361	2.434382157	0.54640249	10.84588118	0.243167022
RAD52	0.30608996	2.468191242	0.005765186	1056.681944	0.770103236
RAD54B	0.564119586	0.653071861	0.070388055	6.059307345	0.70775919
RAD54L	0.16977263	0.923561118	0.711140625	1.199432445	0.550974852
RAD54L2	0.988157304	0.331973161	0.017409684	6.330165448	0.463485945
RAD9A	0.260176978	0.851319326	0.435093494	1.665721514	0.638338981
RAD9B	0.374987654	1.110672696	0.856775122	1.439810525	0.427980768
RAE1	0.141681447	2.671013376	0.973956751	7.325081368	0.05630116
RAET1E	0.287108126	5.680935579	0.037919905	851.0841316	0.496719867
RAET1G	0.643878961	0.663458312	0.132764644	3.315468017	0.617202815
RAET1L	0.487432198	0.917871952	0.54932488	1.533680618	0.743531617
RAF1	0.298564897	0.778332913	0.170825571	3.546319908	0.74602833
RAG1	0.063909342	0.378458459	0.119682365	1.196757812	0.098091409
RAG2	0.374865205	0.935359372	0.571030395	1.532137626	0.790698622

RAI1	0.518618131	0.696254193	0.013611549	35.61460174	0.856888622
RAI14	0.783190643	3.621019755	0.426249032	30.7608536	0.238483915
RAI2	0.20197211	1.772165919	0.901583883	3.483394174	0.097015558
RALA	0.728553263	0.794416381	0.287110649	2.198098151	0.657607926
RALB	0.130130734	0.832449325	0.607144737	1.141361915	0.254769659
RALBP1	0.625514466	0.98363452	0.793009844	1.22008179	0.880660963
RALGDS	0.830814275	0.272136547	0.002537921	29.18070005	0.585320309
RALGPS1	0.279283355	0.484117832	0.064798023	3.616932499	0.479567698
RALGPS2	0.083287015	0.490267341	0.15226108	1.578617903	0.232189684
RALY	0.897903628	0.567124376	0.161726529	1.988727889	0.375614713
RAMP1	0.030887695	0.394994186	0.154770375	1.008076682	0.051999703
RAMP2	0.640283002	7.020939599	0.634224539	77.72262006	0.112115249
RAMP3	0.195599636	1.79704598	0.776410195	4.159366108	0.171024829
RAN	0.076452854	0.497966628	0.172121841	1.440669942	0.198320399
RANBP1	0.454746793	0.804196121	0.158694229	4.07533032	0.79241443
RANBP10	0.540124423	0.440563878	0.018872588	10.284574	0.610072315
RANBP17	0.395454079	0.905027508	0.248061022	3.301908473	0.879886237
RANBP2	0.33147063	1.112810203	0.936884497	1.321770776	0.223445771
RANBP3	0.594949906	1.112215391	0.215844411	5.731086893	0.898830941
RANBP6	0.14511708	0.639408934	0.272619929	1.499684147	0.303848877
RANBP9	0.205740037	1.173869651	0.260549998	5.288696873	0.834659456
RANGAP1	0.100237701	1.357372115	0.39829736	4.625837993	0.625244912
RAP1A	0.245978067	0.0610222	0.003462283	1.075506934	0.056102158
RAP1B	0.937682047	0.975850847	0.778048978	1.223939499	0.832485735
RAP1GDS1	0.093688261	1.049944015	0.853663314	1.291355053	0.644396222
RAP2A	0.487872945	0.898538761	0.000130922	6166.814955	0.981062685
RAP2B	0.948010995	1.859155159	0.651797459	5.302963153	0.246215657
RAP2C	0.065898143	0.249478509	0.045592311	1.365132081	0.109368297
RAPGEF1	0.113170579	0.202238584	0.020431791	2.001804213	0.17176567
RAPGEF3	0.955285912	1.025034861	0.879673885	1.194415889	0.75132163
RAPGEF4	0.267504318	2.513880315	0.502782623	12.56923757	0.261605211
RAPGEF6	0.58307351	0.0312739	0.000578267	1.691359175	0.088784422
RAPGEFL1	0.752960861	1.039537264	0.373737003	2.89143894	0.940778197

RAPH1	0.527188951	28.59267382	0.047736265	17126.20365	0.304113601
RAPSN	0.606304303	1.109665868	0.736804047	1.671215493	0.618441608
RARA	0.479603698	1.867351438	0.627169536	5.559902377	0.261913061
RARB	0.666646341	0.76695833	0.134629812	4.369203748	0.765031273
RARG	0.286677697	1.118433969	0.807631423	1.548843331	0.500433349
RARRES1	0.396259724	0.838142691	0.001126228	623.7487288	0.958260643
RARRES2	0.579647004	1.914808265	0.754686578	4.858295875	0.171472559
RARRES3	0.136383693	2.087419406	0.75500748	5.7712273	0.15609042
RARS	0.680308274	1.938100212	0.365306084	10.28242506	0.437044689
RASA1	0.644325584	0.872284428	0.238525169	3.189936424	0.836368656
RASA2	0.578256218	0.721922374	0.385255349	1.35279605	0.309196889
RASA3	0.596686523	0.141653266	3.73E-05	538.5460565	0.642158103
RASA4	0.627214166	1.023217682	0.434639588	2.408833558	0.958097172
RASAL1	0.386751954	1.053147644	0.684089104	1.621309203	0.814023818
RASAL2	0.487351313	1.069846052	0.778982848	1.469314219	0.676631566
RASD1	0.41950666	1.015119586	0.033358056	30.89112152	0.993129149
RASD2	0.326568185	0.837272024	0.344773722	2.033288496	0.694811993
RASEF	0.755751781	1.984474625	0.677163808	5.815637947	0.211546238
RASGEF1A	0.994639772	1.239681412	0.429129259	3.58122866	0.691403001
RASGEF1B	0.215171886	1.023157099	0.84021588	1.24593033	0.819820127
RASGEF1C	0.05836485	0.5613402	0.260436394	1.209903175	0.14056827
RASGRF1	0.366558853	1.56792137	0.010958586	224.3334551	0.859037357
RASGRF2	0.962267342	0.621419778	0.302902888	1.274872429	0.194424096
RASGRP1	0.206122272	1.22712576	0.699979873	2.15125847	0.474862872
RASGRP2	0.921655525	1.55758219	0.697885854	3.476302417	0.279329333
RASGRP3	0.958951837	0.777784067	0.188140059	3.215413339	0.728555654
RASGRP4	0.9350943	0.913995056	0.10963245	7.619887726	0.933760663
RASIP1	0.902423691	0.085285504	0.000236471	30.75897895	0.412522319
RASL10B	0.377864444	0.469891213	0.004989446	44.25295928	0.744666851
RASL11A	0.355024034	1.234248449	0.382532521	3.982326074	0.724733103
RASL11B	0.081469617	6.637740322	0.315174348	139.7943609	0.223469747
RASL12	0.243940116	178.5017353	0.016759363	1901198.153	0.273173952
RASSF1	0.500153173	1.201836285	0.255044232	5.663372355	0.816186595

RASSF2	0.615672171	0.942583108	0.73178096	1.214110457	0.647078561
RASSF3	0.487027194	1.052234334	0.792295127	1.397455388	0.725055715
RASSF4	0.801040348	1.141636488	0.43286812	3.010925985	0.788921681
RASSF5	0.292108432	1.441811777	0.000209863	9905.612821	0.935305101
RASSF6	0.56582707	1.148326024	0.435147131	3.030360455	0.779977279
RASSF7	0.965400694	0.912794198	0.767467989	1.085639089	0.302413477
RASSF8	0.636976917	1.712748756	0.716147144	4.09623682	0.226466842
RAVER1	0.400801437	1.126732988	0.954074029	1.330638073	0.159726719
RAVER2	0.397136495	3.044203105	0.443106093	20.91411669	0.25756136
RAX	0.065189502	0.757099061	0.184530161	3.10626179	0.699248566
RB1	0.528103635	0.648446647	0.240023078	1.751844273	0.392957187
RB1CC1	0.543069043	0.759364628	0.278805418	2.068233264	0.590255441
RBAK	0.345605066	0.81404705	0.182824855	3.624630787	0.787162749
RBBP4	0.207200759	7.757424254	0.005036207	11948.99976	0.584337895
RBBP5	0.073534857	1.581801332	0.309813847	8.076125318	0.581445419
RBBP6	0.181164653	1.807513218	0.699714128	4.669198324	0.221515101
RBBP7	0.466935809	0.714657228	0.233628882	2.186095096	0.555914319
RBBP8	0.411043434	9.08590211	0.000370131	223038.8273	0.668743737
RBBP9	0.253722176	1.98E-06	2.15E-12	1.828440038	0.060952051
RBKS	0.858200427	1.141741548	0.86755813	1.502578002	0.344139427
RBL1	0.855626234	0.583411887	0.20047974	1.697774698	0.322791612
RBL2	0.598448468	0.441108471	0.108207583	1.798179736	0.253638192
RBM10	0.127331012	370.9830438	0.020586775	6685283.11	0.236690848
RBM11	0.386677298	0.390025842	0.058628335	2.594652522	0.3301446
RBM12	0.134605575	3.898980771	0.898613938	16.91722152	0.069186576
RBM12B	0.085118786	1.290600738	0.694477867	2.398420952	0.419757646
RBM14	0.163651168	2.020299859	0.053546342	76.22577758	0.704198091
RBM15	0.380691559	1.057989853	0.173524466	6.450632335	0.951267414
RBM15B	0.980872466	0.763244956	0.261790153	2.225228327	0.62068719
RBM17	0.152081358	0.520614326	0.197190071	1.37450773	0.187576343
RBM18	0.583005606	0.959279417	0.456120858	2.017485025	0.912724456
RBM19	0.15842554	0.042680833	0.001219793	1.493412564	0.08206091
RBM20	0.153149455	0.295053372	0.052875683	1.646437202	0.164065815

RBM22	0.620094366	0.573315317	0.22598986	1.454447796	0.241500096
RBM23	0.665448372	0.003912641	1.03E-05	1.482968914	0.067266145
RBM24	0.817034838	1.044029763	0.820912539	1.327788399	0.725397544
RBM25	0.050785765	1.49957739	0.981935736	2.290101345	0.060712891
RBM28	0.558295595	0.941119351	0.820363311	1.079650469	0.386411886
RBM3	0.945868803	0.0980735	0.000897839	10.71284964	0.332212888
RBM33	0.132384185	0.687281493	0.365969074	1.290698818	0.243485395
RBM34	0.006276627	2.346832964	1.215157168	4.532438361	0.011077108
RBM4	0.389678659	0.869502264	0.470907835	1.605482284	0.65494134
RBM4B	0.229804843	1.01585596	0.85503254	1.206928722	0.858014539
RBM5	0.072644912	178.5328003	0.010471162	3043975.549	0.296992185
RBM6	0.728185235	0.10849459	0.000152463	77.20588166	0.507436372
RBM7	0.549428342	0.989305776	0.349478095	2.800535803	0.983842574
RBM8A	0.735287152	0.972398706	0.793800364	1.191180158	0.786910072
RBMS1	0.802919077	0.008888142	5.94E-07	132.9597339	0.335569272
RBMS2	0.801688401	0.918252411	0.077933846	10.81927218	0.945972194
RBMS3	0.480716023	0.627592965	3.54E-05	11123.27007	0.925636516
RBMX	0.101617167	1.718226494	0.619994232	4.761822178	0.297974134
RBMX2	0.959545108	1.093325394	0.416291089	2.871453291	0.856283858
RBMXL1	0.861847602	1.746247468	0.51817132	5.884888074	0.36847392
RBMY1A1	0.848675087	1.063777841	0.611515812	1.850521726	0.826747879
RBMY1A3P	0.946997999	0.632493908	0.025605923	15.62328173	0.779498472
RBMY1B	0.742870458	1.000475104	0.813902948	1.229815467	0.996400972
RBMY1D	0.769247315	1.082752584	0.862401795	1.359404821	0.493442043
RBMY1E	0.131857619	4.89E-05	3.65E-10	6.543017363	0.099335567
RBMY1F	0.379680081	1.106419989	0.920533252	1.329843533	0.281198984
RBMY1J	0.707079186	1.017006561	0.881553679	1.17327211	0.817127432
RBMY2EP	0.456212771	23.46530955	0.295693065	1862.136171	0.157366112
RBMY2FP	0.405328028	0.925114625	0.391026683	2.188692246	0.85938357
RBP1	0.288799265	0.96272718	0.205970685	4.499881242	0.961493024
RBP2	0.926194724	0.472389769	0.087439133	2.552084914	0.383553402
RBP3	0.758825246	0.030510969	0.000411331	2.263187757	0.112234178
RBP4	0.357335939	1.088041226	0.929042058	1.27425201	0.295174045

RBP5	0.245073204	0.71867031	0.354031715	1.458872166	0.360455436
RBP7	0.401948348	0.69580537	0.276711399	1.749639209	0.440761601
RBPMS	0.401171351	0.436069433	0.145163143	1.30995063	0.139173487
RBPMS2	0.558773574	22.84275609	0.00015937	3274084.89	0.605525852
RBX1	0.462437207	1.231132644	0.733676044	2.065881257	0.43108334
RC3H1	0.865394808	1.291728518	0.192725863	8.657699273	0.791997607
RCBTB1	0.670980059	0.268286549	0.023412601	3.074313362	0.290337178
RCBTB2	0.711863778	0.727246786	0.205558785	2.572927686	0.62128268
RCC1	0.106600138	0.365325769	0.12210888	1.092982899	0.071711025
RCC2	0.226521702	0.767495975	0.083299345	7.071485047	0.815331926
RCCD1	0.661360155	1.377125497	0.772193521	2.455957715	0.278310188
RCE1	0.284773986	0.997525818	0.642592733	1.548504531	0.991191009
RCHY1	0.333409412	0.856836464	0.523637041	1.402056516	0.538588506
RCL1	0.882326238	11.70507647	0.012710211	10779.42867	0.479929143
RCN1	0.257445967	0.027498564	0.000373293	2.025677459	0.101385522
RCN2	0.552535048	0.064842676	0.001278231	3.289369131	0.172061215
RCN3	0.511541219	0.599436802	0.24311284	1.478015232	0.266376669
RCOR1	0.322934167	0.027121429	5.24E-05	14.03360596	0.257856783
RCOR2	0.077708515	1.810372844	0.772060244	4.24507007	0.172247474
RCOR3	0.903380663	0.913437119	0.418804189	1.992261282	0.819985541
RCSD1	0.580221516	2.12574548	4.81E-05	93931.8587	0.890094333
RDH10	0.605453495	1.642255705	0.621236316	4.341349227	0.317227726
RDH11	0.851559049	0.540819011	0.051477147	5.681845578	0.608491662
RDH12	0.657958336	0.534807595	0.12403507	2.305953979	0.401249131
RDH13	0.20349579	0.69585826	0.389265715	1.243928503	0.221147528
RDH14	0.794774132	0.789209892	0.520946404	1.195616762	0.264011522
RDH16	0.611026299	0.675988184	0.32980187	1.38555923	0.284894914
RDH5	0.619297424	0.978981664	0.715311136	1.339843673	0.894446688
RDH8	0.876903701	1.213030886	0.633580329	2.32242679	0.560039103
RDX	0.534329912	3.03192975	0.165014285	55.70789232	0.455160709
RECK	0.280678408	0.824436815	0.535882319	1.268368143	0.379754325
RECQL	0.316714707	0.078791072	7.12E-07	8714.033948	0.668053101
RECQL4	0.420971046	147.4233263	0.000865339	25115752.73	0.416525025

RECQL5	0.96725532	1.751211543	0.096073692	31.92072455	0.705208221
REEP1	0.548777669	0.729630511	0.313953195	1.695668944	0.46379044
REEP2	0.332114282	105.2387896	0.026470976	418390.4211	0.270842739
REEP3	0.158288326	0.540915485	0.082241181	3.557701368	0.522561229
REEP4	0.206629514	1.330424039	0.428671136	4.129104984	0.621256828
REEP5	0.998603579	1.172577535	0.190613563	7.213222664	0.863627761
REEP6	0.672236747	0.622995111	0.184390049	2.104901588	0.446175855
REG1A	0.645236057	0.800798412	0.464420669	1.380812997	0.42419649
REG1B	0.999734939	0.432409726	0.007692663	24.30603963	0.683397137
REG3A	0.723464316	0.520431175	0.00647713	41.81614714	0.770421749
REG3G	0.595110781	0.17468727	1.69E-05	1810.831543	0.711500794
REG4	0.483909092	0.664366679	0.026331103	16.76280277	0.803917293
REL	0.968263555	0.256079433	0.006769738	9.686737425	0.462386122
RELA	0.785175376	0.338496122	8.25E-05	1388.21107	0.798558954
RELB	0.447241688	1.185365562	0.509631766	2.757072088	0.692958622
RELN	0.345867583	0.749314762	0.392305766	1.431211728	0.382070061
REM1	0.645277398	195.6541181	0.317400747	120606.3132	0.107434842
REN	0.341851598	0.46376137	0.168639578	1.275350726	0.136558881
RENBP	0.068482431	0.076059074	0.004398264	1.315287622	0.076475243
REP15	0.073928114	13614.72562	0.402202047	460864769.8	0.0736462
REPIN1	0.78754677	0.985583144	0.625011039	1.554171165	0.95017223
REPS1	0.838577851	1.131510126	0.812104962	1.576539024	0.465327238
REPS2	0.830605234	0.700137791	0.186399669	2.629795047	0.597532112
RER1	0.274511405	1.613350192	0.751066451	3.465603926	0.220145394
RERE	0.708333412	0.853944738	0.422370275	1.726498425	0.660242342
RERG	0.590641286	1.263592596	0.013105164	121.8348909	0.920052346
RESP18	0.20402196	0.531276434	0.187867691	1.502411869	0.233077668
REST	0.748311462	637.9152272	0.060365044	6741249.723	0.171901432
RET	0.837733351	0.012791616	1.14E-05	14.29861996	0.223542652
RETN	0.924251159	0.423737179	0.141964776	1.264772872	0.123813749
RETNLB	0.117705273	1.399485238	0.694969232	2.818195166	0.346660912
REV3L	0.464892228	0.722598813	0.441275326	1.183272696	0.196638719
REXO1	0.197051077	1.099571127	0.888314559	1.361068162	0.383209981

REXO2	0.100184075	0.00397117	2.25E-08	699.8860577	0.369691083
REXO4	0.113110659	1.138233805	0.916649666	1.413382061	0.241151168
RFC1	0.548332513	0.009878643	2.76E-07	353.2271496	0.38804399
RFC2	0.492527464	0.128295094	0.000693569	23.73176931	0.440726119
RFC3	0.416267439	1.127389651	0.398646983	3.18830313	0.821154024
RFC4	0.616449922	0.51613448	3.03E-06	87938.95601	0.914301806
RFC5	0.447179627	0.877326709	0.600740875	1.28125484	0.498202705
RFFL	0.861600434	0.756716837	0.245822197	2.329408733	0.627015949
RFK	0.845463527	0.030694524	1.69E-06	556.8228399	0.486240405
RFNG	0.799408459	0.494058124	0.005406591	45.1473788	0.759541538
RFPL1	0.751610062	1.355558579	0.779399528	2.357634301	0.281329069
RFPL2	0.517288252	0.528117756	0.13397656	2.081769868	0.361629528
RFPL3	0.620363456	1.057959234	0.762995485	1.466951984	0.735468552
RFT1	0.519068423	11.82677774	0.00295009	47413.01716	0.559480011
RFWD2	0.352978422	5.701731411	0.093280355	348.5164799	0.406796403
RFWD3	0.378307988	1.046740153	0.782336364	1.400503668	0.758453507
RFX1	0.44325106	0.484927852	0.103226416	2.278050819	0.359188042
RFX2	0.197878345	0.522756255	0.221777206	1.232201033	0.138159798
RFX3	0.628614869	1.018695032	0.020723041	50.07660721	0.992563496
RFX4	0.250187588	5.771801426	0.660557969	50.4326543	0.112960739
RFX5	0.087952974	1.799716137	0.985574808	3.286384907	0.055790366
RFXANK	0.143055832	0.956127435	0.443164647	2.062844314	0.908957713
RFXAP	0.765355117	0.987801117	0.696439584	1.401056271	0.945124266
RGAG1	0.504719485	0.04686264	0.001410374	1.557110157	0.086855932
RGAG4	0.303267771	0.919321468	0.3361117	2.514497301	0.869844212
RGL1	0.441593263	0.035180779	1.08E-05	114.1256229	0.417086866
RGL2	0.833795749	1.335771011	0.52146883	3.421650711	0.546341761
RGMA	0.773644629	1.123102057	0.429417584	2.937369771	0.812911303
RGMB	0.988931657	0.9011101	0.370810145	2.189798269	0.818213214
RGN	0.903157858	0.418775963	0.034525691	5.079501666	0.49423386
RGPD2	0.443801999	0.660909439	0.00017816	2451.733068	0.921327018
RGPD4	0.835905933	0.809226574	0.019042826	34.38815405	0.911892172
RGPD5	0.067630087	386266.9504	0.25522841	5.84583E+11	0.07641573

RGR	0.175266405	0.598545328	0.288848256	1.240293139	0.167380364
RGS1	0.62353249	2.731541363	0.673950187	11.07102329	0.15933125
RGS10	0.937996255	1.071998128	0.777012349	1.478972616	0.671991773
RGS11	0.103028358	0.732381883	0.493677223	1.086505913	0.121698835
RGS12	0.741969463	0.999239113	0.877650267	1.137672763	0.990825749
RGS13	0.368850672	1.362753236	0.464614455	3.99706975	0.572926142
RGS14	0.747858956	0.532072964	1.23E-05	22932.68756	0.907740359
RGS16	0.34628522	1.574671933	0.074575202	33.24954722	0.770457753
RGS17	0.420136409	36.7491158	0.189678075	7119.945271	0.179827857
RGS18	0.473246072	1.04714274	0.179113593	6.121857658	0.959221538
RGS19	0.8252197	1.973213827	0.378532762	10.28595989	0.419783261
RGS2	0.91265906	1.240582253	0.436668384	3.524515126	0.685727417
RGS20	0.084453202	1.684005487	0.798973957	3.549395389	0.170682845
RGS22	0.975664582	5.172035939	0.043243305	618.591844	0.500815607
RGS3	0.446388574	0.923210157	0.435526127	1.956982468	0.834889025
RGS4	0.567660647	1.173934362	0.368536703	3.739442708	0.786173713
RGS5	0.359824524	0.02529129	2.12E-08	30123.08232	0.606436151
RGS6	0.375107046	1.236431874	0.919815177	1.662033653	0.159673349
RGS7	0.597374954	1.780291405	0.008883916	356.7613226	0.831106154
RGS8	0.372210496	2.927076986	0.052284734	163.8677102	0.600990518
RGS9	0.441237637	0.023373815	1.18E-05	46.41982857	0.332319142
RGS9BP	0.358645688	1.183626212	0.665166766	2.106195137	0.566414286
RGSL1	0.820565429	0.591287435	0.212530917	1.645035159	0.314173548
RHAG	0.268468021	0.183984217	0.025637226	1.320353138	0.092260283
RHBDD1	0.747877135	11.1292765	0.00021471	576875.1791	0.663534714
RHBDD2	0.244217166	0.611747013	0.083537785	4.479822032	0.628548352
RHBDD3	0.151880707	0.590990096	0.209228255	1.669321832	0.320827301
RHBDF1	0.172398814	0.722305309	0.162303981	3.214492689	0.669335629
RHBDF2	0.212049217	0.673147769	0.277960599	1.630187592	0.380461364
RHBDL1	0.43845092	1.338465385	0.736440198	2.432634165	0.338892254
RHBDL2	0.47041328	0.578059468	0.178885161	1.867973545	0.359752838
RHBDL3	0.328982587	0.006894379	2.12E-07	223.9116476	0.347719753
RHBG	0.880314673	0.692576036	0.146469611	3.274819687	0.643064067

RHCE	0.467990559	1.039378153	0.792721145	1.362783056	0.779916133
RHCG	0.098062868	0.209905514	0.034801604	1.266042951	0.08862905
RHD	0.331381225	0.243244998	0.012630094	4.684694374	0.348908087
RHEB	0.585303169	0.924236783	0.232337224	3.676611159	0.910954069
RHEBL1	0.924805647	1.256456593	0.712034527	2.217144126	0.430772321
RHO	0.730355104	1.355026688	0.094475586	19.43462226	0.82307465
RHOA	0.065156581	1.372496363	0.898099723	2.097480066	0.143390217
RHOB	0.399084294	1.169876294	0.943267651	1.450924922	0.153209717
RHOBTB1	0.477437564	0.448111561	0.109641508	1.8314594	0.263767574
RHOBTB2	0.482767797	1.527330118	0.277342233	8.411042424	0.626568875
RHOBTB3	0.162909363	0.803155533	0.596207082	1.081937517	0.149322419
RHOC	0.943169457	1.563840695	0.823293798	2.970504243	0.171948793
RHOF	0.613364544	1.086307069	0.394084376	2.994442607	0.872867354
RHOG	0.818190688	1.161617796	0.299394338	4.506951981	0.828542193
RHOH	0.742791708	0.547499726	0.130059891	2.304753197	0.411412116
RHOJ	0.660170453	1.058959067	0.780191713	1.43733173	0.713228233
RHOQ	0.069110131	0.450991915	0.005321368	38.22207458	0.725184626
RHOT1	0.751658814	22.09354235	0.068492065	7126.732321	0.293598025
RHOT2	0.574931386	1.396218424	0.266825268	7.306001807	0.69263092
RHOU	0.912166122	1.113619088	0.787019394	1.575752113	0.543427566
RHOV	0.939085229	2.951825955	0.577498375	15.08796708	0.193475735
RHPN1	0.859388003	0.603172953	0.224867494	1.617919983	0.315269734
RHPN2	0.133774822	1.744183542	0.770417564	3.948736857	0.18209239
RIBC1	0.177134288	0.716897901	0.234383599	2.192741309	0.559568314
RIBC2	0.140305848	1.453101872	0.102983724	20.50328894	0.781996987
RIC3	0.6420972	0.915825287	0.733028598	1.144206322	0.438891143
RIC8A	0.450767367	0.009822153	6.99E-07	138.0465682	0.34275276
RIC8B	0.656184419	1.081850944	0.86034381	1.360388081	0.500905529
RICTOR	0.962978726	0.836164915	0.165645676	4.2208875	0.828507922
RIF1	0.067618967	0.001028073	9.90E-08	10.68026148	0.144827914
RILP	0.863965907	1.337171141	0.150143953	11.90874908	0.794534521
RIMBP2	0.750570899	1.03139504	0.797364088	1.334115425	0.813882187
RIMS1	0.881468033	1.940404302	0.437749862	8.601187978	0.382900142

RIMS2	0.765405286	0.949493386	0.79634988	1.132087432	0.563593827
RIMS3	0.913585714	1.313435075	0.58455142	2.951171848	0.509199213
RIMS4	0.173991697	0.478891322	0.128260158	1.788060318	0.273344359
RIN1	0.483446926	1.112649715	0.560763294	2.207686206	0.760111878
RIN2	0.691783604	0.964832143	0.749758885	1.241600578	0.780839929
RIN3	0.761206349	0.771756284	0.183763078	3.241172094	0.723442004
RING1	0.096353558	0.054282796	0.000284995	10.33920251	0.276678946
RIOK1	0.360696836	0.377538894	0.000140345	1015.612158	0.808975272
RIOK2	0.500642259	1.148620065	0.286294249	4.608293935	0.845019758
RIOK3	0.362130416	0.500978645	0.18971205	1.322950239	0.162988571
RIPK1	0.771179159	0.591189802	0.20207221	1.729606367	0.337232867
RIPK2	0.65466923	0.603431212	0.042800371	8.507618505	0.708295385
RIPK3	0.129729432	2.395872365	0.801539637	7.161472901	0.117821195
RIPK4	0.647097065	8.559159525	0.000428947	170788.4849	0.670833328
RIT1	0.744187335	6.26114113	0.001397505	28051.3432	0.668919192
RIT2	0.124440182	1.782799973	0.852857218	3.726738402	0.124320626
RLBP1	0.913332338	0.559636117	0.220266683	1.421879056	0.222419724
RLF	0.162313114	13.30516863	0.071418244	2478.743553	0.331841331
RLN1	0.176271545	0.055578127	0.001341756	2.302152854	0.128238746
RLN2	0.236948697	0.862020491	0.314733571	2.360978921	0.772714004
RLN3	0.955838183	0.103431043	0.000702304	15.2326851	0.373065558
RLTPR	0.095624894	319.0487383	0.090656908	1122827.806	0.166429755
RN7SK	0.376944084	1.374006313	0.991852191	1.903401904	0.056034901
RNASE1	0.151372026	1.721819089	0.644025663	4.603327392	0.278813941
RNASE10	0.689228576	5.340440437	0.07780856	366.5445537	0.43747181
RNASE11	0.451970476	0.238807839	0.001354679	42.09792718	0.587341567
RNASE12	0.65192912	0.987630376	0.873600847	1.116543973	0.842384488
RNASE13	0.938389066	1.620439599	0.453201258	5.793947938	0.457766295
RNASE2	0.55658711	0.563070412	0.253149762	1.252413929	0.159087258
RNASE3	0.35188935	0.025213486	3.76E-06	169.0764796	0.412953554
RNASE4	0.616114174	1.091172996	0.535657256	2.222799173	0.810058009
RNASE6	0.754473041	0.327833293	0.002770954	38.78615982	0.647001759
RNASE7	0.676753054	1.441638238	0.463669436	4.482332987	0.527387953

RNASE8	0.087894201	1.628442725	0.854687675	3.102683926	0.138189956
RNASE9	0.52921206	1678.907053	0.528255326	5335921.384	0.071097196
RNASEH1	0.873837715	3.625515847	0.078184585	168.1196506	0.510556236
RNASEH2A	0.315221395	1.072967359	0.932968794	1.233973698	0.323493957
RNASEL	0.419610053	1.053207313	8.19E-05	13548.5924	0.991432511
RNASET2	0.170130251	0.672373484	0.347174621	1.302186491	0.239189431
RND1	0.513808991	1.036213281	0.412368802	2.603829288	0.939682787
RND2	0.997441983	1.109041916	0.306467338	4.013393337	0.874677854
RND3	0.597719592	0.348686873	0.000651571	186.5991064	0.742393437
RNF10	0.072591573	2.254371204	0.973704517	5.219437148	0.05772873
RNF103	0.053830379	39.07214951	0.002568411	594388.1135	0.455655963
RNF11	0.434666988	1.468675016	0.534730523	4.033819298	0.455900815
RNF111	0.153553041	0.351492849	0.097103641	1.272323275	0.11115677
RNF113A	0.194103054	0.677493859	0.129355514	3.548344503	0.6448935
RNF113B	0.145103113	0.015634968	3.44E-05	7.101449019	0.182854269
RNF121	0.389372643	0.8059603	0.395822232	1.641070038	0.552107882
RNF122	0.225415295	0.666784587	0.218458559	2.035176314	0.476547786
RNF123	0.052821846	1.247921539	0.94533024	1.64736946	0.118013642
RNF125	0.538009634	1.502887456	0.086793579	26.02347697	0.779473412
RNF126	0.495431691	1.01149217	0.332563871	3.076450873	0.983936662
RNF126P1	0.502638891	0.326977781	0.038419728	2.782801347	0.306219579
RNF128	0.607458437	0.014959716	1.66E-05	13.44415049	0.225861308
RNF13	0.237259455	0.621007977	0.365148774	1.056147344	0.078689075
RNF130	0.549589548	1.290658746	0.546720154	3.046897005	0.560434091
RNF133	0.520336315	0.995832861	0.740302425	1.339564822	0.977979735
RNF135	0.210267976	0.491911809	0.212273523	1.139931277	0.098019517
RNF138	0.245113753	2.309968371	0.694381453	7.684470626	0.172184471
RNF138P1	0.774937423	1.055195713	0.196476637	5.667024895	0.950049587
RNF139	0.01592878	1.85578807	1.212763367	2.839753783	0.004389672
RNF14	0.255083488	0.626343281	0.000112658	3482.287614	0.915314401
RNF141	0.718595161	0.953914916	0.368644303	2.468378489	0.922517019
RNF146	0.065609963	0.004472394	1.23E-05	1.630952308	0.072266218
RNF148	0.513397379	0.107511774	3.37E-06	3428.319228	0.673384618

RNF149	0.19173708	4.161256375	0.249806397	69.31789914	0.320475408
RNF150	0.76709383	0.740433117	0.425654619	1.287995424	0.287352903
RNF151	0.250654111	1.092467306	0.742686018	1.606984358	0.653322598
RNF152	0.302572363	0.617946104	0.162604402	2.348382836	0.479783261
RNF157	0.722442405	1.055479636	0.910375901	1.223711284	0.474252506
RNF165	0.551277473	0.299580158	0.006059098	14.81214937	0.544754696
RNF166	0.972330856	0.948630132	0.734370804	1.225401558	0.686398649
RNF167	0.442379257	0.820028062	0.090817115	7.404397559	0.859721589
RNF168	0.216307885	4.07E-05	5.01E-11	33.0599279	0.145369981
RNF169	0.125838338	0.276859874	0.017068844	4.490719469	0.366319878
RNF17	0.267329425	0.88194125	0.432389908	1.7988865	0.729762434
RNF170	0.124737069	0.591001228	0.078115113	4.471381249	0.610479322
RNF175	0.121979548	1.952340215	0.769048843	4.956294195	0.159278009
RNF180	0.745258586	0.70681187	0.393283439	1.270287457	0.246008552
RNF182	0.533647515	0.165527258	0.000602735	45.45827738	0.530149886
RNF183	0.202902689	1.146397341	0.912335492	1.440508317	0.240973948
RNF185	0.386670684	0.023984657	1.05E-06	549.3358577	0.466436481
RNF186	0.692887244	1.027812806	0.105538254	10.00963274	0.981153628
RNF187	0.651199714	1.10226918	0.522441791	2.325612857	0.798250044
RNF2	0.82110122	1.081272986	0.534294682	2.188214312	0.82801351
RNF20	0.650915649	3.743352365	0.304007331	46.09325339	0.302802374
RNF24	0.262373549	0.003614388	1.07E-08	1222.472952	0.386701167
RNF25	0.937133488	1.171033126	0.621575477	2.206198012	0.625147235
RNF26	0.427747021	0.963332538	0.6029227	1.539185006	0.87584042
RNF31	0.077708515	1.83857947	0.842553607	4.012058631	0.126102832
RNF32	0.371821591	3.010028764	0.545206463	16.61805898	0.206190833
RNF34	0.078630523	1.413469091	0.794111699	2.51588646	0.239467531
RNF38	0.168503619	0.563703136	0.016862928	18.84377494	0.748861614
RNF39	0.077351675	41.8790325	0.621125428	2823.670201	0.082155864
RNF4	0.463612448	1.259785752	0.405506763	3.913769842	0.689667562
RNF40	0.433673487	0.749231095	0.457993767	1.225665661	0.250280953
RNF41	0.621156384	0.932395734	0.015781299	55.08810287	0.973168488
RNF43	0.625889563	0.82276549	0.572465497	1.182504543	0.291817059

RNF44	0.809959605	1.003973593	0.73235136	1.376337958	0.980342828
RNF5	0.63714075	0.435492466	0.000504451	375.9606389	0.809563048
RNF5P1	0.180122822	3.041038795	0.727141815	12.71817513	0.127634297
RNF6	0.960419673	1.010682155	0.647441738	1.577714808	0.962702816
RNF7	0.644508559	0.738383655	0.391181583	1.393752791	0.349428938
RNF8	0.423197987	0.090360354	0.002433759	3.354890027	0.192373419
RNGTT	0.721965068	0.944594432	0.69730968	1.279573002	0.71282506
RNH1	0.314845918	1.277104642	0.766144424	2.128836569	0.348144782
RNMT	0.759330265	0.967390794	0.769732239	1.215805836	0.776185158
RNMTL1	0.21090917	1.171422306	0.82207844	1.669220543	0.381217533
RNPC3	0.246022779	0.66777522	0.361714403	1.232806161	0.196741666
RNPEP	0.225850775	0.690616702	0.435092646	1.096206597	0.116344792
RNPEPL1	0.776352872	1.545703288	0.545023444	4.383662175	0.412899672
RNPS1	0.054732247	0.44325051	0.150538084	1.305124982	0.139768518
RNU12	0.995464004	0.820947855	0.407679129	1.653151542	0.580650903
ROBO1	0.476515127	1.358556485	0.699515171	2.638507068	0.365587442
ROBO2	0.555770936	1.949846399	0.425386191	8.937527975	0.39000274
ROBO3	0.088156484	0.000640933	4.53E-08	9.063494687	0.131578961
ROBO4	0.970684082	0.752647303	0.26057958	2.173915406	0.59953135
ROCK1	0.672890079	1.017954228	0.807352291	1.283492749	0.88039378
ROCK2	0.958525903	1.472712489	0.197599023	10.97617814	0.705632239
ROM1	0.689647333	0.067588952	9.46E-05	48.29446212	0.421646305
ROPN1	0.82694692	0.818244306	0.380490886	1.759631488	0.607627774
ROPN1B	0.160283377	0.008327294	2.21E-05	3.14290776	0.113720818
ROPN1L	0.85513498	1.033236705	0.258186326	4.134913355	0.963142332
ROR1	0.888743227	0.645101311	0.270499114	1.538473436	0.322906346
ROR2	0.285141253	0.838481956	0.623242785	1.128054761	0.244474626
RORA	0.911911905	1.399214733	0.623452978	3.14025586	0.415402763
RORB	0.775336307	622.8314327	0.056127365	6911405.76	0.175762376
RORC	0.283691065	0.430366141	0.115409731	1.604847468	0.209280942
ROS1	0.892296154	1.42089323	0.580609822	3.477270786	0.441706579
RP1	0.056839549	1.555910651	0.694423146	3.486142371	0.282827969
RP1L1	0.89613801	0.769843849	0.347698658	1.704520675	0.518940392

RP2	0.691686547	1.638769812	0.505997728	5.307467497	0.410047457
RP9	0.765956561	0.385168666	3.88E-05	3819.780227	0.838970246
RPA1	0.975010425	0.791304526	0.411876614	1.520268041	0.48230083
RPA2	0.56980359	0.871563345	0.552488605	1.374911006	0.554492343
RPA3	0.801726475	0.952579345	0.392832892	2.3099069	0.914395554
RPA4	0.342757657	2.401767864	0.526367117	10.95906009	0.257912045
RPAP1	0.858785449	0.983421516	0.420289227	2.301077008	0.969254382
RPE	0.195428758	0.00063402	2.25E-08	17.88717574	0.159028296
RPE65	0.124265404	1.907287453	0.68560455	5.305894523	0.216125573
RPGR	0.924395414	0.420546049	0.014385629	12.29414256	0.614977758
RPGRIP1	0.921600699	1.406664099	0.595595103	3.322229946	0.436462105
RPH3A	0.873182336	0.536683256	0.191609127	1.503210841	0.2362914
RPH3AL	0.750988525	0.462660215	0.019533295	10.95844142	0.633132117
RPIA	0.609942798	2.178585933	0.910542182	5.212539038	0.080218731
RPL10	0.531301112	0.649531801	0.248521653	1.697604842	0.378692381
RPL10A	0.968663942	8.32360635	0.000449195	154236.8698	0.672557585
RPL10L	0.699041825	1.22525375	0.842299141	1.782320175	0.288043052
RPL11	0.856163351	0.944578224	0.309502597	2.882780404	0.920220789
RPL12	0.058581608	1.434340085	0.624959624	3.291943032	0.394782294
RPL13	0.448946018	0.898454778	0.489106375	1.650399646	0.7299985
RPL13A	0.359354609	1.62425537	4.31E-05	61159.75118	0.928104549
RPL14	0.215028016	0.910660364	0.330612549	2.508381193	0.856344015
RPL15	0.079788117	2.046406152	0.184143121	22.74197437	0.560015471
RPL17	0.206786776	0.035359546	6.90E-06	181.1055995	0.443122308
RPL18	0.182261499	2.098971182	0.624519955	7.054506408	0.230605235
RPL18A	0.830610788	0.333008669	0.092254672	1.20205049	0.093158139
RPL19	0.84710216	2.057818261	0.620913492	6.819977424	0.23782991
RPL22	0.997441734	1.552554939	0.197983239	12.17490354	0.675474502
RPL23	0.272955199	0.97436447	0.444621758	2.135266894	0.948271966
RPL23A	0.094715691	0.063569129	0.00261159	1.547346456	0.090659076
RPL24	0.430774199	1.022917787	0.366729111	2.853225361	0.965466812
RPL26	0.626038811	0.61090116	0.224681507	1.661018889	0.334210851
RPL26L1	0.918104111	1.042719836	0.640781018	1.696780376	0.866274618

RPL27	0.800797158	0.97919162	0.884459024	1.084070831	0.685443173
RPL27A	0.426860598	1.247888912	0.676258271	2.302710079	0.478645366
RPL28	0.591263245	4.374040205	0.016557021	1155.535663	0.604008172
RPL29	0.089379852	0.757626407	0.447109467	1.283796954	0.302290817
RPL3	0.756870471	0.258719813	0.004470834	14.97169097	0.513771429
RPL30	0.455769217	0.692916321	0.185317018	2.590873925	0.585630179
RPL31	0.995276892	1.094345698	0.271854525	4.405269713	0.899032531
RPL32	0.115906358	0.209959203	0.029498435	1.494413744	0.119052087
RPL34	0.84627507	0.111288988	0.002924777	4.234591849	0.236971131
RPL35	0.540993336	1.266995854	0.789385937	2.033578786	0.326943002
RPL35A	0.821015994	0.254657212	4.67E-05	1389.369398	0.755365126
RPL36	0.222023932	2.024163517	0.995542986	4.115581145	0.051459695
RPL36A	0.183762233	0.562421985	0.171885661	1.840284338	0.341335669
RPL36AL	0.913971721	1.070032429	0.155654355	7.355845575	0.945134434
RPL37	0.180942306	0.350242825	0.065968803	1.85951587	0.218061364
RPL37A	0.000638223	1.792496523	1.188893292	2.702550183	0.005337917
RPL38	0.904832693	1.039959195	0.537907284	2.010597661	0.90726639
RPL39	0.759567185	1.196981605	0.378925828	3.781122469	0.759312837
RPL39L	0.674908692	0.129032965	0.003305331	5.037167629	0.27342917
RPL3L	0.429632704	1.16326599	0.745597975	1.814902679	0.50516481
RPL4	0.779329517	0.041838096	0.000166052	10.54141393	0.260557875
RPL41	0.656885361	1.1058545	0.287721793	4.250335586	0.883547967
RPL5	0.579045118	1.086560748	0.529919138	2.227913985	0.82073324
RPL6	0.355832549	1.188580121	0.867525016	1.628451835	0.282208949
RPL7	0.77119275	1.401419215	0.334046623	5.879346416	0.64459664
RPL7A	0.467907648	0.804874246	0.253051422	2.560043122	0.713106831
RPL7L1	0.927828952	0.952716584	0.757750517	1.197846613	0.678408535
RPL8	0.131310977	0.005722685	1.85E-05	1.768187397	0.07754353
RPL9	0.660621991	1.394379636	0.769184983	2.527733397	0.273367242
RPLP0	0.364257676	1.278880838	0.460037467	3.5552239	0.637252009
RPLP1	0.686033431	0.048973644	0.000387193	6.194369762	0.221898372
RPLP2	0.80611156	1.090528537	0.684216418	1.738123287	0.715571118
RPN1	0.965514214	1.046924966	0.793817302	1.380735695	0.745367467

RPN2	0.677731175	0.851595157	0.471807664	1.537097353	0.593917788
RPP14	0.277412161	1.642450817	0.473853686	5.692990826	0.434001159
RPP21	0.940974354	0.924275155	0.767694683	1.112792078	0.405699893
RPP25	0.92620526	1.273996122	0.220083858	7.374762217	0.786929113
RPP30	0.206165833	4.765215126	0.37211903	61.02153713	0.230091457
RPP38	0.496663264	0.204022824	0.008942415	4.654817918	0.319173693
RPP40	0.134711708	0.014068202	3.12E-05	6.335074084	0.171385255
RPPH1	0.333271193	0.461195271	0.146789954	1.449016581	0.185171937
RPRM	0.847262865	0.014560861	1.91E-07	1110.982989	0.460914411
RPRML	0.247541466	0.851578548	0.455429703	1.592311655	0.614860914
RPS10	0.053000946	4109.280531	0.264886357	63748796.46	0.091002515
RPS11	0.873801582	0.108323611	7.87E-05	149.0210256	0.546640605
RPS12	0.338177662	2.4572934	0.01823579	331.1230786	0.71932126
RPS13	0.257602171	0.760241217	0.491976242	1.17478581	0.217013774
RPS14	0.924668417	0.949919871	0.68293293	1.321283132	0.760241209
RPS15	0.651700945	1.000561371	0.387295515	2.584907436	0.99907532
RPS15A	0.533816028	0.868694396	0.273960715	2.754518847	0.811048766
RPS16	0.12193455	11.33932079	0.052081512	2468.826102	0.376639423
RPS17	0.399099506	0.496601461	0.066779655	3.692936276	0.494119728
RPS18	0.621436203	1.368594679	0.054358717	34.45724043	0.848804251
RPS19	0.928275534	46.61077371	0.049652501	43755.3836	0.271277397
RPS19BP1	0.339559979	0.994568353	0.481615887	2.053848797	0.988255051
RPS2	0.807580713	1.006412429	0.815764126	1.241616228	0.952433662
RPS20	0.784344945	0.937613317	0.308679139	2.848001759	0.909524433
RPS21	0.792354727	1.206720705	0.146520416	9.938375123	0.861339376
RPS23	0.179061053	16.27446885	0.003451409	76739.20151	0.518028697
RPS24	0.870052807	1.093688478	0.278686022	4.292122286	0.897847125
RPS25	0.239251704	0.226607131	0.039788452	1.290595365	0.09441555
RPS26	0.07714618	1.345418724	0.994638614	1.819908777	0.054218771
RPS27A	0.195252619	0.002825317	1.26E-07	63.50295665	0.25096532
RPS27L	0.453116698	0.91030833	0.276063478	3.00170548	0.87732157
RPS28	0.010971169	1.664992583	1.119504624	2.476274095	0.011823506
RPS29	0.168802771	0.260880137	0.058926057	1.154980482	0.07670186

RPS3	0.333407256	0.408435756	0.100133905	1.665966861	0.211895371
RPS3A	0.811879018	1.102881901	0.457750827	2.657228374	0.827222742
RPS4X	0.503806654	1.199381474	0.593995323	2.421763041	0.612085476
RPS4Y1	0.602876827	0.682324376	0.001898345	245.2486123	0.898689951
RPS5	0.974271503	0.088951133	0.003360945	2.354190545	0.14770161
RPS6	0.694768486	0.754579385	0.122833734	4.635453392	0.761104518
RPS6KA1	0.922434806	0.866757145	0.158653339	4.73527978	0.868901455
RPS6KA2	0.099683778	1.410889046	0.542105047	3.671996622	0.480605038
RPS6KA3	0.453536366	1.063002107	0.848915117	1.331079465	0.594402918
RPS6KA4	0.255766956	0.703840227	0.202773363	2.443077621	0.580175787
RPS6KA5	0.142641602	0.618585018	0.198959522	1.923242577	0.406581371
RPS6KA6	0.921513965	0.731497718	0.237412416	2.253837102	0.586047717
RPS6KB1	0.207238625	4.339171729	0.756224849	24.89790082	0.099660083
RPS6KB2	0.721511928	17.64573871	0.025756555	12089.04283	0.38889113
RPS6KC1	0.808500436	0.801537214	0.297276005	2.161163013	0.662006448
RPS6KL1	0.327893684	0.129036034	8.90E-05	187.0802647	0.581397609
RPS7	0.154506195	0.01203888	3.80E-07	381.1880005	0.403214943
RPS8	0.777536903	4.226634053	0.062964928	283.7204125	0.501844076
RPS9	0.161776008	2.394421658	0.128529323	44.60659209	0.558466457
RPSA	0.819592028	1.937347639	0.24049214	15.60681307	0.534436816
RPTN	0.248179115	9.446190828	0.020434261	4366.711449	0.473203624
RPUSD1	0.75397361	0.535615606	0.000965838	297.0311855	0.846429332
RPUSD2	0.611671826	1.057945736	0.363051137	3.082896778	0.917784721
RPUSD3	0.516301805	1.539271884	0.726362132	3.261951343	0.260330705
RPUSD4	0.321671672	1.480014607	0.864496131	2.533780268	0.152955596
RQCD1	0.558211056	0.858924309	0.301857703	2.444035592	0.775623007
RRAD	0.631552097	0.890193357	0.002179549	363.5817373	0.96975291
RRAGA	0.230636516	1.805141057	0.480193592	6.78587613	0.382004854
RRAGB	0.659558028	3.795483555	0.537284868	26.81202518	0.181166471
RRAGC	0.245494688	133.3824069	0.17801116	99942.42218	0.147362176
RRAGD	0.306626213	0.661172569	0.160846568	2.717802267	0.566192548
RRAS	0.08774411	0.423170855	0.127213318	1.407663719	0.160803561
RRAS2	0.374660194	0.799304806	0.474593049	1.34618106	0.399642393

RRBP1	0.86721616	0.969064528	0.006411602	146.4666909	0.990207583
RREB1	0.439350919	1.191799707	0.432168079	3.286653066	0.734594301
RRH	0.440589869	0.895774204	0.317811841	2.524800274	0.835084626
RRM1	0.785048801	0.203084233	0.031464516	1.310784673	0.093831959
RRM2	0.290098012	1.175186196	0.854734128	1.615780335	0.320361649
RRM2B	0.633465262	0.746953013	0.239289037	2.331652174	0.615430463
RRN3	0.640909819	0.187357198	3.87E-06	9059.300879	0.760888036
RRS1	0.411427445	0.901755384	0.774575783	1.049816933	0.182465476
RS1	0.555697132	1.04658036	0.829219671	1.320917108	0.701491681
RSAD1	0.782487675	2.191435123	0.025235844	190.3002676	0.730497462
RSAD2	0.530457477	1.237949631	0.512327206	2.991290077	0.635353335
RSBN1	0.400216604	5.684006506	0.002015535	16029.45318	0.668148409
RSBN1L	0.888224449	1.443683176	0.15834644	13.16241219	0.744705265
RSL1D1	0.723434165	1.295201293	2.42E-06	693683.1711	0.969342295
RSPO1	0.467978154	0.989388311	0.304921934	3.210294575	0.985826522
RSPO2	0.271409171	0.648128755	0.083659875	5.021175099	0.67802475
RSPO3	0.841344972	1.308434447	0.296418161	5.775626897	0.722695416
RSPO4	0.875293387	0.972336911	0.537033547	1.760484191	0.926205982
RSPRY1	0.2434742	1.268442536	0.883708891	1.820674753	0.197213039
RSRC1	0.454939354	1.303678451	0.7284686	2.333082721	0.371824041
RSU1	0.848896503	0.049188699	4.61E-06	525.1017296	0.524477405
RTBDN	0.97869016	0.117180894	0.000916706	14.97902378	0.386316191
RTEL1	0.547418585	0.069404954	0.001615292	2.982153555	0.164385938
RTF1	0.586884292	0.557972883	0.007301252	42.64114437	0.792000777
RTKN	0.971183641	1.428297389	0.204938649	9.954361652	0.718945719
RTN1	0.32008244	0.986896228	0.620144766	1.570543231	0.955625738
RTN2	0.396376572	1.61413108	0.042791451	60.88644072	0.796018031
RTN3	0.596703679	0.815165697	0.263861166	2.518351314	0.722511829
RTN4	0.475334091	0.729399138	0.291521633	1.824986697	0.50009949
RTN4IP1	0.505390918	0.529846969	0.128603583	2.182970375	0.37925988
RTN4R	0.083472632	1.296744868	0.963529766	1.74519492	0.086382181
RTN4RL1	0.157432469	0.282145836	1.87E-05	4253.927066	0.79658308
RTN4RL2	0.359594864	0.424794134	0.122868645	1.468642033	0.176147582

RTP1	0.162063038	1.157637503	0.956499321	1.40107218	0.132778588
RTP2	0.407660491	25.48597015	0.003784817	171615.859	0.471532209
RTP3	0.440419797	0.668862062	0.211130552	2.118956515	0.494231455
RTP4	0.930619358	0.490088627	3.80E-05	6327.28415	0.882605576
RTTN	0.658569026	0.749779249	0.336983771	1.668237374	0.480340428
RUFY1	0.206542599	27683.98282	0.718514212	1066649612	0.057616485
RUFY2	0.745056048	0.604696502	0.175910878	2.078654049	0.424594156
RUFY3	0.591834226	0.003980955	2.57E-06	6.154719885	0.140226243
RUNDC1	0.629050785	0.959041126	0.364519095	2.523214541	0.932472517
RUNX1	0.721447074	1.250020356	0.298257057	5.238940223	0.760189366
RUNX1T1	0.752921632	0.001177493	5.28E-08	26.24448564	0.186731314
RUNX2	0.075119916	2.646996964	0.534159283	13.11704795	0.233236996
RUNX3	0.233349608	0.65912094	0.202817994	2.142021052	0.48818225
RUSC1	0.956776297	0.898241632	0.457686625	1.762861283	0.755076077
RUSC2	0.965368677	6.686042547	0.13229664	337.9009838	0.342451633
RUVBL1	0.795077723	1.233561994	0.795532536	1.912775562	0.348297307
RUVBL2	0.167556213	0.965121274	0.352497831	2.642453347	0.944923041
RWDD1	0.499996341	1.045815198	0.501845344	2.179415314	0.904819032
RWDD3	0.185429967	0.582097922	0.098026519	3.456595144	0.551604975
RXRA	0.448359474	1.10100807	0.36039409	3.363592261	0.865892798
RXRB	0.853489968	0.92448477	0.194955859	4.383926149	0.921238279
RXRG	0.708632427	1.325331346	0.153505608	11.4425994	0.79788131
RYBP	0.740269124	0.952681531	0.848572517	1.069563392	0.411655358
RYK	0.954653652	0.793235519	0.423213979	1.486771752	0.46989769
RYR1	0.643824875	1.071592467	0.93258603	1.231318483	0.329356357
RYR2	0.060338731	6.613741257	0.87343057	50.08019515	0.067407147
RYR3	0.113259648	1.123884182	0.914378632	1.381392358	0.267184556
S100A1	0.085767682	0.896422954	0.021853147	36.77155191	0.953986197
S100A10	0.610148934	0.044080198	9.30E-05	20.89585815	0.320683022
S100A11	0.184532466	1.918656461	0.777169976	4.736727783	0.157589498
S100A12	0.542806867	1.05908931	0.878484958	1.276823418	0.547292043
S100A13	0.679058712	1.156086157	0.59778338	2.235818604	0.666468009
S100A14	0.198216387	1.104179106	0.769958763	1.583476358	0.590047372

S100A16	0.562877893	0.431609901	0.09294551	2.004261493	0.283497422
S100A2	0.209342648	1.507998815	0.941968303	2.414158117	0.087088778
S100A3	0.234270385	1104.546598	0.032742211	37261478.65	0.187760541
S100A4	0.407338936	3.483775642	0.020794809	583.6404973	0.632880218
S100A5	0.576910214	2.25592419	0.593108489	8.580544789	0.232642513
S100A6	0.733683971	104.1398083	0.002610791	4153951.487	0.390061096
S100A7	0.260000944	0.593840412	0.259690826	1.357947218	0.216860643
S100A8	0.814474098	0.937397615	0.590496204	1.488094727	0.783952108
S100A9	0.104114443	0.746961973	0.298389386	1.869879478	0.533193058
S100B	0.941484484	1.085617521	0.756033639	1.558879581	0.656320067
S100G	0.225160937	0.596862176	0.311658428	1.143060561	0.119553906
S100P	0.214669259	0.535420295	0.176884871	1.620686332	0.268944537
S100PBP	0.515741703	1.531676022	0.000901073	2603.597715	0.910549762
S100Z	0.732983758	0.905112132	0.683638715	1.198334668	0.486242579
SAA1	0.653828708	1.549841348	0.81120756	2.961027884	0.18466978
SAA2	0.213005492	0.03683707	0.000512146	2.649575844	0.130204014
SAA4	0.808180201	0.057677224	3.40E-05	97.95690902	0.452162078
SAAL1	0.343804584	6.305448807	0.16876196	235.5903226	0.318860074
SAC3D1	0.654527901	1.095369569	0.411579676	2.915193735	0.855272481
SACM1L	0.796222967	0.846607827	0.644286316	1.112463192	0.232057055
SACS	0.936123356	0.9835939	0.752649895	1.285401045	0.903570006
SAE1	0.693609776	2.807382228	7.68E-05	102648.5208	0.847304646
SAFB	0.206414937	1.423698443	0.826579138	2.452175674	0.202874205
SAFB2	0.347764709	0.528578473	0.216599818	1.289914297	0.161309741
SAG	0.067445447	0.000286887	8.58E-09	9.588413399	0.124872692
SAGE1	0.822599191	1.054954968	0.767460212	1.45014682	0.741734142
SALL1	0.621740029	1.583023918	0.553957008	4.52375309	0.391218915
SALL2	0.437529277	10.63599071	0.157103254	720.063368	0.271618851
SALL3	0.161585378	0.928349406	0.782654013	1.101166806	0.393348657
SALL4	0.364604318	28.95813292	0.068730107	12200.9625	0.275012542
SAMD1	0.992152182	1.337222087	0.002597484	688.4211251	0.92731848
SAMD10	0.850278728	0.953348413	0.394353101	2.304719284	0.91552195
SAMD11	0.931886042	5.327835691	0.007232859	3924.565863	0.619436305

SAMD12	0.574709012	0.030450385	1.48E-05	62.51260329	0.369573412
SAMD13	0.106885449	0.402906832	0.113593737	1.429074513	0.159348984
SAMD14	0.255065138	1.726673571	0.613265447	4.861518996	0.301056426
SAMD3	0.73903368	0.85088554	0.210368253	3.441613427	0.820826355
SAMD4A	0.525089005	1.20570186	0.295965156	4.911784196	0.794070373
SAMD4B	0.672398611	0.82914258	0.370625553	1.854911009	0.648341859
SAMD7	0.098554689	709.942438	0.009592416	52543412.07	0.251109
SAMD8	0.537168945	0.940284498	0.24934001	3.545900779	0.927558297
SAMD9	0.134322742	2.123373604	5.97E-05	75535.40675	0.887999872
SAMD9L	0.747908034	0.77741742	0.063384157	9.535156388	0.84393895
SAMHD1	0.245671662	2.505220762	0.402701682	15.58506295	0.324766911
SAMM50	0.605480655	0.782182986	0.023448476	26.09168431	0.890805471
SAMSN1	0.186998054	0.332899441	0.067053998	1.652728271	0.178495656
SAP130	0.533500328	0.816173235	0.325549765	2.046196377	0.66489649
SAP18	0.063585908	0.147781758	0.010440924	2.091715997	0.157319486
SAP30	0.644072986	2.819991976	0.196412715	40.4879834	0.445659851
SAP30BP	0.368627677	1.516847899	0.557133917	4.129756742	0.414902332
SAR1A	0.428122409	1.372938222	0.526712093	3.578728089	0.516716621
SAR1B	0.381965004	300.7951912	0.009061909	9984402.423	0.282652927
SARDH	0.836884585	0.070671082	0.002602536	1.919052201	0.115717771
SARM1	0.631922744	0.695361528	0.282252329	1.713104216	0.429649097
SARS	0.225733644	0.462393509	0.046876443	4.561091756	0.508939314
SARS2	0.594902255	1.019628929	0.701491291	1.482047128	0.918856951
SART1	0.452393249	0.169674532	0.015432972	1.865450585	0.146996573
SART3	0.614417245	0.782202643	0.261729207	2.337687039	0.660112521
SASH1	0.654182314	1.991869373	0.457637172	8.669627041	0.358473334
SASS6	0.784073561	1.130404899	0.018965461	67.37591254	0.953133451
SAT2	0.147249237	205.5748109	0.333163471	126847.6484	0.104232843
SATB1	0.951352708	1.092675114	0.896079453	1.332402947	0.38116595
SATB2	0.803596507	44.23736057	0.11385416	17188.16485	0.212871464
SATL1	0.95786172	0.977686695	0.782484866	1.22158436	0.84258466
SAV1	0.067341956	1.757515121	0.745711384	4.142164736	0.197340031
SBDS	0.817399264	1.171390435	0.384084767	3.57253312	0.780973572

SBF1	0.746625928	1.127993912	0.295619158	4.304085953	0.860074702
SBF2	0.891081793	11.78456073	0.096259435	1442.724779	0.314568033
SBK1	0.094397004	1.297325982	0.588292107	2.860916683	0.518846027
SBNO1	0.802069097	1.048615234	0.922942399	1.191400363	0.466111615
SBSN	0.897369891	0.391043455	0.098325045	1.555198718	0.18252653
SCAMP1	0.628377472	2.947609405	0.545063453	15.94016469	0.209379503
SCAMP2	0.589215839	0.026879042	1.73E-05	41.8300567	0.334868093
SCAMP3	0.716498343	1.392109489	0.651234444	2.975838962	0.393391707
SCAMP4	0.33410838	1.185895741	0.461917714	3.04458709	0.723024296
SCAMP5	0.743685555	0.003491932	3.52E-06	3.468792388	0.108117126
SCAND1	0.86998445	0.90315527	0.088764635	9.189351591	0.93142134
SCAP	0.901880194	3.130675689	0.557180404	17.5905868	0.195023085
SCARA3	0.720742735	1.061662086	0.846116715	1.332116911	0.605306621
SCARA5	0.433673264	0.107706228	0.000109234	106.2002521	0.526375091
SCARB1	0.476123357	2.075378819	0.680099657	6.333185432	0.199597596
SCARB2	0.167544316	1.739099545	0.469241159	6.445443178	0.407715711
SCARF1	0.394303949	1.387515708	5.79E-06	332467.5655	0.958669912
SCARF2	0.250286828	1.416223322	0.509533838	3.936320511	0.504639329
SCCPDH	0.442236107	0.054248408	0.000993414	2.962399399	0.15333271
SCD	0.853248132	1.096971185	0.471753878	2.550791498	0.829792766
SCD5	0.705758239	1.503896525	0.254097482	8.900933384	0.652857145
SCEL	0.643429662	2.497259974	0.002891007	2157.140403	0.790782604
SCFD1	0.577745822	1.344994283	0.363670122	4.974314662	0.656928372
SCFD2	0.069375574	97.40716409	0.010871927	872720.657	0.324056864
SCG2	0.573613979	0.139549875	0.004461143	4.365286993	0.262263143
SCG3	0.321673514	0.495437179	0.18451795	1.330266233	0.163419631
SCGB1A1	0.509405182	2.049577048	0.434264157	9.673296792	0.364709695
SCGB1C1	0.217699078	1.433081459	0.36158736	5.679740753	0.60855765
SCGB1D1	0.469895298	1.782801677	0.273904516	11.60397742	0.545192817
SCGB1D2	0.661351869	20.99488922	0.013193211	33410.01469	0.418323181
SCGB1D4	0.962675154	0.994952795	0.818685228	1.209171768	0.959437442
SCGB2A1	0.948118508	1.043396504	0.625391636	1.740791213	0.870781768
SCGB2A2	0.186211262	1.290865294	0.152175646	10.95006495	0.814947147

SCGB3A1	0.322423262	0.677373384	0.049272766	9.312136022	0.770817221
SCGB3A2	0.787639176	1.151974028	0.79203343	1.675490087	0.459195333
SCGN	0.675854603	1.030028082	0.841080905	1.261421872	0.774769555
SCHIP1	0.768140546	1.039626259	0.389215599	2.776925595	0.93820614
SCIN	0.969870022	0.977459728	0.427525537	2.234784678	0.956907514
SCLY	0.601982335	0.374320611	0.049545159	2.828044592	0.340900665
SCMH1	0.382063714	0.497497833	0.151718452	1.631338119	0.249216614
SCML1	0.524235295	0.807112606	6.07E-06	107407.7355	0.971603216
SCML2	0.11784865	0.405783606	0.124305165	1.32464596	0.135122963
SCML4	0.767495648	0.783049348	0.314506642	1.94961314	0.59925713
SCN10A	0.14635706	0.128498212	0.00397535	4.153543669	0.247268837
SCN11A	0.101171705	0.389946984	0.076888763	1.977644631	0.255617069
SCN1A	0.828023044	1.656547757	0.402410425	6.819282756	0.484478726
SCN1B	0.371369318	0.475844572	0.023330029	9.70543412	0.629288678
SCN2B	0.849208594	1.009915852	0.741633349	1.375248335	0.950058837
SCN3A	0.358971074	2.419146142	0.84880235	6.894735927	0.098291623
SCN3B	0.851369887	0.582459297	0.00574824	59.01960571	0.818574938
SCN4A	0.202125355	0.381089702	0.127241775	1.141365408	0.084760038
SCN4B	0.079273914	3.079784431	0.869400002	10.90990582	0.081317353
SCN5A	0.22657744	2.194796379	0.855240051	5.632490129	0.102097835
SCN7A	0.91396972	0.982060622	0.791118619	1.21908781	0.869650684
SCN8A	0.618758797	0.300440798	4.48E-06	20136.96477	0.832040453
SCN9A	0.780830963	17.14175477	0.152385736	1928.262866	0.238311975
SCNM1	0.405595049	0.895616429	0.739101469	1.085275597	0.26062043
SCNN1A	0.937480399	0.980259191	0.241913416	3.972115718	0.97771936
SCNN1B	0.40912325	1.732929008	0.803585636	3.73705404	0.160838165
SCNN1D	0.399679603	0.427111501	0.036798887	4.957330238	0.496429926
SCNN1G	0.195450329	1.371352041	0.675500672	2.784018579	0.382062006
SCO1	0.556546858	13.3102688	0.006950857	25487.97293	0.502017904
SCO2	0.18924865	1.221161289	0.887494178	1.680275691	0.219820994
SCOC	0.070844117	1.362001049	0.720223444	2.575654642	0.341913466
SCP2	0.365887922	0.001713199	5.98E-07	4.91201253	0.116857315
SCPEP1	0.895975494	1.613080782	0.11114687	23.41073229	0.726091362

SCRG1	0.498039107	0.623581411	0.257222901	1.511738555	0.295888115
SCRIB	0.86124631	1.009743543	0.548444848	1.859042029	0.975160772
SCRN1	0.10571008	0.000197981	3.31E-08	1.1835877	0.054608966
SCRN2	0.134381555	0.27575741	0.054103758	1.405487372	0.121062831
SCRN3	0.913443064	1.11444991	0.771201885	1.610471428	0.564028038
SCRT1	0.614677966	0.009999649	4.42E-06	22.61573513	0.242567751
SCRT2	0.914585345	0.645583738	0.215953145	1.929948103	0.433507677
SCT	0.858801524	0.43896761	0.007402493	26.03076604	0.692650202
SCTR	0.422308355	1.873310613	0.794653529	4.416129202	0.151390043
SCUBE1	0.841666303	1.946698135	0.381451405	9.934774332	0.423115708
SCUBE2	0.256627211	1.594456119	0.597970423	4.251531876	0.351161942
SCUBE3	0.728938484	0.006425819	1.05E-07	394.0049345	0.369504058
SCYL1	0.809491936	0.92089156	0.530761692	1.5977816	0.769418186
SCYL2	0.878612562	0.918686174	0.55815669	1.512092035	0.738694407
SCYL3	0.135453684	0.011074635	1.30E-05	9.414240738	0.19072103
SDAD1	0.529098824	0.976154284	0.17360793	5.488673148	0.978146194
SDC1	0.664738621	1.004497057	0.745478514	1.3535123	0.976474003
SDC2	0.695623237	0.874609092	0.006637141	115.2515907	0.95709621
SDC3	0.407394032	2.547420761	0.000552304	11749.59234	0.828022957
SDC4	0.858849123	0.354773826	0.008691693	14.4810069	0.583975632
SDC4P	0.448496848	1.614142665	0.542034269	4.806811472	0.38979879
SDCBP	0.292021606	0.150471224	0.000911513	24.83955885	0.467253395
SDCBP2	0.904855344	1.186162879	0.549595073	2.56003455	0.663592708
SDCCAG3	0.700281296	143.9456261	0.011715767	1768586.21	0.300962094
SDCCAG8	0.27844658	1.152549854	0.913642492	1.453928836	0.230946033
SDF2	0.912688411	0.929139312	0.571771692	1.509868139	0.76669994
SDF2L1	0.209750362	0.377898737	0.026131833	5.464884772	0.475258018
SDF4	0.341777635	0.001467205	2.05E-08	104.77782	0.252550734
SDHA	0.91933721	1.298051257	0.547040004	3.080098445	0.554053208
SDHB	0.796191435	0.857159518	0.345494905	2.126579662	0.73953929
SDHC	0.516725573	1.526787726	0.439781597	5.300541844	0.505176
SDHD	0.525110154	1.012566359	0.304771036	3.364134086	0.983736097
SDK1	0.265584234	4.630875563	0.601880721	35.62999735	0.140941975

SDK2	0.56464628	95.21773641	0.002601003	3485739.334	0.395425574
SDPR	0.072202947	0.00346041	3.55E-07	33.69189201	0.226542623
SDS	0.707502376	1.113076351	0.164256245	7.542720584	0.912622294
SDSL	0.813232034	0.648082796	0.315475309	1.331360329	0.237679036
SEC14L1	0.366371243	0.085065633	0.002560308	2.82628595	0.167986512
SEC14L2	0.373659214	0.299432683	0.068949998	1.300361622	0.10752349
SEC14L3	0.979295663	1.033989792	0.82946179	1.288950141	0.766286821
SEC14L4	0.586369717	2.039834581	0.004640946	896.5683136	0.818412955
SEC14L5	0.685896134	1.182713272	0.456396945	3.064899318	0.729783595
SEC23A	0.819484353	0.781644786	0.518499403	1.178339971	0.239454772
SEC23B	0.82044991	1.034126188	0.724736953	1.475593273	0.853224581
SEC23IP	0.230760985	3.417714091	0.472896964	24.70045381	0.223277811
SEC24B	0.367549622	1.190225442	0.934537673	1.515868908	0.158160897
SEC24C	0.689141348	0.689762995	0.146381767	3.250220297	0.638639102
SEC24D	0.769812062	0.018145923	4.25E-05	7.755710462	0.194562197
SEC61A1	0.606909539	3.518758706	0.210623786	58.78568156	0.381181635
SEC61A2	0.583136223	0.590989621	0.225450892	1.549200933	0.284758703
SEC61B	0.963868427	0.046421021	5.91E-05	36.4420785	0.366689649
SEC61G	0.825037791	0.865683875	0.402944214	1.85983207	0.711626752
SEC63	0.56792937	0.994403169	0.32489416	3.043568593	0.992153971
SECISBP2	0.107311045	0.38331043	0.116531736	1.260831519	0.114462655
SECTM1	0.905597355	0.852235621	0.269777659	2.692237594	0.785280472
SEH1L	0.514910542	0.672605629	0.373251692	1.212046298	0.18685888
SEL1L	0.058381672	1475.729316	0.143779073	15146689.76	0.121524521
SELE	0.104279504	1.078077923	0.894196237	1.29977287	0.430740788
SELENBP1	0.87686071	0.802318139	0.081408995	7.907165478	0.850350927
SELK	0.954458119	0.947763603	0.74478861	1.206054758	0.662612866
SELL	0.9652599	2.312725932	0.320426508	16.69244303	0.405747668
SELM	0.493877735	0.578688232	0.131351271	2.54950003	0.469698553
SELO	0.348461634	0.82896766	0.250591973	2.742256154	0.758615914
SELP	0.817187206	0.058616638	4.03E-06	852.3105465	0.561859534
SELPLG	0.011190205	0.635989475	0.45094155	0.896973482	0.009887602
SELT	0.303186018	0.532811196	0.177782071	1.596830146	0.260913334

SELV	0.228744671	1.148226058	0.339729216	3.880805704	0.823965126
SEMA3A	0.096760405	2.258252803	0.542389194	9.402299638	0.262999658
SEMA3B	0.457605168	1.123315435	0.483418983	2.610235866	0.786921809
SEMA3C	0.840321272	1.43046785	0.493100734	4.149736813	0.510012389
SEMA3D	0.584197124	0.690713238	0.117439431	4.062390051	0.682299654
SEMA3E	0.5534434	1.831893784	0.472037539	7.109254152	0.381604566
SEMA3F	0.21373471	1.215760942	0.825153623	1.791272106	0.323135844
SEMA3G	0.728317005	0.904326362	0.178660125	4.577440931	0.903262516
SEMA4A	0.565948721	1.069426216	0.808958442	1.413759191	0.637418217
SEMA4B	0.980970328	1.532328361	0.52464254	4.475485741	0.435135455
SEMA4C	0.461693982	0.98231755	0.789578786	1.222104477	0.872806656
SEMA4D	0.557270414	1.046666891	0.889474664	1.231638882	0.582778279
SEMA4F	0.591623765	0.831584307	0.369333344	1.872380252	0.656066082
SEMA4G	0.891125729	0.796146282	0.212212168	2.98686408	0.73541152
SEMA5A	0.87649277	1.289959547	0.770329672	2.16010845	0.333065617
SEMA5B	0.354674616	0.914131145	0.298573137	2.798764009	0.875039612
SEMA6A	0.532256249	1.081643414	0.60881579	1.921685499	0.788972843
SEMA6B	0.594055348	1.373744815	0.7821833	2.412701494	0.269140713
SEMA6C	0.672087694	65.37407762	0.007179361	595285.5468	0.368827431
SEMA6D	0.268212424	21.05167629	0.698834759	634.1600341	0.079478918
SEMA7A	0.663952027	0.123391437	0.00104071	14.62986329	0.390467932
SEMG1	0.972228181	56.03453554	0.170979133	18364.04893	0.173099786
SEMG2	0.530920002	0.71749187	0.280295959	1.83661079	0.488754247
SENP1	0.929884213	0.995901081	0.397974102	2.492169613	0.992997541
SENP2	0.709959657	0.655732057	0.309956957	1.387239489	0.269672599
SENP3	0.837081349	1.075618821	0.462176442	2.503277416	0.865686091
SENP5	0.293221803	1.101089105	0.002540747	477.1814932	0.975200634
SENP6	0.913519535	1.152356673	0.532738604	2.492640651	0.718664231
SENP7	0.825908978	0.040087779	0.000123367	13.02643521	0.275683443
SENP8	0.822179738	0.692015733	0.221582242	2.161210083	0.526341358
SEPHS1	0.476805478	1.711479763	0.544578128	5.378774556	0.357705582
SEPHS2	0.279061954	1.658413502	0.715441609	3.844248518	0.238272607
SEPN1	0.897386117	5.846967559	0.031596865	1081.975373	0.507345965

SEPP1	0.732799218	9.856067662	0.000676276	143642.6534	0.639944658
SEPW1	0.169213707	0.76468429	0.431526866	1.35505367	0.358047482
SERAC1	0.846444938	0.936155775	0.021313344	41.11919973	0.972729108
SERBP1	0.303557753	0.011838964	1.01E-05	13.83600897	0.218334984
SERF1A	0.731665871	0.354333324	0.078808162	1.5931358	0.17613264
SERF1B	0.92269159	1.146929838	0.822735874	1.59887042	0.418632914
SERF2	0.126535332	0.04518901	0.000314066	6.501967705	0.221883512
SERGEF	0.744110006	0.715135577	0.318167347	1.607389631	0.417140004
SERHL	0.857840924	0.635268311	0.16472596	2.449922447	0.510011668
SERHL2	0.458799485	1.072414915	0.537004817	2.141645127	0.842956716
SERINC1	0.353100118	0.049786104	0.001067874	2.321112607	0.125915683
SERINC2	0.168465823	0.001294843	1.98E-08	84.54757796	0.239790237
SERINC3	0.632759977	817.4362903	0.020437295	32695231.22	0.214831807
SERINC4	0.569207948	2.108954823	0.558745584	7.960135301	0.270862822
SERINC5	0.195709146	0.423177667	0.071385165	2.508635193	0.343604701
SERP1	0.534771917	0.596316907	1.87E-05	19032.26755	0.922167934
SERPINA1	0.657364941	1.404609145	0.556309941	3.546452624	0.472150348
SERPINA10	0.339715376	1.276145061	0.304047846	5.35621691	0.738994673
SERPINA11	0.874859972	0.954613993	0.183193963	4.974442726	0.956020251
SERPINA12	0.471136174	1.697501296	1.136657936	2.53507283	0.009711436
SERPINA2	0.51792151	0.000598654	2.61E-09	137.2579526	0.238639472
SERPINA3	0.638708077	1.944672909	0.912348944	4.145072726	0.084996642
SERPINA4	0.126384436	0.902000581	0.369365916	2.202707436	0.820877443
SERPINA5	0.978778764	0.812498914	0.440353315	1.499147304	0.506435803
SERPINA6	0.443262315	2.169603447	0.030034378	156.726374	0.722818135
SERPINA7	0.158879024	0.191752975	0.01344133	2.735533046	0.223269273
SERPINA9	0.535661286	0.793011708	0.523385312	1.201538436	0.273986014
SERPINB1	0.687702846	6.525685001	0.078172646	544.7502058	0.406028877
SERPINB11	0.584619652	1.29429548	0.845325337	1.98172315	0.235280578
SERPINB12	0.641804172	0.86914064	0.375937805	2.009389432	0.742916226
SERPINB13	0.768011999	0.301426803	0.001443313	62.95106642	0.6599173
SERPINB2	0.111740645	1.304032891	0.992755816	1.71291042	0.056428079
SERPINB3	0.085205817	1.139812602	0.917230441	1.416408255	0.237778441

SERPINB4	0.464123261	1.368166592	0.191120379	9.794245059	0.75493391
SERPINB5	0.31224484	0.154455507	0.012122488	1.967954335	0.150274483
SERPINB6	0.357841579	0.860355737	0.535226371	1.382988648	0.534550578
SERPINB7	0.57355737	0.683699566	0.263101155	1.776674436	0.435165441
SERPINB8	0.468352137	0.893135465	0.21937537	3.636191979	0.874633855
SERPINB9	0.211880834	0.640457785	0.212731586	1.92818651	0.428148653
SERPINC1	0.489313998	1.30868248	0.367582094	4.659230847	0.677973564
SERPIND1	0.386689398	6.165543396	0.032699283	1162.530842	0.496220421
SERPINE1	0.536682575	1.800706825	0.663538681	4.886746111	0.248205732
SERPINE2	0.490861317	0.134738031	0.008592628	2.112780603	0.153487527
SERPINF1	0.975175265	6.347269549	0.000843294	47774.34435	0.684905849
SERPINF2	0.350817956	0.940013835	0.557582303	1.584745436	0.816426901
SERPING1	0.251343175	0.583812441	0.270252518	1.261179612	0.170849431
SERPINH1	0.131699952	52.39268618	0.324529537	8458.378214	0.126978793
SERPINI1	0.518811882	0.457194281	0.025364362	8.240956803	0.595794261
SERPINI2	0.69482571	0.883048099	0.07480499	10.42408996	0.921334253
SERTAD1	0.642371447	229.661621	0.010010528	5268898.865	0.288583786
SERTAD2	0.382645293	1.084563374	0.884360027	1.330089191	0.435588461
SERTAD3	0.115204219	0.80347289	0.507875244	1.271116663	0.349820097
SERTAD4	0.809410674	0.214700727	0.000110863	415.7958056	0.690330227
SESN1	0.455204078	0.691170897	0.241474875	1.978330905	0.491193775
SESN2	0.390947136	0.715451251	0.434782462	1.177302531	0.187621469
SESN3	0.404041573	1.011354144	0.748929613	1.365732088	0.941278733
SESTD1	0.803144761	0.485891734	0.037995533	6.213645606	0.57883694
SET	0.800663425	1.179014816	0.958604189	1.450104174	0.118858583
SETBP1	0.305495428	2.4585662	0.427396964	14.1427017	0.313585143
SETD1A	0.54264287	1.134918482	0.216209481	5.957370386	0.881076574
SETD3	0.878110719	19.05222637	0.013084731	27741.2911	0.427732688
SETD4	0.525568558	0.968816057	0.162521525	5.775263025	0.972254646
SETD6	0.287199004	1.005960816	0.401513715	2.52035516	0.989881123
SETD8	0.631545802	1.190912617	0.486596018	2.914682427	0.702014647
SETDB1	0.192798407	0.745836155	0.470652711	1.181915152	0.21187434
SETDB2	0.463655177	1.096501308	0.89830562	1.338425468	0.365116475

SETMAR	0.66011098	1.175054403	0.600128981	2.300760162	0.637966225
SETX	0.841456995	0.50612661	0.117278484	2.184238209	0.361367532
SEZ6	0.732099286	0.828736432	0.247376369	2.776352797	0.760717066
SEZ6L	0.902141021	1.281169363	0.809889461	2.026690082	0.28966269
SEZ6L2	0.76326243	1.268236138	0.758576468	2.120317424	0.36482052
SF1	0.33986366	0.279460374	0.006992215	11.16929403	0.498074147
SF3A1	0.348842227	0.915589239	0.690544335	1.213975137	0.540053372
SF3A2	0.871668277	0.970311231	0.581608022	1.618794532	0.908118576
SF3A3	0.358830026	1.022389168	0.377958058	2.765596844	0.965214478
SF3B1	0.948713374	6.399878126	8.04E-09	5095035014	0.859102927
SF3B2	0.934169728	1.00117401	0.563121102	1.779989056	0.996811337
SF3B3	0.290795417	0.411682842	0.140662928	1.204885789	0.105277216
SF3B4	0.215350066	1.538566021	0.013847375	170.9483192	0.857725886
SF3B5	0.484524585	0.938254079	0.302215939	2.912886462	0.912198927
SFI1	0.914250652	0.870653943	0.500127074	1.515691365	0.624352689
SFMBT1	0.291944487	0.691215818	0.296321119	1.612370085	0.392793833
SFMBT2	0.346124738	0.677076757	0.1764963	2.597408178	0.569701145
SFN	0.438585188	1.005338076	0.835179002	1.2101653	0.955125464
SFPQ	0.296031466	5.208201813	0.002037988	13309.87532	0.680167784
SFRP1	0.357984288	0.586745742	0.163891204	2.10060429	0.412590389
SFRP2	0.57788087	1.002707379	0.744630925	1.350228758	0.985791851
SFRP4	0.17349293	1.497026119	0.456585804	4.908359346	0.505432883
SFRP5	0.959061692	1.36428226	0.385214634	4.831763693	0.630203959
SFT2D1	0.077708515	1.952496573	0.659103829	5.783979243	0.227202537
SFT2D2	0.149859397	0.623171488	0.263489749	1.473843686	0.281561619
SFTPA1	0.498400982	0.023348759	4.14E-05	13.16368784	0.2450362
SFTPA2	0.783872816	0.501179272	0.000202959	1237.595636	0.862399957
SFTPB	0.598286863	4.378815132	0.959052021	19.99268187	0.056649945
SFTPC	0.505582587	0.561000166	0.002082258	151.1441949	0.83957033
SFTPD	0.305863115	2.025721008	0.573132139	7.159859526	0.273141846
SFXN1	0.906258387	0.929280519	0.63770203	1.354178351	0.702629338
SFXN2	0.64689057	0.921326337	0.162357415	5.228231919	0.926291613
SFXN3	0.52030099	0.109302554	8.29E-06	1441.053747	0.647429062

SFXN4	0.56020722	3.94392633	0.578162894	26.90341263	0.161309095
SFXN5	0.776628249	0.455489777	0.091866148	2.258404651	0.335711089
SGCA	0.212787927	0.799080097	0.518437472	1.231641301	0.309580617
SGCB	0.164457694	0.517598544	0.024158568	11.08957507	0.673620542
SGCD	0.222492628	0.016974319	1.19E-05	24.22042982	0.27137152
SGCE	0.310588999	0.686408685	0.20525433	2.295478403	0.54126218
SGCG	0.539105834	1.159527157	0.015673364	85.78268286	0.946259139
SGCZ	0.786909575	1.065440023	0.776794743	1.461341561	0.69417239
SGIP1	0.919488995	1.048369935	0.817095571	1.345105223	0.710292658
SGK2	0.850050098	1.091848218	0.782254982	1.52396924	0.605503899
SGK3	0.886691232	1.52892797	0.847684012	2.757655806	0.158290665
SGOL1	0.304925794	0.939493357	0.700981797	1.259159327	0.676158733
SGOL2	0.222159744	0.823205815	0.017373448	39.00594836	0.921273878
SGPL1	0.794210449	0.989061719	0.243603128	4.015724638	0.987725579
SGPP1	0.376496765	0.097575843	0.000396674	24.00217572	0.407390912
SGPP2	0.991098551	1.334196101	0.463569598	3.839939559	0.592943577
SGSH	0.182970239	0.728189355	0.008566257	61.90098532	0.888712117
SGTA	0.863412305	1.06642269	0.515529928	2.205996765	0.862330944
SGTB	0.943142943	0.855624486	0.560468092	1.306217553	0.470067792
SH2D1A	0.308442372	0.00266654	1.90E-07	37.50199394	0.223897521
SH2D1B	0.195253281	0.554585556	0.171707476	1.791215774	0.324362948
SH2D2A	0.590466522	1.945355121	0.318432701	11.88447836	0.471117016
SH2D3A	0.258395324	0.545789026	0.172986062	1.722021171	0.301659436
SH2D3C	0.837341911	0.970949878	0.506154064	1.862562673	0.929322274
SH2D4A	0.84361809	0.966060154	0.619388374	1.506764187	0.878986583
SH2D4B	0.124956245	0.612644347	0.219804562	1.707576456	0.348830314
SH2D5	0.3335529	1.712025915	0.871820442	3.361968353	0.118387892
SH3BGR	0.19832205	0.144338639	0.013519951	1.540955512	0.109140341
SH3BGRL	0.620283334	3.339814097	0.019559534	570.2773093	0.64564822
SH3BGRL2	0.404636833	1.202389089	0.842212554	1.716596974	0.310282497
SH3BGRL3	0.549615446	0.01128604	2.98E-05	4.280409826	0.138861701
SH3BP1	0.797105671	1.131211637	0.484752227	2.639781102	0.775525088
SH3BP2	0.197160575	0.832153873	0.346199445	2.000234487	0.681347021

SH3BP4	0.923539305	0.54673984	0.001748854	170.9259338	0.836801953
SH3BP5	0.677102167	1.699535908	0.32132306	8.989153489	0.532586926
SH3BP5L	0.068343751	0.704816413	0.390471225	1.272222237	0.245666991
SH3D19	0.41806789	1.145118375	0.236543781	5.543566136	0.86626754
SH3GL1	0.118007083	0.055565997	0.000353235	8.740869371	0.262757614
SH3GL2	0.430998861	3.397445603	0.030501635	378.426815	0.611024968
SH3GL3	0.17029255	0.777221717	0.189793489	3.182794105	0.726049766
SH3GLB1	0.338966589	0.463630097	0.075278087	2.855450722	0.407252375
SH3GLB2	0.689779358	1.626031586	0.457088935	5.784385746	0.452753276
SH3KBP1	0.918489502	0.844622332	0.310436185	2.298014594	0.740894243
SH3PXD2A	0.155439141	75.5322162	0.366477504	15567.43764	0.111671594
SH3PXD2B	0.2057064	37.25111018	0.028845751	48105.70588	0.322263761
SH3RF2	0.613574402	1.056257673	0.831569294	1.341656407	0.653780126
SH3TC1	0.774157414	0.219170249	0.000685608	70.0627443	0.605961419
SH3TC2	0.716518466	1.078693572	0.556437964	2.091122278	0.822531972
SH3YL1	0.460172813	2.08705216	0.659067364	6.609015944	0.210920782
SHANK1	0.71286018	0.905977311	0.594363172	1.380965253	0.646149936
SHANK2	0.300065352	0.02625765	4.89E-05	14.09869829	0.256415408
SHANK3	0.495975019	0.409741059	0.129950811	1.291932954	0.127808418
SHARPIN	0.649694152	1.092300724	0.80759126	1.4773821	0.566644319
SHB	0.623183406	1.639424784	0.215948902	12.44606291	0.632662459
SHBG	0.832598924	0.962630671	0.001975425	469.0927799	0.990376693
SHC1	0.93938631	0.992887748	0.87569342	1.125766229	0.911314614
SHC2	0.803490852	1.182052893	0.904539829	1.544707039	0.220544618
SHC3	0.137426678	0.68674396	0.352680112	1.337238054	0.269048987
SHC4	0.716242531	1.03155233	0.426662188	2.494011046	0.945015983
SHCBP1	0.748985957	0.975695392	0.704457129	1.351368961	0.882300123
SHD	0.504405809	0.998496515	0.853014097	1.168791106	0.985059055
SHE	0.078106911	2.385241641	0.679926047	8.367641907	0.174612844
SHF	0.985801497	1.214282694	0.481759354	3.060620306	0.6806132
SHFM1	0.104601577	0.650070761	0.334509683	1.263317674	0.203924861
SHH	0.959129773	0.966993309	0.67212972	1.391213677	0.856482659
SHKBP1	0.545687951	1.178566271	0.1940311	7.158741329	0.858330692

SHMT1	0.783783441	0.944986407	0.343542809	2.599382917	0.912723225
SHMT2	0.763708079	1.016654997	0.322031663	3.209583101	0.977533882
SHOX	0.933127335	1.741393978	0.052783996	57.45023573	0.755836298
SHOX2	0.549612315	0.353094999	0.108614293	1.147879109	0.083508802
SHPRH	0.439815163	1.284270688	0.688538195	2.395438934	0.431498971
SHQ1	0.962460843	1.47940297	0.427386287	5.120971854	0.536456703
SI	0.312579394	1.972146387	0.857540839	4.535482389	0.109982357
SIAE	0.859098612	1.651478233	0.525845041	5.186661733	0.390243948
SIAH1	0.665103113	0.920319701	0.428472188	1.976763899	0.831422375
SIAH2	0.062428154	0.919582879	0.451272477	1.873884879	0.81744937
SIDT1	0.350575997	1.713486574	0.554056313	5.299165748	0.349849049
SIDT2	0.522673233	0.709274204	0.184046987	2.733377511	0.617728855
SIGIRR	0.374685637	1.030567802	0.820923476	1.293750302	0.795266822
SIGLEC10	0.235936417	735.6392045	0.013949517	38794534.66	0.23410848
SIGLEC11	0.050267934	1.196026448	0.096863609	14.76797404	0.888987225
SIGLEC12	0.077708515	2.633812926	0.873561291	7.941023256	0.085451463
SIGLEC5	0.879835868	0.640086466	0.011167031	36.68931058	0.829000035
SIGLEC6	0.563269296	0.495441982	0.169540145	1.447814959	0.199278493
SIGLEC7	0.91810428	1.039587921	0.701894777	1.539750801	0.846391225
SIGLEC8	0.408141724	5.076154321	0.555847175	46.35688346	0.149988643
SIGLEC9	0.552430693	0.006693575	3.29E-05	1.360591383	0.064833388
SIL1	0.715692688	0.802095589	0.304224112	2.114748007	0.655712463
SIM1	0.743244762	0.508025045	0.176671037	1.460847515	0.208876945
SIM2	0.326307394	1.405566332	0.774398128	2.551164116	0.262993996
SIN3A	0.269566614	1.831316293	0.767767355	4.368145304	0.172525278
SIN3B	0.787237717	1.341514474	0.464632543	3.873299692	0.587072149
SIPA1	0.92392415	0.304440581	0.018667366	4.965031823	0.403742722
SIPA1L1	0.503909387	0.185131149	0.001007312	34.02474521	0.526041092
SIPA1L2	0.568218775	4.433101744	0.000477238	41179.429	0.74939391
SIPA1L3	0.958713127	1.030166524	0.85090859	1.247188101	0.760589774
SIRPB1	0.463445724	1.869524971	0.337241019	10.36387455	0.473967079
SIRPB2	0.64033352	1.151909713	0.494916308	2.681051249	0.742831083
SIRPD	0.305542707	1.352450408	0.875304755	2.089697441	0.173822022

SIRT1	0.104973789	6.032248917	0.343625922	105.8943014	0.218965822
SIRT2	0.335492931	5.128557417	0.681339693	38.60350636	0.112422035
SIRT3	0.977504211	2.706141165	0.874474492	8.374400936	0.084123963
SIRT4	0.403170993	1.067805809	0.304120866	3.749197682	0.918453496
SIRT5	0.369145409	3.007252763	0.020398089	443.3537539	0.665617718
SIRT6	0.783733032	1.477429981	0.818772828	2.665940142	0.19496705
SIRT7	0.054963834	3.430800346	0.720502479	16.33636435	0.121557173
SIT1	0.082818501	0.000280768	7.95E-09	9.916507053	0.125872363
SIX1	0.535542504	1.078486312	0.895779805	1.298458302	0.424971003
SIX2	0.480359026	0.575816831	0.16876922	1.964606005	0.378044853
SIX3	0.343174156	0.887607163	0.709698044	1.110115045	0.296182105
SIX4	0.858303422	0.793084767	0.000468017	1343.932679	0.951271059
SIX5	0.207753874	0.553666672	0.261213703	1.173547865	0.122967353
SIX6	0.989379663	0.926854373	0.409806926	2.096253073	0.855251489
SKI	0.266864593	0.104271064	0.002209843	4.920012737	0.250269193
SKIL	0.2521881	0.799225052	0.163798827	3.899665793	0.781679901
SKIV2L	0.297984517	0.171290782	1.15E-06	25547.39244	0.771593534
SKIV2L2	0.463471834	0.066553211	0.000235909	18.77555087	0.346558974
SKP2	0.764847901	0.590376398	0.205191506	1.698629237	0.32839052
SLA	0.122620445	22.30305741	0.018841872	26400.04998	0.389832277
SLA2	0.87747568	0.891075288	0.432591473	1.83548502	0.75443762
SLAMF1	0.674159984	1.576002154	0.659739218	3.764794817	0.305906105
SLAMF6	0.678377567	1.045103106	0.759506689	1.438092012	0.786484467
SLAMF7	0.564201885	0.697475454	0.169514112	2.869802423	0.617629793
SLAMF8	0.096012503	4742.537118	0.005042976	4459996803	0.227751647
SLAMF9	0.602786132	2.398104347	0.390012428	14.74543897	0.345227738
SLBP	0.406002782	0.544523816	0.128513747	2.30719432	0.409311032
SLC10A1	0.56201712	1.177381175	0.779193094	1.779053796	0.438144741
SLC10A2	0.193388812	0.003286824	8.88E-08	121.6976569	0.286720439
SLC10A3	0.223655911	0.45675099	0.042184064	4.94550426	0.519087948
SLC10A4	0.134800179	0.064915769	0.00079289	5.314805566	0.223711054
SLC10A5	0.600532071	0.794713686	0.391808657	1.611934372	0.524257625
SLC10A6	0.855171116	1.364974052	0.61439046	3.032524564	0.444909391

SLC11A1	0.728312115	0.992366265	0.209394785	4.703034055	0.992297922
SLC11A2	0.85513736	0.777443914	0.042607132	14.18586553	0.865083134
SLC12A1	0.090360905	1.530971772	0.96203809	2.436363581	0.072383238
SLC12A2	0.25695611	2.29031772	0.563054466	9.316248376	0.247024276
SLC12A3	0.415175031	0.577472669	0.172405165	1.934249953	0.373305769
SLC12A5	0.399759214	0.728405226	0.252534942	2.100993112	0.557651118
SLC12A6	0.662891363	0.339919746	3.07E-06	37669.86577	0.855525948
SLC12A7	0.460554395	1.053832184	0.497364509	2.232894089	0.891137463
SLC12A8	0.065922373	2.33576256	0.000682888	7989.288978	0.838098167
SLC12A9	0.625455969	1.066329444	0.856497549	1.327567703	0.565674354
SLC13A1	0.992205255	0.626277705	0.239831538	1.635413618	0.33930175
SLC13A2	0.308854158	0.075561586	0.002425169	2.354290975	0.141026201
SLC13A3	0.363955826	1.317497914	0.942429444	1.841836295	0.106726138
SLC13A4	0.815779376	0.509696301	0.114552001	2.267881105	0.376235116
SLC13A5	0.35851527	0.495172477	0.178494945	1.373684738	0.176987034
SLC14A1	0.077901917	0.017440972	0.000287188	1.059193587	0.053295843
SLC14A2	0.242392271	1.071230087	0.835845046	1.372902675	0.586765064
SLC15A1	0.072761519	1.202776728	0.899384388	1.608513417	0.213156929
SLC15A2	0.775581409	0.090714719	0.00456589	1.802312474	0.115554732
SLC15A3	0.580319358	0.874683405	0.056835401	13.46117117	0.923523316
SLC15A4	0.666152158	1.0500877	0.797739751	1.382260538	0.727446574
SLC16A1	0.557496672	0.067465533	1.03E-05	442.056984	0.547612603
SLC16A10	0.856937144	1.208189436	0.239975949	6.082783367	0.818612943
SLC16A11	0.510175647	0.665493234	0.258769633	1.71148848	0.398127915
SLC16A12	0.501467514	1.443252572	0.547900385	3.801745797	0.457813966
SLC16A13	0.564536607	1.061401991	0.893638699	1.260659578	0.497221029
SLC16A14	0.280429896	0.498328579	0.08155844	3.044827398	0.450712649
SLC16A2	0.74762804	1.246946649	0.535351693	2.904400911	0.608941639
SLC16A3	0.048372965	1.414208004	1.008362304	1.983398497	0.044620092
SLC16A4	0.18390896	0.139539257	0.002791572	6.974996075	0.323757589
SLC16A5	0.313369543	0.002578837	5.54E-06	1.201219621	0.05723967
SLC16A6	0.248230641	4.186819907	0.29034146	60.3753282	0.292946497
SLC16A7	0.471709154	0.248973862	9.80E-07	63254.37327	0.826673723

SLC16A8	0.291845566	0.386119757	0.029672209	5.024515303	0.467302153
SLC16A9	0.473071837	1.11895753	0.315854658	3.964057273	0.861736526
SLC17A1	0.057725947	0.34825307	0.091316828	1.328125416	0.122474173
SLC17A2	0.104016159	1.716260192	0.971008747	3.033493834	0.06306596
SLC17A3	0.97586832	0.875589223	0.172862433	4.435067097	0.872486264
SLC17A4	0.392090112	0.576379765	0.230406826	1.441856735	0.238889812
SLC17A5	0.647020017	0.9224236	0.691337101	1.230753127	0.583125548
SLC17A6	0.419919578	0.379582736	0.010283818	14.01065817	0.598789695
SLC17A7	0.902430922	0.225319352	0.002999738	16.92441252	0.498868632
SLC17A8	0.086342105	0.584617548	0.275301797	1.241465482	0.162398815
SLC18A1	0.747661835	1.41487408	0.140967961	14.20087695	0.768047104
SLC18A2	0.948642811	0.678310389	0.316408961	1.454146501	0.318459786
SLC19A1	0.264476458	1.140500316	0.246507849	5.276671621	0.866417153
SLC19A2	0.314422623	0.031331943	3.85E-05	25.4962446	0.311144617
SLC19A3	0.485831638	2.561765391	0.002542776	2580.896628	0.789761524
SLC1A1	0.944963978	120.9524685	0.001936461	7554761.255	0.394677947
SLC1A2	0.9846754	0.648715261	0.000261915	1606.749324	0.913568776
SLC1A3	0.712761906	1.009395953	0.384049878	2.652989231	0.984866377
SLC1A4	0.275816937	0.791355102	0.434124065	1.442543612	0.444936679
SLC1A5	0.513053507	0.168939531	0.010654592	2.678710213	0.207256718
SLC1A6	0.517801116	1.005732779	0.856104428	1.181512896	0.944547445
SLC1A7	0.053984903	2.032344014	0.926701567	4.457122268	0.076731049
SLC20A1	0.260392695	0.557728106	0.165876235	1.875257413	0.345309302
SLC20A2	0.864488611	0.817029333	0.59281709	1.126041981	0.216951716
SLC22A1	0.93163909	13.96475799	0.059982321	3251.199051	0.343065229
SLC22A11	0.424511583	0.950427574	0.383235092	2.357071657	0.912634567
SLC22A12	0.241058036	0.417005945	0.048002034	3.622637313	0.427794201
SLC22A13	0.250252676	1.698438944	0.143923217	20.04329048	0.674019647
SLC22A14	0.077804036	2.356468204	0.859982348	6.4570423	0.09558106
SLC22A15	0.997794145	11.67367496	0.185104483	736.2041422	0.245160609
SLC22A16	0.714125469	15.49665241	0.050899063	4718.087597	0.347567346
SLC22A17	0.338520843	0.247330285	8.35E-06	7330.366443	0.790300698
SLC22A18	0.452344846	0.928934107	0.638111632	1.352300338	0.700421638

SLC22A18AS	0.474659343	0.943075179	0.384654259	2.312182362	0.898077028
SLC22A2	0.192663626	0.667886634	0.195958193	2.27636594	0.518819587
SLC22A3	0.915198282	2.452437637	0.376979197	15.95432962	0.347776501
SLC22A4	0.951557241	1.702205561	0.532706345	5.439213928	0.369489389
SLC22A5	0.786982929	0.59452755	0.229034954	1.543271026	0.285330892
SLC22A6	0.711538654	1.817759246	0.098090158	33.68583294	0.688275169
SLC22A7	0.108841292	1.303560882	0.673105723	2.524523143	0.431799153
SLC22A8	0.346242863	0.005615441	3.31E-07	95.13425836	0.296912316
SLC22A9	0.476779361	1.525885244	0.656375591	3.547246136	0.32620547
SLC23A1	0.756882722	0.961451646	0.001611164	573.7401529	0.990381888
SLC23A2	0.92783185	1.094319384	0.663016812	1.806190872	0.724428453
SLC23A3	0.509862394	0.082659098	0.001988692	3.435688066	0.18987334
SLC24A1	0.250758807	1.015914431	0.54424213	1.896365744	0.960456127
SLC24A2	0.297312931	0.021947832	0.000435512	1.106071567	0.056190361
SLC24A3	0.078983588	2.708299968	0.925126773	7.928522803	0.069070283
SLC24A4	0.823413113	0.887918631	0.662118188	1.190723211	0.427189788
SLC24A5	0.24246828	0.379205075	0.037876187	3.796487937	0.409387188
SLC25A1	0.34686154	0.502900837	0.137774286	1.835678198	0.298111042
SLC25A10	0.65826396	1.04182013	0.690262363	1.572429909	0.845343372
SLC25A11	0.682938035	0.888266327	0.528437486	1.493113356	0.65477002
SLC25A12	0.327485747	0.513146068	0.14846595	1.773597838	0.29169775
SLC25A13	0.490372273	1.243297949	0.92080003	1.678746458	0.155201847
SLC25A14	0.113211263	1.25106758	0.617771549	2.533574252	0.53382789
SLC25A15	0.420566508	0.05221811	3.26E-05	83.52640923	0.432841198
SLC25A16	0.638387104	0.790884571	0.491399045	1.272892999	0.333940248
SLC25A17	0.427393615	1.260177009	0.3672157	4.324559368	0.713187343
SLC25A18	0.134032797	2.898619097	0.007862643	1068.59651	0.724129267
SLC25A19	0.46650483	1.009924179	0.399783975	2.551244946	0.983336626
SLC25A2	0.152171494	0.963803405	0.767539108	1.210253644	0.750978344
SLC25A20	0.722313175	0.991366751	0.652638942	1.505898548	0.967574615
SLC25A21	0.651449759	0.461644001	0.020632219	10.32924198	0.625937011
SLC25A22	0.630459527	2.694251646	0.854832849	8.491709156	0.090613502
SLC25A23	0.924398474	0.914595047	0.038571518	21.68657475	0.955925731

SLC25A24	0.901687144	1.116986153	0.399278725	3.124779731	0.833056474
SLC25A25	0.207675823	256.7944487	0.124359954	530262.2504	0.154247796
SLC25A26	0.311529668	0.246906731	1.22E-05	4985.972205	0.78212462
SLC25A27	0.774127318	0.377042601	0.04677137	3.039490247	0.35967404
SLC25A28	0.080137113	0.274962203	0.030195568	2.503818221	0.251962202
SLC25A29	0.20993314	0.616603837	0.156161157	2.434666199	0.490150611
SLC25A3	0.428705759	0.027780478	0.00021053	3.665767769	0.150294589
SLC25A30	0.666589718	1.498490898	0.000822	2731.720202	0.915915021
SLC25A31	0.477005383	0.727528367	0.248005883	2.13421358	0.562370425
SLC25A32	0.617320132	0.670757296	0.244464851	1.840409161	0.438063233
SLC25A34	0.124673686	0.389554339	0.078710227	1.927990661	0.24792432
SLC25A35	0.573247633	1.186697487	0.541521504	2.600544792	0.668920888
SLC25A36	0.607771395	1.092745941	0.36437983	3.277057597	0.874232453
SLC25A37	0.074537403	0.052041481	0.001881012	1.439818628	0.081022117
SLC25A4	0.2638408	2.467366213	0.000304326	20004.51719	0.844085491
SLC25A5	0.20935795	0.479743788	0.198750257	1.158006566	0.102327095
SLC25A6	0.127553243	0.875162464	0.731147803	1.047543786	0.146050426
SLC26A1	0.295067939	1.284753786	0.449693223	3.670485138	0.639909721
SLC26A10	0.481173497	1.25586455	0.391604056	4.02752664	0.701588346
SLC26A11	0.565381475	0.145127682	0.00100213	21.01728028	0.447057831
SLC26A2	0.491720911	0.34460825	0.080769034	1.47030168	0.150088129
SLC26A3	0.864317446	0.901074585	0.359591622	2.2579375	0.824118453
SLC26A4	0.203182431	0.986068985	0.811344735	1.198420353	0.887883841
SLC26A5	0.795504301	0.124256151	0.00021095	73.19064375	0.521652613
SLC26A6	0.568285766	0.895700977	0.537260584	1.493279546	0.672748968
SLC26A7	0.986611147	1.038041846	0.812513777	1.326169358	0.765143097
SLC26A8	0.478377953	0.8681342	0.534146276	1.410956182	0.568229743
SLC26A9	0.466823689	2.951236962	0.417078388	20.88288405	0.278352997
SLC27A1	0.691554176	1.18049127	0.3922011	3.553176265	0.767886881
SLC27A2	0.163389604	0.657254266	0.350490857	1.232509099	0.190776744
SLC27A3	0.260078336	0.577125212	0.199717833	1.667720434	0.309965065
SLC27A4	0.616178608	2.545966849	0.573710555	11.2982882	0.219015476
SLC27A5	0.285111691	1.567250823	0.427047584	5.751759833	0.49819461

SLC27A6	0.859509229	0.768816967	0.384961183	1.535426312	0.456311157
SLC28A1	0.235207323	2.676229977	0.186991519	38.30230868	0.468427122
SLC28A2	0.813129312	0.879582248	0.428926208	1.803725015	0.726210487
SLC28A3	0.205307414	1.186657526	0.525816793	2.67803559	0.680263051
SLC29A1	0.184064591	0.951417855	0.417367461	2.168822489	0.905702643
SLC29A2	0.861165237	1.415257579	0.348680318	5.744385081	0.627030316
SLC29A3	0.810831307	1.051149523	0.487688061	2.265618961	0.898692907
SLC29A4	0.490125238	1.153408572	0.379071872	3.50949631	0.801516353
SLC2A1	0.519005138	0.002835048	1.53E-06	5.269662681	0.126701143
SLC2A10	0.522513614	0.797521296	0.007195116	88.3988853	0.92496178
SLC2A11	0.710048365	1.043681	0.303324294	3.59110711	0.945935073
SLC2A12	0.875345938	0.18057031	0.000290219	112.3482384	0.602039157
SLC2A13	0.620084631	0.47198851	0.048308812	4.611439268	0.518538471
SLC2A14	0.125040413	2.228274546	0.970558959	5.115822596	0.058825907
SLC2A2	0.170401825	165.8962391	0.926385304	29708.54786	0.053473722
SLC2A3	0.566538939	0.760695857	0.485425077	1.192064881	0.232706662
SLC2A4	0.060546196	0.147278414	0.017418145	1.24530661	0.078653918
SLC2A4RG	0.903233586	0.020143059	0.000192148	2.111617185	0.099954764
SLC2A5	0.607334939	0.949277085	0.493096289	1.82748685	0.876218807
SLC2A6	0.090360905	1.472797423	0.953769915	2.274272039	0.080732609
SLC2A7	0.43321523	0.986649704	0.880613319	1.105454139	0.816778785
SLC2A8	0.076501472	0.503357474	0.183157831	1.38333559	0.183238208
SLC2A9	0.374741698	1.040507458	0.846948703	1.278301468	0.705336955
SLC30A1	0.700861239	0.00168799	6.66E-07	4.276583217	0.110363772
SLC30A10	0.082652663	1.277984232	0.538314197	3.03399707	0.57818558
SLC30A2	0.638902994	1.192493158	0.518191802	2.744234712	0.678879885
SLC30A3	0.825853224	0.770214707	0.003654602	162.3242983	0.923809487
SLC30A4	0.636770221	0.615711562	0.135645875	2.794782567	0.529769362
SLC30A5	0.260087503	0.504069163	0.10334627	2.458586271	0.396827332
SLC30A6	0.511363286	1.083710739	0.363648448	3.22957233	0.885268013
SLC30A7	0.856109051	0.874660885	0.226596168	3.37618977	0.845916691
SLC30A8	0.925163892	4.301795966	0.000123779	149504.376	0.784474616
SLC30A9	0.285225266	1.58621805	0.000192485	13071.58852	0.9201198

SLC31A1	0.881904888	0.865303072	0.388971717	1.92494563	0.722861124
SLC31A2	0.513334847	0.92463641	0.150221673	5.691272586	0.932653879
SLC32A1	0.584146911	0.729414944	0.340525675	1.562425978	0.416904373
SLC33A1	0.897015005	0.830335922	0.37491032	1.838993773	0.646744231
SLC34A1	0.19967041	0.531514441	0.214871841	1.314772563	0.171393637
SLC34A2	0.739990719	0.710396973	0.257259671	1.961690525	0.509388995
SLC34A3	0.946638936	1.316750869	0.368291505	4.707773139	0.672068686
SLC35A1	0.15091946	1.102889746	0.891122609	1.364981405	0.367969638
SLC35A2	0.878527033	2.719597174	1.83E-06	4047651.21	0.890268465
SLC35A3	0.426915688	1.383339275	0.791653497	2.417253962	0.254481402
SLC35A4	0.957574946	3.396456607	0.015008388	768.6313513	0.658483644
SLC35A5	0.818490799	1.835678272	0.280310284	12.02137386	0.526410402
SLC35B1	0.111395731	3285.231505	0.055980183	192795833.3	0.148351038
SLC35B2	0.424531094	2.179655157	0.169531866	28.02362002	0.549861419
SLC35B3	0.250371201	2038.822808	0.017172624	242059597.7	0.20118003
SLC35B4	0.504842509	1.35975771	0.360424513	5.129898113	0.650101001
SLC35C1	0.397915067	6.369237991	0.001356712	29901.09964	0.66775154
SLC35C2	0.317550667	0.032174104	5.42E-06	191.1550713	0.438264963
SLC35D1	0.538769158	0.372367856	0.057780772	2.399722523	0.298728958
SLC35D2	0.323721891	2.219143468	0.731956754	6.727990014	0.158960655
SLC35D3	0.721496699	1.551975218	0.449487982	5.358601729	0.486937179
SLC35E1	0.891086061	0.972199049	0.257008996	3.677579409	0.96686915
SLC35E2	0.528591602	0.458485137	0.007744618	27.14254235	0.708009491
SLC35E3	0.937058886	0.701875877	0.244687025	2.013305561	0.510270056
SLC35E4	0.575567741	0.849823974	0.24221004	2.981712841	0.799427676
SLC35F1	0.365324506	0.008728749	3.00E-05	2.537787901	0.101384316
SLC35F2	0.082844545	2.839277398	0.951716428	8.470481235	0.061313895
SLC35F3	0.6870285	0.846925439	0.323897626	2.214535211	0.734772931
SLC35F4	0.785640122	1.266515925	0.558431307	2.872443874	0.57173705
SLC36A1	0.503378461	0.083314134	0.000649902	10.68044408	0.315595807
SLC36A2	0.991550225	1.833650892	0.360996731	9.31386715	0.464656644
SLC36A3	0.821099776	0.936957326	0.441163492	1.989940347	0.865448407
SLC36A4	0.613130931	2.649184754	0.844033131	8.315052582	0.095035853

SLC37A1	0.224163688	0.515765477	0.02412437	11.02677618	0.671749644
SLC37A2	0.234407565	1.603758461	0.000181603	14162.95814	0.91884288
SLC37A3	0.511195549	0.677504847	0.252750693	1.816069473	0.438981511
SLC37A4	0.187876349	2.289889347	0.896450288	5.849285	0.083360968
SLC38A1	0.935482104	0.223631161	0.000893534	55.96978767	0.595034131
SLC38A2	0.073306934	0.725659811	0.39738183	1.325128934	0.296616329
SLC38A3	0.331733165	0.660579642	0.190172797	2.29457352	0.513979347
SLC38A4	0.906523791	0.807014541	0.111216522	5.855896769	0.832072387
SLC38A5	0.560314235	0.7934969	0.00580644	108.4377516	0.926545778
SLC38A6	0.496939216	1.17308302	0.16946063	8.120610503	0.871531027
SLC39A1	0.727718459	1.017552778	0.886049698	1.168572889	0.805334244
SLC39A10	0.112775494	0.296026271	0.059766543	1.466230915	0.135916413
SLC39A11	0.757588086	2.132139198	0.234113679	19.41799206	0.501744695
SLC39A12	0.068053279	5.965677856	0.022324632	1594.17242	0.531033571
SLC39A13	0.122406976	2.629933837	0.699129798	9.893087096	0.152580738
SLC39A14	0.215606388	2.844102007	0.52116255	15.52090846	0.227332342
SLC39A2	0.829971115	0.712797725	0.253794406	2.001937732	0.520505737
SLC39A3	0.643752486	0.579203828	0.170812566	1.964007002	0.380733948
SLC39A4	0.107428592	0.075814491	0.005289083	1.086735975	0.05759914
SLC39A5	0.605290459	0.648509828	0.214889103	1.957125753	0.44220796
SLC39A6	0.474648913	1.038658036	0.839545997	1.284992744	0.726859694
SLC39A7	0.483727201	0.736069646	0.206151053	2.628162785	0.636999102
SLC39A8	0.497781105	0.984646432	0.235959149	4.108883247	0.983064327
SLC39A9	0.523648487	1.000475009	0.762780396	1.312239079	0.997262208
SLC3A1	0.987534001	6.641837864	0.000286042	154222.317	0.712016168
SLC3A2	0.936637661	0.554752764	0.172876367	1.780177564	0.321927453
SLC40A1	0.613159088	0.034975252	4.67E-05	26.20209346	0.320756888
SLC41A1	0.321041	6.420080736	0.071065504	579.9921825	0.418385165
SLC41A2	0.826288409	0.741136064	0.344162459	1.595998201	0.444007965
SLC41A3	0.717208176	1.065199621	0.271239329	4.183206906	0.92789031
SLC43A1	0.613082893	1.501654172	0.93018094	2.424222163	0.096156101
SLC43A2	0.963038565	1.143383645	0.402012202	3.251956419	0.801623298
SLC43A3	0.742898352	0.483499757	0.035652313	6.556994374	0.584864712

SLC44A1	0.552115535	1.331456921	0.924171228	1.918234932	0.124374559
SLC44A2	0.909088831	1.08304589	0.466848295	2.512568671	0.852597167
SLC44A3	0.433452446	0.357014714	0.044794529	2.84542574	0.330775309
SLC44A4	0.51318283	187.0299445	0.254839602	137263.596	0.120213312
SLC44A5	0.412248535	1.944464005	0.227037741	16.65335573	0.543931036
SLC45A1	0.09019455	2.654929275	0.527827602	13.35407514	0.236142074
SLC45A2	0.40706571	0.254796301	0.004812118	13.4911823	0.499588533
SLC45A3	0.859813679	0.936808399	0.473670422	1.852786109	0.851187262
SLC45A4	0.00192743	0.305185519	0.11989515	0.776830428	0.012784233
SLC4A1	0.245716501	0.481313326	0.099356685	2.331624866	0.363692936
SLC4A10	0.486655934	0.591092972	0.204169932	1.711275001	0.332335578
SLC4A11	0.333292113	2.884393966	0.173584792	47.92890249	0.460051686
SLC4A1AP	0.124681824	0.812508776	0.615661021	1.072295448	0.14242076
SLC4A2	0.991114026	0.932682425	0.000487236	1785.369204	0.985579355
SLC4A3	0.299690567	1.286364915	0.583937798	2.833751647	0.53201628
SLC4A4	0.271837624	1.516631686	0.557471474	4.126079592	0.414713295
SLC4A5	0.367338893	1.238690728	0.622672254	2.464144996	0.541873222
SLC4A7	0.752972331	0.495233059	0.182206252	1.346033847	0.168366404
SLC4A8	0.264749445	0.908079563	0.478921899	1.721801603	0.767699233
SLC4A9	0.642289306	0.887052918	0.017815071	44.1683837	0.952067724
SLC5A1	0.963848573	0.82180533	0.593818664	1.137323632	0.236498655
SLC5A10	0.832670391	1.075466892	0.773891277	1.494562699	0.664781114
SLC5A11	0.815755846	1.39612224	0.372318539	5.235187361	0.620710328
SLC5A12	0.812069544	0.878846065	0.349362489	2.210799469	0.783788365
SLC5A2	0.785328021	2.372707212	0.456164259	12.34147439	0.304415989
SLC5A3	0.484847584	0.66000831	0.026345896	16.53430075	0.800394692
SLC5A4	0.823852761	0.95394887	0.485203809	1.875538546	0.891281854
SLC5A5	0.850541781	31.02639044	0.000695248	1384595.978	0.52946927
SLC5A6	0.737303624	0.64781252	0.27428573	1.530014194	0.32212325
SLC5A7	0.093392742	0.000683433	7.46E-08	6.258067983	0.117361006
SLC5A8	0.529645354	0.455072031	0.029718555	6.968392303	0.571731748
SLC5A9	0.067042635	33.99276787	0.553976845	2085.842176	0.093197085
SLC6A1	0.313467989	0.006216359	2.49E-05	1.552596397	0.071266673

SLC6A11	0.623229377	1.11089214	0.514760078	2.397391327	0.788733186
SLC6A12	0.148437598	2.06768239	0.914459401	4.675232669	0.08096051
SLC6A13	0.180904187	0.685902998	0.379926257	1.238300628	0.210993633
SLC6A14	0.081594997	0.88300777	0.740128787	1.053468985	0.167102741
SLC6A15	0.2326576	0.955958425	0.380782313	2.399944744	0.923596722
SLC6A16	0.622035601	1.887028226	0.000318508	11179.87259	0.886075494
SLC6A17	0.168197898	1.11598843	0.650836365	1.913584186	0.689986605
SLC6A18	0.184460947	1.34430533	0.813849283	2.220505512	0.24787536
SLC6A19	0.959988917	1.185762114	0.853679407	1.647025546	0.309474031
SLC6A2	0.700420248	0.138065008	0.000412339	46.22883688	0.504432187
SLC6A20	0.574102414	2.819371964	0.074407718	106.8284109	0.576212859
SLC6A3	0.61719843	1.908208212	0.500815192	7.270663195	0.343763016
SLC6A4	0.821128548	8.623609023	0.147216843	505.1503001	0.299530468
SLC6A5	0.935425222	1.021681191	0.292126628	3.573219126	0.973213665
SLC6A6	0.467559147	0.056284707	0.001954094	1.621195432	0.093314847
SLC6A7	0.829461305	0.800864182	0.352610229	1.818958687	0.595720353
SLC6A8	0.707529065	0.98915581	0.522190425	1.873701948	0.973313501
SLC6A9	0.608534028	1.440954782	0.808455909	2.568291802	0.21539361
SLC7A1	0.009278499	2.116380897	1.225514858	3.65484602	0.007155879
SLC7A10	0.969791891	0.180418876	0.004879144	6.671450671	0.35254351
SLC7A11	0.904633827	1.036171699	0.904332183	1.187231652	0.608834408
SLC7A13	0.811636616	1.001979998	0.725894152	1.383072055	0.990403492
SLC7A14	0.686040527	0.783830289	0.302127527	2.033545001	0.616556272
SLC7A2	0.972740999	0.914512222	0.296651646	2.819241412	0.876368216
SLC7A3	0.205247849	1.73111262	0.740688267	4.045900333	0.205175658
SLC7A4	0.882515546	1.677518444	0.470013524	5.987206716	0.425501552
SLC7A5	0.201026368	0.002710145	1.65E-06	4.44300621	0.117563865
SLC7A6	0.069965588	0.087173817	2.84E-05	268.0501685	0.551547164
SLC7A6OS	0.351282199	1.689428657	0.840600328	3.395393855	0.140909855
SLC7A7	0.240151511	3.043047692	0.832714144	11.12042989	0.092356592
SLC7A8	0.253935481	0.172188532	0.006491498	4.567341517	0.292891515
SLC7A9	0.80803958	1.74871927	0.272687274	11.21438137	0.555556167
SLC8A1	0.682836003	1.89643351	0.098068196	36.67305207	0.671956962

SLC8A2	0.980206584	2.303778104	0.578018293	9.182051185	0.236822201
SLC8A3	0.019711469	1.419669828	1.097146208	1.837004409	0.00769733
SLC9A1	0.406416372	2.24453841	0.675043347	7.463154329	0.187202272
SLC9A2	0.685082436	0.728352692	0.376150263	1.410334368	0.347139433
SLC9A3	0.827363555	0.845439424	0.572106301	1.249361909	0.399432847
SLC9A3R1	0.565993278	0.964066823	0.749161858	1.240619538	0.776113052
SLC9A3R2	0.63440252	15.02128728	0.207295259	1088.49123	0.2150236
SLC9A4	0.430607482	1.0218725	0.785456121	1.32944843	0.87196261
SLC9A5	0.442159967	0.660920243	0.132851121	3.28800814	0.612928427
SLC9A6	0.563253637	1.381921246	0.382972451	4.986537088	0.621270431
SLC9A7	0.216819867	0.465947655	0.117392503	1.849412967	0.277580548
SLC9A8	0.820542119	0.83924672	0.617975814	1.139745342	0.261736589
SLC9A9	0.163274295	0.089835739	0.000291255	27.70926119	0.409911909
SLCO1A2	0.94659698	1.330744872	0.613180065	2.888029172	0.469813276
SLCO1B1	0.718619753	0.845469555	0.235145233	3.039903296	0.797102982
SLCO1B3	0.064725068	0.345887574	0.091525494	1.307157261	0.117562193
SLCO1C1	0.867748439	1.101315882	0.027371784	44.31193364	0.959171193
SLCO2A1	0.538647343	1.077186273	0.558945549	2.075927196	0.824214719
SLCO2B1	0.168195471	1.735141971	0.842877239	3.571952733	0.134665075
SLCO3A1	0.321925055	9.313178893	0.215513706	402.4583992	0.245532839
SLCO4A1	0.965995765	0.972731052	0.497742036	1.900996162	0.935541287
SLCO4C1	0.250310008	1.982442254	0.958613365	4.099752242	0.064899558
SLCO5A1	0.148930105	2.278714376	0.903035077	5.750096914	0.081160395
SLCO6A1	0.606383022	1.448129402	0.551327793	3.803687738	0.452352595
SLFN5	0.968649825	0.600469496	0.147928489	2.437418371	0.475507499
SLIT1	0.927469368	0.606585836	0.000758889	484.8484526	0.883451695
SLIT2	0.848369043	0.895975759	0.399252263	2.01069007	0.789979002
SLIT3	0.861150797	0.949207659	0.731537577	1.231645794	0.694886275
SLITRK1	0.556591206	0.491957223	0.025413993	9.52317524	0.638916013
SLITRK2	0.751654854	1.149559614	0.342821964	3.854733484	0.821371717
SLITRK3	0.779070984	0.965388101	0.464294932	2.007289164	0.924858558
SLITRK4	0.132791826	1.553977374	0.538326317	4.48583991	0.415072994
SLITRK5	0.907062619	1.204127024	0.453176362	3.199464963	0.709478646

SLITRK6	0.192508906	3.721310452	0.940389384	14.72597599	0.061152725
SLK	0.977326254	1.149383708	0.210587376	6.27332431	0.872256872
SLMAP	0.417379143	0.097311971	8.55E-06	1107.958894	0.624911702
SLN	0.090360905	1.633889351	0.803867865	3.320936843	0.174883178
SLPI	0.58736523	0.761155918	0.464005089	1.248603402	0.279809888
SLTM	0.53509068	0.924172915	0.275906459	3.095598343	0.898264249
SLU7	0.763526913	0.018406141	3.84E-06	88.25554366	0.355547071
SLURP1	0.473103817	1.586000559	0.702083695	3.582760561	0.267312938
SMAD1	0.606054104	0.799966838	0.464956937	1.376357446	0.420159511
SMAD2	0.337940496	1.365640219	0.675401507	2.761280793	0.385676908
SMAD3	0.620711695	0.873026918	0.039530754	19.28058324	0.931471294
SMAD4	0.051631021	2.745857932	0.874959059	8.61724409	0.083443778
SMAD5	0.528632252	1.078167727	0.893439985	1.301089795	0.432514019
SMAD6	0.520982536	0.330692185	0.003876971	28.20689438	0.625690235
SMAD7	0.870129139	2.548549914	0.00012004	54107.97846	0.853984848
SMAD9	0.042806027	1.624726378	1.116978468	2.363282622	0.011129406
SMAP1	0.337333742	1.135793425	0.539566378	2.390858208	0.737404602
SMARCA1	0.193380502	0.684821573	0.343918031	1.363640589	0.281319904
SMARCA2	0.083510041	0.43947449	0.179201209	1.077770781	0.072442038
SMARCA4	0.288060743	0.336954788	0.094393741	1.202818405	0.09383133
SMARCA5	0.203195264	0.620844358	0.232368797	1.658775713	0.341776997
SMARCAD1	0.671446915	0.254503111	0.000216267	299.4993013	0.704440294
SMARCAL1	0.131585151	0.193832487	0.028288486	1.328138725	0.094729177
SMARCB1	0.675118926	0.782992289	0.460223781	1.332127868	0.366917997
SMARCC1	0.668961098	0.731239631	0.302706916	1.766432707	0.48668386
SMARCC2	0.382315653	0.963431226	0.76043306	1.220619901	0.757632056
SMARCD1	0.981137437	0.04678444	3.72E-07	5887.479198	0.609276749
SMARCD2	0.824460869	3.186735365	0.337673129	30.0742979	0.31154181
SMARCD3	0.782307175	0.12653119	0.002885973	5.547572449	0.283846489
SMARCE1	0.727518824	1.274393414	0.224705469	7.227588091	0.784206901
SMCHD1	0.223342471	0.736198944	0.019324783	28.04631188	0.869023971
SMCP	0.944306356	0.791528213	0.099576218	6.291832766	0.825063443
SMCR8	0.405284605	0.728401281	0.32465997	1.634228036	0.4421063

SMEK2	0.123167089	1.370241042	0.85578443	2.19396433	0.189682627
SMG1	0.615628514	1.111449272	0.908433756	1.359834415	0.304524931
SMG5	0.364853919	0.171137816	0.005701364	5.13704341	0.309111003
SMG6	0.136247819	1.329595227	0.684783705	2.581579345	0.400079648
SMG7	0.370231176	0.321972827	5.88E-05	1762.789956	0.796375071
SMN1	0.529471395	0.253266497	0.045719411	1.402990933	0.115881908
SMN2	0.439163574	0.05662663	4.81E-06	667.1490868	0.548291291
SMNDC1	0.244150256	0.71236476	0.329469242	1.540245602	0.388646653
SMO	0.89241796	0.929503517	0.74129267	1.165500244	0.52655156
SMOC1	0.800320203	1.470657511	0.567649247	3.810158342	0.427122211
SMOC2	0.68710492	0.885093464	0.468072638	1.67365143	0.707268231
SMOX	0.881388519	0.771718747	0.170129819	3.500561092	0.736948732
SMPD1	0.330468184	3.352208681	0.221723097	50.68169809	0.382705189
SMPD2	0.574798859	0.651218128	0.00979332	43.30350207	0.841253153
SMPD3	0.288648987	0.819775229	0.244519084	2.748380271	0.747478525
SMPDL3A	0.929276118	0.867715288	0.111239314	6.768558658	0.892308504
SMPDL3B	0.940801338	5.182084414	0.064811076	414.3427432	0.461762173
SMPX	0.101254405	1.090773812	0.933130834	1.275048972	0.275289701
SMR3A	0.239367241	2.039964933	0.897822881	4.635053323	0.088649168
SMR3B	0.917977579	0.796406805	0.357251173	1.775400186	0.57782954
SMS	0.291297867	1.923965864	0.494647764	7.483395078	0.345039935
SMTN	0.328210126	1.943541705	0.775567419	4.870439717	0.15627273
SMU1	0.79917635	1.405279422	0.744229958	2.653494708	0.294132632
SMUG1	0.233989563	1.045316397	0.587642128	1.859441857	0.880119015
SMURF1	0.833584467	0.993252316	0.283721869	3.477173504	0.991550006
SMURF2	0.37258903	0.571212332	0.000226609	1439.85314	0.88855428
SMYD1	0.118591702	0.829067357	0.632487838	1.086744505	0.174612435
SMYD2	0.805176042	1.183553812	0.468652527	2.988994076	0.721442509
SMYD3	0.811350082	0.945821915	0.413225379	2.164869679	0.895110531
SMYD5	0.582323108	0.820869283	0.359508917	1.874296708	0.639362327
SNAI1	0.871291957	1.352609392	0.595937724	3.070039186	0.470153314
SNAI2	0.824948173	0.004154788	3.87E-08	445.5249962	0.353467533
SNAI3	0.237070954	1.757962972	0.567641851	5.44433749	0.327998274

SNAP23	0.869504052	0.001459206	6.37E-08	33.44448881	0.202393345
SNAP25	0.40134241	0.8081799	0.420112876	1.554712526	0.52347764
SNAP29	0.912594563	0.951154991	0.284499681	3.179953712	0.935185616
SNAP91	0.515985476	1.991483528	0.55997783	7.082435111	0.28724211
SNAPC1	0.817488605	0.848775064	0.357361057	2.01594185	0.710271512
SNAPC2	0.854589384	0.943810591	0.800982714	1.112106936	0.489715828
SNAPC3	0.416891802	2.619660783	0.612143914	11.21079938	0.194179023
SNAPC4	0.35467661	0.548894204	0.21748052	1.385341761	0.204114615
SNAPC5	0.221907988	5.398931122	0.256713848	113.5445458	0.277923187
SNCA	0.215618798	0.828124047	0.386630823	1.773757797	0.627477797
SNCAIP	0.477342832	1.387550224	0.819387662	2.349676124	0.222935833
SNCB	0.295857556	1.29987823	0.349510209	4.834432208	0.695535513
SNCG	0.903710287	0.880510168	0.436492704	1.776199575	0.722270634
SND1	0.189793095	0.081373308	0.000772284	8.574070149	0.291094821
SNED1	0.170301987	1.083158593	0.597256486	1.96436969	0.792546201
SNF8	0.687285278	0.377789498	0.072433615	1.970423601	0.248043456
SNIP1	0.076990453	0.595072107	0.249327444	1.420264081	0.24220336
SNN	0.731998623	0.701899813	0.099966033	4.928307476	0.721868219
SNPH	0.603403817	1.176970008	0.601041135	2.304764716	0.634631765
SNRK	0.491745004	2.628444706	0.207679198	33.26631478	0.455517022
SNRPA	0.704666162	0.041056842	2.53E-05	66.5201059	0.397132188
SNRPA1	0.727926503	1.450110133	0.484037534	4.344331278	0.506784941
SNRPB	0.382100704	0.701336647	0.45407046	1.08325279	0.109724137
SNRPB2	0.162497022	0.143365075	4.91E-07	41889.23991	0.762273898
SNRPC	0.778692569	0.224224047	0.002574548	19.52825082	0.511820181
SNRPD1	0.621730516	0.092439113	0.004450386	1.920055787	0.123930823
SNRPD2	0.844329985	3.349123595	0.413821295	27.10500639	0.257238246
SNRPD3	0.262094685	0.89019125	0.779018074	1.01722988	0.087455392
SNRPE	0.613143074	0.585658711	0.126644395	2.708340369	0.493492866
SNRPF	0.401670988	3.061280424	0.582011286	16.10181463	0.186524962
SNRPG	0.577368042	0.605644223	0.144373416	2.540668046	0.493064879
SNRPN	0.398997698	1.175128001	0.144974783	9.525282885	0.879857043
SNTA1	0.533436513	0.099502033	0.001205401	8.213576929	0.305462803

SNTB1	0.203779518	0.475410076	0.146966809	1.537862475	0.214452482
SNTB2	0.493036717	1.039837213	0.280083574	3.860495685	0.953454753
SNTG1	0.846584745	0.530077308	0.135589878	2.07229299	0.361520619
SNTG2	0.743551103	0.029379354	0.000165986	5.200114628	0.181652062
SNURF	0.516611319	0.63654835	0.137054423	2.95644454	0.564283912
SNW1	0.07505503	60.08757644	0.061827399	58396.7123	0.243234648
SNX1	0.682375482	0.704161217	0.153588811	3.228379829	0.651656912
SNX10	0.317565273	0.652237033	0.218624038	1.9458663	0.443510054
SNX11	0.877180503	0.988300525	0.609947106	1.601348571	0.961880976
SNX12	0.655977872	302.1719152	0.032212422	2834554.548	0.221028373
SNX13	0.863395121	1.206606954	0.848680391	1.715487193	0.295516851
SNX14	0.518544683	0.50129077	0.098473961	2.551866841	0.405582954
SNX15	0.879779044	11.64173561	0.017781555	7621.943396	0.458119877
SNX16	0.452235146	1.013915071	0.772402453	1.330943171	0.920699877
SNX17	0.774008315	0.896333114	0.418379515	1.920297294	0.778303505
SNX19	0.053387998	0.855707499	0.639854727	1.144377455	0.293413223
SNX2	0.611043318	1.034427726	0.658198183	1.625712056	0.883335925
SNX22	0.30018512	0.451300383	0.099135074	2.054490185	0.303545716
SNX24	0.364477083	258.0909827	0.182431438	365128.708	0.133534001
SNX25	0.933330364	0.85626866	0.193317413	3.79270552	0.838076993
SNX27	0.572694021	1.109164206	0.263849716	4.662674094	0.887544261
SNX3	0.586521636	0.730043525	0.388130473	1.373155641	0.328982447
SNX30	0.542574995	1.462164457	0.622422627	3.434844437	0.383278403
SNX4	0.741649332	1.263049464	0.587727387	2.714343391	0.549642973
SNX5	0.16654629	0.011680781	7.34E-05	1.859683561	0.085407516
SNX6	0.061165324	51.65040769	0.106853927	24966.46304	0.210999114
SNX7	0.391685844	2.216800191	0.366927503	13.39284475	0.385691119
SNX8	0.387078952	2.240853945	0.764053048	6.572091321	0.1416304
SNX9	0.431837547	0.263417219	0.046239506	1.500635235	0.132906212
SOAT1	0.957659183	1.229281943	0.327888222	4.608686723	0.759481993
SOAT2	0.12254728	0.466929233	0.15294259	1.42552123	0.181100207
SOCS1	0.215739201	0.462333113	0.121212604	1.763446217	0.25870388
SOCS2	0.303179492	0.000191143	3.99E-12	9149.001313	0.3426161

SOCS3	0.803142049	1.041317121	0.544381792	1.991876586	0.902625806
SOCS4	0.597488667	0.990147895	0.825712029	1.187330231	0.914905008
SOCS5	0.504730566	0.287002108	0.006883529	11.96627615	0.511920556
SOCS6	0.37753246	0.734037476	0.317671565	1.696126045	0.469336257
SOCS7	0.878031022	2.027017828	0.265195887	15.49345778	0.495936855
SOD1	0.441929251	0.778658815	0.357346901	1.696697377	0.528977909
SOD2	0.976032536	0.990454365	0.798464421	1.228608092	0.930476796
SOD3	0.3257743	0.005289578	5.84E-07	47.89232166	0.259459571
SON	0.905110826	1.121531701	0.750875386	1.67515593	0.57527438
SORBS1	0.476122158	1.402849286	0.717363763	2.743358697	0.322547751
SORBS2	0.804504948	5.345163254	0.007353494	3885.332763	0.618048961
SORBS3	0.893010943	0.86888159	0.182249646	4.142423498	0.859998246
SORCS1	0.641171532	0.840467639	0.38478603	1.835788718	0.662835473
SORCS2	0.072009125	3.959842611	0.772620333	20.29503087	0.098826923
SORCS3	0.731465183	1.630548459	0.552921298	4.808438902	0.375573405
SORD	0.255918226	0.816665532	0.019363943	34.44250008	0.915516537
SORL1	0.224520126	1.556538738	0.726653842	3.334204958	0.254944675
SORT1	0.594082713	1.176933922	0.338969841	4.086420941	0.797550595
SOS1	0.51394077	1.604635142	0.946690996	2.719846233	0.079005703
SOS2	0.528519285	0.964617522	0.20975023	4.436166598	0.963092152
SOST	0.414350456	0.364169323	0.011258727	11.77924468	0.569021141
SOSTDC1	0.122287533	0.416092545	0.045415032	3.812240075	0.437829067
SOX1	0.190029959	0.860755111	0.65335799	1.133986839	0.286411786
SOX11	0.125283112	0.722073951	0.229308982	2.273747792	0.577939835
SOX12	0.256478967	2.786240319	0.897689926	8.647902678	0.076196553
SOX13	0.345930758	0.937946421	0.763475558	1.152287692	0.541813681
SOX14	0.369495376	0.400976645	0.018917301	8.499218065	0.557528982
SOX15	0.443221733	757.7065108	0.102293721	5612457.469	0.14471482
SOX17	0.247686077	0.766762785	0.358662279	1.639216616	0.493292022
SOX18	0.495700273	0.636830929	0.126762955	3.199307185	0.583750486
SOX2	0.853673043	0.809267666	0.215207709	3.04317238	0.754164909
SOX21	0.725134643	0.72026598	0.204400441	2.538072216	0.609624869
SOX3	0.598078694	0.212578521	6.18E-06	7312.566497	0.771405106

SOX30	0.362378092	0.897460263	0.692411404	1.163231743	0.413661746
SOX4	0.748729447	1.440950009	0.802279382	2.588047227	0.221465194
SOX5	0.647100466	0.393017828	0.022888586	6.748473323	0.51971697
SOX6	0.477210721	0.93555782	0.809631101	1.081070666	0.366466202
SOX7	0.128594958	0.874906623	0.644221968	1.188195431	0.392130266
SOX8	0.847599868	0.822536292	0.470832332	1.43695729	0.492497378
SOX9	0.298754188	1.074718982	0.875848251	1.318745444	0.490057346
SP1	0.444740276	1.211158013	0.911270572	1.609734561	0.186889026
SP100	0.678602077	0.870584435	0.118450198	6.398615397	0.891679782
SP110	0.69348977	64.50656256	0.000944262	4406720.376	0.463172288
SP140	0.140304174	2.371840186	0.872568224	6.447204604	0.090495941
SP2	0.379109976	0.860114586	0.006389358	115.7858184	0.951960619
SP3	0.602920625	0.075704487	0.000474997	12.06569871	0.318531462
SP4	0.245119917	0.013243376	5.61E-06	31.28574384	0.275208651
SP5	0.373013844	0.074088808	0.000294971	18.60914551	0.355992365
SP6	0.760413924	5.919590388	0.024134907	1451.903288	0.526455517
SP7	0.883416477	1.071838743	0.520395971	2.207623338	0.850729063
SP8	0.697511411	0.928848375	0.365030582	2.363526088	0.876905722
SPA17	0.680548436	0.872662233	0.200295162	3.80208571	0.856062363
SPACA1	0.42030654	0.559545875	0.198797878	1.574924184	0.271461471
SPACA3	0.071117903	1.097857082	0.782088634	1.541117105	0.589516336
SPACA4	0.84810184	1.522108947	0.441892604	5.242938271	0.505578715
SPAG1	0.962325524	41.94258418	0.009808103	179359.8991	0.381100666
SPAG16	0.213546999	1.00432481	0.79765885	1.264535989	0.970714208
SPAG17	0.432439865	2.413234965	0.000200168	29094.10628	0.854217279
SPAG4	0.54097197	1.155407622	0.438807297	3.04226202	0.769951938
SPAG5	0.568056402	0.567637961	0.118724174	2.713961649	0.478121077
SPAG6	0.421558892	0.985035853	0.689855027	1.406521072	0.933881606
SPAG7	0.4378762	0.006466111	6.94E-06	6.026702569	0.148436634
SPAG8	0.904596836	1.070895701	0.336346722	3.409629192	0.907715517
SPAG9	0.133026207	1.450585746	0.891753696	2.359619048	0.134017556
SPAM1	0.53713537	4556.273692	0.016767351	1238098357	0.186978116
SPANXA1	0.506042979	0.073320171	4.31E-05	124.6323533	0.491140759

SPANXC	0.813188308	1.1419951	0.007323973	178.0663098	0.958896368
SPANXD	0.731148055	2.568453216	0.372543562	17.70786723	0.338264937
SPARC	0.119578267	1.251789966	0.92000747	1.703223258	0.152910684
SPARCL1	0.605017755	3.984691814	0.543058489	29.23767732	0.173973796
SPAST	0.965515357	0.847328216	0.396182952	1.812205959	0.669292023
SPATA1	0.816368741	0.307046927	0.068689454	1.372522418	0.122225794
SPATA12	0.720762104	1.032191089	0.78774671	1.352488599	0.818267847
SPATA13	0.126376575	1.242103206	0.003900286	395.5659353	0.941226828
SPATA16	0.770669726	0.934882717	0.486417518	1.796821992	0.839922539
SPATA18	0.534151544	1.008276494	0.694680198	1.463438125	0.965411849
SPATA19	0.479996783	0.500931938	0.126953572	1.976571454	0.323610704
SPATA2	0.06505312	29.22115483	0.650558124	1312.528209	0.082123062
SPATA20	0.47999745	0.491230362	0.063951549	3.77328262	0.494379663
SPATA21	0.212651493	0.065355218	2.11E-05	202.2774668	0.50591962
SPATA22	0.471975324	0.940020096	0.735182356	1.201930071	0.621837811
SPATA3	0.306663124	0.689074335	0.306941369	1.546951591	0.366753438
SPATA4	0.32691493	0.538973648	0.117433123	2.473685336	0.426608829
SPATA5	0.27192121	0.689151991	0.140939543	3.369746031	0.64569643
SPATA5L1	0.615790242	0.93896635	0.790287902	1.115615974	0.473976199
SPATA6	0.8050763	1.141180731	0.41619981	3.129010222	0.79747289
SPATA7	0.835654361	0.096622212	0.002732535	3.41655301	0.198933204
SPATA8	0.247967012	1.111156205	0.947021643	1.303738011	0.196191935
SPATA9	0.088833227	0.00325845	7.40E-06	1.435413717	0.065241844
SPATC1	0.580975941	1.360524065	0.908088618	2.038375657	0.135554343
SPATS1	0.412055182	0.063356962	0.00063059	6.365636199	0.240788145
SPATS2	0.877139138	0.981367768	0.030432487	31.64653284	0.991532344
SPCS1	0.389920175	0.744131097	0.243960521	2.269756953	0.603479676
SPCS2	0.730601191	1.198041136	0.428363128	3.350667858	0.73059232
SPCS3	0.172789493	1.594896477	0.984872031	2.582766786	0.057697981
SPDEF	0.168869576	0.943315141	0.485902579	1.831320707	0.863117619
SPECC1	0.143469222	0.742703951	0.128772962	4.283579006	0.73934608
SPEN	0.095610217	0.654636747	0.098927004	4.331974606	0.660351061
SPESP1	0.969119046	0.63353861	0.265048018	1.514333791	0.304605925

SPG20	0.675007448	1.35855203	0.528920733	3.489490018	0.524355526
SPG21	0.430762344	0.113251916	0.008581312	1.494642881	0.097992843
SPG7	0.712942689	0.139189895	0.001955173	9.909010481	0.364877797
SPHK1	0.488491261	0.688331447	0.242974458	1.949999948	0.482071959
SPHK2	0.320813171	0.465333207	0.126443157	1.71250859	0.249837221
SPI1	0.963842224	0.234152831	0.005412774	10.12928871	0.45005889
SPIB	0.09019455	1.250747647	0.802577301	1.949182559	0.32295323
SPIC	0.814846884	0.785797383	0.117314736	5.263426823	0.803806644
SPIN1	0.428771865	0.047596091	0.000392994	5.764433286	0.213423586
SPIN3	0.171446295	0.087511412	5.08E-06	1508.923561	0.624538184
SPINK1	0.199683292	0.106862103	0.00019745	57.83492836	0.486188873
SPINK2	0.9552185	0.575660536	0.200287138	1.654549847	0.305271741
SPINK4	0.805652474	1.550647913	0.201035454	11.9606214	0.67386147
SPINK5	0.394151042	0.862080298	0.523742916	1.418983279	0.559440154
SPINK6	0.699221529	1.793754951	0.071407378	45.05916468	0.722397254
SPINK7	0.671858625	0.756383919	0.569150767	1.005211038	0.054336356
SPINT1	0.655944595	1.309680596	0.613146694	2.797476168	0.485977393
SPINT2	0.693069808	0.790427719	0.201688371	3.097729319	0.735755522
SPINT4	0.355082307	1.152435333	0.353632594	3.755613083	0.813912025
SPIRE1	0.168527363	0.562399306	0.239529957	1.320473577	0.186295972
SPIRE2	0.453246107	0.353596238	0.024403733	5.123408749	0.44596356
SPN	0.951214524	0.011017573	3.29E-07	368.5072039	0.396341508
SPO11	0.948223407	0.670068559	0.010015582	44.82933508	0.851900234
SPOCD1	0.472988854	0.262199945	0.006970075	9.863424859	0.469505003
SPOCK2	0.902756349	1.355320196	0.214821903	8.550770698	0.746307858
SPON1	0.697021839	0.522557844	0.107126942	2.549001174	0.422148394
SPON2	0.463122929	102.5385542	0.031065825	338447.6416	0.262661731
SPOP	0.874851653	0.768106272	0.265086169	2.225643262	0.626934233
SPP1	0.76667167	46.63158001	0.027778156	78281.0874	0.310519642
SPP2	0.852955133	0.653728028	0.096092215	4.447398123	0.663922774
SPPL2A	0.64611117	1.012148736	0.149619447	6.847004765	0.990122308
SPPL2B	0.190559285	1.443920127	0.483555049	4.311619401	0.510423339
SPPL3	0.683647055	8.165678632	0.001688454	39490.74913	0.627582391

SPR	0.557774801	0.638044561	0.024970025	16.30358224	0.785805874
SPRED1	0.82907378	0.980119433	0.763525559	1.258155792	0.874770035
SPRED2	0.153704342	0.49231529	0.175552625	1.380636399	0.178011219
SPRED3	0.374463888	0.095972524	0.004264487	2.15986733	0.140144532
SPRN	0.895740691	0.724918855	0.129402378	4.061033144	0.714432183
SPRR1A	0.968607487	0.491076815	0.147641888	1.633387662	0.246137917
SPRR1B	0.443002433	0.060378584	0.000315663	11.54892283	0.294992648
SPRR2A	0.942154355	0.562543073	0.116641671	2.713050212	0.473592563
SPRR2B	0.727685114	0.945324478	0.625255745	1.429236558	0.789778611
SPRR2D	0.70913714	0.801766953	0.439998422	1.460983075	0.47050422
SPRR2E	0.682575429	0.142390671	0.004095931	4.950059361	0.281668761
SPRR2F	0.492326563	10.00931419	0.499040507	200.7579928	0.132157866
SPRR2G	0.100435039	0.718322677	0.400805672	1.287375664	0.266403739
SPRR3	0.690899481	0.931059463	0.206270974	4.202587045	0.925987325
SPRR4	0.732522425	0.855213328	0.294244543	2.485653019	0.773871806
SPRY1	0.063043582	0.219358824	0.037946615	1.268052316	0.090137014
SPRY2	0.382826397	1.020463611	0.3212715	3.241326982	0.972595365
SPRY3	0.299255324	0.401956538	0.144614528	1.117239467	0.080565448
SPRY4	0.381471249	0.034540014	4.03E-05	29.61494967	0.328719376
SPRYD3	0.993040367	0.765297176	0.379761765	1.542229422	0.454344648
SPSB1	0.179707607	0.631107593	0.321291187	1.239675441	0.181472599
SPSB2	0.377720238	0.585002722	0.194407636	1.760363899	0.340161898
SPSB3	0.272329642	0.681437697	0.003230573	143.7383989	0.888286663
SPSB4	0.31686598	1.18040918	0.408482431	3.411078974	0.759343055
SPTA1	0.290367672	0.001807011	8.68E-08	37.62388151	0.213155407
SPTAN1	0.462138128	0.284677746	0.003968879	20.41922191	0.564406669
SPTB	0.097665354	1.361848135	0.643259227	2.88317721	0.419643385
SPTBN1	0.162658265	0.595544534	0.20425737	1.736403892	0.342483797
SPTBN2	0.9336446	0.821708456	0.543545605	1.242222879	0.351701986
SPTBN4	0.968431526	0.033249018	1.56E-05	70.69939342	0.38393655
SPTBN5	0.14061017	1.201515119	0.795899825	1.813844576	0.382321261
SPTLC1	0.165978367	10.17553135	0.728827274	142.0658117	0.084564139
SPTLC2	0.365810826	1.942375541	0.330035431	11.43156882	0.462861986

SPTY2D1	0.525797828	1.170289024	0.427543719	3.203359885	0.759544714
SPZ1	0.792179445	1.369081619	4.23E-06	442668.3328	0.961291929
SQLE	0.22146254	1.562465342	0.758369101	3.219142161	0.226271099
SQRDL	0.104607996	0.363512172	0.0028182	46.88848123	0.683181929
SQSTM1	0.1217037	0.763127385	0.459017034	1.268718504	0.297275039
SRA1	0.66851909	0.452819592	1.41E-06	145046.3399	0.902511611
SRC	0.952988038	1.454170544	0.399157076	5.29769381	0.570271544
SRCAP	0.816557599	1.173815389	0.829330795	1.661390818	0.365908227
SRD5A1	0.776172852	0.579033728	0.258836861	1.295333508	0.183498963
SRD5A2	0.922115732	0.333794295	0.031627016	3.522894251	0.361457865
SREBF1	0.095946064	0.000186216	1.33E-09	26.12779657	0.155507537
SREBF2	0.446326505	0.803041848	0.379873874	1.697606113	0.565753307
SRF	0.732067594	1.055292078	0.601169214	1.852459081	0.851303537
SRFBP1	0.332677108	0.757208117	0.287325043	1.995524398	0.573758836
SRGAP1	0.405394215	0.921287239	0.745458527	1.138588059	0.447995744
SRGAP2	0.313877713	1.284045107	0.540294511	3.051616854	0.571346959
SRGAP3	0.689438651	1.908969112	0.041562468	87.6791796	0.740552944
SRI	0.635588453	0.776866338	0.194880723	3.096875343	0.720454741
SRL	0.796467863	0.945499679	0.719979195	1.241660383	0.686878557
SRM	0.9844749	21.73691562	0.020708505	22816.39812	0.385650054
SRMS	0.570043637	0.69413616	0.068522634	7.031618333	0.757299547
SRP14	0.464076162	1.088472633	0.475628932	2.490960061	0.840934318
SRP19	0.114531547	0.616715631	0.311031303	1.222829234	0.166369558
SRP54	0.873035099	0.713862098	0.161500127	3.15540988	0.656667774
SRP68	0.199976719	0.009048127	1.34E-08	6120.1431	0.492112781
SRP72	0.609071564	6.690161946	0.014676041	3049.750796	0.542873512
SRP9	0.932296903	2.161987033	0.204842459	22.81845253	0.521345347
SRPK1	0.139811401	0.033029772	4.69E-06	232.4251606	0.45054197
SRPK2	0.711928547	0.796905277	0.318898754	1.991409541	0.627090299
SRPR	0.356749866	0.897811816	0.246587923	3.268878892	0.870129426
SRPRB	0.086907423	1.928763087	0.994227438	3.741726392	0.052035361
SRPX	0.942128815	3.230529601	0.643770151	16.2112541	0.154204848
SRPX2	0.544082386	0.713830231	0.416545764	1.223283591	0.219961077

SRR	0.662504656	0.973812622	0.391494396	2.422285047	0.954484686
SRRM1	0.896619601	12.75926707	2.81E-05	5799519.307	0.701650211
SRRM2	0.704980544	0.661221291	0.190377106	2.296566033	0.514930525
SRXN1	0.637715882	0.882073849	0.538399529	1.445124362	0.618362396
SRY	0.287056898	109.2023484	0.026404049	451641.0674	0.269332053
SS18	0.851304469	0.973205644	0.656947198	1.441712863	0.892252815
SS18L1	0.655190528	0.925607941	0.676254321	1.266905117	0.62930092
SS18L2	0.88164647	1.748536593	0.247776846	12.33924909	0.575150033
SSB	0.970563782	1.037879527	0.367563262	2.930635412	0.94403407
SSBP1	0.547053664	0.835271634	0.260975498	2.673349449	0.761692655
SSBP2	0.297993671	0.929558629	0.237087318	3.644561223	0.916546091
SSBP3	0.993122175	1.126048445	0.579075744	2.189670547	0.726436361
SSBP4	0.548149256	0.676477342	0.14137891	3.236844841	0.624589978
SSFA2	0.229023244	2.132247763	0.852511107	5.3330455	0.105487706
SSH1	0.805994	0.933284869	0.513156283	1.697378899	0.821009066
SSH2	0.381847886	1.160652025	0.882993042	1.525621447	0.285542316
SSH3	0.962199824	1.071090848	0.465399864	2.465053589	0.871709442
SSNA1	0.884488935	1.230842964	0.891867138	1.698654809	0.206339856
SSPN	0.867548121	0.664594912	0.206944476	2.1343232	0.492484783
SSR1	0.971612278	0.709374673	0.438081541	1.148672974	0.162618751
SSR2	0.303478275	1.305817518	0.056411197	30.22732166	0.867801892
SSR3	0.592993799	0.609352688	0.097256939	3.817832475	0.596749765
SSR4	0.135860727	0.040622988	0.000696826	2.368206865	0.122506459
SSRP1	0.449482479	2.005211523	0.285319281	14.09253956	0.484338357
SSSCA1	0.777097723	0.620101533	0.244038187	1.575679263	0.315211557
SST	0.753104779	0.272147134	0.020519905	3.609376566	0.323760555
SSTR1	0.702322996	0.966486368	0.656993886	1.42177259	0.862580724
SSTR2	0.912739726	1.059400005	0.499702348	2.245993792	0.880367301
SSTR3	0.149227523	0.674975678	0.414744634	1.09848839	0.113666295
SSTR4	0.337838218	0.529957122	0.202782086	1.385006721	0.195163345
SSTR5	0.662930992	1.045351557	0.872960359	1.251786368	0.629547751
SSU72	0.653661716	0.983056193	0.28036064	3.446986996	0.978701381
SSX1	0.401271543	0.800826212	0.125982603	5.090564947	0.813915971

SSX2	0.744076117	0.683168749	0.333006632	1.401532265	0.298695752
SSX2IP	0.237455153	3.60723266	0.330905785	39.32275603	0.292524105
SSX3	0.846942858	0.011177252	5.17E-09	24177.16387	0.545968476
SSX4	0.265753911	0.500849789	0.162287163	1.545719989	0.229145387
SSX5	0.526449331	0.004707603	1.28E-05	1.729760143	0.075382875
SSX6	0.05096898	4.061328634	0.879276346	18.75905151	0.072626517
SSX7	0.54210091	0.966109182	0.870995023	1.071609971	0.514383687
SSX8	0.953917102	0.904274694	0.257962343	3.169891829	0.875064198
SSX9	0.639268052	0.765552175	0.329593238	1.77816188	0.534381408
ST13	0.79033773	2.519580797	0.196574094	32.2946288	0.477676389
ST14	0.466834988	0.590583376	0.168579229	2.068989917	0.410324328
ST18	0.418039911	0.980228902	0.2309722	4.16001883	0.978398685
ST3GAL1	0.910262004	4.073044215	0.017805819	931.7004145	0.612385187
ST3GAL2	0.679407281	0.767259426	0.276379254	2.129997161	0.61106879
ST3GAL3	0.565477698	0.807105848	0.13027601	5.000305504	0.817858593
ST3GAL4	0.48044368	0.763528256	0.310164829	1.879566423	0.557195052
ST3GAL5	0.579576987	118.99068	0.491452577	28810.06752	0.087947713
ST3GAL6	0.600197492	0.016361567	0.000115505	2.317653296	0.103658325
ST5	0.090326972	0.000465324	1.10E-07	1.966934789	0.071677189
ST6GAL1	0.685296497	1.007971528	0.545611013	1.862144598	0.979772655
ST6GAL2	0.16257571	1.492448081	0.85016637	2.619959285	0.163133369
ST6GALNAC1	0.833961635	0.813271221	0.001251388	528.5412695	0.950127107
ST6GALNAC2	0.105833575	0.729565725	0.001499738	354.9061835	0.920437724
ST6GALNAC3	0.465146307	0.525502449	0.209026714	1.321136512	0.171348636
ST6GALNAC4	0.068338872	0.477522477	0.130426731	1.748320416	0.264306269
ST6GALNAC5	0.975058851	0.003981361	2.15E-06	7.358261934	0.149889849
ST6GALNAC6	0.320758343	0.425835618	0.071958075	2.520022574	0.346660066
ST7	0.11746473	0.003027364	1.56E-07	58.89562545	0.249697477
ST7L	0.242743566	0.864382338	0.623239844	1.19882712	0.382494468
ST8SIA1	0.111243847	0.022374899	4.17E-05	12.01531222	0.236105851
ST8SIA2	0.332604424	0.164890381	0.005374809	5.058568565	0.302116853
ST8SIA3	0.911933574	0.977329046	0.50221005	1.901937375	0.946179062
ST8SIA4	0.086795474	3.519454006	0.548806714	22.5699799	0.184464115

ST8SIA5	0.447620018	0.744763277	0.202531605	2.73869522	0.657366941
ST8SIA6	0.408208695	0.326003769	0.105630002	1.006138935	0.05125721
STAB1	0.278974757	0.20719716	0.003220952	13.32856194	0.458747387
STAB2	0.895099787	2.653886489	0.375011293	18.78107043	0.328276564
STAC	0.72156218	1.422876566	0.322776951	6.272373887	0.641243396
STAC2	0.424935392	0.351076703	0.018465947	6.674710594	0.486041856
STAC3	0.356984059	55.63639191	0.247824087	12490.34403	0.145690194
STAG1	0.072568374	2.09860169	0.797652314	5.52136435	0.133123898
STAG2	0.955184829	0.967353887	0.721994386	1.296095316	0.824027832
STAG3	0.952075504	1.006208021	0.880311244	1.150109791	0.927694441
STAM	0.968013006	0.703300833	0.224308543	2.205141432	0.546064025
STAM2	0.205631079	1.126376358	0.36217793	3.503039789	0.83712545
STAMBP	0.683347411	1.007961824	0.854577614	1.188876261	0.924985303
STAMBPL1	0.905746432	1.026724081	0.797813918	1.321313549	0.837638125
STAP2	0.968468172	0.023469436	1.84E-07	2996.205662	0.531654516
STAR	0.832773915	20.29201594	0.13315491	3092.382485	0.240485707
STARD10	0.830570095	0.513829309	0.105926795	2.492481319	0.408553494
STARD13	0.463072331	0.761833132	0.327836953	1.770360892	0.527189199
STARD3	0.056655503	0.519640665	0.177373241	1.522362784	0.232616695
STARD3NL	0.776721649	0.529847226	0.048064408	5.840872586	0.603970154
STARD4	0.972659232	1.073409384	0.828465656	1.390773048	0.59193434
STARD5	0.710247341	1.550023695	0.408910345	5.875550676	0.519164408
STARD6	0.720976524	0.319798699	0.002788097	36.68136948	0.637513517
STARD7	0.282637938	0.897794584	0.696919801	1.156567962	0.404095105
STARD8	0.45887401	0.48440501	0.115573344	2.030296999	0.321505181
STAT1	0.848169864	0.674985871	0.068333955	6.667343152	0.736589503
STAT2	0.958767131	1.698426535	0.00632437	456.1170242	0.852740535
STAT3	0.099944082	2.868115899	0.862093496	9.541991495	0.085795104
STAT4	0.549762016	1.071308629	0.186611427	6.150224537	0.938424017
STAT5A	0.530511519	0.073979435	0.000232236	23.56635272	0.375901106
STAT5B	0.402098859	351.9147031	0.056219822	2202852.185	0.188645815
STAT6	0.446876407	0.735512207	0.082511198	6.556421656	0.783148168
STATH	0.344050672	2.236275475	0.606002561	8.252321563	0.227006846

STAU2	0.2864595	0.864978107	0.673645977	1.110653298	0.255462233
STC1	0.515198594	0.814623792	0.308293227	2.152534868	0.679193893
STC2	0.833794758	2.776704361	0.235740628	32.70580546	0.417020519
STEAP1	0.960344521	1.153275094	0.491737883	2.704781322	0.742991468
STEAP2	0.269081567	1.085758188	0.852258438	1.383231646	0.505424145
STEAP3	0.981798189	0.720684837	0.156088118	3.327521913	0.674731558
STEAP4	0.811851137	0.433147974	0.029831356	6.289260451	0.539935357
STH	0.258054668	0.822957269	0.345887395	1.958032229	0.659508595
STIL	0.060775474	0.974059593	0.797001151	1.190452598	0.797352231
STIM1	0.993814727	2.228815959	0.636602137	7.803336324	0.209990021
STIM2	0.205496877	0.075689826	0.002448908	2.339389574	0.140356911
STIP1	0.065017046	0.751019388	0.402897911	1.3999331	0.367513042
STK10	0.160343327	0.496763889	0.146465544	1.684862899	0.261534006
STK11	0.312421127	1.170063067	0.769878137	1.778265305	0.462091415
STK11IP	0.267491777	0.000176689	6.39E-09	4.88560353	0.097728008
STK16	0.137721724	0.056801687	0.000549742	5.868993353	0.225475546
STK17A	0.871173835	0.264356007	0.000964076	72.48814861	0.642289195
STK17B	0.659423134	0.136647243	0.005691985	3.280484819	0.219681723
STK19	0.954414214	0.799836683	0.017287589	37.005664	0.909107601
STK24	0.950286685	0.749132031	0.246170398	2.279716831	0.610969909
STK25	0.734113328	1.124941808	0.315280244	4.013870494	0.856051246
STK3	0.251804774	0.015975229	1.74E-05	14.70736386	0.234854311
STK31	0.328005038	0.929086228	0.786286834	1.097819753	0.387658917
STK32A	0.17715556	1015.82129	0.523622306	1970681.693	0.073059061
STK32B	0.258200448	0.610950359	0.104894084	3.558449897	0.583637189
STK32C	0.787528287	6.757957595	6.56E-05	696203.5515	0.745602463
STK33	0.147802896	1.098863717	0.776101707	1.555854675	0.595170746
STK35	0.725950438	3.036220189	0.55338965	16.65848472	0.200997925
STK36	0.522758852	0.396377842	0.03722115	4.221132128	0.443233714
STK38	0.607672793	0.677745069	0.144959413	3.168737836	0.621081843
STK38L	0.228548025	10.78552665	0.502320346	231.5804768	0.128529136
STK39	0.18148758	2.018626324	0.585792202	6.956139436	0.265812355
STK4	0.779806396	6.445722155	0.00038956	106651.9954	0.70693224

STK40	0.581027202	17.13947975	0.021521101	13649.94129	0.404466336
STMN1	0.384282389	1.244786765	0.64433415	2.404798956	0.514578816
STMN2	0.077708515	1.521096648	0.912316352	2.536110427	0.107809005
STMN3	0.38633403	0.64515331	0.037611156	11.06647159	0.762478381
STMN4	0.31600924	1.002958951	0.499115224	2.015419706	0.993379315
STOM	0.2140934	0.4883945	0.224107197	1.064353092	0.071380687
STOML1	0.902174112	0.961782876	0.353896586	2.613832224	0.939109188
STOML2	0.13631262	2.083905985	0.829082751	5.237914006	0.118434077
STOML3	0.901295016	0.988783488	0.370921805	2.635846081	0.982010641
STON1	0.236758102	0.864001548	0.55772966	1.338459703	0.512739456
STON2	0.383287177	1.175462665	0.793594735	1.741080701	0.419921562
STOX1	0.3611881	0.01508395	4.96E-05	4.588950746	0.150524838
STOX2	0.955756712	0.589249562	0.144573964	2.40164298	0.460643136
STRA13	0.091176933	0.407376258	0.101217223	1.639596605	0.206229223
STRA6	0.89536325	0.313831256	0.063283361	1.556334178	0.156035196
STRAP	0.656205294	75.19050211	0.194743966	29030.99762	0.155146827
STRBP	0.268046781	0.197623729	0.004689941	8.327426956	0.395612929
STRC	0.598068543	3.539547617	0.055991525	223.7552436	0.550201544
STRN	0.59174234	3.217599833	0.050191881	206.2673991	0.581958701
STRN3	0.178739868	0.107341418	0.005661538	2.035168	0.137113315
STRN4	0.127823244	1.057184099	0.828527908	1.348944565	0.654722847
STS	0.988639819	1.115557745	0.458805787	2.712409298	0.80937496
STT3A	0.649347587	7.585502548	0.019337405	2975.572349	0.506049789
STT3B	0.894601961	27.1840662	0.018270837	40445.51705	0.375563175
STUB1	0.251132045	0.472344515	0.011442053	19.49906552	0.692743466
STX10	0.073542645	1.229577059	0.780494335	1.937054091	0.372801122
STX11	0.92905594	1.44117138	0.388691756	5.34350141	0.584652088
STX12	0.797595505	0.108098722	0.00236841	4.933831579	0.253783951
STX16	0.627669396	1.776152066	0.703725858	4.482876573	0.223940481
STX17	0.078710303	1.231766601	0.810380972	1.872266269	0.329180565
STX18	0.093130459	0.337092811	0.04058532	2.799819334	0.314050926
STX19	0.442970114	1.175958765	0.000717761	1926.657274	0.965764465
STX1A	0.726184591	4513.46827	0.023542973	865285630.8	0.175133105

STX6	0.713130394	1.124110007	0.374249192	3.376422272	0.834848636
STX7	0.505906792	0.2901124	0.013733371	6.12851747	0.426550562
STX8	0.94279876	1.453527088	0.529888996	3.987138834	0.467583473
STXBP1	0.215207798	106.6327347	0.844311549	13467.2327	0.058569222
STXBP2	0.305950923	6.42688689	0.022151334	1864.667601	0.520171431
STXBP3	0.960690863	0.602313881	0.026310741	13.78836174	0.750954782
STXBP4	0.926959976	0.899350845	0.42615876	1.897959209	0.780714735
STXBP5	0.252167967	0.662765048	0.371931313	1.181017822	0.162862478
STXBP6	0.42273304	0.624462234	0.218835238	1.781948305	0.378789991
STYK1	0.245603291	0.079675812	0.000237621	26.71583654	0.393844171
STYX	0.279481295	0.000739383	1.13E-07	4.822265271	0.107641396
STYXL1	0.76248003	1.188596937	0.464099631	3.044093516	0.718787245
SUB1	0.271018365	2.535015972	0.940128059	6.835564493	0.066066226
SUCLA2	0.326766046	2.033079198	0.255053644	16.20604577	0.502892389
SUCLG1	0.579994149	1.223049631	0.442260518	3.382283379	0.698046059
SUCLG2	0.222537956	0.332076743	0.070482216	1.56457855	0.163330232
SUCNR1	0.784009922	2.629861546	0.909240285	7.606539065	0.074361751
SUDS3	0.086867158	0.693512297	0.218885183	2.19731322	0.533932682
SUFU	0.882888044	1.833702436	0.153612453	21.88927104	0.631754452
SUGT1	0.185795391	0.439902892	0.124959305	1.548620599	0.200947656
SUGT1P	0.920060102	1.122720389	0.035932234	35.07995297	0.947444628
SULF1	0.055261206	1.091693409	0.94635601	1.259351118	0.22876143
SULF2	0.834057874	0.923878465	0.461803285	1.848300881	0.822927024
SULT1A1	0.371096578	2.67572362	0.729386983	9.81577278	0.137773053
SULT1A2	0.594384279	0.193844506	0.019631359	1.91406479	0.160234105
SULT1A3	0.574558493	5.871268856	0.048554663	709.9585397	0.469373496
SULT1A4	0.99044431	1.103712288	0.386051572	3.155487257	0.853921913
SULT1B1	0.548182352	0.333325496	0.003841613	28.92167783	0.62948589
SULT1C2	0.069924923	0.577766727	0.266104418	1.254448887	0.165484377
SULT1C3	0.093687928	0.401147793	0.007545039	21.32786259	0.652305282
SULT1E1	0.646095956	0.947753403	0.78131814	1.149642465	0.586011764
SULT2A1	0.494092804	0.666362541	0.167138313	2.656716035	0.565115599
SULT2B1	0.999715185	0.017339059	9.00E-07	333.8735806	0.420499397

SULT4A1	0.760110946	1.049489142	0.82805406	1.330139554	0.689526384
SULT6B1	0.050930966	0.542301943	0.255575613	1.150702109	0.110878951
SUMF1	0.941183408	0.727267398	0.338923388	1.560582381	0.413646865
SUMF2	0.971251446	0.965462798	0.313530512	2.972975126	0.951160321
SUMO1	0.619582994	0.29294257	1.06E-05	8085.788761	0.813952484
SUMO2	0.401859832	1.77373441	0.502520416	6.260708337	0.373143809
SUMO3	0.994487102	0.882015267	0.399480244	1.947407769	0.756050707
SUMO4	0.522751847	0.634325088	0.229133838	1.756040573	0.380938418
SUOX	0.721900072	0.000809882	5.47E-08	11.9854248	0.146221405
SUPT16H	0.317441651	0.87573928	0.591954992	1.295570267	0.506667511
SUPT3H	0.925588268	1.042566036	0.802615846	1.354251781	0.754769938
SUPT4H1	0.706710748	0.312443707	0.007966723	12.25360436	0.534322792
SUPT5H	0.275806863	0.6981683	0.372935654	1.307032381	0.261421608
SUPT6H	0.582623004	0.691247594	0.299023561	1.597945108	0.387771591
SUPT7L	0.056931196	14.72024024	0.378069058	573.1372836	0.150049631
SUPV3L1	0.621183144	2.297176526	0.818795638	6.444856014	0.114077133
SURF1	0.532849832	0.318010137	0.015810667	6.396342795	0.45437377
SURF2	0.775608667	0.932826403	0.754666679	1.153045606	0.520196401
SURF4	0.432618161	0.516505268	0.106905235	2.495459574	0.411032926
SURF6	0.400378288	1.814585215	0.705145182	4.669562501	0.216623617
SUSD1	0.589003118	1.433031131	0.487730954	4.210473426	0.512927181
SUSD2	0.339341019	0.680875031	0.283138354	1.637329602	0.39056663
SUSD3	0.740643141	1.068904781	0.787887853	1.450152361	0.668539555
SUSD4	0.674524665	0.611213853	0.208416555	1.792479364	0.36980965
SUV39H1	0.07719765	0.484590416	0.197607847	1.188352966	0.1134427
SUV39H2	0.468102027	1.064244857	0.840060696	1.348256288	0.605913272
SUV420H1	0.197896527	0.657979817	0.363373863	1.19143803	0.167048124
SUV420H2	0.136822907	2.861874332	0.378285822	21.65115426	0.308478349
SUZ12	0.197111912	1.584546601	0.674757643	3.721021844	0.29061467
SV2A	0.284067824	0.969266255	0.75407285	1.245870439	0.80745969
SV2B	0.785823628	1508.915216	0.580471872	3922369.437	0.068093619
SV2C	0.338849042	0.81959665	0.561674953	1.195956246	0.302147065
SVIL	0.534771069	0.024174867	1.12E-05	52.33112877	0.342124145

SVOP	0.130460064	15.49961942	0.005838168	41149.58601	0.495647525
SWAP70	0.078035252	0.535052362	0.275828523	1.037894947	0.064322144
SYAP1	0.530290498	1.159079897	0.873925941	1.537276952	0.305535617
SYCE1	0.086523953	1.909827514	0.669805614	5.445521887	0.226166324
SYCN	0.116943084	1.7093125	0.531462352	5.497565752	0.368427008
SYCP1	0.143971744	0.399109722	0.023964543	6.646843724	0.522135404
SYCP2	0.151372026	1.453871534	0.941670026	2.244674225	0.091266494
SYCP3	0.118081315	0.424403677	0.11057414	1.628938568	0.211685957
SYDE1	0.314852727	0.962134041	0.848644662	1.090800371	0.546651345
SYDE2	0.262934809	0.516008632	0.122224911	2.178483145	0.367920787
SYF2	0.954605202	0.997132448	0.261756904	3.798459964	0.996642328
SYK	0.287477774	0.484261298	0.1863248	1.258603285	0.136753192
SYMPK	0.586083373	0.552672405	0.162526074	1.879370982	0.342316502
SYN1	0.511831687	0.908155726	0.655921869	1.257385766	0.561697543
SYN2	0.824584129	0.785380534	0.350298723	1.760847366	0.557562794
SYN3	0.483669358	0.684002132	0.288231765	1.623203871	0.389040402
SYNCRIP	0.109603412	2.541257006	0.950920827	6.791298485	0.062938975
SYNE1	0.256267417	0.948276787	0.03414887	26.33260962	0.975017615
SYNE2	0.566555635	0.28700604	0.005781509	14.24757271	0.530962246
SYNGAP1	0.394717639	0.579935914	0.120313616	2.795408168	0.497169975
SYNGR1	0.599700144	154.4233765	0.022180579	1075110.751	0.264277273
SYNGR2	0.903789311	0.674295084	0.077161164	5.892522065	0.721608928
SYNGR3	0.548282703	1.054997683	0.292201822	3.809079987	0.934858522
SYNGR4	0.124931507	0.782955476	0.080920307	7.575592578	0.832655213
SYNJ1	0.244657389	1.085215483	0.248792411	4.733635715	0.913345023
SYNJ2	0.801955467	0.048566044	0.000569185	4.143922801	0.182427767
SYNJ2BP	0.975339195	1.068820327	0.310041781	3.68459015	0.916056474
SYNPO	0.822809969	2.803026895	0.06973827	112.6635319	0.584438721
SYNPO2	0.079709637	2.899115051	0.95855793	8.768242184	0.059428749
SYNPO2L	0.731793509	0.965427881	0.701158047	1.329302284	0.829294588
SYNPR	0.077708515	2.778632139	0.83352858	9.262785646	0.09620047
SYP	0.250511053	1.349279384	0.473211051	3.847236559	0.575226614
SYPL1	0.077708515	1.969084158	0.715288634	5.420598397	0.189710358

SYPL2	0.606939511	0.672254425	0.248834522	1.816170872	0.433536019
SYT1	0.826618107	1.130950671	0.904718323	1.413754301	0.279853059
SYT10	0.058423701	4.055723987	0.999444075	16.4580465	0.050091023
SYT11	0.96333103	0.86150929	0.263976437	2.811607985	0.804899907
SYT12	0.313246896	0.949106221	0.166109148	5.422956106	0.953158588
SYT13	0.626982305	1.11329647	0.543409122	2.280839575	0.769299425
SYT14	0.909737688	0.62832899	0.221232886	1.784532702	0.382924689
SYT15	0.744754496	0.01982395	3.55E-06	110.6222242	0.373046771
SYT16	0.255645622	5.592680816	0.581961472	53.74596125	0.135944351
SYT17	0.290608171	2.574167056	0.81941657	8.086651256	0.105457429
SYT2	0.354307807	0.197289751	0.003605059	10.79684108	0.426711578
SYT3	0.590751028	1.162245869	0.422129791	3.200000303	0.771077722
SYT4	0.254130498	1.112616057	0.55589489	2.226885897	0.763090574
SYT5	0.266023073	0.57261405	0.205832528	1.592978784	0.285502133
SYT6	0.62526322	0.676637855	0.302544302	1.513295026	0.341521543
SYT7	0.31565823	3.37320008	0.056235108	202.3376348	0.560518425
SYT8	0.135587713	0.005687102	1.47E-06	21.9792579	0.219934301
SYT9	0.269551217	5.189984655	0.179751105	149.8513222	0.337184449
SYTL1	0.538665884	35.50541209	0.001186067	1062869.572	0.4972523
SYTL2	0.486378041	5.446380207	0.001093926	27116.14681	0.696362893
SYTL3	0.582337456	0.933872195	0.76011726	1.147345708	0.514817793
SYTL4	0.101586444	0.750930897	0.456898205	1.234185658	0.258500475
SYTL5	0.080261843	6.069668538	0.312630437	117.8416167	0.233407464
SYVN1	0.460523303	1.163627352	0.667753921	2.027735924	0.592786712
T	0.244910427	2.658297536	0.651769581	10.84209205	0.172839518
TAAR1	0.927424217	0.760872961	0.018421273	31.42712617	0.88553908
TAAR2	0.700173495	2.287740766	0.846838582	6.180348799	0.102658438
TAAR5	0.779023283	1.005322746	0.903763797	1.118294212	0.922169938
TAAR6	0.850522872	1.663060164	0.121563983	22.75155062	0.703127233
TAAR8	0.085906094	0.886805295	0.284672428	2.762556376	0.835845682
TAAR9	0.29086892	2.55761002	0.997807017	6.555745652	0.050536747
TAB3	0.362489026	0.594344476	0.218141944	1.619337158	0.308959167
TAC1	0.645120169	1.629849984	0.002593198	1024.376565	0.881877012

TAC3	0.929197633	0.036855665	9.85E-05	13.78667516	0.274844571
TAC4	0.054705256	2.459611864	1.128373328	5.361426374	0.023589449
TACC1	0.592843364	1.17397813	0.849749456	1.621918837	0.330723431
TACC2	0.856950628	0.844915398	0.186398085	3.82987856	0.82700845
TACC3	0.391976936	1.887829096	0.149098037	23.90305574	0.623712631
TACR1	0.736754567	0.095951609	2.78E-05	331.3526117	0.572835959
TACR2	0.503665293	1.082821033	0.691038916	1.696722663	0.728413943
TACR3	0.749722869	1.126648998	0.859916494	1.476117707	0.386985597
TACSTD2	0.073474946	0.576712703	0.238193917	1.396330969	0.222469282
TAF1	0.086907423	3.138169628	0.886742469	11.10593996	0.076136498
TAF10	0.624861353	0.879766441	0.523483597	1.478535325	0.628659175
TAF11	0.261943188	0.817603038	0.600091259	1.113955115	0.20191708
TAF12	0.239468238	2.545353852	0.87282561	7.422818665	0.087103514
TAF13	0.167757567	1.781160577	0.770079969	4.119744866	0.177242275
TAF15	0.347427519	1.005241564	0.799448486	1.264009653	0.964320621
TAF1A	0.686318316	0.358546435	0.005796701	22.17736453	0.625989178
TAF1B	0.062376679	1.808970526	0.519972803	6.29335678	0.351408834
TAF1C	0.66001294	0.873448753	0.454789872	1.677505966	0.684479994
TAF1L	0.703319748	0.028073849	9.30E-05	8.470223712	0.220002757
TAF2	0.114548657	0.018288725	1.64E-06	204.1945093	0.400097883
TAF3	0.685537431	2.100383273	0.124199068	35.52047513	0.607019229
TAF4	0.102786645	0.784685984	0.427924717	1.43887948	0.433167825
TAF4B	0.491590831	6.393708156	5.69E-05	718861.554	0.754533805
TAF5	0.812805315	0.82507227	0.311916304	2.182458056	0.698435498
TAF5L	0.12856938	0.369042072	0.108615999	1.253885728	0.110174295
TAF6	0.077672752	0.242421608	0.018522101	3.17287093	0.280147575
TAF6L	0.332403776	1.511687473	0.962685189	2.373776019	0.072686982
TAF7	0.436803321	0.008142385	2.36E-05	2.80842578	0.106614168
TAF7L	0.207994208	1.329804382	0.985099748	1.795127548	0.062617274
TAF9	0.159621976	1.21852766	0.23758844	6.249502967	0.812698436
TAGAP	0.05992501	3.344272501	0.529685326	21.11472227	0.199120937
TAGLN	0.594864204	0.874605668	0.346227586	2.209341788	0.776886769
TAGLN2	0.605808784	1.032116053	0.828290659	1.286098709	0.778236264

TAGLN3	0.27518332	0.559545621	0.233345417	1.341750384	0.193198621
TAL1	0.208678124	0.558018676	0.045448613	6.851360667	0.648444816
TAL2	0.914489594	0.251094887	0.00049173	128.2180619	0.664026793
TALDO1	0.059917769	0.650809756	0.281852022	1.502750756	0.314402551
TANC1	0.531135987	1.459306043	0.495150466	4.300862614	0.493107757
TANK	0.785013726	1.675349172	0.265517524	10.57103429	0.582978242
TAOK1	0.727822311	0.591538869	0.000435791	802.949476	0.886560509
TAOK2	0.194450504	0.78565312	0.45438678	1.358426021	0.387864857
TAOK3	0.688201017	2.234710118	0.051692761	96.60790491	0.675634212
TAP1	0.892528693	0.91338364	0.406614237	2.051747329	0.826326071
TAP2	0.937396264	0.300676922	0.054055505	1.67247741	0.169893046
TAPBP	0.06134564	0.00351796	2.97E-06	4.166778413	0.117648602
TAPBPL	0.544794946	764.0389102	0.227516597	2565770.869	0.109031089
TARBP1	0.568756648	1.120192657	0.270969183	4.630901478	0.875447609
TARBP2	0.64987581	1.268351118	0.293464503	5.48180289	0.750248432
TARDBP	0.846475225	0.967398064	0.834622808	1.121295758	0.659906859
TARS	0.144378138	964.3949494	0.151853961	6124684.598	0.124030518
TARSL2	0.111740645	2.045500579	0.754831454	5.543055473	0.159430181
TAS1R1	0.255845622	0.032149313	0.000685033	1.508801694	0.080032264
TAS1R2	0.65503186	0.243110109	0.00814845	7.253222626	0.41433497
TAS1R3	0.639974937	1.019663997	0.431298931	2.410659039	0.964619162
TAS2R1	0.150400441	29.78694061	0.739195834	1200.30686	0.071904109
TAS2R10	0.062008194	0.680790707	0.276436585	1.676608712	0.403066142
TAS2R13	0.603734767	1.524679345	0.387972522	5.991782853	0.545822103
TAS2R14	0.951526165	0.266054142	0.006267476	11.29398926	0.488726366
TAS2R16	0.66008423	0.952940289	0.418389957	2.170451698	0.908622977
TAS2R3	0.766203233	1.106878464	0.224348541	5.461055958	0.900766589
TAS2R38	0.80868174	712.2883409	0.08871347	5719026.443	0.152171631
TAS2R39	0.853293179	3.315074439	0.002446858	4491.35914	0.7446288
TAS2R4	0.870970008	0.974248088	0.274284176	3.460496155	0.967820126
TAS2R40	0.461124472	0.677656362	0.090509711	5.073689207	0.704815168
TAS2R41	0.39309009	1.585112687	0.909821581	2.761620831	0.103882865
TAS2R43	0.348853265	0.260019974	0.006552384	10.31844082	0.473233497

TAS2R46	0.34108942	1.457959236	0.082982613	25.61554849	0.796538716
TAS2R5	0.847428314	0.640093039	5.07E-05	8080.848912	0.926224418
TAS2R50	0.472376563	1.332983383	0.886440879	2.00447062	0.167325185
TAS2R60	0.595177487	0.29470343	0.040290053	2.155621665	0.228811336
TAS2R7	0.751438581	0.925947633	0.415301474	2.064473818	0.85082378
TAS2R8	0.876663681	0.674474038	0.154965545	2.935589511	0.599704355
TAS2R9	0.770502346	0.714015363	0.101080607	5.043677053	0.735582876
TASP1	0.35204801	1.983028417	0.566857943	6.937190797	0.283933353
TAT	0.998569162	3.789779782	0.664221273	21.62296119	0.13374777
TATDN1	0.59198553	1.460422326	0.523801633	4.071834136	0.469112259
TATDN2	0.601284649	1.030819294	0.689404584	1.541313247	0.882431025
TATDN3	0.360012067	0.348577686	0.052784095	2.301951062	0.273837855
TAX1BP1	0.182547673	0.761274024	0.472356168	1.226909222	0.262649289
TAX1BP3	0.748641826	0.324272685	0.033671	3.122947774	0.329794769
TAZ	0.247446381	2.764051179	0.665667203	11.47717492	0.161605397
TBC1D1	0.303198442	4936.77718	0.034760306	701137932.5	0.160024131
TBC1D10A	0.431883828	0.548508698	0.03165875	9.503274444	0.679836098
TBC1D10B	0.66381301	0.722091545	0.298966981	1.744059488	0.469250915
TBC1D10C	0.618300118	14.40266828	0.000571042	363260.1382	0.605982829
TBC1D12	0.102753631	1.620025135	0.891171843	2.944977963	0.113622572
TBC1D13	0.340450407	1.627510133	0.875410969	3.025766554	0.123706331
TBC1D14	0.963921553	0.146155728	2.02E-05	1059.433686	0.671531523
TBC1D15	0.186453735	0.594995679	0.246649862	1.435313425	0.247839128
TBC1D16	0.897966642	1.199760914	0.637253699	2.258796227	0.572641349
TBC1D17	0.866312475	1.401291846	0.788069897	2.491681062	0.250585184
TBC1D19	0.301948567	0.61104424	0.284598077	1.311938112	0.206399616
TBC1D2	0.545198282	0.958601776	0.695075266	1.322040086	0.796572979
TBC1D20	0.671147997	1.052619305	0.821100485	1.349417546	0.685739034
TBC1D21	0.304814389	0.395681608	0.075575938	2.071610859	0.272346038
TBC1D22A	0.710688906	0.958005567	0.851068223	1.078379666	0.477446151
TBC1D22B	0.34714144	1.590138676	0.21264299	11.8910151	0.651388525
TBC1D23	0.291716376	0.033104934	2.18E-05	50.34476085	0.36194794
TBC1D2B	0.48074925	1.249810119	0.835973358	1.868510904	0.277126561

TBC1D3	0.699178812	0.374027347	0.001680122	83.26564919	0.721406189
TBC1D3C	0.191740703	0.557517044	0.121463195	2.559007718	0.452377177
TBC1D4	0.696211982	1.487466261	0.71540402	3.092736157	0.287686423
TBC1D5	0.323559322	1.543412209	0.342505522	6.954986402	0.572060565
TBC1D7	0.403427369	0.780463775	0.175380621	3.473152854	0.744872798
TBC1D8	0.488041843	0.632084956	0.169778	2.353257729	0.493995726
TBC1D9	0.758917464	6.211407054	0.482672116	79.93330525	0.161170582
TBCA	0.179385827	1.765546455	0.367730037	8.476746434	0.477598831
TBCC	0.254083317	1.408461519	0.147938494	13.4093825	0.765786607
TBCD	0.982569338	1.026871143	0.648222209	1.626701971	0.910052993
TBCE	0.257351144	0.674343734	0.170328802	2.669774386	0.574641683
TBK1	0.960711414	1.62682883	0.619293733	4.273532743	0.323373841
TBKBP1	0.936850854	7.659736979	0.00261369	22447.7967	0.617167006
TBL1X	0.186435583	0.070414408	0.000467485	10.60609733	0.29972179
TBL1XR1	0.33972366	1.191176523	0.683471615	2.076021121	0.53708235
TBL1Y	0.40388468	1.212420597	0.416681568	3.527786723	0.723734895
TBL2	0.489038246	0.949187736	0.321966433	2.798295926	0.924682617
TBL3	0.613254757	1.010734539	0.461509441	2.21357185	0.978702825
TBP	0.177793881	0.093852766	0.003698361	2.381687729	0.151571653
TBPL1	0.843090147	0.812186654	0.338580401	1.948273321	0.641226686
TBPL2	0.517106536	0.951493528	0.511341056	1.770520718	0.875298512
TBR1	0.922793109	7.538105944	0.476894243	119.1522901	0.151509049
TBRG1	0.190463768	0.5618102	0.324987619	0.971208386	0.038963721
TBRG4	0.327921398	1.321872195	0.212265937	8.231872358	0.764912618
TBX1	0.360200019	0.246807114	0.014893065	4.090074895	0.32871977
TBX10	0.381917627	0.544922174	0.175853533	1.688565315	0.292752847
TBX15	0.188744298	2.582390802	0.715537143	9.319910677	0.147392494
TBX18	0.293123074	0.020333083	6.74E-05	6.130387003	0.181083496
TBX19	0.847164279	1.174935145	0.397003659	3.477228894	0.770889229
TBX2	0.181278005	1.925790905	0.655028917	5.661842572	0.23363649
TBX20	0.14558004	0.704125305	0.307028771	1.61480777	0.407465161
TBX21	0.080552767	1399.698359	0.029828229	65681253.4	0.186845238
TBX22	0.899535224	0.887009172	0.697677497	1.127720579	0.327696684

TBX3	0.978633704	0.3137117	0.003189295	30.85792495	0.620484
TBX4	0.264129633	1.176182349	0.740655916	1.867810531	0.491649142
TBX5	0.569162523	0.019078496	0.000197555	1.842465354	0.089527975
TBX6	0.340866558	0.513655268	0.1182035	2.232097467	0.374125948
TBXA2R	0.962866129	0.369580778	0.001215315	112.3905627	0.732932568
TBXAS1	0.180982183	2.04602112	0.044729765	93.58874224	0.71360214
TCAP	0.208063055	3635.842396	0.000247955	53313491094	0.330143319
TCEA1	0.059913725	4.495622125	0.249810677	80.90374085	0.308045159
TCEA2	0.185363842	1.211051199	0.311440836	4.709225111	0.782268983
TCEA3	0.621331925	0.98955778	0.215938742	4.534733284	0.989216608
TCEAL1	0.094748855	0.475669839	0.131652881	1.71862396	0.256915743
TCEAL2	0.390035111	2592.494963	0.112605966	59686270.46	0.12507387
TCEAL3	0.675859262	0.885394041	0.55385017	1.415405555	0.611080738
TCEAL4	0.717604118	3.815787854	0.835266594	17.43184397	0.084037044
TCEAL5	0.50839338	0.922252443	0.715881391	1.18811521	0.53115024
TCEAL6	0.605617256	0.471105296	0.05141152	4.316935208	0.505446546
TCEAL7	0.456006218	1.052906236	0.76982474	1.440083028	0.74694194
TCEAL8	0.386118736	0.095143557	6.43E-05	140.7719552	0.527631537
TCEB1	0.138207906	0.571318452	0.170699449	1.912160677	0.36374613
TCEB2	0.912269161	1.136145067	0.411318308	3.138264423	0.805508011
TCEB3	0.777961559	1.06579684	0.380011051	2.989183866	0.90360682
TCEB3B	0.994092566	0.554447648	0.043045181	7.141616912	0.651052843
TCEB3C	0.8604139	1.402732491	0.530747561	3.70733393	0.494936096
TCERG1	0.393985177	5.766746763	0.210479636	157.9980318	0.299580078
TCERG1L	0.390386594	0.078185112	0.002812468	2.173504649	0.133008758
TCF12	0.961073367	0.941951288	0.297178021	2.985658981	0.919073356
TCF15	0.79440423	0.937663258	0.380087308	2.313185333	0.888893347
TCF19	0.76498924	0.945686669	0.414925331	2.155383654	0.894303684
TCF20	0.1908993	0.161195291	0.009844058	2.639554006	0.20071529
TCF21	0.246253231	0.000140883	2.88E-09	6.899355076	0.107524544
TCF23	0.603050424	1.03439162	0.813569193	1.315150613	0.782563676
TCF3	0.460742186	1.255026593	0.030038351	52.43602572	0.905050562
TCF4	0.55251848	2.785929184	0.569898589	13.61891672	0.205703829

TCF7	0.189947517	1.40645257	0.468159551	4.225287786	0.543382865
TCF7L1	0.263221797	0.922831349	0.24395269	3.490913334	0.905825514
TCF7L2	0.753562369	6.341213842	0.018237816	2204.814019	0.536116903
TCFL5	0.595936209	1.071023833	0.20764968	5.524169599	0.934665836
TCHHL1	0.646044862	0.697972941	0.37589018	1.296033396	0.254807057
TCHP	0.598052252	0.304218219	0.001554816	59.52391084	0.658459964
TCIRG1	0.896342936	0.119791489	0.001410265	10.17539433	0.349115569
TCL1A	0.134527261	0.317229958	0.069421194	1.449627136	0.138605278
TCL1B	0.115921762	279.7821764	0.036241046	2159928.452	0.217360435
TCL6	0.085118786	1.248752425	0.757536416	2.058491954	0.383704537
TCN1	0.718758819	0.989543926	0.77724895	1.259824387	0.9320134
TCN2	0.067063659	0.04879778	0.000281195	8.468237344	0.250993356
TCOF1	0.423432115	0.285160951	0.020141007	4.037373473	0.353467225
TCP1	0.945225232	1.777996258	0.406977076	7.767687377	0.444290733
TCP10	0.700852463	10.01611082	0.114373411	877.1485852	0.312609642
TCP10L	0.152410413	1.188007549	0.474123006	2.976784338	0.713177231
TCP10L2	0.408837345	1.677532065	0.693693607	4.056710057	0.250876935
TCP11	0.318118233	0.890826873	0.558472419	1.420969937	0.627503599
TCP11L1	0.63899913	496.7935459	0.436595656	565291.5325	0.083784687
TCP11L2	0.865980648	1.208681963	0.010428573	140.0874413	0.93770094
TCTA	0.563147134	0.862417674	0.422539476	1.760224279	0.684287718
TCTE3	0.14470359	1.253582578	0.761459975	2.063758216	0.374245086
TCTEX1D1	0.899630436	1.025258796	0.463714229	2.266817651	0.950865293
TDG	0.200052786	1.59174521	0.454164213	5.578715232	0.467567261
TDGF1	0.099921283	0.68613432	0.36079663	1.30483565	0.25071457
TDH	0.267424166	2.937155157	0.0929252	92.8368234	0.540870297
TDO2	0.264123376	0.446868821	0.033479959	5.964515775	0.5423661
TDP1	0.501516574	0.396210388	0.015875657	9.888263251	0.572737898
TDRD1	0.768515414	0.065493854	1.20E-05	356.0217687	0.534494312
TDRD10	0.479953546	0.808010806	0.321937954	2.027972949	0.649792466
TDRD3	0.735850484	0.149782619	0.007279445	3.08194276	0.218517744
TDRD5	0.408954288	1.753807679	0.730684443	4.209534506	0.208545189
TDRD6	0.70210635	1.155171284	0.381375399	3.498968991	0.798635222

TDRD7	0.215938466	1.493223808	0.701764379	3.177301967	0.298017548
TDRD9	0.634431182	0.599330079	0.058243838	6.167116692	0.66688846
TDRKH	0.782883978	0.926769312	0.716976805	1.197948596	0.561407778
TEAD1	0.577598333	0.991248213	0.472409151	2.079919529	0.981453373
TEAD2	0.569597579	1.897853461	0.564120981	6.384885301	0.300621729
TEAD3	0.766673475	0.844121192	0.328148933	2.171393887	0.725192066
TEAD4	0.099893795	1.033693005	0.734118557	1.455515896	0.849477047
TEC	0.859528322	0.882191397	0.45799279	1.699288023	0.707841114
TECTA	0.950370387	0.958255605	0.049134293	18.68865425	0.977555109
TECTB	0.168368983	0.341051455	0.007962807	14.6074242	0.574696309
TEDDM1	0.362009402	1.069309439	0.907892706	1.259424894	0.422191824
TEF	0.214813702	42.01517454	0.030879094	57167.31408	0.309943112
TEK	0.821848447	0.906770656	0.73474573	1.119071523	0.361867951
TEKT1	0.581389658	1.319105032	0.628683561	2.767748665	0.463882173
TEKT2	0.659630146	1.514557093	0.516238206	4.443458777	0.449684936
TEKT3	0.3412406	0.885549574	0.537392718	1.459264373	0.633396616
TEKT4	0.34567779	1.383830478	0.506087391	3.783905364	0.526753572
TEP1	0.543940392	46.52881877	0.01271669	170243.2715	0.358983097
TEPP	0.596435065	1.027511342	0.375077685	2.814829033	0.957904685
TERC	0.560611979	1.299075834	0.511614419	3.298574002	0.58208367
TERF1	0.141550165	1.964789349	0.635543874	6.074163162	0.240862211
TERF2	0.924638862	0.476981078	0.132942143	1.711353107	0.256085078
TERF2IP	0.507540843	0.834119674	0.561678378	1.238708234	0.368669053
TERT	0.053077603	1.737427499	1.149976846	2.624969647	0.00869871
TES	0.876508989	1.192067983	0.338258644	4.201004475	0.784569344
TESC	0.430249766	0.962044397	0.351189976	2.635409565	0.940009292
TESK1	0.873277519	0.618442275	9.64E-05	3966.446384	0.914437625
TESK2	0.367048057	1.159746776	0.539889979	2.491271627	0.704018074
TEX10	0.570930075	0.507791457	0.032235507	7.999010663	0.629970352
TEX101	0.658391551	4.507531643	0.764134452	26.5893541	0.096336431
TEX11	0.704922652	0.740648026	0.406964952	1.347928109	0.325754868
TEX12	0.476426618	1.075560044	0.331948799	3.484963383	0.903342604
TEX13A	0.509994041	0.034961957	6.47E-05	18.89081571	0.296213207

TEX13B	0.312779904	3.788022476	0.397669472	36.08301692	0.246817015
TEX14	0.753920558	0.006677901	7.46E-07	59.75567461	0.280621486
TEX15	0.945036118	1.163939207	0.346514496	3.909661767	0.806015275
TEX2	0.344402377	0.580266502	0.019336961	17.41272625	0.75381491
TEX261	0.309679354	0.008969033	2.74E-05	2.93687694	0.110632564
TEX264	0.675180745	1.423433446	0.347593626	5.829113718	0.623526917
TEX9	0.778181404	1.097157736	0.633155577	1.90119955	0.740970939
TF	0.130018113	1.308782298	0.942676357	1.817072309	0.107976331
TFAM	0.355792723	0.840830981	0.360476697	1.961282782	0.688284441
TFAP2A	0.275545762	0.735024778	0.403748905	1.338112422	0.313876881
TFAP2B	0.659286413	1.037430719	0.959241805	1.121992903	0.3580222
TFAP2C	0.426461647	0.864381753	0.697898653	1.070579248	0.181821399
TFAP2D	0.614488674	0.714572684	0.385854505	1.323333315	0.285111819
TFAP2E	0.172032378	1.140134519	0.567768818	2.289500023	0.712363097
TFAP4	0.356682203	0.370425306	0.009067004	15.13343358	0.599828336
TFB1M	0.557458778	1.224219897	0.527835096	2.839360946	0.637413606
TFB2M	0.203263797	6.301246332	0.010245548	3875.41065	0.574240577
TFCP2	0.247147201	1.226447584	0.945446339	1.590966737	0.124186686
TFCP2L1	0.820025836	6.778636129	0.151553085	303.1934837	0.323675612
TFDP1	0.475581488	0.022374425	0.000157137	3.185842048	0.133107735
TFDP2	0.238516825	0.176163177	2.15E-07	144496.6537	0.80265358
TFDP3	0.70820662	0.819962467	0.392880069	1.711307089	0.596963434
TFE3	0.457211943	0.641633741	0.197164483	2.088073121	0.46108884
TFEB	0.299871402	1.86451069	0.911344117	3.814585565	0.088048873
TFEC	0.948816696	0.93926276	0.343066388	2.571556299	0.902948978
TFF1	0.764124565	0.683152943	0.273940594	1.703646534	0.413780148
TFF2	0.144063665	0.646905692	0.328262833	1.274853359	0.208252034
TFF3	0.381666799	1.624587053	0.507837616	5.197100428	0.413420685
TFG	0.499869949	1.11263946	0.140458877	8.813729621	0.919485336
TFIP11	0.914196994	1.794726733	0.26564809	12.12522948	0.548496113
TFPI	0.181685627	1.116059386	0.096942316	12.84876007	0.929815189
TFPI2	0.841264852	1.373646708	0.18212276	10.3606231	0.758119795
TFPT	0.921574698	0.821338036	0.542720327	1.242990423	0.351841076

TFR2	0.384670304	4.050240579	0.060585696	270.7643872	0.514164969
TFRC	0.083057302	1.471532834	0.807664095	2.681076074	0.206917537
TG	0.70483155	0.947358906	0.104122546	8.619544315	0.961716157
TGDS	0.448259591	0.866405146	0.609137605	1.232328904	0.424999176
TGFA	0.066600763	0.041972856	7.94E-06	221.9481017	0.468525851
TGFB1	0.754992875	1.134709316	0.913438007	1.409581407	0.253501895
TGFB1I1	0.083765105	2.209168604	0.899276432	5.427058626	0.08390734
TGFB2	0.996091392	0.693048059	0.20766327	2.312954102	0.550984412
TGFB3	0.283496387	0.613195578	0.15888828	2.366498131	0.477832921
TGFBI	0.404176659	1.308447864	0.5121763	3.342668947	0.574258167
TGFBR1	0.171279544	2.35970546	0.808343664	6.888419009	0.116251745
TGFBR2	0.798894237	0.173058234	8.77E-05	341.430548	0.650454593
TGFBR3	0.254093248	1.2038748	0.792000984	1.829940321	0.385136015
TGFBRAP1	0.149867411	0.471557269	0.142983189	1.555191622	0.216957895
TGIF2	0.716673008	1.203460809	0.731552047	1.979787937	0.465878281
TGIF2LX	0.613296965	1.158338238	0.952603278	1.408506042	0.140677962
TGIF2LY	0.330125318	1.076639471	0.863668113	1.342127299	0.511407
TGM1	0.890256128	0.835553576	0.591814126	1.179677449	0.307276537
TGM2	0.059638094	2.261577767	0.825136898	6.198648989	0.112662972
TGM3	0.153964588	0.874833493	0.61245341	1.249619363	0.462309414
TGM4	0.881737254	1.311270222	0.10184022	16.88360061	0.835341743
TGM5	0.646923669	1.083002231	0.761676823	1.539883841	0.657028119
TGM6	0.75428238	0.896183532	0.1411223	5.691126929	0.907479492
TGM7	0.954222157	1.362604671	0.441486885	4.205541661	0.590527821
TGOLN2	0.269258921	1.939451496	0.044109479	85.27582237	0.731488165
TH	0.765034547	1.043556924	0.800523569	1.360373506	0.752621606
THADA	0.717301192	0.463944327	0.086605839	2.485332887	0.369810568
THAP1	0.915125347	1.412830426	0.209902865	9.509588241	0.722403568
THAP10	0.549797786	1.603075336	0.027413721	93.74322278	0.820161246
THAP11	0.309769042	1.826239437	0.952862718	3.500137445	0.069601997
THAP2	0.792927883	1.0856927	0.501165443	2.35197509	0.834872434
THAP3	0.942056501	0.973410885	0.776959847	1.219533744	0.814739269
THAP4	0.349987804	1.316916542	0.723510817	2.397019005	0.36765403

THAP5	0.1211302	0.781533075	0.545422247	1.119855215	0.179223845
THAP6	0.401546756	1.113775625	0.795596937	1.559201758	0.530146839
THAP7	0.784571494	0.964923996	0.333933541	2.788214437	0.947415886
THAP8	0.059762763	2.126669047	0.417487262	10.83319576	0.363674595
THAP9	0.617369692	1.084857327	0.888876881	1.324047735	0.423013034
THBD	0.99777521	1.263571642	0.126376687	12.63376447	0.84214897
THBS1	0.78344721	1.017919176	0.829504444	1.249130679	0.864959677
THBS2	0.402858375	0.45708043	0.154311526	1.353900935	0.157631089
THBS3	0.341656754	61.79408794	0.003130715	1219692.238	0.413804885
THBS4	0.643038492	0.352059378	2.67E-06	46420.74178	0.862215479
THEG	0.083765105	1.466463243	0.929712646	2.313095829	0.099654391
THEM4	0.32719863	0.229554856	0.017772121	2.965061468	0.259599224
THEM5	0.50567227	0.628859784	0.000389754	1014.651093	0.902039849
THNSL1	0.883480757	0.994840057	0.771651076	1.282583243	0.968163723
THOC1	0.420862541	0.666670032	0.219340536	2.026296371	0.474696175
THOC2	0.08948907	1.8799104	0.609808273	5.795367606	0.271812974
THOC3	0.081546212	1.101112028	0.563120334	2.153088116	0.778308711
THOP1	0.489252524	1.188210649	0.720983217	1.958221098	0.498695747
THPO	0.459848594	1.324623082	0.774936136	2.264220532	0.304048833
THRA	0.671825569	0.941458409	0.422793625	2.096398532	0.882585058
THRAP3	0.053995226	0.793562198	0.52641952	1.196272058	0.269518198
THRB	0.985108161	0.746768036	0.358855767	1.554001774	0.434828665
THRSP	0.255195591	0.757325294	0.193081969	2.970456553	0.690167243
THSD1	0.071870907	2.053844923	0.580253579	7.269716411	0.264427332
THSD4	0.587026787	1.010642535	0.356641131	2.86393869	0.984107291
THTPA	0.059576544	0.958520279	0.776611164	1.183038781	0.693176428
THUMPD1	0.737380252	0.039421474	0.00039861	3.898684104	0.167747588
THUMPD2	0.388585579	0.528310299	0.048061071	5.807439757	0.601887361
THUMPD3	0.875896125	6.855948196	0.002106413	22314.72373	0.640843427
THY1	0.206270226	0.001128679	1.32E-07	9.667350022	0.141855882
THYN1	0.35115991	1.184499954	0.893613908	1.5700742	0.238938777
TIA1	0.816540415	1.582928399	1.31E-05	191854.8221	0.93870077
TIAF1	0.220644856	0.053745624	0.00067355	4.288610094	0.190748419

TIAL1	0.963142046	0.468226434	0.041969995	5.223636394	0.537501414
TIAM1	0.319893284	0.782852503	0.250304063	2.448454226	0.673903711
TIAM2	0.595131005	1.354716092	0.729835886	2.51461421	0.336044929
TICAM1	0.82222492	0.4530279	0.099677861	2.05897554	0.305350041
TICAM2	0.953746422	1.012418924	0.403985477	2.537200309	0.978993367
TIE1	0.970376735	1.092650165	0.909981393	1.311987686	0.34246126
TIFA	0.891854573	0.691279805	0.283825905	1.683665091	0.416269452
TIFAB	0.935418247	22.12433489	0.492417019	994.048084	0.110698648
TIGD1	0.144667543	0.063738284	0.001125286	3.61025367	0.18133669
TIGD2	0.558586381	0.706715783	0.291605557	1.712749242	0.442150445
TIGD3	0.743676156	0.111004677	0.001881127	6.550347556	0.290709804
TIGD4	0.418083274	0.991249061	0.8415513	1.167575524	0.916198746
TIGD5	0.286675418	1.692256833	0.075304182	38.02887323	0.740426294
TIGD6	0.762516125	0.332877465	0.006900706	16.05740141	0.578072768
TIGD7	0.745892132	0.288447464	0.004424848	18.80334558	0.55967454
TIMD4	0.893727465	1.227399125	0.528693933	2.849491013	0.633496037
TIMELESS	0.300495525	0.179854779	0.001822556	17.74855784	0.46400282
TIMM10	0.869784038	0.136097304	2.13E-05	868.7561194	0.655488629
TIMM13	0.781486355	1.137520251	0.486508884	2.659668434	0.766209897
TIMM17A	0.662181604	1.162880754	0.371164936	3.643371232	0.795649516
TIMM17B	0.673479352	1.819262307	0.137901288	24.00061227	0.649342561
TIMM22	0.436389121	3.577327089	0.272502019	46.96210753	0.331907538
TIMM23	0.451092558	1.264128891	0.371472758	4.301854767	0.707579019
TIMM44	0.902233968	1.01490276	0.553977279	1.859331876	0.961804448
TIMM50	0.170848696	1.065826058	0.072797725	15.60467968	0.962867095
TIMM8A	0.564527044	1.081571966	0.915452795	1.277835322	0.35669677
TIMM8B	0.741925212	1.934947914	0.624237621	5.997753588	0.252799134
TIMM9	0.944925132	0.922112525	0.33866046	2.510749289	0.873933016
TIMP1	0.491534744	1.114117386	0.819294158	1.515032833	0.490787388
TIMP2	0.791959935	0.294834664	2.06E-05	4210.474665	0.80241633
TIMP3	0.207565832	2.026541101	0.628753184	6.531766263	0.236855947
TIMP4	0.198646807	1.427622626	0.636423195	3.20243885	0.387764387
TINAG	0.600171285	1.383715722	0.681311881	2.810268324	0.368957968

TINAGL1	0.246466127	0.000176348	5.48E-09	5.671040068	0.102629118
TINF2	0.790180895	0.946890059	0.516337747	1.736461823	0.859996684
TIPARP	0.817030826	0.996608414	0.006547388	151.6984002	0.998942776
TIPRL	0.765772976	0.335305542	0.02157582	5.210917043	0.435010675
TIRAP	0.191359033	2.501982073	0.57123299	10.95860079	0.223632347
TJAP1	0.708008087	72.64270668	0.003710194	1422287.671	0.395345398
TJP1	0.30242554	0.498126545	0.02562834	9.681862104	0.645271999
TJP2	0.533401446	0.415923711	0.043285498	3.996547139	0.447321919
TJP3	0.320691123	7.089489412	0.245884406	204.4084902	0.253457668
TK1	0.478666774	1.426818468	0.392734953	5.183676482	0.589182526
TK2	0.473415241	1.378741707	0.670897701	2.833410656	0.382168689
TKT	0.283339217	1.180238558	0.881300537	1.580576655	0.266118748
TKTL1	0.779611967	1.475718986	0.517702952	4.206556127	0.466536708
TKTL2	0.186978149	0.060411645	0.000918563	3.973127759	0.188829174
TLCD1	0.178902532	0.943710422	0.54748294	1.62669792	0.834800515
TLE1	0.848910431	0.560292439	0.145793153	2.153239782	0.399023046
TLE2	0.102543081	1.30857543	0.988867555	1.731647122	0.059885078
TLE3	0.107909104	0.639798009	0.338107944	1.21068286	0.169926254
TLE4	0.19112431	151.2831752	0.067618801	338465.0257	0.202159862
TLE6	0.474214435	1.02464691	0.710916485	1.476827884	0.896133447
TLK1	0.325447707	0.634883199	0.347417348	1.160208836	0.139704457
TLK2	0.970751321	2.568065256	0.444956509	14.82158149	0.291633383
TLL1	0.014730977	0.375210947	0.148217075	0.94984505	0.038588649
TLL2	0.699168304	1.042691866	0.145709722	7.461453573	0.96678874
TLN1	0.227091538	1.235816386	0.91029692	1.677740641	0.174646394
TLN2	0.386089063	0.844642904	0.370709294	1.924477338	0.687793373
TLR1	0.777983056	1.034068531	0.756387526	1.413690323	0.833684317
TLR10	0.100229648	13.13572334	0.658383817	262.0769575	0.091740702
TLR2	0.483214662	1.029623359	0.392628714	2.700068091	0.952674184
TLR3	0.741366364	1.106121229	0.679351296	1.800988944	0.685095979
TLR4	0.533177879	0.182616205	5.98E-07	55753.90375	0.791866174
TLR5	0.643140715	0.959250659	0.001081251	851.016175	0.990415777
TLR6	0.060910032	1.475354221	0.64250659	3.387778604	0.359176739

TLR7	0.77780449	0.872931601	0.363646229	2.095469495	0.760997463
TLR8	0.068950768	9213.417964	0.061141471	1388371409	0.133464662
TLR9	0.519317445	1.026095688	0.600401689	1.753613256	0.924939573
TLX1	0.631147115	1.141868361	0.604798644	2.155863557	0.682435374
TLX2	0.10535282	0.520776488	0.203469726	1.332916475	0.173623019
TLX3	0.576648251	1.22281074	0.364055933	4.107242787	0.744880687
TM2D1	0.223345897	1.140058538	0.744089416	1.746743661	0.547090269
TM2D2	0.664743452	1.214330472	0.415886494	3.545675366	0.722438696
TM2D3	0.951452937	1.050117232	0.910514663	1.211124044	0.501641979
TM4SF1	0.756913089	1.131954387	0.140356481	9.129045743	0.90735745
TM4SF18	0.984913038	110.3461936	0.019380301	628281.3778	0.286366129
TM4SF19	0.903846052	1.241139324	0.401975863	3.832137608	0.707239517
TM4SF20	0.176045272	0.102602665	0.000575765	18.28404931	0.389224165
TM4SF4	0.255885539	0.019678597	9.62E-05	4.026563689	0.14792368
TM4SF5	0.672910111	1.029596698	0.89299516	1.187094184	0.687966319
TM6SF1	0.89855329	0.008398503	3.20E-07	220.545834	0.357249283
TM6SF2	0.25858363	2.861632225	0.309832096	26.43024756	0.353958906
TM7SF2	0.220074673	0.008966448	7.19E-05	1.117482258	0.055512131
TM7SF3	0.42634236	0.788515362	0.371000251	1.675892333	0.536790866
TM9SF1	0.446896496	0.948420764	0.778238736	1.155817494	0.599698156
TM9SF2	0.526287463	1.069586968	0.723192074	1.581898257	0.736182699
TM9SF4	0.188278815	542.655883	0.914682255	321942.845	0.053286454
TMBIM1	0.429962159	0.847713104	0.59216181	1.213549228	0.366749046
TMBIM4	0.531955921	1.193251169	0.701824558	2.028781033	0.514111434
TMC1	0.306958709	1.045142099	0.002888426	378.1720791	0.988279994
TMC2	0.376488436	0.747327061	0.487665908	1.145246626	0.18113412
TMC3	0.531466481	0.634586693	0.257396948	1.564510666	0.323245528
TMC4	0.40845156	1.277446146	0.179327482	9.099936269	0.806894863
TMC5	0.185643983	0.914161295	0.43453587	1.923180416	0.813032667
TMC6	0.644357712	0.472230412	0.002212059	100.8117421	0.783951783
TMC7	0.620738465	0.83366352	0.450585401	1.542426503	0.562239572
TMC8	0.311315009	0.494264079	0.139544251	1.750677496	0.274791963
TMCC1	0.502883485	1.454245564	0.679121039	3.114069572	0.335078636

TMCC2	0.62992203	1.570352988	0.779996219	3.161564692	0.206215892
TMCC3	0.531550456	0.287932484	0.076308972	1.086439938	0.066121565
TMCO1	0.193988693	0.27114635	0.018542483	3.964967591	0.340320061
TMCO2	0.366533993	0.537133453	0.073933287	3.902333565	0.539041583
TMCO3	0.300237741	0.583464095	0.14617969	2.328848489	0.445519065
TMCO4	0.274834547	2.192932254	0.89422641	5.377778848	0.086217604
TMED1	0.598241316	4.803974329	0.000991539	23275.09141	0.716979458
TMED10	0.52768738	0.800444848	0.035463442	18.06682922	0.888677576
TMED2	0.894412853	0.570643681	0.100198605	3.249887664	0.527354278
TMED3	0.065272317	0.125283501	0.009429424	1.66457197	0.115518277
TMED4	0.40616575	0.148314444	0.014681087	1.498334149	0.1058149
TMED5	0.183212824	0.890918744	0.62422149	1.271561812	0.52454898
TMED6	0.508856623	0.050941343	1.84E-06	1410.62648	0.568378374
TMED7	0.27235075	1.043945993	0.775809277	1.404756645	0.776445335
TMED8	0.498850096	0.923487571	0.811035365	1.051531573	0.229558779
TMED9	0.162524483	0.00358529	6.36E-06	2.022142627	0.081489271
TMEFF1	0.763050937	1.94471982	0.101575875	37.23261238	0.658784982
TMEFF2	0.540473495	0.616804149	0.054592558	6.968850213	0.696095345
TMEM100	0.368220119	0.110858667	0.000559691	21.95792814	0.414995708
TMEM101	0.209520757	1.451030115	0.712715859	2.954176435	0.304750837
TMEM102	0.087593978	2.81272725	0.318270149	24.85760796	0.35226962
TMEM104	0.299212534	0.439278486	0.064275406	3.002168336	0.401531386
TMEM105	0.303286086	1.023943895	0.195961402	5.350344958	0.977624446
TMEM106A	0.4414444	0.550251341	0.10808074	2.80139217	0.471885998
TMEM106B	0.960559419	0.976055689	0.119084043	8.000103837	0.98198543
TMEM106C	0.363149334	0.912620831	0.483955333	1.720978621	0.777546539
TMEM107	0.150494607	0.740503466	0.312089023	1.757015924	0.495571566
TMEM108	0.053584036	223.626094	0.054445457	918508.7707	0.20253566
TMEM109	0.40698719	0.042658931	5.83E-05	31.20294615	0.348509126
TMEM11	0.437920418	0.814753425	0.569914864	1.164775978	0.261225089
TMEM110	0.788609186	0.686621467	0.324045108	1.454887074	0.326422988
TMEM115	0.164384189	0.649971889	0.268233461	1.574984174	0.340057528
TMEM116	0.368953508	0.682188312	0.323265785	1.439623103	0.315527649

TMEM117	0.210416334	0.366687081	0.054291008	2.476642475	0.303287267
TMEM119	0.835680066	1.008814358	0.528912373	1.924149371	0.978748766
TMEM121	0.213822198	1.12484341	0.69550691	1.819209386	0.631502878
TMEM123	0.342757041	12.63613639	5.51E-06	28994988.03	0.734272911
TMEM125	0.275743386	0.871850714	0.593389523	1.280985993	0.484825797
TMEM126A	0.65800571	0.672713188	0.225784059	2.004317912	0.47664528
TMEM126B	0.61548269	0.579664894	0.170681167	1.968649473	0.382038187
TMEM127	0.163923352	2.398289108	0.562985757	10.21658288	0.236803957
TMEM128	0.772105213	1.586119366	0.592385721	4.246852268	0.358626498
TMEM129	0.529574427	0.651714162	0.305376747	1.390843779	0.268302582
TMEM130	0.338635105	0.111889353	3.79E-06	3300.424885	0.676606234
TMEM14A	0.206884328	16.89779761	0.001460189	195546.9865	0.553692801
TMEM14B	0.481132179	0.080874774	3.49E-06	1874.886278	0.623855471
TMEM14C	0.645759841	0.026883756	3.86E-07	1873.306331	0.525056447
TMEM17	0.21352981	1.257735575	0.589167609	2.684972411	0.553411414
TMEM18	0.778291343	0.519168743	0.124458444	2.165672132	0.368352577
TMEM19	0.422897852	0.072262509	0.00054072	9.657257765	0.292799627
TMEM2	0.842898125	1.039101245	0.736092114	1.466842774	0.827384288
TMEM25	0.09455674	0.948574674	0.784930769	1.146335382	0.584766321
TMEM26	0.889112662	1.09964306	0.67942527	1.779761384	0.699017027
TMEM27	0.546572452	4.014701404	0.000950744	16952.85012	0.744174689
TMEM30A	0.393991142	0.769179086	0.01454444	40.67784383	0.896865258
TMEM30B	0.420271183	1.620660386	0.594358025	4.419121097	0.345473575
TMEM31	0.427927422	0.347355069	0.007608193	15.85863399	0.587560166
TMEM33	0.903346733	1.149009738	0.296313537	4.455494649	0.840792525
TMEM35	0.999962225	0.814458193	0.204871067	3.23785178	0.770704286
TMEM37	0.055884193	54.71486697	0.155698209	19227.68854	0.180856182
TMEM38A	0.181342922	0.80550886	0.379146395	1.711329798	0.573748077
TMEM38B	0.937275612	9.548820377	6.34E-05	1438718.949	0.710692418
TMEM39A	0.689859568	2.405265474	0.308510806	18.752348	0.402244991
TMEM39B	0.155586853	0.384262812	0.115152837	1.282277645	0.119810321
TMEM40	0.399950089	0.002055438	9.26E-08	45.63096565	0.22561562
TMEM41A	0.232260812	0.486231105	0.069458673	3.403760501	0.467677349

TMEM41B	0.760277058	1.002277401	0.840047614	1.195836965	0.97985502
TMEM42	0.634455205	1.094955014	0.406795316	2.947247508	0.85749649
TMEM43	0.843435124	0.982166596	0.19401458	4.972055301	0.982650577
TMEM44	0.198217669	0.798489616	0.316364458	2.01535176	0.633794682
TMEM45A	0.952189927	0.442068526	7.43E-05	2628.9372	0.853939013
TMEM45B	0.14127583	0.61284293	0.235118298	1.597393571	0.316467735
TMEM47	0.774367239	42.51765178	0.130736621	13827.42418	0.203875046
TMEM5	0.404483628	1.880963227	0.279940479	12.63848186	0.515675807
TMEM50A	0.85714455	1.198956499	0.664236535	2.164133724	0.547043041
TMEM50B	0.362973288	1.485535997	0.955785385	2.308904524	0.078580838
TMEM51	0.817324355	0.6663701	0.038946266	11.40158365	0.779352242
TMEM52	0.915187557	1.25521233	0.37197754	4.235626684	0.71413796
TMEM53	0.674909139	0.492427046	0.055205472	4.392397787	0.525757637
TMEM54	0.997952598	0.768306552	0.292875147	2.015517412	0.592216801
TMEM55A	0.345040619	76.24243314	0.637150673	9123.287248	0.075844789
TMEM55B	0.181322951	1.430947791	0.986633993	2.075350734	0.058888087
TMEM56	0.614403748	0.175443888	0.003539775	8.695624822	0.382153673
TMEM57	0.390865011	0.922426516	0.647391767	1.314305681	0.654876601
TMEM59	0.228309668	0.911554168	0.236913901	3.507312136	0.892849702
TMEM60	0.613648302	20.72697815	0.311971902	1377.071527	0.156805118
TMEM61	0.711169897	0.19970866	0.000731713	54.50707395	0.57351948
TMEM62	0.300415263	52.67799654	0.176800917	15695.45775	0.172618721
TMEM63A	0.442784557	0.006723166	1.78E-06	25.45944361	0.234076085
TMEM63B	0.333855242	0.005287869	2.49E-06	11.23974707	0.179906526
TMEM63C	0.492671817	1.082710596	0.214178128	5.473305078	0.923425483
TMEM64	0.831333991	0.759522357	0.227574853	2.534876782	0.654641794
TMEM65	0.217702577	0.154377783	0.007269625	3.278367336	0.230767048
TMEM67	0.852305966	1.024345102	0.678437187	1.546617592	0.908902951
TMEM68	0.629084966	0.83188542	0.213650989	3.239083302	0.790712476
TMEM69	0.809113088	0.929493245	0.69209024	1.248330989	0.627035575
TMEM70	0.130306541	0.470987484	0.0045669	48.57325776	0.750246645
TMEM71	0.399221591	0.627531305	0.297803348	1.322334156	0.220473152
TMEM74	0.375636611	1.122704289	0.815602	1.545441183	0.477795751

TMEM79	0.548419728	2.563104416	0.830084199	7.914262499	0.101792839
TMEM80	0.755029296	0.936715799	0.300651303	2.918452299	0.910228069
TMEM81	0.178348234	0.570745337	0.042216831	7.716122578	0.6729602
TMEM86A	0.664670794	1.508203478	0.725021469	3.137393622	0.271530674
TMEM86B	0.860001999	0.323205972	0.002470828	42.27817287	0.649675939
TMEM87A	0.334195324	2.020951087	0.965058162	4.2321214	0.062090175
TMEM87B	0.515996005	3.270858525	0.344419184	31.06248425	0.302138394
TMEM88	0.941790981	0.886171696	0.166849706	4.706632657	0.887204448
TMEM89	0.57095935	8.735843562	0.542229365	140.7429544	0.126421761
TMEM9	0.682659144	1.039197824	0.171653982	6.291331561	0.966618917
TMEM92	0.380137432	0.634503955	0.187584593	2.146206481	0.464376204
TMEM95	0.484747415	0.639102688	0.119572251	3.415945114	0.600628435
TMEM97	0.515078934	0.965905592	0.775894853	1.202448515	0.756268225
TMEM98	0.361809944	0.330260043	0.002016048	54.10172978	0.670203421
TMEM99	0.147366459	168.8596645	0.412923932	69052.87889	0.094586103
TMEM9B	0.312828989	0.674135285	0.215859838	2.105340144	0.497351801
TMF1	0.315460118	4.895677662	0.91305253	26.2500338	0.063767715
TMIE	0.695371984	0.092064533	0.003138393	2.700706506	0.166466826
TMLHE	0.943715925	0.816249342	0.303113677	2.198063102	0.687894978
TMOD1	0.57493443	2.482535972	0.885016113	6.963697903	0.084014971
TMOD2	0.77979368	1.073524297	0.767931818	1.500724918	0.678080786
TMOD3	0.233054399	1.028254845	0.414704284	2.549546912	0.952043895
TMPO	0.677888936	1.546480601	0.017335539	137.9594985	0.849095797
TMPRSS11A	0.651215716	0.436649089	0.167323624	1.139483014	0.090425658
TMPRSS11B	0.830942269	1694.430327	0.007338791	391221699.2	0.238003247
TMPRSS11D	0.365187304	1.335964481	0.744891236	2.396055973	0.331138588
TMPRSS11E	0.497237883	1.495202528	0.646017316	3.460635722	0.347474841
TMPRSS11F	0.331303074	146.3067947	0.097781348	218913.7159	0.181340474
TMPRSS12	0.870291806	1.115695807	0.850191208	1.464114333	0.429799978
TMPRSS13	0.341697413	1.306856126	0.402223082	4.246083847	0.656222733
TMPRSS2	0.441352018	2.04201511	0.079612629	52.37643541	0.666266001
TMPRSS3	0.523573953	0.444769496	2.82E-05	7017.116118	0.869512336
TMPRSS4	0.060727535	2.413230603	0.880565525	6.613570238	0.086769328

TMPRSS5	0.91214937	0.36309272	0.117319527	1.123737263	0.078819695
TMPRSS6	0.910121016	1.225634521	0.554228933	2.710396173	0.615342841
TMPRSS7	0.301468025	1.134447281	0.264820371	4.859787145	0.865055327
TMPRSS9	0.435873973	0.592252515	0.119498299	2.935297351	0.521251497
TMSB10	0.525375486	0.434952005	0.00238302	79.38803601	0.753994845
TMSB4Y	0.736062568	0.258520388	0.007174036	9.315926712	0.459491826
TMTC2	0.179281603	1.496731451	0.945017503	2.370543433	0.085628529
TMTC3	0.727512093	0.87751823	0.479293432	1.6066113	0.671981448
TMTC4	0.226266231	0.893554898	0.636935093	1.253566282	0.51466615
TNC	0.060743745	121.271364	0.110032973	133657.6062	0.179445636
TNF	0.553464837	0.933112681	0.64846488	1.342708451	0.709259905
TNFAIP1	0.665726997	0.9158158	0.411233492	2.039519144	0.829554406
TNFAIP2	0.232017996	0.64365789	0.314425358	1.317627439	0.228068844
TNFAIP3	0.056351761	81.09267597	0.090940474	72311.28002	0.204719199
TNFAIP6	0.188260308	1.586494141	0.001723378	1460.48231	0.894558264
TNFAIP8	0.111282498	16.77520451	0.528216893	532.7498797	0.109992651
TNFAIP8L1	0.734284598	0.818006489	0.36544428	1.831016803	0.625094348
TNFAIP8L2	0.079165107	0.548318273	0.167593627	1.793940104	0.320412144
TNFAIP8L3	0.600400758	0.730068305	0.197239012	2.702303792	0.637515046
TNFRSF10A	0.430989989	0.480514072	0.087645347	2.634409942	0.398557678
TNFRSF10B	0.93603746	0.575707697	4.20E-05	7883.549255	0.909538266
TNFRSF10C	0.89594036	0.713247051	0.270505896	1.880629458	0.494519589
TNFRSF10D	0.713837477	1.285919825	0.819852775	2.016935048	0.273501257
TNFRSF11A	0.114717056	2.349777297	0.648035397	8.520295918	0.193636133
TNFRSF11B	0.293337802	2.02683242	0.754692567	5.443341881	0.161035725
TNFRSF12A	0.869994491	1.530142157	0.376627488	6.216580298	0.552041253
TNFRSF13B	0.710386957	0.718268659	0.211646938	2.437596651	0.595570159
TNFRSF13C	0.728024399	0.010100852	1.09E-05	9.320190069	0.187116873
TNFRSF14	0.570459311	1.375829277	0.455363947	4.156908363	0.571698207
TNFRSF17	0.732528233	0.014754797	1.10E-06	198.3924323	0.384704254
TNFRSF18	0.618018021	0.03560702	8.49E-06	149.4169748	0.433264888
TNFRSF19	0.942781203	0.217526243	0.003272802	14.45784457	0.476203929
TNFRSF1A	0.776985576	0.741767228	0.298583659	1.842762001	0.519967807

TNFRSF1B	0.165107797	6.723087495	0.358282199	126.1572739	0.202728743
TNFRSF21	0.610509061	4.335617744	0.477865347	39.33656486	0.192341499
TNFRSF25	0.623045821	0.658530868	0.290456168	1.493040778	0.317189767
TNFRSF4	0.948914389	1.185857968	0.225753667	6.229175105	0.840371435
TNFRSF8	0.147128989	0.223124893	0.037882941	1.314172474	0.097320242
TNFRSF9	0.994814901	0.981632909	0.267100132	3.607647669	0.977730222
TNFSF10	0.684339559	0.794843217	0.371997907	1.698331434	0.553367231
TNFSF11	0.34413018	0.019892792	6.60E-05	5.996118414	0.178624527
TNFSF12	0.490064332	1.393188336	0.848703368	2.2869872	0.189769552
TNFSF12-TNFSF13	0.906653334	2.156052856	0.032448034	143.2618061	0.71972098
TNFSF13	0.691289448	0.949377379	0.296396363	3.040919259	0.930302452
TNFSF13B	0.104016159	1.762774149	0.933240953	3.329657459	0.08063095
TNFSF14	0.526447866	1.768970325	0.602774295	5.19142246	0.299080884
TNFSF15	0.994474104	0.574550455	0.166496019	1.982679393	0.380538657
TNFSF18	0.779102963	1.561376215	0.501073814	4.865342421	0.442272309
TNFSF4	0.382323135	0.611206518	0.241194905	1.548844524	0.299386671
TNFSF8	0.185354672	0.359290437	0.085297496	1.513404539	0.162957941
TNFSF9	0.882953825	1.878544301	0.895342393	3.941429244	0.095399707
TNIK	0.358943087	1.342539227	0.404410741	4.456883547	0.630405876
TNIP1	0.572853695	0.251954102	0.020909585	3.035969904	0.277704789
TNIP2	0.230794998	1.296337566	0.99151666	1.694869238	0.057739215
TNIP3	0.629178982	1.375085424	0.264909982	7.137745085	0.704637505
TNK1	0.817603016	4.467638726	0.003514986	5678.485173	0.681468936
TNK2	0.395944334	16.33235581	0.117261456	2274.79561	0.267439423
TNKS	0.312828921	0.61474391	0.111648248	3.384827618	0.57614253
TNKS1BP1	0.486818993	1.27145218	0.259727917	6.224169757	0.766954407
TNKS2	0.55111646	0.41382852	0.084895486	2.017233808	0.274965851
TNMD	0.54732908	0.853506007	0.657568211	1.107828042	0.233885583
TNN	0.999884715	0.896891997	0.495276527	1.624173989	0.719466779
TNNC1	0.745590659	0.844716718	0.056512149	12.62642372	0.90266561
TNNC2	0.197955033	0.000146222	6.78E-10	31.54583094	0.158783619
TNNI1	0.178395928	1.744181156	0.850182932	3.578250973	0.129196592
TNNI2	0.15954705	0.18418326	0.009337385	3.633080876	0.266132994

TNNI3	0.146218947	0.898345863	0.750913511	1.074724689	0.241174161
TNNI3K	0.363438813	0.81594145	0.361021472	1.844102083	0.624886498
TNNT1	0.785837436	0.976424597	2.46E-06	388110.7641	0.997106217
TNNT2	0.405065182	0.751348617	0.234325527	2.409147437	0.63058718
TNNT3	0.888559986	191.8042939	0.023113061	1591692.559	0.253579079
TNP1	0.383576286	0.84138241	0.060174075	11.76460725	0.897889632
TNP2	0.8215643	0.773629667	0.487584447	1.227485547	0.275835518
TNPO1	0.872749677	0.015986134	1.67E-06	152.891994	0.376464193
TNPO2	0.169905872	0.496755527	0.234481351	1.05239096	0.067753052
TNPO3	0.777310785	1.16239659	0.835994342	1.616238011	0.370891686
TNR	0.991255662	0.952369996	2.08E-05	43528.23106	0.992887559
TNRC6A	0.694923615	1.262634998	0.65295392	2.441592106	0.488246118
TNRC6B	0.607169767	0.957794927	0.81308941	1.128253683	0.605855385
TNRC6C	0.20992533	0.659464975	0.226955527	1.916208248	0.444283505
TNS1	0.867520742	0.957642559	0.216176956	4.242261926	0.954549973
TNS3	0.256661373	0.886375867	0.009326098	84.24340111	0.958603121
TNS4	0.70062529	1.058694046	0.909696014	1.23209629	0.461122711
TNXA	0.238250386	1.138530889	0.371385117	3.490319151	0.82043381
TNXB	0.314206627	0.764453227	0.456793817	1.27932716	0.306617073
TOB1	0.45127294	1.098720678	0.827837694	1.458241315	0.514509367
TOB2	0.227753718	1.033002838	0.713901867	1.49473606	0.863247311
TOE1	0.197759839	1.163844505	0.913186516	1.483304899	0.220161299
TOLLIP	0.234925155	1.190967353	0.73768899	1.922765901	0.474543701
TOM1	0.559272274	1.176145828	0.164586419	8.404818698	0.871543305
TOM1L1	0.838593879	0.232913875	0.025520453	2.1257018	0.196516701
TOM1L2	0.666651429	0.942735778	0.130278137	6.821948555	0.953431108
TOMM20	0.409421259	0.894987807	0.298039016	2.687578237	0.843236757
TOMM22	0.174588744	1.160504765	0.977900095	1.377207464	0.088358241
TOMM34	0.993275201	0.859253129	0.552189149	1.337070714	0.501338802
TOMM40	0.117767182	1.381878641	0.519346186	3.67690883	0.517125641
TOMM40L	0.221988153	0.000142515	3.61E-09	5.622436543	0.100971272
TOMM7	0.997454583	1.033711199	0.534609057	1.998766816	0.921493101
TOMM70A	0.835616887	0.02954836	0.000106524	8.196300426	0.219817234

TOP1	0.499999773	1.113038192	0.37599461	3.294871751	0.846640509
TOP1MT	0.572559522	1807.262877	0.032756602	99711170.64	0.178215065
TOP2A	0.74209267	3.76299612	0.095834403	147.7563307	0.479153813
TOP2B	0.671131223	1.05134382	0.864161977	1.279070195	0.616710014
TOP3A	0.965216803	0.392834078	0.000790469	195.2241036	0.768016608
TOP3B	0.615596184	0.937589008	0.744682929	1.180466362	0.583472631
TOPBP1	0.971618054	0.645463442	0.22483928	1.852981627	0.415853673
TOPORS	0.393511419	0.409430682	0.087155528	1.923383267	0.25792354
TOR1A	0.773283812	0.87880849	0.268417774	2.877247471	0.830944489
TOR1AIP1	0.789983852	1.04814082	0.384686423	2.855830392	0.926747367
TOR1AIP2	0.32610298	1.078777319	0.140008769	8.312054411	0.9419762
TOR1B	0.366609439	2.337239366	0.295837732	18.46514917	0.420795877
TOR2A	0.722250945	0.85155325	0.542421849	1.336861592	0.484979768
TOR3A	0.281065509	1.009293941	0.397460043	2.562960168	0.984477008
TOX	0.069654485	0.600560288	0.265040895	1.360818901	0.221799999
TP53	0.07625215	177.6582612	0.034411754	917199.9007	0.235023762
TP53BP1	0.166648992	0.553429784	0.223201389	1.372233958	0.201617219
TP53BP2	0.841369931	0.126517801	2.14E-05	748.3959895	0.640834525
TP53I11	0.0580514	1.304273077	0.579311916	2.936463436	0.521165458
TP53I13	0.106007257	1.637098092	0.534642353	5.012865417	0.38796676
TP53I3	0.357061922	0.821313242	0.323410322	2.085757305	0.678890254
TP53INP1	0.359513919	0.910747356	0.193130916	4.294810814	0.905949815
TP53INP2	0.844008189	0.018584519	4.29E-05	8.044219359	0.1981682
TP53RK	0.707159347	0.506620222	0.105666475	2.429001724	0.395179634
TP53TG3	0.883923331	0.451786286	0.024962346	8.176749311	0.59073934
TP73	0.405343145	0.712707023	0.271336819	1.872032337	0.491842833
TPBG	0.875610151	1.223567665	0.003376138	443.4409775	0.94649436
TPCN1	0.535460217	1.066551189	0.625613645	1.818265073	0.81287097
TPCN2	0.388309941	0.983519685	0.768478763	1.258734811	0.894976963
TPD52	0.233531727	1.84710288	0.291853456	11.69007589	0.514523372
TPD52L1	0.310202414	0.497095797	0.136473557	1.810638166	0.289232604
TPD52L2	0.15839716	186.1430072	0.917236738	37775.65564	0.053843399
TPD52L3	0.439086341	0.650960862	2.70E-05	15677.77899	0.933535399

TPH1	0.213050093	1.081522802	0.894135115	1.308182121	0.41949738
TPH2	0.243658527	0.23314205	0.000594908	91.36748109	0.63267626
TPI1	0.68974284	1.095415488	0.905252838	1.325524805	0.348875615
TPK1	0.384115709	0.089578806	0.000573495	13.99202515	0.349188735
TPM1	0.288478876	0.637897122	0.181403174	2.243140125	0.483461222
TPM2	0.350953389	0.652589567	0.219417797	1.94092343	0.442798701
TPM3	0.998918142	1.074048739	0.85849309	1.343727407	0.531961537
TPM4	0.679007694	1.481186814	0.404863403	5.418900208	0.552763677
TPMT	0.60332841	0.881808636	0.257436936	3.020493024	0.841299782
TPO	0.542791591	0.194503385	4.58E-05	826.6265278	0.700901154
TPP1	0.066609967	2.226436944	0.860990321	5.757348654	0.098698379
TPP2	0.848349797	0.37522443	0.055813937	2.522548659	0.3133354
TPPP	0.520915401	1.362688862	0.823211374	2.255703691	0.228810823
TPR	0.60180059	4.300512639	0.291040069	63.54592004	0.288392449
TPRKB	0.159486817	0.62746219	0.01339906	29.38331546	0.812280901
TPRX1	0.090360905	1.491805608	0.995723504	2.23504212	0.052478626
TPRXL	0.875952028	1.056287445	0.851260263	1.3106957	0.618937948
TPSAB1	0.414193805	0.000354763	6.34E-09	19.86627623	0.154410431
TPSD1	0.591819482	0.153640925	0.007075529	3.336221664	0.232964076
TPSG1	0.380392096	0.289837639	0.069993535	1.200194516	0.087589498
TPST1	0.690230555	1.348439598	0.638984791	2.845590968	0.432711405
TPST2	0.819893534	0.175564784	0.007137546	4.318429913	0.287012843
TPT1	0.377448708	1.043212064	0.536905053	2.026971816	0.900660091
TPTE	0.925039867	0.103786126	7.23E-05	149.0784658	0.541359761
TPTE2	0.380165012	0.72705341	0.354201565	1.492389399	0.384982749
TPX2	0.881439714	1.028408492	0.815380238	1.297093033	0.813015846
TRA2A	0.716998296	2.097128177	0.135464115	32.46576836	0.596240726
TRADD	0.091062719	0.189553616	0.026498505	1.355947201	0.097591687
TRAF1	0.617739459	0.947386481	0.500994652	1.791518414	0.86794462
TRAF2	0.738805456	0.984274201	0.789535871	1.227044569	0.887933847
TRAF3	0.805819601	1.263368177	0.860408496	1.855048104	0.232933944
TRAF3IP1	0.250310008	1.389987459	0.765201415	2.524910564	0.279588998
TRAF3IP2	0.082090352	0.22174235	0.020395915	2.410760698	0.21601428

TRAF3IP3	0.613577808	0.986363373	0.776955682	1.252211324	0.910214219
TRAF4	0.559772071	0.082975774	0.000315184	21.8443143	0.381354138
TRAF5	0.68127675	1.150401271	0.874655185	1.513079792	0.316294746
TRAF6	0.307417166	0.390319742	0.099505014	1.531073599	0.17730036
TRAF7	0.180444622	1.211332456	0.728166457	2.015097378	0.460319129
TRAFD1	0.118301025	0.421939194	0.113852706	1.563710599	0.19668024
TRAIP	0.236368451	0.092403929	0.000379574	22.4948904	0.395610528
TRAK1	0.924380469	0.002496441	8.78E-07	7.101875113	0.139712731
TRAK2	0.402161452	0.609311171	0.282750146	1.313032399	0.205967442
TRAM1	0.160783289	0.04882393	0.000537848	4.432061642	0.18928382
TRAM1L1	0.624420721	1.169969111	0.661299345	2.069906361	0.589697688
TRAM2	0.620923051	1.393802512	0.51836091	3.747746805	0.510578758
TRAP1	0.824035538	1.276921628	0.862406918	1.890672269	0.222183357
TRAPPC1	0.090389517	1.801359985	0.653462054	4.965701945	0.255295751
TRAPPC2	0.637666197	0.804398964	0.005972515	108.3392348	0.930663258
TRAPPC3	0.714170022	1.413396588	0.613717553	3.255064008	0.416272076
TRAPPC4	0.16326667	2.981340525	0.584008528	15.2196259	0.189070838
TRAPPC6A	0.974485091	0.679474757	0.255683292	1.805694619	0.438382498
TRAPPC6B	0.083207585	0.014203727	0.000145638	1.385256952	0.068681569
TRAT1	0.638054314	5.142651003	0.219979475	120.2242133	0.3085191
TRDN	0.316271225	40.64002641	0.001328402	1243307.582	0.482042167
TREH	0.345123559	15.24592172	0.008223374	28265.54286	0.477972668
TREM1	0.244554612	167.8819599	0.016867482	1670928.265	0.275365323
TREM2	0.779604934	0.917670562	0.000585122	1439.220246	0.981740789
TREML1	0.595772374	0.999805572	0.789448344	1.266214806	0.998712756
TREML2	0.786353724	1.046658221	0.520250268	2.105704694	0.898261012
TREML4	0.88154469	0.752669554	0.188316525	3.008293925	0.687731442
TRERF1	0.350835461	0.866927879	0.381976167	1.967567645	0.732737044
TREX1	0.648993638	1.088466488	0.48260407	2.45493018	0.838135626
TREX2	0.875377081	0.014349751	4.49E-06	45.85832464	0.302634086
TRH	0.929477021	0.406453982	0.031276642	5.282051714	0.49143287
TRHDE	0.860070699	0.728637318	0.238748501	2.223730572	0.578138423
TRHR	0.93755179	0.430383756	0.038894485	4.762376401	0.491827193

TRIAP1	0.242112856	0.001668236	5.25E-07	5.30459986	0.12007867
TRIB1	0.337893349	2.09939925	0.718952004	6.130419256	0.174949586
TRIB2	0.554842252	0.806277361	0.225964154	2.876930569	0.740059617
TRIB3	0.421093504	0.116597692	0.005887499	2.309133801	0.158352211
TRIM10	0.689929405	0.161971737	0.000705894	37.16539999	0.511592018
TRIM11	0.858279759	0.952093578	0.216984979	4.177626426	0.948123299
TRIM14	0.618297212	0.157992244	6.65E-05	375.4697683	0.641754702
TRIM15	0.783278623	1.265808035	0.392813279	4.078960839	0.692980088
TRIM16	0.495373066	11.86028732	0.021424824	6565.580773	0.442827873
TRIM17	0.437444931	5.11075434	0.003402135	7677.477024	0.662026301
TRIM2	0.652860417	1.137688484	0.485842635	2.664103547	0.766354876
TRIM21	0.748045558	0.335924545	1.92E-05	5879.970371	0.826778024
TRIM22	0.130836701	0.564853696	0.207807503	1.535361779	0.262901392
TRIM23	0.318994992	28.74376429	0.448146707	1843.6016	0.113671839
TRIM24	0.860526906	1.147827105	0.615167702	2.141703893	0.664844693
TRIM25	0.881110931	2.803615598	0.692871847	11.344465	0.14831804
TRIM26	0.544311635	1.434744354	0.420448312	4.895943935	0.56432515
TRIM28	0.146932955	0.467103921	0.119191746	1.830546832	0.274685755
TRIM29	0.242746519	1.080700927	0.509433802	2.292573616	0.8397137
TRIM3	0.132999936	0.003342228	3.45E-06	3.237636778	0.104145576
TRIM31	0.531354689	0.669598337	0.304023657	1.474760015	0.319445426
TRIM32	0.966272232	0.362051103	0.001915253	68.44057606	0.704040461
TRIM33	0.111952052	1.427101777	0.789340674	2.580152714	0.239175401
TRIM34	0.988272315	0.565224973	0.23480993	1.360586708	0.20303522
TRIM35	0.188517138	0.138076911	0.008626366	2.210111893	0.161682156
TRIM36	0.37796708	0.76292568	0.250501864	2.323557937	0.633922577
TRIM37	0.803855055	0.962030724	0.40549759	2.282388692	0.930021975
TRIM38	0.755280353	0.155604976	0.000971078	24.93406233	0.472595005
TRIM39	0.18404971	0.002343427	3.09E-08	177.63247	0.290775021
TRIM4	0.474225701	1.041179714	0.694478825	1.560962203	0.845144867
TRIM40	0.849591885	0.977721646	0.621476135	1.538175906	0.922366879
TRIM41	0.937716991	1.146768188	0.535592639	2.455368466	0.724419913
TRIM42	0.398832896	0.912621086	0.416663096	1.998922524	0.819203468

TRIM43	0.791795508	0.011466369	0.000102949	1.277113365	0.063134724
TRIM44	0.762588729	1.241493596	0.341188446	4.517463489	0.742728155
TRIM45	0.543928974	0.028829653	0.00016646	4.993079853	0.17749722
TRIM46	0.755690378	1.113686196	0.455956915	2.720206453	0.813185657
TRIM47	0.627463407	4.352627743	0.139731486	135.5841036	0.40187548
TRIM48	0.511086205	0.630646855	0.237093483	1.677462626	0.355688532
TRIM49	0.935936402	1.020532342	0.831166031	1.25304238	0.846112696
TRIM5	0.757949136	1.269427967	0.735209126	2.191821762	0.391934143
TRIM51	0.944705614	0.858496477	0.019520203	37.75658503	0.93700695
TRIM52	0.066825334	0.454868917	0.173198611	1.194615422	0.109819024
TRIM54	0.487678882	7.686549313	0.000381682	154796.3163	0.686695635
TRIM55	0.948262397	0.605993959	0.00165921	221.3273624	0.867859619
TRIM56	0.772595879	1.38454424	0.173131077	11.07232041	0.759048861
TRIM58	0.913061573	1.87714161	0.005185878	679.4723603	0.834057331
TRIM59	0.389974394	0.882750544	0.343122231	2.271052273	0.795889448
TRIM6	0.470013924	4.887707654	0.000317136	75329.43923	0.747067296
TRIM60	0.947877371	1.038970503	0.917288464	1.176794159	0.547480035
TRIM61	0.97273179	0.988412001	0.374512939	2.608610226	0.981219746
TRIM62	0.77477135	0.028295458	1.68E-06	475.383996	0.472641419
TRIM63	0.721930551	1.351381693	0.596146488	3.063395517	0.470807776
TRIM64	0.645228529	1.515550507	0.590092782	3.892427447	0.38762301
TRIM65	0.690301378	0.883185794	0.195746092	3.984841472	0.87163099
TRIM67	0.659351364	0.980037302	0.46320133	2.073554307	0.957942151
TRIM68	0.022887515	0.564785707	0.318436769	1.001715021	0.050689043
TRIM6-TRIM34	0.875976075	0.112301705	0.00082184	15.34566119	0.383472568
TRIM7	0.211363663	0.418086278	0.121442625	1.439331003	0.166790712
TRIM8	0.090786966	0.640380031	0.217179978	1.888233842	0.419184195
TRIM9	0.962917426	0.844062905	0.030502991	23.35646944	0.92028956
TRIO	0.725319678	1.34660464	0.396142588	4.577503436	0.633585115
TRIP10	0.544370382	1.028062454	0.036842814	28.68707087	0.98699869
TRIP11	0.677568721	0.135406474	2.47E-08	742776.2831	0.800619895
TRIP12	0.849087511	0.256191156	0.000240749	272.6238604	0.701755992
TRIP13	0.057455801	0.484797822	0.018598837	12.63675402	0.663410088

TRIP4	0.4065419	1.30074167	0.682731288	2.478176877	0.424006086
TRIP6	0.07122717	0.377040098	0.018263019	7.783994137	0.527733775
TRIT1	0.464251004	367.3539152	0.166658898	809731.1368	0.132641735
TRMT1	0.956092372	0.817975697	0.408955913	1.636079144	0.569986765
TRMT12	0.83164542	0.784804513	0.298447588	2.06373966	0.623264723
TRMT5	0.112884644	0.715456749	0.477726544	1.07148821	0.104187089
TRMU	0.582364389	1.248113326	0.855930746	1.819991724	0.249473922
TRNT1	0.929932679	0.419639178	3.06E-05	5755.189448	0.858204645
TRO	0.456019797	0.232837645	0.030145129	1.798412236	0.162330275
TROAP	0.679948683	1.025012753	0.12050957	8.718404216	0.981954255
TROVE2	0.962861212	0.828517748	0.36555165	1.87782399	0.652270956
TRPA1	0.530731555	0.013338019	9.68E-05	1.837354687	0.08581565
TRPC1	0.419492702	0.124187771	0.000135245	114.0345632	0.549001577
TRPC2	0.070319001	0.288211663	0.053883641	1.541580374	0.145922064
TRPC3	0.988761126	0.024326793	4.45E-08	13291.17784	0.581410885
TRPC4	0.073449786	0.854808552	0.663648061	1.101031862	0.2244758
TRPC4AP	0.913972123	0.881475147	0.015187141	51.16160114	0.951450192
TRPC5	0.761277368	9.66045643	0.009574065	9747.627262	0.520429029
TRPC6	0.582903112	0.007957676	3.59E-05	1.761604098	0.079354603
TRPC7	0.941405561	5.937947509	0.509106625	69.25704533	0.155224847
TRPM1	0.158131303	0.156448618	0.017859968	1.37044868	0.093865577
TRPM2	0.676087608	0.609624432	0.111027337	3.347301291	0.568971604
TRPM3	0.308943529	0.842107645	0.434919166	1.630522038	0.61022805
TRPM4	0.925129217	0.698135417	0.291945685	1.669464854	0.419191791
TRPM5	0.189750344	5.407866867	1.470544664	19.88720559	0.011073304
TRPM6	0.71831642	0.792949357	0.310235366	2.026747278	0.628004424
TRPM7	0.693688612	1.106077541	0.876344713	1.396034582	0.396017535
TRPM8	0.843144568	0.965736355	0.748150455	1.246603141	0.788952226
TRPS1	0.223611485	0.583733027	0.081524168	4.179671549	0.591982584
TRPT1	0.807209844	0.874920242	0.572696224	1.336634323	0.536573769
TRPV1	0.113005863	0.797475377	0.126019114	5.046591405	0.810017598
TRPV2	0.955421172	0.276807172	0.000152764	501.5717355	0.737201397
TRPV3	0.491989916	1.239645905	0.794302962	1.934679891	0.344181871

TRPV4	0.087439864	0.540201377	0.165997015	1.757968525	0.306363817
TRPV5	0.141663994	0.003140851	4.30E-06	2.292755082	0.086658165
TRPV6	0.640062875	1.004131706	0.139113149	7.247916454	0.99673785
TRRAP	0.590906371	0.796920377	0.341434049	1.860043217	0.599646591
TRUB1	0.124688844	22.19905035	0.144580225	3408.473304	0.227433421
TRUB2	0.708105478	768.5776863	0.005664826	104277113.8	0.270476262
TSC1	0.256163042	1.123440254	0.932848234	1.352972498	0.219778813
TSC2	0.394577042	0.746653914	0.003318936	167.9731184	0.915799337
TSC22D1	0.490768042	0.963234166	0.74479714	1.245735259	0.775286276
TSC22D2	0.799225514	0.375506969	0.064712886	2.178939829	0.274918037
TSC22D3	0.318659167	0.542013723	0.166578102	1.763610415	0.308942518
TSC22D4	0.375624095	0.380532414	3.85E-05	3759.93113	0.836890468
TSEN2	0.487038011	1.842650712	0.187287094	18.12918111	0.600306048
TSEN34	0.311568708	1.695622836	0.865806066	3.320763061	0.12361231
TSEN54	0.879619316	2.775059892	0.771332335	9.983968071	0.118170372
TSFM	0.52258716	169767.571	0.175783269	1.63958E+11	0.086766892
TSG101	0.657966735	1.005966368	0.647889556	1.561945741	0.978859347
TSGA10	0.886060554	0.503998785	0.055805866	4.551757604	0.541709194
TSGA10IP	0.820106564	0.803846697	0.092759851	6.966047316	0.842903206
TSGA13	0.077708515	1.311343309	0.897037029	1.917001439	0.161783213
TSHB	0.54038142	2.622915942	0.045420189	151.4676217	0.641245255
TSHR	0.885116213	5.092348713	5.91E-06	4387430.882	0.815419823
TSKS	0.947806989	1.013271104	0.775947763	1.323179704	0.92286039
TSLP	0.987319146	0.03177129	2.83E-06	357.2458295	0.468598668
TSN	0.804820162	3.917340434	0.011928427	1286.469336	0.644177911
TSNARE1	0.054457398	0.552656263	0.270473324	1.129238701	0.10382558
TSNAX	0.205442109	259.0268724	0.08883354	755288.1545	0.172193113
TSNAXIP1	0.90952315	0.294156535	0.000184009	470.2384056	0.745098005
TSPAN1	0.876461961	0.388235953	0.003452508	43.65729033	0.694560831
TSPAN10	0.482662343	1.341811382	0.683223334	2.635240478	0.393220859
TSPAN11	0.630828629	1.059100328	0.447252652	2.5079639	0.896131365
TSPAN12	0.124132205	0.508584334	0.197036308	1.31274295	0.162260256
TSPAN13	0.476752326	1.128155303	0.779429346	1.6329054	0.52273073

TSPAN14	0.804527995	0.713332121	0.272717535	1.865823245	0.491077854
TSPAN15	0.093114307	1.119690885	0.979937772	1.279374788	0.09650799
TSPAN16	0.21129861	0.825458614	0.075945411	8.971995955	0.874794768
TSPAN17	0.943306197	0.978527148	0.715603869	1.338052266	0.89185391
TSPAN18	0.092218143	1.32126843	0.55702514	3.134060094	0.527274284
TSPAN19	0.460303363	0.189558241	0.020781665	1.729039799	0.140350811
TSPAN2	0.938516253	0.810419695	0.212282857	3.093891295	0.758432534
TSPAN3	0.999458498	0.044560077	2.72E-05	72.88339537	0.409949671
TSPAN31	0.304581102	2.046977177	0.721815302	5.804969156	0.177979521
TSPAN32	0.423976754	14.97823056	0.757063028	296.3391188	0.075532129
TSPAN33	0.85389841	0.498494605	0.109426233	2.27090767	0.36820956
TSPAN4	0.918588485	0.983066252	0.518277108	1.864676717	0.958298298
TSPAN5	0.471222814	5.221812368	0.183666317	148.4612137	0.333170319
TSPAN6	0.310044822	1.61767975	0.477371517	5.481868278	0.439852944
TSPAN7	0.80190143	0.086014167	6.57E-05	112.5841111	0.502883011
TSPAN8	0.432547515	0.859323944	0.03420189	21.5905505	0.926561705
TSPAN9	0.855440982	0.597589226	0.254857448	1.401226001	0.23637273
TSPY1	0.17161354	0.607203291	0.346828082	1.063050702	0.080814203
TSPY2	0.919978465	652.6673089	0.010454486	40745629.62	0.249972381
TSPYL1	0.601097003	2.589353845	0.047672067	140.6432262	0.640652812
TSPYL2	0.092078972	2.199823912	0.998268947	4.847616725	0.050504492
TSPYL4	0.701406732	0.845212576	0.188784041	3.784134999	0.825962125
TSPYL5	0.489597831	0.015417198	1.10E-05	21.67666724	0.259250882
TSPYL6	0.595483197	0.785949792	0.300584554	2.055052623	0.62331598
TSR1	0.107669573	0.774722341	0.527879205	1.136992516	0.192216678
TSSC1	0.910210663	0.898648634	0.729044284	1.107709622	0.316639274
TSSC4	0.769624357	0.65591388	0.040294417	10.67698839	0.767016127
TSSK3	0.286352432	0.169939415	0.001397666	20.66259124	0.469320496
TSSK4	0.162965373	0.15111522	0.00010533	216.8022924	0.610366426
TSSK6	0.961729934	0.559927585	0.182527185	1.71765592	0.310550496
TST	0.680350923	1.008122502	0.474629009	2.141274469	0.983207572
TSTA3	0.576011405	3.163610047	0.006922253	1445.834065	0.712457192
TTBK1	0.901828351	0.142957644	2.55E-07	80231.52174	0.773345213

TTBK2	0.467155971	1.057849521	0.820025686	1.36464702	0.66513502
TTC1	0.599983325	0.745241617	0.001184334	468.9428989	0.928741785
TTC12	0.36546665	0.046182294	0.00087061	2.449781091	0.129078943
TTC13	0.87815644	0.934244639	0.508944449	1.714947569	0.826277777
TTC14	0.218856554	0.418757279	0.125400415	1.398381802	0.157092888
TTC16	0.65221925	1.109987949	0.185516199	6.641324353	0.908981279
TTC17	0.173338963	0.419054503	0.107000831	1.641171147	0.21177313
TTC19	0.085849674	0.861776961	0.149763066	4.958896397	0.867675113
TTC21A	0.775470183	0.487851793	0.118944144	2.000933913	0.31889244
TTC21B	0.370204302	1.061205409	0.52760841	2.134455968	0.867671975
TTC23	0.583953714	2.041411462	0.808733184	5.152948879	0.130888963
TTC25	0.796333359	2.819567185	0.052444027	151.5894092	0.610135141
TTC3	0.463177883	1.755622594	0.678980164	4.539470893	0.245565248
TTC4	0.085806669	1.992359924	0.561016903	7.075540946	0.286397042
TTC5	0.46281369	0.000927775	2.54E-08	33.92560611	0.192724179
TTC6	0.60816518	0.03555553	6.35E-06	198.9351374	0.448557358
TTC7A	0.153612618	0.00977383	6.08E-07	157.2403771	0.349014093
TTC7B	0.574186362	0.91539337	0.238948676	3.506799183	0.897355888
TTC8	0.59157331	1.439173794	0.201554988	10.27620914	0.716608374
TTC9B	0.880252147	1.467548953	0.377247	5.708991525	0.579957208
TTC9C	0.656506126	0.984787778	0.799860957	1.212469441	0.885142822
TTF1	0.994846052	36.51108147	0.231698904	5753.4112	0.163457798
TTF2	0.197478614	0.249563886	0.04557333	1.366635547	0.109614048
TTK	0.656306713	1.089409581	0.802273272	1.479312943	0.583272064
TTL	0.446351379	4.340338121	0.118924249	158.4078536	0.423814385
TTLL1	0.029939311	0.239915679	0.057016862	1.00951773	0.051529539
TTLL10	0.140211407	0.334852077	0.001864963	60.12232994	0.679511218
TTLL11	0.246613665	0.024434378	6.40E-06	93.28057347	0.377728389
TTLL12	0.124881048	0.001264826	7.17E-09	223.1159375	0.278981388
TTLL2	0.685019414	0.937780763	0.811393247	1.083855163	0.384443249
TTLL3	0.763208955	1.146718805	0.454162208	2.895362043	0.772040429
TTLL4	0.420437743	0.145830727	0.010825572	1.964478245	0.146762848
TTLL5	0.365721382	0.154236453	0.01174971	2.024635849	0.154740651

TTLL6	0.105890913	0.665761276	0.257609844	1.720578956	0.401030483
TTLL7	0.061979696	1.816924812	0.86113225	3.833575822	0.11699613
TTLL9	0.153405253	1.067362841	0.862290781	1.321205628	0.549257004
TTN	0.561379115	1.262006866	0.003804108	418.668769	0.937369167
TTPA	0.383847202	4.709911038	7.00E-05	316836.9021	0.784680469
TTR	0.393045952	0.206574406	0.000522019	81.74604686	0.605270491
TTTY1	0.963214379	0.653847514	0.109535593	3.902992262	0.641141541
TTTY10	0.882895892	0.84024597	0.402939129	1.752158675	0.642496965
TTTY11	0.629905559	4.478338809	0.273748928	73.26245478	0.293069715
TTTY12	0.529617381	0.994437833	0.778616195	1.270082242	0.964360097
TTTY13	0.507603058	0.871476855	0.618737879	1.227453394	0.431160729
TTTY14	0.391182541	1.032698322	0.887946592	1.201047263	0.676256043
TTTY15	0.277455187	9.08E-05	1.02E-09	8.114932569	0.109595604
TTTY16	0.344758676	17.31917732	0.080088052	3745.301546	0.298516841
TTTY18	0.244938542	0.965261581	0.816949826	1.14049834	0.677852876
TTTY19	0.641717702	0.971986965	0.37586797	2.513538624	0.953260558
TTTY20	0.344294908	1.111806723	0.783847279	1.576983454	0.552301323
TTTY23	0.277866067	0.480361175	0.191295734	1.206231069	0.118565113
TTTY4	0.940582022	1.112835544	0.845260114	1.465114617	0.44611338
TTTY5	0.238321522	2.296489616	0.764724304	6.896425975	0.138379261
TTTY6	0.134361892	0.052283381	0.000757357	3.609331809	0.171972963
TTTY6B	0.359695097	1.055519624	0.778724814	1.430700109	0.72767822
TTYH1	0.331911226	0.007246954	1.04E-05	5.070773359	0.140424059
TTYH2	0.344906697	1.26620403	0.587295054	2.729927037	0.547078697
TTYH3	0.932407198	0.306070983	0.000237158	395.0088053	0.745968253
TUB	0.278978456	1.893036321	0.750480812	4.775054141	0.176405739
TUBA8	0.856422407	1.49031038	0.004972812	446.6336391	0.890931314
TUBAL3	0.438415487	1.283413614	0.894282063	1.841869108	0.175812072
TUBB	0.760453138	1.772985479	0.538555762	5.836865436	0.346200345
TUBB1	0.985337817	0.793632526	0.398391546	1.580988835	0.510974691
TUBB2B	0.543108445	0.966786909	0.770177075	1.213587054	0.770913533
TUBB3	0.237703737	1.556348975	0.695546708	3.482472283	0.281724324
TUBB6	0.564753098	1.740442045	0.456636069	6.633594487	0.41695011

TUBB8	0.157352514	0.629767562	0.003100752	127.9067607	0.86457117
TUBD1	0.258263694	0.977856737	0.317650409	3.010239463	0.968865101
TUBE1	0.47616062	0.622050767	0.11516273	3.360003344	0.581185679
TUBG1	0.789047008	0.852758073	0.444617965	1.635553188	0.631689484
TUBG2	0.694857309	2.09035664	0.191963124	22.76265772	0.54502916
TUBGCP2	0.229629507	0.334937865	0.022101305	5.075871094	0.430309122
TUBGCP3	0.675809435	1.171842295	0.008980568	152.9095233	0.94912646
TUBGCP5	0.590580549	0.611525551	0.127470497	2.933725915	0.538747092
TUBGCP6	0.233252102	1.551113581	0.399766915	6.018390346	0.525712829
TUFM	0.488095269	1.315818042	0.428438935	4.041129269	0.631647355
TUFT1	0.078847892	0.860277213	0.369909167	2.000698956	0.726715094
TUG1	0.68724345	0.511303923	0.091157441	2.867914	0.445799815
TULP1	0.751772958	0.865799169	0.201176562	3.726120953	0.846552451
TULP2	0.111128558	1.09941411	0.897239035	1.34714534	0.360649557
TULP3	0.358085583	1.397393517	0.30289096	6.446903007	0.667978504
TULP4	0.092078972	1.591522292	0.428354126	5.913199037	0.487727436
TUSC1	0.467931493	0.796932356	0.507165566	1.252256112	0.324917559
TUSC2	0.199769794	1.186336535	0.780001821	1.80434755	0.424490703
TUSC3	0.311222638	4.27845255	0.006697206	2733.252624	0.659181799
TUSC5	0.779330642	1.808178145	2.02E-06	1617062.275	0.932487513
TWIST1	0.114893379	0.193446436	0.013358356	2.801357053	0.228355669
TWIST2	0.847730863	0.613981549	0.140890512	2.675647471	0.516015491
TWISTNB	0.93228408	0.00019675	1.45E-09	26.65696641	0.156945059
TWSG1	0.131531545	1.269066689	0.919928473	1.750712482	0.146626479
TXK	0.629076179	1.003512229	0.166575739	6.045518998	0.996946853
TXLNA	0.298086347	1.003965581	0.281688075	3.578237698	0.995130135
TXLNB	0.742723998	1.42835789	0.368208465	5.540899937	0.606229938
TXN	0.177313715	1.716685072	0.832333442	3.540657489	0.14344318
TXN2	0.749679607	0.017341567	2.64E-05	11.39381283	0.220603701
TXNDC11	0.20805251	0.877675285	0.382016062	2.016443762	0.758508094
TXNDC12	0.432001016	1.059715914	0.624470962	1.798318714	0.829801851
TXNDC2	0.177871389	0.828123232	0.664208335	1.032489432	0.093767257
TXNDC5	0.165447856	16.23687107	0.557142641	473.1929721	0.105233338

TXNDC8	0.309091531	0.784747125	0.346658834	1.776467207	0.560915363
TXNDC9	0.8789952	0.98219429	0.321267674	3.002809495	0.974862743
TXNIP	0.113441341	0.750700009	0.551277043	1.022263689	0.068729853
TXNL1	0.507677626	0.532163647	0.062545751	4.527855859	0.563634664
TXNL4A	0.341012405	2.316412514	0.411750272	13.03160507	0.340520796
TXNL4B	0.496538443	3.632635046	0.535820286	24.62773006	0.186503036
TXNRD1	0.774356282	0.852647073	0.027035531	26.89079925	0.927865955
TXNRD2	0.362039174	2.519419315	0.79333774	8.000972806	0.117046011
TYK2	0.145194678	0.828208043	0.357667791	1.917781191	0.659949991
TYMS	0.092792055	2.427809145	0.661906714	8.9049667	0.181002919
TYR	0.067295857	0.019106189	1.66E-07	2205.402118	0.505747524
TYRO3	0.539284322	1.194282917	0.625012268	2.2820539	0.590990222
TYROBP	0.167937934	0.829063429	0.386224128	1.779656214	0.630529134
TYRP1	0.541042784	0.563359905	0.001130856	280.6498044	0.856302095
TYSND1	0.524100478	0.178951497	0.017310652	1.849938305	0.148798161
U2AF1	0.438396935	1.075657109	0.914511268	1.265198426	0.378452155
U2AF1L4	0.35805465	9.478268331	0.000110765	811062.4951	0.697924298
U2AF2	0.382267028	0.599844362	0.245081091	1.468139614	0.263086492
UACA	0.785904713	0.647216324	0.061644279	6.795261129	0.716856276
UAP1	0.003468966	1.518662549	1.027749174	2.244064988	0.035961955
UAP1L1	0.863206392	1.097327543	0.801971025	1.501460403	0.56154502
UBA2	0.991611358	0.789435141	0.352096832	1.769989916	0.56600551
UBA52	0.773034391	0.975203832	0.523928818	1.815175042	0.936865779
UBAP1	0.838352871	0.826018621	0.413944076	1.648306624	0.587655913
UBAP2	0.902617579	0.668339414	0.286241198	1.56049365	0.351650236
UBAP2L	0.090360905	1.855195697	0.750521917	4.585810215	0.180759666
UBASH3A	0.715438302	2.418165006	0.010661013	548.495925	0.74967684
UBB	0.868678349	2.699965831	0.093283143	78.14719009	0.56295567
UBC	0.703912393	0.966359017	0.175528749	5.320209694	0.968635126
UBD	0.19891974	1.068286639	0.957551234	1.19182797	0.236775333
UBE2A	0.629087211	1.467447914	0.002494317	863.323797	0.906169853
UBE2B	0.463491526	22.37189292	0.000916232	546260.6066	0.546571254
UBE2C	0.718694404	5.571596216	1.04E-05	2976172.972	0.79851609

UBE2D1	0.986745838	0.901808185	0.31308105	2.59759574	0.848153464
UBE2D2	0.910754313	1.453717187	0.365327605	5.784653639	0.595462263
UBE2D3	0.525802283	1.191664814	0.835450509	1.699759608	0.333169358
UBE2D4	0.246187656	2.454660003	0.072535068	83.06817512	0.61723745
UBE2E1	0.620416049	0.797080234	0.258981833	2.453210294	0.692539805
UBE2E2	0.085118786	1.696971565	0.650033119	4.43010119	0.280061332
UBE2E3	0.910010659	0.576792864	0.066880874	4.974366919	0.616673703
UBE2F	0.57799918	1.765211575	0.644092526	4.837770628	0.269268292
UBE2G1	0.811367506	0.709557963	0.244080721	2.062729492	0.52857866
UBE2G2	0.91569631	0.161427491	0.00674041	3.866060983	0.260394618
UBE2H	0.335922399	1.085206652	0.882876842	1.333904596	0.437327708
UBE2I	0.502794071	0.303661813	0.034202567	2.69601101	0.284722832
UBE2J1	0.824987209	1.557513457	0.703439653	3.448551924	0.274584133
UBE2J2	0.214971507	0.011167201	6.21E-07	200.7633391	0.368534058
UBE2L3	0.000195536	0.425488322	0.206565374	0.876431073	0.02046515
UBE2L6	0.560030886	4.56664823	0.005861423	3557.886056	0.654813953
UBE2M	0.429574803	1.345896574	0.926939705	1.954212962	0.11846784
UBE2MP1	0.325918117	0.42684781	0.085676426	2.126594917	0.298777582
UBE2N	0.840892732	1.013912538	0.850660435	1.208494708	0.877411854
UBE2NL	0.782163136	0.752955954	0.091574566	6.191049461	0.791806363
UBE2O	0.800759358	1.05070777	0.488924131	2.257992087	0.899156479
UBE2Q1	0.104960936	6.029244104	0.044539163	816.1757453	0.473088517
UBE2Q2	0.962294232	0.135142923	4.76E-05	383.9521809	0.621798534
UBE2R2	0.744870631	1.231757505	0.349357049	4.342910941	0.745778508
UBE2S	0.736607417	2.42972603	5.35E-05	110438.4008	0.871110869
UBE2T	0.905310062	0.991210834	0.644130602	1.525310106	0.967979246
UBE2U	0.266898708	0.882147104	0.439538631	1.77045533	0.724238026
UBE2V1	0.363383282	1.054991149	0.88254792	1.261128488	0.556613212
UBE2V2	0.758966724	1.119574355	0.703242461	1.782382045	0.634022097
UBE2W	0.058605375	0.027325264	5.72E-05	13.06025233	0.25276938
UBE2Z	0.19455711	1.472875207	0.879784746	2.465786527	0.140802564
UBE3A	0.372201616	0.236901158	1.34E-06	42033.2301	0.815346792
UBE3B	0.23384629	1.154204459	0.951991081	1.399370182	0.144476998

UBE3C	0.470842036	0.676266594	0.198286215	2.306446295	0.532036532
UBE4A	0.807037608	1.072890586	0.407549214	2.824429957	0.886715027
UBE4B	0.811660848	0.56880529	0.223868683	1.445219821	0.235653788
UBIAD1	0.756113718	1.15028825	0.409806234	3.228752881	0.790324727
UBL3	0.451631146	0.058044113	5.85E-05	57.5740817	0.418736673
UBL4A	0.80840363	1.035306897	0.567257774	1.889547256	0.910002369
UBL4B	0.555927574	0.775592951	0.000578586	1039.681083	0.944854134
UBL5	0.916851823	1.231629308	0.988654333	1.534318621	0.063141736
UBL7	0.59863774	0.961963802	0.660857087	1.400263952	0.839571793
UBLCP1	0.876648745	1.223319438	0.321013857	4.661825072	0.767763465
UBN1	0.130376228	1.206743942	0.8039177	1.811417937	0.36451262
UBOX5	0.659741477	1.705298745	0.575505317	5.05302683	0.33552174
UBP1	0.652934511	0.902076291	0.464483443	1.751928182	0.760899084
UBQLN1	0.209426413	0.752549652	0.483605455	1.171059948	0.207648247
UBQLN2	0.913283083	0.742986018	0.103545549	5.331259806	0.767638854
UBQLN3	0.272816651	1.242018633	0.219705724	7.021256692	0.806273789
UBQLN4	0.993581898	1.343213175	0.280956216	6.421718155	0.711665257
UBR1	0.965290948	1.490562362	0.574881843	3.864752705	0.411572482
UBR2	0.880448063	1.820790892	0.608710733	5.446395623	0.283729358
UBTD1	0.420667088	0.366762508	0.092679394	1.45139854	0.152954808
UBTF	0.273760751	3.030262449	0.759921121	12.08347848	0.116195869
UCHL1	0.309918303	0.000139735	5.37E-10	36.367728	0.162984422
UCHL3	0.478998424	0.767269826	0.251466683	2.341077469	0.64160618
UCHL5	0.611009578	0.54877339	0.019164949	15.71369901	0.725890398
UCK1	0.551823932	0.723296053	0.193213464	2.707664204	0.630530969
UCK2	0.82984393	4.174200448	0.043475268	400.7784281	0.539498691
UCKL1	0.454758921	4.951307145	1.58E-05	1553685.417	0.804351414
UCN	0.716107193	96.88454544	0.020048745	468189.6625	0.290657485
UCN2	0.061395321	0.253047489	0.032190151	1.989211892	0.191473986
UCN3	0.053532048	0.661282186	0.217101962	2.014233887	0.466759182
UCP1	0.836008163	1.374096079	0.002121973	889.8041454	0.923343744
UCP2	0.322925041	0.676571356	0.352062147	1.300193171	0.241070376
UCP3	0.434268752	0.3800551	0.002211951	65.30066078	0.712546498

UFC1	0.14210327	1.766632586	0.677404173	4.60727999	0.244593027
UFD1L	0.721247834	0.777698368	0.425121819	1.422685744	0.414563065
UFM1	0.303491828	0.514771281	0.125544675	2.110718531	0.356350537
UGCG	0.959621944	0.408276834	1.10E-05	15184.08086	0.867498779
UGDH	0.684523511	2.77023289	0.277941601	27.61080106	0.385085467
UGP2	0.610285672	1.117560144	0.424379562	2.942980263	0.821992159
UGT1A1	0.717236962	0.35923702	0.107037071	1.205668608	0.097477651
UGT1A10	0.239800074	1.284508932	0.925242026	1.783277403	0.134711979
UGT1A3	0.090360905	3.368870105	0.447084726	25.38508949	0.238509047
UGT1A4	0.099793105	0.177707711	0.010623066	2.972779418	0.229378072
UGT1A5	0.555760147	0.697185957	0.159811418	3.041511457	0.631276916
UGT1A6	0.737665636	1.006715023	0.321919317	3.148227162	0.990820666
UGT1A7	0.518477232	0.702033125	0.419695289	1.174305553	0.177717888
UGT1A8	0.789326533	0.669492887	0.173003419	2.590820054	0.561145281
UGT1A9	0.8924548	0.046674943	0.000427486	5.096191821	0.20059682
UGT2A1	0.066641656	1.174036836	0.918391018	1.500844917	0.200359133
UGT2A3	0.374197834	7.55376053	0.002048133	27859.1759	0.629413996
UGT2B10	0.830149101	43.83317152	0.35152443	5465.756471	0.12469609
UGT2B11	0.761434816	1.257717853	0.592220396	2.671056603	0.55070964
UGT2B15	0.315267156	0.875978936	0.752486463	1.019738074	0.087661826
UGT2B17	0.914133871	0.283439102	7.27E-06	11045.63004	0.815166243
UGT2B28	0.874581215	0.9807231	0.288404834	3.334957276	0.975133185
UGT2B4	0.297332252	1.414703321	0.206319126	9.700436062	0.723957298
UGT2B7	0.431380361	0.483949099	0.142984998	1.637981147	0.243329357
UGT3A1	0.671345676	0.909658467	0.664182558	1.245860068	0.55514996
UGT3A2	0.326403042	0.807873337	0.256374839	2.545723013	0.715616028
UGT8	0.459058996	11.59607885	0.00031089	432529.0038	0.648183233
UHMK1	0.156264944	6.332341465	0.519170412	77.23581217	0.148096307
UHRF1	0.943622627	0.105433357	1.24E-05	899.1555778	0.626150058
UHRF2	0.005393576	2.731663526	1.576974661	4.731836092	0.000337123
ULBP1	0.452063294	318.6338613	0.044621217	2275319.766	0.202968543
ULBP2	0.93102779	0.621461394	0.000238125	1621.900237	0.905663951
ULBP3	0.095866634	1.577286937	0.910040813	2.733760997	0.104370696

ULK1	0.29606405	0.014650183	1.53E-05	13.9946994	0.227706729
ULK2	0.091240738	0.00690634	1.53E-05	3.123434732	0.110741046
ULK3	0.463213135	0.611481739	0.131845291	2.835974745	0.529775468
UMOD	0.869174427	1.126746198	0.430031586	2.952241272	0.808145889
UMODL1	0.278061515	0.624733593	0.225439413	1.731250344	0.365682188
UMPS	0.603735972	1.362662027	0.706298863	2.628983137	0.356058211
UNC119	0.515588783	0.989902745	0.411256132	2.382718141	0.981933726
UNC13A	0.968209014	0.68106523	0.170940589	2.713514966	0.58603204
UNC13B	0.110539874	1.369021123	0.895915783	2.091958718	0.146526674
UNC13C	0.633583779	1.1181814	0.783398031	1.596033679	0.538356143
UNC13D	0.122618205	0.230972673	0.023785918	2.242855489	0.206402385
UNC45A	0.613517064	1.206357416	0.082673398	17.60298059	0.890890296
UNC45B	0.107395683	0.876506379	0.762284585	1.007843327	0.064271291
UNC50	0.202713987	0.511492919	0.138816432	1.884683262	0.31368005
UNC5A	0.052489088	0.723528948	0.471291475	1.110765132	0.138966179
UNC5B	0.849830889	0.685720159	0.217619574	2.160706996	0.5193868
UNC5C	0.822665309	1.007818619	0.616201485	1.648321846	0.975247557
UNC5CL	0.081169315	0.934351844	0.765755474	1.140068073	0.503619075
UNC5D	0.246628563	1.059833176	0.90889135	1.235842284	0.458502765
UNC93A	0.798655103	0.000434621	1.45E-07	1.298377412	0.057958841
UNC93B1	0.200856304	0.530268411	0.203889505	1.379102801	0.193314414
UNG	0.944974157	0.382412496	0.009388364	15.57665634	0.611291535
UNKL	0.706986417	1.074707803	0.760920553	1.517894156	0.682549124
UPB1	0.686524545	1.0016125	0.765327877	1.310846804	0.990635696
UPF2	0.839355051	1.121405674	0.73758544	1.704955951	0.59192902
UPF3A	0.913535071	0.039306016	0.000341545	4.523457664	0.18134346
UPF3B	0.951645413	1.069947981	0.401845674	2.848826687	0.892363127
UPK1A	0.735545599	5589.645605	0.30485711	102487811.3	0.084927001
UPK1B	0.446070938	1.353486184	0.024106038	75.99444029	0.882909331
UPK2	0.874919699	1.021349548	0.787339916	1.324910471	0.873581267
UPK3A	0.211206565	0.351658489	0.105081364	1.176837535	0.089931925
UPK3B	0.539618595	0.309632838	0.04025898	2.381394015	0.260020667
UPP1	0.917009651	1.020071028	0.754894927	1.378396999	0.897059079

UPP2	0.171367207	1.002414826	0.456065661	2.203269329	0.99521061
UQCRB	0.52452175	1.00565281	0.480515536	2.104692769	0.98806446
UQCRC1	0.172560968	0.392783167	0.020677764	7.46108814	0.533877127
UQCRC2	0.439198361	0.688711102	0.27158137	1.746522535	0.432172388
UQCRFS1	0.142219606	0.846429072	0.625819451	1.144806499	0.279168666
UQCRH	0.337710279	2.149987055	0.508425944	9.091676744	0.298113068
UROC1	0.712872921	0.889551953	0.498611811	1.587011497	0.691915715
UROD	0.515947377	3.263848591	0.402690907	26.45380725	0.267867404
UROS	0.384241683	76.23037653	0.124275356	46759.63511	0.185749602
USF1	0.955931969	1.186129496	0.766297419	1.835975363	0.443803496
USF2	0.652241857	0.762772373	0.201677239	2.884915014	0.689913402
USH1C	0.365222717	0.022232278	1.19E-05	41.5143127	0.321972423
USH1G	0.284262069	0.586441579	0.204253256	1.683761284	0.321325534
USH2A	0.400486197	12.34325144	0.002373237	64197.48016	0.564852438
USHBP1	0.600663303	1.117545856	0.249772936	5.000176393	0.88441488
USMG5	0.07312101	2.267226683	0.81678549	6.293349841	0.116079785
USP1	0.911229757	1.875171587	0.000599576	5864.590943	0.878311308
USP10	0.239622466	1.670830518	0.635404384	4.393540066	0.298050102
USP11	0.302838085	1.645405135	0.649045716	4.171290234	0.294071553
USP12	0.713808178	0.035046873	2.64E-07	4655.608416	0.577695235
USP13	0.237393996	0.833225357	0.547197979	1.2687629	0.395089503
USP14	0.969226976	0.640000018	0.211414072	1.937430276	0.429705244
USP15	0.692159053	0.700578122	0.158860543	3.089563314	0.638341152
USP16	0.963990961	0.967811958	0.7899224	1.185762027	0.752209998
USP18	0.29529825	16.90971534	0.00099107	288514.8807	0.569504199
USP19	0.381711964	0.002922714	4.43E-06	1.928770937	0.078128131
USP2	0.61972601	0.748319404	0.119574687	4.683114323	0.756669684
USP20	0.935508168	1.062710941	0.750703744	1.504394445	0.731608067
USP21	0.730966435	0.966845666	0.300727223	3.108433396	0.954875125
USP22	0.560857526	0.994654509	0.784776466	1.260661648	0.964644669
USP24	0.372474581	1.02002496	0.811563464	1.282032725	0.86502722
USP25	0.302316012	0.955010631	0.253007924	3.604809248	0.945846684
USP26	0.830646472	1.690550903	0.004987797	572.9908916	0.859789043

USP27X	0.238547436	1.429937482	0.379013498	5.394850615	0.597573851
USP28	0.873183621	0.012026749	1.22E-06	119.036264	0.346314378
USP29	0.863350391	0.913666296	0.512045572	1.630296494	0.759900429
USP3	0.213914659	2.328853117	0.684946347	7.918221422	0.175764074
USP30	0.748700829	0.809898259	0.402173462	1.630975815	0.554963695
USP31	0.562689192	0.771449893	0.520026543	1.144431847	0.197215408
USP32	0.871052418	0.947769966	0.158716812	5.659563709	0.953082784
USP33	0.250220971	5.066596122	0.418676598	61.31318633	0.202112992
USP34	0.076175107	0.854825317	0.71234136	1.025809204	0.091783948
USP35	0.392438908	1.276899726	0.881187863	1.850312491	0.196491702
USP36	0.88845475	0.452798982	0.000223723	916.433338	0.838365536
USP37	0.296589858	0.926320751	0.659035022	1.302009917	0.659490433
USP38	0.060226985	0.142904919	0.018360051	1.112296234	0.063124867
USP39	0.743664207	1.25565036	0.597967864	2.636693242	0.547545604
USP4	0.402953417	2.732461696	0.701414856	10.64469459	0.1473938
USP40	0.064059981	0.422720368	0.104400193	1.711610902	0.227528156
USP41	0.988978677	1.051151313	0.407782688	2.709578207	0.91775892
USP42	0.765602097	1.123177422	0.442680062	2.849750033	0.806821868
USP44	0.546873963	13.14926978	0.044630415	3874.113562	0.374476597
USP46	0.376795359	0.168098417	0.003912535	7.222191926	0.352663034
USP47	0.306431062	1.069359791	0.009798822	116.7008001	0.977654677
USP48	0.196044531	0.60495668	0.264537477	1.383443244	0.233695982
USP49	0.635241801	0.816590741	0.528658623	1.261344108	0.361055657
USP5	0.719130412	0.57146513	0.088153571	3.704584995	0.557373976
USP50	0.957980833	0.021560234	1.62E-06	287.6704019	0.428532043
USP51	0.903803302	2.41295281	0.201172881	28.94197877	0.487118468
USP53	0.211441494	0.073885101	0.000716948	7.614231775	0.270637611
USP54	0.593764046	1.033831853	0.804465378	1.32859453	0.794890411
USP6	0.195103537	0.634061185	0.285334371	1.40899109	0.263420448
USP6NL	0.053475067	6.727701976	0.260665574	173.6400136	0.250424495
USP7	0.837378213	0.633836078	0.003938326	102.009882	0.860384749
USP8	0.538993231	0.953076445	0.154526742	5.878301046	0.958707763
USP9X	0.121546071	1.487960313	0.743835013	2.976501315	0.261266892

USP9Y	0.258614289	0.050764123	6.86E-05	37.55217946	0.376546331
USPL1	0.551553715	0.708144905	0.000120744	4153.144734	0.937863658
UST	0.860788538	1221.922182	0.000599914	2488845969	0.337542229
UTF1	0.758111351	0.561700293	0.241060798	1.308828405	0.181420503
UTP11L	0.540097527	1.0677846	0.904492491	1.26055657	0.438618843
UTP14A	0.314415341	0.725501081	0.245414968	2.144742124	0.561747156
UTP14C	0.662937525	239.0366544	0.150655629	379265.7647	0.145236593
UTP15	0.75125133	0.472990768	0.074044108	3.021445918	0.428773302
UTP20	0.123802389	0.019561166	0.000230336	1.661221797	0.082563496
UTRN	0.179414699	0.826686764	0.472133102	1.447496484	0.505446511
UTS2	0.86734412	1.071029846	0.808142704	1.419433628	0.632975238
UTS2R	0.349627289	3702.315114	0.035845893	382390728.7	0.163045798
UTY	0.971463801	0.971478622	0.684745287	1.378279968	0.871192316
UVRAG	0.35541671	1.117666858	0.953522853	1.3100674	0.169845961
UXS1	0.74897578	2.757339713	0.003328506	2284.184834	0.767348959
UXT	0.521394078	1.29045712	0.982259503	1.695356038	0.067039892
VAC14	0.666836016	1.753507298	0.203461092	15.11241194	0.609315668
VAMP1	0.717613474	1.685677418	0.706825941	4.02009631	0.238987227
VAMP2	0.211172577	3.027191581	0.915566382	10.00898356	0.069464597
VAMP3	0.404145144	1.10332133	0.56360759	2.159867925	0.774192945
VAMP4	0.278123697	0.135482267	0.005752453	3.190890032	0.21493154
VAMP5	0.793255719	0.917910386	0.450316345	1.871039073	0.813634856
VAMP8	0.900641119	1.696279035	0.490294671	5.868639283	0.404022851
VANGL1	0.191985294	0.521521811	0.203302726	1.337832523	0.175600165
VANGL2	0.677927061	1.010293005	0.925431569	1.102936176	0.819051634
VAPA	0.406764395	0.501945239	0.139386276	1.807559755	0.291702837
VAPB	0.785839606	1.150969919	0.59455293	2.22811408	0.676533183
VARS	0.199537208	0.881764489	0.516180941	1.506271448	0.64510381
VASH1	0.214686381	1.738524548	0.381622988	7.920035477	0.474716798
VASP	0.558310817	0.589011035	0.209340139	1.657274142	0.315934905
VAT1	0.425531924	5.689582553	0.001729531	18716.83429	0.67391923
VAV1	0.148608847	0.726393111	0.278971936	1.891397962	0.512664361
VAV2	0.452702505	1.043030728	0.673569335	1.615146419	0.850225842

VAV3	0.70329711	1.257048396	0.588842288	2.683521041	0.554360303
VAX1	0.157579568	0.168651737	0.000278525	102.1216171	0.586047481
VAX2	0.937616988	0.982408183	0.002124225	454.3426393	0.9954771
VBP1	0.094361057	1.581149183	0.68989188	3.62380369	0.27894168
VCAM1	0.16012769	0.532682203	0.189725201	1.495585864	0.231788767
VCL	0.080600574	120.2769385	0.075915003	190562.3584	0.202611636
VCP	0.571764125	2.419180234	3.10E-05	188765.8446	0.877840437
VCPIP1	0.587302419	1.707683646	0.322933367	9.030294581	0.528844176
VCX	0.050535464	0.47575258	0.213628942	1.059503059	0.068991888
VCX2	0.494124328	0.892375278	0.357138282	2.229762746	0.807457606
VCX3A	0.373296306	21.92124175	0.042200665	11387.04434	0.333154911
VCY	0.55168089	1.071561266	0.889785094	1.290472895	0.466162984
VCY1B	0.069420558	1.473045647	0.886813314	2.446809768	0.134649825
VDAC1	0.508958279	1.093099932	0.824599139	1.449028268	0.535938797
VDAC2	0.136394189	2.006372587	0.075947703	53.00398575	0.676789118
VDAC3	0.985045097	1.020487596	0.746557624	1.394929072	0.89880457
VDR	0.293968112	1.314612262	0.659728374	2.619571125	0.436803979
VEGFB	0.54469457	1.291901629	0.286306245	5.829456564	0.739029356
VEGFC	0.318013006	0.599044586	0.24330367	1.474923977	0.265002823
VENTX	0.264957417	1.039953137	0.835814189	1.293950906	0.725311211
VENTXP7	0.096865283	1.668984274	0.815979226	3.413700273	0.16063251
VEPH1	0.585605944	3.405493644	0.005262218	2203.897291	0.71059286
VEZT	0.461017861	1.236730272	0.876211991	1.745584153	0.226894702
VGF	0.140312936	2.099162573	0.827249073	5.32667083	0.118573332
VGLL1	0.434964692	1.457396522	0.488587817	4.347232063	0.49937093
VGLL2	0.722920954	0.400760844	8.59E-06	18703.01187	0.867605454
VGLL3	0.970866425	0.753357764	0.097460212	5.823380729	0.786063762
VGLL4	0.983639369	0.185934184	0.021101728	1.638326506	0.12969414
VHL	0.465005916	0.804582089	0.392087381	1.651040992	0.553285111
VHLL	0.079183013	0.051289822	5.75E-05	45.76784415	0.391503174
VIL1	0.147449561	1.240986594	0.687269705	2.240820039	0.473928895
VILL	0.673421639	1.06055551	0.828836305	1.357056855	0.640196744
VIM	0.089509306	1.545454118	0.63784175	3.744547028	0.334998499

VIP	0.058273416	3.860402715	0.461113962	32.31892842	0.212788399
VIPR1	0.30282892	2.388123564	1.26E-05	450848.8172	0.888309377
VIPR2	0.544079911	0.728401713	0.191367765	2.772510066	0.642160368
VIT	0.404511996	0.954689468	0.759808568	1.199554755	0.690595734
VKORC1	0.912202361	1.055940265	0.372166754	2.99599529	0.918517812
VKORC1L1	0.609604153	0.960010209	0.847709072	1.087188556	0.520247733
VLDLR	0.442565096	1.040437367	0.268807822	4.027077436	0.954220606
VMAC	0.319784104	1.506996662	0.42876779	5.296664054	0.52250137
VMO1	0.628932759	0.356035534	0.073525125	1.724054203	0.199426609
VN1R1	0.40389199	1.042472184	0.489111292	2.221883386	0.914211016
VN1R2	0.062392308	1.093191223	0.971420789	1.230225937	0.13920816
VN1R4	0.131374315	2.881832315	0.88855312	9.346607762	0.077878375
VN1R5	0.597829079	1.076910613	0.271420516	4.272840118	0.916078075
VNN1	0.807400521	0.900978295	0.581593711	1.39575424	0.640559745
VNN2	0.758931956	1.270230061	0.610365296	2.643473374	0.522379935
VNN3	0.679181621	1.855439435	0.467239456	7.368075302	0.379667016
VPRBP	0.296377254	0.96285555	0.195164503	4.750304465	0.962925966
VPREB1	0.477946354	0.264052883	0.06380922	1.092693579	0.066116692
VPREB3	0.786306915	1.155373284	0.950795099	1.403969612	0.146360689
VPS11	0.684619269	1.054311539	0.393285808	2.826374095	0.916281386
VPS13A	0.354787692	1.182967228	0.388754076	3.599734505	0.767281036
VPS13B	0.299826678	0.417145695	0.035785126	4.862649639	0.48532624
VPS13C	0.847379601	1.27529669	0.289011275	5.627398609	0.748155553
VPS13D	0.205377565	18.08889885	0.196638525	1664.00893	0.20948165
VPS16	0.846221944	1.220095471	0.346085246	4.301347638	0.756987017
VPS18	0.201304167	0.049501492	0.001850484	1.324193127	0.073052162
VPS25	0.742449799	0.77731257	0.206419098	2.927126602	0.709616354
VPS26B	0.603320168	0.759031303	0.298805615	1.928104729	0.562145228
VPS28	0.57816806	1.31093652	0.558516614	3.0769981	0.533983604
VPS29	0.926169078	0.072527858	0.001887313	2.787184106	0.158726526
VPS33A	0.576920905	0.666816646	0.137289352	3.238739446	0.61527452
VPS33B	0.808540069	1.16187678	0.669682844	2.015816389	0.593543781
VPS35	0.252489849	0.664726422	0.367234475	1.203212788	0.177365854

VPS36	0.962705039	1.128342218	0.143411976	8.877613997	0.908658549
VPS37A	0.253722128	0.208642489	4.78E-06	9115.375493	0.773756032
VPS37B	0.698027234	0.697373364	0.395354698	1.230109599	0.213226327
VPS37C	0.06500497	0.59415867	0.184243602	1.916074805	0.383506328
VPS37D	0.053015642	1.623087076	0.879160518	2.996508147	0.121558301
VPS39	0.807112051	1.261874928	0.422345006	3.770207556	0.67703571
VPS41	0.483095616	0.287052147	1.57E-05	5262.685076	0.803210386
VPS4A	0.715048631	0.847468084	0.317031242	2.265398664	0.741473189
VPS4B	0.516493581	1.487379742	0.295265787	7.492566336	0.63033611
VPS52	0.386463247	0.820256379	0.265499	2.534173486	0.730639637
VPS53	0.579521756	0.44254021	0.013083324	14.9688132	0.64999481
VPS54	0.353184401	2.226113351	0.795720563	6.227790109	0.127353787
VPS72	0.255724958	0.994483039	0.456611817	2.165945947	0.988885809
VRK1	0.170277738	1.079886648	0.763655509	1.527069679	0.663750956
VRK2	0.834289073	0.483940333	0.22532961	1.039358501	0.062746772
VRK3	0.167395926	1.857748813	0.316177299	10.91549162	0.493014747
VSIG1	0.424355485	0.11562241	0.004483856	2.98148333	0.193213585
VSIG2	0.960811373	0.319261474	7.76E-06	13133.54703	0.833183012
VSIG4	0.709019213	1.255757463	0.259368024	6.07988132	0.777177104
VSIG8	0.62893192	55.53313076	0.284764246	10829.76059	0.135415491
VSNL1	0.966006969	0.232854754	0.008980674	6.037557619	0.380252833
VSX1	0.434270029	1.049725149	0.746224628	1.476663791	0.780462623
VTCN1	0.458293463	0.009538134	2.54E-07	357.8152479	0.386618973
VTI1A	0.958050112	1.738326418	0.397383406	7.604189533	0.462746879
VTI1B	0.787607867	0.918356162	0.405753454	2.078548025	0.838071446
VTN	0.668883825	0.105174122	0.000107359	103.0340187	0.521577781
VWA1	0.91762745	1.81014595	0.003023103	1083.862606	0.855682214
VWA2	0.772371468	4.183004407	0.027659973	632.5937513	0.576262584
VWCE	0.95158624	1.17754482	5.86E-06	236744.5068	0.979072742
VWF	0.290243856	1.085980856	0.651874627	1.809173685	0.751433438
WAC	0.859311887	0.083250874	3.44E-06	2014.830541	0.629322564
WARS	0.297085863	0.800378518	0.553353069	1.157679983	0.23702989
WAS	0.077708515	1.919117171	0.884779399	4.162631636	0.098925287

WASF1	0.135320754	0.79863949	0.532424253	1.1979639	0.277097258
WASF2	0.843667056	0.529127678	3.76E-05	7447.315792	0.896086912
WASF3	0.68676947	0.545260701	0.16630692	1.787714139	0.31679239
WASL	0.311269993	0.015775778	1.54E-06	161.9879396	0.378621637
WBP1	0.057269697	1.529544876	0.853603139	2.740743819	0.153275919
WBP11	0.076196155	1.136818207	0.108198231	11.94433238	0.914901586
WBP2	0.116352661	3.578609327	0.54498452	23.49873116	0.184240497
WBP4	0.851721146	6.792513428	0.160240999	287.9302983	0.316272826
WBP5	0.247382441	2.280347536	0.831316986	6.255116848	0.109348673
WBSCR16	0.145356301	0.414636865	0.008328394	20.64308317	0.658815009
WBSCR17	0.73049808	1.138505728	0.793367393	1.633789469	0.481492534
WBSCR22	0.5715745	1.658811879	0.27059879	10.16876997	0.584337137
WBSCR27	0.801063786	0.067373908	9.81E-05	46.28128758	0.418302117
WBSCR28	0.140106392	0.730519731	0.498496599	1.070537048	0.107312166
WDFY1	0.897900076	1.00736884	0.888215481	1.142506521	0.908991724
WDFY2	0.767992319	0.959612913	0.388544756	2.370015114	0.928788975
WDFY3	0.725160472	0.442760772	0.015272573	12.83589206	0.635311692
WDHD1	0.660198427	0.117069208	0.000102591	133.5909255	0.550378496
WDR1	0.662144366	2.329477167	0.006631786	818.2507247	0.777357327
WDR12	0.795993761	1.206610173	0.228180847	6.380500936	0.825069024
WDR13	0.805584323	1.247285065	0.378451054	4.110756246	0.716502013
WDR17	0.215239823	46.97304622	0.040140267	54968.91804	0.285542187
WDR18	0.853495966	6.033879011	0.005243437	6943.479365	0.617201385
WDR19	0.675416438	2.509609373	0.392909285	16.02949956	0.330773517
WDR20	0.346179606	1.025978522	0.772049333	1.363425733	0.859684535
WDR24	0.327150927	0.878741611	0.423552407	1.823119892	0.728479941
WDR25	0.437421313	1.31790154	0.600870073	2.890582422	0.490921519
WDR26	0.759771218	0.291194037	0.013875365	6.111116353	0.426947002
WDR27	0.868659961	0.311650373	0.002270899	42.76982048	0.642444235
WDR3	0.40419527	0.838360444	0.362768295	1.93745772	0.679963281
WDR31	0.450597878	0.877487585	0.673166771	1.143824226	0.33386449
WDR33	0.126180886	0.285070582	0.044009031	1.846558192	0.187984944
WDR34	0.871379532	0.231193789	8.16E-06	6548.282942	0.7794807

WDR35	0.441806539	3.754001936	0.045961593	306.6153624	0.555944789
WDR36	0.638715955	3.213862175	0.006153904	1678.432024	0.714636255
WDR37	0.685903962	0.998324162	0.694998659	1.434033171	0.992757837
WDR4	0.393304323	0.960844684	0.768670998	1.201063275	0.725721783
WDR41	0.336453825	1.057488413	0.850405129	1.314998822	0.615183321
WDR43	0.949618612	3.734143345	0.304547794	45.78534728	0.302888354
WDR44	0.11602227	798.9879658	0.828374984	770643.4667	0.056616597
WDR45	0.737754422	1.326167505	0.36288479	4.846497564	0.669430876
WDR46	0.369886902	0.004969267	2.92E-06	8.470003957	0.162352997
WDR47	0.765011008	0.623901662	0.169761815	2.292937806	0.477464001
WDR48	0.421775825	0.131957748	0.006927912	2.513433579	0.177984882
WDR49	0.347666201	4.083131376	0.923567938	18.05168971	0.063579193
WDR5	0.204380619	0.671587797	0.415284032	1.086076358	0.104529241
WDR53	0.022466366	0.316787857	0.113376951	0.885140634	0.02832948
WDR54	0.459705625	0.868816582	0.584402304	1.291648318	0.487024458
WDR55	0.437731957	0.240431257	0.008111408	7.126652503	0.409787365
WDR59	0.082306076	5.536414632	0.872936007	35.11355556	0.069404375
WDR5B	0.438906996	0.001803532	2.18E-07	14.92031525	0.169834949
WDR6	0.937019665	0.754541502	0.294243625	1.934903022	0.557749529
WDR60	0.771452405	1.607520206	0.616674165	4.190415878	0.331517843
WDR61	0.291059809	0.854104559	0.030504244	23.91452769	0.926095223
WDR62	0.451855987	0.140284264	0.003204065	6.142095069	0.308394113
WDR63	0.62594206	0.545623287	0.233463042	1.275168731	0.161890708
WDR64	0.819640295	2.502976661	0.037234004	168.2572775	0.669135752
WDR66	0.80844391	0.462446894	0.00110836	192.9490913	0.802182625
WDR7	0.313853795	1.282646536	0.684024103	2.40515229	0.437726937
WDR70	0.771902206	1.316288891	0.379920597	4.560469892	0.664675552
WDR72	0.891021973	0.76216548	0.17735635	3.275305452	0.715040382
WDR73	0.635448369	0.183643055	0.000124865	270.0896334	0.648801344
WDR74	0.920067816	0.620003236	0.26716885	1.438805507	0.26573367
WDR75	0.027433692	2.612816082	1.151120235	5.930577596	0.021648671
WDR76	0.493854513	0.976615251	0.904691053	1.054257523	0.544348822
WDR77	0.572853481	1.88571749	0.627230236	5.669258666	0.258715898

WDR78	0.554431782	0.017793732	7.10E-07	446.1184358	0.435651731
WDR81	0.143209138	14.35096387	0.326583559	630.6201218	0.167537639
WDSUB1	0.062883192	1.140589905	0.954802624	1.362528021	0.147027011
WDTC1	0.157196	1.636544297	0.160525308	16.6844547	0.677553062
WEE1	0.088918937	6518.686287	0.287954261	147569516	0.086047919
WFDC1	0.554298562	0.989356248	0.303742795	3.222548166	0.985829722
WFDC10A	0.873521512	1.540417983	0.096664889	24.54756423	0.759706161
WFDC10B	0.235296556	1.109833067	0.139783919	8.811667643	0.921470818
WFDC11	0.172798379	1.311977038	0.88360049	1.948033945	0.178184256
WFDC12	0.63177908	0.981188317	0.288226782	3.340184097	0.975760468
WFDC13	0.443407077	1.73419804	0.115183315	26.11005639	0.690694909
WFDC2	0.363222067	0.935249981	0.370174545	2.362919169	0.887428055
WFDC3	0.5850625	1.310511244	0.445596312	3.854250305	0.623205217
WFDC5	0.855832166	0.002713531	6.40E-06	1.150561141	0.055552893
WFDC6	0.968624282	0.901404377	0.331381086	2.451949994	0.83889463
WFDC8	0.064106685	0.644315023	0.136423198	3.043044399	0.578921066
WFDC9	0.635048528	0.606692674	0.22114856	1.664383435	0.331776642
WFIKKN1	0.548346722	1.007162509	0.332540224	3.050386829	0.989928368
WFIKKN2	0.134326114	1.206701956	0.449176251	3.24177783	0.709412487
WFS1	0.167486351	0.482348318	0.150473224	1.54618804	0.219926398
WHSC1	0.322527064	0.026341592	4.17E-05	16.62842044	0.268965616
WHSC1L1	0.365477064	1.196661701	0.485517377	2.949429406	0.696475883
WIBG	0.651527068	0.682570733	0.263925528	1.765281324	0.430861726
WIF1	0.270460871	2.713261149	7.60E-06	968223.572	0.878384377
WIPI1	0.44929389	0.419874981	0.148685001	1.18569458	0.10134108
WIPI2	0.608089657	1.05670183	0.906629597	1.231615161	0.480365534
WISP1	0.681488263	1.046950901	0.469486361	2.334692296	0.910721226
WISP2	0.893459335	1.460077476	0.569747461	3.741703795	0.430524455
WISP3	0.141478969	0.723060782	0.218466683	2.39311957	0.595413401
WNK1	0.716574139	0.406066605	1.93E-05	8559.232449	0.859178501
WNK2	0.160948392	1.950505305	0.436171517	8.7224195	0.381992837
WNK3	0.897924315	19.60193393	0.242892665	1581.916085	0.184088608
WNK4	0.286831008	0.334941313	0.064868893	1.729421897	0.191574164

WNT1	0.346548424	1.285366698	0.677840983	2.437396954	0.441926254
WNT10A	0.459178411	0.317909546	0.00344069	29.37389587	0.619714698
WNT10B	0.851520453	0.769325789	0.106337249	5.565896952	0.795070877
WNT11	0.086907423	2.773287802	0.853051903	9.01601087	0.089934312
WNT16	0.73713394	36.24058325	0.698443524	1880.438187	0.074775616
WNT2	0.236155361	2.018625232	0.533454968	7.638597583	0.300901912
WNT2B	0.473761235	0.004718106	1.34E-06	16.59631211	0.198555091
WNT3	0.591607129	2.174330584	0.13887883	34.04200247	0.579986791
WNT3A	0.301740651	0.61862284	0.003263169	117.2768646	0.857567534
WNT4	0.932142373	2.029157485	0.513350359	8.020799106	0.312931403
WNT5A	0.167707749	0.610284829	0.273168986	1.363432865	0.228555696
WNT5B	0.110401857	0.192316729	0.029618831	1.248723278	0.08412322
WNT6	0.978512204	1.096076136	0.000349993	3432.593842	0.982178882
WNT7A	0.711896539	1.496118171	0.182534441	12.2627246	0.707400847
WNT7B	0.882917794	1.206933599	0.533242438	2.731756906	0.651785758
WNT8A	0.185028382	2.456599513	0.212514014	28.39756796	0.471687428
WNT8B	0.753777471	1.454371194	0.247670051	8.540376839	0.67834671
WNT9A	0.277342784	0.011857377	4.69E-06	29.96850468	0.267259732
WNT9B	0.670900599	1.06416199	0.632212358	1.791234742	0.814928925
WRB	0.74034344	1.091159969	0.020121123	59.17314383	0.965845042
WRN	0.785419194	1.031512345	0.581751397	1.828990398	0.915444312
WRNIP1	0.653285687	0.813087937	0.444708495	1.486618764	0.501531463
WSB1	0.063938856	0.052535862	0.002725138	1.012798828	0.05099283
WSB2	0.301454481	1.106377652	0.832010691	1.471220888	0.486924664
WT1	0.814952206	0.588164491	0.000607479	569.4640722	0.879739819
WTAP	0.1967509	0.014080846	0.000161203	1.229940055	0.061593077
WTIP	0.245127815	0.963588513	0.454175053	2.044372133	0.9230059
WWC1	0.243162792	0.091493922	0.003517743	2.379690235	0.150297867
WWC2	0.684985572	0.995638467	0.48726839	2.034394138	0.990434196
WWC3	0.594263255	1.858951487	0.724138006	4.77215752	0.197416022
WWOX	0.914632702	0.914163918	0.379354859	2.202939146	0.841489338
WWP1	0.857245092	1.285249627	0.491863565	3.35838375	0.608593266
WWP2	0.991992027	0.926267592	0.717982142	1.194976312	0.55562797

WWTR1	0.578029322	140.7038909	0.056324218	351493.2934	0.21524059
XAB2	0.104994396	0.114822189	0.012313813	1.070678549	0.057431454
XAGE3	0.988920171	1.323699532	0.327863983	5.344229743	0.693701442
XAGE5	0.07314731	0.283309339	0.023093908	3.475556505	0.324120424
XCL1	0.102346383	0.515349811	0.223257876	1.189590408	0.120375912
XCL2	0.826490813	1.057185237	0.526692746	2.121997378	0.875692723
XCR1	0.053145684	0.942581703	0.443441302	2.003557772	0.877846987
XDH	0.353981683	0.971676692	0.739410857	1.27690253	0.836673426
XG	0.652700916	1.093107889	0.905917461	1.318977621	0.352917497
XIST	0.917552019	1.018740972	0.112227107	9.247615781	0.986836946
XK	0.654514303	1.833363319	0.668017825	5.031633788	0.239295964
XKR3	0.908629539	0.898747928	0.358527874	2.25295687	0.819899805
XKR4	0.432672635	1.198622092	0.681947943	2.106751597	0.528940722
XKR5	0.931707586	0.974144063	0.812516754	1.167922569	0.77717365
XKR6	0.145371381	0.950656269	0.834565418	1.082895746	0.446355395
XKR7	0.759768271	1.121346593	0.107439556	11.70349385	0.92375053
XKR8	0.78039592	0.93514554	0.447014135	1.95630767	0.85868282
XKR9	0.466251839	0.923165204	0.473333262	1.800494623	0.814544083
XKRX	0.398748444	0.663093565	0.008865588	49.59547594	0.851956143
XKRY	0.571692908	1.599856827	0.626089914	4.088137842	0.326243476
XPA	0.667959118	1.413912515	0.104496061	19.13132969	0.794400932
XPC	0.817383482	1.144697366	5.39E-05	24300.31794	0.978790659
XPNPEP1	0.163265111	1.170605244	0.509754904	2.68818726	0.710363491
XPNPEP2	0.937051567	0.876571294	0.262134562	2.931232069	0.830632338
XPO1	0.776580445	41.66531026	0.009424074	184208.8803	0.383838316
XPO4	0.947495578	0.711318437	0.245316038	2.062539092	0.530569775
XPO5	0.083703146	0.658658654	0.277464753	1.563554354	0.343821372
XPO6	0.11200226	0.811626546	0.543065532	1.212998455	0.308641405
XPO7	0.299448323	0.343296046	0.074766681	1.576265978	0.16918931
XPOT	0.542494872	0.470985067	0.155951918	1.422405933	0.181827044
XPR1	0.759352685	0.144760182	0.002047278	10.23579183	0.373736787
XRCC1	0.280502658	0.809954857	0.18161215	3.612241082	0.782308761
XRCC2	0.282808534	1.399678945	0.899870471	2.177092385	0.135736581

XRCC3	0.244630785	0.079113885	0.000339313	18.44614213	0.361749683
XRCC4	0.982443916	1.234220474	0.341624939	4.458984117	0.748130471
XRCC5	0.63005647	1863.241873	2.67E-07	1.29913E+13	0.514943716
XRCC6	0.944787407	1.005273187	0.35422759	2.852895167	0.992115101
XRCC6BP1	0.667612399	71.31800229	0.006619186	768411.342	0.367718067
XRN1	0.182478026	10.83648035	0.011311071	10381.80238	0.496291632
XRN2	0.444854221	0.954077433	0.693119906	1.313284672	0.773081491
XRRA1	0.326272224	0.501021976	0.18511965	1.356004187	0.173683512
XYLB	0.70866917	7.63878978	0.001331423	43826.13807	0.645193456
XYLT1	0.988839441	0.048405211	4.58E-05	51.20799537	0.39407897
XYLT2	0.204970912	0.894656795	0.308941245	2.590818789	0.837425658
YAF2	0.43487644	0.920190277	0.685123167	1.235909375	0.580507945
YAP1	0.418408706	1.696079143	0.725971994	3.962528145	0.222357812
YARS	0.564599727	0.061529957	1.27E-06	2983.418795	0.61249427
YARS2	0.077048908	0.146165973	0.014989103	1.425334936	0.097932848
YBX1	0.100417827	2662.901845	0.003377478	2099508985	0.254904077
YBX2	0.527755836	1.172140073	0.316625354	4.339236684	0.812003744
YEATS2	0.088059345	0.514732397	0.099588641	2.660438356	0.428115932
YEATS4	0.204419459	4.052143772	0.001037095	15832.56606	0.740196052
YES1	0.270974366	0.873676093	0.01332263	57.29423562	0.949549503
YIF1A	0.614811391	3.257674086	0.167981991	63.1760608	0.434971602
YIF1B	0.590498488	0.907843912	0.617321671	1.335090938	0.623197478
YIPF1	0.228310901	1.567253343	0.002234749	1099.131754	0.893093107
YIPF2	0.165618528	0.800564624	0.545477557	1.174940579	0.255806325
YIPF3	0.860453517	0.651579291	0.139160332	3.050837598	0.58655268
YIPF4	0.315390366	1.177800443	0.864344102	1.604932434	0.299941514
YIPF5	0.465821888	0.07229824	0.001632373	3.202108644	0.174390098
YIPF6	0.841723914	0.013262154	2.87E-05	6.121652663	0.167247435
YIPF7	0.08259059	0.034399898	0.001136535	1.041193528	0.052774401
YKT6	0.388858513	1.010341055	0.571542976	1.786023256	0.971765669
YME1L1	0.091514745	1.78634399	0.309054399	10.32512353	0.516890083
YOD1	0.301545054	0.790804098	0.19928539	3.138068081	0.738567336
YPEL1	0.346911872	0.002674806	3.04E-06	2.355846218	0.086845159

YPEL2	0.692936403	9.304048393	1.94E-06	44681203.12	0.776292298
YPEL3	0.470785332	1.019239765	0.403231928	2.576308142	0.967870402
YPEL4	0.594411292	0.937367109	0.619945204	1.417314128	0.759131057
YPEL5	0.826834573	0.897970164	0.689661918	1.169196667	0.424193923
YRDC	0.250300792	1.149069139	0.841734903	1.568617245	0.38156654
YTHDC1	0.373815136	1.033335261	0.296154633	3.605487278	0.958982723
YTHDC2	0.596286332	0.952057664	0.691687146	1.310438975	0.763115342
YTHDF1	0.628677097	0.908633439	0.365148395	2.261038902	0.836796358
YTHDF2	0.087081585	3.591699282	0.466817203	27.63459371	0.219374152
YTHDF3	0.05173067	0.018314968	2.97E-05	11.29725394	0.222351592
YWHAB	0.665300136	0.358403839	0.006092309	21.0845041	0.621611229
YWHAE	0.570362606	0.894165233	0.381837364	2.093905779	0.79666119
YWHAG	0.542723595	0.74133967	0.241497705	2.275733871	0.600965662
YWHAH	0.433987077	0.973180546	0.10749346	8.810585998	0.980705051
YWHAQ	0.685329015	0.897427027	0.617405451	1.304451179	0.570618319
YWHAZ	0.455835298	1.321682063	0.437084831	3.996577668	0.621295471
YY1	0.156085651	0.540317086	0.185638629	1.572639031	0.258747753
YY1AP1	0.073542645	1.490944473	0.896986378	2.478204214	0.123408242
YY2	0.994275001	1.13531218	0.048380471	26.64161201	0.9371726
ZADH2	0.170533733	0.000300498	5.33E-10	169.3830954	0.229999605
ZAN	0.282354671	1.246426761	0.748634976	2.075216522	0.397044343
ZAP70	0.617548293	0.966371948	0.090635492	10.30363187	0.977400715
ZAR1	0.658812483	1.062589903	0.664266482	1.699765581	0.800048972
ZBED1	0.945286551	1.000995467	0.745538379	1.343984364	0.994719213
ZBED2	0.510879104	7.963883246	1.38E-05	4587951.896	0.75914721
ZBED3	0.958185315	0.676323046	0.154773196	2.955375184	0.603223242
ZBED4	0.127983756	1.244044865	0.768109748	2.014878251	0.374754779
ZBP1	0.954818869	27.59905889	0.00540205	141003.5063	0.446325748
ZBTB1	0.65942742	0.997381244	0.805233537	1.235379925	0.980840005
ZBTB10	0.140973227	2.466588062	0.100129015	60.76217431	0.580767664
ZBTB11	0.882430773	1.123030758	0.568494033	2.21848957	0.738345197
ZBTB12	0.196068488	0.259809433	0.05396013	1.250941057	0.092809861
ZBTB16	0.599829837	0.566429013	0.200225591	1.602401697	0.28403544

ZBTB17	0.424160339	0.557092714	0.111307012	2.788254646	0.476467731
ZBTB2	0.182466574	7.692964906	0.986882356	59.96835255	0.051491462
ZBTB20	0.907976304	1.637126609	0.33792219	7.931362952	0.540334045
ZBTB24	0.180669627	0.82498166	0.302588745	2.249240097	0.70694461
ZBTB25	0.333344078	1.557429255	0.607967684	3.98966252	0.355953371
ZBTB26	0.504507013	0.738881984	0.221305907	2.466931828	0.622738602
ZBTB3	0.216379891	0.213762921	0.032027684	1.426721512	0.111152484
ZBTB32	0.246698222	2.295006836	0.378221419	13.9258543	0.366498497
ZBTB33	0.213917951	1.26153846	0.520688272	3.056491516	0.606852796
ZBTB34	0.610801318	0.189102607	0.012002525	2.979356141	0.236447534
ZBTB37	0.943108804	0.328766211	0.022159549	4.877681515	0.418868418
ZBTB39	0.884315344	0.013251076	6.52E-06	26.91899793	0.265873347
ZBTB4	0.70578263	0.007727708	2.42E-07	247.1877384	0.358180169
ZBTB40	0.132535061	0.383678552	0.122735337	1.199403815	0.099496968
ZBTB41	0.222940927	1.318151665	0.831507486	2.089606936	0.239972229
ZBTB5	0.879873345	1.407569023	0.602256536	3.289712004	0.429953033
ZBTB7A	0.386430065	0.908135114	0.315168993	2.616721198	0.858358124
ZBTB7B	0.279762668	0.590871363	0.140209457	2.490052921	0.473428922
ZBTB8OS	0.896144898	0.526991155	9.32E-05	2980.050458	0.884467957
ZBTB9	0.276939916	0.463848459	0.163832117	1.313267489	0.147971319
ZC3H10	0.369908897	0.826897046	0.053931785	12.67821417	0.891454763
ZC3H11A	0.05450119	1.861466109	0.897767397	3.859636789	0.094899457
ZC3H12A	0.673875719	1.563317743	0.536454311	4.555769827	0.41292369
ZC3H12B	0.765447759	0.661407829	0.171451033	2.551517529	0.548419729
ZC3H3	0.478160937	5.522391699	0.002190299	13923.58454	0.668940602
ZC3H6	0.41218991	5.872378432	0.036242862	951.492966	0.495265742
ZC3H7A	0.074782092	0.047905912	0.001242388	1.847230062	0.102969002
ZC3H7B	0.094505926	1.191249708	0.853311547	1.66302199	0.303915693
ZC3H8	0.621900242	33.76103563	0.003106117	366955.727	0.457970108
ZC3HAV1	0.343904627	0.67533566	0.168886702	2.700498315	0.578818146
ZC3HC1	0.528860679	1.069076958	0.82367851	1.387586939	0.615640095
ZCCHC10	0.714700373	6.362167829	5.09E-05	794879.3819	0.757297946
ZCCHC11	0.670302344	0.042399952	1.27E-06	1410.553186	0.551886323

ZCCHC12	0.239549649	0.768780101	0.35464187	1.666534308	0.505335717
ZCCHC13	0.594019479	1.220121799	0.269358862	5.526817252	0.796312121
ZCCHC14	0.820113112	1.028607015	0.837318433	1.263596202	0.788179276
ZCCHC16	0.206774448	19.28710699	0.037099311	10026.93824	0.353651935
ZCCHC17	0.323869156	0.005055436	3.18E-06	8.028139695	0.15971054
ZCCHC2	0.658260894	2.453844719	0.68856878	8.744738484	0.166215927
ZCCHC3	0.304414985	0.082620341	0.002786105	2.450058412	0.149356515
ZCCHC4	0.478416143	0.813318424	0.261633508	2.528295645	0.721031826
ZCCHC5	0.547286526	1.281720461	0.517369825	3.175305673	0.591798602
ZCCHC6	0.432943621	1.025017509	0.684254127	1.53548346	0.904612832
ZCCHC7	0.444278759	0.679479137	0.124140449	3.719109295	0.655926493
ZCCHC8	0.348048512	1.976910882	0.878137093	4.45053132	0.099744559
ZCCHC9	0.925764371	0.272150078	0.02395568	3.091778857	0.293898894
ZCRB1	0.969940372	0.99815664	0.694421622	1.434743169	0.992047799
ZCWPW1	0.489910109	7.69369581	0.030977463	1910.839366	0.468361602
ZDHHC1	0.247512227	2.271174332	0.515473913	10.00677768	0.278299653
ZDHHC11	0.45829896	0.854907981	1.42E-05	51543.97548	0.977730835
ZDHHC12	0.308882742	1.094764412	0.917529247	1.306235328	0.315004584
ZDHHC13	0.077708515	1.290233096	0.96672378	1.722003201	0.083597716
ZDHHC14	0.988141613	1.063465241	0.819968873	1.379269819	0.64278053
ZDHHC15	0.498496433	12.54925478	0.000445597	353422.2712	0.628448326
ZDHHC16	0.882126368	0.932767034	0.461766047	1.884188639	0.846162214
ZDHHC17	0.600043399	0.97895579	0.7616019	1.2583404	0.868126552
ZDHHC18	0.928847449	1.198642866	0.402464518	3.569866798	0.744875352
ZDHHC19	0.808578128	0.918657737	0.723120519	1.167069688	0.487194424
ZDHHC2	0.530953678	0.913470144	0.740551804	1.1267648	0.3979561
ZDHHC20	0.177605511	3.782472989	0.022103698	647.2718707	0.612113598
ZDHHC21	0.235234134	0.636487193	0.175805649	2.304339764	0.491294481
ZDHHC22	0.445304394	1.384680699	0.354817654	5.403735174	0.639432704
ZDHHC23	0.043262671	2.657053333	1.490211566	4.737536989	0.000926412
ZDHHC24	0.548905264	40.21028476	0.003202783	504831.8618	0.442986356
ZDHHC3	0.221235161	5.447856796	0.499560599	59.41049738	0.164335906
ZDHHC4	0.727149214	0.319381999	1.19E-06	86040.23569	0.858010913

ZDHHC5	0.111775683	1.164552939	0.865880198	1.566248484	0.313683249
ZDHHC6	0.415931009	1.03061668	0.451255291	2.353813375	0.942944968
ZDHHC7	0.359845036	0.079547281	0.002682961	2.358502374	0.143247661
ZDHHC8	0.645905443	0.836308869	0.257749226	2.713538799	0.765956996
ZDHHC9	0.715029986	1.41254422	0.15236048	13.09579216	0.761134835
ZFAND1	0.066539034	1.799613039	0.782912497	4.136614374	0.166465984
ZFAND2A	0.505213807	233.1610775	0.003386059	16055269.65	0.337463427
ZFAND2B	0.780044744	2.768879643	0.000161419	47495.65475	0.83778278
ZFAND3	0.576886286	18.0449457	0.710926801	458.0219296	0.079569091
ZFHX2	0.813172328	1.241139014	0.656930533	2.344884237	0.505716033
ZFHX4	0.477915766	2.378604146	0.763888924	7.406518805	0.134858146
ZFP1	0.322844776	0.04205718	0.000208672	8.476471338	0.24180804
ZFP2	0.587836472	0.003836146	1.92E-07	76.57110114	0.270796307
ZFP28	0.944456597	0.52684636	3.81E-06	72775.84148	0.915487083
ZFP3	0.17959334	140.2661784	0.094463796	208276.6271	0.184600368
ZFP36	0.119310665	1.243552681	0.071114725	21.74547212	0.881315268
ZFP36L1	0.535330282	1.110979978	0.42980968	2.871681516	0.828047261
ZFP37	0.312969087	0.000370989	2.41E-09	57.14559305	0.19492427
ZFP41	0.283899917	3.806859765	0.609083684	23.79341567	0.152800844
ZFP42	0.043037077	1.71787187	1.139339155	2.590171458	0.009806152
ZFP64	0.770806677	1.058719914	0.84671796	1.323803095	0.616720503
ZFP90	0.048226782	0.203007104	0.076698513	0.537323121	0.001323988
ZFP91	0.496194958	0.528002833	0.153062474	1.821393483	0.312069845
ZFPL1	0.604332119	0.045070134	0.000129435	15.69368621	0.299287733
ZFPM1	0.616386628	0.343965566	0.025225438	4.690198479	0.423367251
ZFPM2	0.420237194	1.287235582	0.767877496	2.15786431	0.338100784
ZFR	0.583991412	0.930205363	0.329065839	2.629510318	0.891456793
ZFX	0.79102472	0.036644393	1.18E-05	113.8036555	0.420272291
ZFY	0.100507994	8.642927311	0.110245643	677.5795457	0.332480555
ZFYVE1	0.332087294	3.97046456	0.108793071	144.9043462	0.452473497
ZFYVE16	0.211480356	99.60197366	0.126329454	78529.2177	0.176363258
ZFYVE19	0.842646927	0.832698248	0.388867827	1.783090097	0.637449835
ZFYVE21	0.119988276	14.35759667	0.000402772	511804.6734	0.618339839

ZFYVE26	0.33992395	1.274805726	0.006120459	265.5241232	0.928977125
ZFYVE27	0.774410519	0.562162395	0.083177312	3.799432183	0.55466863
ZFYVE28	0.338208589	1.247617575	0.599165341	2.597863239	0.554389632
ZFYVE9	0.144227159	220.3461254	0.647315613	75005.78397	0.069715824
ZG16	0.921073049	0.692356177	0.106340362	4.507762324	0.700510444
ZGPAT	0.390049735	1.810588398	0.363394393	9.021136302	0.468740334
ZHX1	0.759409361	1.089917968	0.871722464	1.362728652	0.449978345
ZHX2	0.792581498	0.99523379	0.445879018	2.221432848	0.990695119
ZHX3	0.841379445	0.941844524	0.325439353	2.725764719	0.912007632
ZIC1	0.126094274	1.696851681	0.729180217	3.948688624	0.21980303
ZIC2	0.717963638	1.407893857	0.547343452	3.621428387	0.47789823
ZIC3	0.883328849	0.910575812	0.665549549	1.245810039	0.558056049
ZIC4	0.921673265	0.561239148	0.239553275	1.314903259	0.183608292
ZIC5	0.083765105	2.296720309	0.581302833	9.074313552	0.235578645
ZIK1	0.114945014	1.83289402	0.967272524	3.473168527	0.063179093
ZIM2	0.362506832	2.183061356	0.828860311	5.74977089	0.114088697
ZIM3	0.083016062	2.417469174	0.169792506	34.41940598	0.514776422
ZKSCAN1	0.056482606	1.336086358	0.87657813	2.036471932	0.177855315
ZMAT1	0.305648211	0.006840693	3.92E-06	11.94896669	0.190633994
ZMAT2	0.998563919	0.890040399	0.211900552	3.738413634	0.873600636
ZMAT4	0.687222553	0.974598099	0.490686528	1.935739824	0.941416453
ZMAT5	0.109121626	924.4029839	0.009579672	89201477.44	0.243529942
ZMPSTE24	0.483118806	0.05672245	0.00123043	2.614887565	0.14205743
ZMYM1	0.438096185	1.161173688	0.859026646	1.569595472	0.331162563
ZMYM3	0.349000237	0.118259595	9.49E-05	147.4450381	0.557208839
ZMYM4	0.160403819	2.492487108	0.436539721	14.2312181	0.304203539
ZMYM5	0.050020101	25.18134321	0.000152149	4167629.688	0.598759075
ZMYM6	0.523318205	1.077580314	0.701078827	1.656274998	0.733340273
ZMYND10	0.12975708	1.035780128	0.783830025	1.368715715	0.804744467
ZMYND11	0.229630602	0.010325314	6.36E-05	1.675531161	0.078205546
ZMYND12	0.304703372	1.152330801	0.617627662	2.149946247	0.655891321
ZMYND15	0.268956292	0.777899445	0.16259991	3.721573684	0.75315473
ZMYND19	0.122501846	1.199002318	0.951184976	1.511384846	0.124460683

ZNF10	0.078294077	0.000103418	3.80E-11	281.7966236	0.224822169
ZNF100	0.303325255	0.46332043	0.017705578	12.12419132	0.644157209
ZNF101	0.70767541	0.39736558	0.072428287	2.180079221	0.28795627
ZNF114	0.840228996	0.832093999	0.175070106	3.954875219	0.817221402
ZNF117	0.166020406	1.364669819	0.462702837	4.024880695	0.573153982
ZNF12	0.590378071	1.179087048	0.478097764	2.907870255	0.720570703
ZNF121	0.579021647	1.092015724	0.375705408	3.174024961	0.871544702
ZNF124	0.577951884	0.788756308	0.217992561	2.853934608	0.717605081
ZNF132	0.230818595	0.113448588	0.005615979	2.291778724	0.155846625
ZNF133	0.889000632	6.320095674	0.004598543	8686.144202	0.616999277
ZNF134	0.950783712	1.834317499	0.008779568	383.2444502	0.82385749
ZNF135	0.253328584	0.946516866	0.41965599	2.134829953	0.894624799
ZNF136	0.882049677	0.955911085	0.715079645	1.277852067	0.76077847
ZNF138	0.202161378	1.126542578	0.965526286	1.314410802	0.129986388
ZNF14	0.931328165	2.025350315	0.784547141	5.228549932	0.144702332
ZNF140	0.27375092	0.168390799	0.004170496	6.799061466	0.345105746
ZNF141	0.300251109	0.591579364	0.000828232	422.5457898	0.87557897
ZNF142	0.952407447	0.840575586	0.552619821	1.278577583	0.41704131
ZNF143	0.861305922	4.970093084	0.173334262	142.5097671	0.349044321
ZNF146	0.159553597	1.432431664	0.716164087	2.865070322	0.309596342
ZNF148	0.470514011	1.013869714	0.177055317	5.805709841	0.987656691
ZNF154	0.07266121	2.090838179	0.669547022	6.529196828	0.204263656
ZNF155	0.111952052	1.48909716	0.986965498	2.246694901	0.057769766
ZNF157	0.160661965	0.371389424	0.124923228	1.104118959	0.074783503
ZNF16	0.600167373	1.084030266	0.740467639	1.586999291	0.678218246
ZNF160	0.463739566	173.2488563	0.005124339	5857373.688	0.332646466
ZNF165	0.245579894	0.750344006	0.017035996	33.04861849	0.881772017
ZNF169	0.494192424	0.503682549	0.176647591	1.436170786	0.199543174
ZNF17	0.151900582	3.41E-06	6.00E-12	1.93251143	0.062532576
ZNF174	0.421305106	0.488939298	0.173610046	1.377003481	0.175607097
ZNF175	0.981563413	0.62432954	0.204080923	1.909964781	0.408960244
ZNF177	0.572560283	1.122707117	0.483407505	2.607471454	0.787763351
ZNF18	0.196849161	0.331541855	0.053025166	2.072977979	0.237809875

ZNF180	0.906247284	7.72092995	0.000392435	151904.9742	0.685345701
ZNF181	0.143741326	0.971890856	0.810752698	1.165055432	0.757882261
ZNF184	0.192316216	26.51742428	0.242959904	2894.196859	0.170991203
ZNF185	0.823738686	0.409760843	0.074616465	2.250226528	0.304574045
ZNF189	0.068102497	0.977694983	0.28515805	3.352132196	0.971376576
ZNF19	0.929051095	1.491100666	0.674262326	3.297501744	0.323827363
ZNF195	0.570689229	0.002916302	8.93E-08	95.2803694	0.271018656
ZNF197	0.271920509	0.923709505	0.780871619	1.092675452	0.354500806
ZNF2	0.095137673	0.749053069	0.380348407	1.475175103	0.403364986
ZNF20	0.540550255	1.223920694	0.68184992	2.196937806	0.498426911
ZNF200	0.965197227	0.006495416	3.86E-06	10.91972265	0.183808801
ZNF202	0.63571843	1.290846281	0.40470064	4.117325143	0.666182787
ZNF205	0.276577973	1.096462476	0.577800623	2.080700355	0.778137795
ZNF207	0.39241013	1.209318077	0.143697122	10.17731037	0.861177049
ZNF208	0.592872697	0.656627638	0.234289683	1.840285277	0.423717172
ZNF211	0.282963446	36.0322781	0.026184147	49584.3944	0.331004789
ZNF212	0.704934365	0.74776492	0.477492562	1.171017981	0.204044738
ZNF213	0.970522761	0.187390409	0.000176039	199.4738225	0.637733513
ZNF214	0.339476093	1.92921556	0.729027405	5.105257568	0.185687979
ZNF215	0.342698147	0.001333431	5.91E-08	30.07704686	0.195520281
ZNF217	0.134700443	0.485397395	0.148138059	1.590480077	0.232619656
ZNF219	0.580411341	0.96574676	0.252008575	3.700932811	0.959446226
ZNF22	0.838500309	1.06771785	0.315984231	3.607842712	0.915999686
ZNF221	0.059953748	1.045543944	0.733523039	1.490290122	0.805462099
ZNF222	0.061916005	1.385012514	0.598613251	3.20450585	0.446646832
ZNF223	0.596593105	0.668571229	0.020639936	21.65643747	0.820508937
ZNF224	0.335452802	2.862939013	0.00323136	2536.523165	0.761304303
ZNF225	0.175821597	0.517044173	0.011881958	22.49921135	0.731863795
ZNF226	0.089266955	3.286291404	0.199270263	54.19630119	0.405426568
ZNF227	0.365877228	1.043613453	0.256857364	4.240209508	0.95240912
ZNF229	0.085331251	7947.265148	0.048107097	1312883694	0.142925953
ZNF23	0.641754198	0.795894193	0.266797495	2.374263547	0.682263757
ZNF230	0.959368032	0.66122027	0.241863289	1.807683371	0.420145908

ZNF232	0.788726205	1.19809757	0.012485985	114.9639191	0.938132887
ZNF233	0.273258844	0.971006645	0.733568923	1.285296957	0.837066213
ZNF234	0.129077708	4.55639207	0.423675113	49.00148266	0.210808296
ZNF235	0.569419094	0.911848841	0.657130429	1.265301792	0.580871175
ZNF236	0.363569597	15.67276149	0.528922239	464.4074962	0.111474763
ZNF239	0.213150152	0.677550867	0.000277642	1653.480834	0.922078342
ZNF24	0.245421863	3.436806349	0.02561741	461.0785333	0.621372432
ZNF248	0.80858546	0.969199953	0.626954152	1.498273114	0.88805688
ZNF25	0.843513607	1.043044199	0.595939642	1.825589582	0.88268757
ZNF250	0.611245109	1.321653191	0.382378177	4.568166441	0.659411139
ZNF251	0.606050381	0.068071113	0.000272276	17.01828018	0.340146173
ZNF253	0.735584566	0.198450588	0.008258981	4.768462065	0.318767232
ZNF254	0.353511105	1.100311324	0.64064026	1.889804756	0.729044628
ZNF256	0.077808265	1.883994698	0.449201366	7.901659013	0.386541931
ZNF257	0.319262265	0.37065959	3.16E-05	4352.818355	0.83555982
ZNF26	0.372280473	0.089057165	2.18E-05	364.1263927	0.568676061
ZNF263	0.983338805	0.624834197	0.11342781	3.441993417	0.58907678
ZNF264	0.486309563	2.060335871	0.577648935	7.348726265	0.265221761
ZNF266	0.433260315	0.827857181	0.179899898	3.809604792	0.808339898
ZNF267	0.65138197	0.688279839	0.259926585	1.822549766	0.452132011
ZNF268	0.331261025	0.005996365	8.43E-06	4.267537527	0.126777261
ZNF273	0.565383919	1.432688811	0.345200099	5.94610846	0.620483675
ZNF274	0.626934664	0.505511474	0.000483675	528.3339635	0.847484207
ZNF276	0.433912365	0.123633072	0.004684155	3.263157369	0.210656733
ZNF277	0.192296809	0.019189848	1.40E-05	26.21672167	0.283168229
ZNF28	0.396804244	2.781333112	0.021387465	361.6985019	0.68043883
ZNF281	0.51917874	118.0694552	0.000468777	29737792.93	0.452091781
ZNF282	0.779618318	0.926580663	0.054637314	15.71365191	0.957894066
ZNF283	0.732025615	1.060227036	0.204183105	5.505261401	0.944522611
ZNF284	0.688149603	0.733897162	0.333272646	1.616109367	0.442396285
ZNF285	0.457718979	1.554568147	0.573230362	4.215900421	0.3860761
ZNF287	0.402954042	54.69853973	0.105237394	28430.29595	0.209741705
ZNF292	0.227571066	1.932465277	0.804821164	4.640064419	0.14045266

ZNF3	0.149234506	1.520550167	0.602151203	3.839688099	0.375240743
ZNF30	0.851440644	0.626941229	0.175638346	2.237867261	0.472025726
ZNF300	0.439396025	1.405783503	0.965187522	2.047506016	0.075852046
ZNF302	0.449423706	0.435374579	0.019619403	9.661406291	0.599029565
ZNF304	0.364916621	1.038046631	0.451978156	2.384055056	0.929859685
ZNF311	0.091732788	1.916642231	0.757031745	4.85252761	0.169856347
ZNF317	0.288935418	0.861408142	0.442828997	1.675644533	0.660338186
ZNF318	0.848290996	0.75108707	0.273504148	2.062607794	0.578661717
ZNF319	0.722727643	0.034494289	5.12E-07	2326.167768	0.552844597
ZNF32	0.319056914	0.793915459	0.303178456	2.078979371	0.638452154
ZNF324	0.638168611	0.917209633	0.637680737	1.319270698	0.641238426
ZNF326	0.396309597	0.279119036	0.074102935	1.051340766	0.059298846
ZNF329	0.230852089	1.42351375	0.155131	13.06245297	0.75485701
ZNF330	0.251332341	0.97618509	0.418354812	2.27782089	0.955538213
ZNF331	0.927552799	1.166376285	0.754212057	1.803781346	0.489021835
ZNF333	0.370409878	0.623288557	0.327322239	1.186869022	0.150257987
ZNF334	0.121243527	1.379388229	0.053047581	35.86802377	0.846581801
ZNF335	0.602122681	0.792860142	0.573077541	1.096932194	0.161100409
ZNF337	0.9230666	0.955531445	0.745754447	1.224317663	0.719087275
ZNF33A	0.939943781	0.944117886	0.275280455	3.238001711	0.927136505
ZNF34	0.953640075	1.12628005	0.172777794	7.341839048	0.90105327
ZNF341	0.937047194	1.063256646	0.671413518	1.683783042	0.793700115
ZNF343	0.840696812	0.779027765	0.480306325	1.263535844	0.311544215
ZNF345	0.835865825	4.362653257	0.309876275	61.42046027	0.274963919
ZNF346	0.828064305	0.980395668	0.358227422	2.683143748	0.969254131
ZNF347	0.952853168	1.196768525	0.702221974	2.03960422	0.509022397
ZNF35	0.829501347	0.009926033	3.36E-06	29.2963637	0.257856892
ZNF350	0.516461839	0.923153147	0.686551515	1.241293208	0.596630364
ZNF354A	0.633655008	0.045267482	1.06E-06	1935.269783	0.569415217
ZNF354B	0.444369063	1.314408842	0.993725227	1.738579798	0.055383831
ZNF354C	0.770217808	1.010838248	0.357022132	2.861990543	0.983803048
ZNF358	0.518779819	0.961392934	0.712811746	1.296662659	0.79645062
ZNF365	0.508389773	1.0369645	0.512530616	2.098011982	0.919586248

ZNF366	0.139392837	0.6572249	0.246802975	1.750159491	0.40094986
ZNF367	0.736520303	1.287433987	0.572563198	2.894852962	0.541114647
ZNF37A	0.075569278	0.732859834	0.479220398	1.120744315	0.151569556
ZNF382	0.065429189	1.157742405	0.677318983	1.978930917	0.592296134
ZNF383	0.864603595	1.029380573	0.80736168	1.312453132	0.81528218
ZNF384	0.446551083	0.834300699	0.554315715	1.255706158	0.385153674
ZNF394	0.857710843	0.698812521	0.279656191	1.746211798	0.443105778
ZNF395	0.389895281	0.808386474	0.270749088	2.413632104	0.703096204
ZNF396	0.202978713	1.936595708	0.824163222	4.550558476	0.129443708
ZNF397	0.581967923	0.689173823	0.279196918	1.701166909	0.419390034
ZNF398	0.165179977	0.572180871	0.070590379	4.63789756	0.60102793
ZNF404	0.852053489	1.172067078	0.757433948	1.813677929	0.475995693
ZNF407	0.326054504	1.126930786	0.945467686	1.343222	0.182210346
ZNF408	0.915504231	0.350662313	0.02012482	6.110069926	0.472335025
ZNF41	0.820430873	0.923519698	0.381025699	2.238401858	0.860184215
ZNF410	0.924154159	4.085500841	0.827337608	20.17473516	0.084106498
ZNF414	0.113844329	1.030929174	0.931567843	1.140888417	0.55580667
ZNF415	0.14943217	1.225064175	0.782446289	1.918064224	0.374842712
ZNF416	0.561172087	1.135800248	0.079569158	16.21284212	0.925204469
ZNF417	0.140282672	1.056876265	0.823801935	1.355893196	0.663435799
ZNF418	0.989159937	1.099885063	0.66119974	1.82962436	0.713865887
ZNF419	0.735307127	1.260038073	0.968930588	1.638606486	0.084620084
ZNF420	0.492771588	2.86277549	0.000992578	8256.767079	0.795826856
ZNF423	0.544669354	4.648556211	0.001771414	12198.77486	0.702057422
ZNF425	0.974592484	1.013665567	0.749695766	1.37057981	0.929727798
ZNF426	0.801566731	0.622877618	0.205623807	1.886826882	0.402486768
ZNF429	0.27390417	1.395877496	0.571281839	3.410705279	0.46435456
ZNF43	0.552826122	10.03725836	0.282699087	356.3738269	0.205406524
ZNF430	0.272735579	0.896430017	0.496997768	1.616882062	0.716373425
ZNF431	0.072973613	0.09439063	0.005696851	1.563950151	0.099402174
ZNF432	0.463525496	2.508808187	0.186070974	33.82643939	0.488310566
ZNF433	0.115468291	0.882917668	0.657590403	1.185454661	0.40749509
ZNF436	0.130005043	0.559021526	0.18947902	1.649285848	0.292086864

ZNF438	0.138152193	2.378711961	0.801693493	7.057897616	0.118372022
ZNF439	0.311717668	0.967141386	0.813244236	1.150161809	0.705554288
ZNF44	0.282669256	1.147108911	0.85233904	1.543820934	0.365116527
ZNF440	0.75504349	0.98907347	0.769095211	1.27197038	0.931782958
ZNF441	0.079652465	1.822310272	0.833067054	3.986251425	0.132932378
ZNF442	0.212774215	1.661781087	0.099874311	27.6499166	0.723314186
ZNF443	0.991847314	1.194452714	0.434254897	3.285437413	0.730698342
ZNF444	0.870085718	1.003019798	0.128689117	7.817667406	0.997703623
ZNF445	0.793897221	0.301844217	0.029798911	3.057491934	0.310608261
ZNF446	0.860133085	1.066233441	0.799656601	1.421677441	0.662187443
ZNF449	0.488378144	5.99652346	0.799175882	44.99421768	0.081517023
ZNF45	0.255156662	0.000247092	6.00E-08	1.017019587	0.050466376
ZNF451	0.826482654	0.97363759	0.721714186	1.313498023	0.861169104
ZNF454	0.625897139	0.008331013	3.46E-07	200.3357979	0.352256354
ZNF462	0.272277837	0.724403427	0.327603688	1.60181446	0.425852709
ZNF467	0.41415237	2.920684816	0.373209519	22.85686553	0.307234777
ZNF468	0.533796974	86.87483603	9.48E-05	79605140.8	0.523869399
ZNF469	0.628716363	1.215961745	0.316641642	4.669515219	0.775775669
ZNF471	0.961238797	0.056619294	0.00023133	13.8578553	0.306214358
ZNF473	0.496091689	2.22077895	0.19191976	25.69750575	0.523046527
ZNF479	0.35996285	3.986143385	0.790244222	20.10687157	0.093966012
ZNF480	0.814390107	473.7967914	0.123524198	1817323.265	0.143398205
ZNF483	0.85728935	0.000789226	1.08E-09	575.183485	0.29958866
ZNF484	0.680868338	1.292243306	0.413124562	4.042104776	0.659476577
ZNF485	0.770710273	1.027288709	0.709530158	1.4873534	0.886615925
ZNF486	0.689649728	0.381600145	0.069847079	2.084821199	0.266151924
ZNF488	0.346969498	3.006823975	0.313610424	28.82873053	0.339817673
ZNF490	0.740883948	3.719315323	0.012344736	1120.58346	0.651970242
ZNF491	0.916505175	0.823581966	0.366474752	1.850843072	0.638497078
ZNF492	0.473615191	0.998179669	0.557896311	1.785928019	0.995102355
ZNF493	0.987256027	0.623684503	0.144547637	2.691032284	0.526800028
ZNF496	0.296756345	2.110892849	0.665956495	6.690930495	0.204336265
ZNF497	0.952092582	0.586039441	0.176781905	1.942745361	0.382173073

ZNF500	0.165751475	1.170375031	0.927284103	1.477193138	0.185365011
ZNF501	0.824473144	0.587962215	0.01811181	19.08696952	0.764858286
ZNF502	0.802651049	0.727926824	0.248845956	2.129339247	0.562013056
ZNF503	0.846337187	0.935805455	0.758573298	1.154445922	0.535700699
ZNF507	0.877714294	0.884631361	0.269303753	2.905910653	0.839906299
ZNF510	0.110268837	0.724532281	0.519856037	1.009793075	0.057115312
ZNF511	0.64343459	1.146585714	0.716968912	1.833634313	0.567985643
ZNF512	0.304135248	1.484351942	0.548311237	4.018339472	0.43695957
ZNF513	0.273789331	3.049570527	0.846891069	10.98120022	0.088057283
ZNF514	0.359201516	1.064513128	0.40800909	2.777360179	0.898328666
ZNF516	0.729178369	1.092781765	0.647563171	1.844101148	0.739634343
ZNF517	0.977516678	0.020880243	2.29E-05	19.03706556	0.265863914
ZNF519	0.536424297	1.016504565	0.591660693	1.746408954	0.952725475
ZNF521	0.990005679	0.463694784	0.047382164	4.53784371	0.509017142
ZNF524	0.669110784	0.904190708	0.716503404	1.141042502	0.39618825
ZNF525	0.815776377	0.330991233	9.18E-07	119278.3943	0.865506124
ZNF526	0.864384852	0.856383258	0.077564895	9.455208886	0.899314831
ZNF527	0.776994401	0.885222648	0.66674396	1.17529244	0.399194811
ZNF528	0.337085393	1.058564112	0.757334372	1.479607978	0.739047434
ZNF529	0.204542888	1.00817237	0.783533953	1.297214402	0.94954092
ZNF530	0.880077857	0.840834889	0.373742039	1.89168795	0.675178519
ZNF532	0.551359095	0.951210436	0.261702128	3.457370795	0.939445504
ZNF536	0.615348187	0.165713881	0.021106915	1.301047082	0.087329717
ZNF540	0.664261441	0.07043727	0.001081721	4.586589693	0.213086195
ZNF541	0.931182088	1.595670848	0.000220465	11549.04772	0.917917221
ZNF543	0.96513594	20.05673027	0.002745121	146540.8598	0.50886494
ZNF544	0.823023989	1.104939617	0.351418069	3.474185495	0.864433947
ZNF546	0.593251362	2.247269691	0.246295428	20.50472921	0.472880108
ZNF547	0.558724973	0.920433031	0.478566679	1.770279882	0.803781233
ZNF548	0.773115234	0.265370229	0.0018405	38.26208522	0.600936978
ZNF549	0.380467778	0.334383167	0.010624284	10.52420163	0.53361612
ZNF550	0.703413456	0.977580721	0.729553747	1.309929625	0.879298622
ZNF551	0.464719889	0.955527378	0.748284601	1.220167526	0.715333694

ZNF552	0.666128836	0.066128706	4.16E-05	105.1815379	0.470203392
ZNF554	0.447008344	0.826220864	0.61171906	1.115938608	0.213241349
ZNF555	0.11426096	0.598712762	0.305801221	1.172189468	0.134527009
ZNF556	0.81211436	0.306320852	0.063491677	1.477870319	0.140617262
ZNF557	0.252033501	0.672992472	0.253592417	1.786011081	0.426457049
ZNF558	0.503605316	0.910891881	0.668996201	1.240252212	0.55340112
ZNF559	0.156463745	1.094745029	0.575287717	2.083247466	0.782740665
ZNF560	0.114276655	0.692972984	0.408977808	1.174175095	0.172826459
ZNF561	0.151513089	48.73271838	0.118155252	20099.63848	0.20592237
ZNF562	0.428104154	7.804262665	0.000117439	518623.1881	0.716858385
ZNF563	0.887035533	0.829483803	0.269154904	2.556310025	0.744760351
ZNF564	0.54282392	0.298224181	0.023938953	3.715185914	0.347140002
ZNF565	0.28921293	1.641469441	0.582679505	4.624192032	0.348320655
ZNF566	0.508357177	1.167210629	0.786116674	1.733051463	0.443271505
ZNF567	0.764190036	0.665103823	0.281060084	1.573909355	0.353442878
ZNF568	0.11137496	0.010101369	3.34E-05	3.059344996	0.114942637
ZNF569	0.195317229	0.984522073	0.871572409	1.112109219	0.801895311
ZNF570	0.799613878	1.905381246	0.489002776	7.424247606	0.352870256
ZNF571	0.483217337	0.665487877	0.08518196	5.199153852	0.697820888
ZNF572	0.817004948	0.29830647	0.047099056	1.889353144	0.199000995
ZNF573	0.542566594	0.728794336	0.205220996	2.588142509	0.62464633
ZNF574	0.714057502	0.010484	9.71E-06	11.31557692	0.20086254
ZNF575	0.75276654	0.70881646	0.001074935	467.3965538	0.917237864
ZNF576	0.683647388	0.793145009	0.234723966	2.68007999	0.709113689
ZNF577	0.655983365	124.2186383	0.059353251	259973.461	0.21644813
ZNF578	0.103970962	1.581282798	0.775561478	3.224058129	0.207417666
ZNF579	0.366967316	1.015446044	0.327259723	3.150802243	0.978833497
ZNF580	0.883873891	2.913854834	0.467355215	18.16723067	0.252068185
ZNF581	0.616724945	1.335386771	0.586718676	3.039374581	0.490664236
ZNF582	0.740956273	0.504320839	0.072897929	3.488981288	0.487884195
ZNF583	0.439980805	10.43940644	0.041715827	2612.466649	0.405145422
ZNF584	0.140931833	1.604408353	0.520925027	4.941452283	0.410110396
ZNF585A	0.104293832	0.484165709	0.216336876	1.08357132	0.077615982

ZNF585B	0.837264563	1.152632357	0.528160774	2.515448736	0.721278118
ZNF586	0.104788673	0.614282787	0.305704381	1.234340643	0.17111056
ZNF587	0.115818837	0.950867442	0.806058532	1.121691362	0.550067558
ZNF589	0.476245378	1.004574464	0.853779571	1.182002812	0.956139465
ZNF592	0.835114568	0.083027607	0.002446947	2.817218685	0.166371009
ZNF593	0.650520776	1.017374307	0.78351725	1.321030879	0.897153422
ZNF594	0.172170585	0.885358542	0.372877784	2.102189461	0.782564507
ZNF595	0.426728244	3.032018115	0.519665591	17.69048021	0.217727127
ZNF596	0.613402845	1.149920217	0.146270399	9.040219459	0.894366895
ZNF597	0.785964192	0.958073886	0.221472327	4.144561013	0.954293788
ZNF598	0.258507913	1.361499892	0.743535221	2.493065431	0.31739594
ZNF599	0.134231046	0.733452487	0.020553055	26.17384872	0.865039615
ZNF600	0.642732856	1.070133683	0.170237887	6.726975515	0.942388601
ZNF605	0.366006275	11.55648197	0.002321775	57521.63521	0.573124411
ZNF606	0.48015609	1.890126134	0.136763017	26.12238952	0.634684498
ZNF607	0.292419385	1.020056138	0.697700567	1.491348258	0.918383938
ZNF608	0.407544453	0.893332505	0.509896459	1.565107875	0.693395455
ZNF609	0.738536761	0.122150959	0.000156304	95.46042874	0.536160565
ZNF610	0.206529079	0.95060973	0.616900866	1.464836425	0.818407073
ZNF611	0.561368017	20.48757887	0.00876528	47886.76502	0.445439081
ZNF613	0.683588609	2.73594863	0.000354027	21143.64068	0.825602751
ZNF614	0.419710557	0.000339066	7.98E-09	14.41179232	0.141753784
ZNF615	0.300075907	0.772624771	0.260260433	2.293660358	0.642178745
ZNF616	0.912699525	2.5244746	0.344993255	18.47274376	0.361802824
ZNF618	0.890600813	42.92549431	0.001323132	1392602.844	0.478092741
ZNF619	0.732157796	0.667335792	0.215369442	2.067782024	0.483334505
ZNF620	0.869618999	0.63812695	0.163230579	2.494667394	0.518416205
ZNF621	0.543594728	0.150674331	0.001996519	11.37117127	0.390924736
ZNF622	0.477447957	0.148896634	0.001622158	13.66710433	0.408847456
ZNF623	0.819249819	2.969854304	0.388402738	22.70847687	0.294281383
ZNF624	0.079170064	1.174019141	0.868279594	1.587416026	0.297260951
ZNF625	0.228872859	0.626972781	0.136712605	2.875337418	0.547981219
ZNF626	0.16515213	132.8890461	0.021689942	814179.1685	0.2717916

ZNF627	0.458996074	1.366491684	0.20394467	9.155912343	0.747652299
ZNF628	0.273347045	2.768026416	0.54535249	14.04957414	0.219291417
ZNF629	0.199417405	1.166668209	0.793968081	1.714319182	0.432432064
ZNF630	0.931802041	1.103451087	0.739350288	1.646857142	0.629914849
ZNF638	0.829261094	1.125483625	0.491648627	2.576460747	0.779666652
ZNF639	0.387895323	0.753225959	0.398699963	1.42299824	0.382603145
ZNF641	0.861252989	1.017142022	0.835578799	1.238157184	0.865464622
ZNF644	0.347276589	1.113184136	0.606083991	2.044566328	0.729588002
ZNF645	0.931680862	1.482123721	0.545934051	4.023729095	0.44000956
ZNF646	0.938291631	4.410111308	0.0073462	2647.502483	0.649385831
ZNF648	0.066307362	19.1916627	0.301867636	1220.137152	0.163140206
ZNF649	0.876133858	1.688286979	0.621320757	4.587506359	0.304491438
ZNF652	0.587893719	1.548817682	0.049089048	48.86703461	0.803805196
ZNF653	0.643068616	0.694696216	0.068716492	7.023100554	0.757614209
ZNF654	0.383557921	2.04242182	0.737301415	5.65777687	0.169527024
ZNF655	0.677932963	0.865564424	0.675108186	1.109750686	0.254845475
ZNF658	0.076726887	1.093171861	0.758797991	1.574891778	0.63249225
ZNF660	0.22552363	0.363432623	0.023335879	5.660094162	0.469964195
ZNF662	0.913918999	1.22672567	0.083112718	18.10620448	0.881723119
ZNF664	0.528629799	1.113912359	0.160923249	7.710512638	0.912974993
ZNF665	0.176285805	69.40879983	0.251730761	19137.8339	0.139179871
ZNF667	0.813305403	1.136105435	0.261997072	4.92652666	0.864630267
ZNF668	0.353893132	9.429438877	0.019457722	4569.615883	0.476936067
ZNF669	0.292944786	73.30984419	0.001376187	3905235.525	0.439262223
ZNF670	0.504848771	0.620148828	0.273643322	1.405422818	0.252361531
ZNF671	0.311062095	2.132697542	0.070929637	64.12550557	0.662720277
ZNF672	0.334134589	1.007080084	0.285827594	3.548328839	0.991239805
ZNF675	0.573502618	13.07069041	0.000450492	379236.2787	0.623939611
ZNF676	0.830724161	0.552378311	0.000875223	348.6217353	0.856820139
ZNF677	0.288971825	0.005199336	6.46E-06	4.184621863	0.123403641
ZNF678	0.526428588	8.184185734	0.44594955	150.1983713	0.156772175
ZNF679	0.414848473	1.662237798	4.52E-05	61133.48184	0.924520125
ZNF680	0.409805026	0.052028652	0.000264826	10.22173086	0.272567417

ZNF681	0.686792982	0.394543347	0.00546017	28.50908367	0.670204563
ZNF682	0.40168631	2.063374386	0.028119474	151.4080201	0.741026058
ZNF683	0.41909274	0.687024568	0.247296484	1.908651309	0.471489798
ZNF684	0.700127627	3.528982359	0.292406905	42.59036384	0.321033077
ZNF687	0.847283863	219.2925638	0.010785094	4458860.562	0.286866523
ZNF688	0.89116945	0.843882739	0.244104512	2.9173491	0.788540528
ZNF689	0.270367668	0.970772748	0.221793507	4.248996027	0.968587597
ZNF69	0.790083048	1.917202293	0.421346747	8.723609863	0.399822697
ZNF691	0.239060087	2.546792069	0.717475762	9.040235488	0.148093975
ZNF692	0.845780097	1.976380915	0.361189942	10.81448033	0.432088398
ZNF695	0.073445348	0.615109483	0.29437043	1.285318219	0.196216083
ZNF696	0.799233666	0.7113636	0.233225172	2.1697408	0.549462555
ZNF699	0.686738098	1.020686373	0.732554914	1.422146863	0.903700313
ZNF7	0.084152583	0.001294319	1.89E-07	8.880305695	0.14009872
ZNF70	0.071545612	1.4843687	0.718586903	3.066226825	0.285908707
ZNF700	0.884438973	1.301740355	0.222811893	7.605195262	0.769667792
ZNF701	0.803704441	0.839330547	0.472945119	1.48955077	0.549536247
ZNF703	0.237011672	471.4719857	0.000465429	477593066.5	0.382936629
ZNF704	0.415855479	0.828897027	0.580269172	1.184054428	0.302347568
ZNF705A	0.890820835	1.189833974	0.411447952	3.440787297	0.748350862
ZNF706	0.701397475	2.5586495	0.000442974	14778.95524	0.831646581
ZNF707	0.054068471	56.1063188	0.016471796	191109.638	0.33180702
ZNF708	0.540064355	7.669175902	0.00812715	7237.009107	0.559947361
ZNF709	0.666969293	1.131832565	0.452580557	2.830534666	0.791167609
ZNF71	0.276580762	1.515501761	0.644348235	3.564447705	0.340718569
ZNF710	0.792255208	0.763638082	0.224025942	2.603016048	0.666481812
ZNF713	0.747959274	0.14100598	0.003368847	5.901926479	0.303863296
ZNF714	0.370059414	0.044466188	4.76E-05	41.56583019	0.372403315
ZNF717	0.480611407	1.140215469	0.426826559	3.04594756	0.793524466
ZNF720	0.053944191	3.176574242	0.288080734	35.02706963	0.345290402
ZNF721	0.083471825	2.090934748	0.826822355	5.287723645	0.119178215
ZNF74	0.853574399	4.748414962	0.886406011	25.43692662	0.068887811
ZNF740	0.471753393	0.713410562	0.266237956	1.911653163	0.501901291

ZNF75A	0.686156718	0.577804159	0.030361382	10.99612799	0.715170485
ZNF76	0.350215839	5.778704022	0.452255204	73.83755863	0.177173466
ZNF77	0.385017815	0.013881905	0.000147496	1.306529928	0.06508832
ZNF79	0.627677759	0.614902816	0.04906783	7.705771288	0.706185749
ZNF8	0.773406943	2.481334048	0.568349054	10.83316426	0.226828489
ZNF80	0.558452166	0.044651224	0.001012637	1.968850549	0.107553653
ZNF81	0.470396731	0.003113173	1.69E-07	57.21577243	0.249249449
ZNF83	0.57506882	1.364059951	0.671758092	2.769835709	0.390299863
ZNF84	0.662372403	1.054925277	0.747084324	1.489614095	0.761338064
ZNF85	0.823175849	2.453609766	0.666082625	9.038219369	0.177282219
ZNF90	0.583565251	1.71262822	0.228530325	12.83460051	0.600582034
ZNF91	0.917392127	0.929013926	0.070492598	12.24336877	0.955368781
ZNF92	0.073355468	2.245275678	0.571346341	8.823479753	0.246730047
ZNF93	0.092078972	1.266586373	0.843898493	1.900988156	0.253984757
ZNFX1	0.37525521	0.424861443	0.001924229	93.80755924	0.755917118
ZNHIT1	0.202389546	0.006244939	5.70E-06	6.839244616	0.155165279
ZNHIT2	0.209016253	0.937106354	0.294888609	2.977966228	0.912317175
ZNHIT3	0.090360905	1.281794555	0.836690518	1.963685792	0.25399148
ZNRD1	0.741839521	0.21817505	0.032348926	1.471466236	0.117974061
ZNRF1	0.230012197	1.980523776	0.449499057	8.726324034	0.366443554
ZNRF2	0.127145794	0.498329875	0.201844002	1.230319765	0.130926896
ZNRF3	0.083507753	1.18695236	0.850032433	1.657414294	0.314353251
ZNRF4	0.22036939	0.982131244	0.355150771	2.715978276	0.972285594
ZP1	0.647874845	1.338885961	0.472099466	3.797114262	0.5831949
ZP2	0.176469052	0.647255745	0.269214493	1.556156932	0.331084115
ZP3	0.340811423	0.727162959	0.255717989	2.067769933	0.550160099
ZP4	0.844616551	0.705964367	0.290561799	1.715248491	0.442052287
ZPBP	0.057789385	0.010247362	2.86E-05	3.667109595	0.126798919
ZPBP2	0.646626868	0.833697005	0.300003874	2.316805739	0.727247622
ZPLD1	0.475791376	1.005842606	0.855274682	1.182917451	0.943865295
ZRANB1	0.116456883	0.040023715	7.85E-05	20.41341709	0.31165849
ZRANB3	0.54587673	1.112893861	0.951736128	1.301340476	0.180188719
ZSCAN1	0.469282543	0.943217983	0.260698217	3.412605475	0.929003306

ZSCAN2	0.113754544	4.559667222	0.263894306	78.78368225	0.296660404
ZSCAN4	0.386554139	1.129626617	0.868294677	1.469612021	0.363899546
ZSWIM1	0.235850953	841.3920725	0.571755519	1238187.646	0.070335195
ZSWIM2	0.0952336	1.238920052	0.32771081	4.683772542	0.752193224
ZSWIM3	0.6630807	2.014420651	0.459471696	8.831644255	0.35304553
ZSWIM4	0.723101884	20.94222481	0.073944214	5931.184586	0.291020409
ZSWIM5	0.179416199	0.718825076	0.210035045	2.46011083	0.598946745
ZSWIM6	0.775096963	1.285949956	0.925320487	1.787129229	0.134200033
ZW10	0.544457857	0.101759925	2.66E-05	389.7953705	0.587244396
ZWILCH	0.350041492	2.705670266	0.505995984	14.46780571	0.244589677
ZWINT	0.365950802	0.021169535	1.61E-05	27.79181424	0.292624015
ZXDA	0.295320715	1.0404376	0.653349411	1.656862899	0.86738159
ZXDB	0.92691281	0.546569333	0.167463632	1.783897988	0.316857526
ZXDC	0.142261998	0.593301772	0.256761732	1.370948034	0.221837864
ZYG11A	0.09418529	0.809284833	0.01871509	34.99539367	0.912327979
ZYG11B	0.257654482	1.096454266	0.713601611	1.684710262	0.67434679
ZYX	0.046944591	1.42915276	0.998649584	2.045239536	0.050870008
ZZEF1	0.547658573	0.979815763	0.7858054	1.221726053	0.856273465
ZZZ3	0.13735414	2.010841772	0.6767798	5.974594149	0.208649445

Table S2. Potential prognostic genes identified in the training cohort (p-value < 0.05).

ID	KM_pvalue	HR	HR_95L	HR_95H	Cox_pvalue
AADAT	0.048440578	1.541487933	1.053239832	2.256072146	0.025954766
ANXA5	0.03557234	0.420397926	0.196286843	0.900388498	0.025748239
ARX	0.006810533	2.93007972	1.842841359	4.658766269	5.53E-06
ATF4	0.04733989	2.388561762	1.172733539	4.864896501	0.01644083
ATP6V1E1	0.049105093	0.573283685	0.338769103	0.970142144	0.038181513
BBS4	0.000421943	0.270682686	0.106099794	0.690567945	0.006242367
BTAF1	0.018128901	2.161298445	1.160348572	4.025696316	0.015157217
C3orf14	0.020953361	0.277249513	0.131871185	0.582896803	0.000715463
CLTC	0.002847646	0.260082797	0.114638214	0.590056833	0.001272619
CORT	0.01449994	1.76460989	1.347616492	2.310633687	3.64E-05
CPE	0.002543812	1.563003882	1.198971121	2.03756462	0.000962212
CTNNBIP1	0.00542909	0.159994864	0.073127071	0.350053079	4.48E-06
CYP2C8	0.007170617	2.642643692	1.163316098	6.003153994	0.020269688
DDX26B	0.016376302	2.622709369	1.143883329	6.013379386	0.022757025
DLEU2	0.003123082	2.236812115	1.231254809	4.063601133	0.00821937
EI24	0.019314314	2.081166706	1.038750879	4.169676238	0.038715807
EIF4E3	0.01892268	0.308127555	0.121625778	0.780612395	0.013057994
FAM81A	0.012761385	1.743021047	1.00547449	3.021580757	0.047769431
FBXL5	0.00906649	0.189770359	0.077269495	0.466067355	0.000288681
GALNT14	0.000496403	1.543123009	1.176455246	2.024070724	0.001724692
GNG12	0.017726109	0.677162098	0.480124461	0.955061747	0.026280821
HMGCL	0.02887385	0.410662316	0.182639787	0.92336692	0.031332581
IFNGR1	0.032615795	0.379721423	0.164515537	0.8764422	0.023268251
LAMP2	0.008224709	0.567353305	0.331883098	0.969889018	0.038291613
LHPP	0.024523477	0.414822177	0.197755603	0.870152027	0.019915081
MCAM	0.048314168	1.759337456	1.206296372	2.565926878	0.003345769
MKL2	0.010080595	0.175027348	0.064512392	0.474863377	0.000620698
MLLT3	0.035433415	2.304329699	1.097496741	4.838224262	0.027399106
MPP7	0.02514617	2.270620208	1.09195997	4.721524843	0.028128203
MYC	0.025914635	2.214087845	1.479799429	3.312736098	0.000110504
NDUFB10	0.007731678	0.447053773	0.222423277	0.898543885	0.023801496
NID2	0.01804926	0.712964466	0.526529821	0.965412233	0.028701387
NPC2	0.002741643	0.611409875	0.418744999	0.89272	0.010846857
OR5AR1	0.008062474	2302.172041	1.990801321	2662242.61	0.03145251
PLCB1	0.033105954	0.327876985	0.116934772	0.919344312	0.034020637
PLCB4	0.010465829	1.899903349	1.224044305	2.94893961	0.004220247
PLD3	0.001897011	0.40600305	0.219443529	0.75116581	0.004086015
RAB8B	0.002763785	0.484731937	0.25455773	0.923032472	0.027546263
RBM34	0.006276627	2.346832964	1.215157168	4.532438361	0.011077108
RNF139	0.01592878	1.85578807	1.212763367	2.839753783	0.004389672
RPL37A	0.000638223	1.792496523	1.188893292	2.702550183	0.005337917
RPS28	0.010971169	1.664992583	1.119504624	2.476274095	0.011823506
SELPLG	0.011190205	0.635989475	0.45094155	0.896973482	0.009887602
SLC16A3	0.048372965	1.414208004	1.008362304	1.983398497	0.044620092
SLC45A4	0.00192743	0.305185519	0.11989515	0.776830428	0.012784233
SLC7A1	0.009278499	2.116380897	1.225514858	3.65484602	0.007155879
SLC8A3	0.019711469	1.419669828	1.097146208	1.837004409	0.00769733

SMAD9	0.042806027	1.624726378	1.116978468	2.363282622	0.011129406
TLL1	0.014730977	0.375210947	0.148217075	0.94984505	0.038588649
UAP1	0.003468966	1.518662549	1.027749174	2.244064988	0.035961955
UBE2L3	0.000195536	0.425488322	0.206565374	0.876431073	0.02046515
UHRF2	0.005393576	2.731663526	1.576974661	4.731836092	0.000337123
WDR53	0.022466366	0.316787857	0.113376951	0.885140634	0.02832948
WDR75	0.027433692	2.612816082	1.151120235	5.930577596	0.021648671
ZDHHC23	0.043262671	2.657053333	1.490211566	4.737536989	0.000926412
ZFP42	0.043037077	1.71787187	1.139339155	2.590171458	0.009806152
ZFP90	0.048226782	0.203007104	0.076698513	0.537323121	0.001323988

Table S3. Correlations of risk score with 15 immune relevant signatures by Pearson coefficient.

ID	r	p-value
PRF1	-0.2814	0.00908
CD8A	-0.2725	0.01163
HAVCR2	-0.2714	0.01198
TBX2	0.25904	0.01667
LAG3	-0.2494	0.02134
CD274	-0.249	0.02154
GZMA	-0.245	0.02381
GZMB	-0.134	0.22162
TNF	-0.1326	0.22645
PDCD1	-0.1306	0.23349
CTLA4	-0.1294	0.2379
IFNG	-0.1223	0.26495
CXCL10	-0.1199	0.27443
IDO1	-0.0518	0.63806
CXCL9	-0.0198	0.85729

Table S4. Correlations that determined between the seventeen-gene signature and the 22 TICs by the Pearson coefficient.

TIC	r	p-value
T cells CD8	-0.232299536	0.032407859
Mast cells activated	0.332147573	0.001899048
T cells CD4 memory activated	-0.240915367	0.02634645
T cells regulatory (Tregs)	-0.260169308	0.016185979
T cells CD4 naive	0.144665678	0.186513248
Dendritic cells resting	0.239545108	0.027240681
Macrophages M0	0.172850956	0.113662359
Dendritic cells activated	0.186513392	0.087424931
Monocytes	-0.16660658	0.127517092
Plasma cells	-0.09730241	0.3756682
T cells gamma delta	0.048139264	0.661745519
NK cells resting	0.150344513	0.169626476
B cells naive	-0.100415655	0.360510364
Macrophages M2	-0.029110496	0.791419525
B cells memory	0.033112018	0.763536374
T cells follicular helper	-0.040359055	0.713818753
Mast cells resting	0.002120241	0.984635091
T cells CD4 memory resting	-0.024686206	0.8225551
Neutrophils	-0.031079698	0.777662696
NK cells activated	-0.024116451	0.826585823
Eosinophils	-0.078758877	0.473692656
Macrophages M1	0.044564205	0.685492506

Table S5. The prognostic ability of 22 TICs that tested by the Kaplan-Meier estimator.

TIC	P-value
B cells naive	0.348294868
B cells memory	0.814319965
Plasma cells	0.581366016
T cells CD8	0.021726632
T cells CD4 naive	0.008624662
T cells CD4 memory resting	0.36425876
T cells CD4 memory activated	0.10030296
T cells follicular helper	0.254635984
T cells regulatory (Tregs)	0.674650397
T cells gamma delta	0.184566798
NK cells resting	0.518362195
NK cells activated	0.890663643
Monocytes	0.329225779
Macrophages M0	0.885003231
Macrophages M1	0.534206839
Macrophages M2	0.802931997
Dendritic cells resting	0.093454413
Dendritic cells activated	0.635381386
Mast cells resting	0.641581025
Mast cells activated	0.000124234
Eosinophils	0.555229953
Neutrophils	0.470803973
